# Site-Selective C_sp^3^_–C_sp_/C_sp^3^_–C_sp^2^_ Cross-Coupling Reactions Using Frustrated Lewis Pairs

**DOI:** 10.1021/jacs.1c01622

**Published:** 2021-03-15

**Authors:** Ayan Dasgupta, Katarina Stefkova, Rasool Babaahmadi, Brian F. Yates, Niklaas J. Buurma, Alireza Ariafard, Emma Richards, Rebecca L. Melen

**Affiliations:** † Cardiff Catalysis Institute, School of Chemistry, Cardiff University, Main Building, Park Place, Cardiff CF10 3AT, Cymru/Wales, United Kingdom; ‡ School of Natural Sciences-Chemistry, University of Tasmania Private Bag 75, Hobart, Tasmania 7001, Australia

## Abstract

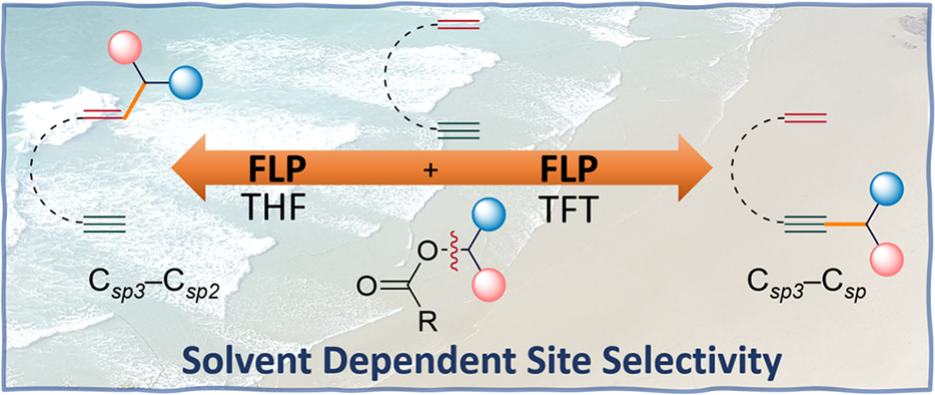

The donor–acceptor ability of frustrated Lewis pairs (FLPs) has led to widespread applications in organic synthesis. Single electron transfer from a donor Lewis base to an acceptor Lewis acid can generate a frustrated radical pair (FRP) depending on the substrate and energy required (thermal or photochemical) to promote an FLP into an FRP system. Herein, we report the C_sp^3^_–C_sp_ cross-coupling reaction of aryl esters with terminal alkynes using the B(C_6_F_5_)_3_/Mes_3_P FLP. Significantly, when the 1-ethynyl-4-vinylbenzene substrate was employed, the exclusive formation of C_sp^3^_–C_sp_ cross-coupled products was observed. However, when 1-ethynyl-2-vinylbenzene was employed, solvent-dependent site-selective C_sp^3^_–C_sp_ or C_sp^3^_–C_sp^2^_ cross-coupling resulted. The nature of these reaction pathways and their selectivity has been investigated by extensive electron paramagnetic resonance (EPR) studies, kinetic studies, and density functional theory (DFT) calculations both to elucidate the mechanism of these coupling reactions and to explain the solvent-dependent site selectivity.

## Introduction

**Scheme 1:**
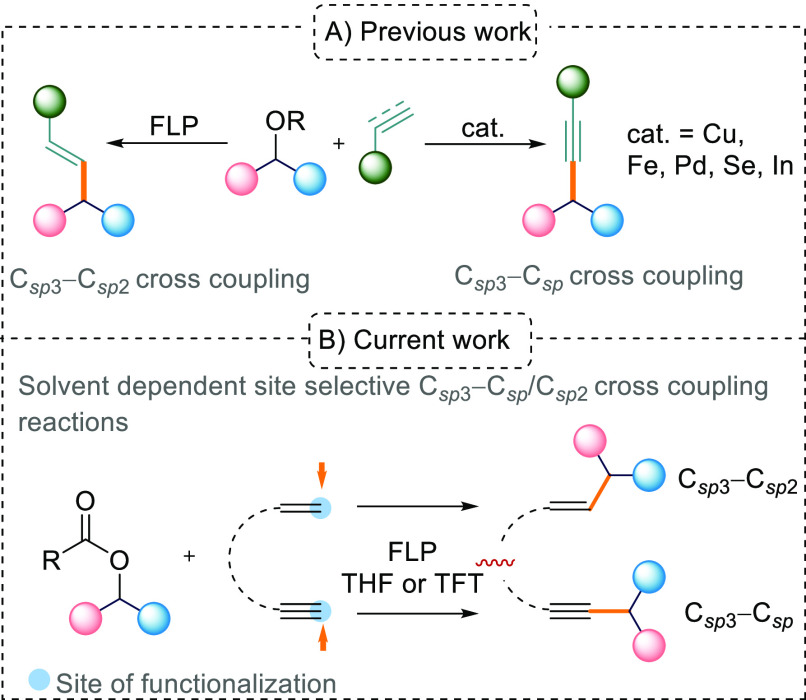
(A) Previous Work on Metal-Catalyzed C_sp^3^_–C_sp_ Cross-Coupling Reactions and FLP-Mediated C_sp^3^_–C_sp^2^_ Cross Coupling and (B) This Work on FLP-Mediated, Solvent-Dependent, Site-Selective C_sp^3^_–C_sp_/C_sp^3^_–C_sp^2^_ Cross-Coupling Reactions

Frustrated Lewis pairs (FLPs) have garnered much attention over the last two decades, with numerous FLP systems being reported in the literature.^1^ The cooperative reactivity of the Lewis acidic and basic components has led to a plethora of different small-molecule activation reactions^2^ and catalysis.^3^ Current studies have focused on using FLP systems as alternatives or complementary systems to the use of transition-metal catalysts in organic synthesis.^4^ Recently, new reactivities of FLPs have been disclosed, indicating that Lewis acids and bases undergo single electron transfer (SET) events^5^ depending on the energy required (thermal or photochemical) to promote the FLP into a frustrated radical pair (FRP) system. In these instances, an electron is transferred from the donor Lewis base (LB) to the acceptor Lewis acid (LA) to generate a reactive FRP. Indeed, we and others have postulated that such radical reactivity may be taking place in the reactions of the B(C_6_F_5_)_3_/Mes_3_P FLP with certain substrates.^6^ The radical reactivity of FLPs has the potential to open up new opportunities for metal-free synthesis. In a previous study of the B(C_6_F_5_)_3_/Mes_3_P FLP with diaryl esters and alkenes, we observed C_sp^3^_–C_sp^2^_ coupling reactions. We proposed a radical mechanism for the reaction based on the observation of [Ar_2_CH]^•^ and [Mes_3_P]^•+^ in electron paramagnetic resonance (EPR) studies (Scheme 1A).

In this current study, we were interested in the reactions of FLPs with alkynes in the presence of aryl esters (Scheme 1B). The 1,2-trans-addition of the Lewis acidic and basic components of FLPs to alkynes is well established^7^ and has also been employed in catalytic transformations.^8^ Interestingly, when using terminal acetylenes such as phenylacetylene (PhC≡CH) with stronger bases such as *t*Bu_3_P or TMP, deprotonation occurs instead of 1,2-addition to the alkyne generating [LBH][PhC≡CB(C_6_F_5_)_3_] salts.^7c^ In transition-metal chemistry, the activation of terminal alkynes for cross-coupling reactions is commonplace in the synthetic chemist’s toolbox to construct carbon–carbon bonds.^9^ For example, palladium- or copper-catalyzed Sonogashira cross-coupling reactions of terminal alkynes with aryl or alkenyl halides have been used for C_sp^3^_–C_sp^2^_ coupling.^10^ In this study, we are interested in the less well reported C_sp^3^_–C_sp_ coupling reactions. Typically, palladium^11^ or earth-abundant metal catalysts such as iron^12^ and copper^13^ are employed for these reactions, although examples are known with other elements such as indium^14^ as well as stoichiometric reactions using Brønsted^15^ and Lewis acids.^16^

Herein, we report the high reactivity of frustrated Lewis pairs in selective C_sp^3^_–C_sp_ coupling reactions between aryl esters and terminal alkynes or 1-ethynyl-4-vinylbenzene. We also report solvent-dependent site selectivity when using 1-ethynyl-2-vinylbenzene as a substrate leading to selective C_sp^3^_–C_sp_ or C_sp^3^_–C_sp^2^_ cross-coupling depending upon the solvent employed.

## Results and Discussion

### Reaction Optimization and Development

Initially, the FLP-mediated C_sp^3^_–C_sp_ cross-coupling reaction between bis(4-fluorophenyl)methyl-4-fluorobenzoate (**1a**) and phenylacetylene was investigated using a range of reaction conditions (Table 1). As expected, no reaction occurred in the absence of an FLP (Table 1, entry 1). Reaction with only the Lewis base component of the FLP (Mes_3_P) showed no cross-coupled product, and a stoichiometric or catalytic (10 mol %) amount of the Lewis acid B(C_6_F_5_)_3_ led to only 22 and 5% isolated yields of the desired C_sp^3^_–C_sp_ cross-coupled product, **2a** (Table 1, entries 3 and 4). Stoichiometric amounts of both a Lewis acid and Lewis base were required for the C_sp^3^_–C_sp_ coupling reaction to attain satisfactory yields of the cross-coupled products. The B(C_6_F_5_)_3_/Mes_3_P FLP in toluene at 70 °C led to the C_sp^3^_–C_sp_ cross-coupled product, **2a**, being formed in 54% yield (Table 1, entry 5). The optimum temperature was 70 °C with both lower (21 °C) and higher (110 °C) temperatures giving reduced yields (18 and 45% respectively) (Table 1, entries 6 and 7). More polar THF gave the highest yield of 83%, with trifluorotoluene (TFT) giving a 71% yield. CH_2_Cl_2_ and hexane, on the other hand, showed poorer or low yields (Table 1, entries 8–11). Interestingly, other Lewis acid boranes (BF_3_·OEt_2_ and BPh_3_) as well as Brønsted acids (CF_3_SO_3_H) showed no product formation when combined with Mes_3_P (Table 1, entries 12–14). Other basic phosphines including *t*Bu_3_P, Ph_3_P and *o-*tol_3_P had complicated reaction mixtures with no or moderate yields of **2a** being formed (Table 1, entries 15–17). Nitrogen Lewis bases including TMP (2,2,6,6-tetramethylpiperidine), DABCO (1,4-diazabicyclo(2,2,2)octane), and DMA (4-bromo dimethyl aniline) led to no product formation (Table 1, entries 18–20). The optimum conditions for the reaction were therefore chosen to be the use of the B(C_6_F_5_)_3_/Mes_3_P FLP in THF at 70 °C for 22–24 h.

**Table 1:** Optimization of the Reaction Conditions for C_sp^3^_–C_sp_ Cross-Coupling Reactions^a^

entry	LA	LB	solvent	temp (°C)	yield (%)
1			toluene	70	0
2		Mes_3_P	toluene	70	0
3	B(C_6_F_5_)_3_		toluene	70	22
4^b^	B(C_6_F_5_)_3_		toluene	70	5
5	B(C_6_F_5_)_3_	Mes_3_P	toluene	70	54
6	B(C_6_F_5_)_3_	Mes_3_P	toluene	21	18
7	B(C_6_F_5_)_3_	Mes_3_P	toluene	110	45
8	B(C_6_F_5_)_3_	Mes_3_P	THF	70	83
9	B(C_6_F_5_)_3_	Mes_3_P	TFT	70	71
10	B(C_6_F_5_)_3_	Mes_3_P	CH_2_Cl_2_	45	38
11	B(C_6_F_5_)_3_	Mes_3_P	hexane	70	0
12	BF_3_·OEt	Mes_3_P	toluene	70	0
13	BPh_3_	Mes_3_P	toluene	70	0
14	CF_3_SO_3_H	Mes_3_P	THF	70	0
15	B(C_6_F_5_)_3_	*t*Bu_3_P	toluene	70	45
16	B(C_6_F_5_)_3_	Ph_3_P	THF	70	0
17	B(C_6_F_5_)_3_	*o-*tol_3_P	THF	70	0
18	B(C_6_F_5_)_3_	TMP	THF	70	0
19	B(C_6_F_5_)_3_	DABCO	THF	70	0
20	B(C_6_F_5_)_3_	DMA	THF	70	0

a All of the reactions were carried out on a 0.1 mmol scale, and reported yields are isolated. All reactions were carried out for 20–22 h.

b 10 mol % B(C_6_F_5_)_3_.

### Reaction Scope

**Scheme 2:**
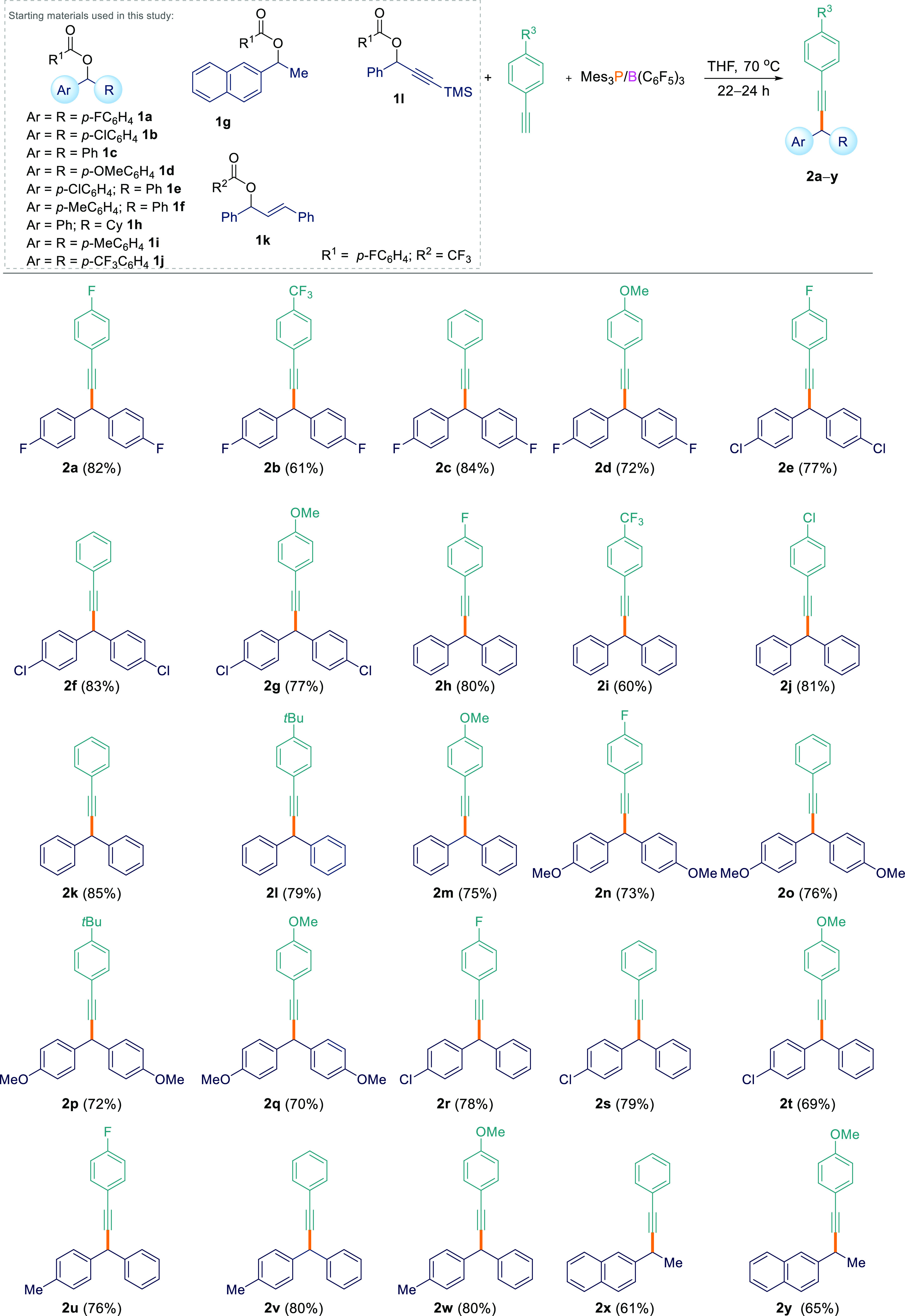
C_sp^3^_–C_sp_ Cross-Coupling Reactions between Esters **1** and Acetylenes All reactions were performed on a 0.1 mmol scale.

With the optimized reaction conditions in hand, several aryl esters (**1a**–**l**) were tested for the FLP-mediated C_sp^3^_–C_sp_ coupling reaction with various acetylenic compounds (Scheme 2). Diaryl esters bearing electron-withdrawing/π-releasing (*p*-F, **1a**; *p*-Cl, **1b**), neutral (*p*-H, **1c**), and electron-donating (*p*-OMe, **1d**) groups all worked well for the reactions when coupled with aryl-substituted terminal acetylenes with electron-releasing (*p*-OMe, *p*-*t*Bu), neutral (*p*-H), electron-withdrawing/π-releasing (*p*-F, *p*-Cl), and electron-withdrawing (*p*-CF_3_) groups generating products **2a**–**q** in 60–85% yields. Asymmetrical diaryl esters **1e** and **1f** were also found to undergo the C_sp^3^_–C_sp_ cross-coupling reaction, with several alkynes generating C–H-functionalized products **2r**–**w** in excellent isolated yields (69–80%). We could also use alkyl/aryl esters containing just one aryl-stabilizing group. **1g** could be cross-coupled with electron-neutral (phenylacetylene) and electron-releasing (4-ethynylanisole) alkynes to give **2x** and **2y** albeit in slightly lower yields of 61 and 65%, respectively. However, when cyclohexyl(phenyl)methyl-4-fluorobenzoate (**1h**) was employed, poor conversion was observed. Diaryl esters bearing strongly electron-withdrawing (*p*-CF_3_, **1j**) groups also failed to react at all with terminal alkynes. We attribute this to the electron-deficient nature of the ester which is not Lewis basic enough to form an adduct with the Lewis acidic borane in the initial step of the reaction as observed by *in situ*^11^B and ^1^H NMR spectroscopy. The unwillingness of ester **1j** to react with arylacetylenes can also be interpreted from DFT calculations (see later and SIFigure S180).

**Scheme 3:**
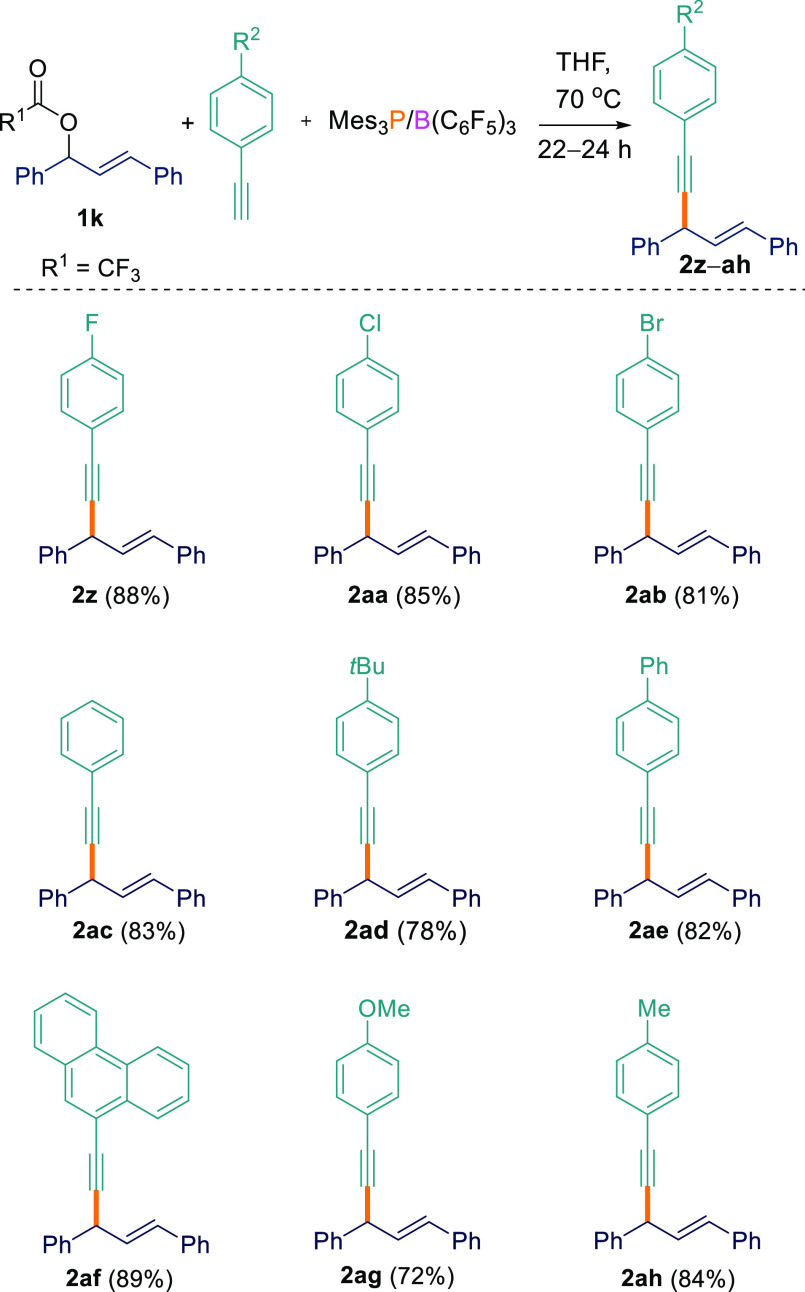
C_sp^3^_–C_sp_ Cross-Coupling Reactions between Ester **1k** and Acetylenes All reactions were performed on a 0.1 mmol scale.

After achieving good success for the C_sp^3^_–C_sp_ cross-coupling reaction at the benzylic position, we investigated a wider substrate scope (Scheme 3). To this end, allylic ester (*E*)-1,3-diphenylallyl-2,2,2-trifluoroacetate (**1k**) was used in the C–H functionalization. To our delight, excellent yields of products **2z**–**ah** (72–89%) were obtained. While benzylic and allenylic esters worked well, the same was not true for cross-coupling at the propargylic position. When the aryl/alkynyl ester 1-phenyl-3-(trimethylsilyl)prop-2-yn-1-yl 4-fluorobenzoate (**1l**) was employed with terminal acetylenes, an inseparable complicated reaction mixture resulted.

In our previous studies,^6^ we have shown that reactions of esters **1** with styrenes in the presence of the same FLP leads to C_sp^3^_–C_sp^2^_ coupled products. We therefore undertook an experiment to investigate the regioselectivity of the reaction by reacting the ester starting material with a 1:1 mixture of an acetylene and a styrene. For this reaction, three outcomes are theoretically possible: (i) formation of the C_sp^3^_–C_sp_ coupled product, (ii) formation of the C_sp^3^_–C_sp^2^_ coupled product, or (iii) formation of a mixture of C_sp^3^_–C_sp_ and C_sp^3^_–C_sp^2^_ coupled products. Using the optimized reaction conditions, an equimolar mixture of aryl ester **1a**, 4-fluorophenylacetylene, and 4-fluorostyrene were reacted together with B(C_6_F_5_)_3_/Mes_3_P (Table 2).

**Table 2:**
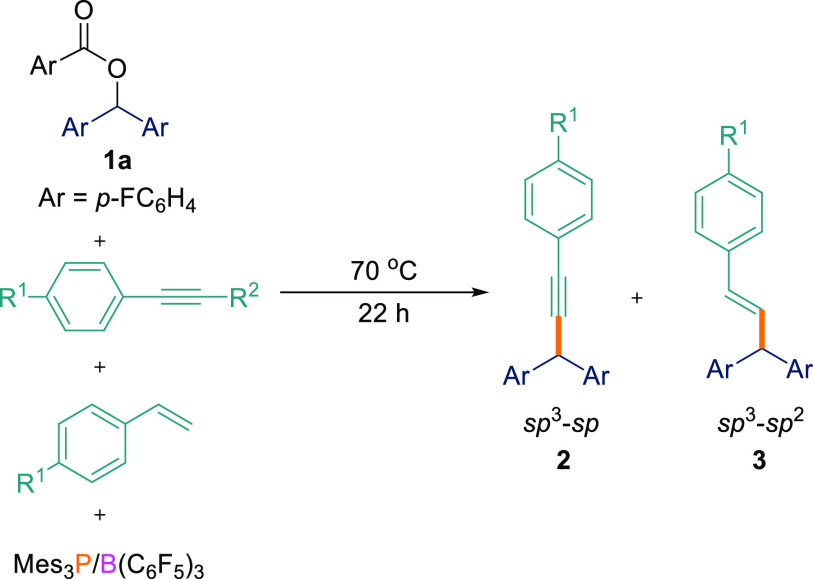
Selectivity Reactions between Esters **1a** and Acetylenes/Styrene^a^

entry	R^1^	R^2^	solvent	yield C_sp^3^_–C_sp_ (%)	yield C_sp^3^_–C_sp^2^_ (%)
1	F	H	THF	78 (**2a**)	n.d.
2	F	H	TFT	72 (**2a**)	n.d.
3	H	Ph	THF	n.d.	63 (**3a**)
4	H	TMS	THF	58 (**2c**)	18 (**3a**)

a 0.1 mmol scale, reported yields are isolated; n.d. = not detected.

The crude ^1^H NMR spectrum clearly showed a sharp singlet at δ = 5.09 ppm which confirmed the formation of the C_sp^3^_–C_sp_ cross-coupled product, **2a**, isolated as the major product in 78% yield (Table 2, entry 1). We were not able to detect any characteristic peaks (i.e., a doublet at δ = 4.98 ppm in the ^1^H NMR spectrum)^6^ for the C_sp^3^_–C_sp^2^_ coupled compound. To investigate the effect of solvent on the reaction, we also conducted the reaction in TFT, where again **2a** was isolated as the major product albeit with a slightly reduced yield of 72% (Table 2, entry 2). The observation of exclusive C_sp^3^_–C_sp_ coupling is presumably a consequence of the higher reactivity of the alkyne functionality over the alkene. A similar competition experiment, using a 1:1 mixture of styrene and the internal alkyne diphenylacetylene, gave only C_sp^3^_–C_sp^2^_ coupling producing **3a** in 63% yield (Table 2, entry 3). Interestingly, TMS-protected alkynes behaved in the same manner as terminal alkynes, predominantly giving the C_sp^3^_–C_sp_ coupled product with the loss of the TMS group. Using a 1:1 mixture of styrene and trimethyl(phenylethynyl)silane afforded the C_sp^3^_–C_sp_ coupled product (**2c**, 58% isolated) as the major product with small amounts of the C_sp^3^_–C_sp^2^_ cross-coupled product (**3a**, 18% isolated) formed (Table 2, entry 4). This was also observed by *in situ*^1^H NMR spectroscopy of the crude reaction mixture, which displayed a 1:0.4 ratio of C_sp^3^_–C_sp_ to C_sp^3^_–C_sp^2^_ cross-coupled products.

**Scheme 4:**
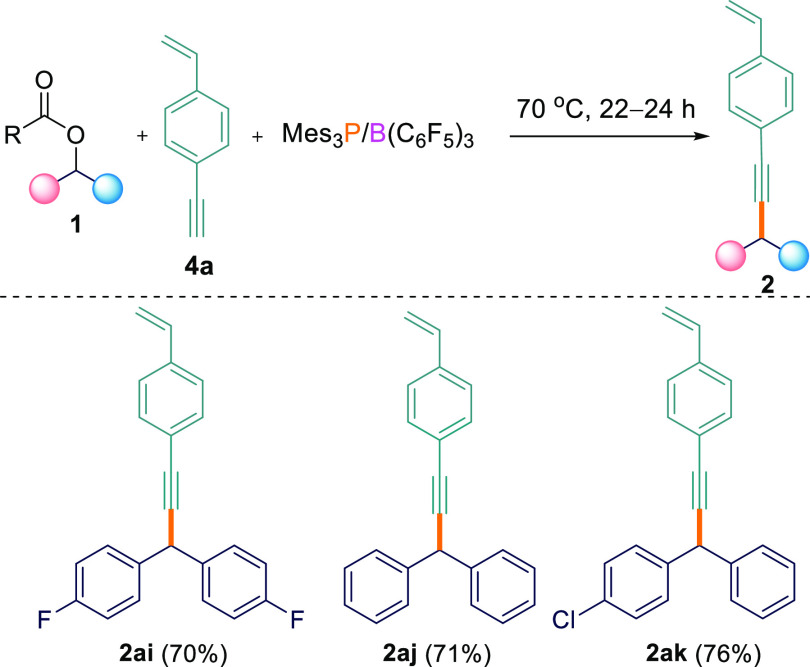
Cross-Coupling Reactions between Esters **1a**,**c**,**e** and 1-Ethynyl-4-vinylbenzene **4a** 0.1 mmol scale; reported yields are isolated.

To demonstrate the scope for this selectivity, we synthesized a substrate containing both alkene and alkyne functionalities, namely, 1-ethynyl-4-vinylbenzene (**4a**).^17^**4a** was subsequently reacted with different aryl esters (**1a**,**c**,**e**) in the presence of the B(C_6_F_5_)_3_/Mes_3_P FLP. In agreement with the observation above, we observed only the formation of the C_sp^3^_–C_sp_ compounds as the major product (**2ai**–**ak**; 70–76%) using the optimized reaction conditions (Scheme 4). In all cases, the C_sp^3^_–C_sp^2^_ coupled product was either not detected or was observed in <5% yield in both THF and TFT solvents.

**Scheme 5:**
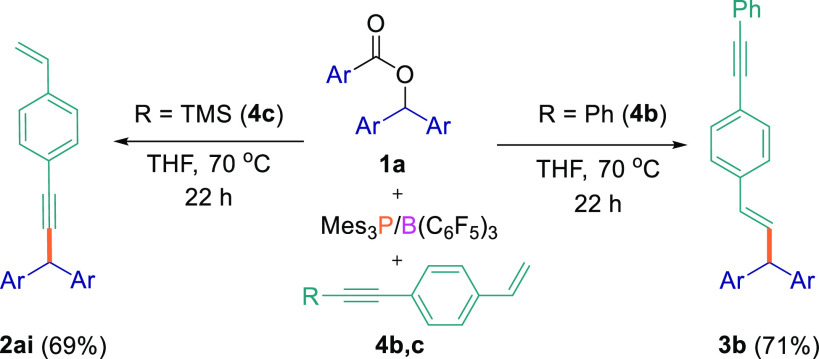
Cross-Coupling Reactions between Ester **1a** and Substrates **4** bearing Internal Alkynes All reactions were performed on a 0.1 mmol scale.

As for the intermolecular competition reactions, we also synthesized internal alkynes in which the acetylenic proton in **4a** was replaced by a phenyl or TMS group to explore how this affected the regioselectivity of the reaction (Scheme 5). Using the optimized reaction conditions, the reaction of aryl ester **1a** with 1-(phenylethynyl)-4-vinylbenzene (**4b**) exclusively gave the C_sp^3^_–C_sp^2^_ cross-coupled product in 71% isolated yield from the reaction with the alkene, whereas when **1a** was reacted with trimethyl{(4-vinylphenyl)ethynyl}silane (**4c**), reaction at the alkyne and removal of the TMS group *in situ* afforded the C_sp^3^_–C_sp_ cross-coupled product (**2ai**) in 69% yield, with no significant reaction at the alkene site observed.

**Scheme 6:**
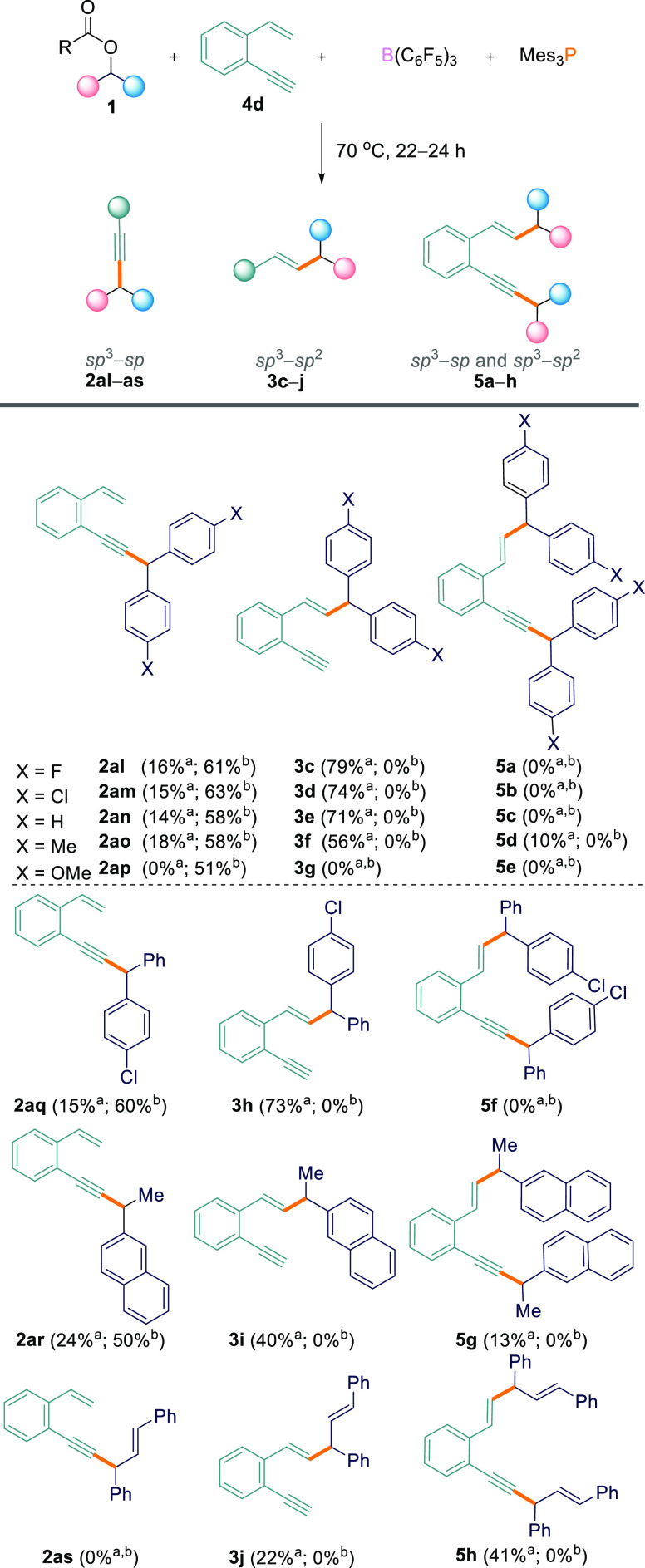
Products of Solvent-Dependent Site-Selective Cross-Coupling Reactions Reactions were carried out on a 0.1 mmol scale, and yields were isolated. ^a^Yield in THF. ^b^Yield in TFT.

With these results in hand, we further explored the substrate scope using the 1-ethynyl-2-vinylbenzene (**4d**).^18^**4d** was reacted with ester **1a** and the B(C_6_F_5_)_3_/Mes_3_P FLP under the optimized reaction conditions (THF, 70 °C, 24 h). Contrary to the reactions above, the reactions were found to be highly site-selective for the C_sp^3^_–C_sp^2^_ coupled product, **3**, from reaction at the alkene functional group. Examining the crude ^1^H NMR spectrum revealed a 0.2:1:0 ratio of the products (C_sp^3^_–C_sp_ coupled, **2**)/(C_sp^3^_–C_sp^2^_ coupled, **3**)/(C_sp^3^_–C_sp_ and C_sp^3^_–C_sp^2^_ coupled, **5**) (Table 3, entry 1) with isolated yields of 79% for the C_sp^3^_–C_sp^2^_ coupled product, **3c**, and 16% for the C_sp^3^_–C_sp_ coupled product, **2al** (Scheme 6). Interestingly, when changing the solvent to TFT, the selectivity was completely reversed, exclusively giving C_sp^3^_–C_sp_ product **2al** from reaction at the alkyne site.

**Table 3:** Solvent-Dependent Site-Selective Studies^a^

entry	ester	ratio of **2:3:5** in THF	entry	ester	ratio of **2:3:5** in TFT
1	**1a**	0.2:1:0	9	**1a**	1:0:0
2	**1b**	0.2:1:0	10	**1b**	1:0:0
3	**1c**	0.2:1:0	11	**1c**	1:0:0
4	**1i**	0.4:1:0.1	12	**1i**	1:0:0
5	**1d**	0:0:0	13	**1d**	1:0:0
6	**1e**	0.2:1:0	14	**1e**	1:0:0
7	**1g**	0.5:1:0.1	15	**1g**	1:0:0
8	**1j**	0:1:1.5	16	**1j**	0:0:0

a Ratios were determined from the crude ^1^H NMR spectra.

This was observed by crude ^1^H NMR, indicating a 1:0:0 ratio of **2**/**3**/**5** (Table 3, entry 9) in which **2al** could be isolated in 61% yield (Scheme 6). Remarkably, by simply changing the solvent we can switch the site selectivity of the reaction.

We next investigated this solvent-dependent site selectivity for a range of other esters and found the same general trend. In the following discussion, all reaction product ratios were determined via crude ^1^H NMR studies and are listed in Table 3, with the corresponding isolated yields for the products shown in Scheme 6. Initially, we explored the reactions in THF solvent. When electron-withdrawing **1b** (*p*-Cl) or electron-neutral symmetrical diaryl esters **1c** (*p*-H) and **1i** (*p*-Me) were used, there was a clear preference for reaction at the alkene site leading to C_sp^3^_–C_sp^2^_ coupled products **3d**, **3e**, and **3f** in ratios of 0.2:1:0, 0.2:1:0, and 0.4:1:0.1 for **2**/**3**/**5**, respectively (Table 3, entries 2–4). In all cases, the major and minor regioisomers could be separated. The C_sp^3^_–C_sp^2^_ cross-coupled products were isolated in 74% (**3d**), 71% (**3e**), and 56% (**3f**) yields, and the C_sp^3^_–C_sp_ cross-coupled products were isolated in 15% (**2am**), 14% (**2an**), and 18% (**2ao**) yields. In the case of **1i** as the starting material, double cross-coupled product **5d** was observed in small amounts and could be separated and isolated in 10% yield (Scheme 6). Electron-rich *p*-OMe ester **1d**, on the other hand, showed no reactivity at all in THF (Table 3, entry 5). Asymmetrical diaryl ester **1e** gave a ratio of 0.2:1:0 for **2**/**3**/**5** (Table 3, entry 6), with **2aq** and **3h** being isolated in 15 and 73% yields, respectively. Alkyl/aryl ester **1g** also gave the C_sp^3^_–C_sp^2^_ cross-coupled product as the major isomer, albeit less selectively, showing a 0.5:1:0.1 ratio of the three products **2ar**/**3i**/**5g** in isolated yields of 24% (**2ar**), 40% (**3i**), and 13% (**5g**) (Table 3, entry 7 and Scheme 6). 1,3-Diphenylallyl-2,2,2-trifluoroacetate (**1k**), on the other hand, showed a preference for the double cross-coupled product, giving a 0:1:1.5 ratio of **2**/**3**/**5** with isolated yields of 22% (**3j**) and 41% (**5h**) (Table 3, entry 8 and Scheme 6).

Subsequently, we repeated the above series of reactions in TFT, and remarkably, the regioselectivity was altered and the selectivity was improved. No C_sp^3^_–C_sp^2^_ coupled product (**3**) or double C_sp^3^_–C_sp_/C_sp^3^_–C_sp^2^_ coupled product (**5**) was observed with any combination of substrates. Rather, a ratio of 1:0:0 of products **2**/**3**/**5** was observed in all cases for esters **1a**–**e**, **1g**, and **1i** (Table 3, entries 9–15). This included *p*-OMe ester **1d** which did not show any product formation in THF. C_sp^3^_–C_sp_ cross-coupled products **2al**–**ar** could be isolated in 50–63% yields (Scheme 6). The only exception was 1,3-diphenylallyl 2,2,2-trifluoroacetate (**1k**), which gave a complex mixture of inseparable products when reacted with 1-ethynyl-2-vinylbenzene (**4d**) in TFT, none of which could be identified as **2**, **3**, or **5** (Table 2, entry 16).

### Proposed Reaction Mechanism

The C_sp^3^_–C_sp_ cross-coupling reaction could be explained by either a single- or a two-electron pathway.

**Scheme 7:**
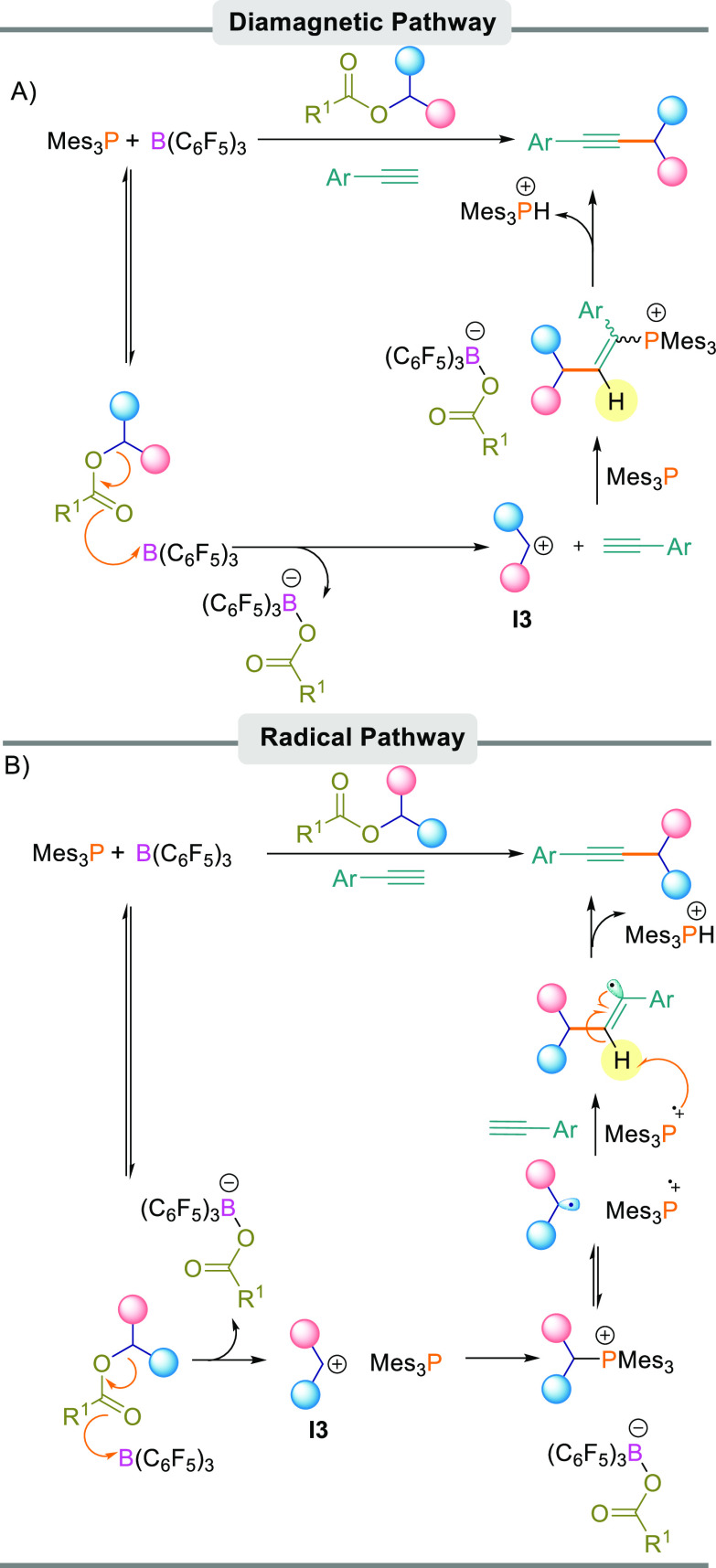
Possible Reaction Mechanisms (A) Diamagnetic pathway. (B) Radical pathway.

First, a diamagnetic pathway could operate (Scheme 7A) in which the Lewis acid component of the FLP activates the ester carbonyl atom, leading to the formation of diaryl methylene cation **I3** and the borate anion [R_1_CO_2_B(C_6_F_5_)_3_]−. This was observed in our previous studies when B(C_6_F_5_)_3_ was added to the diaryl ester in the presence of a nucleophile to trap the resultant carbocation.^19^ The diaryl methylene cation and the Lewis basic component of the FLP can then undergo a 1,2-FLP addition to the alkyne similar to other FLP 1,2-additions.^20^ Finally, the elimination of [Mes_3_PH]^+^ leads to the C–C-bonded product and salt [R^1^CO_2_B(C_6_F_5_)_3_][HPMes_3_] (R^1^ = *p-*FC_6_H_4_ or CF_3_). This can be observed in the reaction by multinuclear NMR spectroscopy showing a clear ^1^*J*_PH_ coupling of 479.5 Hz.

Alternatively, a radical pathway could operate (Scheme 7B), which may explain the necessity for using Mes_3_P as a Lewis base rather than other phosphine or nitrogen bases. Previous studies have postulated that the B(C_6_F_5_)_3_/Mes_3_P FLP can undergo a single electron transfer (SET) process generating radical ion pair [B(C_6_F_5_)_3_]^•–^/[Mes_3_P]^•+^.^21^ Slootweg et al. later postulated that this process is promoted upon irradiation with visible light (390–500 nm).^5b^ In this pathway (Scheme 7B), we propose that the first step of the reaction is identical to the diamagnetic pathway whereby B(C_6_F_5_)_3_ activates the diaryl ester to generate diaryl methylene cation **I3** and borate anion [R^1^CO_2_B(C_6_F_5_)_3_]^−^. The Lewis base then reacts with cation **I3**, forming a Lewis acid–base adduct. We propose that this adduct is in equilibrium with diaryl methylene radical [Ar_2_CH]^•^ and phosphonium radical cation [Mes_3_P]^•+^, which are formed from the homolytic cleavage of the C–P bond. From here, diaryl methylene radical [Ar_2_CH]^•^ adds to arylacetylene, leading to a vinylic radical species which, upon abstraction of a hydrogen atom by [Mes_3_P]^•+^, generates the desired C–C bonded product.

To understand which pathway is operating, we undertook extensive electron paramagnetic resonance (EPR), kinetic, and density functional theory (DFT) studies to understand the reaction mechanism for the C_sp^3^_–C_sp_ coupling reaction.

### EPR Studies

**Figure 1:**
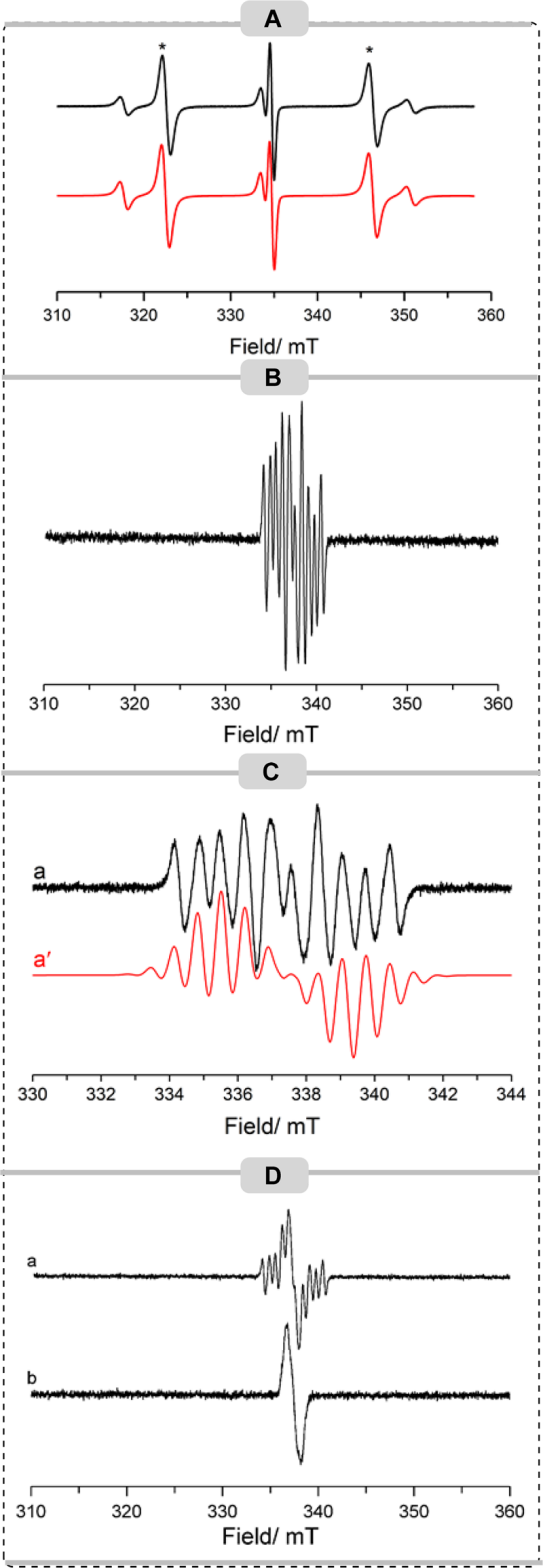
CW X-band EPR spectra (*T* = 298 K) of (A) FLP + ester **1d**, (B) FLP + phenylacetylene, (C) FLP + phenylacetylene (black, experimental; red, simulated), and (D) FLP + phenylacetylene + ester recorded at (a) *t* = 0 min and (b) after storage at 77 K for 30 min. TFT was used as the solvent for all EPR measurements.

The knowledge that the B(C_6_F_5_)_3_/Mes_3_P FLP can generate radical species prompted us to undertake an EPR study to determine if radical species could be observed in these reactions. As reported previously, no EPR signal could be detected from the B(C_6_F_5_)_3_/Mes_3_P FLP in the absence of any substrates.^21^ Upon addition of an equimolar ratio of ester **1d** to the FLP in a TFT solution, several EPR signals arising from multiple paramagnetic species were detected at room temperature (Figure 1A). Upon comparison with previous reports, the two intense resonance lines in a 1:1 ratio (marked with asterisks) centered on *g*_i__so_ = 2.012 (*B* ≈ 335 mT) and separated by a phosphorus hyperfine splitting of *a*_iso_(^31^P) = 670 MHz (23.8 mT) are attributed to the formation of the [Mes_3_P]^•+^ cation.^22^ A second paramagnetic species was also detected in this sample, which is characterized by a 1:2:1 triplet centered on *g*_iso_ = 2.010 and separated by a hyperfine splitting of 470 MHz (16.7 mT). This EPR profile must originate from two identical *I* = 1/2 nuclei, in this case associated with two equivalent ^31^P nuclei. The signal is therefore tentatively assigned to the formation of a [(P(Mes)_*n*=2,3_)_2_]^•+^ dimer formed from the association of excess Mes_3_P with radical cation [Mes_3_P]^•+^ as observed previously.^23^ This assignment is supported by our observation that the relative ratios of the EPR signals of [Mes_3_P]^•+^/[(P(Mes)_*n*=2,3_)_2_]^•+^ are inter-related (i.e., when a large signal intensity of the monomer is observed, only a trace of the corresponding dimer is detected). The varying ratio of these EPR signals under different reaction conditions demonstrates the conversion between monomer and dimer via the reaction of the monomer with a second molecule of phosphine to yield the dimer radical cation, as previously observed for a series of phosphines,^24^ and possibly also the varying stabilities of the two radical species. The EPR spectrum of [(PMes_2_)_2_]^•+^ has previously been reported,^25^ characterized by *g*_iso_ = 2.006 and *a*_iso_(^31^P) = 474 MHz (17 mT), and it is noted that literature examples of phosphine dimer cation radicals of divalent (R_2_P)_2_^•+^ and trivalent (R_3_P)_2_^•+^ systems yield very similar EPR spectra,^26^ dominated by the 1:2:1 phosphorus hyperfine splitting.

The corresponding anisotropic EPR spectra of the [Mes_3_P]^•+^ monomer and [P(Mes)_*n*=2,3_]_2_^•+^ dimer species in frozen solutions are shown in the SI (Figure S176), from which the principal values of the **g** and **A** tensors were determined. The spin Hamiltonian parameters for all of the paramagnetic species detected in this work are listed in the SI (Table S1). Importantly, contrary to our previous reports,^6^ no evidence for the formation of the carbon-based bismethoxy-diphenylmethylene radical formed upon C–O bond cleavage was observed in this case, perhaps due to the instability of the radical species.

We then probed the room-temperature EPR spectrum of the FLP in the presence of phenylacetylene (Figure 1B,C). Under these experimental conditions, we postulated that another possible mechanism could be the abstraction of the terminal hydrogen atom of the acetylene by the [Mes_3_P]^•+^ radical cation to form the diamagnetic [Mes_3_PH]^+^ cation and the corresponding phenylacetylene radical. As can be seen from the wide field scanning range in Figure 1B, no evidence of monomer [Mes_3_P]^•+^ or dimer [P(Mes)_*n*=2,3_]_2_^•+^ was observed in this solution, suggesting the formation of the diamagnetic [Mes_3_PH]^+^ cation. However, no EPR evidence for the generation of the phenylacetylene radical was obtained. It is noted in previous literature studies that the terminal phenylacetylene radical is inherently unstable and is typically observed only via EPR spectroscopy under controlled conditions, such as neat liquids sealed under vacuum, or via matrix isolation methods.^27^ Notably, the remaining boron component of the FLP is not involved in the above hydrogen atom abstraction from the alkyne and may be expected to remain in solution. The intense multiplet signal observed, reproduced in Figure 1C across a narrow field range, is therefore assigned to the boron radical anion, [B(C_6_F_5_)_3_]^•–^. A satisfactory simulation of the experimental data was achieved using *g*_iso_ = 2.0114 and incorporating a single boron nucleus, *a*_*i*__so_(^10,11^B) = 27 MHz, two sets of six equivalent fluorine nuclei from the ortho and meta positions, with *a*_i__so_(^19^F)_ortho_ = 18.28 MHz and *a*_i__so_(^19^F)_meta_ = 3 MHz, and three para fluorine nuclei with *a*_i__so_(^19^F)_para_ = 20.2 MHz, which agrees well with previous literature reports of this species.^28^ The corresponding anisotropic spectrum for this sample was unfortunately not resolved due to a poor-quality glass of the frozen solvent, thereby preventing the determination of the complete anisotropic spin Hamiltonian parameters for this radical anion.

Having determined the reactivity of the FLP to the individual substrates, the EPR spectrum of a full reaction mixture containing equimolar amounts of Mes_3_P, B(C_6_F_5_)_3_, ester, and phenylacetylene was recorded (Figure 1Da). As can easily be seen, evidence of the [B(C_6_F_5_)_3_]^•–^ radical anion is clearly observed, but there are no signals attributed to monomer or dimer phosphorus radicals present. However, it is noticed that there is additional intensity superimposed in the center of this signal (*g*_iso_ ≈ 2.001) which must originate from a second paramagnetic species not previously observed. Upon storage of this sample at 77 K for 30 min, the signal intensity originating from the [B(C_6_F_5_)_3_]^•–^ radical anion was lost completely, leaving only a narrow resonance (Figure 1Db). Unfortunately, the short lifetime of this radical in solution prevented full resolution of the hyperfine coupling, but the narrow spectral width arising from only small hyperfine couplings is an indication of a carbon-based radical rather than a boron or phosphorus species (upon consideration of the theoretical isotropic hyperfine *a*_0_ values *a*_0_(^10^B) = 30.43 mT, *a*_0_(^11^B) = 90.88 mT, and *a*_0_(^31^P) = 474.79 mT). The experimental spectrum is reproduced again in the SI (Figure S177) alongside simulations of the styrene and phenylacetylene radicals, using previously reported literature values.^29^ Gratifyingly, there is reasonable agreement between the experimental and simulated data, thereby this signal is tentatively attributed to a carbon-based radical, perhaps indicating that the reaction could be occurring through a radical mechanism.

### DFT and Kinetic Studies

**Scheme 8:**
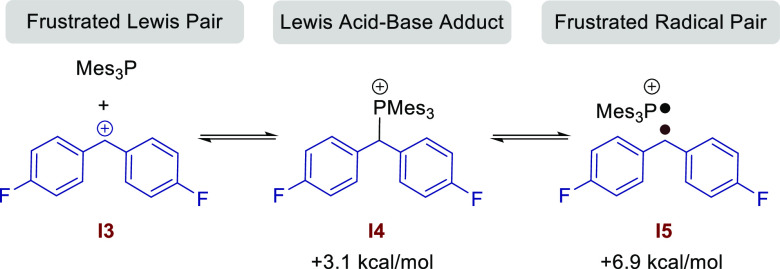
Mes_3_P/Diaryl Methylene Cation Equilibria in Solution

**Figure 2:**
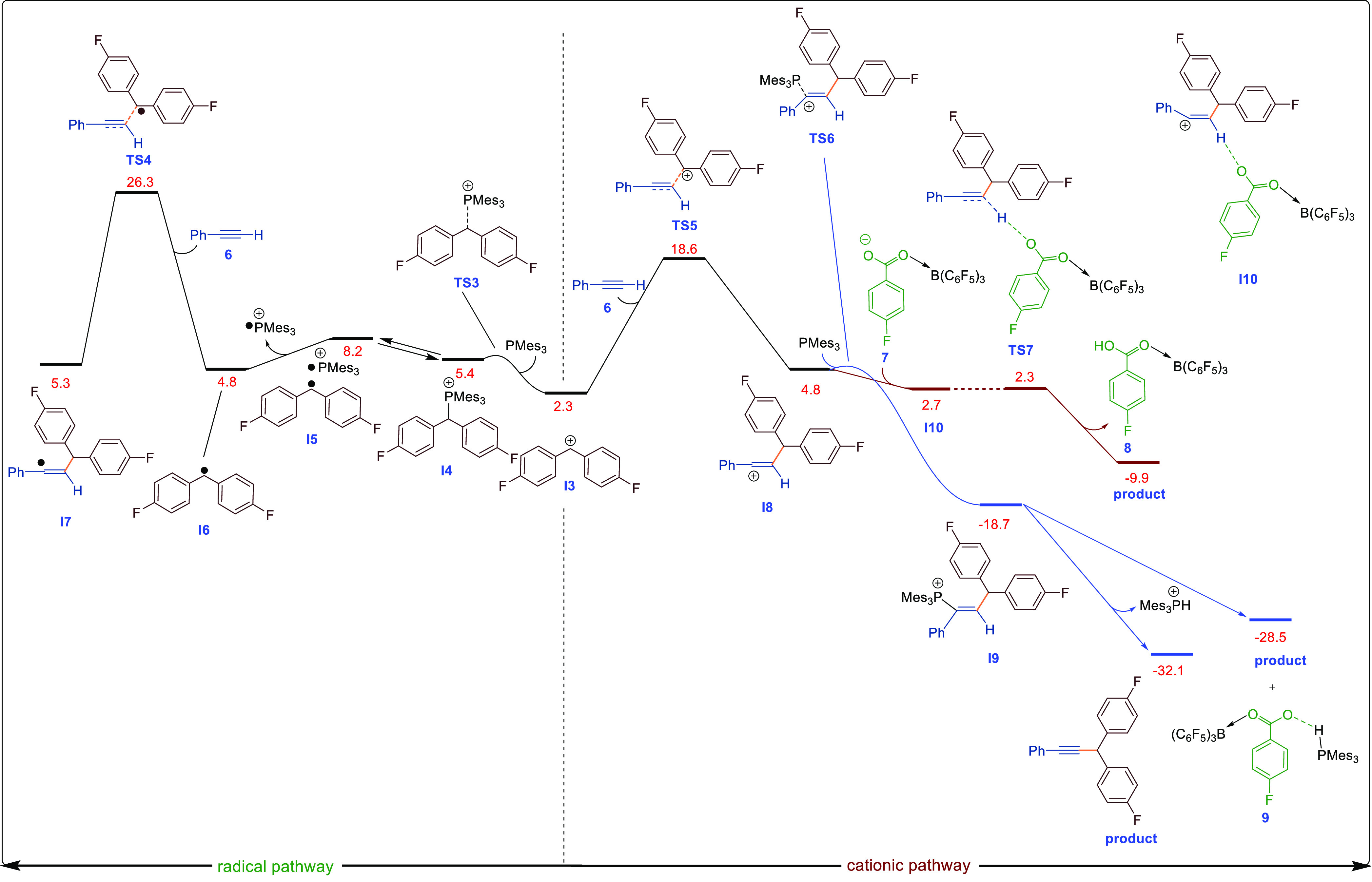
DFT-computed reaction pathways for the reaction of **I3** with phenylacetylene calculated by SMD/B3LYP-D3/def2-TZVP//SMD/B3LYP-D3/6-31G(d) in THF. The relative free energies are given in kcal/mol. For this energy profile, structure **1a** is set as the reference point as indicated in Figure S178.

To examine the contrasting reaction pathways and to explain the experimental and EPR observations, we undertook a thorough DFT investigation of all potential reaction pathways. Calculations were performed at the SMD/B3LYP-D3/def2-TZVP//SMD/B3LYP-D3/6-31G(d) level of theory in THF and toluene solvent to examine the origin of the products. As previously reported by Slootweg et al., we found that the formation of the frustrated radical ion pair from the FLP is energetically unfavorable by 35.7 kcal/mol. This corroborates the observation that we and others^6^ fail to see any EPR signal in solutions of B(C_6_F_5_)_3_ and Mes_3_P. Very recently, Slootweg et al.^5a^ showed that the coordination of B(C_6_F_5_)_3_ to the diaryl ester increases the electron affinity of the substrate, and the energy required for SET from Mes_3_P to the methylene carbon atom is 40.0 kcal/mol.^5a^ This is still quite large, and our calculations have revealed that, regardless of whether a diamagnetic or paramagnetic reaction pathway is ultimately operative, the first step in the reaction is the same: B(C_6_F_5_)_3_ activation of the ester to generate diaryl methylene cation **I3** (Scheme 8) and the borate anion [R^1^CO_2_B(C_6_F_5_)_3_]^−^ with an activation energy of 10.7 kcal/mol. (See SIFigure S178 for the free-energy profile.) The energies of **I3** can be noticeably varied by changing the substitution at the para position of the aryl esters. Electron-withdrawing (*p*-CF_3_, **1j**), electron-withdrawing/π-releasing (*p*-F, **1a**), and electron-releasing (*p*-OMe, **1d**) showed different energies for **I3** (SIFigure S179–180). As expected, **I3**_**1d**_ (−9.4 kcal/mol) is energetically more favorable than **I3**_**1j**_ (13.5 kcal/mol) due to the electron-releasing substituents (*p*-OMe). Strongly electron-withdrawing substituents (*p*-CF_3_) conversely make **I3** formation thermodynamically less favorable. **I3** is then the branching point for the single- and two-electron pathways (Figure 2). The combination of diaryl methylene cation **I3** with Mes_3_P can lead to three possible species in solution (Scheme 8): (i) the frustrated Lewis pair (uncoordinated **I3** + Mes_3_P), (ii) the Lewis acid–base adduct (**I4**), and (iii) the frustrated radical pair (FRP, **I5**). The energy difference and reaction barriers between these species are very low; therefore, it is likely that all three scenarios exist in equilibrium under the reaction conditions. This supports the EPR data which shows the formation of [Mes_3_P]^•+^ and the [(P(Mes_*n*=2,3_)_2_]^•+^ dimer in the reaction of the ester with the Mes_3_P/B(C_6_F_5_)_3_ FLP.

Although the carbon-based radical could not be observed in this case, we have observed a weak carbon-based radical EPR signal when reacting other esters with the Mes_3_P/B(C_6_F_5_)_3_ FLP in the absence of irradiation or heat.^6^ We then investigated the addition of the cation (**I3**) or radical (**I6**) to the alkyne. Although both pathways are feasible under the reaction conditions, the cationic pathway was lower in activation energy than the radical pathway by about 26.3–18.6 = 7.7 kcal/mol. In the case of the diamagnetic pathway, the addition of the **I3** cation to the alkyne generates **I8** via TS5. The resulting cation in **I8** is highly reactive and is rapidly trapped by the Lewis base Mes_3_P generating **I9**. Finally, anti-elimination of [Mes_3_PH]^+^ generates the cross-coupled compound and phosphonium borate salt as the final products.

The Lewis base employed is very important for the reaction to occur as seen in the screening studies. First, the ability to form an FLP (or weak adduct) with both the Lewis acid borane and the (di)aryl methylene cation (**I3**) is critical, as other strong, less hindered Lewis acids such as BF_3_ do not work in the reaction. Likewise, smaller phosphines or amines tend to coordinate more strongly to the carbocation (**I3**). In addition to being able to form an FLP, the base also functions to trap reactive **I8**. Indeed, one could possibly conceive that the reaction could proceed with the Lewis acid only, whereby R^1^CO_2_^–^ accepts the proton in the last step (**I8** → **I10** → product, Figure 2). However, DFT calculations showed that this pathway was less favorable thermodynamically, and experimentally this reaction showed only a 22% yield with many side products formed.

We were curious to know whether both the paramagnetic and diamagnetic pathways are operative in parallel or if a diamagnetic mechanism is purely responsible for the product formation for all alkynes and the radicals observed from EPR studies were simply off-pathway intermediates. To establish this, we undertook further DFT calculations to investigate the effect of electron-withdrawing, electron-donating, and electron-neutral substituents on the ester (**1**) and the alkyne on the activation barrier for the reaction.

**Figure 3:**
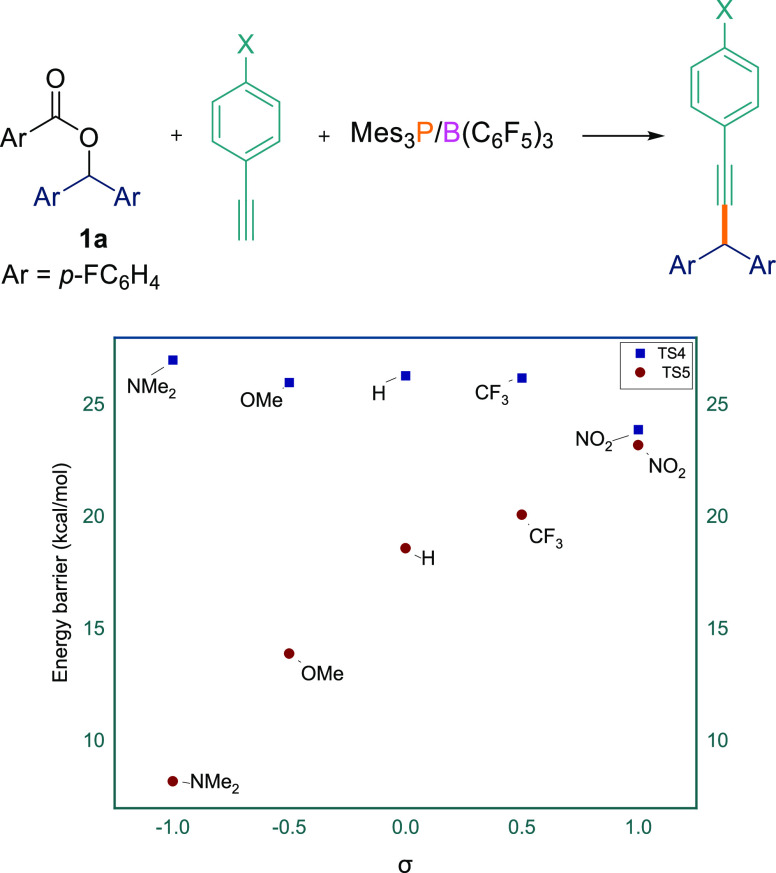
DFT (SMD/B3LYP-D3/def2-TZVP//SMD/B3LYP-D3/6-31G(d) in THF)-computed energy barrier (**TS4** and **TS5**, Table 3; entry 1–5) plotted as a function of the Hammett-substituent constant in a Hammett-style plot. The relative free energies are given in kcal/mol.

The energy barriers **TS4**_radical pathway_ and **TS5**_cationic pathway_ were calculated (Table 4, see SIFigure S181). Initially we varied the electronic properties of the alkyne using *p*-XC_6_H_4_C≡CH (X = NO_2_, CF_3_, H, OMe, NMe_2_) with ester **1a**. As evidence from the DFT calculations, changing the substitution at the *para* position of the arylacetylene did not make significant difference for **TS4**_radical pathway_ in their respective energy barrier (23.9–27.0 kcal/mol) (Table 4, entries 1–5). However, the energy barrier for **TS5**_cationic pathway_ changed dramatically (8.2–23.2 kcal/mol). When electron-withdrawing groups (*p*-NO_2_ and *p*-CF_3_) on the arylacetylene were employed, the differences between **TS4**_radical pathway_ and **TS5**_cationic pathway_ are 0.7 and 6.1 kcal/mol (Table 4, entries 1 and 2). Electronically neutral phenylacetylene exhibits a **TS4**_radical pathway_ → **TS5**_cationic pathway_ difference of 7.7 kcal/mol (Table 3, entry 3). Electron-donating groups such as methoxy (**TS4**_radical pathway_ – **TS5**_cationic pathway_ = 12.1 kcal/mol) and *N*,*N*-dimethylamine (**TS4**_radical pathway_ – **TS5**_cationic pathway_ = 18.8 kcal/mol), on the other hand, showed a significant energy difference (Table 4, entries 4 and 5). These observations can be seen in Figure 3, which shows that for **TS4** there is a negligible change in the energy barrier when changing the electronic properties of the acetylenic substrate. This is in agreement with little charge formation on the reaction center in the radical mechanism. Conversely, for **TS5**, there is a strong positive correlation between the **TS5** energy barrier and the substituent *σ*_*p*_ constant. This is expected for the cationic pathway because a developing positive charge adjacent to the substituted phenyl ring will be stabilized by electron-donating groups (e.g., *p*-NMe_2_ or *p*-OMe) on the acetylene. As can be seen in Figure 3, **TS5** is lower than **TS4** for all substituents explored, although the difference becomes small for strongly electron-withdrawing groups.

**Table 4:** DFT-Computed **TS4**_radical pathway_ and **TS5**_cationic pathway_ for the Reaction of **1a**, **d**, or **j** with Various Arylacetylenes (*p*-XC_5_H_4_C≡CH) Calculated by SMD/B3LYP-D3/def2-TZVP//SMD/B3LYP-D3/6-31G(d) in THF^a^

entry	ester (Ar)	X	TS4_radical pathway_	TS5_cationic pathway_
1	**1a** (*p*-FC_6_H_4_)	NO_2_	23.9	23.2
2	**1a** (*p*-FC_6_H_4_)	CF_3_	26.2	20.1
3	**1a** (*p*-FC_6_H_4_)	H	26.3	18.6
4	**1a** (*p*-FC_6_H_4_)	OMe	26.0	13.9
5	**1a** (*p*-FC_6_H_4_)	NMe_2_	27.0	8.2
6	**1d** (*p*-OMeC_6_H_4_)	CF_3_	23.7	13.0
7	**1d** (*p*-OMeC_6_H_4_)	OMe	27.8	8.1
8	**1j** (*p*-CF_3_C_6_H_4_)	CF_3_	24.1	25.5
9	**1j** (*p*-CF_3_C_6_H_4_)	OMe	22.2	17.5

a The relative free energies are given in kcal/mol.

We subsequently computed the energy barrier for the two transition states by varying the electronic properties of the aryl ester using electron-donating (*p*-OMe, **1d**) and electron-withdrawing (*p*-CF_3_, **1j**) esters with electron-deficient (1-ethynyl-4-(trifluoromethyl)benzene) and electron-rich (1-ethynyl-4-methoxybenzene) acetylenes (Table 4, entries 6–9). For both esters, a smaller change in the **TS4**_radical pathway_ energy barrier was observed (range 22.2–27.8 kcal/mol) compared to the **TS5**_cationic pathway_ energy barrier (range 8.1–25.5 kcal/mol) when changing the substituent on the phenylacetylene (Table 4, entries 6–9). As with ester **1a**, both esters disclosed a larger energy difference between the two pathways when reacted with acetylenic compounds bearing an electron-donating group (*p*-OMe). Likewise, a much smaller energy difference was noted for both esters when reacted with acetylenic compounds bearing an electron-withdrawing group (*p*-CF_3_). Interestingly, DFT studies showed that for the case of the reaction of ester **1j** with 1-ethynyl-4-(trifluoromethyl), the radical pathway is slightly energetically more favorable compared with the cationic pathway (Table 4, entry 8). These results suggest that for electron-withdrawing arylacetylenes both paramagnetic and diamagnetic mechanisms are potentially possible, whereas for electron-rich arylacetylenes a purely diamagnetic pathway is operative.

The key difference in the two mechanisms involves the reaction of either a cationic intermediate or a radical intermediate with the arylacetylene, generating a new cationic or radical species. Whether this new intermediate is a cationic or a neutral radical species can be probed using a Hammett plot (cf. computational studies) based on substituted arylacetylenes. To gain this insight into the reaction pathway and substituent effects also from experimental evidence, we examined competition reactions among the FLP, aryl ester **1a**, and arylacetylenes *p*-XC_6_H_4_C≡CH bearing electron-withdrawing, electron-neutral, and electron-releasing groups. The Hammett plot requires relative rate constants for the reaction of different substituted alkynes that were obtained using a series of competition experiments. Initial competition experiments in the presence of 1.5 equiv of five arylacetylenes *p*-XC_6_H_4_C≡CH (X = CF_3_, F, Cl, H, and OMe) were unsuccessful. The excess arylacetylene present in the reaction mixture destroyed the efficacy of the FLP system, producing a complicated reaction mixture which was not suitable for *in situ* NMR analysis. We therefore carried out three binary competition experiments with two alkynes being present at 1.5 equiv each (Table 5).

**Table 5:**
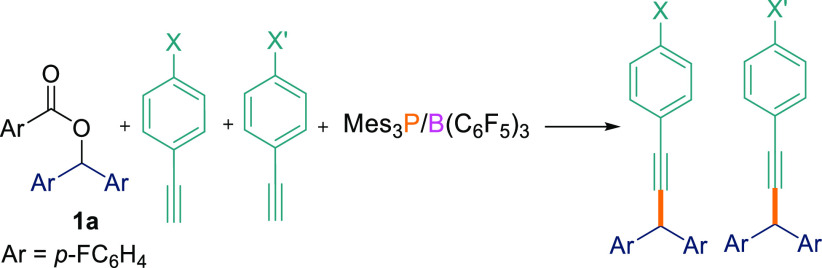
Competitive Reaction among **1a**, Different Acetylenes, and the FLP

entry	X	X′	product ratio	*k*_*x*_/*k*_*x*′_
1	OMe	H	(**2d**:**2c**) 14.3:1	21.9
2	CF_3_	H	(**2b**:**2c**) 0.12:1	0.082
3	OMe	CF_3_	(**2d**:**2b**) 1: < 0.05	

**Figure 4:**
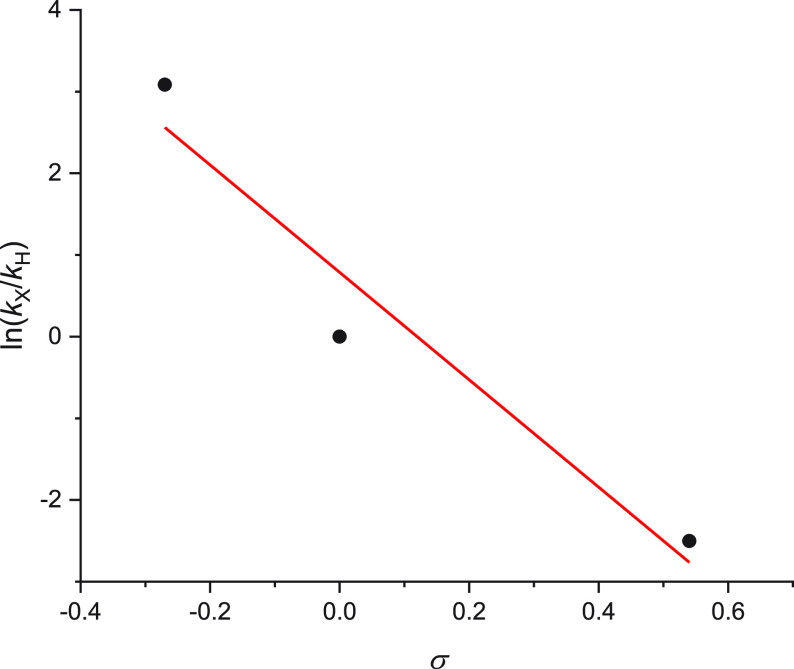
Hammett plot for the reaction of **I3** with arylacetylenes.

Using the optimized reaction conditions, three parallel reactions were carried out in which equimolar mixtures of (a) 1-ethynyl-4-methoxybenzene and phenylacetylene, (b) 1-ethynyl-4-(trifluoromethyl)benzene and phenylacetylene, and (c) 1-ethynyl-4-methoxybenzene and 1-ethynyl-4-(trifluoromethyl)benzene were reacted with 1 equiv of ester **1a**. The ratios of C_sp^3^_–C_sp_ cross-coupled products (**2d**/**2c**, **2b**/**2c**, and **2d**/**2b**) were determined from the crude reaction mixture using ^19^F NMR spectroscopy (Table 5, see SI, Figure S175). For entries 1 and 2 in Table 5, the relative integrals for the products were used to calculate the remaining equivalents for the alkynes after reaction. Using the approach developed by Ingold and Shaw^30^ and proposed for one-pot Hammett plots by Harper and co-workers,^31^ we obtained relative rate constants *k*_*x*_/*k*_*x′*_ for the reaction of differently substituted arylacetylenes with reaction intermediate **I3** or its equilibrium species. Entry 3 confirms that in the competition between **1a** and 1-ethynyl-4-(trifluoromethyl)benzene/1-ethynyl-4-methoxybenzene, product **2b** is undetectable, in agreement with the >200-fold difference in rate constants deduced from entries 1 and 2. The relative rate constants in Table 5 are normalized with respect to the unsubstituted alkyne and can therefore be used directly to construct a Hammett plot (using Hammett substituent constants from ref (32)). The Hammett plot (Figure 4) shows a clearly negative slope of −6.6 ± 1.7, suggesting the formation of a positive charge on the new intermediate, which is indicative of the cationic reaction mechanism being operative. The Hammett plot is also in agreement with the Hammett-style plot constructed using the computational data (Figure 3) for the cationic mechanism. The slope of the Hammett-style plot for **TS5** (Figure 3) is 8.9 ± 1.2 kcal mol^–1^. At 70 °C, this corresponds to a Hammett ρ value of 5.7 ± 0.8. The computational data is thus in excellent agreement with the experimental findings.

**Figure 5:**
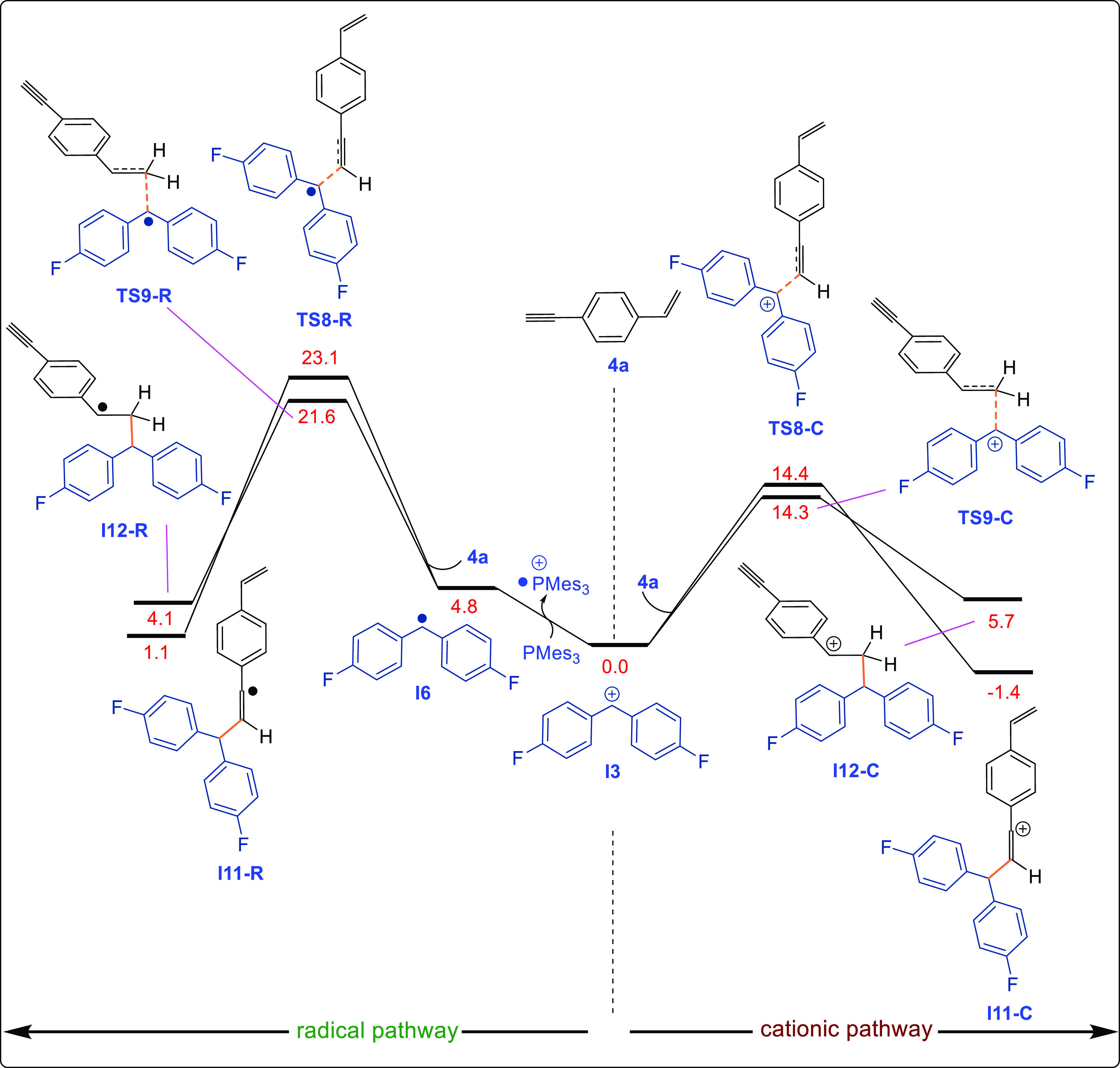
DFT-computed reaction pathways for the reaction of **I3** with 1-ethynyl-4-vinylbenzene (**4a**) and Mes_3_P calculated using the SMD/B3LYP-D3/def2-TZVP//SMD/B3LYP-D3/6-31G(d) level of theory in THF.

Finally, we turned our attention to explaining the regioselectivity with compound **4a**. The DFT-computed (SMD/B3LYP-D3/def2-TZVP//SMD/B3LYP-D3/6-31G(d)) reaction pathways for the reaction of **I3** with 1-ethynyl-4-vinylbenzene (**4a**) and Mes_3_P in THF reveal that the cationic pathway is energetically more favorable (Figure 5). After the generation of **I3**, reaction at either the alkyne or alkene site affords the corresponding C_sp^3^_–C_sp_ or C_sp^3^_–C_sp^2^_ cross-coupled product. Although the transition-state energies for the **I3**-alkyne adduct (14.4 kcal/mol) and **I3**-alkene adduct (14.3 kcal/mol) are very similar, the C_sp^3^_–C_sp_ cross-coupled products are thermodynamically more stable (−1.4 kcal/mol) than the C_sp^2^_–C_sp^2^_ cross-coupled product (5.7 kcal/mol), explaining why only the C_sp^3^_–C_sp_ cross-coupled product is observed for 1-ethynyl-4-vinylbenzene (**4a**) (Figure 5). To confirm this, we also executed single-point benchmark calculations for this transition state with a different method and solvent system (SI, Table S2), which showed results similar to those indicated in Figure 5.

**Figure 6:**
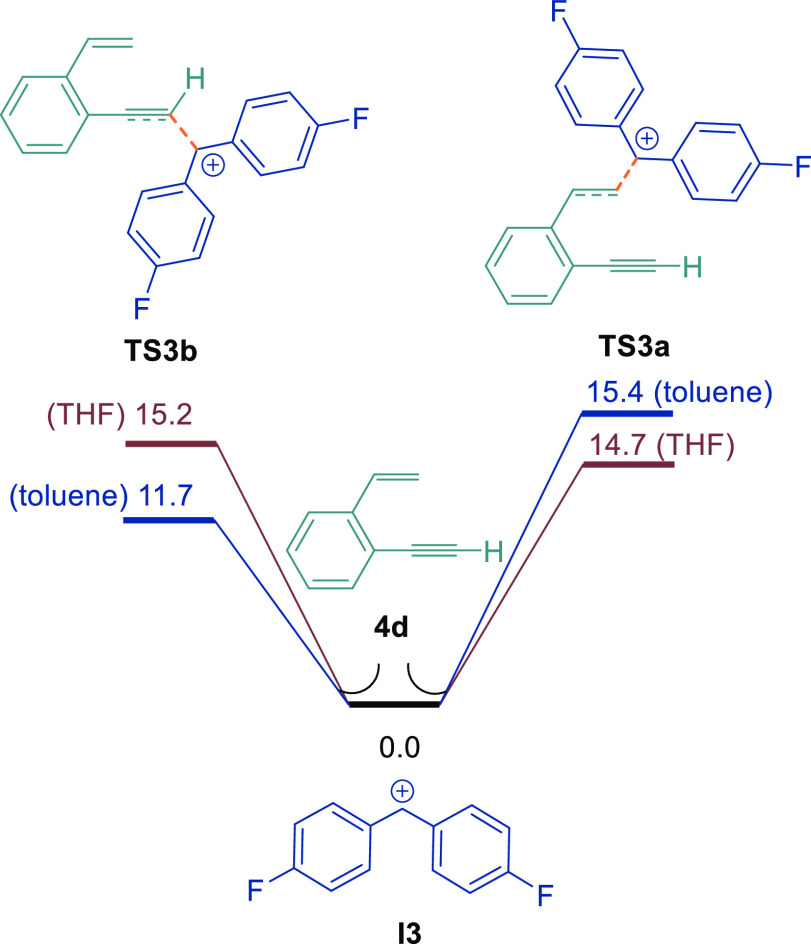
DFT calculations of the site selectivity of **I3** with 1-ethynyl-2-vinylbenzene **4d**, calculated at the SMD/B3LYP-D3/def2-TZVP//SMD/B3LYP-D3/6-31G(d) level in THF and toluene.

The alternating site selectivity when using 1-ethynyl-2-vinylbenzene (**4d**) in differing solvents can also be highlighted in DFT studies. For the calculations, the mechanism was studied by utilizing two different solvents (toluene and THF). Experimentally, both toluene and TFT solvents lead to preferential C_sp^3^_–C_sp_ coupling. DFT calculations for the reaction of **I3** with **4d** showed that the transition-state energy for the addition of the diaryl methylene cation to the alkene or alkyne varies depending upon the solvent (Figure 6). In toluene solvent, the energy barrier for the addition of **I3** to the more nucleophilic alkyne was 3.7 kcal/mol lower in energy than the transition state for addition to the alkene (11.7 versus 15.4 kcal/mol). The converse was true for reactions in THF, whereby the addition of **I3** to the alkene was 0.5 kcal/mol lower in energy than the energy barrier for addition to the alkyne (14.7 versus 15.2 kcal/mol). We attribute this to the higher dipole moment of the calculated transition-state structure of **TS3a**, which can be stabilized better by the THF molecule. These results also explain why, in THF, the reactions were less selective, leading to a mixture of C_sp^3^_–C_sp_ and C_sp^3^_–C_sp^2^_ products due to the small energy difference between the two pathways. The reactions in a solvent such as toluene (or TFT) are more selective toward C_sp^3^_–C_sp_ coupling due to the larger energy difference between the two pathways.

## Conclusions

We have demonstrated new reactivities of FLPs in the functionalization of terminal alkynes through C_sp^3^_–C_sp_ coupling reactions with aryl esters. DFT studies found that a diamagnetic pathway was most likely, although a low-energy single-electron pathway could operate to some extent. In particular, DFT studies indicate that the combination of the Mes_3_P/diaryl methylene cation led to three species of similar energy in solution: the FLP (**I3**), the Lewis acid–base adduct (**I4**), and the frustrated radical pair (**I5**). According to the Curtin–Hammett principle, the reaction proceeds predominantly via **TS5** from rapidly equilibrating **I3**, **I4**, **I5**, and **I6**. These rapidly equilibrating species in solution are supported by the observation of radical species of varying stabilities and lifetimes in the reaction mixture. Thus, radical species are formed in the reaction but are not making a substantial contribution on the reaction pathway to the product, with the possible exception of arylacetylenes with strongly electron-withdrawing (e.g., *p*-NO_2_, *p*-CF_3_) substituents. These observations will be of importance when designing future reactions that can switch between one- and two-electron pathways depending upon the substrate. Moreover, we observed high site selectivity when ethynyl vinylbenzene substrates were employed in these reactions. 1-Ethynyl-4-vinylbenzene substrates reacted only at the alkyne site, but 1-ethynyl-2-vinylbenzene substrates showed high selectivities depending upon the polarity of the solvent. For 1-ethynyl-2-vinylbenzene in THF, C_sp^3^_–C_sp_ coupling was observed, resulting in alkene functionalization, whereas in toluene or TFT exclusive C_sp^3^_–C_sp_ coupling and alkyne functionalization resulted. The contrasting selectivity was explained by DFT and computed transition states in the differing solvents. FLP-mediated C–C bond-forming reactions are still relatively new, but there is no doubt that advances will continue to be made in this area. This reported methodology can be utilized to generate compounds that can be subsequently employed for the synthesis of useful novel natural products where metal-free synthesis is highly desirable for avoiding metal toxicities.^15,33^

## Supporting Information Available

Detailed experimental procedures, NMR spectra, EPR data, kinetic data, and DFT data (PDF)
