# Supplementary material for: Site-Selective Csp3–Csp/Csp3–Csp2 Cross-Coupling Reactions Using Frustrated Lewis Pairs
Source: J Am Chem Soc. 2021 Mar 15;143(11):4451–64. doi: 10.1021/jacs.1c01622 (PMC8041292; doi:10.1021/jacs.1c01622)
Supplement: Supplementary file 1 — ja1c01622_si_001.pdf [file ja1c01622_si_001.pdf]

Supporting Information

Site Selective C_{sp3}–C_{sp}/C_{sp3}–C_{sp2} Cross Coupling Reactions Using Frustrated Lewis Pairs

Ayan Dasgupta[†], Katarina Stefkova[†], Rasool Babaahmadi[‡], Brian F. Yates[‡], Niklaas Buurma[†], Alireza Ariafard[‡], Emma Richards[†], and Rebecca L. Melen[†]*

[†] Cardiff Catalysis Institute, School of Chemistry, Cardiff University, Main Building, Cardiff, CF10 3AT, UK.

[‡] School of Natural Sciences-Chemistry, University of Tasmania Private Bag 75, Hobart, Tasmania 7001, Australia.

Email: MelenR@cardiff.ac.uk

Table of Content

1	Experimental		03
	1.1	General experimental	03
	1.2	General procedure a: synthesis of aryl esters	04
	1.2.1	Synthesis and spectral characterization of aryl esters	04
2	Product Characterization		12
	2.1	General procedure b: C–C cross coupling reaction	12
	2.2	Synthesis and spectral characterization of C–C cross coupled compounds	13
3	NMR Spectra		41
4	Competition experiments		215
5	EPR Studies		215
6	Computational Data		219
	5.1	Computational details	219
	5.2	Additional free energy profiles	219
	5.3	Cartesian coordinates and total energies for the calculated structures	222
7	References		324

1. Experimental:

1.1 General experimental:

Except for the starting materials, all reactions and manipulations were carried out under an atmosphere of dry, O_2-free nitrogen using standard double-manifold techniques with a rotary oil pump. A nitrogen-filled glove box (MBraun) was used to manipulate solids including the storage of starting materials, ambient temperature reactions, product recovery and sample preparation for analysis. All solvents (toluene, tetrahydrofuran, dichloromethane, hexane, diethyl ether) were dried by employing a Grubbs-type column system (Innovative Technology) or a solvent purification system MB SPS-800 and stored under a nitrogen atmosphere. Anhydrous (with Sure/Seal) α,α,α-trifluorotoluene was purchased from Merck and dried over molecular sieves before use. Deuterated solvents were distilled and/or dried over molecular sieves before use. Chemicals were purchased from commercial suppliers and used as received. Tris(pentafluorophenyl)borane and trimesitylphosphine were prepared as per the standard literature report.[1] Thin-layer chromatography (TLC) was performed on pre-coated aluminum sheets of Merck silica gel 60 F254 (0.20 mm). ^{1}H, ^{13}C, and ^{19}F NMR spectra were recorded on a Bruker Avance II 400 or Bruker Avance 500 spectrometers. All coupling constants are habsolute values and are expressed in Hertz (Hz). ^{13}C NMR was measured as ^{1}H decoupled. Yields are given as isolated yields. Chemical shifts are expressed as parts per million (ppm, δ) downfield of tetramethylsilane (TMS) and are referenced to $CDCl_3$ (7.26/77.16 ppm) as internal standard. NMR spectra were referenced to $CFCl_3$ (^{19}F).[2] The description of signals includes s = singlet, d = doublet, t = triplet, q = quartet, and m = multiplet, br. = broad. All coupling constants are absolute values and are expressed in Hertz (Hz). ^{13}C NMR spectroscopy was measured as ^{1}H decoupled. Yields are given as isolated yields. All spectra were analyzed assuming a first order approximation. IR-Spectra were measured on a Shimadzu IRAffinity-1 photo-spectrometer. Mass spectra were measured on a Waters LCT Premier/XE or a Waters GCT Premier spectrometer. Ions were generated by the Atmospheric Solids, Analysis Probe (ASAP), Electrospray (ES) or Electron Ionization (EI). The molecular ion peaks values quoted for either molecular ion (M^+), molecular ion plus or minus hydrogen ($M+H^+$, $M-H^-$), molecular ion minus chlorine ($M-Cl^-$), or molecular ion plus sodium ($M+Na^+$).

1.2 Synthesis of aryl esters:

General Procedure a: acyl chloride (1.2 equiv) and alcohol (1 equiv) were dissolved in pyridine at 0 °C. The mixture was stirred at ambient temperature overnight. The reaction was quenched with water and extracted with ethyl acetate (3 × 25 mL). The combined organic fractions were washed with brine solution (1 × 25 mL) and dried over $MgSO_4$. All volatiles were removed *in vacuo* and the crude compound was purified *via* column chromatography using silica gel (Merck, 60 Å, 230−400 mesh particle size) and hexane/ethyl acetate as eluent.

1.2.1 Synthesis and spectral characterization of aryl ester compounds:

Synthesis of bis(4-fluorophenyl) methyl 4-fluorobenzoate (**1a**)[3]

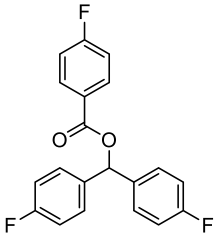

Synthesized in accordance with *General Procedure a* using 4-fluorobenzoyl chloride (2.7 mL, 23.2 mmol.), bis(4-fluorophenyl) methanol (4.4 g, 20.0 mmol), and pyridine (25 mL). All volatiles were removed *in vacuo* and the crude compound was purified *via* column chromatography using silica gel and hexane/ethyl acetate (20:1 v/v) as eluent: The desired product **1a** was obtained as a white solid. Yield: 5.81 g, 17.0 mmol, 85%.

^{1}H NMR (400 MHz, $CDCl_3$, 298 K) δ: 8.15–8.12 (m, 2H, Ar–CH), 7.39–7.36 (m, 4H, Ar–CH), 7.16–7.12 (m, 2H, Ar–CH), 7.08–7.04 (m, 5H, Ar–CH and CH); ^{13}C NMR (101 MHz, $CDCl_3$, 298 K) δ: 166.1 (d, $J_{C–F}$ = 254.7 Hz), 164.8 (C=O), 162.6 (d, $J_{C–F}$ = 247.3 Hz), 135.8 (d, $J_{C–F}$ = 3.3 Hz), 132.4 (d, $J_{C–F}$ = 9.4 Hz), 129.0 (d, $J_{C–F}$ = 8.3 Hz), 126.2 (d, $J_{C–F}$ = 3.0 Hz), 115.8 (d, $J_{C–F}$ = 22.2 Hz), 115.7 (d, $J_{C–F}$ = 21.6 Hz), 76.4 (CH); ^{19}F NMR (376 MHz, $CDCl_3$, 298 K) δ: -104.80 (s, 1F, Ar–F), -113.67 (s, 2F, Ar–F); IR ν_{max} (cm^{-1}): 3116, 3074, 1724 (C=O), 1602, 1504, 1413, 1340, 1301, 1265, 1186, 1099; HRMS (EI+) $[M]^+$ calculated for $[C_{20}H_{13}O_2F_3]^+$: 342.0868, found: 342.0871.

Synthesis of bis(4-chlorophenyl)methyl 4-fluorobenzoate (**1b**)

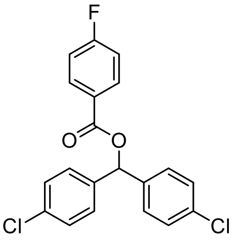

Synthesized in accordance with *General Procedure a* using 4-fluorobenzoyl chloride (1.5 mL, 14.2 mmol), bis(4-chlorophenyl) methanol (3 g, 11.8 mmol), and pyridine (20 mL). All volatiles were removed *in vacuo* and the crude compound was purified *via* column chromatography using silica gel and hexane/ethyl acetate (20:1 v/v) as eluent: The desired product **1b** was obtained as a white solid. Yield: 3.5 g, 9.3 mmol, 78%.

^{1}H NMR (500 MHz, $CDCl_3$, 298 K) δ: 8.12 (dd, *J* = 8.9, 5.4 Hz, 2H, Ar–CH), 7.35–7.31 (m, 8H, Ar–CH), 7.16–7.12 (m, 2H, Ar–CH), 7.02 (s, 1H, CH); ^{13}C NMR (126 MHz, $CDCl_3$, 298 K) δ: 166.1 (d, $J_{C–F}$ = 254.9 Hz), 164.5 (C=O), 138.2, 134.3, 132.4 (d, $J_{C–F}$ = 9.4 Hz), 129.0, 128.6, 126.0 (d, $J_{C–F}$ = 3.0 Hz), 115.9 (d, *J* = 22.0 Hz), 76.3 (CH); ^{19}F NMR (471 MHz, $CDCl_3$, 298 K) δ: -104.64; IR ν_{max} (cm^{-1}): 3023, 2994, 1723 (C=O), 1602, 1507, 1492, 1412, 1340, 1302, 1260, 1238, 1153, 1108, 1087, 1014; HRMS (EI+) [M]$^+$calculated for $[C_{20}H_{13}O_2Cl_2F]^+$: 374.0271, found: 374.0271.

Synthesis of benzhydryl 4-fluorobenzoate (**1c**)[3]

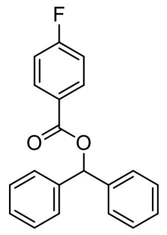

Synthesized in accordance with *General Procedure a* using 4-fluorobenzoyl chloride (2.7 mL, 23.2 mmol), diphenylmethanol (3.68 g, 20.0 mmol), and pyridine (25 mL). All volatiles were removed *in vacuo* and the crude product was purified *via* column chromatography using silica gel and hexane/ethyl acetate (20:1 v/v) as eluent. The desired product **1c** was obtained as a white solid. Yield: 5.49 g, 17.8 mmol, 89%.

^{1}H NMR (400 MHz, $CDCl_3$, 298 K) δ: 8.18–8.15 (m, 2H, Ar–CH), 7.44–7.42 (m, 4H, Ar–CH), 7.38–7.34 (m, 4H, Ar–CH), 7.32–7.29 (m, 2H, Ar–CH), 7.15–7.11 (m, 3H, Ar–CH and CH); ^{13}C NMR (101 MHz, $CDCl_3$, 298 K) δ: 166.0 (d, $J_{C–F}$ = 254.3 Hz), 164.7 (C=O), 143.9, 140.2, 132.5 (d, $J_{C–F}$ = 9.3 Hz), 128.7, 128.6, 127.7, 127.2, 126.6, 126.6, 126.5, 115.76 (d, $J_{C–F}$ = 22.0 Hz), 77.7 (CH); ^{19}F NMR (376 MHz, $CDCl_3$, 298 K) δ: -105.19 (Ar–F); IR ν_{max} (cm^{-1}): 3026, 3030, 1716 (C=O), 1598, 1504, 1454, 1411, 1361, 1294, 1184, 1105, 1089, 1014; HRMS (EI+) [M]$^+$ calculated for $[C_{20}H_{15}O_2F]^+$: 306.1056, found: 306.1056.

Synthesis of bis(4-methoxyphenyl) methyl 4-fluorobenzoate (**1d**)[3]

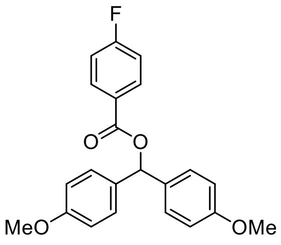

Synthesized in accordance with *General Procedure a* using 4-fluorobenzoyl chloride (2.7 mL, 23.2 mmol), bis(4-methoxyphenyl) methanol (4.86 g, 20.0 mmol), and pyridine (25 mL). All volatiles were removed *in vacuo* and the crude product was purified *via* column chromatography using silica gel and hexane/ethyl acetate (90:10 v/v) as eluent. The desired product **1d** was obtained as a colorless oil. Yield: 5.46 g, 14.9 mmol, 76%.

^{1}H NMR (400 MHz, $CDCl_3$, 298 K) δ: 8.13 (ddd, *J* = 8.9, 5.4, 1.4 Hz, 2H, Ar–CH), 7.39–7.29 (m, 4H, Ar–CH), 7.12 (td, *J* = 8.5, 1.4 Hz, 2H, Ar–CH), 7.04 (s, 1H, CH), 6.95–6.86 (m, 4H,

Ar–CH), 3.80 (s, 6H, OCH_3); ^{13}C NMR (101 MHz, $CDCl_3$, 298 K) δ: 165.9 (d, $J_{C–F}$ = 254.0 Hz), 164.8 (C=O), 159.4, 132.6, 132.4 (d, $J_{C–F}$ = 9.3 Hz), 128.6, 126.7 (d, $J_{C–F}$ = 3.0 Hz), 115.6 (d, $J_{C–F}$ = 22.0 Hz), 114.0, 76.9 (CH), 55.4 (OCH_3); ^{19}F NMR (376 MHz, $CDCl_3$, 298 K) δ: -105.46 (Ar–F); IR ν_{max} (cm^{-1}): 3116, 3074, 1724 (C=O), 1602, 1413, 1340, 1301, 1263, 1186, 1099, 1014; HRMS (ES+) $[M+Na]^+$ calculated for $[C_{22}H_{19}O_4FNa]^+$: 389.1165, found: 389.1166.

Synthesis of (4-chlorophenyl) (phenyl)methyl 4-fluorobenzoate (**1e**)[3]

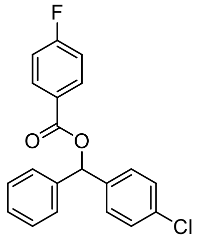

Synthesized in accordance with *General Procedure a* using 4-fluorobenzoyl chloride (2.7 mL, 23.2 mmol), 4-chlorobenzohydrol (4.37 g, 20.0 mmol), and pyridine (25 mL). All volatiles were removed *in vacuo* and the crude product was purified *via* column chromatography using silica gel and hexane/ethyl acetate (90:10 v/v) as eluent. The desired product **1e** was obtained as a white solid. Yield 5.59 g, 16.4 mmol, 82%.

^{1}H NMR (500 MHz, $CDCl_3$, 298 K) δ: 8.17–8.11 (m, 2H, Ar–CH), 7.42–7.30 (m, 9H, Ar–CH), 7.16–7.11 (m, 2H, Ar–CH), 7.07 (s, 1H, CH); ^{13}C NMR (126 MHz, $CDCl_3$, 298 K) δ: 166.1 (d, $J_{C–F}$ = 254.6 Hz), 164.6 (C=O), 139.7, 138.8, 134.1, 132.4 (d, $J_{C–F}$ = 9.4 Hz), 128.9, 128.8, 128.6, 128.4, 127.2, 126.3 (d, $J_{C–F}$ = 3.0 Hz), 115.8 (d, $J_{C–F}$ = 22.1 Hz), 77.0 (CH); ^{19}F NMR (471 MHz, $CDCl_3$, 298 K) δ: -104.96 (Ar–F); IR ν_{max} (cm^{-1}): 3053, 2970, 1722 (C=O), 1602, 1506, 1490, 1415, 1367, 1263, 1232, 1151, 1105, 1087, 1012; HRMS (EI) [M] calculated for $[C_{20}H_{14}ClFO_2]$: 340.0666, found 340.0661.

Synthesis of phenyl(p-tolyl)methyl 4-fluorobenzoate (**1f**)[3]

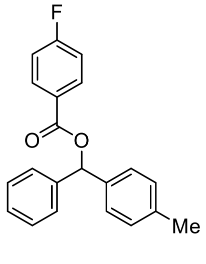

Synthesized in accordance with *General Procedure a* using 4-fluorobenzoyl chloride (2.7 mL, 23.2 mmol), (4-methylphenyl) (phenyl)methanol (3.97 g, 20 mmol), and pyridine (25 mL). All volatiles were removed *in vacuo* and the crude product was purified *via* column chromatography using silica gel and hexane/ethyl acetate (90:10 v/v) as eluent. The desired product **1f** was obtained as a white solid. Yield: 4.99 g. 15.6 mmol, 78%.

^{1}H NMR (500 MHz, $CDCl_3$, 298 K) δ: 8.18–8.13 (m, 2H, Ar–CH), 7.42 (d, J = 7.1 Hz, 2H, Ar–CH), 7.36 (t, J = 7.5 Hz, 2H, Ar–CH), 7.31 (t, J = 6.8 Hz, 3H, Ar–CH), 7.17 (d, J = 7.7 Hz, 2H, Ar–CH), 7.13 (t, J = 8.7 Hz, 2H, Ar–CH), 7.08 (s, 1H, CH), 2.34 (s, 3H, CH_3); ^{13}C NMR (126 MHz, $CDCl_3$, 298 K) δ: 166.0 (d, $J_{C–F}$ = 254.1 Hz), 164.8 (C=O), 140.4, 138.0, 137.3, 132.5 (d, $J_{C–F}$ = 9.3 Hz), 129.4, 128.7, 128.1, 127.3, 127.1, 126.7 (d, $J_{C–F}$ = 3.0 Hz), 115.7 (d,

$J_{C–F}$ = 22.0 Hz), 77.7 (CH), 21.3 (CH_3); ^{19}F NMR (471 MHz, $CDCl_3$, 298 K) δ: -105.39 (Ar–F); IR ν_{max} (cm^{-1}): 3053, 2097, 1718 (C=O), 1602, 1506, 1450, 1411, 1309, 1261, 1236, 1151, 1107, 1087, 1014; HRMS (EI+) $[M]^+$ $[C_{21}H_{17}FO_2]^+$: calculated 320.1213, found 320.1207.

Synthesis of 1-(naphthalen-2-yl)ethyl 4-fluorobenzoate (**1g**)[3]

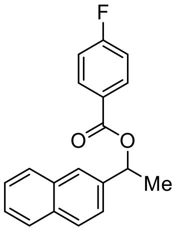

Synthesized in accordance with *General Procedure a* using 4-fluorobenzoyl chloride (2.3 mL, 20.2 mmol), 1-(naphthalen-2-yl)ethan-1-ol (3.17 g, 18.4 mmol), and pyridine (15 mL). All volatiles were removed *in vacuo* and the crude compound was purified *via* column chromatography using silica gel and hexane/ ethyl acetate (90:10 v/v) as eluent. The desired product **1g** was obtained as a while solid. Yield: 2 g, 6.8 mmol, 37%.

^{1}H NMR (500 MHz, $CDCl_3$, 298 K) δ: 8.16–8.10 (m, 2H, Ar–CH), 7.92–7.81 (m, 4H, Ar–CH), 7.58 (dd, J = 8.5, 1.8 Hz, 1H, Ar–CH), 7.53–7.46 (m, 2H, Ar–CH), 7.16–7.09 (m, 2H, Ar–CH), 6.30 (q, J = 6.6 Hz, 1H, Ar–CH), 1.77 (d, J = 6.6 Hz, 3H, CH_3); ^{13}C NMR (126 MHz, $CDCl_3$, 298 K) δ: 165.9 (d, J = 254.5 Hz), 165.0, 139.0, 133.2 (d, J = 13.8 Hz), 132.3 (d, J = 8.8 Hz), 128.6, 128.1, 127.8, 126.3 (d, J = 18.9 Hz), 125.2, 124.1, 115.6 (d, J = 22.0 Hz), 73.4 (CH), 22.4 (CH_3); ^{19}F NMR (471 MHz, $CDCl_3$, 298 K) δ: -105.69 (Ar–F); HRMS (EI+) $[M^+]$ $[C_{19}H_{15}O_2F]^+$: calculated 294.1056, found: 294.1061.

Synthesis of cyclohexyl(phenyl)methyl 4-fluorobenzoate (**1h**)[3]

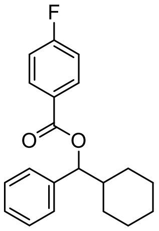

Synthesized in accordance with *General Procedure a* using 4-fluorobenzoyl chloride (3.4 mL, 28.9 mmol), cyclohexyl(phenyl)methanol (5 g, 26.3 mmol), and pyridine (18 mL). All volatiles were removed *in vacuo* and the crude compound was purified *via* column chromatography using silica gel and hexane/ethyl acetate (95:05 v/v) as eluent. The desired product **1h** was obtained as a white solid. Yield: 5.4 g, 17.4 mmol, 66%.

^{1}H NMR (500 MHz, $CDCl_3$, 298 K) δ: 8.12–8.08 (m, 2H, Ar–CH), 7.37–7.32 (m, 4H, Ar–CH), 7.29–7.26 (m, 1H, Ar–CH), 7.13–7.10 (m, 2H, Ar–CH), 5.73 (d, J = 7.5 Hz, 1H, CH), 1.95–1.88 (m, 2H, CH_2), 1.78–1.65 (m, 3H, CH_2), 1.50–1.48 (m, 1H, CH), 1.29–1.12 (m, 4H, CH_2), 1.02 (qd, J = 12.3, 3.8 Hz, 1H, CH); ^{13}C NMR (126 MHz, $CDCl_3$, 298 K) δ: 165.8 (d, $J_{C–F}$ = 254.5 Hz), 165.0, 139.7, 132.2 (d, $J_{C–F}$ = 10.0 Hz), 128.3, 127.9, 127.1, 126.9 (d, $J_{C–F}$ = 3.0 Hz), 115.6 (d, $J_{C–F}$ = 22.6 Hz), 81.1 (CH), 43.3 (CH), 29.2 (CH_2), 29.1 (CH_2), 26.4 (CH_2), 26.05 (CH_2), 26.00 (CH_2); ^{19}F NMR (471 MHz, $CDCl_3$, 298 K) δ: -105.88 (Ar–F); IR ν_{max} (cm$^-$

[1]): 2939, 2848, 1720 (C=O), 1600, 1504, 1448, 1292, 1253, 1238, 1219, 1153, 1107, 1087, 1053, 1012; HRMS (EI+) $[M]^+$ $[C_{20}H_{21}O_2F]^+$: calculated 312.1526, found 312.1519.

Synthesis of di-p-tolylmethyl 4-fluorobenzoate (**1i**)

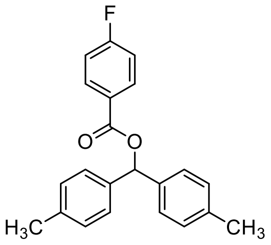

Synthesized in accordance with *General Procedure a* using 4-fluorobenzoyl chloride (2.7 mL, 23.2 mmol), di-*p*-tolylmethanol (4.24 g, 20.0 mmol), and pyridine (25 mL). All volatiles were removed *in vacuo* and the crude product was purified *via* column chromatography using silica gel and hexane/ethyl acetate (90:10 v/v) as eluent. The desired product **1i** was obtained as a colorless oil. Yield: 5.68 g, 17.0 mmol, 85%.

^{1}H NMR (500 MHz, $CDCl_3$, 298 K) δ: 8.04 (ddd, J = 8.9, 5.3, 2.5 Hz, 2H, Ar–CH), 7.22 (m, 4H, Ar–CH), 7.06 (m, 4H, Ar–CH), 7.04–6.95 (m, 3H, Ar–CH and CH), 2.22 (s, 6H, CH_3); ^{13}C NMR (126 MHz, $CDCl_3$, 298 K) δ: 166.0 (d, $J_{C–F}$ = 254.1 Hz), 164.8 (C=O), 137.8, 137.5, 132.4 (d, $J_{C–F}$ = 9.3 Hz), 129.4, 128.4, 128.0, 127.2, 126.8, 125.9, 115.7 (d, $J_{C–F}$ = 22.0 Hz), 77.6 (CH), 21.3 (CH_3); ^{19}F NMR (476 MHz, $CDCl_3$, 298 K) δ: -105.39 (Ar–F); IR ν_{max} (cm^{-1}): 3026, 2965, 2922, 1721 (C=O), 1604, 1506, 1311, 1259, 1237, 1180, 1152, 1102, 1088, 1014.

Synthesis of bis(4-(trifluoromethyl)phenyl)methyl 4-fluorobenzoate (**1j**)

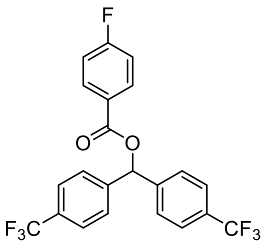

Synthesized in accordance with *General Procedure a* using 4-fluorobenzoyl chloride (1.4 mL, 13.1 mmol), bis(4-(trifluoromethyl) phenyl)methanol (3.5 g, 10.9 mmol), and pyridine (20 mL). All volatiles were removed *in vacuo* and the crude compound was purified *via* column chromatography using silica gel and hexane/ethyl acetate (20:1 v/v) as eluent: The desired product **1j** was obtained as a white solid. Yield: 4.24 g, 9.6 mmol, 88%.

^{1}H NMR (500 MHz, $CDCl_3$, 298 K) δ: 8.15 (dd, J = 8.8, 5.4 Hz, 2H, Ar–CH), 7.65 (d, J = 8.2 Hz, 4H, Ar–CH), 7.54 (d, J = 8.0 Hz, 4H, Ar–CH), 7.18–7.14 (m, 3H, Ar–CH and CH); ^{13}C NMR (126 MHz, $CDCl_3$, 298 K) δ: 166.2 (d, $J_{C–F}$ = 255.3 Hz), 164.4 (C=O), 143.2, 132.5 (d, $J_{C–F}$ = 9.4 Hz), 130.7 (q, $J_{C–F}$ = 32.7 Hz), 127.5, 126.0 (q, $J_{C–F}$ = 3.7 Hz), 125.7 (d, $J_{C–F}$ = 3.0 Hz), 123.9 (q, $J_{C–F}$ = 272.3 Hz), 116.0 (d, $J_{C–F}$ = 22.0 Hz), 76.3 (CH); ^{19}F NMR (471 MHz, $CDCl_3$, 298 K) δ: -62.73, -104.17; IR ν_{max} (cm^{-1}): 3109, 2978, 1724 (C=O), 1620, 1605, 1508, 1413, 1323, 1259, 1239, 1168, 1154, 1128, 1112, 1089, 1066, 1018; HRMS (ASAP-) $[M–H]^-$ calculated for $[C_{22}H_{12}O_2F_7]^-$: 441.0726, found: 441.0724.

Synthesis of (E)-1,3-diphenylallyl 2,2,2-trifluoroacetate (**1k**)[4]

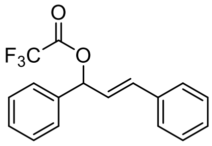

Pyridine (1.4 mL, 14.3 mmol, 1.5 equiv.) was added to a stirred CH_2Cl_2 (25 mL) solution of (*E*)-1,3-diphenylprop-2-en-1-ol (2 g, 1 equiv.) at 0 °C. The reaction mixture was allowed to stir for 15 min under nitrogen at same temperature. Trifluoroacetic anhydride (2 mL, 14.3 mmol, 1.5 equiv.) was added to the reaction mixture dropwise at 0 °C. The reaction mixture was allowed to stir over night at ambient temperature and quenched the reaction with saturated aq. $NaHCO_3$ solution (1 × 30 mL). The organic compounds were extracted with ethyl acetate (3 × 25 mL), the combined organic fractions were washed with brine solution (1 × 30 mL), dried over $MgSO_4$ and concentrated using vacuum. The crude compound was purified *via* column chromatography using silica gel (Merck, 60 Å, 230–400 mesh particle size) and hexane/ethyl acetate as eluent. The desired compound **1k** was obtained as thick liquid which was recrystallized using pentane at -30 °C. A white solid was obtained as pure compound. Yield: 2 g, 6.7 mmol, 71%.

^{1}H NMR (500 MHz, $CDCl_3$, 298 K) δ: 7.46–7.43 (m, 2H, Ar–CH), 7.41–7.36 (m, 4H, Ar–CH), 7.33–7.29 (m, 3H, Ar–CH), 7.26–7.23 (m, 1H, Ar–CH), 6.61 (dd, *J* = 15.8, 5.0 Hz, 1H, CH), 6.36 (ddd, *J* = 26.2, 15.9, 7.1 Hz, 1H, CH), 5.11 (dd, *J* = 9.6, 7.2 Hz, 1H, CH); ^{13}C NMR (126 MHz, $CDCl_3$, 298 K) δ: 141.39, 141.31, 136.7 (d, $J_{C–F}$ = 2.1 Hz), 131.6 (d, $J_{C–F}$ = 22.7 Hz), 130.5 (d, $J_{C–F}$ = 21.1 Hz), 128.69, 128.67, 127.87 (d, $J_{C–F}$ = 1.9 Hz), 127.82, 127.2, 126.7 (d, $J_{C–F}$ = 2.3 Hz), 79.3, 79.2; ^{19}F NMR (471 MHz, $CDCl_3$, 298 K) δ: -75.15 (CF_3); IR ν_{max} (cm^{-1}): 3061, 3026, 1651, 1598, 1492, 1448, 1296, 1093, 1068, 1024.

Synthesis of 1-phenyl-3-(trimethylsilyl)prop-2-yn-1-yl 4-fluorobenzoate (**1l**)[4]

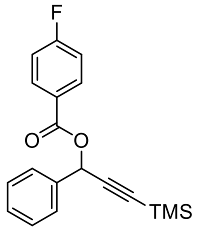

TMS acetylene (3.2 mL, 22.6 mmol, 1.2 equiv.) was dissolved in dry tetrahydrofuran (25 mL) and the reaction mixture was cooled to 0 °C. ^{n}BuLi (9.1 mL, 22.8 mmol, 2.5 M in hexane, 1.2 equiv.) was added dropwise to the reaction mixture at 0 °C. The reaction mixture was stirred for 1 h at ambient temperature. The mixture was cooled down to 0 °C and the benzaldehyde (2 g, 18.8 mmol, 1 equiv.) was added dropwise, allowed the reaction mixture to warm to ambient temperature and stirred for additional 2 h. 4-Fluorobenzoyl chloride (2.7 mL, 22.6 mmol, 1.2 equiv.) was added to the reaction mixture dropwise at 0 °C. The reaction was stirred at ambient temperature for 15 min. Saturated aqueous NH_4Cl solution was used to quench the reaction. The organic layer was extracted with ethyl acetate (3 × 25 mL). The combined organic fractions were washed with brine solution and dried over $MgSO_4$ and concentrated using vacuum. The crude compound was purified *via* column chromatography using hexane/ethyl acetate (95:5

v/v) as eluent. The desired product **1l** was obtained as a yellow liquid. Yield: 5.11 g, 15.6 mmol, 83%.

^{1}H NMR (500 MHz, $CDCl_3$, 298 K) δ: 8.11–8.08 (m, 2H, Ar–CH), 7.61–7.59 (m, 2H, Ar–CH), 7.42–7.37 (m, 3H, Ar–CH), 7.12–7.08 (m, 2H, Ar–CH), 6.72 (s, 1H, CH), 0.20 (s, 9H, $Si(CH_3)_3$); ^{13}C NMR (126 MHz, $CDCl_3$, 298 K) δ: 166.0 (d, $J_{C–F}$ = 254.3 Hz), 164.56 (C=O), 137.0, 132.6 (d, $J_{C–F}$ = 9.4 Hz), 129.1, 128.8, 127.9, 126.2 (d, $J_{C–F}$ = 3.0 Hz), 115.6 (d, $J_{C–F}$ = 22.0 Hz), 101.2 (C≡C), 93.0 (C≡C), 66.6 (CH), -0.1 ($Si(CH_3)_3$); ^{19}F NMR (471 MHz, $CDCl_3$, 298 K) δ: -105.17 (Ar–F); IR ν_{max} (cm^{-1}): 3062, 2960, 2177 (C≡C), 1720 (C=O), 1602, 1506, 1454, 1411, 1345, 1256, 1125, 1054; HRMS (EI+) $[M]^+$ $[C_{19}H_{19}FO_2Si]^+$: calculated 326.1138, found 326.1133.

Synthesis of 1-ethynyl-4-vinylbenzene (**4a**) [5]

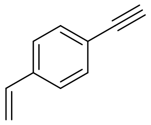

To a solution of trimethyl((4-vinylphenyl)ethynyl)silane (1 g, 5.0 mmol, 1.0 equiv.) in dry THF (5 mL), 1.0 M solution of tetra-*n*-butyl ammonium fluoride (8 mL, 8 mmol, 1.6 equiv.) was added dropwise and the reaction was stirred at ambient temperature for 1 h under nitrogen atmosphere. After completion, the reaction mixture was quenched with water (15 mL), and the organic layer was extracted with CH_2Cl_2 (3 × 15 mL). The combined organic layers were washed with saturated brine solution (1 × 15 mL), dried over anhydrous $MgSO_4$ and concentrated *in vacuo*. The crude product was purified by flash column chromatography using hexane as an eluent. The desired product **4a** was obtained as a colorless liquid. Yield: 570 mg, 4.5 mmol, 89%.

^{1}H NMR (500 MHz, $CDCl_3$, 298 K) δ: 7.45 (d, J = 8.3 Hz, 2H, Ar–CH), 7.36 (d, J = 8.3 Hz, 2H, Ar–CH), 6.70 (dd, J = 17.6, 10.9 Hz, 1H, CH_2), 5.78 (d, J = 17.6 Hz, 1H, CH_2), 5.31 (dd, J = 10.9, 0.8 Hz, 1H, CH_2), 3.11 (s, 1H, CH); ^{13}C NMR (126 MHz, $CDCl_3$, 298 K) δ: 138.1, 136.3, 132.5, 126.2, 121.4, 115.2, 83.8 (C≡C), 77.9 (C≡C).

Synthesis of 1-ethenyl-4-(2-phenylethynyl)benzene (**4b**)[6]

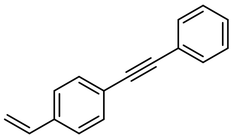

In an oven dried Schlenk flask, 1-bromo-4-vinylbenzene (3 mL, 22.9 mmol, 1 equiv.), CuI (436 mg, 2.3 mmol, 0.1 equiv.) and $PdCl_2(PPh_3)_2$ (805 mg, 1.1 mmol, 0.05 equiv.) were all dissolved in NEt_3 (40 mL) and the resulting solution was stirred for 10 minutes at room temperature under a nitrogen atmosphere. Phenylacetylene (3 mL, 27.5 mmol, 1.2 equiv) was added dropwise to vigorously stirred reaction mixture. The reaction mixture was then heated at 70 °C for next 12 h and

subsequently quenched with water. The organic layer was extracted with diethyl ether (3 × 20 mL) and the combined organic layers were washed with brine solution (1 × 20 mL), and dried over $MgSO_4$. All volatiles were removed *in vacuo* and the crude compound was purified by flash column chromatography using hexane as eluent. The desired product **4b** was obtained as a white solid. Yield: 2.63 g, 12.8 mmol, 56%.

^{1}H NMR (500 MHz, $CDCl_3$, 298 K) δ: 7.55–7.53 (m, 2H, Ar–CH), 7.51–7.49 (m, 2H, Ar–CH), 7.40–7.39 (m, 2H, Ar–CH), 7.36–7.34 (m, 3H, Ar–CH), 6.72 (dd, *J* = 17.6, 10.9 Hz, 1H, CH_2), 5.79 (d, *J* = 17.6 Hz, 1H, CH_2), 5.30 (d, *J* = 10.9 Hz, 1H, CH_2); ^{13}C NMR (126 MHz, $CDCl_3$, 298 K) δ: 137.5, 136.4, 131.9, 131.7, 128.5, 128.4, 126.3, 123.4, 122.7, 114.9, 90.2 (C≡C), 89.5 (C≡C).

Synthesis of 4-(trimethylsilyl)ethynylstyrene (**4c**)[5]

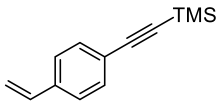

In an oven dried Schlenk flask, 1-bromo-4-vinylbenzene (2 mL, 15.3 mmol, 1 equiv.), CuI (291 mg, 1.5 mmol, 0.1 equiv) and $PdCl_2(PPh_3)_2$ (536 mg, 0.76 mmol, 0.05 equiv.) were all dissolved in NEt_3 (30 mL) and the resulting solution was stirred for 10 minutes at room temperature under a nitrogen atmosphere. Ethynyltrimethylsilane (2.5 mL, 18.3 mmol, 1.2 equiv) was added dropwise to the vigorously stirred reaction mixture. The reaction mixture was then heated at 60 °C for next 16 h and subsequently quenched with water. The organic layer was extracted with diethyl ether (3 × 20 mL), and the combined organic layers were washed with brine solution (1 × 20 mL) and dried over $MgSO_4$. All volatiles were removed *in vacuo* and the crude compound was purified by flash column chromatography using hexane as eluent. The desired product **4c** was obtained as a yellow liquid. Yield: 1.9 g, 9.4 mmol, 62%.

^{1}H NMR (500 MHz, $CDCl_3$, 298 K) δ: 7.44–7.41 (m, 2H, Ar–CH), 7.35–7.32 (m, 2H, Ar–CH), 6.69 (dd, *J* = 17.7, 10.8 Hz, 1H, CH_2), 5.76 (dd, *J* = 17.6, 0.8 Hz, 1H, CH_2), 5.29 (dd, *J* = 10.9, 0.8 Hz, 1H, CH_2), 0.26 (s, 9H, $Si(CH_3)_3$); ^{13}C NMR (126 MHz, $CDCl_3$, 298 K) δ: 137.7, 136.3, 132.2, 126.1, 122.5, 114.9, 105.2 (C≡C), 94.9 (C≡C), 0.1 ($Si(CH_3)_3$).

Synthesis of 1-ethynyl-2-vinylbenzene (**4d**)[7]

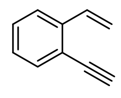

To a solution of trimethyl((2-vinylphenyl)ethynyl)silane (2 g, 10.0 mmol, 1.0 equiv.) in MeOH (17 mL), K_2CO_3 (1.66 g , 12.0 mmol, 1.2 equiv.) was added in one portion and the reaction was stirred at ambient temperature for 30 minutes under nitrogen atmosphere. The reaction mixture was quenched with water (20 mL), and the organic layer was

extracted with hexane (3 × 20 mL). The combined organic layers were washed with saturated brine solution (1 × 20 mL), dried over anhydrous $MgSO_4$ and concentrated *in vacuo*. The crude product was purified by flash column chromatography using hexane as an eluent. The desired product **4d** was obtained as a colourless liquid. Yield: 1.05 g, 8.2 mmol, 82%.

^{1}H NMR (500 MHz, $CDCl_3$, 298 K) δ: 7.57 (d, J = 7.9 Hz, 1H, Ar–CH), 7.48 (d, J = 7.8 Hz, 1H, Ar–CH), 7.31 (t, J = 7.7 Hz, 1H, Ar–CH), 7.25–7.18 (m, 2H, Ar–CH), 5.81 (d, J = 17.6 Hz, 1H, CH_2), 5.36 (d, J = 11.0 Hz, 1H, CH_2), 3.30 (s, 1H, CH); ^{13}C NMR (126 MHz, $CDCl_3$, 298 K) δ: 139.8, 134.8, 133.2, 129.0, 127.5, 124.7, 120.9, 115.9, 82.0 (C≡C), 81.8 (C≡C).

Synthesis of trimethyl(2-phenylethynyl)silane[8]

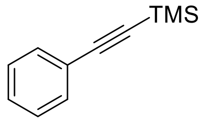

In an oven dried Schlenk flask, bromobenzene (3 mL, 19.1 mmol, 1 equiv.), CuI (145 mg, 0.76 mmol, 0.04 equiv.), $PdCl_2(PPh_3)_2$ (268 mg, 0.38 mmol, 0.02 equiv.) were dissolved in NEt_3 (30 mL) and the mixture was stirred for 10 minutes at room temperature under a nitrogen atmosphere. Ethynyltrimethylsilane (3.2 mL, 22.9 mmol, 1.2 equiv.) was added dropwise to the vigorously stirred reaction mixture. The reaction mixture was heated to 80 ˚C for 6 h. After completion, the reaction was quenched with water. The organic layers were extracted with diethyl ether (3 × 20 mL) and the combined organic layers were washed with brine solution (1 × 20 mL) and dried over $MgSO_4$. All volatiles were removed *in vacuo* and the crude compound was purified by flash column chromatography using hexane as eluent. The desired product was obtained as a yellow liquid. Yield: 2.4 g, 13.7 mmol, 72%.

^{1}H NMR (500 MHz, $CDCl_3$, 298 K) δ: 7.48–7.46 (m, 2H, Ar–CH), 7.32–7.27 (m, 3H, Ar–CH), 0.26 (s, 9H, $Si(CH_3)_3$); ^{13}C NMR (126 MHz, $CDCl_3$, 298 K) δ: 132.1, 128.6, 128.3, 123.2, 105.2 (C≡C), 94.2 (C≡C), 0.1 ($Si(CH_3)_3$).

2. Product Characterization

2.1 General Procedure b: C–C cross-coupling reaction: Tris(pentafluorophenyl)borane [$B(C_6F_5)_3$] (51 mg, 0.1 mmol, 1 equiv.) and trimesitylphosphine [Mes_3P] (39 mg, 0.1 mmol, 1 equiv.) were dissolved separately in THF (0.3 mL) and added to the reaction tube. Aryl esters (1 equiv) and aryl acetylene (1.2 equiv) were dissolved separately in THF/TFT (0.3 mL) and added together same time dropwise to the reaction tube at ambient temperature. The reaction tube was sealed in the glove box under nitrogen atmosphere and heated at 80 ^{o}C for 22–24 h in

fume cupboard. All volatiles were removed *in vacuo* and the crude compound was purified *via* preparative thin layer chromatography using hexane/ethyl acetate as eluent.
[when TFT was used as solvent for C–C cross coupling reaction, 0.6 mL of TFT was taken to dissolve each reactant]

2.2 Synthesis and spectral characterization of C–C cross coupled compounds

Synthesis of 4,4',4''-(prop-2-yne-1,1,3-triyl)tris(fluorobenzene) (**2a**)

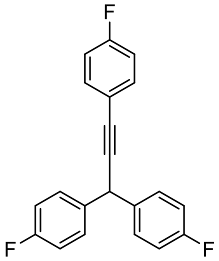

Synthesized in accordance with *General Procedure b* using $B(C_6F_5)_3$ (51 mg, 0.1 mmol), Mes_3P (39 mg, 0.1 mmol) 1-ethynyl-4-fluorobenzene (15 mg, 0.12 mmol) and aryl ester **1a** (34 mg, 0.1 mmol) in THF to afford **2a**. The crude reaction mixture was purified *via* preparative thin layer chromatography using hexane/ethyl acetate (95:05 v/v) as eluent. The desired compound **2a** was obtained as a pale-yellow liquid. Yield: 26 mg, 0.08 mmol, 82%.
^{1}H NMR (400 MHz, $CDCl_3$, 298 K) δ: 7.39–7.30 (m, 3H, Ar–CH), 7.29–7.22 (m, 5H, Ar–CH), 7.21–7.16 (m, 2H, Ar–CH), 6.93 (ddt, J = 9.9, 8.7, 1.5 Hz, 2H, Ar–CH), 5.09 (s, 1H, CH); ^{13}C NMR (101 MHz, $CDCl_3$, 298 K) δ: 162.6 (d, $J_{C–F}$ = 249.2 Hz), 141.3, 140.3, 133.7 (d, $J_{C–F}$ = 8.3 Hz), 132.9, 129.4, 128.9, 127.9, 127.3, 119.4 (d, $J_{C–F}$ = 3.6 Hz), 115.7 (d, $J_{C–F}$ = 22.0 Hz), 89.4 (C≡C), 84.3 (C≡C), 43.2 (CH); ^{19}F NMR (376 MHz, $CDCl_3$, 298 K) δ: -111.08 (s, 1F, Ar–F), -115.69 (s, 2F, Ar–F); IR ν_{max} (cm^{-1}): 3033, 2902, 2184 (C≡C), 1633, 1556, 1502, 1477, 1423, 1333, 1302, 1256, 1221, 1165, 1031; HRMS (ES+) $[M+H]^+$ $[C_{21}H_{14}F_3]^+$: calculated 323.1048, found 323.1045.

Synthesis of 4,4',4''-(prop-2-yne-1,1,3-triyl)tris(fluorobenzene) (**2b**)

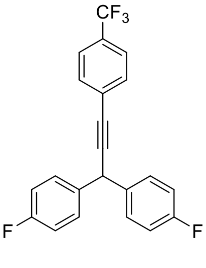

Synthesized in accordance with *General Procedure b* using $B(C_6F_5)_3$ (51 mg, 0.1 mmol), Mes_3P (39 mg, 0.1 mmol) 1-ethynyl-4-(trifluoromethyl)benzene (21 mg, 0.12 mmol) and aryl ester **1a** (34 mg, 0.1 mmol) in THF to afford **2b**. The crude reaction mixture was purified *via* preparative thin layer chromatography using hexane/ethyl acetate (98:02 v/v) as eluent. The desired compound **2b** was obtained as a colorless liquid. Yield: 23 mg, 0.06 mmol, 61%.
^{1}H NMR (500 MHz, $CDCl_3$, 298 K) δ: 7.57 (s, 4H, Ar–CH), 7.42 – 7.32 (m, 4H, Ar–CH), 7.02 (q, J = 8.2 Hz, 4H, Ar–CH), 5.20 (s, 1H, CH); ^{13}C NMR (125 MHz, $CDCl_3$, 298 K) δ: 162.1 (d, $J_{C–F}$ = 245.7 Hz), 136.9 (d, $J_{C–F}$ = 3.3 Hz), 132.1, 129.5 (d, $J_{C–F}$ = 8.7 Hz), 128.9 (d, $J_{C–F}$ = 8.7 Hz), 127.0, 125.4, 115.8 (d, $J_{C–F}$ = 21.2 Hz), 92.4 (C≡C), 84.1 (C≡C), 42.4 (CH); ^{19}F NMR

(471 MHz, $CDCl_3$, 298 K) δ -62.82 (s, 3F, Ar–CF_3), -115.42 (s, 2F, Ar–F); IR ν_{max} (cm^{-1}): 3015, 2923, 2180 (C≡C), 1685, 1536, 1498, 1431, 1401, 1384, 1325, 1212, 1130, 1022; HRMS (ES+) $[M+H]^+$ $[C_{22}H_{14}F_5]^+$: calculated 373.1016, found 373.1019.

Synthesis of 4,4'-(3-phenylprop-2-yne-1,1-diyl)bis(fluorobenzene) (**2c**)[9]

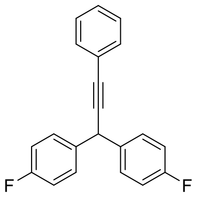

Synthesized in accordance with *General Procedure b* using $B(C_6F_5)_3$ (51 mg, 0.1 mmol), Mes_3P (39 mg, 0.1 mmol) phenylacetylene (12 mg, 0.12 mmol) and aryl ester **1a** (34 mg, 0.1 mmol) in THF to afford **2c**. The crude reaction mixture was purified *via* preparative thin layer chromatography using hexane/ethyl acetate (95:05 v/v) as eluent. The desired compound **2c** was obtained as a white solid. Yield: 26 mg, 0.08 mmol, 84%.

^{1}H NMR (400 MHz, $CDCl_3$, 298 K) δ: 7.48–7.46 (m, 2H, Ar–CH), 7.39–7.36 (m, 4H, Ar–CH), 7.33–7.31 (m, 3H, Ar–CH), 7.05–7.00 (m, 4H, Ar–CH), 5.18 (s, 1H, CH); ^{13}C NMR (101 MHz, $CDCl_3$, 298 K) δ: 162.0 (d, $J_{C–F}$ = 245.7 Hz), 137.5 (d, $J_{C–F}$ = 3.0 Hz), 131.8, 129.5 (d, $J_{C–F}$ = 8.1 Hz), 128.4, 128.4, 123.3, 115.6 (d, $J_{C–F}$ = 21.5 Hz), 89.8 (C≡C), 85.5 (C≡C), 42.4 (CH); ^{19}F NMR (376 MHz, $CDCl_3$, 298 K) δ: -115.81 (Ar–F); IR ν_{max} (cm^{-1}): 3002, 2921, 2188 (C≡C), 1602, 1522, 1454, 1435, 1321, 1311, 1254, 1218, 1152, 1028; HRMS (EI+) $[M]^+$ $[C_{21}H_{14}F_2]^+$: calculated 304.1064, found 304.1058.

Synthesis of 4,4'-(3-(4-methoxyphenyl)prop-2-yne-1,1-diyl)bis(fluorobenzene) (**2d**)[10]

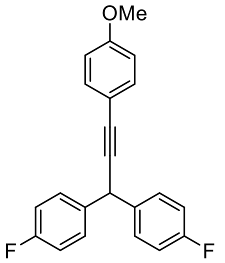

Synthesized in accordance with *General Procedure b* using $B(C_6F_5)_3$ (51 mg, 0.1 mmol), Mes_3P (39 mg, 0.1 mmol) 1-ethynyl-4-methoxybenzene (16 mg, 0.12 mmol) and aryl ester **1a** (34 mg, 0.1 mmol) in THF to afford **2d**. The crude reaction mixture was purified *via* preparative thin layer chromatography using hexane/ethyl acetate (92:08 v/v) as eluent. The desired compound **2d** was obtained as a yellow liquid. Yield: 24 mg, 0.07 mmol, 72%.

^{1}H NMR (400 MHz, $CDCl_3$, 298 K) δ: 7.33–7.27 (m, 6H, Ar–CH), 6.92 (d, J = 8.4 Hz, 4H, Ar–CH), 6.76 (d, J = 8.4 Hz, 2H, Ar–CH), 5.07 (s, 1H, CH), 3.72 (s, 3H, OCH_3);^{13}C NMR (101 MHz, $CDCl_3$, 298 K) δ: 162.0 (d, $J_{C–F}$ = 245.6 Hz), 159.7, 137.7 (d, $J_{C–F}$ = 3.2 Hz), 133.2, 129.5 (d, $J_{C–F}$ = 8.0 Hz), 115.7, 115.5, 115.4, 114.0, 88.3 (C≡C), 85.3 (C≡C), 55.4 (OCH_3), 42.4 (CH); ^{19}F NMR (376 MHz, $CDCl_3$, 298 K) δ: -115.90 (Ar–F); IR ν_{max} (cm^{-1}): 3012, 2954, 2182 (C≡C), 1621, 1535, 1444, 1425, 1333, 1316, 1241, 1208, 1135, 1033; HRMS (ES+) $[M+H]^+$ $[C_{22}H_{17}F_2O]^+$: calculated 335.1247, found 335.1246.

Synthesis of 4,4'-(3-(4-fluorophenyl)prop-2-yne-1,1-diyl)bis(chlorobenzene) (**2e**)

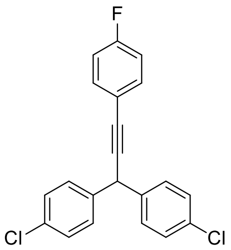

Synthesized in accordance with *General Procedure b* using $B(C_6F_5)_3$ (51 mg, 0.1 mmol), Mes_3P (39 mg, 0.1 mmol) 1-ethynyl-4-fluorobenzene (15 mg, 0.12 mmol) and aryl ester **1b** (37 mg, 0.1 mmol) in THF to afford **2e**. The crude reaction mixture was purified *via* preparative thin layer chromatography using hexane/ethyl acetate (95:05 v/v) as eluent. The desired compound **2e** was obtained as a colorless liquid. Yield: 27 mg, 0.08 mmol, 77%.

^{1}H NMR (500 MHz, $CDCl_3$, 298 K) δ: 7.44 (dd, *J* = 8.4, 5.4 Hz, 2H, Ar–CH), 7.31 (q, *J* = 8.5 Hz, 8H, Ar–CH), 7.01 (t, *J* = 8.5 Hz, 2H, Ar–CH), 5.14 (s, 1H, CH);^{13}C NMR (126 MHz, $CDCl_3$, 298 K) δ: 162.6 (d, $J_{C–F}$ = 249.8 Hz), 139.8, 133.7 (d, $J_{C–F}$ = 8.7 Hz), 133.2, 129.2 (d, $J_{C–F}$ = 41.8 Hz), 115.7, 88.8 (C≡C), 84.6 (C≡C), 42.6 (CH); ^{19}F NMR (471 MHz, $CDCl_3$, 298 K) δ: -110.92 (Ar–F); IR ν_{max} (cm^{-1}): 3023, 2966, 2172 (C≡C), 1685, 1610, 1542, 1425, 1401, 1346, 1361, 1223, 1211, 1154, 1021; HRMS (ES+) [M+H]$^+$ $[C_{21}H_{14}Cl_2F]^+$: calculated 355.0457, found 355.0459.

Synthesis of 4,4'-(3-phenylprop-2-yne-1,1-diyl)bis(chlorobenzene) (**2f**)

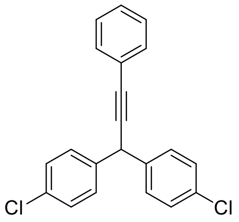

Synthesized in accordance with *General Procedure b* using $B(C_6F_5)_3$ (51 mg, 0.1 mmol), Mes_3P (39 mg, 0.1 mmol) phenylacetylene (12 mg, 0.12 mmol) and aryl ester **1b** (37 mg, 0.1 mmol) in THF to afford **2f**. The crude reaction mixture was purified *via* preparative thin layer chromatography using hexane/ethyl acetate (95:05 v/v) as eluent. The desired compound **2f** was obtained as a yellow liquid. Yield: 28 mg, 0.08 mmol, 83%.

^{1}H NMR (500 MHz, $CDCl_3$, 298 K) δ: 7.48–7.44 (m, 2H, Ar–CH), 7.37–7.29 (m, 11H, Ar–CH), 5.15 (s, 1H, CH);^{13}C NMR (125 MHz, $CDCl_3$, 298 K) δ: 139.9, 133.1, 131.8, 129.3, 129.0, 128.5, 123.1, 89.1 (C≡C), 85.7 (C≡C), 42.7 (CH); IR ν_{max} (cm^{-1}): 3021, 2978, 2172 (C≡C), 1654, 1602, 1544, 1441, 1405, 1364, 1302, 1287, 1232, 1174, 1031; HRMS (EI+) [M]$^+$ $[C_{21}H_{14}Cl_2]$: calculated 336.0473, found 336.0465.

Synthesis of 4,4'-(3-(4-methoxyphenyl)prop-2-yne-1,1-diyl)bis(chlorobenzene) (**2g**)

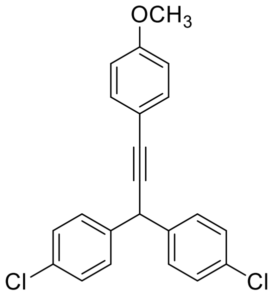

Synthesized in accordance with *General Procedure b* using $B(C_6F_5)_3$ (51 mg, 0.1 mmol), Mes_3P (39 mg, 0.1 mmol) 1-ethynyl-4-methoxybenzene (16 mg, 0.12 mmol) and aryl ester **1b** (37 mg, 0.1 mmol) in THF to afford **2g**. The crude reaction mixture was purified *via* preparative thin layer chromatography using hexane/ethyl acetate (95:05 v/v) as eluent. The desired compound **2g** was obtained as a pale-yellow liquid. Yield: 27 mg, 0.07 mmol, 77%.

^{1}H NMR (500 MHz, $CDCl_3$, 298 K) δ: 7.81–7.58 (m, 4H, Ar–CH), 7.55–7.45 (m, 4H, Ar–CH), 7.41–7.38 (m, 1H, Ar–CH), 7.30–7.29 (m, 1H, Ar–CH), 7.03–6.77 (m, 2H, Ar–CH), 5.13 (s, 1H, CH), 3.81 (s, 3H, (OCH_3); ^{13}C NMR (125 MHz, $CDCl_3$, 298 K) δ: 159.7, 140.1, 139.3, 135.6, 133.2, 133.1, 131.5, 129.3, 128.9, 115.2, 114.1, 87.6 (C≡C), 85.6 (C≡C), 55.5 (OCH_3), 42.7 (CH); IR ν_{max} (cm^{-1}): 3023, 2987, 2174 (C≡C), 1654, 1578, 1456, 1433, 1378, 1305, 1256, 1215, 1147, 1032; HRMS (ES$^-$) [M-H]$^-$ $[C_{22}H_{15}Cl_2O]^-$: calculated 365.0500, found 365.0493.

Synthesis of (3-(4-fluorophenyl)prop-2-yne-1,1-diyl)dibenzene (**2h**)[11]

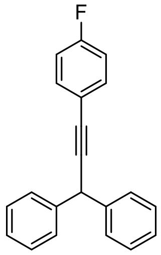

Synthesized in accordance with *General Procedure b* using $B(C_6F_5)_3$ (51 mg, 0.1 mmol), Mes_3P (39 mg, 0.1 mmol) 1-ethynyl-4-fluorobenzene (15 mg, 0.12 mmol) and aryl ester **1c** (31 mg, 0.1 mmol) in THF to afford **2h**. The crude reaction mixture was purified *via* preparative thin layer chromatography using hexane/ethyl acetate (95:05 v/v) as eluent. The desired compound **2h** was obtained as a yellow oil. Yield: 23 mg, 0.08 mmol, 80%.

^{1}H NMR (400 MHz, $CDCl_3$, 298 K) δ: 7.39–7.35 (m, 5H, Ar–CH), 7.25 (t, J = 7.7 Hz, 4H, Ar–CH), 7.19–7.14 (m, 3H, Ar–CH), 6.94–6.90 (m, 2H, Ar–CH), 5.13 (s, 1H, CH);^{13}C NMR (101 MHz, $CDCl_3$, 298 K) δ: 162.5 (d, $J_{C–F}$ = 248.8 Hz), 141.8, 133.7 (d, $J_{C–F}$ = 8.4 Hz), 128.8, 128.0, 127.1, 119.7 (d, $J_{C–F}$ = 3.6 Hz), 115.6 (d, $J_{C–F}$ = 21.9 Hz), 90.0 (C≡C), 83.9 (C≡C), 43.8 (CH); ^{19}F NMR (376 MHz, $CDCl_3$, 298 K) δ: -111.55 (Ar–F); IR ν_{max} (cm^{-1}): 3030, 2969, 2180 (C≡C), 1630, 1545, 1465, 1402, 1329, 1332, 1245, 1221, 1144, 1028; HRMS (EI+) [M]$^+$ $[C_{21}H_{15}F]^+$: calculated 286.1158, found 286.1158.

Synthesis of (3-(4-(trifluoromethyl)phenyl)prop-2-yne-1,1-diyl)dibenzene (**2i**)[12]

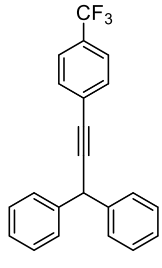

Synthesized in accordance with *General Procedure b* using $B(C_6F_5)_3$ (51 mg, 0.1 mmol), Mes_3P (39 mg, 0.1 mmol) 1-ethynyl-4-(trifluoromethyl)benzene (21 mg, 0.12 mmol) and aryl ester **1c** (31 mg, 0.1 mmol) in THF to afford **2i**. The crude reaction mixture was purified *via* preparative thin layer chromatography using hexane/ethyl acetate (95:05 v/v) as eluent. The desired compound **2i** was obtained as a yellow oil. Yield: 20 mg, 0.06 mmol, 60%.

^{1}H NMR (500 MHz, $CDCl_3$, 298 K) δ: 7.57 (d, *J* = 1.1 Hz, 2H, Ar–CH), 7.53–7.51 (m, 2H, Ar–CH), 7.44–7.52 (m, 2H, Ar–CH), 7.36–7.29 (m, 7H, Ar–CH), 7.27 (td, *J* = 1.3, 0.3 Hz, 1H, Ar–CH), 5.24 (s, 1H, CH);^{13}C NMR (126 MHz, $CDCl_3$, 298 K) δ: 141.4, 134.1, 132.1, 129.7, 128.9, 128.7, 128.5, 128.0, 128.0, 127.9, 127.4, 127.2, 127.1, 125.3 (q, $J_{C–F}$ = 3.9 Hz), 93.1 (C≡C), 83.7 (C≡C), 43.9 (CH); ^{19}F NMR (376 MHz, $CDCl_3$, 298 K) δ: -62.78. (Ar–CF_3); IR ν_{max} (cm^{-1}): 3020, 2969, 2185 (C≡C), 1670, 1566, 1430, 1410, 1334, 1318, 1230, 1202, 1132, 1033; HRMS (EI+) $[M]^+$ $[C_{22}H_{15}F_3]^+$: calculated 336.1126, found 336.1119.

Synthesis of (3-(4-chlorophenyl)prop-2-yne-1,1-diyl)dibenzene (**2j**)[11]

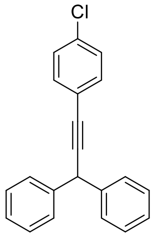

Synthesized in accordance with *General Procedure b* using $B(C_6F_5)_3$ (51 mg, 0.1 mmol), Mes_3P (39 mg, 0.1 mmol) 1-chloro-4-ethynylbenzene (16 mg, 0.12 mmol) and aryl ester **1c** (31 mg, 0.1 mmol) in THF to afford **2j**. The crude reaction mixture was purified *via* preparative thin layer chromatography using hexane/ethyl acetate (95:05 v/v) as eluent. The desired compound **2j** was obtained as a white solid. Yield: 24 mg, 0.08 mmol, 81%.

^{1}H NMR (500 MHz, $CDCl_3$, 298 K) δ: 7.38–7.30 (m, 6H, Ar–CH), 7.28–7.22 (m, 4H, Ar–CH), 7.21–7.14 (m, 4H, Ar–CH), 5.13 (s, 1H, CH);^{13}C NMR (126 MHz, $CDCl_3$, 298 K) δ: 141.6, 134.1, 133.07, 128.8, 128.7, 128.0, 127.1, 122.1, 91.4 (C≡C), 83.9 (C≡C), 43.9 (CH); IR ν_{max} (cm^{-1}): 3022, 2945, 2178 (C≡C), 1655, 1578, 1502, 1444, 1361, 1341, 1277, 1145, 1102, 1031; HRMS (EI+) $[M]^+$ $[C_{21}H_{15}Cl]$: calculated 302.0862, found 302.0861.

Synthesis of prop-2-yne-1,1,3-triyltribenzene (**2k**)[11]

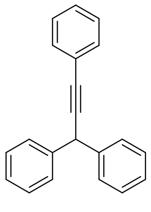

Synthesized in accordance with *General Procedure b* using $B(C_6F_5)_3$ (51 mg, 0.1 mmol), Mes_3P (39 mg, 0.1 mmol) phenylacetylene (12 mg, 0.12 mmol) and aryl ester **1c** (31 mg, 0.1 mmol) in THF to afford **2k**. The crude reaction mixture was purified *via* preparative thin layer chromatography using hexane/ethyl acetate

(95:05 v/v) as eluent. The desired compound **2k** was obtained as a white solid. Yield: 23 mg, 0.08 mmol, 85%.

^{1}H NMR (500 MHz, $CDCl_3$, 298 K) δ: 7.50–7.44 (m, 6H, Ar–CH), 7.35–7.30 (m, 7H, Ar–CH), 7.25–7.22 (m, 2H, Ar–CH), 5.22 (s, 1H, CH);^{13}C NMR (126 MHz, $CDCl_3$, 298 K) δ 141.9, 131.3, 128.8, 128.4, 128.1, 128.0, 127.04, 123.6, 90.3 (C≡C), 85.0 (C≡C), 43.9 (CH); IR ν_{max} (cm^{-1}): 3010, 2915, 2181 (C≡C), 1645, 1528, 1415, 1364, 1328, 1254, 1189, 1142, 1082, 1025; HRMS (EI+) $[M]^+$ $[C_{21}H_{16}]^+$: calculated 268.1252, found 268.1247.

Synthesis of (3-(4-(tert-butyl)phenyl)prop-2-yne-1,1-diyl)dibenzene (**2l**)[13]

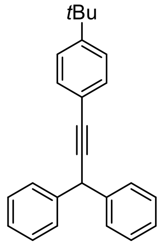

Synthesized in accordance with *General Procedure b* using $B(C_6F_5)_3$ (51 mg, 0.1 mmol), Mes_3P (39 mg, 0.1 mmol) 1-(*tert*-butyl)-4-ethynylbenzene (19 mg, 0.12 mmol) and aryl ester **1c** (31 mg, 0.1 mmol) in THF to afford **2l**. The crude reaction mixture was purified *via* preparative thin layer chromatography using hexane/ethyl acetate (95:05 v/v) as eluent. The desired compound **2l** was obtained as a white solid. Yield: 26 mg, 0.08 mmol, 79%.

^{1}H NMR (500 MHz, $CDCl_3$, 298 K) δ: 7.46–7.42 (m, 6H, Ar–CH), 7.34–7.31 (m, 6H, Ar–CH), 7.25–7.22 (m, 2H, Ar–CH), 5.21 (s, 1H, CH), 1.31 (s, 9H, $C(CH_3)_3$);^{13}C NMR (126 MHz, $CDCl_3$, 298 K) δ: 151.3, 142.0, 137.7, 134.1, 132.6, 131.5, 130.2, 129.7, 128.7, 128.4, 128.0, 127.9, 127.0, 125.4, 120.6, 89.6(C≡C), 85.1(C≡C), 43.9 (CH), 34.9 (quaternary C), 31.3 (CH_3); IR ν_{max} (cm^{-1}): 3025, 2975, 2182 (C≡C), 1603, 1565, 1455, 1332, 1302, 1278, 1220, 1177, 1132, 1025; HRMS (EI+) $[M]^+$ $[C_{25}H_{24}]^+$: calculated 324.1878, found 324.1872.

Synthesis of (3-(4-methoxyphenyl)prop-2-yne-1,1-diyl)dibenzene (**2m**)[12]

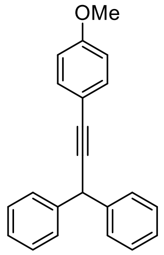

Synthesized in accordance with *General Procedure b* using $B(C_6F_5)_3$ (51 mg, 0.1 mmol), Mes_3P (39 mg, 0.1 mmol) 1-ethynyl-4-methoxybenzene (16 mg, 0.12 mmol) and aryl ester **1c** (31 mg, 0.1 mmol) in THF to afford **2m**. The crude reaction mixture was purified *via* preparative thin layer chromatography using hexane/ethyl acetate (95:05 v/v) as eluent. The desired compound **2m** was obtained as a yellow liquid. Yield: 22 mg, 0.07 mmol, 75%.

^{1}H NMR (400 MHz, $CDCl_3$, 298 K) δ: 7.38–7.32 (m, 6H, Ar–CH), 7.27–7.22 (m, 4H, Ar–CH), 7.17–7.13 (m, 2H, Ar–CH), 6.77–6.74 (m, 2H, Ar–CH), 5.12 (s, 1H, CH), 3.72 (s, 3H, OCH_3);^{13}C NMR (101 MHz, $CDCl_3$, 298 K) δ: 159.5, 142.1, 133.2, 128.7, 128.0, 127.0, 115.8, 114.0, 88.8 (C≡C), 84.8 (C≡C), 55.4 (OCH_3), 43.9 (CH); IR ν_{max} (cm^{-1}): 3017, 2181 (C≡C),

1668, 1525, 1421, 1367, 1255, 1235, 1165, 1118, 1033; HRMS (EI+) $[M]^+$ $[C_{22}H_{18}O]^+$: calculated 298.1358, found 298.1353.

Synthesis of 4,4'-(3-(4-fluorophenyl)prop-2-yne-1,1-diyl)bis(methoxybenzene) (**2n)**

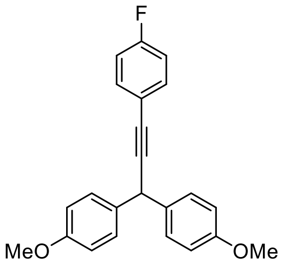

Synthesized in accordance with *General Procedure b* using $B(C_6F_5)_3$ (51 mg, 0.1 mmol), Mes_3P (39 mg, 0.1 mmol) 1-ethynyl-4-fluorobenzene (15 mg, 0.12 mmol) and aryl ester **1d** (37 mg, 0.1 mmol) in THF to afford **2n**. The crude reaction mixture was purified *via* preparative thin layer chromatography using hexane/ethyl acetate (98:02 v/v) as eluent. The desired compound **2n** was obtained as a yellow liquid. Yield: 25 mg, 0.07 mmol, 73%.

^{1}H NMR (500 MHz, $CDCl_3$, 298 K) δ: 7.45–7.40 (m, 2H, Ar–CH), 7.33–7.30 (m, 4H, Ar–CH), 7.01–6.97 (m, 2H, Ar–CH), 6.87–6.84 (m, 4H, Ar–CH), 5.11 (s, 1H, CH), 3.79 (s, 6H, OCH_3);^{13}C NMR (101 MHz, $CDCl_3$, 298 K) δ: 162.4 (d, J_{C-F} = 248.7 Hz), 158.6, 134.3, 133.6 (d, J_{C-F} = 8.3 Hz), 128.9, 115.6 (d, J_{C-F} = 21.9 Hz), 114.1, 90.6 (C≡C), 83.6 (C≡C), 55.4 (OCH_3), 42.1 (CH); ^{19}F NMR (376 MHz, $CDCl_3$, 298 K) δ: -111.71 (Ar–F); IR ν_{max} (cm^{-1}): 3003, 2927, 2186 (C≡C), 1635, 1546, 1454, 1387, 1324, 1232, 1222, 1131, 1165, 1043; HRMS (EI+) $[M]^+$ $[C_{23}H_{20}FO_2]^+$: calculated 347.1447, found 347.1443.

Synthesis of 4,4'-(3-phenylprop-2-yne-1,1-diyl)bis(methoxybenzene) (**2o**)[14]

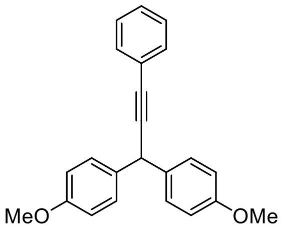

Synthesized in accordance with *General Procedure b* using $B(C_6F_5)_3$ (51 mg, 0.1 mmol), Mes_3P (39 mg, 0.1 mmol) phenylacetylene (12 mg, 0.12 mmol) and aryl ester **1d** (37 mg, 0.1 mmol) in THF to afford **2o**. The crude reaction mixture was purified *via* preparative thin layer chromatography using hexane/ethyl acetate (98:02 v/v) as eluent. The desired compound **2o** was obtained as a yellow oil. Yield: 25 mg, 0.08 mmol, 76%.

^{1}H NMR (400 MHz, $CDCl_3$, 298 K) δ: 7.48–7.43 (m, 3H, Ar–CH), 7.35–7.29 (m, 7H, Ar–CH), 6.86 (d, J = 8.3 Hz, 1H, Ar–CH), 5.13 (s, 1H, CH), 3.79 (s, 6H, OCH_3);^{13}C NMR (101 MHz, $CDCl_3$, 298 K) δ: 158.6, 134.4, 131.8, 131.8, 129.9, 128.9, 128.3, 128.2, 128.0, 123.7, 114.1, 90.9 (C≡C), 84.7 (C≡C), 55.4 (OCH_3), 42.2 (CH); IR ν_{max} (cm^{-1}): 3022, 2956, 2185 (C≡C), 1647, 1510, 1433, 1325, 1287, 1215, 1130, 1156, 1033; HRMS (EI+) $[M]^+$ $[C_{23}H_{21}O_2]^+$: calculated 329.1542, found 329.1537.

Synthesis of 4,4'-(3-(4-(tert-butyl)phenyl)prop-2-yne-1,1-diyl)bis(methoxybenzene) (**2p**)

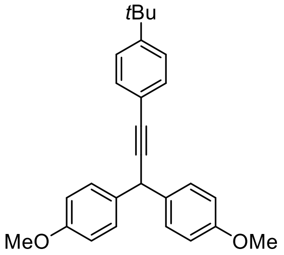

Synthesized in accordance with *General Procedure b* using $B(C_6F_5)_3$ (51 mg, 0.1 mmol), Mes_3P (39 mg, 0.1 mmol) 1-(*tert*-butyl)-4-ethynylbenzene (19 mg, 0.12 mmol) and aryl ester **1d** (37 mg, 0.1 mmol) in THF to afford **2p**. The crude reaction mixture was purified *via* preparative thin layer chromatography using hexane/ethyl acetate (98:02 v/v) as eluent. The desired compound **2p** was obtained as a yellow oil. Yield: 28 mg, 0.07 mmol, 72%.

^{1}H NMR (400 MHz, $CDCl_3$, 298 K) δ: 7.41–7.40 (m, 2H, Ar–CH), 7.34–7.31 (m, 6H, Ar–CH), 6.87–6.84 (m, 4H, Ar–CH), 5.11 (s, 1H. CH), 3.79 (s, 6H, OCH_3), 1.31 (s, 9H, $C(CH_3)_3$);^{13}C NMR (101 MHz, $CDCl_3$, 298 K) δ: 158.5, 151.3, 134.6, 131.5, 129.0, 125.3, 120.7, 114.1, 90.2 (C≡C), 84.7 (C≡C), 55.4 (OCH_3), 42.2 (CH), 34.9 (quaternary C), 31.3 (CH_3); IR ν_{max} (cm^{-1}): 3003, 2933, 2180 (C≡C), 1623, 1545, 1453, 1378, 1322, 1244, 1231, 1178, 1105, 1031; HRMS (EI+) $[M]^+$ $[C_{27}H_{28}O_2]^+$: calculated 384.2089, found 384.2084.

Synthesis of 4,4',4''-(prop-2-yne-1,1,3-triyl)tris(methoxybenzene) (**2q**)

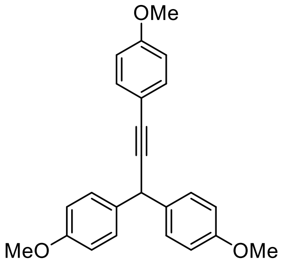

Synthesized in accordance with *General Procedure b* using $B(C_6F_5)_3$ (51 mg, 0.1 mmol), Mes_3P (39 mg, 0.1 mmol) 1-ethynyl-4-methoxybenzene (16 mg, 0.12 mmol) and aryl ester **1d** (37 mg, 0.1 mmol) in THF to afford **2q**. The crude reaction mixture was purified *via* preparative thin layer chromatography using hexane/ethyl acetate (92:08 v/v) as eluent. The desired compound **2q** was obtained as a pale-yellow oil. Yield: 25 mg, 0.07 mmol, 70%.

^{1}H NMR (400 MHz, $CDCl_3$, 298 K) δ: 7.42–7.39 (m, 2H, Ar–CH), 7.36–7.32 (m, 4H, Ar–CH), 6.88– 6.82 (m, 3H, Ar–CH), 5.12 (s, 1H, Ar–CH), 3.81 (s, 3H, OCH_3), 3.79 (s, 6H, OCH_3);^{13}C NMR (101 MHz, $CDCl_3$, 298 K) δ: 159.4, 158.5, 134.6, 133.1, 128.9, 115.9, 114.1, 89.4 (C≡C), 84.4 (C≡C), 55.4 (OCH_3), 42.2 (CH); IR ν_{max} (cm^{-1}): 3010, 2954, 2184 (C≡C), 1645, 1514, 1465, 1366, 1330, 1265, 1210, 1134, 1110, 1033; HRMS (ES+) $[M+H]^+$ $[C_{24}H_{23}O_3]^+$: calculated 359.1647, found 359.1652.

Synthesis of 1-chloro-4-(3-(4-fluorophenyl)-1-phenylprop-2-yn-1-yl)benzene (**2r**)

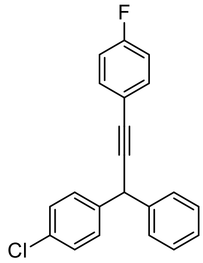

Synthesized in accordance with *General Procedure b* using $B(C_6F_5)_3$ (51 mg, 0.1 mmol), Mes_3P (39 mg, 0.1 mmol) 1-ethynyl-4-fluorobenzene (15 mg, 0.12 mmol) and aryl ester **1e** (34 mg, 0.1 mmol) in THF to afford **2r**. The crude reaction mixture was purified *via* preparative thin layer chromatography using hexane/ethyl acetate (98:02 v/v) as eluent. The desired compound **2r** was obtained as a pale-yellow liquid. Yield: 25 mg, 0.08 mmol, 78%.

^{1}H NMR (400 MHz, $CDCl_3$, 298 K) δ: 7.46–7.39 (m, 3H, Ar–CH), 7.37–7.28 (m, 8H, Ar–CH), 7.03–6.98 (m, 2H, Ar–CH), 5.17 (s, 1H, CH);^{13}C NMR (101 MHz, $CDCl_3$, 298 K) δ: 162.6 (d, J_{C-F} = 249.1 Hz), 141.3, 140.3, 133.7 (d, J_{C-F} = 8.4 Hz), 132.9, 129.4, 128.9, 128.8, 128.7, 128.7, 128.7, 128.6, 127.9, 127.3, 127.2, 119.4 (d, J_{C-F} = 3.5 Hz), 115.7 (d, J_{C-F} = 22.0 Hz), 89.4 (C≡C), 84.3 (C≡C), 43.2 (CH); ^{19}F NMR (376 MHz, $CDCl_3$, 298 K) δ: -111.24 (Ar–F); IR ν_{max} (cm^{-1}): 3005, 2984, 2180 (C≡C), 1622, 1542, 1431, 1321, 1298, 1232, 1254, 1127, 1103, 1031; HRMS (ES+) $[M+H]^+$ $[C_{21}H_{15}ClF]^+$: calculated 321.0846, found 321.0847.

Synthesis of 3-(4-chlorophenyl)prop-1-yne-1,3-diyl dibenzene (**2s**)[11]

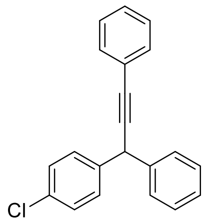

Synthesized in accordance with *General Procedure b* using $B(C_6F_5)_3$ (51 mg, 0.1 mmol), Mes_3P (39 mg, 0.1 mmol) phenylacetylene (12 mg, 0.12 mmol) and aryl ester **1e** (34 mg, 0.1 mmol) in THF to afford **2s**. The crude reaction mixture was purified *via* preparative thin layer chromatography using hexane/ethyl acetate (95:05 v/v) as eluent. The desired compound **2s** was obtained as a yellow liquid. Yield: 24 mg, 0.08 mmol, 79%.

^{1}H NMR (500 MHz, $CDCl_3$, 298 K) δ: 7.41–7.38 (m, 2H, Ar–CH), 7.35–7.32 (m, 2H, Ar–CH), 7.31–7.2 8(m, 2H, Ar–CH), 7.27–7.22 (m, 7H, Ar–CH), 7.21–7.18 (m, 1H, Ar–CH), 7.18–7.15 (m, 1H, Ar–CH), 5.10 (s, 1H, CH);^{13}C NMR (126 MHz, $CDCl_3$, 298 K) δ: 141.4, 140.4, 132.9, 131.8, 129.4, 128.9, 128.4, 128.3, 128.0, 127.3, 125.9, 123.4, 89.7 (C≡C), 85.4 (C≡C), 43.3 (CH); IR ν_{max} (cm^{-1}): 3023, 2965, 2181 (C≡C), 1665, 1530, 1456, 1333, 1254, 1175, 1142, 1033; HRMS (EI+) $[M]^+$ $[C_{21}H_{15}Cl]^+$: calculated 302.0862, found 302.0860.

Synthesis of 1-chloro-4-(3-(4-methoxyphenyl)-1-phenylprop-2-yn-1-yl)benzene (**2t**)

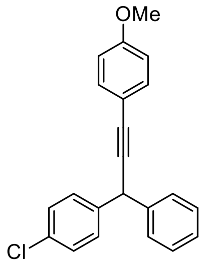

Synthesized in accordance with *General Procedure b* using $B(C_6F_5)_3$ (51 mg, 0.1 mmol), Mes_3P (39 mg, 0.1 mmol) 1-ethynyl-4-methoxybenzene (16 mg, 0.12 mmol) and aryl ester **1e** (34 mg, 0.1 mmol) in THF to afford **2t**. The crude reaction mixture was purified *via* preparative thin layer chromatography using hexane/ethyl acetate (95:05 v/v) as eluent. The desired compound **2t** was obtained as a yellow liquid. Yield: 23 mg, 0.07 mmol, 69%.

^{1}H NMR (400 MHz, $CDCl_3$, 298 K) δ: 7.42–7.38 (m, 4H, Ar–CH), 7.36–7.31 (m, 4H, Ar–CH), 7.30–7.27 (m, 2H, Ar–CH), 7.25–7.24 (m, 1H, Ar–CH), 6.86– 6.83 (m, 2H, Ar–CH), 5.16 (s, 1H, CH), 3.81 (s, 3H, OCH_3);^{13}C NMR (101 MHz, $CDCl_3$, 298 K) δ: 159.6, 141.6, 140.7, 133.2, 132.8, 129.4, 128.8, 128.0, 127.2, 115.5, 114.0, 88.2 (C≡C), 85.2 (C≡C), 55.4 (OCH_3), 43.3 (CH); IR ν_{max} (cm^{-1}): 3030, 2947, 2182 (C≡C), 1644, 1556, 1430, 1341, 1277, 1195, 1114, 1025; HRMS (EI+) $[M]^+$ $[C_{22}H_{17}ClO]^+$: calculated 332.0968, found 332.0972.

Synthesis of 1-fluoro-4-(3-phenyl-3-(p-tolyl)prop-1-yn-1-yl)benzene (**2u**)

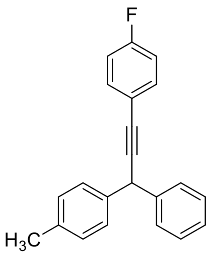

Synthesized in accordance with *General Procedure b* using $B(C_6F_5)_3$ (51 mg, 0.1 mmol), Mes_3P (39 mg, 0.1 mmol) 1-ethynyl-4-fluorobenzene (15 mg, 0.12 mmol) and aryl ester **1f** (32 mg, 0.1 mmol) in THF to afford **2u**. The crude reaction mixture was purified *via* preparative thin layer chromatography using hexane/ethyl acetate (95:05 v/v) as eluent. The desired compound **2u** was obtained as a pale-yellow liquid. Yield: 23 mg, 0.08 mmol, 76%.

^{1}H NMR (400 MHz, $CDCl_3$, 298 K) δ: 7.46–7.41 (m, 4H, Ar–CH), 7.32 (dd, J = 8.4, 7.3 Hz, 4H, Ar–CH), 7.25–7.21 (m, 1H, Ar–CH), 7.13 (d, J = 7.8 Hz, 2H, Ar–CH), 7.02–6.97 (m, 2H, Ar–CH), 5.16 (s, 1H, CH), 2.32 (s, 3H, CH_3);^{13}C NMR (101 MHz, $CDCl_3$, 298 K) δ: 161.2 (d, $J_{C–F}$ = 249.1 Hz) 142.0, 138.9, 136.7, 133.6 (d, $J_{C–F}$ = 8.3 Hz), 129.5, 128.8, 127.9 (d, $J_{C–F}$ = 7.8 Hz), 127.0, 115.6 (d, $J_{C–F}$ = 22.0 Hz), 90.2 (C≡C), 83.7 (C≡C), 43.4 (CH), 21.2 (CH_3); ^{19}F NMR (376 MHz, $CDCl_3$, 298 K) δ: -111.67 (Ar–F); IR ν_{max} (cm^{-1}): 3021, 2902, 2172 (C≡C), 1687, 1532, 1421, 1333, 1298, 1221, 1177, 1121, 1030; HRMS (ES+) $[M+H]^+$ $[C_{22}H_{18}F]^+$: calculated 301.1393, found 301.1397.

Synthesis of 3-(p-tolyl)prop-1-yne-1,3-diyl dibenzene (**2v**)[11]

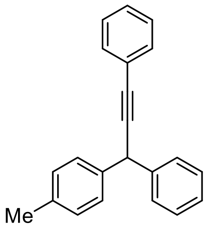

Synthesized in accordance with *General Procedure b* using $B(C_6F_5)_3$ (51 mg, 0.1 mmol), Mes_3P (39 mg, 0.1 mmol) phenylacetylene (12 mg, 0.12 mmol) and aryl ester **1f** (32 mg, 0.1 mmol) in THF to afford **2v**. The crude reaction mixture was purified *via* preparative thin layer chromatography using hexane/ethyl acetate (95:05 v/v) as eluent. The desired compound **2v** was obtained as a yellow liquid. Yield: 23 mg, 0.08 mmol, 80%.

^{1}H NMR (500 MHz, $CDCl_3$, 298 K) δ: 7.49–7.47 (m, 2H, Ar–CH), 7.45–7.43 (m, 2H, Ar–CH), 7.34–7.29 (m, 7H, Ar–CH), 7.25–7.21 (m, 1H, Ar–CH), 7.14–7.13 (m, 2H, Ar–CH), 5.18 (s, 1H, CH), 2.33 (s, 3H, CH_3);^{13}C NMR (101 MHz, $CDCl_3$, 298 K) δ: 142.1, 139.0, 136.6, 131.8, 129.4, 128.7, 128.3, 128.1, 128.0, 127.9, 127.0, 123.7, 90.5 (C≡C), 84.8 (C≡C), 43.5 (CH); IR ν_{max} (cm^{-1}): 3001, 2932, 2173 (C≡C), 1656, 1534, 1471, 1341, 1287, 1235, 1165, 1033; HRMS (EI+) $[M]^+$ $[C_{22}H_{18}]^+$: calculated 282.1409, found 282.1404.

Synthesis of 1-methoxy-4-(3-phenyl-3-(p-tolyl)prop-1-yn-1-yl)benzene (**2w**)

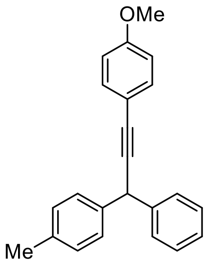

Synthesized in accordance with *General Procedure b* using $B(C_6F_5)_3$ (51 mg, 0.1 mmol), Mes_3P (39 mg, 0.1 mmol) 1-ethynyl-4-methoxybenzene (16 mg, 0.12 mmol) and aryl ester **1f** (32 mg, 0.1 mmol) in THF to afford **2w**. The crude reaction mixture was purified *via* preparative thin layer chromatography using hexane/ethyl acetate (97:03 v/v) as eluent. The desired compound **2w** was obtained as a white solid. Yield: 23 mg, 0.08 mmol, 80%.

^{1}H NMR (400 MHz, $CDCl_3$, 298 K) δ: 7.38–7.32 (m, 4H, Ar–CH), 7.27–7.22 (m, 4H, Ar–CH), 7.19–7.14 (m, 1H, Ar–CH), 7.07–7.04 (m, 2H, Ar–CH), 6.77–6.74 (m, 2H, Ar–CH), 5.09 (s, 1H, CH), 3.73 (s, 3H, OCH_3), 2.24 (s, 3H, CH_3);^{13}C NMR (101 MHz, $CDCl_3$, 298 K) δ: 159.5, 142.3, 139.2, 136.6, 133.2, 129.4, 128.7, 128.0, 127.9, 126.9, 115.9, 114.0, 89.0 (C≡C), 84.6 (C≡C), 55.4 (OCH_3), 43.5 (CH), 21.2 (CH_3); IR ν_{max} (cm^{-1}): 3001, 2912, 2177 (C≡C), 1588, 1465, 1333, 1256, 1201, 1134, 1030; HRMS (ES+) $[M+H]^+$ $[C_{23}H_{21}O]^+$: calculated 313.1592, found 313.1596.

Synthesis of 2-(4-phenylbut-3-yn-2-yl)naphthalene (**2x**)[15]

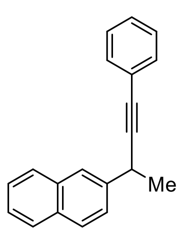

Synthesized in accordance with *General Procedure b* using $B(C_6F_5)_3$ (51 mg, 0.1 mmol), Mes_3P (39 mg, 0.1 mmol) phenylacetylene (12 mg, 0.12 mmol) and aryl ester **1g** (30 mg, 0.1 mmol) in THF to afford **2x**. The crude reaction mixture was purified *via* preparative thin layer chromatography using hexane/ethyl acetate (95:05 v/v) as eluent. The desired compound **2x** was obtained as a colorless liquid. Yield: 16 mg, 0.06 mmol, 61%.

^{1}H NMR (400 MHz, $CDCl_3$, 298 K) δ: 7.89 (dd, *J* = 1.7, 0.8 Hz, 1H, Ar–CH), 7.85–7.81 (m, 3H, Ar–CH), 7.58 (dd, *J* = 8.1 Hz, 4.2 Hz, 1H), 7.49–7.43 (m, 4H, Ar–CH), 7.32–7.29 (m, 3H, Ar–CH), 4.16 (q, *J* = 7.2 Hz, 1H), 1.67 (d, *J* = 7.1 Hz, 3H); ^{13}C NMR (126 MHz, $CDCl_3$, 298 K) δ: 140.8, 133.7, 132.6, 131.8, 128.4, 128.4, 128.1, 127.9, 127.9, 127.8, 127.7, 126.2, 125.7, 125.7, 125.3, 123.9, 92.7 (C≡C), 82.8 (C≡C), 32.8 (CH), 24.5 (CH_3); IR ν_{max} (cm^{-1}): 3033, 2914, 2177 (C≡C), 1654, 1566, 1432, 1357, 1269, 1278, 1145, 1101, 1033; HRMS (ES+) $[M+H]^+$ $[C_{21}H_{19}O]^+$: calculated 287.1436, found 287.1439.

Synthesis of 2-(4-(4-methoxyphenyl)but-3-yn-2-yl)naphthalene (**2y**)

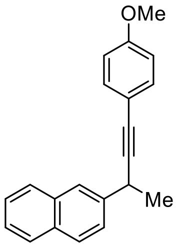

Synthesized in accordance with *General Procedure b* using $B(C_6F_5)_3$ (51 mg, 0.1 mmol), Mes_3P (39 mg, 0.1 mmol) 1-ethynyl-4-methoxybenzene (16 mg, 0.12 mmol) and aryl ester **1g** (30 mg, 0.1 mmol) in THF to afford **2y**. The crude reaction mixture was purified *via* preparative thin layer chromatography using hexane/ethyl acetate (95:05 v/v) as eluent. The desired compound **2y** was obtained as a colorless liquid. Yield: 19 mg, 0.06 mmol, 65%.

^{1}H NMR (500 MHz, $CDCl_3$, 298 K) δ: 7.89 (s, 1H, Ar–CH), 7.85–7.82 (m, 2H, Ar–CH), 7.58 (dd, *J* = 8.6, 1.8 Hz, 1H, Ar–CH), 7.50–7.38 (m, 5H, Ar–CH), 6.85–6.83 (m, 2H, Ar–CH), 4.14 (q, *J* = 7.1 Hz, 1H, CH), 3.81 (s, 1H, OCH_3), 1.66 (d, *J* = 7.1 Hz, 3H, CH_3); ^{13}C NMR (126 MHz, $CDCl_3$, 298 K) δ: 159.4, 141.1, 133.7, 133.1, 132.5, 129.4, 128.8, 128.4, 128.0, 127.9, 127.7, 126.2, 125.8, 125.7, 125.2, 116.0, 114.0, 91.1 (C≡C), 82.5 (C≡C), 55.4 (OCH_3), 32.8, (CH), 24.6 (CH_3); IR ν_{max} (cm^{-1}): 3021, 2902, 2175 (C≡C), 1687, 1532, 1421, 1333, 1298, 1221, 1177, 1121, 1030; HRMS (ES+) $[M+H]^+$ $[C_{21}H_{19}O]^+$: calculated 287.1436, found 287.1439.

Synthesis of (E)-(5-(4-fluorophenyl)pent-1-en-4-yne-1,3-diyl)dibenzene (**2z**)[16]

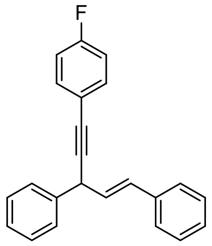

Synthesized in accordance with *General Procedure b* using $B(C_6F_5)_3$ (51 mg, 0.1 mmol), Mes_3P (39 mg, 0.1 mmol) 1-ethynyl-4-fluorobenzene (15 mg, 0.12 mmol) and aryl ester **1k** (31 mg, 0.1 mmol) in THF to afford **2z**. The crude reaction mixture was purified *via* preparative thin layer chromatography using hexane/ethyl acetate (95:05 v/v) as eluent. The desired compound **2z** was obtained as a colorless liquid. Yield: 27 mg, 0.09 mmol, 88%.

^{1}H NMR (500 MHz, $CDCl_3$, 298 K) δ: 7.49–7.45 (m, 4H, Ar–CH), 7.41–7.35 (m, 4H, Ar–CH), 7.32–7.27 (m, 3H, Ar–CH), 7.24–7.21 (m, 1H, Ar–CH), 7.03–6.99 (m, 2H, Ar–CH), 6.76 (dd, J = 15.7, 1.5 Hz, 1H, CH_2), 6.33 (dd, J = 15.7, 6.6 Hz, 1H, CH_2), 4.74 (d, J = 6.6 Hz, 1H, CH);^{13}C NMR (126 MHz, $CDCl_3$, 298 K) δ: 162.5 (d, $J_{C–F}$ = 248.9 Hz), 140.3, 136.9, 133.7 (d, $J_{C–F}$ = 8.3 Hz), 130.7, 129.6, 128.9, 128.7, 127.9, 127.7, 127.3, 126.7, 119.6 (d, $J_{C–F}$ = 3.5 Hz), 115.6 (d, $J_{C–F}$ = 22.0 Hz), 88.7 (C≡C), 84.4 (C≡C), 41.3 (CH); ^{19}F NMR (471 MHz, $CDCl_3$, 298 K) δ: -111.52 (Ar–F); IR ν_{max} (cm^{-1}): 2998, 2895, 2173 (C≡C), 1650 (C=C), 1521, 1487, 1378, 1305, 1277, 1205, 1165, 1031; HRMS (EI+) $[M]^+$ $[C_{23}H_{17}F]^+$: calculated 312.1314, found 312.1319.

Synthesis of (E)-(5-(4-chlorophenyl)pent-1-en-4-yne-1,3-diyl)dibenzene (**2aa**)

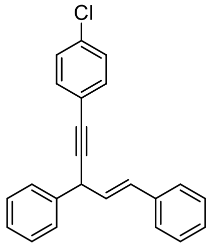

Synthesized in accordance with *General Procedure b* using $B(C_6F_5)_3$ (51 mg, 0.1 mmol), Mes_3P (39 mg, 0.1 mmol) 1-chloro-4-ethynylbenzene (16 mg, 0.12 mmol) and aryl ester **1k** (31 mg, 0.1 mmol) in THF to afford **2aa**. The crude reaction mixture was purified *via* preparative thin layer chromatography using hexane/ethyl acetate (95:05 v/v) as eluent. The desired compound **2aa** was obtained as a pale-yellow liquid. Yield: 28 mg, 0.08 mmol, 85%.

^{1}H NMR (500 MHz, $CDCl_3$, 298 K) δ: 7.49–7.46 (m, 2H, Ar–CH), 7.43–7.35 (m, 6H, Ar–CH), 7.32–7.27 (m, 5H, Ar–CH), 7.25–7.21 (m, 1H, Ar–CH), 6.75 (dd, J = 15.7, 1.5 Hz, 1H, CH_2), 6.33 (dd, J = 15.7, 6.6 Hz, 1H, CH_2), 4.74 (d, J = 5.2 Hz, 1H, CH);^{13}C NMR (126 MHz, $CDCl_3$, 298 K) δ: 140.2, 136.9, 134.2, 133.1, 130.7, 129.5, 128.9, 128.7, 128.7, 127.9, 127.8, 127.3, 126.7, 122.1, 90.1 (C≡C), 84.4 (C≡C), 41.4 (CH); IR ν_{max} (cm^{-1}): 3001, 2968, 2170 (C≡C), 1645 (C=C), 1578, 1467, 1357, 1341, 1265, 1212, 1137, 1030; HRMS (EI+) $[M]^+$ $[C_{23}H_{17}Cl]^+$: calculated 328.1019, found 328.1022.

Synthesis of (E)-(5-(4-bromophenyl)pent-1-en-4-yne-1,3-diyl)dibenzene (**2ab**)

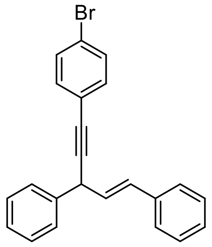

Synthesized in accordance with *General Procedure b* using $B(C_6F_5)_3$ (51 mg, 0.1 mmol), Mes_3P (39 mg, 0.1 mmol) 1-bromo-4-ethynylbenzene (22 mg, 0.12 mmol) and aryl ester **1k** (31 mg, 0.1 mmol) in THF to afford **2ab**. The crude reaction mixture was purified *via* preparative thin layer chromatography using hexane/ethyl acetate (95:05 v/v) as eluent. The desired compound **2ab** was obtained as a pale-yellow liquid. Yield: 30 mg, 0.08 mmol, 81%.

^1H NMR (500 MHz, $CDCl_3$, 298 K) δ: 7.48–7.44 (m, 4H, Ar–CH), 7.40–7.34 (m, 6H, Ar–CH), 7.32–7.27 (m, 3H, Ar–CH), 7.25–7.21 (m, 1H, Ar–CH), 6.74 (dd, *J* = 15.7, 1.5 Hz, 1H, CH_2), 6.33 (dd, *J* = 15.6, 6.6 Hz, 1H, CH_2), 4.73 (d, *J* = 5.4 Hz, 1H, CH);^{13}C NMR (126 MHz, $CDCl_3$, 298 K) δ: 140.2, 136.9, 133.3, 131.6, 130.8, 129.4, 128.9, 128.7, 127.9, 127.8, 127.3, 126.7, 122.5, 122.3, 90.3 (C≡C), 84.5 (C≡C), 41.4 (CH); IR ν_{max} (cm^{-1}): 3011, 2981, 2171 (C≡C), 1655 (C=C), 1535, 1418, 1377, 1332, 1259, 1249, 1141, 1033; HRMS (EI+) $[M]^+$ $[C_{23}H_{17}Br]^+$: calculated 372.0514, found 372.0519.

Synthesis of (E)-pent-1-en-4-yne-1,3,5-triyltribenzene (**2ac**)[16]

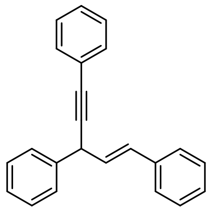

Synthesized in accordance with *General Procedure b* using $B(C_6F_5)_3$ (51 mg, 0.1 mmol), Mes_3P (39 mg, 0.1 mmol) phenylacetylene (22 mg, 0.12 mmol) and aryl ester **1k** (31 mg, 0.1 mmol) in THF to afford **2ac**. The crude reaction mixture was purified *via* preparative thin layer chromatography using hexane/ethyl acetate (95:05 v/v) as eluent. The desired compound **2ac** was obtained as a colorless liquid. Yield: 24 mg, 0.08 mmol, 83%.

^1H NMR (500 MHz, $CDCl_3$, 298 K) δ: 7.51–7.49 (m, 4H, Ar–CH), 7.41–7.36 (m, 4H, Ar–CH), 7.33–7.27 (m, 6H, Ar–CH), 7.24–7.21 (m, 1H, Ar–CH), 6.78 (dd, *J* = 15.7, 1.5 Hz, 1H, CH_2), 6.35 (dd, *J* = 15.6, 6.6 Hz, 1H, CH_2), 4.76 (d, *J* = 6.6 Hz, 1H, CH_2);^{13}C NMR (126 MHz, $CDCl_3$, 298 K) δ: 140.4, 137.0, 131.8, 130.6, 129.8, 128.9, 128.7, 128.4, 128.2, 127.9, 127.7, 127.2, 126.7, 123.6, 89.0 (C≡C), 85.5 (C≡C), 41.4 (CH); IR ν_{max} (cm^{-1}): 3023, 2974, 2171 (C≡C), 1652 (C=C), 1532, 1425, 1364, 1314, 1251, 1222, 1140, 1028; HRMS (EI+) $[M]^+$ $[C_{23}H_{18}]^+$: calculated 294.1409, found 294.1412.

Synthesis of (E)-(5-(4-(tert-butyl)phenyl)pent-1-en-4-yne-1,3-diyl)dibenzene (**2ad**)

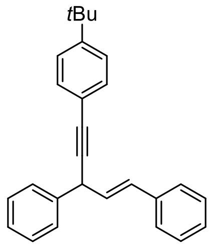

Synthesized in accordance with *General Procedure b* using $B(C_6F_5)_3$ (51 mg, 0.1 mmol), Mes_3P (39 mg, 0.1 mmol) 1-(*tert*-butyl)-4-ethynylbenzene (19 mg, 0.12 mmol) and aryl ester **1k** (31 mg, 0.1 mmol) in THF to afford **2ad**. The crude reaction mixture was purified *via* preparative thin layer chromatography using hexane/ethyl acetate (95:05 v/v) as eluent. The desired compound **2ad** was obtained as a white solid. Yield: 27 mg, 0.08 mmol, 78%.

^{1}H NMR (500 MHz, $CDCl_3$, 298 K) δ: 7.52–7.50 (m, 2H, Ar–CH), 7.46–7.44 (m, 2H, Ar–CH), 7.42–7.39 (m, 2H, Ar–CH), 7.37–7.34 (m, 4H, Ar–CH), 7.33–7.29 (m, 3H, Ar–CH), 7.25–7.22 (m, 1H, Ar–CH), 6.79 (d, J = 15.6 Hz, 1H, CH_2), 6.35 (dd, J = 15.6, 6.5 Hz, 1H, CH_2), 4.76 (d, J = 6.5 Hz, 1H, CH), 1.33 (s, 9H, CH_3);^{13}C NMR (126 MHz, $CDCl_3$, 298 K) δ: 151.4, 140.6, 137.0, 131.5, 130.5, 123.0, 128.8, 128.7, 127.9, 127.6, 127.2, 126.7, 125.4, 120.6, 88.2 (C≡C), 85.6 (C≡C), 41.4 (CH), 34.9 (quaternary C), 31.3 (CH_3); IR ν_{max} (cm^{-1}): 3023, 2984, 2177 (C≡C), 1648 (C=C), 1498, 1461, 1351, 1314, 1237, 1202, 1177, 1028; HRMS (ES+) $[M+H]^+$ $[C_{27}H_{27}]^+$: calculated 351.2113, found 351.2111.

Synthesis of (E)-4-(3,5-diphenylpent-4-en-1-yn-1-yl)-1,1'-biphenyl (**2ae**)

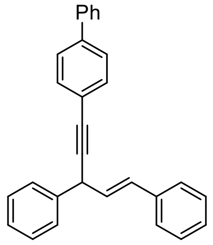

Synthesized in accordance with *General Procedure b* using $B(C_6F_5)_3$ (51 mg, 0.1 mmol), Mes_3P (39 mg, 0.1 mmol) 4-ethynyl-1,1'-biphenyl (21 mg, 0.12 mmol) and aryl ester **1k** (31 mg, 0.1 mmol) in THF to afford **2ae**. The crude reaction mixture was purified *via* preparative thin layer chromatography using hexane/ethyl acetate (95:05 v/v) as eluent. The desired compound **2ae** was obtained as a colorless liquid. Yield: 30 mg, 0.08 mmol, 82%.

^{1}H NMR (500 MHz, $CDCl_3$, 298 K) δ: 7.62–7.60 (m, 2H, Ar–CH), 7.58 (d, J = 0.7 Hz, 3H, Ar–CH), 7.54–7.52 (m, 2H, Ar–CH), 7.48–7.35 (m, 6H, Ar–CH), 7.34– 7.27 (m, 4H, Ar–CH), 7.25– 7.10 (m, 2H, Ar–CH), 6.81 (dd, J = 15.7, 1.5 Hz, 1H, CH_2), 6.37 (dd, J = 15.6, 6.6 Hz, 1H, CH_2), 4.79 (dd, J = 6.6, 1.4 Hz, 1H, CH);^{13}C NMR (126 MHz, $CDCl_3$, 298 K) δ: 140.9, 140.6, 140.4, 137.0, 132.3, 130.6, 129.8, 129.0, 128.8, 128.7, 127.9, 127.7, 127.6, 127.3, 127.2, 127.1, 126.7, 122.5, 89.7 (C≡C), 85.4 (C≡C), 41.5 (CH); IR ν_{max} (cm^{-1}): 3009, 2974, 2174 (C≡C), 1647 (C=C), 1503, 1477, 1337, 1323, 1237, 1242, 1156, 1038; HRMS (ES+) $[M+H]^+$ $[C_{29}H_{23}]^+$: calculated 371.1800, found 371.1803.

Synthesis of (E)-9-(3,5-diphenylpent-4-en-1-yn-1-yl)phenanthrene (**2af**)

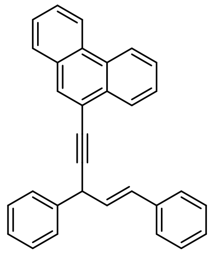

Synthesized in accordance with *General Procedure b* using $B(C_6F_5)_3$ (51 mg, 0.1 mmol), Mes_3P (39 mg, 0.1 mmol) 9-ethynylphenanthrene (24 mg, 0.12 mmol) and aryl ester **1k** (31 mg, 0.1 mmol) in THF to afford **2af**. The crude reaction mixture was purified *via* preparative thin layer chromatography using hexane/ethyl acetate (95:05 v/v) as eluent. The desired compound **2af** was obtained as a white solid. Yield: 35 mg, 0.09 mmol, 89%.

^{1}H NMR (500 MHz, $CDCl_3$, 298 K) δ: 8.58–8.52 (m, 2H, Ar–CH), 8.40–8.39 (m, 1H, Ar–CH), 7.94 (s, 1H, Ar–CH), 7.73 (dt, *J* = 8.1, 3.8 Hz, 1H, Ar–CH), 7.58–7.46 (m, 6H, Ar–CH), 7.34–7.30 (m, 4H, Ar–CH), 7.23–7.20 (m, 3H, Ar–CH), 7.15–7.11 (m, 1H, Ar–CH), 6.81 (dd, *J* = 15.7, 1.4 Hz, 1H, CH_2), 6.36 (dd, *J* = 15.6, 6.5 Hz, 1H, CH_2), 4.85 (dd, *J* = 6.6, 1.5 Hz, 1H, CH);^{13}C NMR (126 MHz, $CDCl_3$, 298 K) δ: 140.5, 137.0, 131.9, 131.5, 131.4, 130.8, 130.3, 130.2, 129.8, 129.0, 128.7, 128.6, 128.0, 127.7, 127.4, 127.3 127.2, 127.1, 127.0, 126.72, 122.9, 122.7, 119.9, 93.6 (C≡C) 83.8 (C≡C), 41.8 (CH); IR ν_{max} (cm^{-1}): 3015, 2987, 2178 (C≡C), 1644 (C=C), 1533, 1481, 1351, 1333, 1245, 1230, 1151, 1033; HRMS (ES+) $[M+H]^+$ $[C_{31}H_{23}]^+$: calculated 395.1800, found 395.1805.

Synthesis of (E)-(5-(4-methoxyphenyl)pent-1-en-4-yne-1,3-diyl)dibenzene (**2ag**)[16]

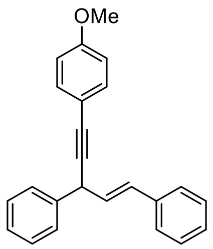

Synthesized in accordance with *General Procedure b* using $B(C_6F_5)_3$ (51 mg, 0.1 mmol), Mes_3P (39 mg, 0.1 mmol) 1-ethynyl-4-methoxybenzene (16 mg, 0.12 mmol) and aryl ester **1k** (31 mg, 0.1 mmol) in THF to afford **2ag**. The crude reaction mixture was purified *via* preparative thin layer chromatography using hexane/ethyl acetate (92:08 v/v) as eluent. The desired compound **2ag** was obtained as a colorless liquid. Yield: 23 mg, 72%, 0.07 mmol.

^{1}H NMR (500 MHz, $CDCl_3$, 298 K) δ: 7.52–7.50 (m, 2H, Ar–CH), 7.46–7.44 (m, 2H, Ar–CH), 7.42–7.37 (m, 4H, Ar–CH), 7.33–7.29 (m, 3H, Ar–CH), 7.25–7.22 (m, 1H, Ar–CH), 6.88–6.85 (m, 2H, Ar–CH), 6.79 (dd, *J* = 15.6, 1.5 Hz, 1H, CH_2), 6.36 (dd, *J* = 15.6, 6.6 Hz, 1H, CH_2), 4.76 (d, *J* = 6.2 Hz, 1H, CH), 3.82 (s, 3H, OCH_3);^{13}C NMR (126 MHz, $CDCl_3$, 298 K) δ: 159.5, 140.6, 137.0, 133.2, 130.4, 130.0, 128.8, 128.6, 127.9, 127.6, 127.2, 126.6, 115.7, 114.0, 87.4 (C≡C), 85.3 (C≡C), 55.4 (OCH_3), 41.4 (CH); IR ν_{max} (cm^{-1}): 3009, 2978, 2171 (C≡C), 1655 (C=C), 1545, 1471, 1332, 1303, 1211, 1137, 1031; HRMS (ES+) $[M+H]^+$ $[C_{24}H_{21}O]^+$: calculated 325.1592, found 325.1595.

Synthesis of (E)-(5-(p-tolyl)pent-1-en-4-yne-1,3-diyl)dibenzene (**2ah**)[16]

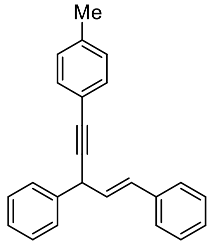

Synthesized in accordance with *General Procedure b* using $B(C_6F_5)_3$ (51 mg, 0.1 mmol), Mes_3P (39 mg, 0.1 mmol) 1-ethynyl-4-methylbenzene (14 mg, 0.12 mmol) and aryl ester **1k** (31 mg, 0.1 mmol) in THF to afford **2ah**. The crude reaction mixture was purified *via* preparative thin layer chromatography using hexane/ethyl acetate (95:05 v/v) as eluent. The desired compound **2ah** was obtained as a colorless liquid. Yield: 26 mg, 0.08 mmol, 84%.

^{1}H NMR (500 MHz, $CDCl_3$, 298 K) δ: 7.52–7.49 (m, 2H, Ar–CH), 7.41–7.36 (m, 6H, Ar–CH), 7.33–7.28 (m, 3H, Ar–CH), 7.25–7.21 (m, 1H, Ar–CH), 7.13 (dt, *J* = 7.8, 0.7 Hz, 2H, Ar–CH), 6.78 (dd, *J* = 15.7, 1.5 Hz, 1H, CH_2), 6.35 (dd, *J* = 15.7, 6.6 Hz, 1H, CH_2), 4.75 (d, *J* = 6.0 Hz, 1H, CH), 2.36 (s, 3H, CH_3);^{13}C NMR (126 MHz, $CDCl_3$, 298 K) δ: 140.6, 138.2, 137.0, 131.7, 130.5, 129.9, 129.1, 128.8, 128.7, 127.9, 127.6, 127.2, 126.7, 120.5, 88.2 (C≡C), 85.6 (C≡C), 41.4 (CH), 21.6 (CH_3); IR ν_{max} (cm^{-1}): 3010, 2965, 2175 (C≡C), 1648 (C=C), 1563, 1474, 1322, 1347, 1257, 1232, 1165, 1030; HRMS (ES+) $[M+H]^+$ $[C_{24}H_{21}]^+$: calculated 309.1643, found 309.1647.

Synthesis of 4,4'-(3-(4-vinylphenyl)prop-2-yne-1,1-diyl)bis(fluorobenzene) (**2ai**)

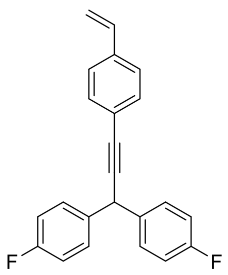

Synthesized in accordance with *General Procedure b* using $B(C_6F_5)_3$ (51 mg, 0.1 mmol), Mes_3P (39 mg, 0.1 mmol), 1-ethynyl-4-vinylbenzene (**4a**) (15 mg, 0.12 mmol) and aryl ester **1a** (34 mg, 0.1 mmol) in THF to afford **2ai**. The crude reaction mixture was purified *via* preparative thin layer chromatography using hexane/ethyl acetate (98:02 v/v) as eluent. The desired compound **2ai** was obtained as a yellow liquid. Yield: 23 mg, 0.07 mmol, 70%.

^{1}H NMR (500 MHz, $CDCl_3$, 298 K) δ: 7.43–7.41 (m, 2H, Ar–CH), 7.38–7.34 (m, 6H, Ar–CH), 7.04–6.99 (m, 4H, Ar–CH), 6.69 (dd, *J* = 17.6, 10.9 Hz, 1H, CH_2), 5.76 (dd, *J* = 17.6, 0.8 Hz, 1H, CH_2), 5.29 (dd, *J* = 10.9, 0.7 Hz, 1H, CH_2), 5.18 (s, 1H, CH); ^{13}C NMR (126 MHz, $CDCl_3$, 298 K) δ: 162.0 (d, $J_{C–F}$ = 245.6 Hz), 137.6, 137.4 (d, $J_{C–F}$ = 3.1 Hz), 136.3, 132.0, 129.5 (d, $J_{C–F}$ = 8.0 Hz), 126.2, 122.5, 115.7 (d, $J_{C–F}$ = 21.5 Hz), 114.9, 90.4 (C≡C), 85.4 (C≡C), 42.5 (CH); ^{19}F NMR (471 MHz, $CDCl_3$, 298 K) δ: -115.77 (Ar–F); IR ν_{max} (cm^{-1}): 3066, 2998, 2180 (C≡C), 1602 (C=C), 1506, 1375, 1276, 1224, 1157; HRMS: (ASAP+) [M+] $[C_{23}H_{16}F_2]^+$: calculated 330.1220, found 330.1228.

Synthesis of (3-(4-vinylphenyl)prop-2-yne-1,1-diyl)dibenzene (**2aj**)

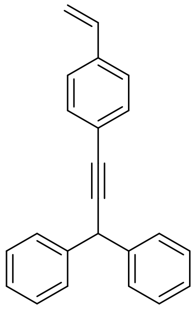

Synthesized in accordance with *General Procedure b* using $B(C_6F_5)_3$ (51 mg, 0.1 mmol), Mes_3P (39 mg, 0.1 mmol), 1-ethynyl-4-vinylbenzene (**4a**) (15 mg, 0.12 mmol) and aryl ester **1c** (31 mg, 0.1 mmol) in THF to afford **2aj**. The crude reaction mixture was purified *via* preparative thin layer chromatography using hexane/ethyl acetate (97:03 v/v) as eluent. The desired compound **2aj** was obtained as a colorless liquid. Yield: 21 mg, 0.07 mmol, 71%.

^{1}H NMR (500 MHz, $CDCl_3$, 298 K) δ: 7.45–7.43 (m, 6H, Ar–CH), 7.36–7.31 (m, 6H, Ar–CH), 7.26–7.23 (m, 2H, Ar–CH), 6.69 (dd, J = 17.6, 10.9 Hz, 1H, CH_2), 5.76 (dd, J = 17.6, 0.8 Hz, 1H, CH_2), 5.28 (dd, J = 10.9, 0.8 Hz, 1H, CH_2), 5.22 (s, 1H, CH); ^{13}C NMR (126 MHz, $CDCl_3$, 298 K) δ: 141.9, 137.3, 136.4, 132.0, 128.8, 128.1, 127.1, 126.2, 122.1, 114.7, 91.0 (C≡C), 85.0 (C≡C), 44.0 (CH); IR ν_{max} (cm^{-1}): 3028, 2928, 2120 (C≡C), 1600 (C=C), 1506, 1492, 1276, 1111, 1029; HRMS: (ASAP+) [M]$^+$ $[C_{23}H_{18}]^+$: calculated 294.1409, found 294.1406.

Synthesis of 1-chloro-4-(1-phenyl-3-(4-vinylphenyl)prop-2-yn-1-yl)benzene (**2ak**)

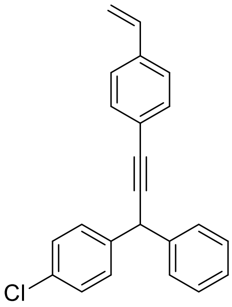

Synthesized in accordance with *General Procedure b* using $B(C_6F_5)_3$ (51 mg, 0.1 mmol), Mes_3P (39 mg, 0.1 mmol), 1-ethynyl-4-vinylbenzene (**4a**) (15 mg, 0.12 mmol) and aryl ester **1e** (34 mg, 0.1 mmol) in THF to afford **2ak**. The crude reaction mixture was purified *via* preparative thin layer chromatography using hexane/ethyl acetate (97:03 v/v) as eluent. The desired compound **2ak** was obtained as a colorless liquid. Yield: 25 mg, 0.07 mmol, 76%.

^{1}H NMR (500 MHz, $CDCl_3$, 298 K) δ: 7.37–7.32 (m, 4H, Ar–CH), 7.31–7.24 (m, 6H, Ar–CH), 7.24–7.19 (m, 3H, Ar–CH), 6.62 (dd, J = 16.8, 10.5 Hz, 1H, CH_2), 5.69 (dd, J = 17.6, 0.8 Hz, 1H, CH_2), 5.21 (dd, J = 10.9, 0.8 Hz, 1H, CH_2), 5.12 (s, 1H, CH); ^{13}C NMR (126 MHz, $CDCl_3$, 298 K) δ: 141.3, 140.4, 137.5, 136.4, 132.9, 131.9, 129.4, 128.9, 127.9, 127.2, 126.2, 122.6, 114.8, 90.3(C≡C), 85.3(C≡C), 43.3(CH); IR ν_{max} (cm^{-1}): 3028, 2924, 2116 (C≡C), 1654, 1600 (C=C), 1489, 1450, 1402, 1271, 1176, 1014; HRMS: (ES+) [M+H] $[C_{23}H_{18}Cl]$: calculated 329.1097, found 329.1084.

Synthesis of 4,4'-(3-(2-vinylphenyl)prop-2-yne-1,1-diyl)bis(fluorobenzene) (**2al**)

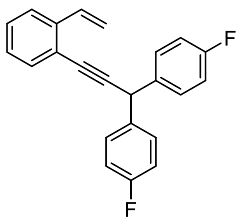

Synthesized in accordance with *General Procedure b* using $B(C_6F_5)_3$ (51 mg, 0.1 mmol), Mes_3P (39 mg, 0.1 mmol), 1-ethynyl-2-vinylbenzene (**4d**) (15 mg, 0.12 mmol) and aryl ester **1a** (34 mg, 0.1 mmol) in TFT to afford **2al**. The crude reaction mixture was purified *via* preparative thin layer chromatography using hexane/ethyl acetate (97:03 v/v) as eluent. The desired compound **2al** was obtained as a yellow liquid. Yield: 20 mg, 0.06 mmol, 61%.

^{1}H NMR (500 MHz, $CDCl_3$, 298 K) δ: 7.58–7.57 (m, 1H, Ar–CH), 7.47–7.45 (m, 1H, Ar–CH), 7.40–7.37 (m, 4H, Ar–CH), 7.30–7.29 (m, 1H, Ar–CH), 7.21 (td, J = 7.5, 1.3 Hz, 1H, Ar–CH), 7.16 (dd, J = 17.6, 11.0 Hz, 1H, Ar–CH), 7.04–7.01 (m, 4H, Ar–CH), 5.79 (dd, J = 17.6, 1.1 Hz, 1H, CH_2), 5.31 (dd, J = 11.0, 1.1 Hz, 1H, CH_2), 5.23 (s, 1H, CH); ^{13}C NMR (126 MHz, $CDCl_3$, 298 K) δ: 162.0 (d, $J_{C–F}$ = 245.7 Hz), 139.3, 137.4 (d, $J_{C–F}$ = 3.3 Hz), 135.0, 132.7, 129.5 (d, $J_{C–F}$ = 8.1 Hz), 128.5, 127.6, 124.8, 121.9, 115.8, 115.6 (d, $J_{C–F}$ = 21.7 Hz), 94.4 (C≡C), 83.9 (C≡C), 42.6 (CH); ^{19}F NMR (471 MHz, $CDCl_3$, 298 K) δ: -115.75 (Ar–F); IR ν_{max} (cm^{-1}): 2958, 2928, 2177 (C≡C), 1654, 1620 (C=C), 1506, 1297, 1261, 1230, 1157, 1096, 1015; HRMS (GC-MS) [M+] $[C_{23}H_{16}F_2]^+$: calculated 330.1220, found 330.1219.

Synthesis of 4,4'-(3-(2-vinylphenyl)prop-2-yne-1,1-diyl)bis(chlorobenzene) (**2am**)

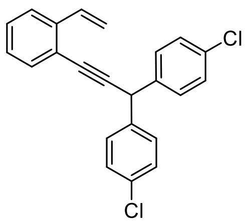

Synthesized in accordance with *General Procedure b* using $B(C_6F_5)_3$ (51 mg, 0.1 mmol), Mes_3P (39 mg, 0.1 mmol), 1-ethynyl-2-vinylbenzene (**4d**) (15 mg, 0.12 mmol) and aryl ester **1b** (37 mg, 0.1 mmol) in TFT to afford **2am**. The crude reaction mixture was purified *via* preparative thin layer chromatography using hexane/ethyl acetate (98:02 v/v) as eluent. The desired compound **2am** was obtained as a yellow liquid. Yield: 23 mg, 0.06 mmol, 63%.

^{1}H NMR (500 MHz, $CDCl_3$, 298 K) δ: 7.58–7.57 (m, 1H, Ar–CH), 7.47–7.45 (m, 1H, Ar–CH), 7.40–7.37 (m, 4H, Ar–CH), 7.30–7.29 (m, 1H, Ar–CH), 7.21 (td, J = 7.5, 1.3 Hz, 1H, Ar–CH), 7.16 (dd, J = 17.6, 11.0 Hz, 1H, Ar–CH), 7.04–7.01 (m, 4H, Ar–CH), 5.79 (dd, J = 17.6, 1.1 Hz, 1H, CH_2), 5.31 (dd, J = 11.0, 1.1 Hz, 1H, CH_2), 5.23 (s, 1H, CH); ^{13}C NMR (126 MHz, $CDCl_3$, 298 K) δ: 162.0 (d, $J_{C–F}$ = 245.7 Hz), 139.3, 137.4 (d, $J_{C–F}$ = 3.3 Hz), 135.0, 132.7, 129.5 (d, $J_{C–F}$ = 8.1 Hz), 128.5, 127.6, 124.8, 121.9, 115.8, 115.6 (d, $J_{C–F}$ = 21.7 Hz), 94.4 (C≡C), 83.9 (C≡C), 42.6 (CH); ^{19}F NMR (471 MHz, $CDCl_3$, 298 K) δ: -115.75 (Ar–F); IR ν_{max} (cm^{-1}): 2958, 2928, 2181 (C≡C), 1654, 1620 (C=C), 1506, 1297, 1261, 1230, 1157, 1096, 1015; HMRS (EI-) [M-Cl]$^-$ $[C_{23}H_{16}Cl]^-$: calculated 327.0935, found 327.0936.

Synthesis of (3-(2-vinylphenyl)prop-2-yne-1,1-diyl)dibenzene (**2an**)

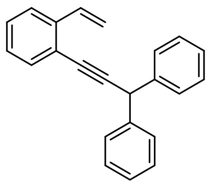

Synthesized in accordance with *General Procedure b* using $B(C_6F_5)_3$ (51 mg, 0.1 mmol), Mes_3P (39 mg, 0.1 mmol), 1-ethynyl-2-vinylbenzene (**4d**) (15 mg, 0.12 mmol) and aryl ester **1c** (31 mg, 0.1 mmol) in TFT to afford **2an**. The crude reaction mixture was purified *via* preparative thin layer chromatography using hexane/ethyl acetate (97:03 v/v) as eluent. The desired compound **2an** was obtained as a colorless liquid. Yield: 17 mg, 0.06 mmol, 58%.

^{1}H NMR (500 MHz, $CDCl_3$, 298 K) δ: 7.58–7.56 (m, 1H, Ar–CH), 7.48–7.44 (m, 5H, Ar–CH), 7.35–7.31 (m, 4H, Ar–CH), 7.30–7.28 (m, 1H, Ar–CH), 7.25–7.18 (m, 4H, Ar–CH), 5.79 (dd, *J* = 17.6, 1.1 Hz, 1H, CH_2), 5.31 (dd, *J* = 11.0, 1.1 Hz, 1H, CH_2), 5.27 (s, 1H, CH); ^{13}C NMR (126 MHz, $CDCl_3$, 298 K) δ: 141.8, 139.3, 135.2, 132.7, 128.7, 128.3, 128.0, 127.5, 127.0, 124.6, 122.3, 115.5, 95.0 (C≡C), 83.4 (C≡C), 44.1 (CH); IR ν_{max} (cm^{-1}): 3086, 3028, 2175 (C≡C), 1625 (C=C), 1597, 1558, 1541, 1519, 1492, 1477, 1452, 1076, 1029; HRMS (ASAP+) $[M+H]^+$ $[C_{23}H_{19}]^+$: calculated 295.1487, found 295.1495.

Synthesis of 4,4'-(3-(2-vinylphenyl)prop-2-yne-1,1-diyl)bis(methylbenzene)(**2ao**)

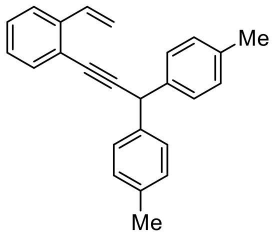

Synthesized in accordance with *General Procedure b* using $B(C_6F_5)_3$ (51 mg, 0.1 mmol), Mes_3P (39 mg, 0.1 mmol), 1-ethynyl-2-vinylbenzene (**4d**) (15 mg, 0.12 mmol), and aryl ester **1i** (33 mg, 0.1 mmol) in TFT to afford **2ao**. The crude reaction mixture was purified *via* preparative thin layer chromatography using hexane/ethyl acetate (98:02 v/v) as eluent. The desired compound **2ao** was obtained as a colorless liquid. Yield: 19 mg, 0.06 mmol, 58%.

^{1}H NMR (500 MHz, $CDCl_3$, 298 K) δ: 7.58–7.56 (m, 1H, Ar–CH), 7.46 (dd, *J* = 7.7, 0.9 Hz, 1H, Ar–CH), 7.34–7.32 (m, 4H, Ar–CH), 7.28 (dd, *J* = 6.9, 1.0 Hz, 1H, Ar–CH), 7.23–7.17 (m, 2H, Ar–CH), 7.14–7.12 (m, 4H, Ar–CH), 5.78 (dd, *J* = 17.6, 1.2 Hz, 1H, CH_2), 5.30 (dd, *J* = 11.0, 1.1 Hz, 1H, CH_2), 5.20 (s, 1H, CH), 2.32 (s, 6H, CH_3); ^{13}C NMR (126 MHz, $CDCl_3$, 298 K) δ: 139.2, 139.1, 136.5, 135.3, 132.7, 129.4, 128.1, 127.8, 127.5, 124.6, 122.5, 115.4, 95.4 (C≡C), 83.0 (C≡C), 43.3 (CH), 21.1 (CH_3); IR ν_{max} (cm^{-1}): 3018 , 2921, 2182 (C≡C), 1645 (C=C), 1493, 1477, 1447, 1413, 1379, 1327, 1297, 1260, 1186, 1118, 1100, 1021; HRMS (ASAP+) $[M+H]^+$ $[C_{25}H_{23}]^+$: calculated 323.1800, found: 323.1793.

Synthesis of 4,4'-(3-(2-vinylphenyl)prop-2-yne-1,1-diyl)bis(methoxybenzene) (**2ap**)

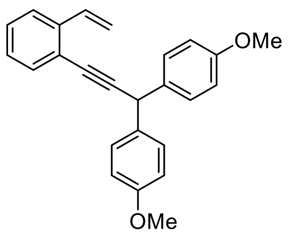

Synthesized in accordance with *General Procedure b* using $B(C_6F_5)_3$ (51 mg, 0.1 mmol), Mes_3P (39 mg, 0.1 mmol), aryl ester **1d** (37 mg, 0.1 mmol) and 1-ethynyl-2-vinylbenzene (**4d**) (15 mg, 0.12 mmol) in TFT to afford **2ap**. The crude reaction mixture was purified *via* preparative thin layer chromatography using hexane/ethyl acetate (92:08 v/v) as eluent. The desired compound **2ap** was obtained as a yellow liquid. Yield: 18 mg, 0.05 mmol, 51%.

^{1}H NMR (500 MHz, $CDCl_3$, 298 K) δ: 7.58–7.56 (m, 1H, Ar–CH), 7.47–7.45 (m, 1H, Ar–CH), 7.36–7.33 (m, 4H, Ar–CH), 7.24–7.17 (m, 3H, Ar–CH), 6.88–6.85 (m, 4H, Ar–CH), 5.79 (dd, *J* = 17.6, 1.1 Hz, 1H, CH_2), 5.31 (dd, *J* = 11.0, 1.1 Hz, 1H, CH_2), 5.18 (s, 1H, CH), 3.79 (s, 6H, OCH_3); ^{13}C NMR (126 MHz, $CDCl_3$, 298 K) δ: 158.5, 139.1, 138.9, 135.1, 134.3, 132.7, 130.9, 129.0, 128.9, 128.6, 128.2, 127.5, 124.6, 115.5, 114.1, 114.0, 113.9, 95.5 (C≡C), 83.0 (C≡C), 55.4 (OCH_3), 42.4 (CH); IR ν_{max} (cm^{-1}): 3036, 2957, 2177 (C≡C), 1640 (C=C), 1613, 1583, 1559, 1540, 1508, 1461, 1441, 1301, 1246, 1175, 1110, 1034; HRMS (ASAP+) $[M+H]^+$ $[C_{25}H_{22}O_2]^+$: calculated 355.1698, found 355.1702.

Synthesis of 1-(3-(4-chlorophenyl)-3-phenylprop-1-en-1-yl)-2-ethynylbenzene (**2aq**)

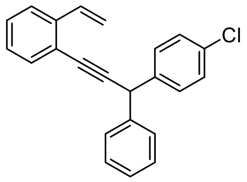

Synthesized in accordance with *General Procedure b* using $B(C_6F_5)_3$ (51 mg, 0.1 mmol), Mes_3P (39 mg, 0.1 mmol), 1-ethynyl-2-vinylbenzene (**4d**) (15 mg, 0.12 mmol) and aryl ester **1e** (34 mg, 0.1 mmol) in TFT to afford **2aq**. The crude reaction mixture was purified *via* preparative thin layer chromatography using hexane/ethyl acetate (97:03 v/v) as eluent. The desired compound **2aq** was obtained as a yellow liquid. Yield: 21 mg, 0.06 mmol, 60%.

^{1}H NMR (500 MHz, $CDCl_3$, 298 K) δ: 7.58–7.56 (m, 1H, Ar–CH), 7.46 (dd, *J* = 7.7, 0.9 Hz, 1H, Ar–CH), 7.44–7.42 (m, 2H, Ar–CH), 7.39–7.37 (m, 2H, Ar–CH), 7.36–7.32 (m, 2H, Ar–CH), 7.31–7.28 (m, 3H, Ar–CH), 7.24–7.15 (m, 3H, Ar–CH), 5.79 (dd, *J* = 17.6, 1.1 Hz, 1H, CH_2), 5.31 (dd, *J* = 11.0, 1.1 Hz, 1H, CH_2), 5.24 (s, 1H, CH); ^{13}C NMR (126 MHz, $CDCl_3$, 298 K) δ: 141.3, 140.4, 139.3, 135.1, 132.9, 132.7, 129.4, 128.9, 128.4, 127.9, 127.5, 127.2, 124.7, 122.0, 115.7, 94.4 (C≡C), 83.8 (C≡C), 43.5 (CH); IR ν_{max} (cm^{-1}): 2959, 2924, 2175 (C≡C), 1642 (C=C), 1539, 1490, 1477, 1449, 1261, 1091, 1015; HRMS (GC-MS) [M+] $[C_{23}H_{17}Cl]^+$: calculated 328.1019, found 328.1014.

Synthesis of 2-(4-(2-vinylphenyl)but-3-yn-2-yl)naphthalene (**2ar**)

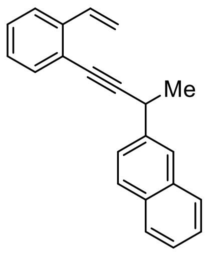

Synthesized in accordance with *General Procedure b* using $B(C_6F_5)_3$ (51 mg, 0.1 mmol), Mes_3P (39 mg, 0.1 mmol), 1-ethynyl-2-vinylbenzene (**4d**) (15 mg, 0.12 mmol) and aryl ester **1g** (29 mg, 0.1 mmol) in TFT to afford **2ar**. The crude reaction mixture was purified *via* preparative thin layer chromatography using hexane/ethyl acetate (98:02 v/v) as eluent. The desired compound **2ar** was obtained as a white solid. Yield: 14 mg, 0.05 mmol, 50%.

^{1}H NMR (500 MHz, $CDCl_3$, 298 K) δ: 7.92 (s, 1H, Ar–CH), 7.84 (dd, *J* = 8.0, 5.4 Hz, 3H, Ar–CH), 7.60–7.58 (m, 2H, Ar–CH), 7.50–7.44 (m, 3H, Ar–CH), 7.30–7.19 (m, 3H, Ar–CH), 5.82 (dd, *J* = 17.7, 1.2 Hz, 1H, CH_2), 5.34 (dd, *J* = 11.0, 1.1 Hz, 1H, CH_2), 4.21 (q, *J* = 7.1 Hz, 1H, CH), 1.70 (d, *J* = 7.2 Hz, 3H, CH_3); ^{13}C NMR (126 MHz, $CDCl_3$, 298 K) δ: 140.8, 139.1, 135.2, 133.6, 132.7, 132.5, 128.4, 128.1, 127.9, 127.7, 127.5, 126.2, 125.75, 125.74, 125.3, 124.7, 122.5, 115.4, 97.4 (C≡C), 81.2 (C≡C), 33.0 (CH), 24.5 (CH_3); IR ν_{max} (cm^{-1}): 3057, 2974, 2182 (C≡C), 1625 (C=C), 1600, 1559, 1507, 1476, 1447, 1374, 1363, 1302, 1260, 1198, 1126, 1100, 1073, 1054, 1019; HRMS (ASAP)+ $[M+H]^+$ $[C_{22}H_{19}]^+$: calculated 283.1487, found 283.1487.

Synthesis of 4,4'-(3-phenylprop-2-ene-1,1-diyl)bis(fluorobenzene) (**3a**)[17]

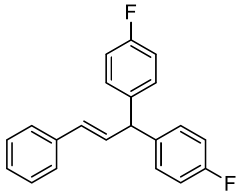

A competition reaction between equimolar mixture of styrene (11 mg, 0.1 mmol), diphenylacetylene (18 mg, 0.1 mmol) was carried out in accordance with *General Procedure b* using $B(C_6F_5)_3$ (51 mg, 0.1 mmol), Mes_3P (39 mg, 0.1 mmol), and aryl ester **1a** (34 mg, 0.1 mmol) in THF. The crude reaction mixture was purified *via* preparative thin layer chromatography using hexane/ethyl acetate (97:03 v/v) as eluent. The desired compound **3a** was obtained as a yellow liquid. Yield: 19 mg, 0.06 mmol, 63%.

^{1}H NMR (500 MHz, $CDCl_3$, 298 K) δ: 7.38–7.36 (m, 2H, Ar–CH), 7.32–7.29 (m, 2H, Ar–CH), 7.24–7.23 (m, 1H, Ar–CH), 7.19–7.16 (m, 4H, Ar–CH), 7.03–6.99 (m, 4H, Ar–CH), 6.59 (dd, J = 15.8, 7.4 Hz, 1H, CH_2), 6.31 (dd, *J* = 15.8, 1.3 Hz, 1H, CH_2), 4.87 (d, *J* = 7.4 Hz, 1H, CH); ^{13}C NMR (126 MHz, $CDCl_3$, 298 K) δ: 161.7 (d, $J_{C–F}$ = 245.1 Hz), 139.1 (d, $J_{C–F}$ = 3.3 Hz), 137.0, 132.2, 131.9, 130.1 (d, $J_{C–F}$ = 7.9 Hz), 128.7, 127.7, 126.4, 115.5 (d, $J_{C–F}$ = 21.3 Hz), 52.7 (CH); ^{19}F NMR (471 MHz, $CDCl_3$, 298 K) δ: -166.44.

Synthesis of 4,4'-(3-(4-(phenylethynyl)phenyl)prop-2-ene-1,1-diyl)bis(fluoro benzene) (**3b**)

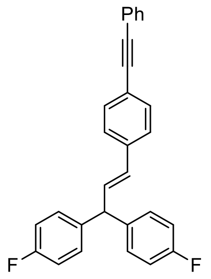

Synthesized in accordance with *General Procedure b* using $B(C_6F_5)_3$ (51 mg, 0.1 mmol), Mes_3P (39 mg, 0.1 mmol), 1-(phenylethynyl)-4-vinylbenzene (**4b**) (24 mg, 0.12 mmol) and aryl ester **1a** (34 mg, 0.1 mmol) in THF to afford **3b**. The crude reaction mixture was purified *via* preparative thin layer chromatography using hexane/ethyl acetate (97:03 v/v) as eluent. The desired compound **3b** was obtained as a yellow liquid. Yield: 29 mg, 0.07 mmol, 71%.

^{1}H NMR (500 MHz, $CDCl_3$, 298 K) δ: 7.71–7.69 (m, 2H, Ar–CH), 7.52–7.50 (m, 2H, Ar–CH), 7.39–7.36 (m, 5H, Ar–CH), 7.33–7.31 (m, 1H, Ar–CH), 7.25–7.22 (m, 4H, Ar–CH), 7.04–7.00 (m, 4H, Ar–CH), 6.76 (d, J = 9.9 Hz, 1H, CH_2), 5.56 (d, J = 9.9 Hz, 1H, CH_2); ^{13}C NMR (126 MHz, $CDCl_3$, 298 K) δ: 161.8 (d, $J_{C–F}$ = 245.2 Hz), 139.1 (d, $J_{C–F}$ = 3.3 Hz), 138.5, 137.6, 131.8, 129.9 (d, $J_{C–F}$ = 7.9 Hz), 128.7, 128.64, 128.60, 128.2, 126.4, 124.5, 123.1, 115.6 (d, $J_{C–F}$ = 21.3 Hz), 96.1 (C≡C), 86.4 (C≡C), 50.8 (CH); ^{19}F NMR (471 MHz, $CDCl_3$, 298 K) δ: -116.26 (2F, Ar–F); IR ν_{max} (cm^{-1}): 3047, 2984, 2172 (C≡C), 1640 (C=C), 1601, 1559, 1505, 1489, 1447, 1360, 1222, 1157, 1085, 1015; HRMS (ES+) [M+H]$^+$ [$C_{29}H_{21}F_2$]: calculated 407.1611, found 407.1607.

Synthesis of 4,4'-(3-(2-ethynylphenyl)prop-2-ene-1,1-diyl)bis(fluorobenzene) (**3c**)

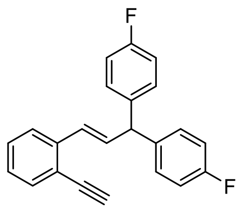

Synthesized in accordance with *General Procedure b* using $B(C_6F_5)_3$ (51 mg, 0.1 mmol), Mes_3P (39 mg, 0.1 mmol), 1-ethynyl-2-vinylbenzene (**4d**) (15 mg, 0.12 mmol) and aryl ester **1a** (34 mg, 0.1 mmol) in THF to afford **3c**. The crude reaction mixture was purified *via* preparative thin layer chromatography using hexane/ethyl acetate (96:04 v/v) as eluent. The desired compound **3c** was obtained as a yellow liquid. Yield: 26 mg, 0.07 mmol, 79%.

^{1}H NMR (500 MHz, $CDCl_3$, 298 K) δ: 7.57–7.55 (m, 1H, Ar–CH), 7.47 (dd, J = 7.7, 0.9 Hz, 1H, Ar–CH), 7.32–7.28 (m, 1H, Ar–CH), 7.21–7.17 (m, 5H, Ar–CH), 7.04–6.99 (m, 4H, Ar–CH), 6.87–6.83 (m, 1H, Ar–CH), 6.66 (dd, J = 15.8, 7.6 Hz, 1H, CH_2), 4.91 (d, J = 7.6 Hz, 1H, CH), 3.23 (s, 1H, C≡CH); ^{13}C NMR (126 MHz, $CDCl_3$, 298 K) δ: 161.7 (d, $J_{C–F}$ = 245.2 Hz), 139.2, 139.0 (d, $J_{C–F}$ = 3.1 Hz), 134.2, 133.3, 130.1 (d, $J_{C–F}$ = 7.9 Hz), 129.7, 129.0, 127.2, 125.0, 120.8, 115.5 (d, $J_{C–F}$ = 21.2 Hz), 82.0 (C≡C), 81.9 (C≡C), 52.9 (CH); ^{19}F NMR (471 MHz, $CDCl_3$, 298 K) δ: -116.41 (2F, Ar–F); IR ν_{max} (cm^{-1}): 3003, 2953, 2159 (C≡C), 1624

(C=C), 1505, 1474, 1445, 1259, 1221, 1157, 1096, 1015; HRMS (GC-MS) [M] [$C_{23}H_{16}F_2$]: calculated 330.1220, found 330.1219.

Synthesis of 4,4'-(3-(2-ethynylphenyl)prop-2-ene-1,1-diyl)bis(chlorobenzene) (**3d**)

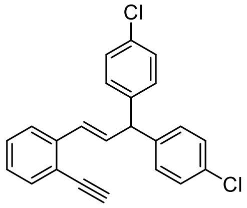

Synthesized in accordance with *General Procedure b* using $B(C_6F_5)_3$ (51 mg, 0.1 mmol), Mes_3P (39 mg, 0.1 mmol), 1-ethynyl-2-vinylbenzene (**4d**) (15 mg, 0.12 mmol) and aryl ester **1b** (37 mg, 0.1 mmol) in THF to afford **3d**. The crude reaction mixture was purified *via* preparative thin layer chromatography using hexane/ethyl acetate (98:02 v/v) as eluent. The desired compound **3d** was obtained as a yellow liquid. Yield: 27 mg, 0.07 mmol, 74%.

^{1}H NMR (500 MHz, $CDCl_3$, 298 K) δ: 7.54 (d, *J* = 7.9 Hz, 1H, Ar–CH), 7.47 (dd, *J* = 7.8, 1.4 Hz, 1H, Ar–CH), 7.32–7.28 (m, 5H, Ar–CH), 7.21–7.14 (m, 5H, Ar–CH), 6.88–6.85 (m, 1H, CH_2), 6.63 (dd, *J* = 15.9, 7.7 Hz, 1H, CH_2), 4.88 (d, *J* = 7.6 Hz, 1H, CH), 3.24 (s, 1H, C≡CH); ^{13}C NMR (126 MHz, $CDCl_3$, 298 K) δ: 141.5, 139.0, 133.5, 133.3, 132.7, 130.2, 130.0, 129.0, 128.8, 127.3, 125.1, 120.8, 82.0 (C≡C), 81.9 (C≡C), 53.2 (CH); IR ν_{max} (cm^{-1}): 3027, 2926, 2172 (C≡C), 1637 (C=C), 1488, 1408, 1260, 1089, 1089, 1014; HRMS (ES-) [M-H]$^-$ calculated for [$C_{23}H_{15}Cl_2$]$^-$: 361.0511, found: 361.0545.

Synthesis of 3-(2-ethynylphenyl)prop-2-ene-1,1-diyl)dibenzene (**3e**)

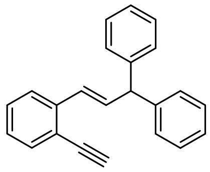

Synthesized in accordance with *General Procedure b* using $B(C_6F_5)_3$ (51 mg, 0.1 mmol), Mes_3P (39 mg, 0.1 mmol), 1-ethynyl-2-vinylbenzene (**4d**) (15 mg, 0.12 mmol) and aryl ester **1c** (31 mg, 0.1 mmol) in THF to afford **3e**. The crude reaction mixture was purified *via* preparative thin layer chromatography using hexane/ethyl acetate (97:03 v/v) as eluent. The desired compound **3e** was obtained as a colorless liquid. Yield: 21 mg, 0.07 mmol, 71%.

^{1}H NMR (500 MHz, $CDCl_3$, 298 K) δ: 7.58–7.56 (m, 1H, Ar–CH), 7.45 (dd, *J* = 7.7, 0.9 Hz, 1H, Ar–CH), 7.35–7.27 (m, 7H, Ar–CH), 7.26–7.20 (m, 4H, Ar–CH), 7.16 (td, *J* = 7.6, 1.3 Hz, 1H, Ar–CH), 6.92–6.88 (m, 1H, CH_2), 6.74 (dd, *J* = 15.8, 7.8 Hz, 1H, CH_2), 4.94 (d, *J* = 7.8 Hz, 1H, CH), 3.22 (s, 1H, C≡CH); ^{13}C NMR (126 MHz, $CDCl_3$, 298 K) δ: 143.5, 139.5, 134.7, 133.2, 129.4, 129.0, 128.7, 128.6, 127.0, 126.6, 125.0, 120.7, 82.1 (C≡C), 81.8 (C≡C), 54.5 (CH); IR ν_{max} (cm^{-1}): 3302 (C≡CH), 3061, 3026, 2247 (C≡C), 1645 (C=C), 1595, 1492, 1475, 1446, 1029; HRMS (ASAP+) [M+H]$^+$ [$C_{23}H_{19}$]$^+$: calculated 295.1487, found 295.1495.

Synthesis of 4,4'-(3-(2-ethynylphenyl)prop-2-ene-1,1-diyl)bis(methylbenzene) (**3f**)

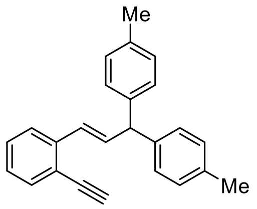

Synthesized in accordance with *General Procedure b* using $B(C_6F_5)_3$ (51 mg, 0.1 mmol), Mes_3P (39 mg, 0.1 mmol), 1-ethynyl-2-vinylbenzene (**4d**) (15 mg, 0.12 mmol) and aryl ester **1i** (33 mg, 0.1 mmol) in THF to afford **3f**. The crude reaction mixture was purified *via* preparative thin layer chromatography using hexane/ethyl acetate (98:02 v/v) as eluent. The desired compound **4d** was obtained as a colorless liquid. Yield: 18 mg, 0.06 mmol, 56%.

^{1}H NMR (500 MHz, $CDCl_3$, 298 K) δ: 7.57–7.55 (m, 1H, Ar–CH), 7.45 (dd, *J* = 7.8, 1.0 Hz, 1H, Ar–CH), 7.17–7.10 (m, 10H, Ar–CH), 6.90 (d, *J* = 15.9 Hz, 1H, CH_2), 6.71 (dd, *J* = 15.8, 8.1 Hz, 1H, CH_2), 4.86 (d, *J* = 8.0 Hz, 1H, CH), 3.24 (s, 1H, C≡CH), 2.32 (s, 6H, CH_3); ^{13}C NMR (126 MHz, $CDCl_3$, 298 K) δ: 140.8, 139.7, 136.0, 135.1, 133.2, 129.3, 129.1, 128.98, 128.97, 128.5, 127.2, 126.9, 125.1, 120.6, 82.2 (C≡C), 81.7 (C≡C), 53.8 (CH), 21.1 (CH_3); IR ν_{max} (cm^{-1}): 3002, 2964, 2923, 2170 (C≡C), 1654 (C=C), 1558, 1511, 1475, 1445, 1259, 1091, 1020; HRMS (ASAP +) $[M+H]^+$ $[C_{25}H_{23}]^+$: calculated 323.1800, found 323.1801.

Synthesis of 1-(3-(4-chlorophenyl)-3-phenylprop-1-en-1-yl)-2-ethynylbenzene (**3h**)

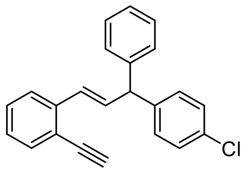

Synthesized in accordance with *General Procedure b* using $B(C_6F_5)_3$ (51 mg, 0.1 mmol), Mes_3P (39 mg, 0.1 mmol), 1-ethynyl-2-vinylbenzene (**4d**) (15 mg, 0.12 mmol) and aryl ester **1e** (34 mg, 0.1 mmol) in THF to afford **3h**. The crude reaction mixture was purified *via* preparative thin layer chromatography using hexane/ethyl acetate (97:03 v/v) as eluent. The desired compound **3h** was obtained as a yellow liquid. Yield: 24 mg, 0.07 mmol, 73%.

^{1}H NMR (500 MHz, $CDCl_3$, 298 K) δ: 7.57–7.55 (m, 1H, Ar–CH), 7.47 (dd, *J* = 7.7, 0.9 Hz, 1H, Ar–CH), 7.34–7.28 (m, 6H, Ar–CH), 7.25–7.23 (m, 2H, Ar–CH), 7.20–7.18 (m, 3H, Ar–CH), 6.91–6.87 (m, 1H, Ar–CH), 6.69 (dd, *J* = 15.8, 7.8 Hz, 1H, CH_2), 4.92 (d, *J* = 7.7 Hz, 1H, CH), 3.24 (s, 1H, C≡CH); ^{13}C NMR (126 MHz, $CDCl_3$, 298 K) δ: 143.0, 142.0, 139.3, 134.1, 133.3, 132.4, 130.1, 129.8, 129.0, 128.75, 128.73, 128.70, 127.2, 126.8, 125.0, 120.7, 82.0 (C≡C), 81.9 (C≡C), 53.8 (CH); IR ν_{max} (cm^{-1}): 3025, 2963, 2928, 2187 (C≡C), 1653 (C=C), 1600, 1490, 1475, 1447, 1405, 1259, 1090, 1014; HRMS (GC-MS) $[M]^+$ $[C_{23}H_{17}Cl]^+$: calculated 328.1019, found 328.1016.

Synthesis of 2-(4-(2-ethynylphenyl)but-3-en-2-yl)naphthalene (**3i**)

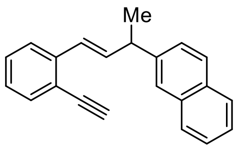

Synthesized in accordance with *General Procedure b* using $B(C_6F_5)_3$ (51 mg, 0.1 mmol), Mes_3P (39 mg, 0.1 mmol), 1-ethynyl-2-vinylbenzene (**4d**) (15 mg, 0.12 mmol) and aryl ester **1g** (29 mg, 0.1 mmol) in THF to afford **3i**. The crude reaction mixture was purified *via* preparative thin layer chromatography using hexane/ethyl acetate (98:02 v/v) as eluent. The desired compound **3i** was obtained as a white solid. Yield: 11 mg, 0.04 mmol, 40%.

^{1}H NMR (500 MHz, $CDCl_3$, 298 K) δ: 7.82–7.80 (m, 3H, Ar–CH), 7.72 (s, 1H, Ar–CH), 7.54 (d, J = 8.0 Hz, 1H, Ar–CH), 7.49–7.43 (m, 4 H, Ar–CH), 7.28 (d, *J* = 7.5 Hz, 1H, Ar–CH), 7.16 (t, *J* = 7.6 Hz, 1H, Ar–CH), 7.03 (d, *J* = 15.9 Hz, 1H, CH_2), 6.53 (dd, *J* = 15.9, 7.0 Hz, 1H, CH_2), 3.90–3.84 (m, 1H, CH), 3.31 (s, 1H, C≡CH), 1.59 (d, *J* = 7.0 Hz, 3H, CH_3); ^{13}C NMR (126 MHz, $CDCl_3$, 298 K) δ: 143.0, 139.8, 137.3, 133.8, 133.2, 132.4, 129.0, 128.2, 127.8, 127.7, 126.8, 126.7, 126.4, 126.1, 125.5, 125.3, 124.8, 120.6, 82.3 (C≡C), 81.7 (C≡C), 43.0 (CH), 21.2 (CH_3); IR ν_{max} (cm^{-1}): 3054, 2963, 2927, 2185 (C≡C), 1644 (C=C), 1559, 1549, 1506, 1474, 1446, 1373, 1362, 1270, 1260, 1160, 1018; HRMS (ASAP+) $[M]^+$ $[C_{22}H_{18}]^+$: calculated 282.1409, found 282.1408.

Synthesis of ((1E)-5-(2-ethynylphenyl)penta-1,4-diene-1,3-diyl)dibenzene (**3j**)

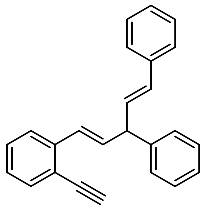

Synthesized in accordance with *General Procedure b* using $B(C_6F_5)_3$ (51 mg, 0.1 mmol), Mes_3P (39 mg, 0.1 mmol), 1-ethynyl-2-vinylbenzene (**4d**) (15 mg, 0.12 mmol) and aryl ester **1k** (31 mg, 0.1 mmol) in THF to afford **3j**. The crude reaction mixture was purified *via* preparative thin layer chromatography using hexane/ethyl acetate (96:04 v/v) as eluent. The desired compound **3j** was obtained as a colorless liquid. Yield: 7 mg, 0.02 mmol, 22%.

^{1}H NMR (500 MHz, $CDCl_3$, 298 K) δ: 7.58–7.56 (m, 1H, Ar–CH), 7.48 (dd, *J* = 7.7, 0.9 Hz, 1H, Ar–CH), 7.41–7.39 (m, 2H, Ar–CH), 7.35–7.34 (m, 4H, Ar–CH), 7.32–7.27 (m, 4H, Ar–CH) 7.25–7.20 (m, 2H, Ar–CH), 7.18 (td, *J* = 7.6, 1.3 Hz, 1H, CH_2), 7.04–7.01 (m, 1H, CH_2), 6.55 (dd, *J* = 15.9, 7.4 Hz, 1H, CH_2), 6.51–6.50 (m, 1H, CH_2), 4.46–4.43 (m, 1H, CH), 3.27 (s, 1H, C≡CH); ^{13}C NMR (126 MHz, $CDCl_3$, 298 K) δ: 142.8, 139.5, 137.5, 134.0, 133.3, 131.9, 131.1, 129.0, 128.8, 128.6, 128.2, 127.4, 127.0, 126.8, 126.4, 125.0, 120.7, 82.2 (C≡C), 81.8 (C≡C), 52.0 (CH); IR ν_{max} (cm^{-1}): 3025, 2960, 2921, 2173 (C≡C), 1653, 1646 (C=C), 1598, 1575, 1570, 1559, 1542, 1533, 1520, 1507, 1492, 1473, 1447, 1377, 1363, 1258, 1080, 1011; HRMS (ASAP+) $[M+H]^+$: $[C_{25}H_{21}]^+$ calculated 321.1643, found 321.1647.

Synthesis of 4,4'-(3-(2-(3,3-di-p-tolylprop-1-en-1-yl)phenyl)prop-2-yne-1,1-diyl)bis (methyl benzene) (**5d**)

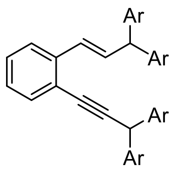

Ar = *p*MeC$_6$H$_4$

Synthesized in accordance with *General Procedure b* using B(C$_6$F$_5$)$_3$ (51 mg, 0.1 mmol), Mes$_3$P (39 mg, 0.1 mmol), 1-ethynyl-2-vinylbenzene (**4d**) (15 mg, 0.12 mmol) and aryl ester **1i** (33 mg, 0.1 mmol) in THF to afford **5d**. The crude reaction mixture was purified *via* preparative thin layer chromatography using hexane/ethyl acetate (98:02 v/v) as eluent. The desired compound **5d** was obtained as a colorless liquid. Yield: 5 mg, 0.01 mmol, 10%.

^{1}H NMR (500 MHz, CDCl$_3$, 298 K) δ: 7.50–7.49 (m, 1H, Ar–CH), 7.38 (dd, *J* = 7.7, 1.0 Hz, 1H, Ar–CH), 7.24–7.18 (m, 6H, Ar–CH), 7.11–7.03 (m, 12H, Ar–CH), 6.87 (d, *J* = 15.8 Hz, 1H, CH$_2$), 6.63 (dd, *J* = 15.8, 8.0 Hz, 1H, CH$_2$), 5.08 (s, 1H, CH), 4.72 (d, *J* = 8.0 Hz, 1H, CH), 2.27 (s, 6H, CH$_3$), 2.26 (s, 6H, CH$_3$); ^{13}C NMR (126 MHz, CDCl$_3$, 298 K) δ: 140.9, 139.3, 139.1, 136.4, 135.9, 134.6, 132.5, 129.5, 129.3, 129.2, 128.5, 128.1, 127.8, 126.8, 125.1, 122.2, 95.4 (C≡C), 83.3 (C≡C), 53.9 (CH), 43.3 (CH), 21.18 (CH$_3$), 21.16 (CH$_3$); IR ν_{max} (cm^{-1}): 3020, 2919, 2176 (C≡C), 1653 (C=C), 1509, 1476, 1445, 1412, 1378, 1327, 1296, 1263, 1211, 1186, 1110, 1038, 1020; HRMS (ASAP+) [M+H]$^+$ [C$_{40}$H$_{37}$]$^+$: calculated 517.2895, found 517.2894.

Synthesis of 2-(4-(2-(3-(naphthalen-2-yl)but-1-en-1-yl)phenyl)but-3-yn-2-yl)naphthalene (**5g**)

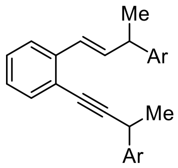

Ar = 2-napthyl

Synthesized in accordance with *General Procedure b* using B(C$_6$F$_5$)$_3$ (51 mg, 0.1 mmol), Mes$_3$P (39 mg, 0.1 mmol), 1-ethynyl-2-vinylbenzene (**4d**) (15 mg, 0.12 mmol) and aryl ester **1g** (29 mg, 0.1 mmol) in THF to afford **5g**. The crude reaction mixture was purified *via* preparative thin layer chromatography using hexane/ethyl acetate (98:02 v/v) as eluent. The desired compound **5g** was obtained as a white solid. Yield: 6 mg, 0.01 mmol, 13%.

^{1}H NMR (500 MHz, CDCl$_3$, 298 K) δ: 7.87 (t, *J* = 1.9 Hz, 1H, Ar–CH), 7.82–7.73 (m, 6H, Ar–CH), 7.68–7.65 (m, 1H, Ar–CH), 7.57–7.54 (m, 1H, Ar–CH), 7.53 (d, *J* = 7.3 Hz, 1H, Ar–CH), 7.46–7.36 (m, 6H, Ar–CH), 7.23 (td, *J* = 8.2, 1.5 Hz, 1H, Ar–CH), 7.15 (td, *J* = 7.4, 1.2 Hz, 1H, Ar–H), 7.00 (dd, *J* = 16.0, 1.5 Hz, 1H, CH$_2$), 6.53 (ddd, *J* = 16.0, 6.6, 2.5 Hz, 1H, CH$_2$), 4.12 (q, *J* = 7.0 Hz, 1H, CH), 3.79 (h, *J* = 6.1 Hz, 1H, CH), 1.59 (dd, *J* = 7.1, 4.7 Hz, 3H, CH$_3$), 1.51 (dd, *J* = 7.0, 5.6 Hz, 3H, CH$_3$); ^{13}C NMR (126 MHz, CDCl$_3$, 298 K) δ: 143.0, 140.8, 139.2, 136.7, 133.7, 133.6, 132.58, 132.57, 132.3, 128.4, 128.1, 128.0, 127.9, 127.8, 127.77, 127.72, 127.3, 126.8, 126.5, 126.4, 126.2, 126.0, 125.7, 125.4, 125.27, 125.26, 124.8, 122.2, 97.4 (C≡C), 81.3 (C≡C), 42.9 (CH), 32.9 (CH), 24.3 (CH$_3$), 21.0 (CH$_3$); IR ν_{max} (cm^{-1}): 3059, 2960,

2925, 1700, 1680, 1635, 1600, 1559, 1507, 1476, 1457, 1448, 1374, 1363, 1270, 1126, 1019; HRMS (ASAP+) $[M+H]^+$ $[C_{34}H_{28}]^+$: calculated 437.2269, found 437.2264.

Synthesis of ((1E)-5-(2-((E)-3,5-diphenylpent-4-en-1-yn-1-yl)phenyl)penta-1,4-diene-1,3-diyl)dibenzene (**5h**)

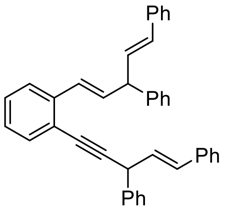

Synthesized in accordance with *General Procedure b* using $B(C_6F_5)_3$ (51 mg, 0.1 mmol), Mes_3P (39 mg, 0.1 mmol), 1-ethynyl-2-vinylbenzene (**4d**) (15 mg, 0.12 mmol) and aryl ester **1k** (31 mg, 0.1 mmol) in THF to afford **5h**. The crude reaction mixture was purified *via* preparative thin layer chromatography using hexane/ethyl acetate (96:04 v/v) as eluent. The desired compound **5h** was obtained as a colorless liquid. Yield: 21 mg, 0.04 mmol, 41%.

^{1}H NMR (500 MHz, $CDCl_3$, 298 K) δ: 7.57 (dd, *J* = 8.0, 0.7 Hz, 1H, Ar–CH), 7.49–7.45 (m, 3H, Ar–CH), 7.37–7.27 (m, 14H, Ar–CH), 7.26–7.17 (m, 6H, Ar–CH), 7.06 (d, *J* = 15.9 Hz, 1H, CH_2), 6.76 (dd, *J* = 15.3, 1.2 Hz, 1H, CH_2), 6.56 (ddd, *J* = 15.9, 7.3, 1.5 Hz, 1H, CH_2), 6.46–6.45 (m, 2H, CH_2), 6.30 (ddd, *J* = 15.6, 6.5, 2.1 Hz, 1H, CH_2), 4.73 (dd, *J* = 6.5, 1.5 Hz, 1H, CH), 4.38–4.35 (m, 1H, CH); ^{13}C NMR (126 MHz, $CDCl_3$, 298 K) δ: 142.8, 140.4, 139.0, 137.4, 136.9, 133.7, 132.7, 131.9, 131.0, 130.6, 129.6, 129.3, 128.8, 128.7, 128.67, 128.63, 128.3, 128.2, 127.9, 127.6, 127.4, 127.2, 127.0, 126.7, 126.6, 126.4, 125.1, 122.0, 93.7 (C≡C), 84.1 (C≡C), 52.0 (CH), 41.5 (CH); IR ν_{max} (cm^{-1}): 3025, 2956, 2929, 2185 (C≡C), 1647 (C=C), 1630 (C=C), 1598, 1491, 1476, 1447, 1309, 1264, 1203, 1156, 1074, 1029; HRMS (ASAP+) $[M+H]^+$ $[C_{40}H_{33}]^+$: calculated 513.2582, found: 513.2579.

3. NMR Spectra

Figure S1: ^{1}**H NMR** (400 MHz, $CDCl_3$, 298 K) spectrum of **1a**.

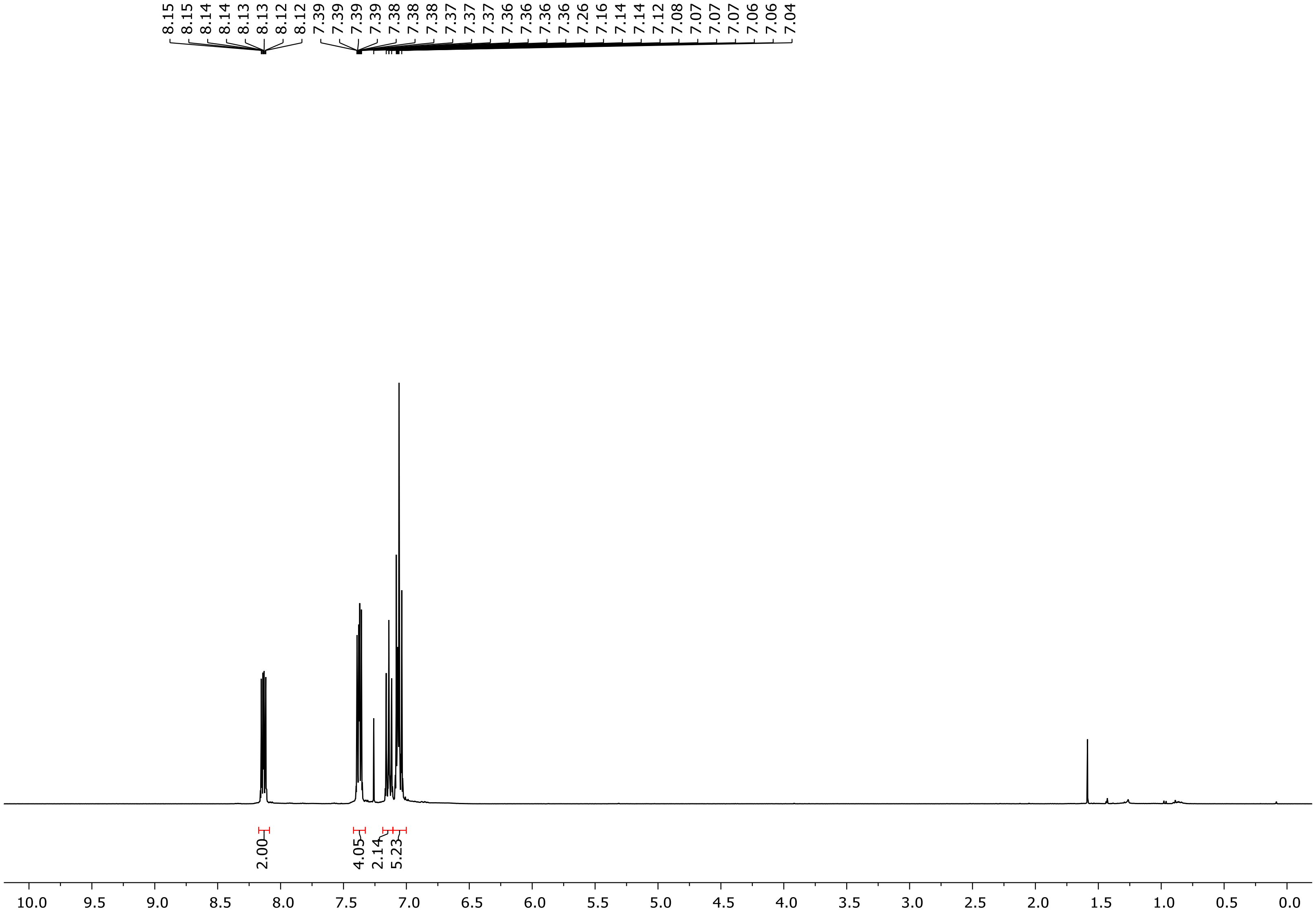

Figure S2: **^{13}C NMR** (101 MHz, $CDCl_3$, 298 K) spectrum of **1a**.

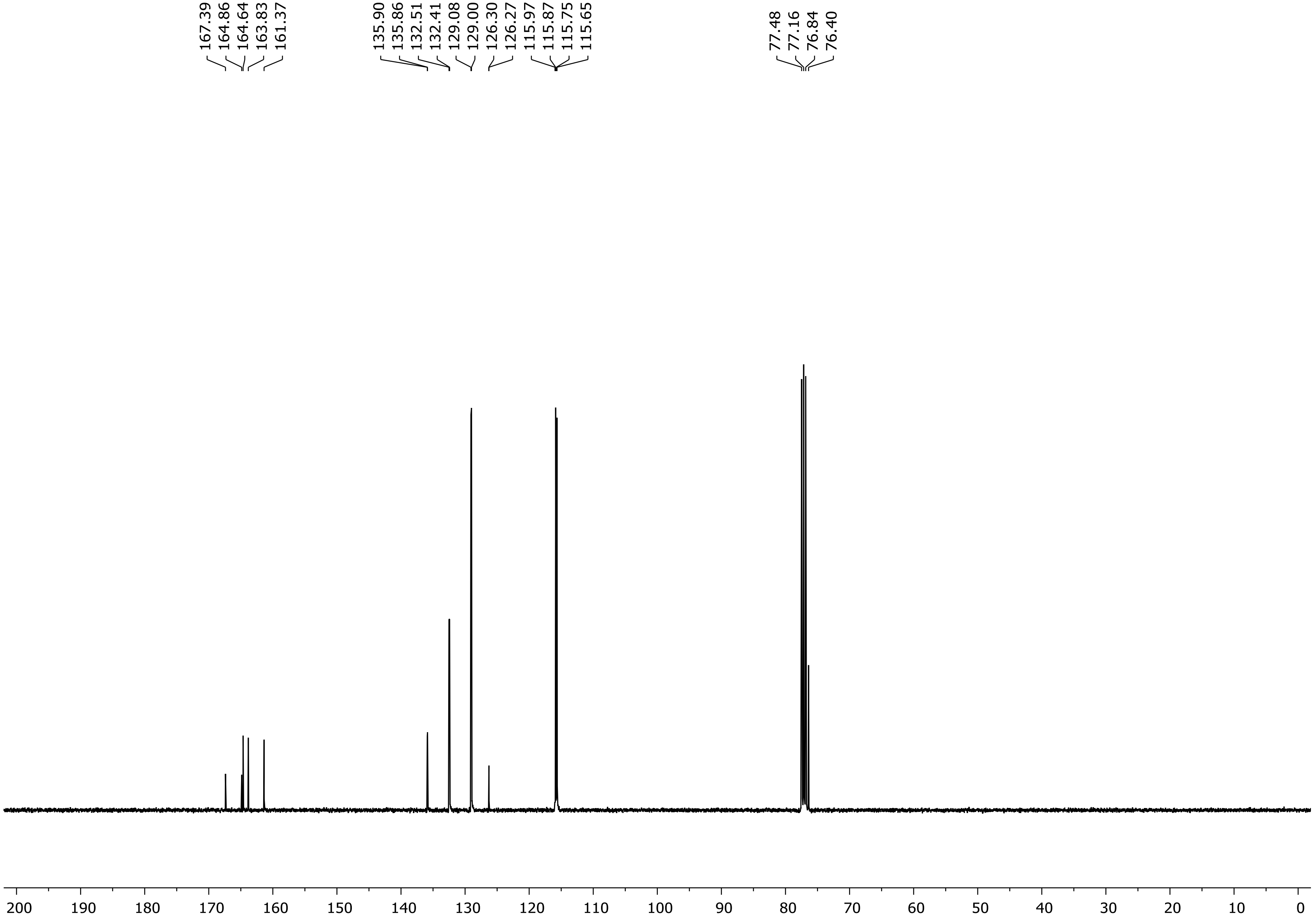

Figure S3: **^{19}F NMR** (376 MHz, $CDCl_3$, 298 K) spectrum of **1a**.

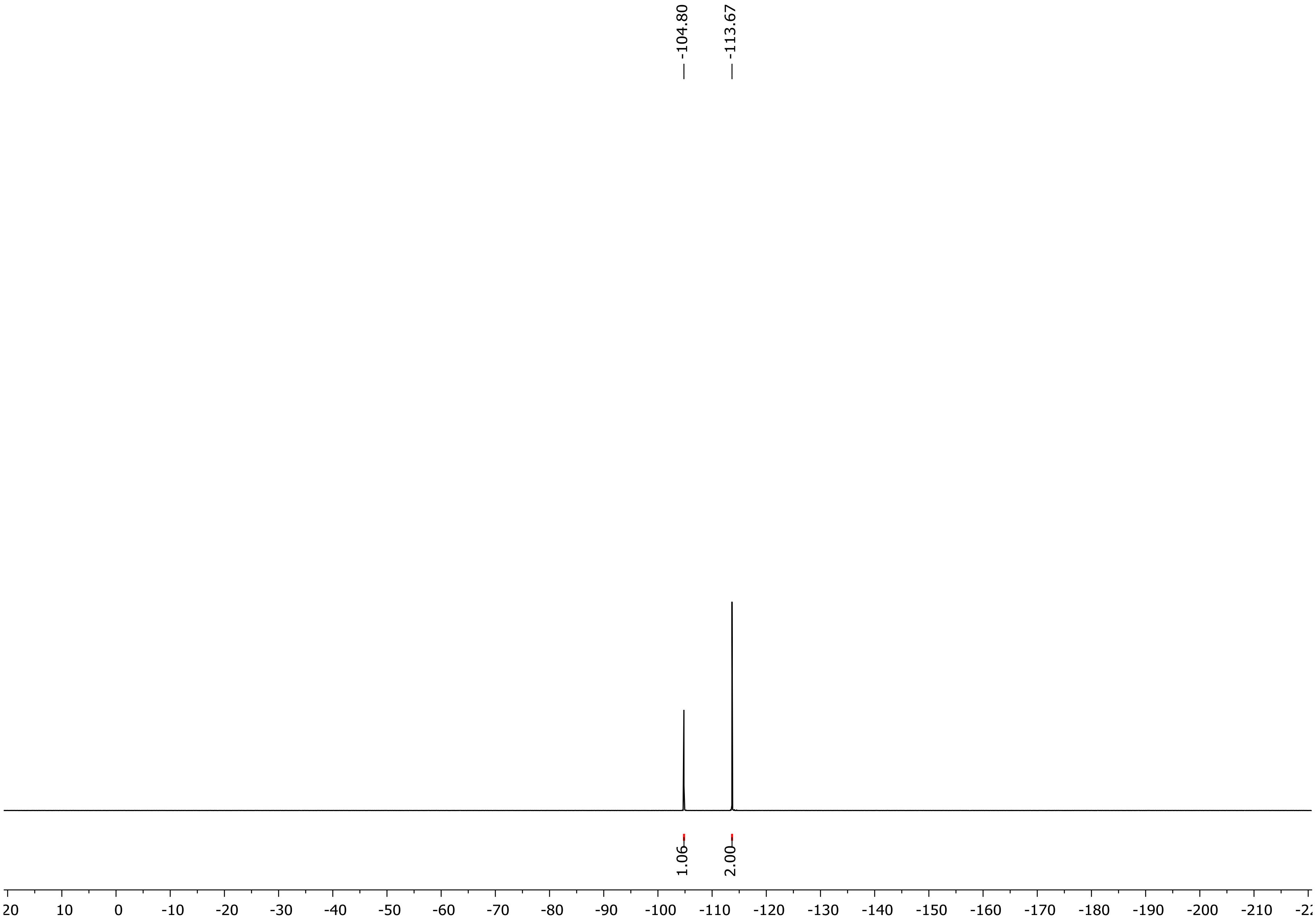

Figure S4: **^{1}H NMR** (400 MHz, $CDCl_3$, 298 K) spectrum of **1b**.

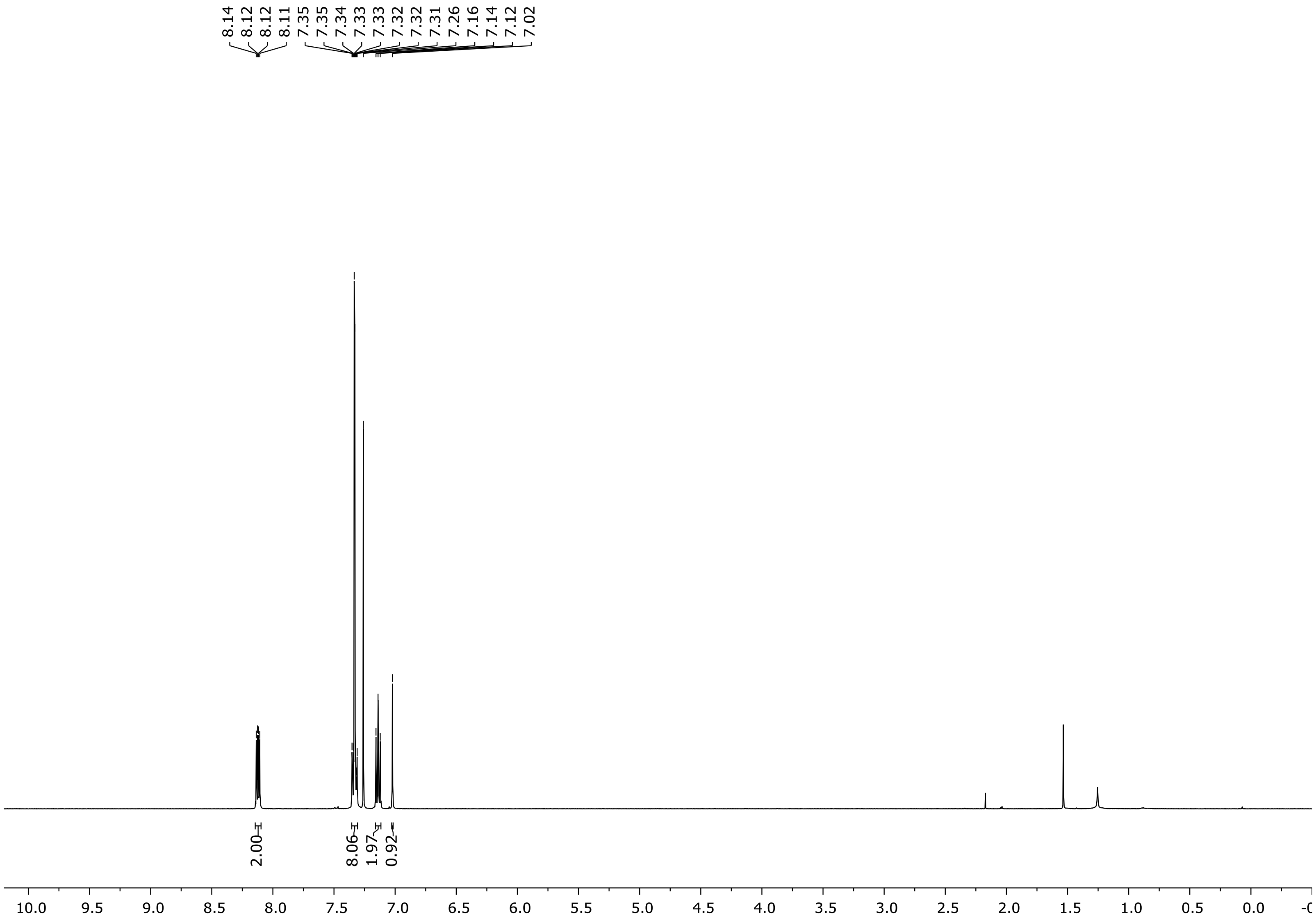

Figure S5: **^{13}C NMR** (126 MHz, $CDCl_3$, 298 K) spectrum of **1b**.

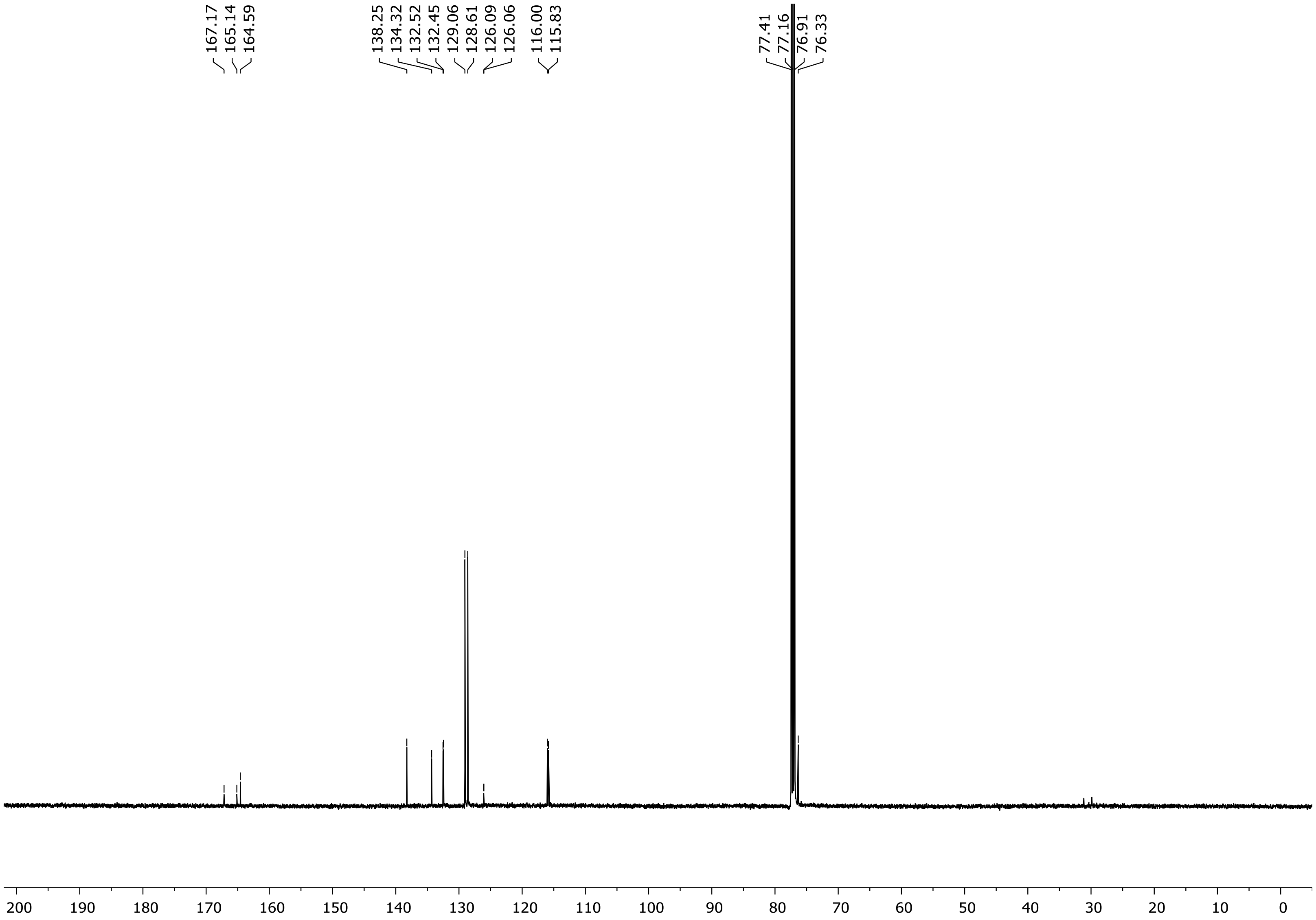

Figure S6: ^{19}**F NMR** (471MHz, $CDCl_3$, 298 K) spectrum of **1b**.

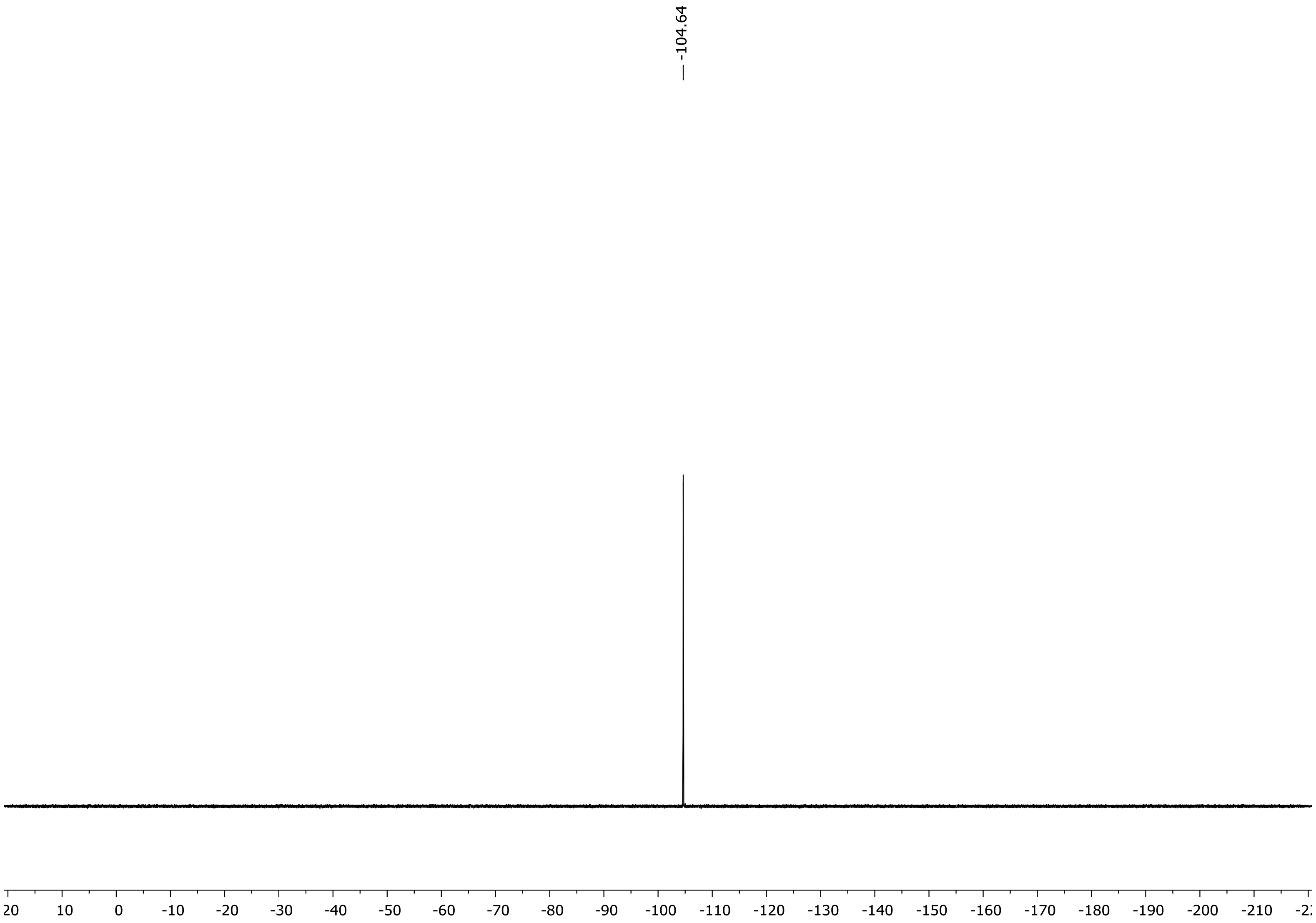

Figure S7: **^{1}H NMR** (400 MHz, $CDCl_3$, 298 K) spectrum of **1c**.

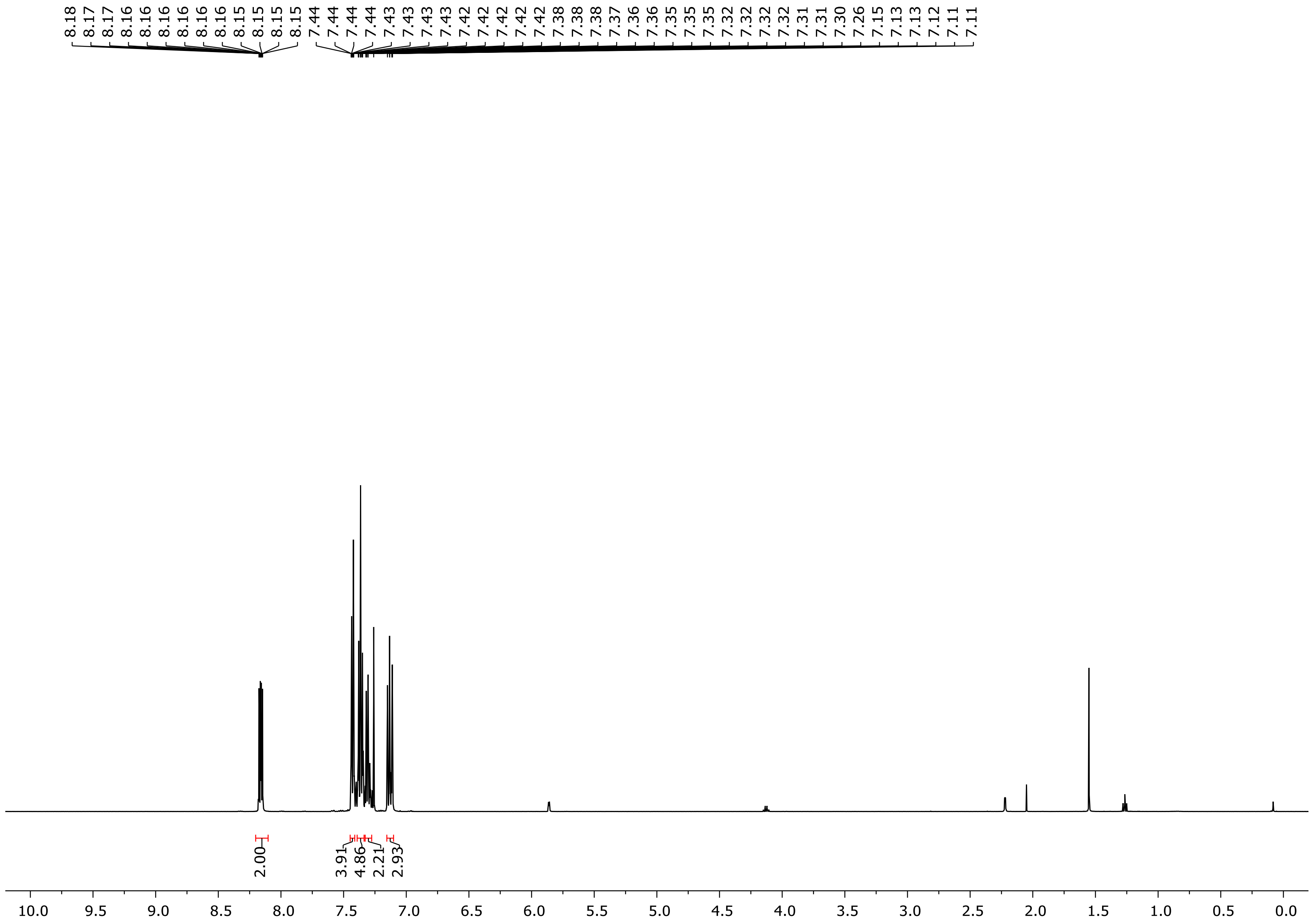

Figure S8: ^{13}**C NMR** (101 MHz, $CDCl_3$, 298 K) spectrum of **1c**.

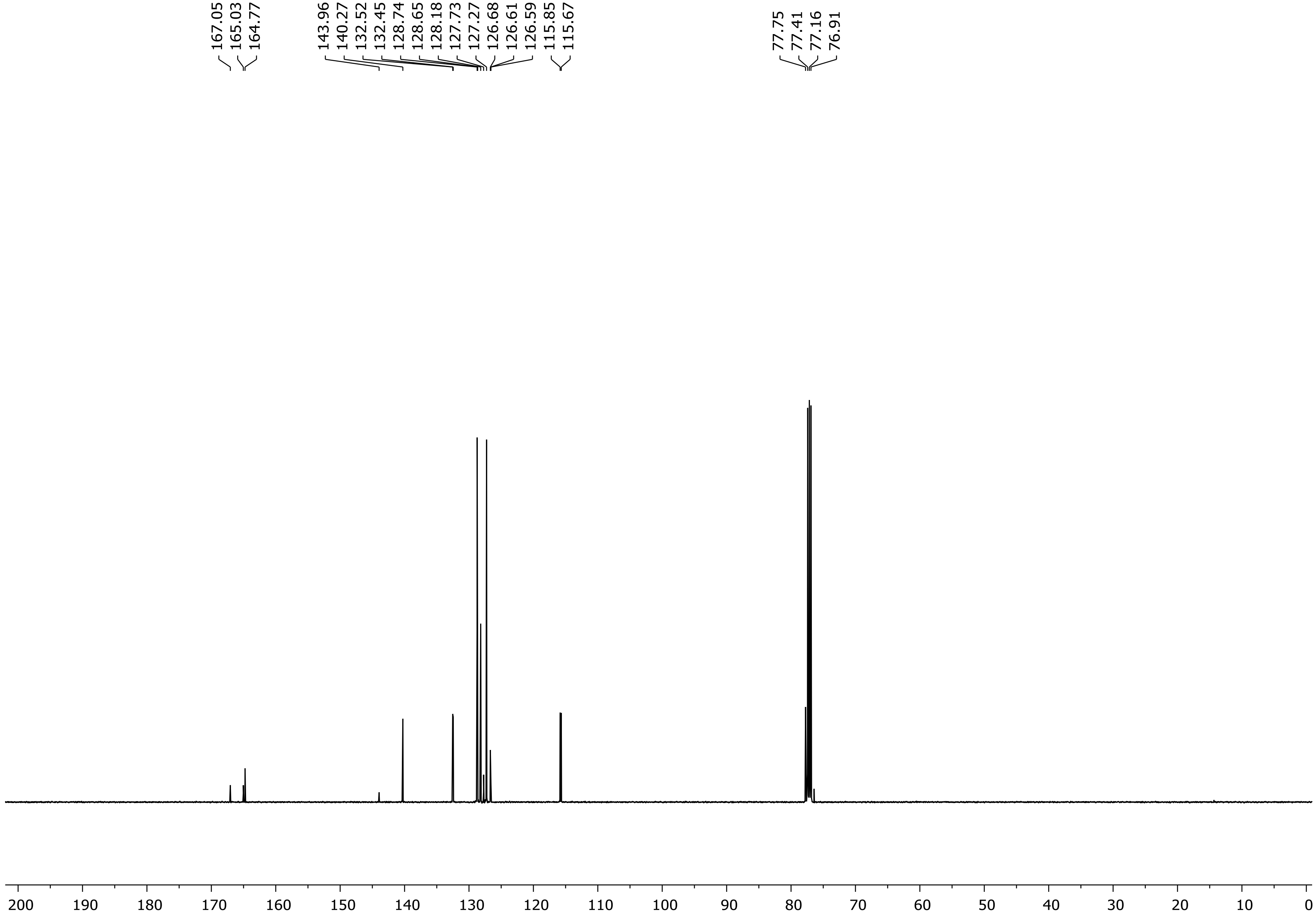

Figure S9: ^{19}**F NMR** (376 MHz, $CDCl_3$, 298 K) spectrum of **1c**.

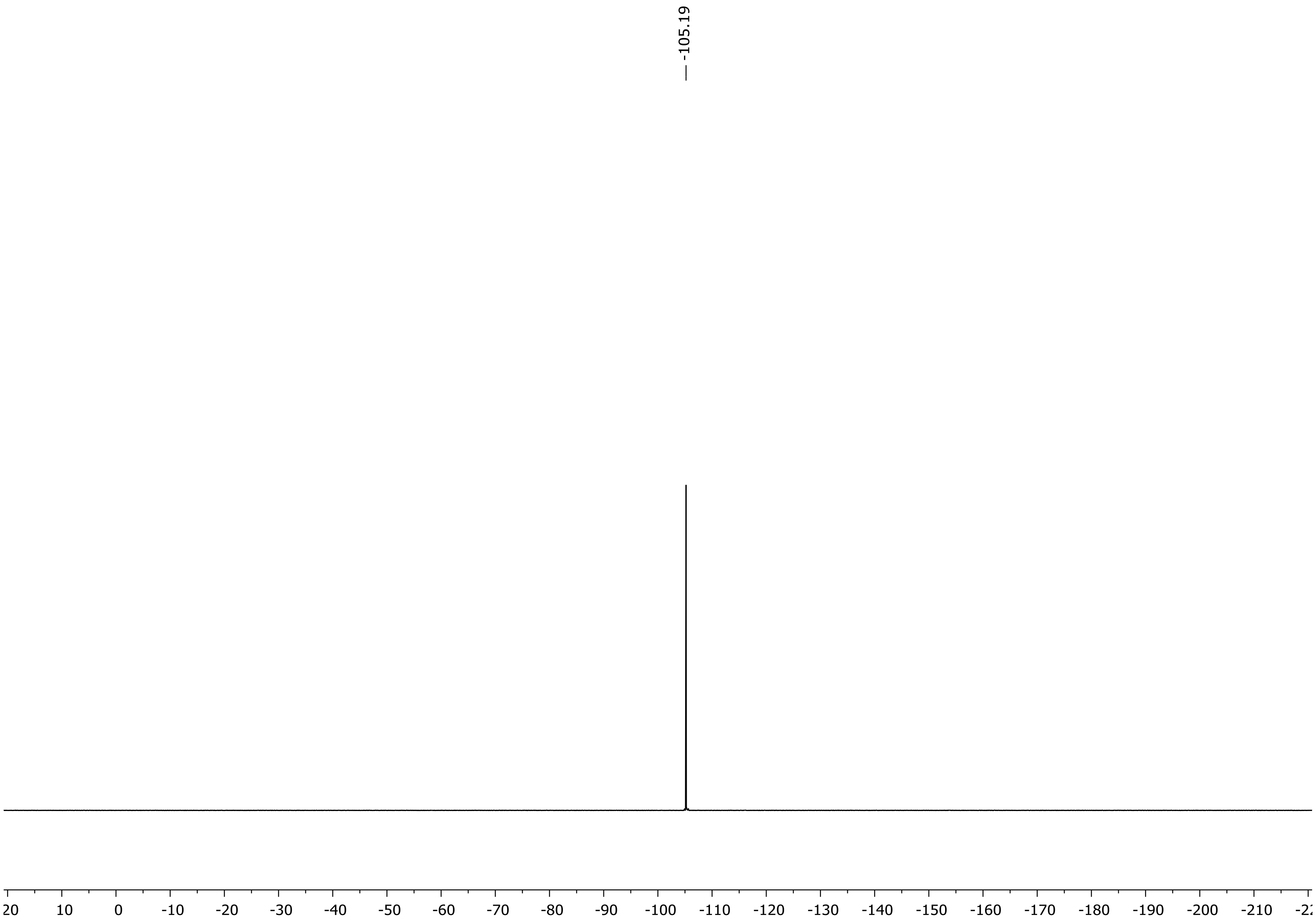

Figure S10: **^{1}H NMR** (400 MHz, $CDCl_3$, 298 K) spectrum of **1d**.

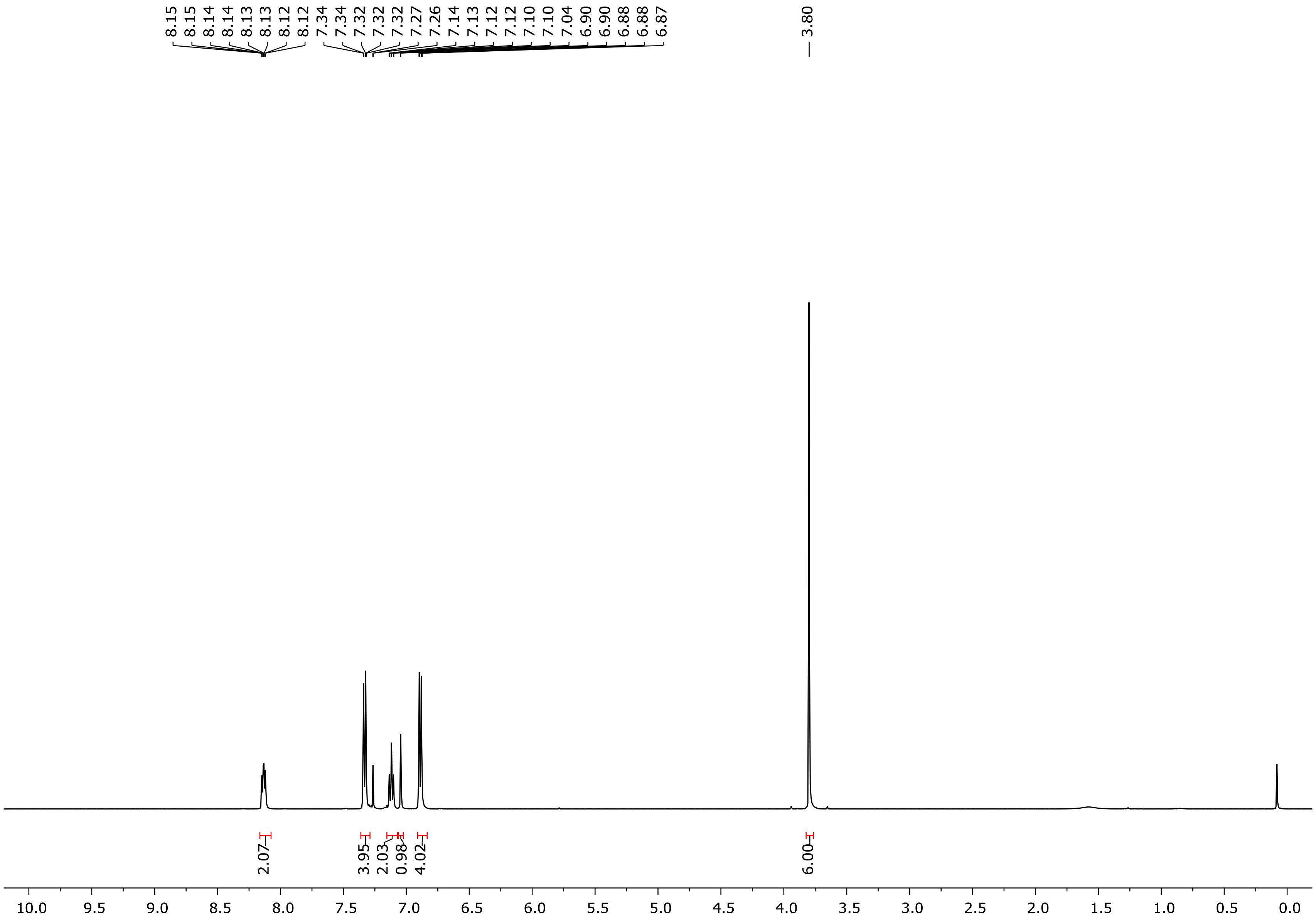

Figure S11: ^{13}**C NMR** (101 MHz, $CDCl_3$, 298 K) spectrum of **1d**.

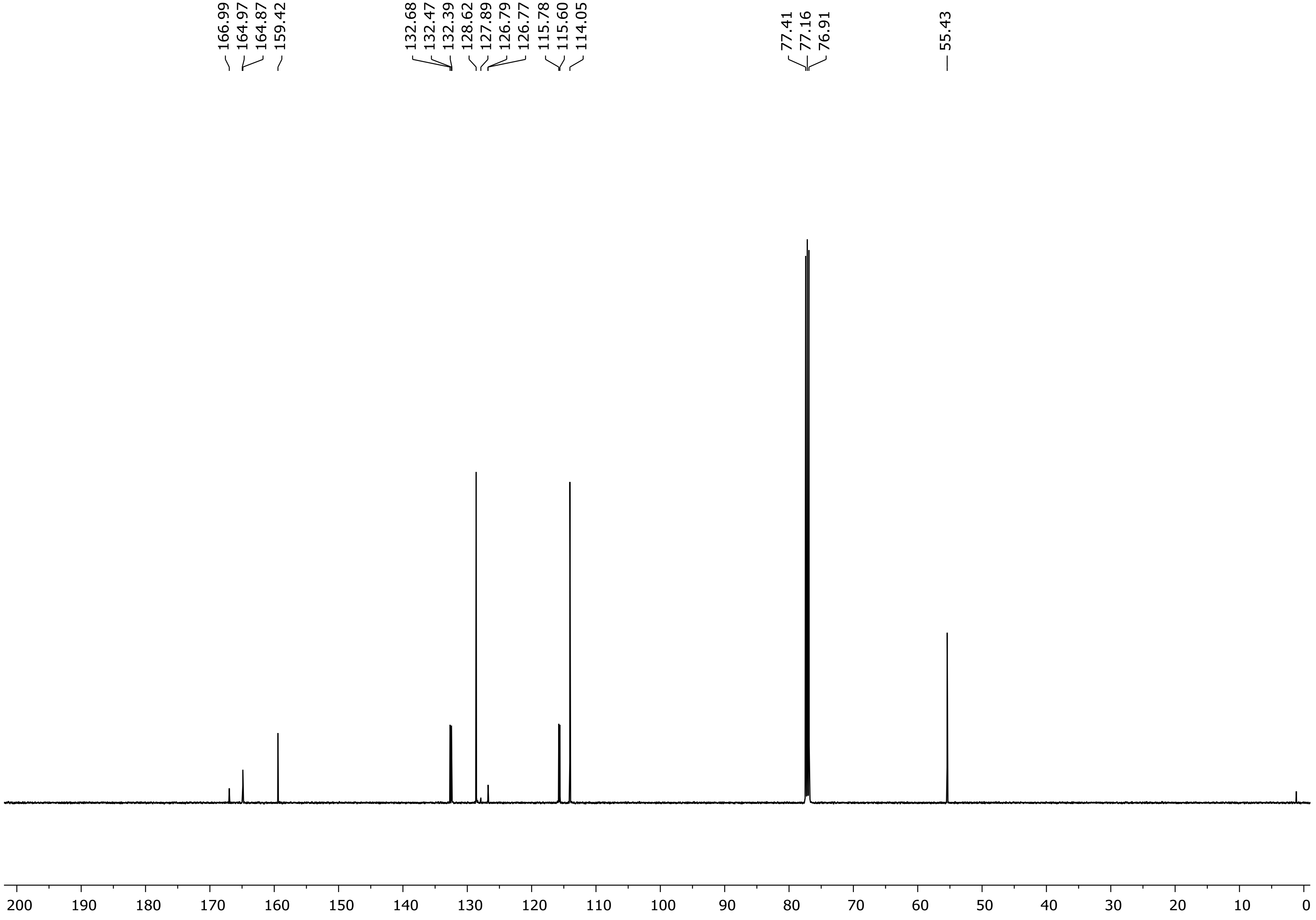

Figure S12: **^{19}F NMR** (376 MHz, $CDCl_3$, 298 K) spectrum of **1d**.

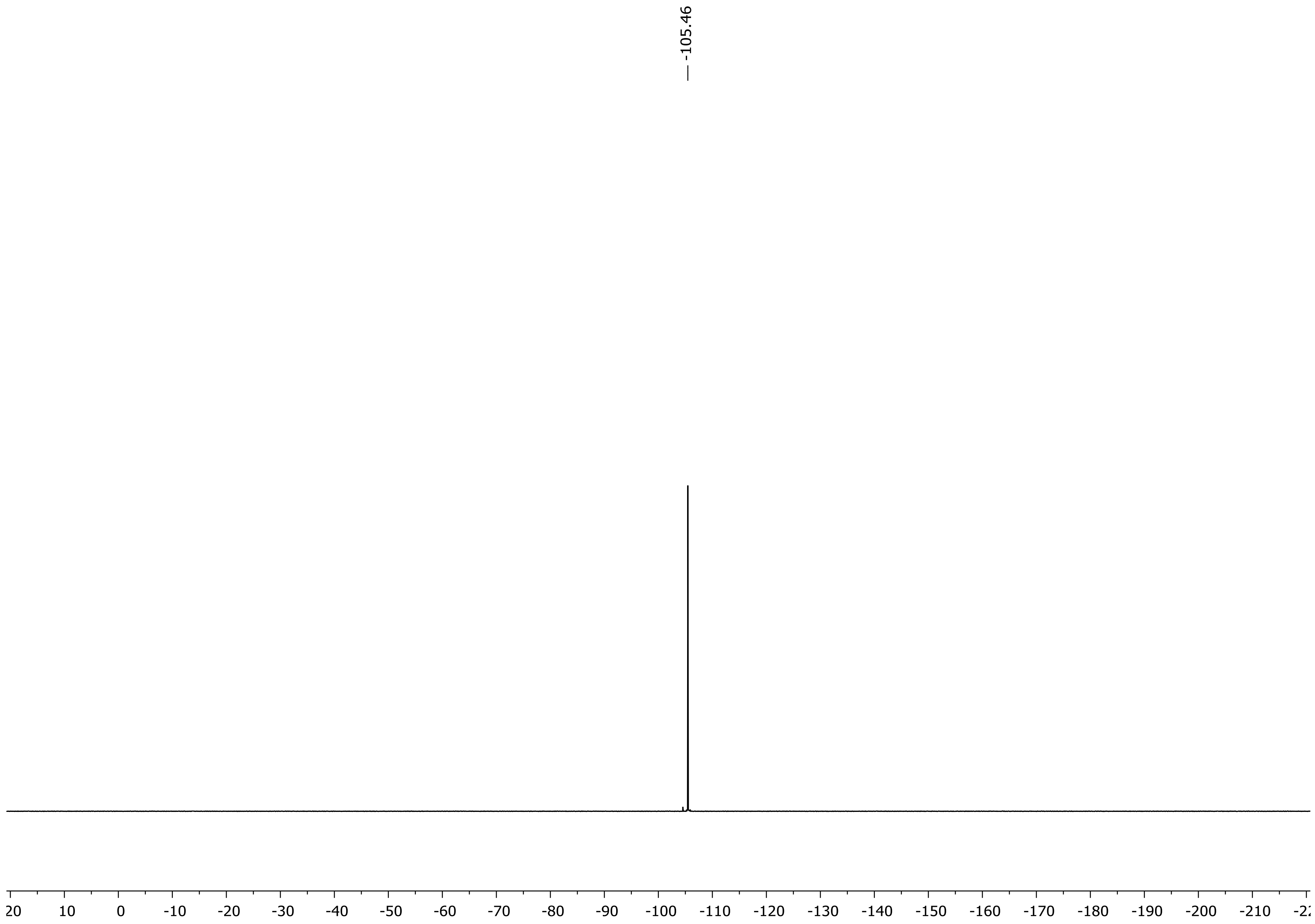

Figure S13: ^{1}**H NMR** (500 MHz, $CDCl_3$, 298 K) spectrum of **1e**.

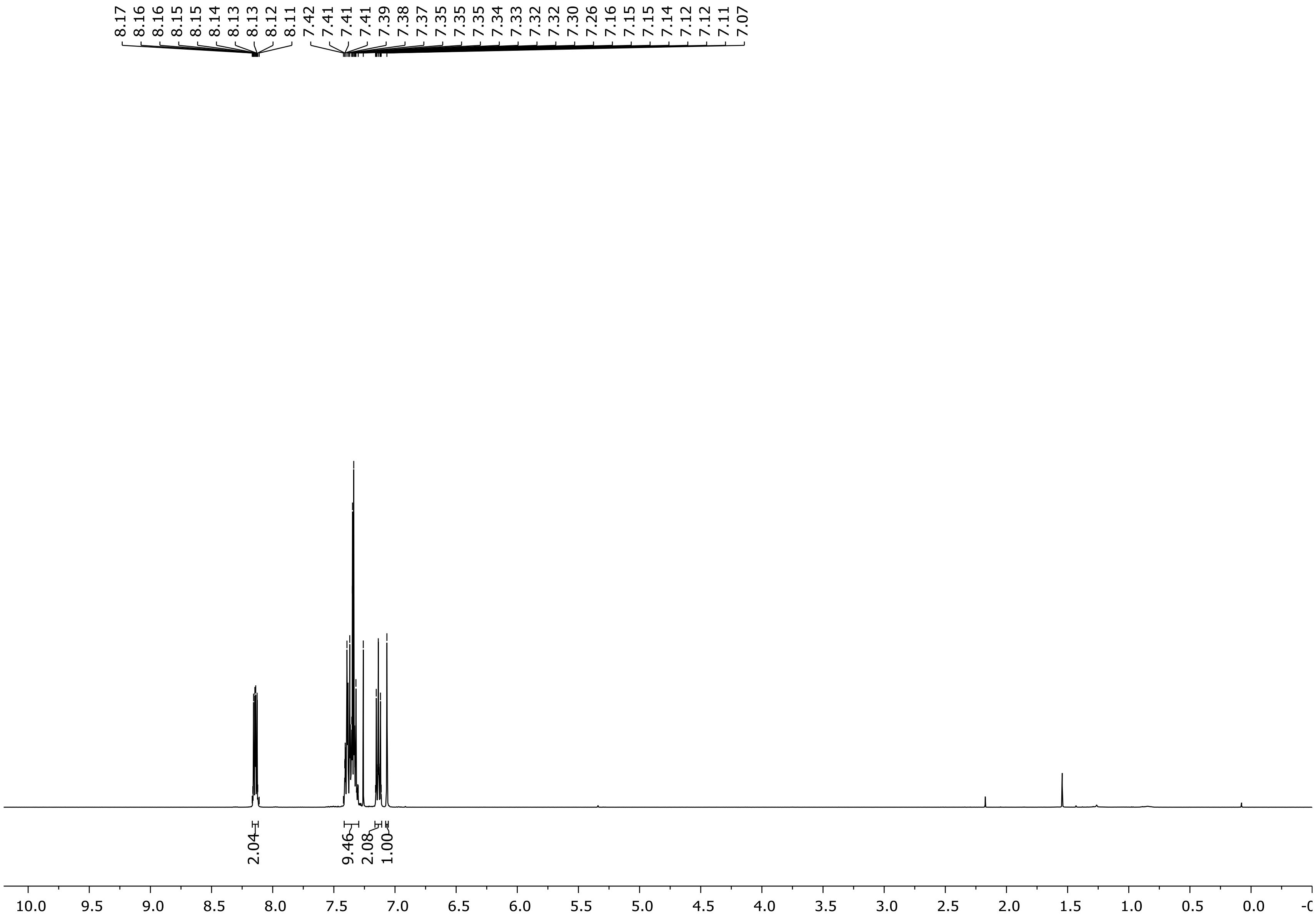

Figure S14: **^{13}C NMR** (126 MHz, $CDCl_3$, 298 K) spectrum of **1e**.

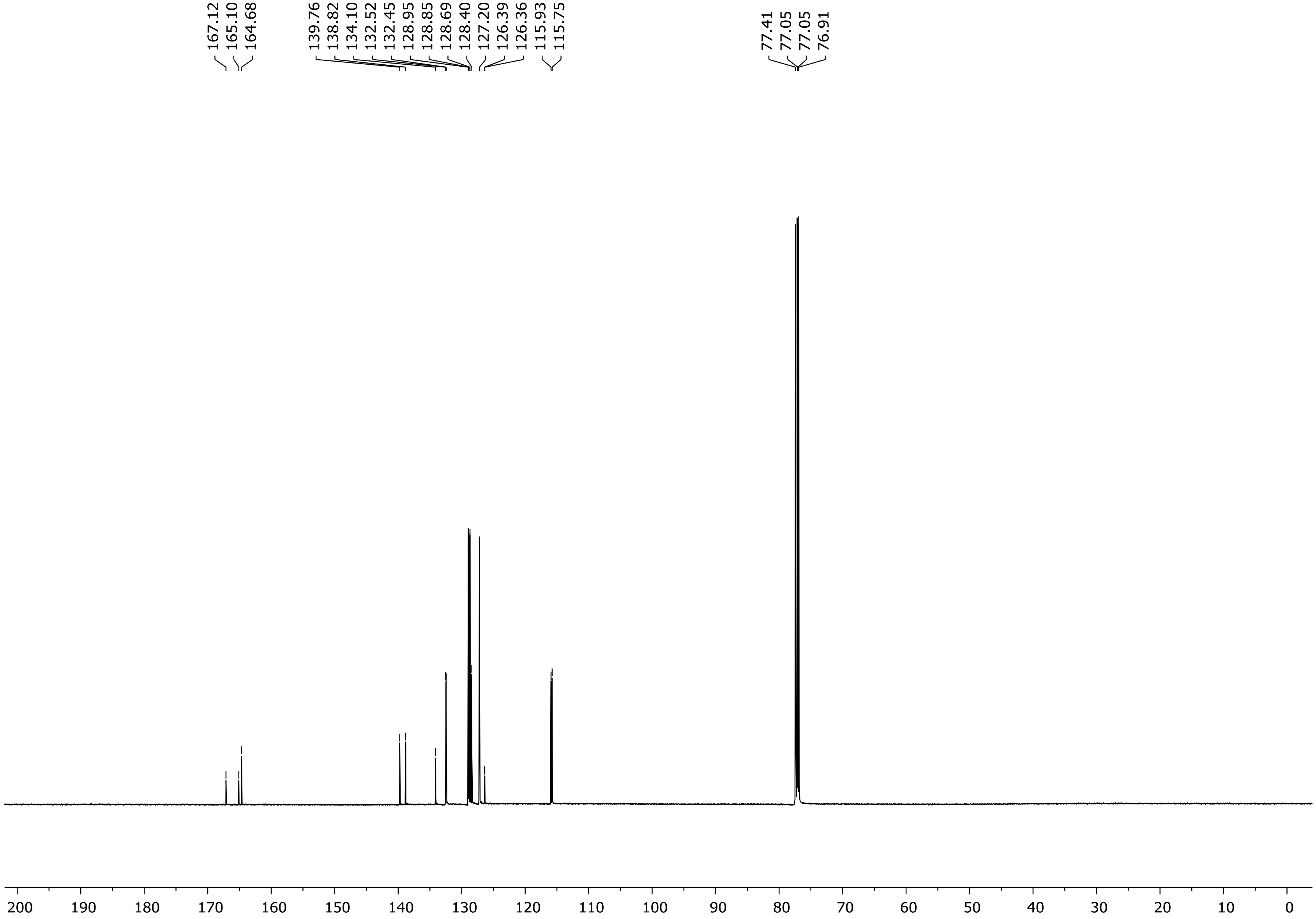

Figure S15: **^{19}F NMR** (471 MHz, $CDCl_3$, 298 K) spectrum of **1e**.

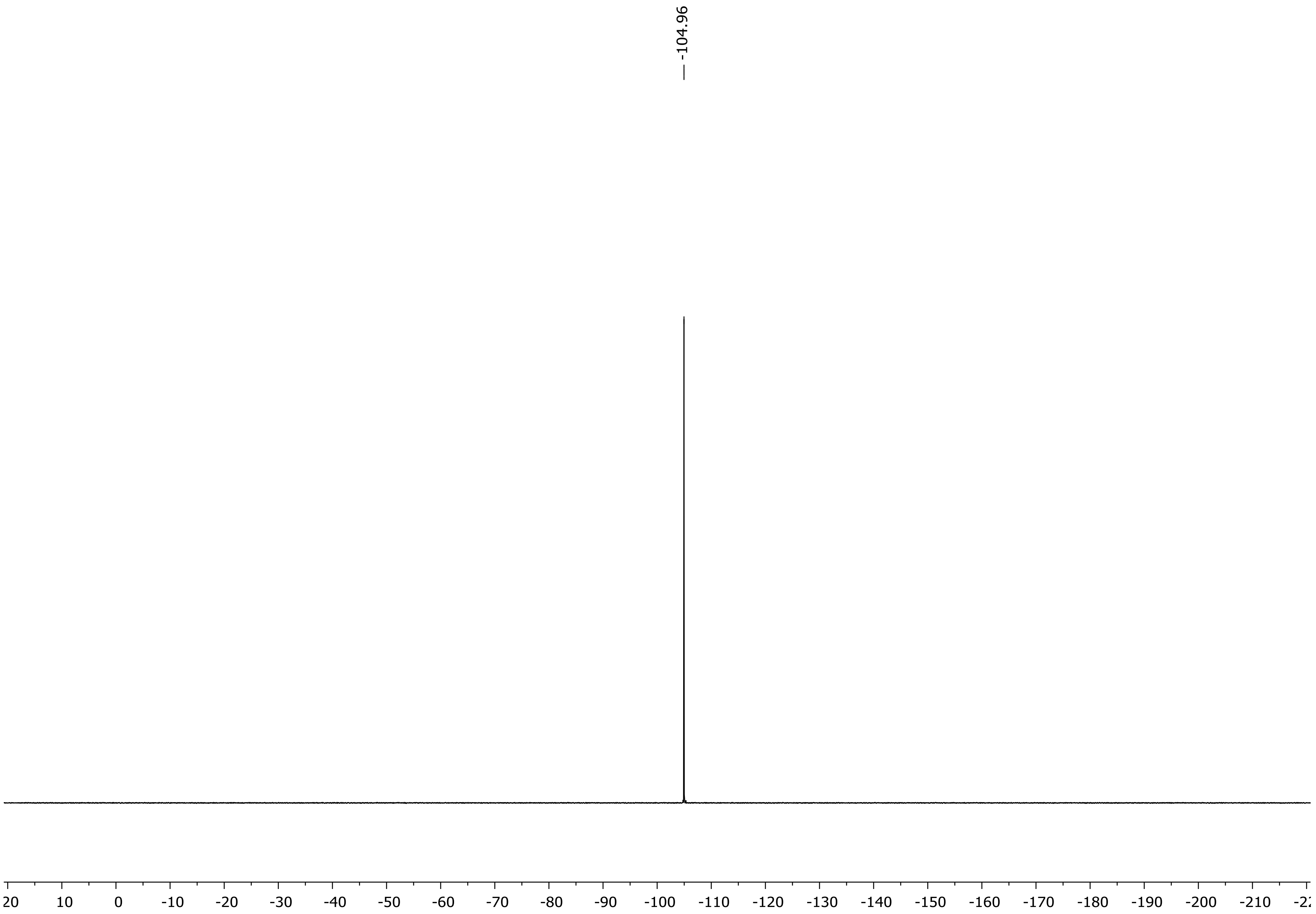

Figure S16: **^{1}H NMR** (500 MHz, $CDCl_3$, 298 K) spectrum of **1f**.

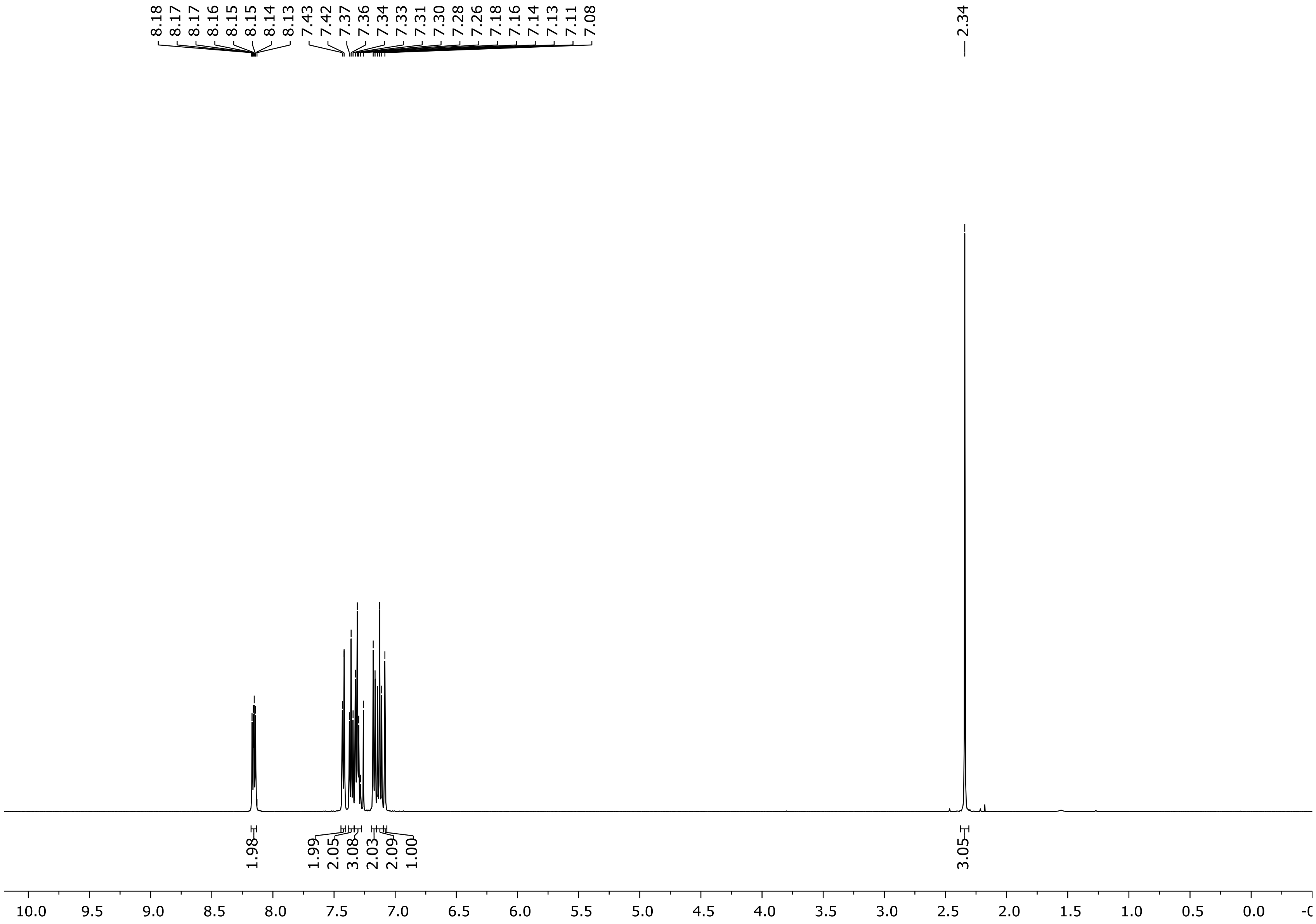

Figure S17: **^{13}C NMR** (126 MHz, $CDCl_3$, 298 K) spectrum of **1f**.

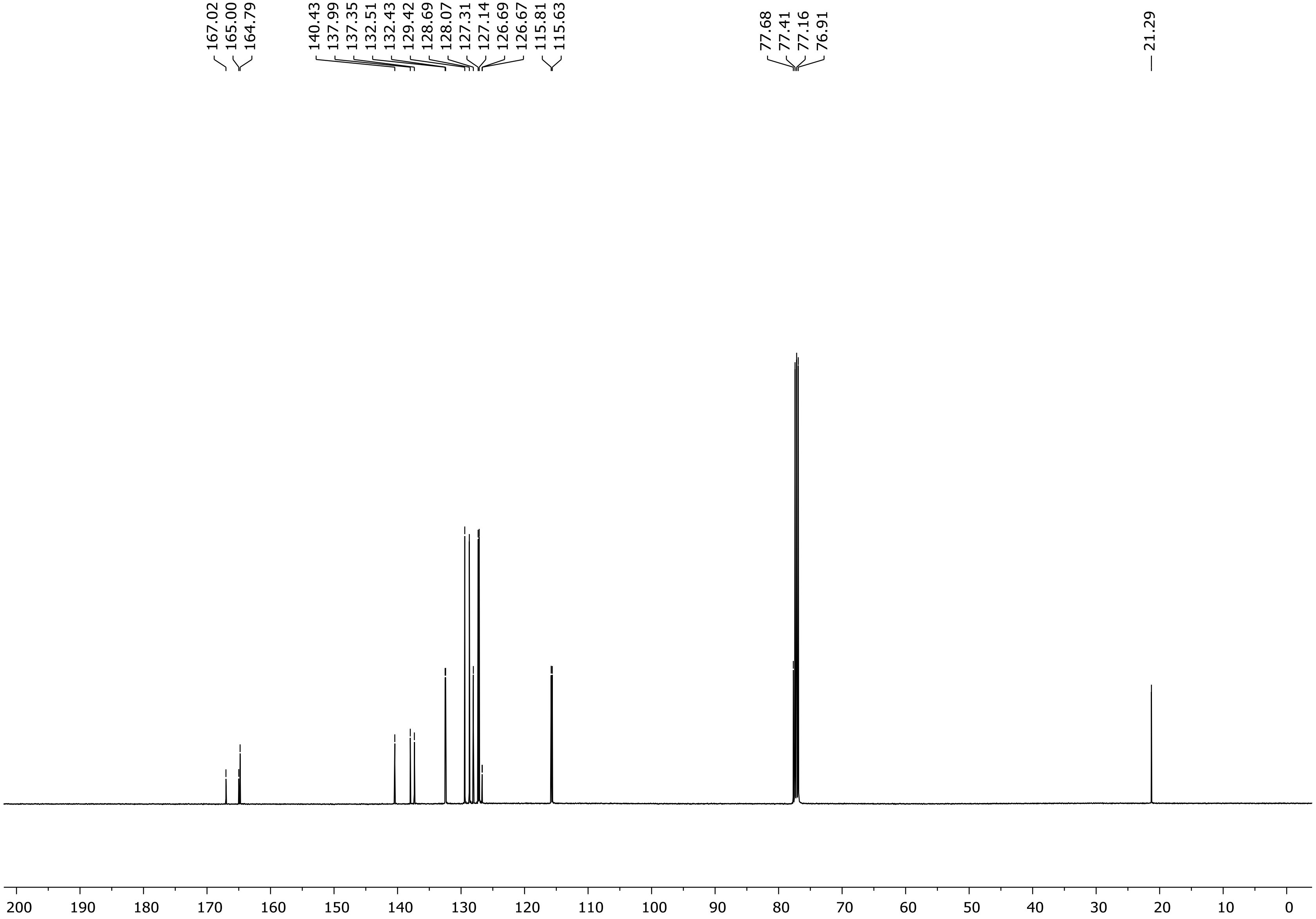

Figure S18: **^{19}F NMR** (471 MHz, $CDCl_3$, 298 K) spectrum of **1f**.

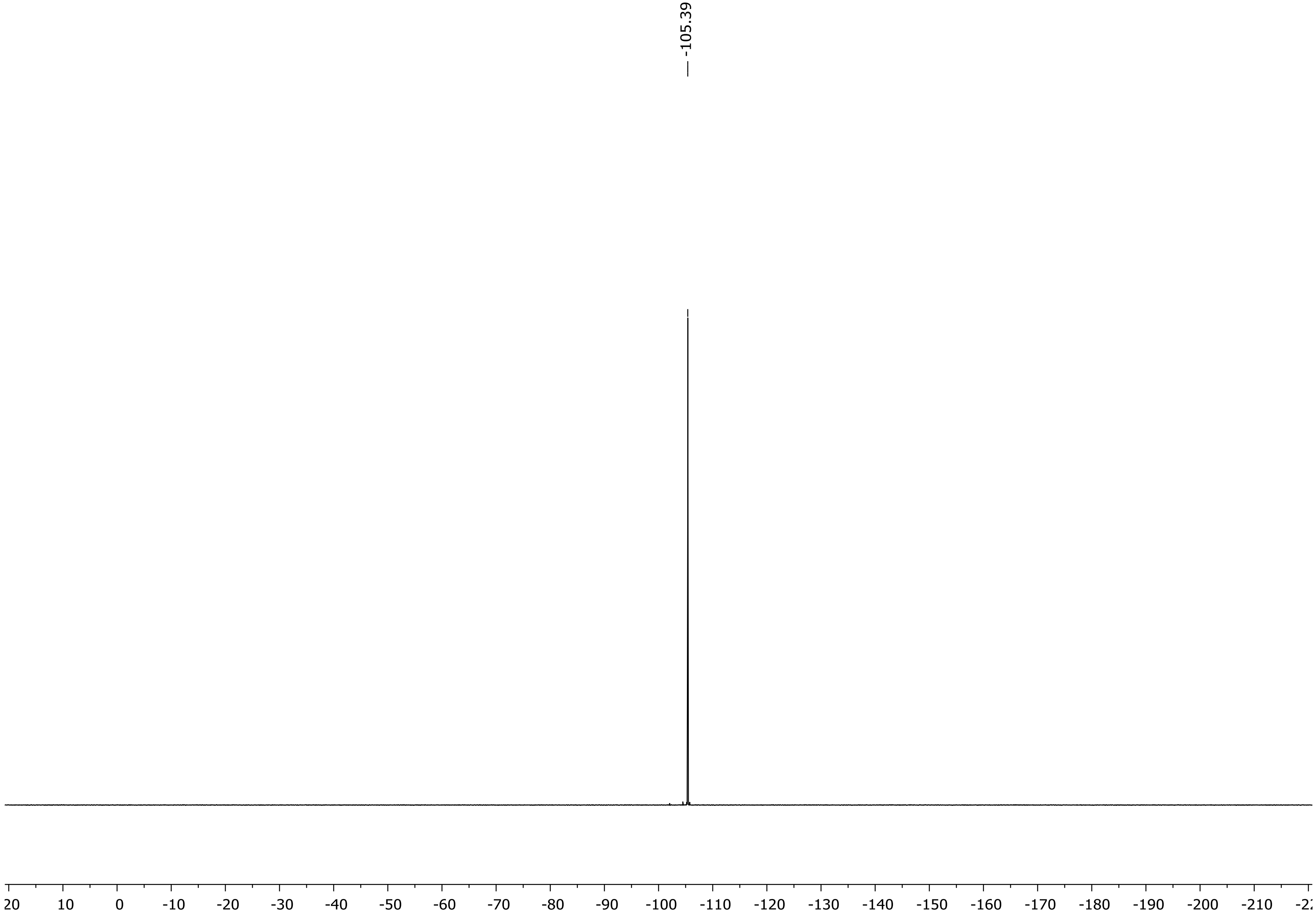

Figure S19: **^{1}H NMR** (500 MHz, $CDCl_3$, 298 K) spectrum of **1g**.

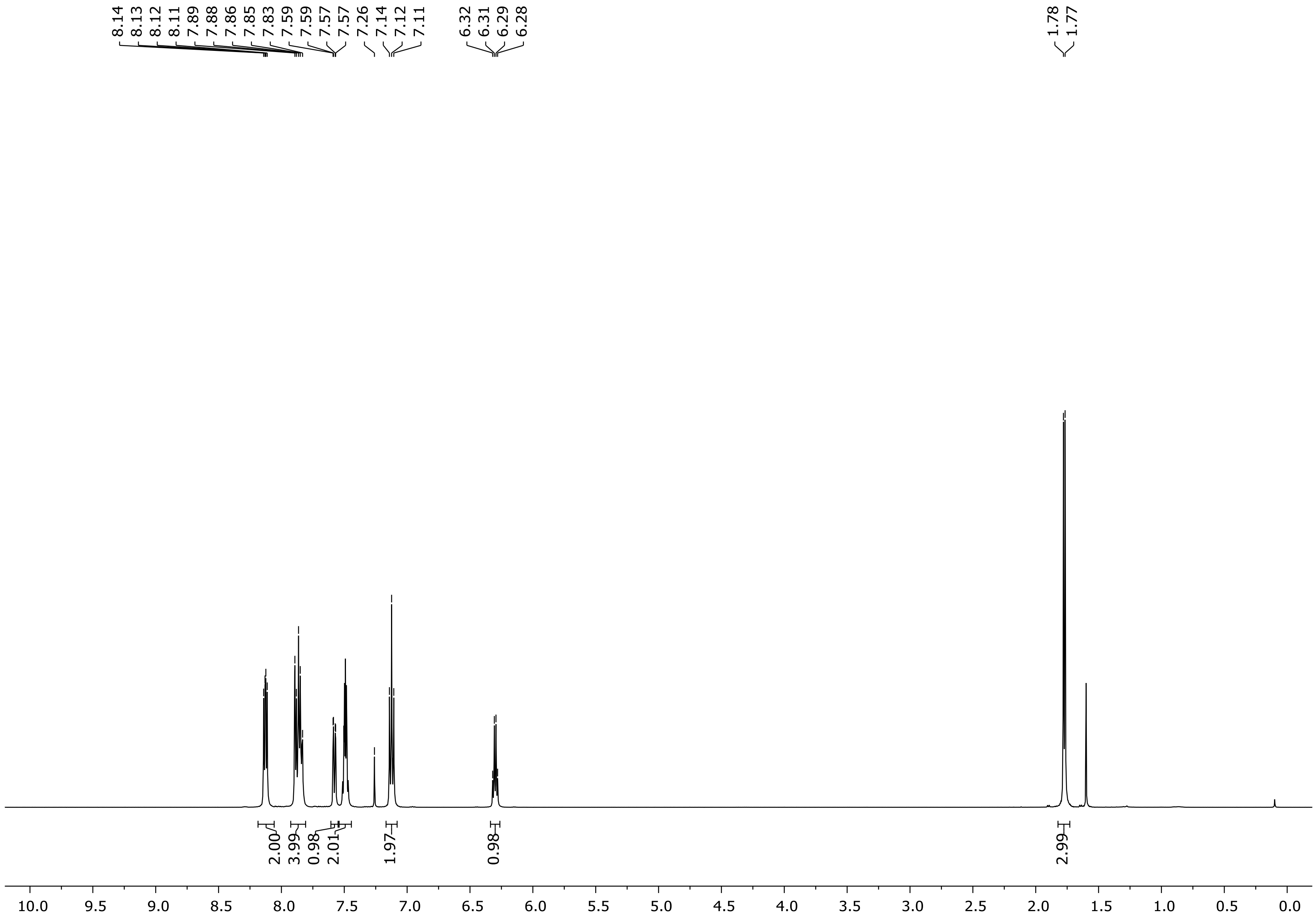

Figure S20: **^{13}C NMR** (126 MHz, $CDCl_3$, 298 K) spectrum of **1g**.

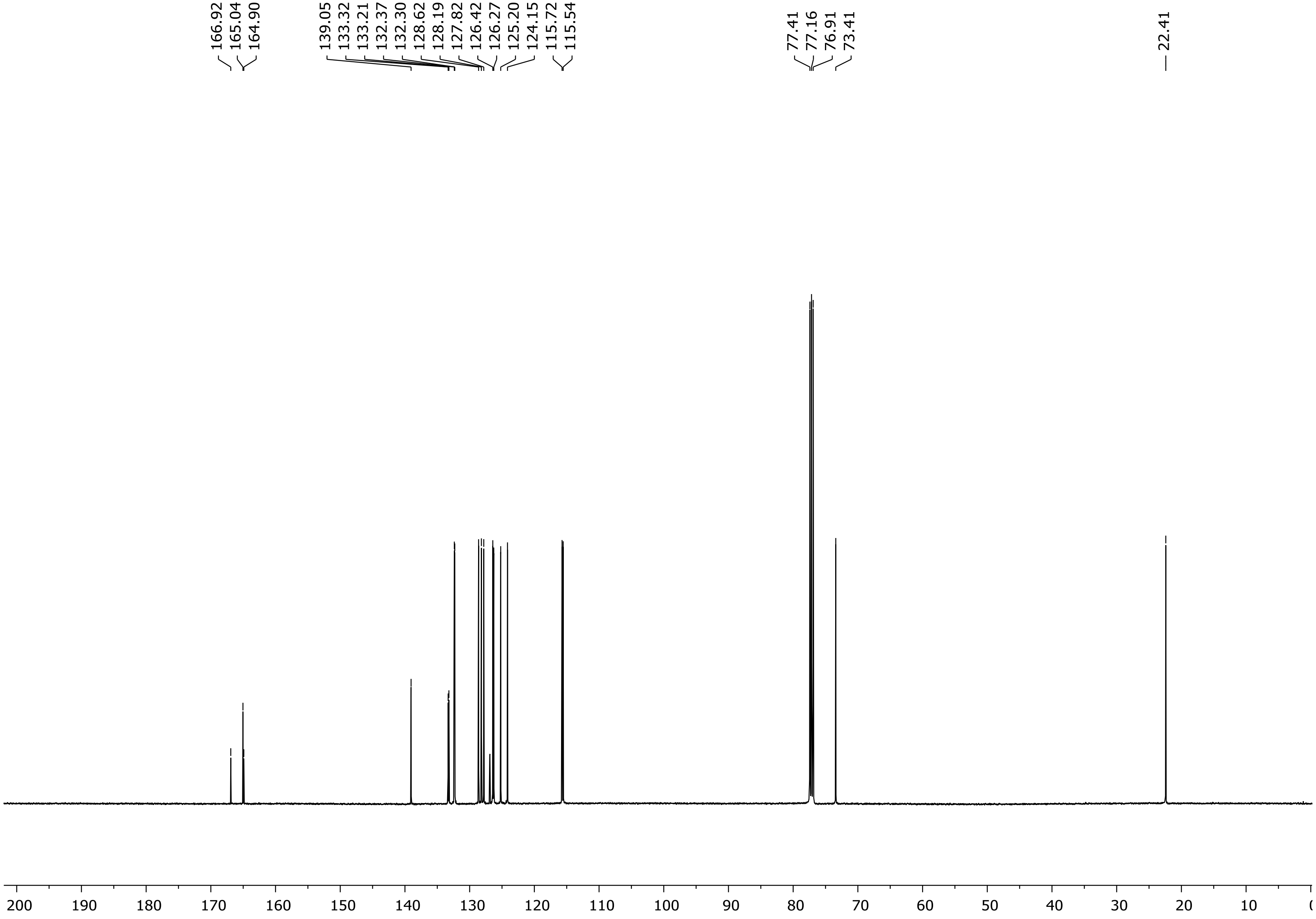

Figure S21: **^{19}F NMR** (471 MHz, $CDCl_3$, 298 K) spectrum of **1g**.

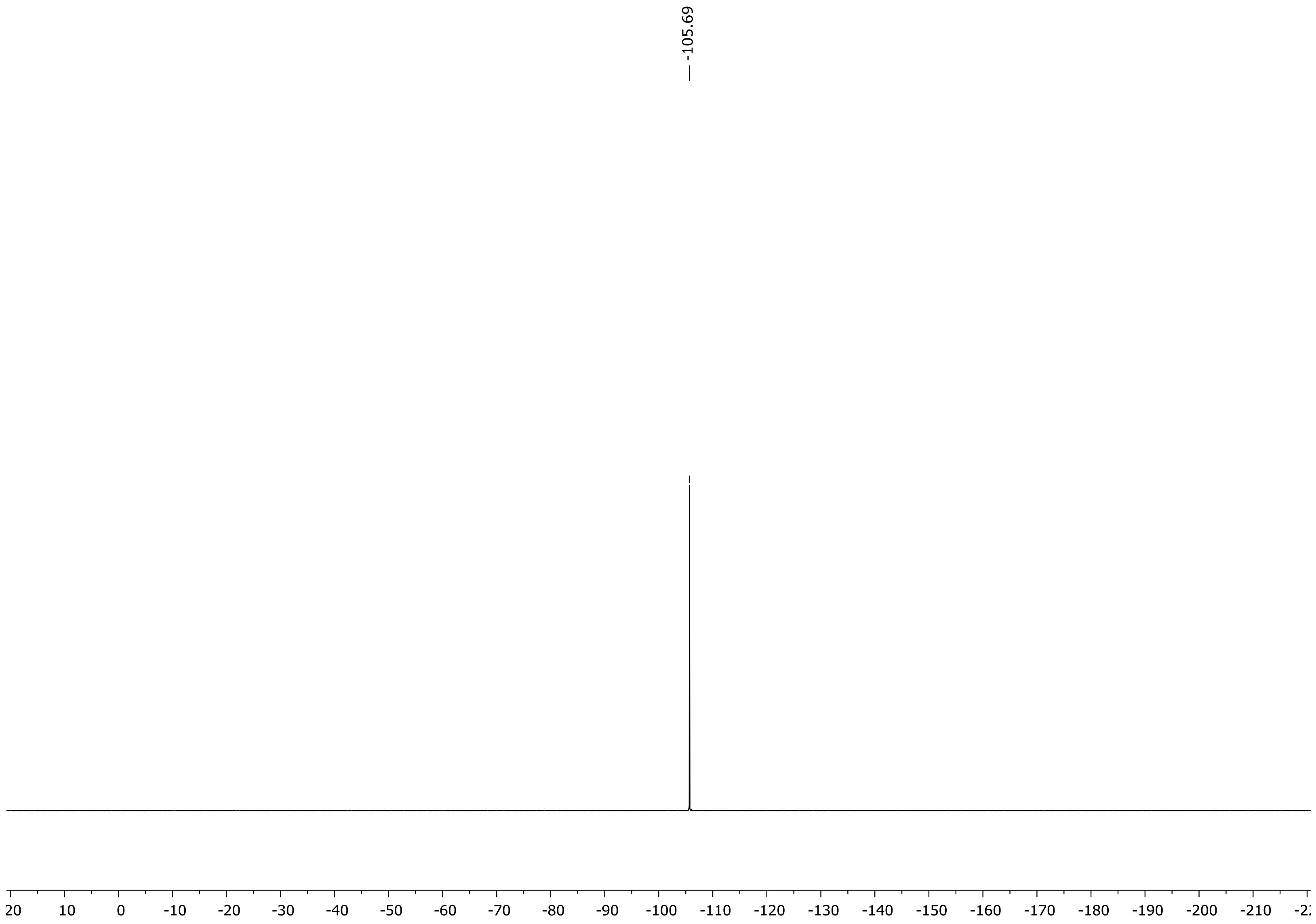

Figure S22: **^{1}H NMR** (500 MHz, $CDCl_3$, 298 K) spectrum of **1h**.

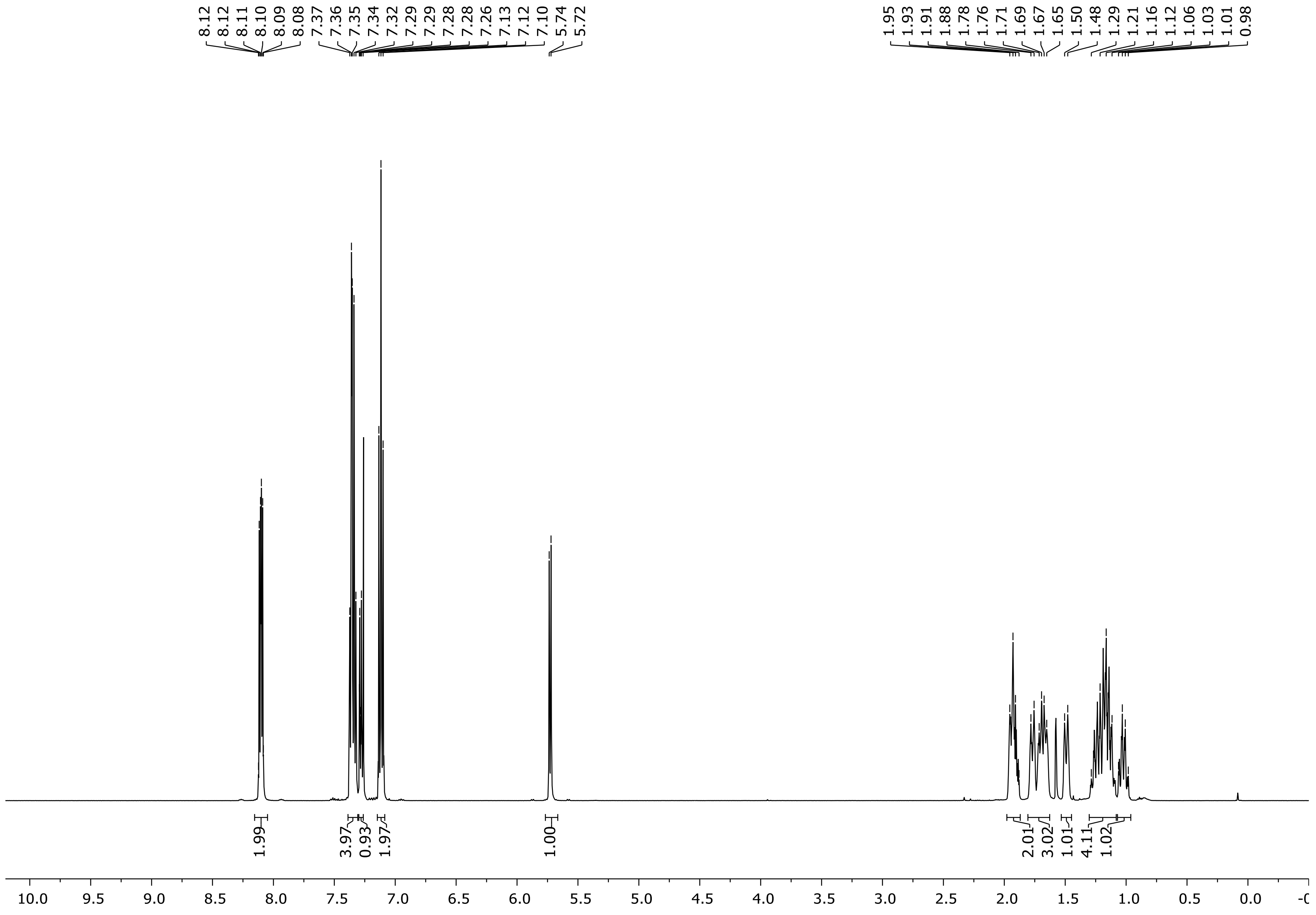

Figure S23: **^{13}C NMR** (126 MHz, $CDCl_3$, 298 K) spectrum of **1h**.

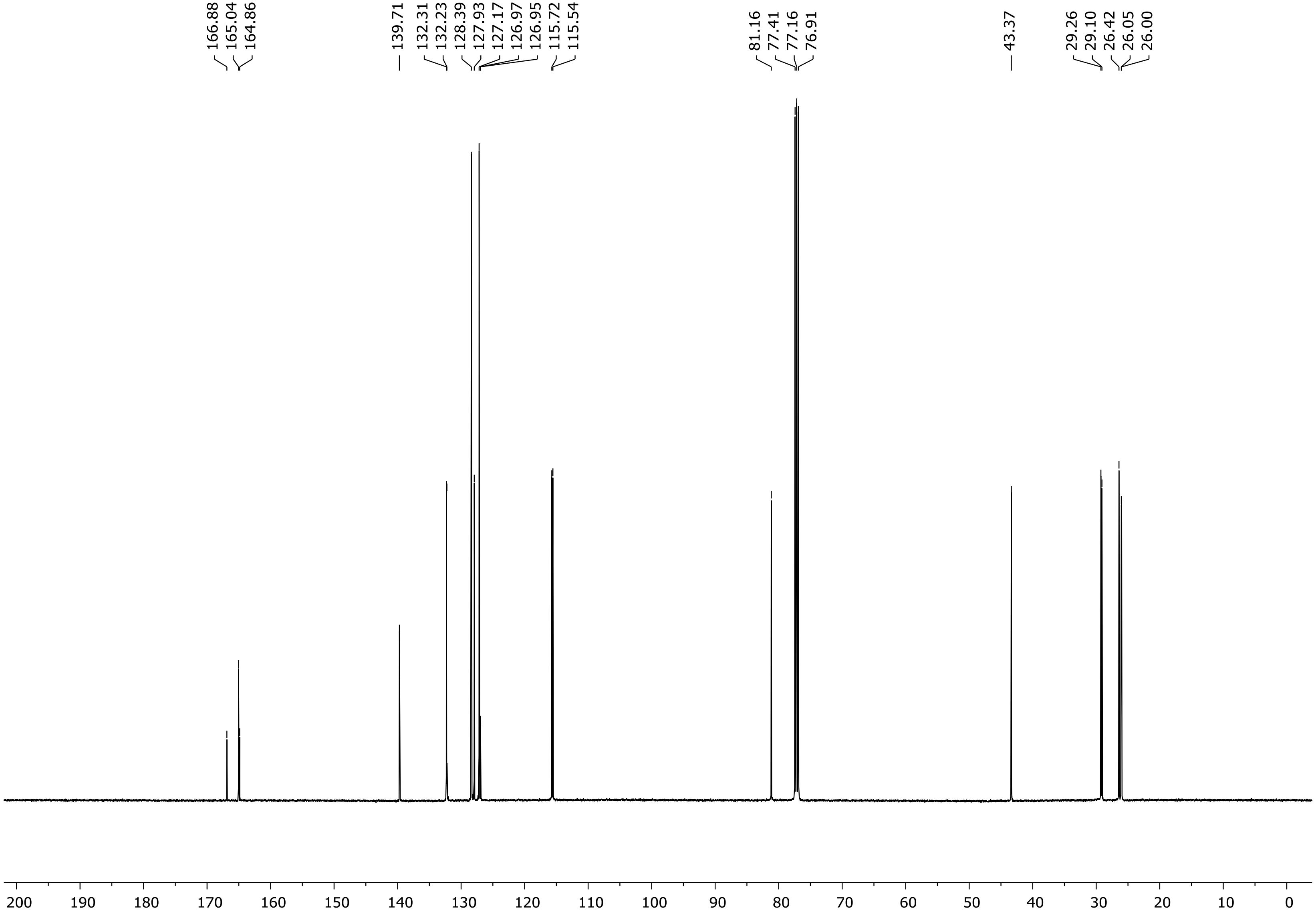

Figure S24: **^{19}F NMR** (471 MHz, $CDCl_3$, 298 K) spectrum of **1h**.

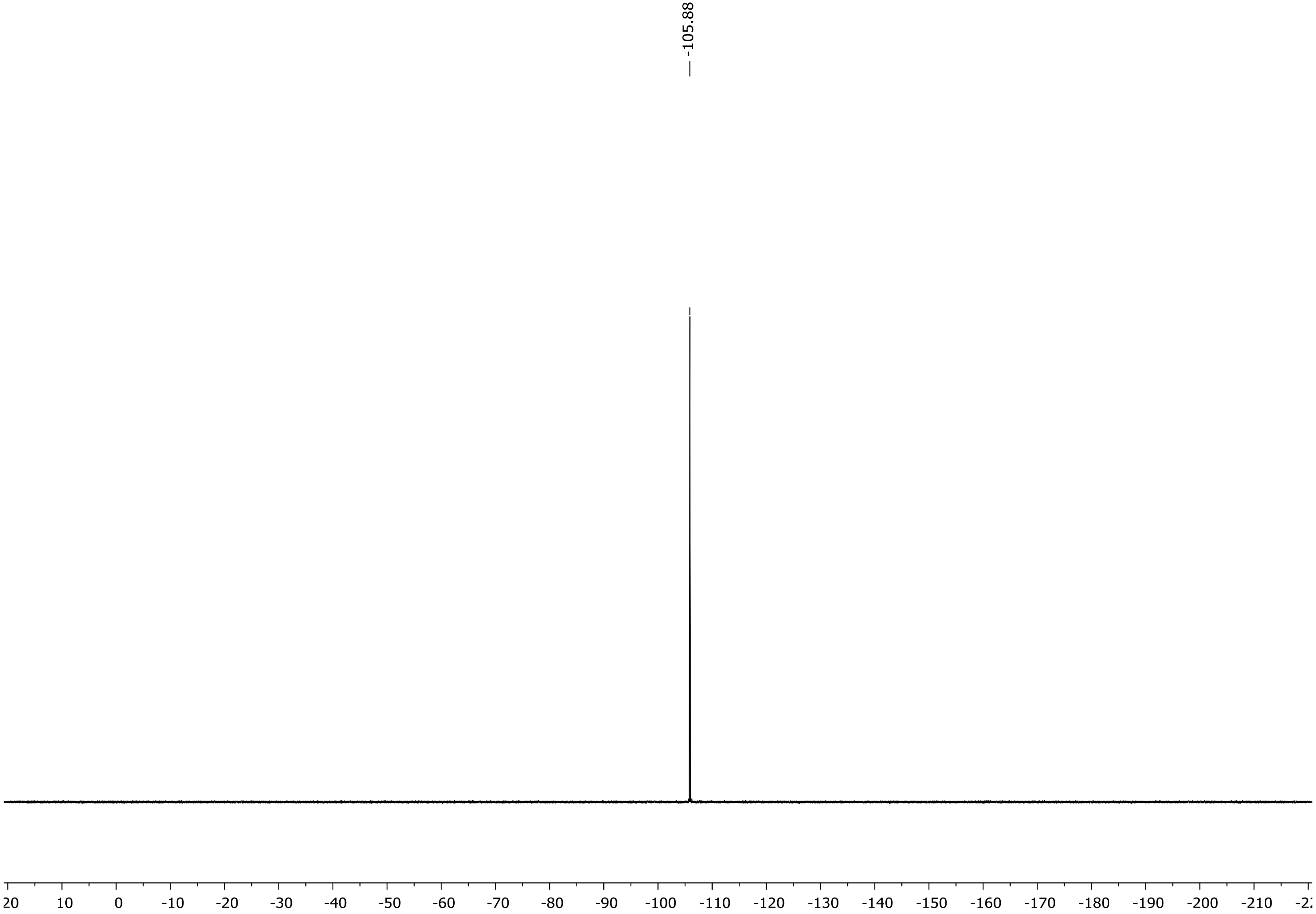

Figure S25: **^{1}H NMR** (400 MHz, $CDCl_3$, 298 K) spectrum of **1i**.

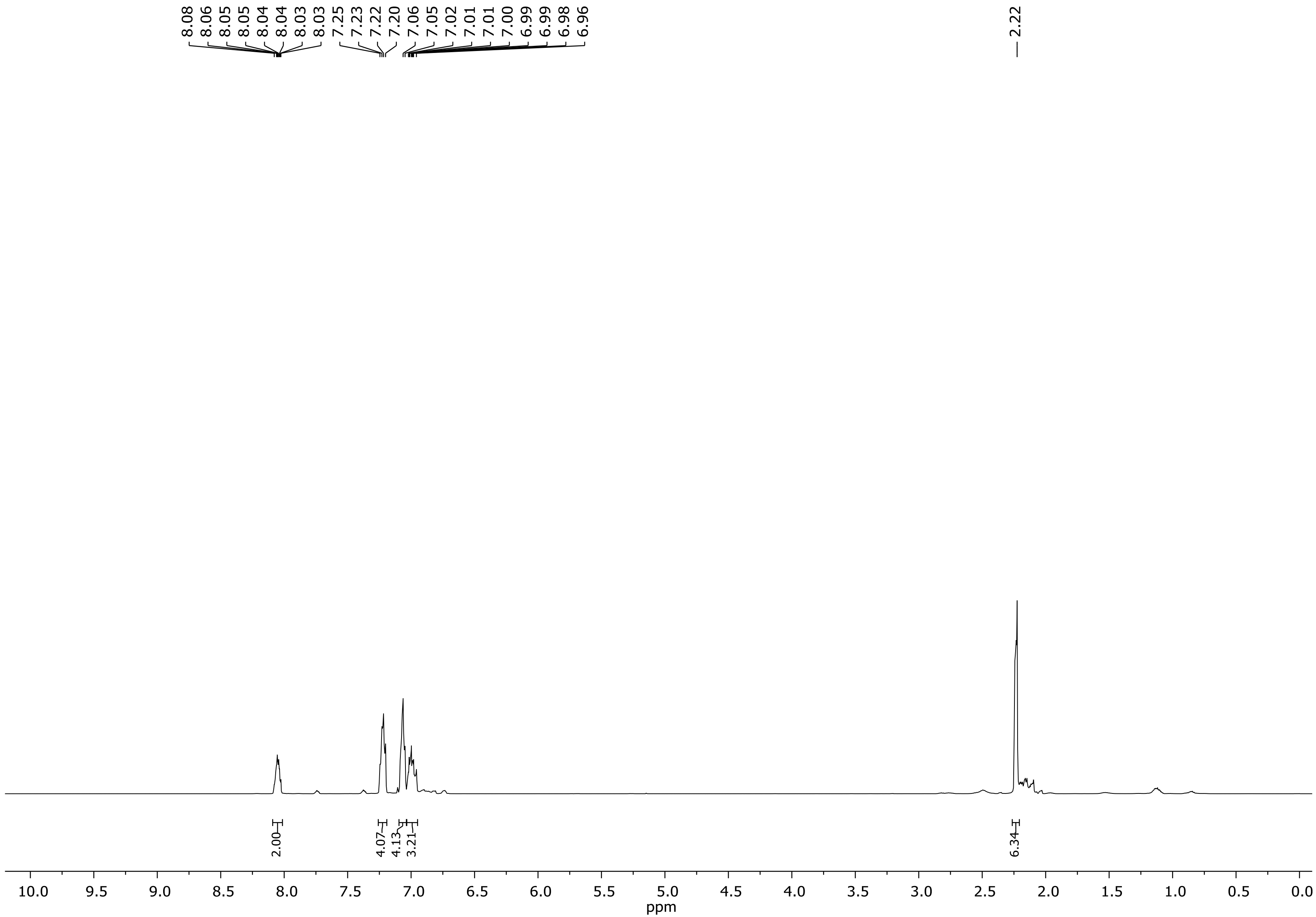

Figure S26: **^{13}C NMR** (101 MHz, $CDCl_3$, 298 K) spectrum of **1i**.

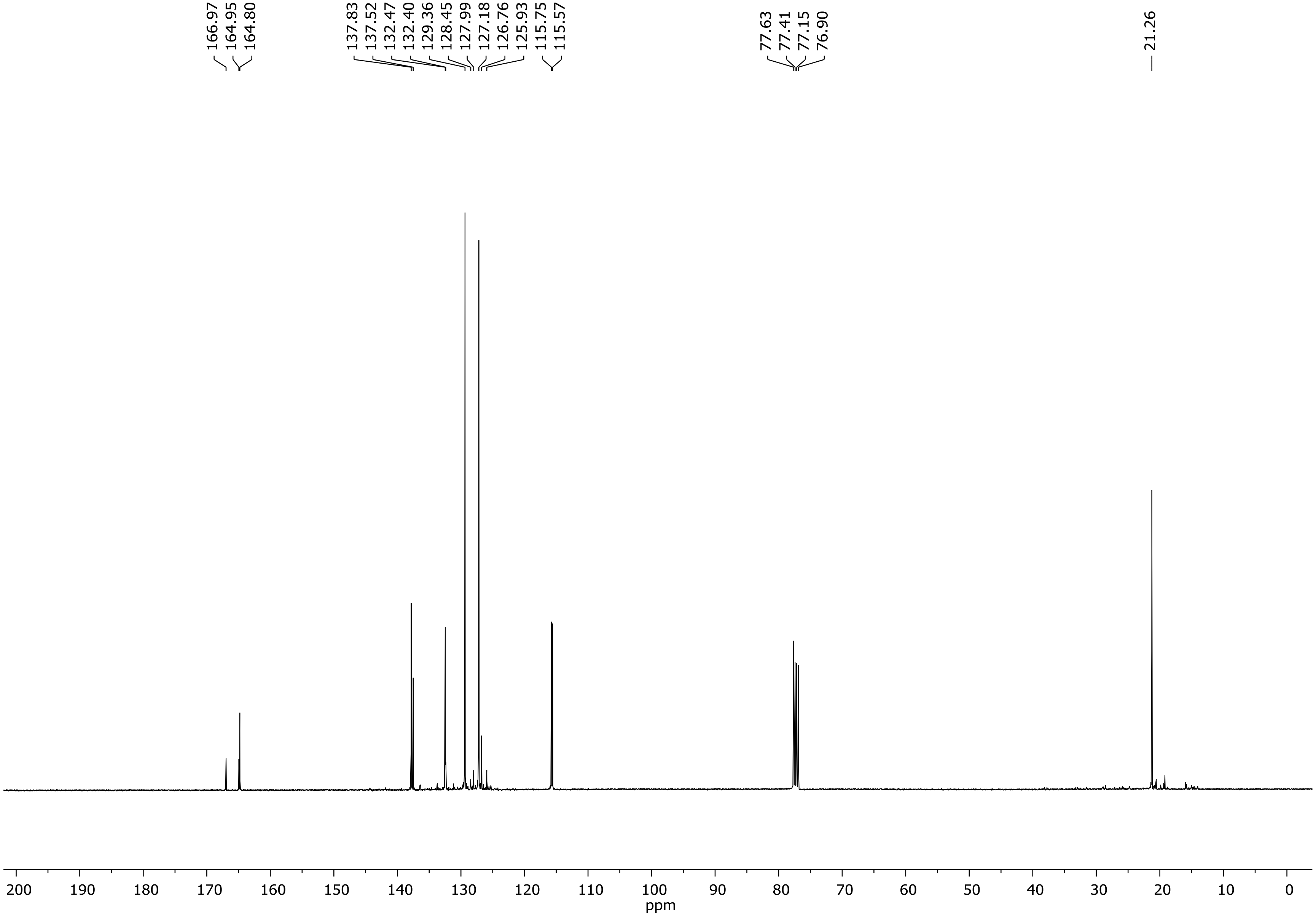

Figure S27: **^{19}F NMR** (376 MHz, $CDCl_3$, 298 K) spectrum of **1i**.

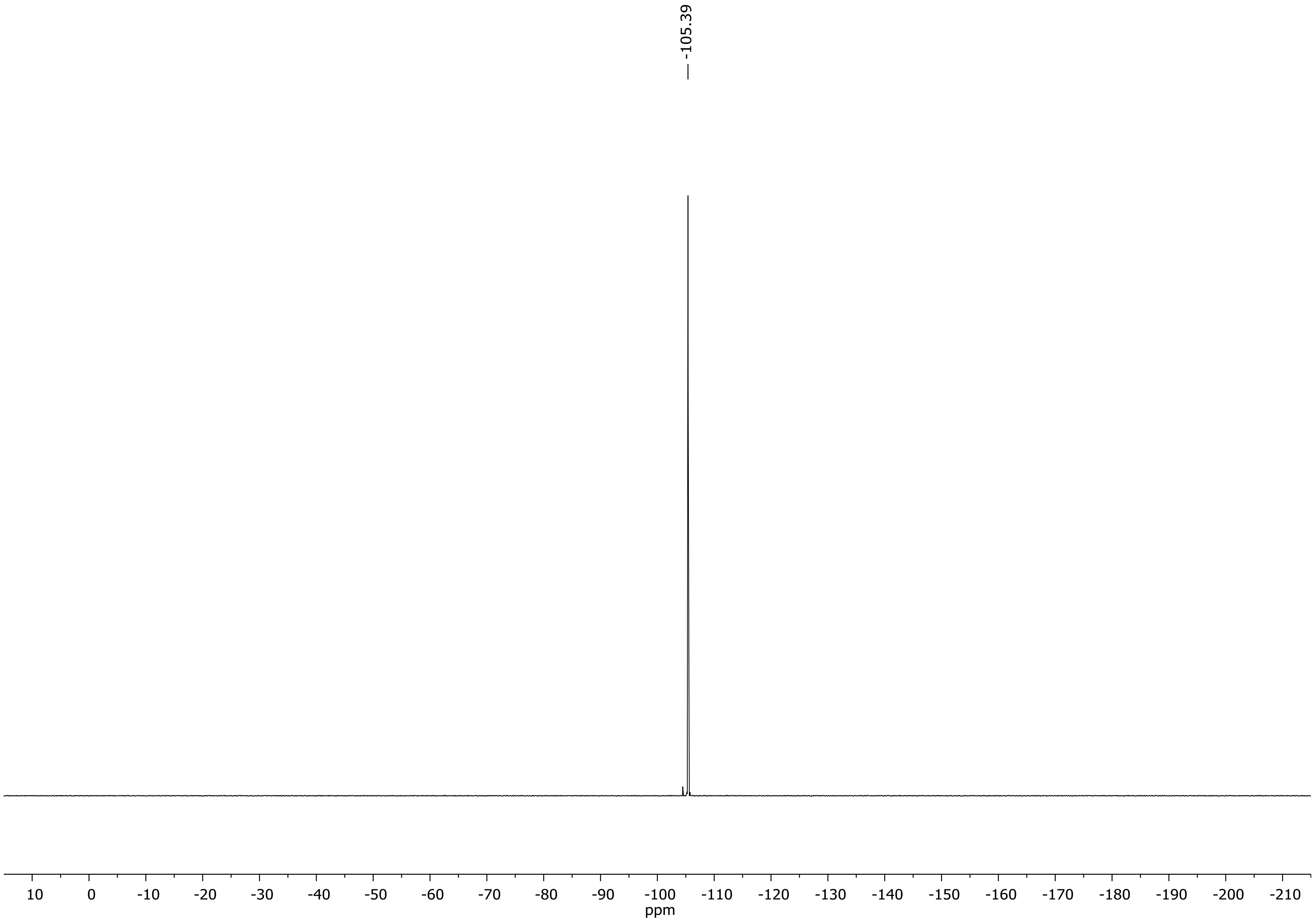

Figure S28: **^{1}H NMR** (500 MHz, $CDCl_3$, 298 K) spectrum of compound **1j**.

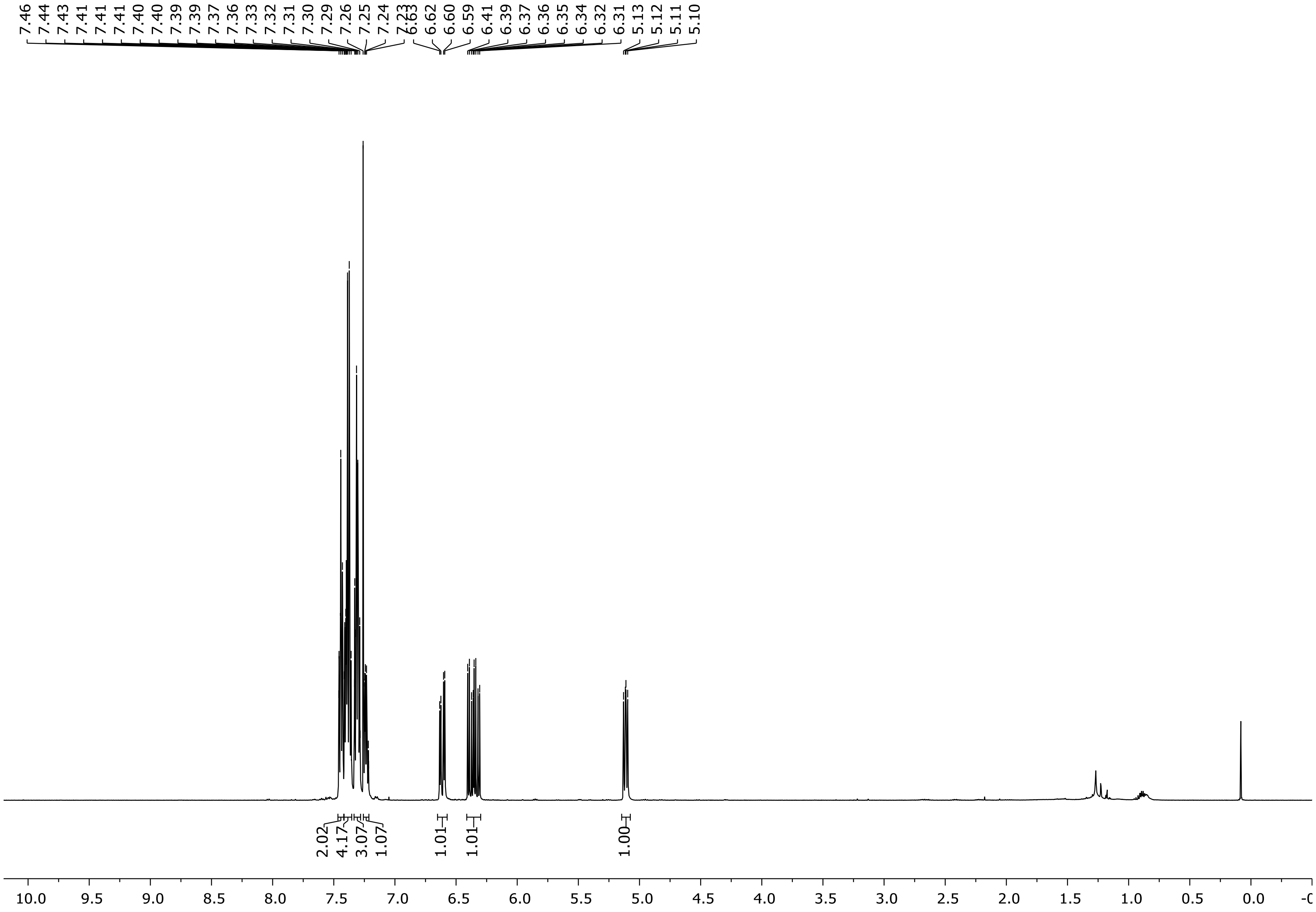

Figure S29: **^{13}C NMR** (126 MHz, $CDCl_3$, 298 K) spectrum of compound **1j**.

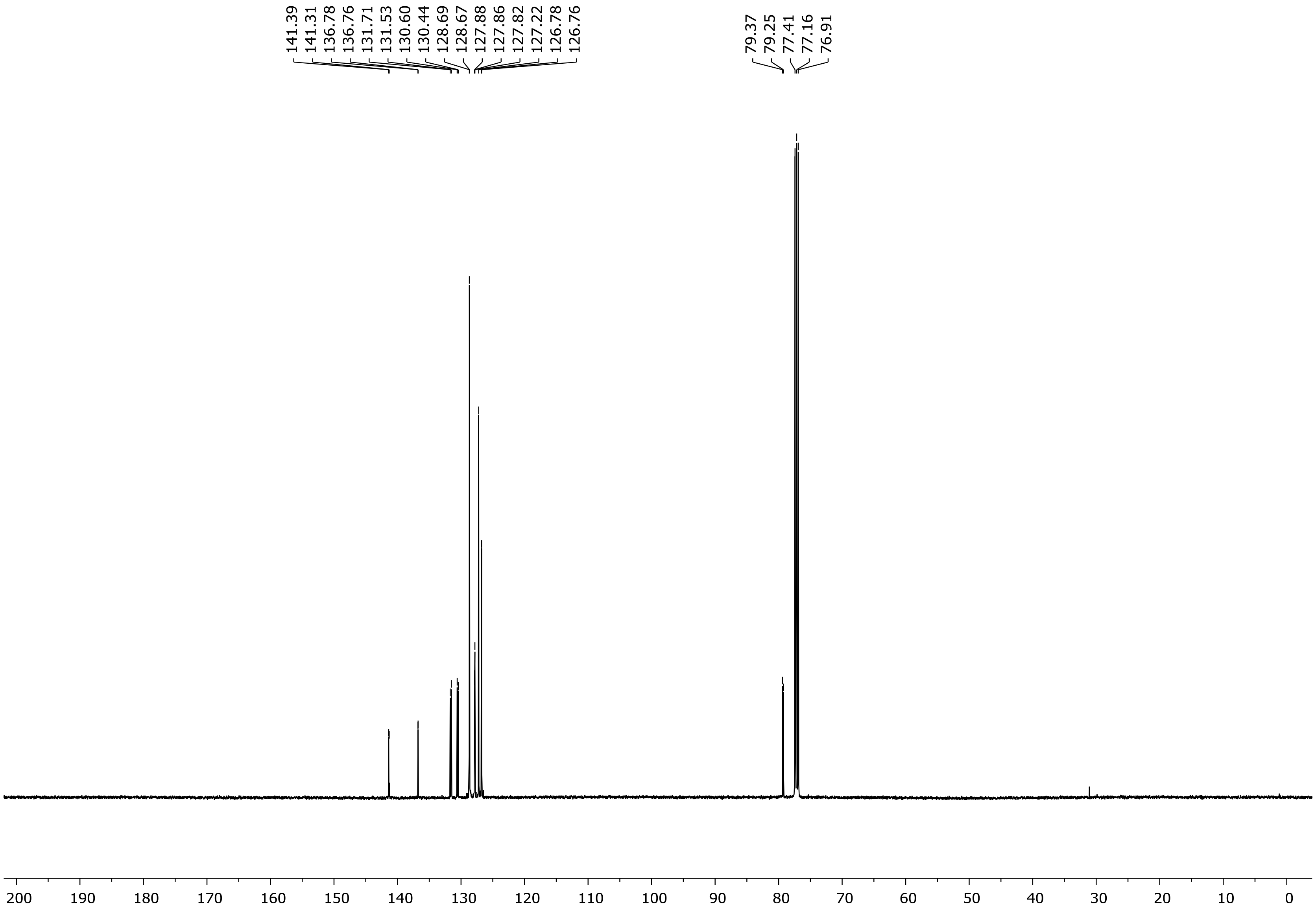

Figure S30: **^{19}F NMR** (471 MHz, $CDCl_3$, 298 K) spectrum of compound **1j**.

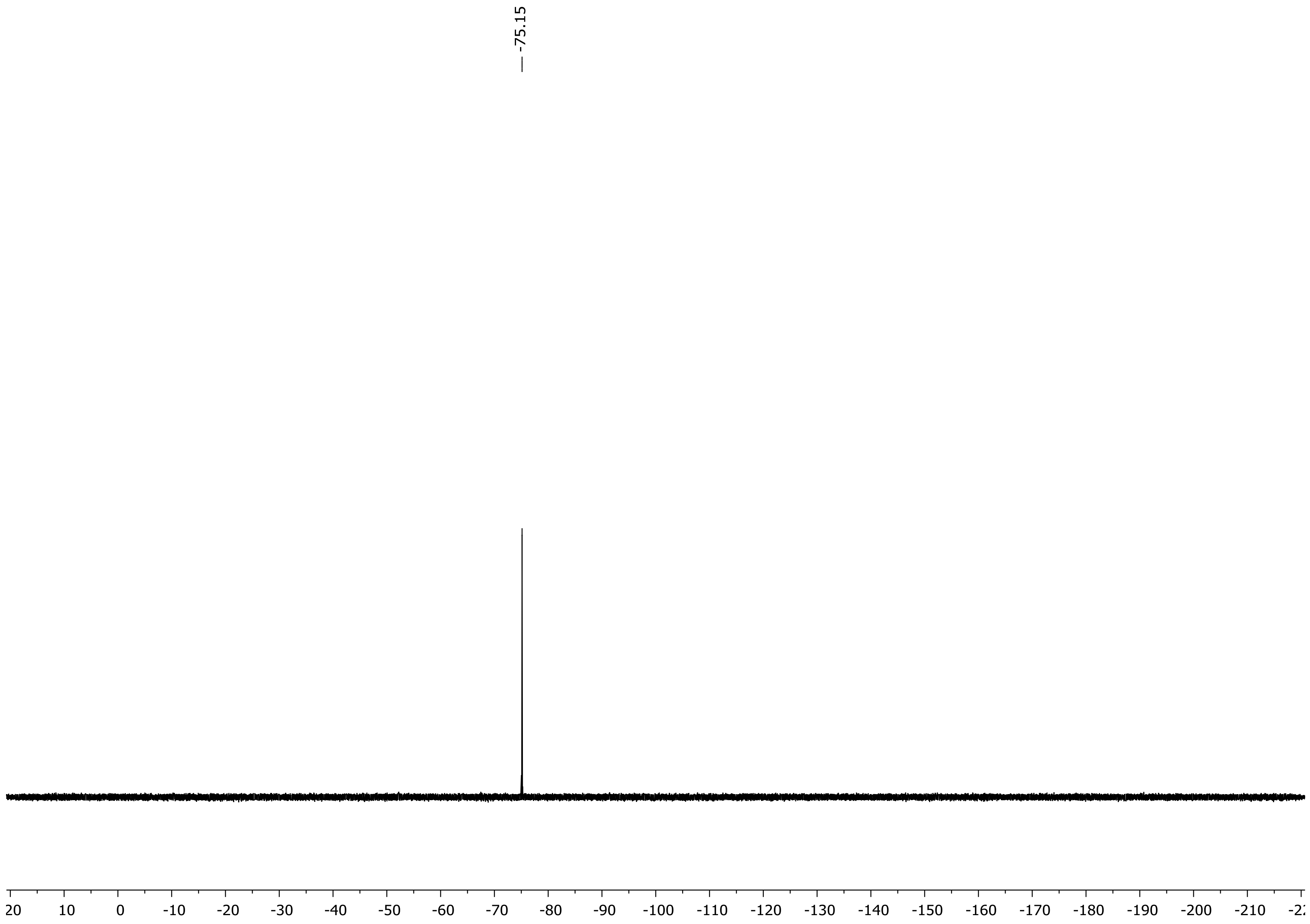

Figure S31: **^{1}H NMR** (500 MHz, $CDCl_3$, 298 K) spectrum of **1k**.

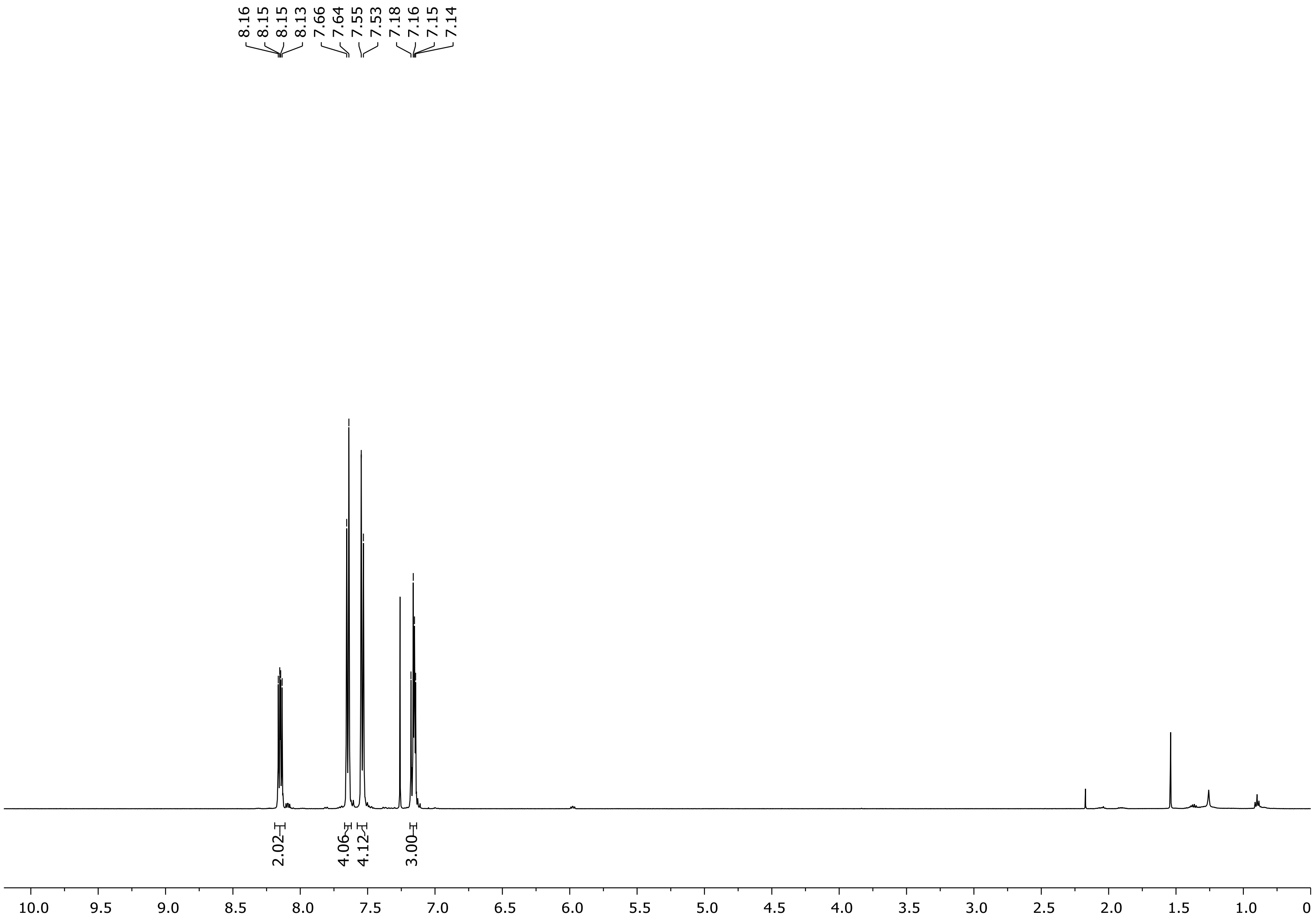

Figure S32: **^{13}C NMR** (126 MHz, $CDCl_3$, 298 K) spectrum of **1k.**

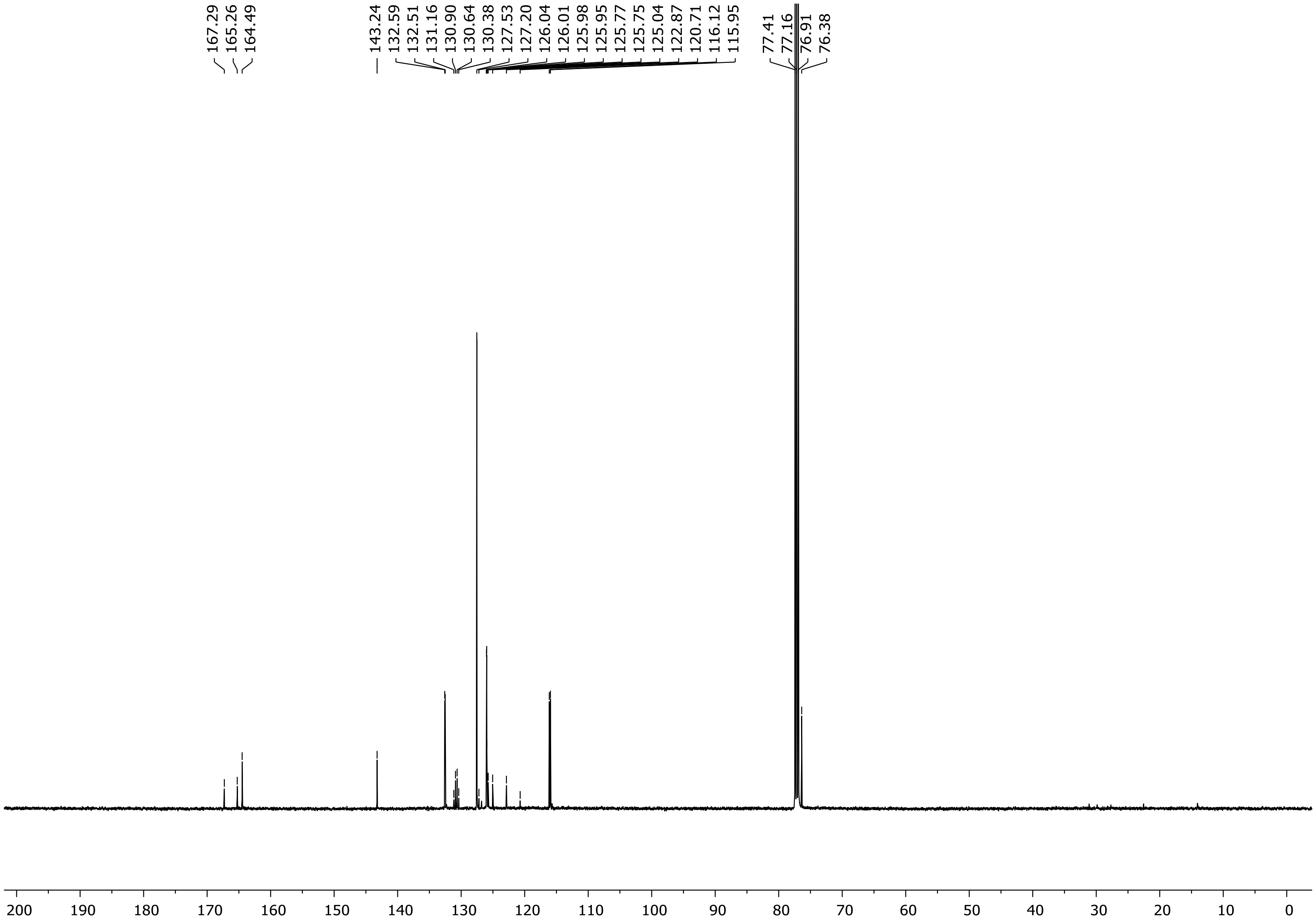

Figure S33: ^{19}**F NMR** (471 MHz, $CDCl_3$, 298 K) spectrum of **1k**.

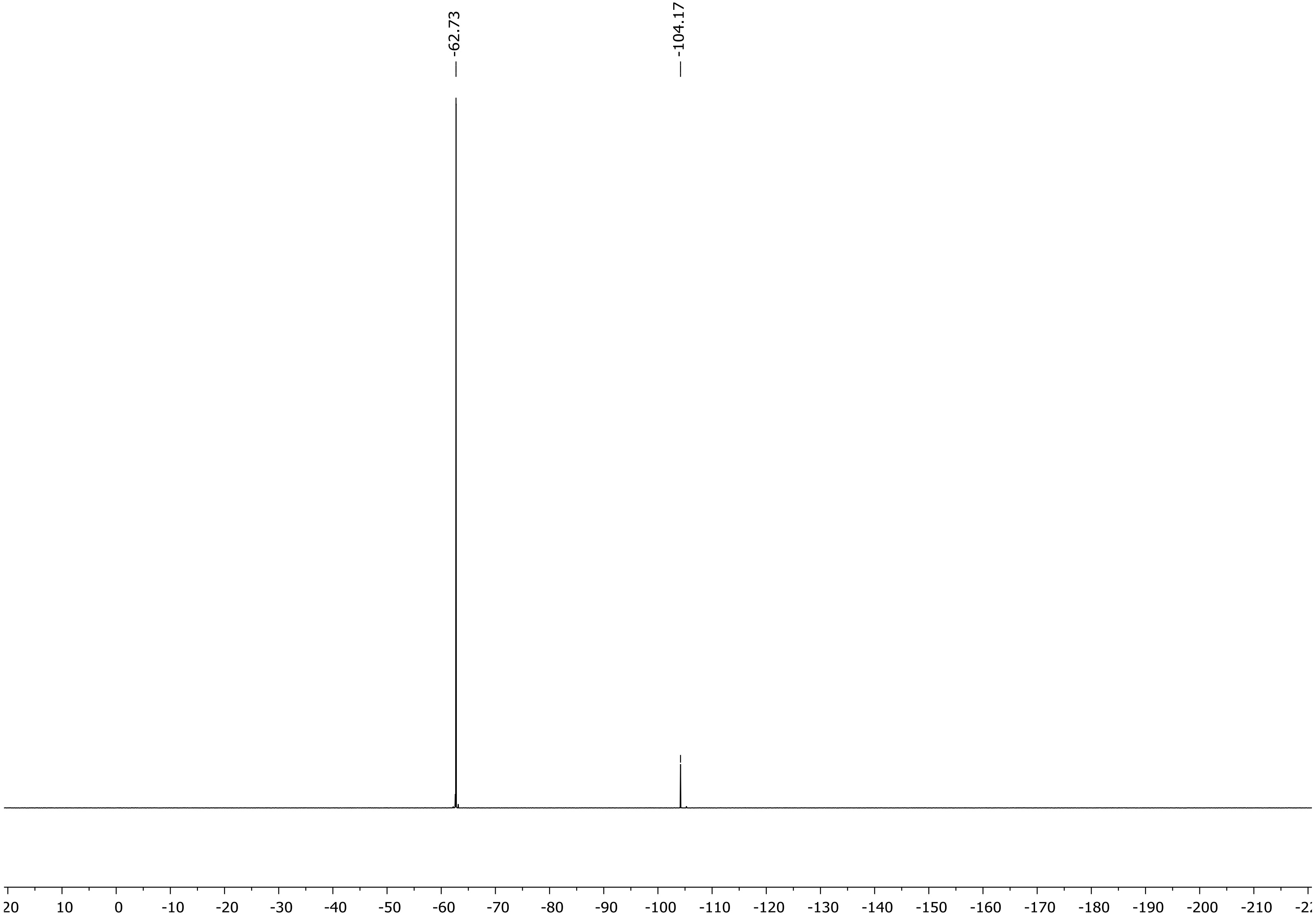

Figure S34: **^{1}H NMR** (500 MHz, $CDCl_3$, 298 K) spectrum of **1l**.

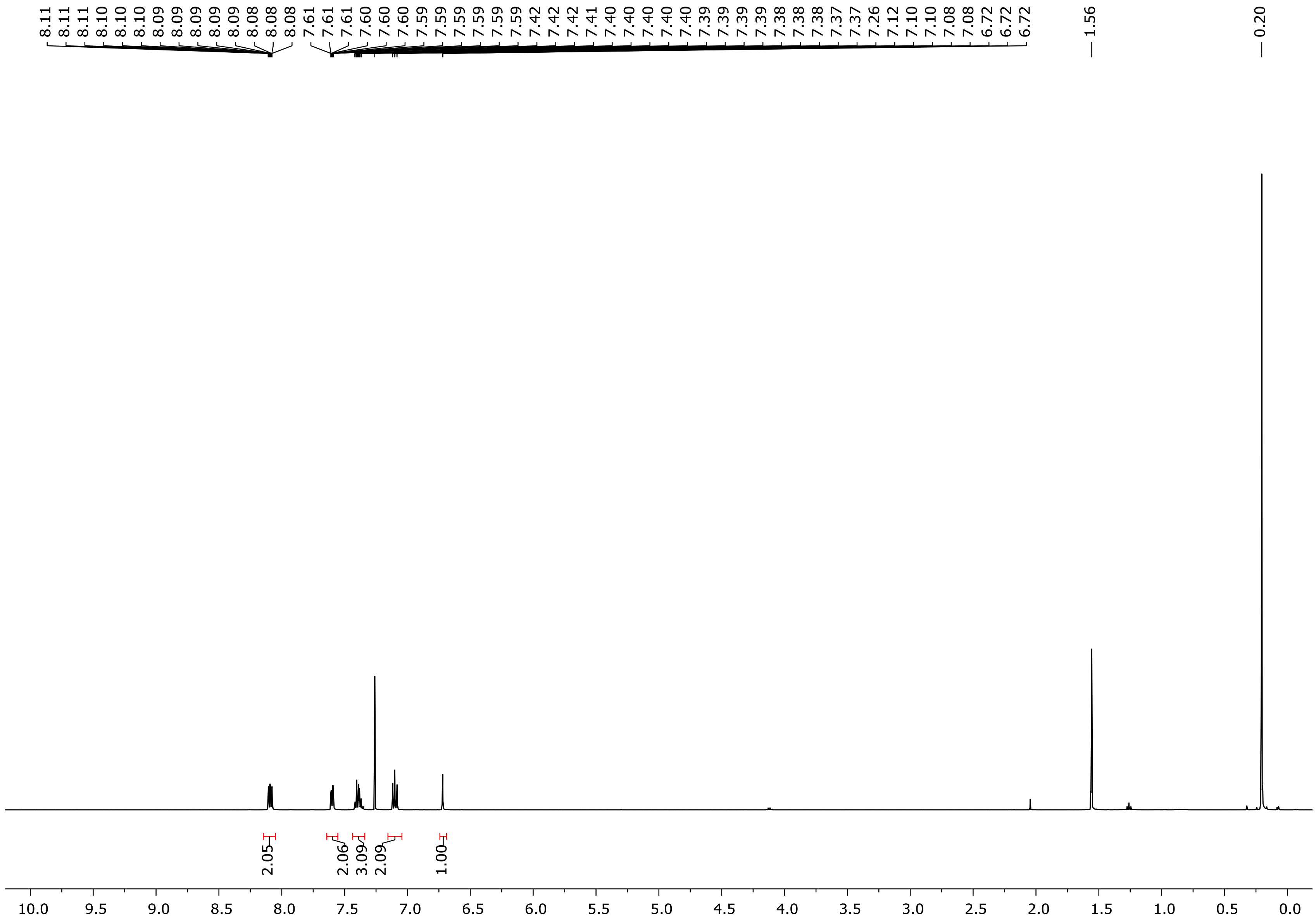

Figure S35: **^{13}C NMR** (126 MHz, $CDCl_3$, 298 K) spectrum of **1l**.

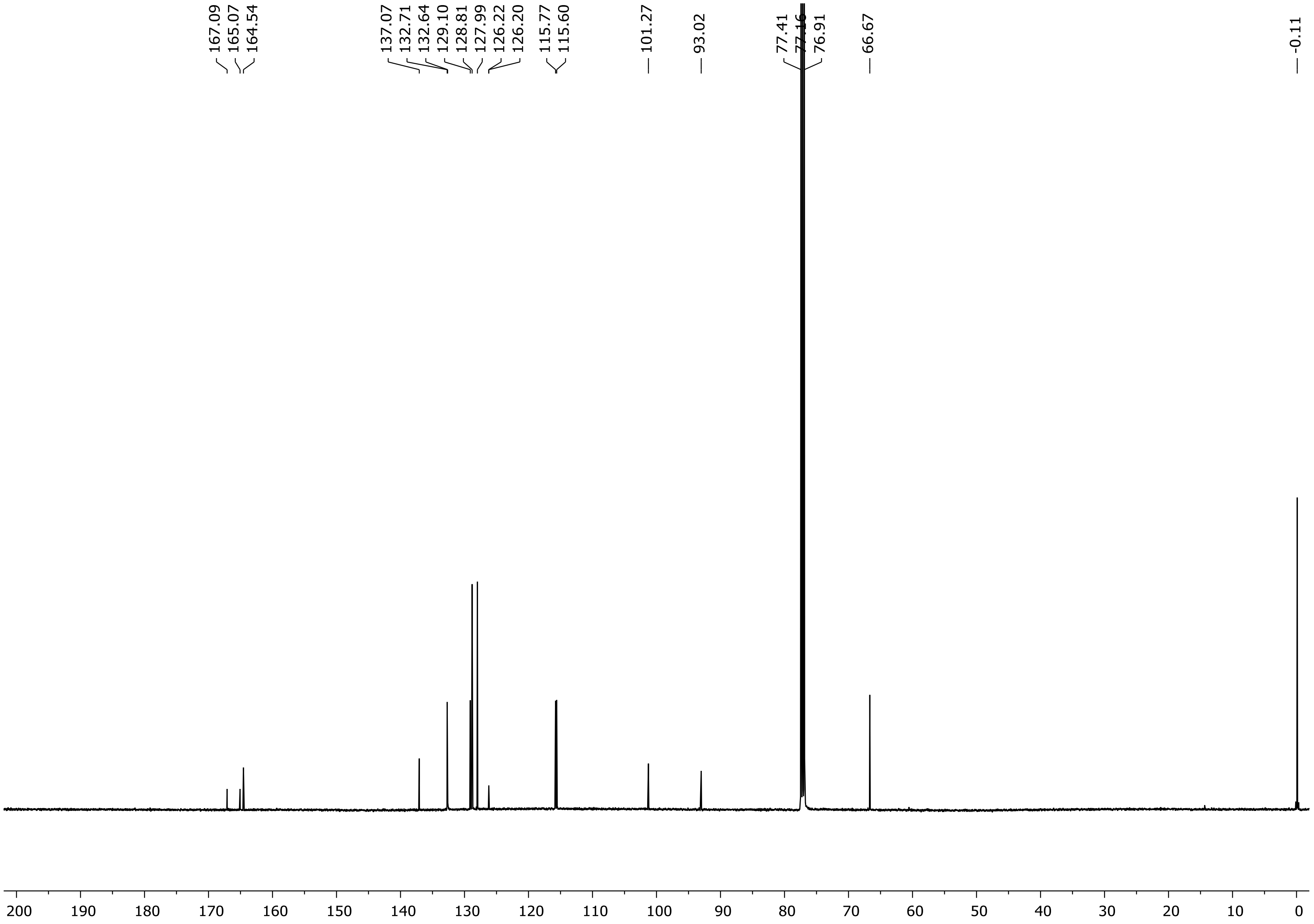

Figure S36: **^{19}F NMR** (471 MHz, $CDCl_3$, 298 K) spectrum of **1l**.

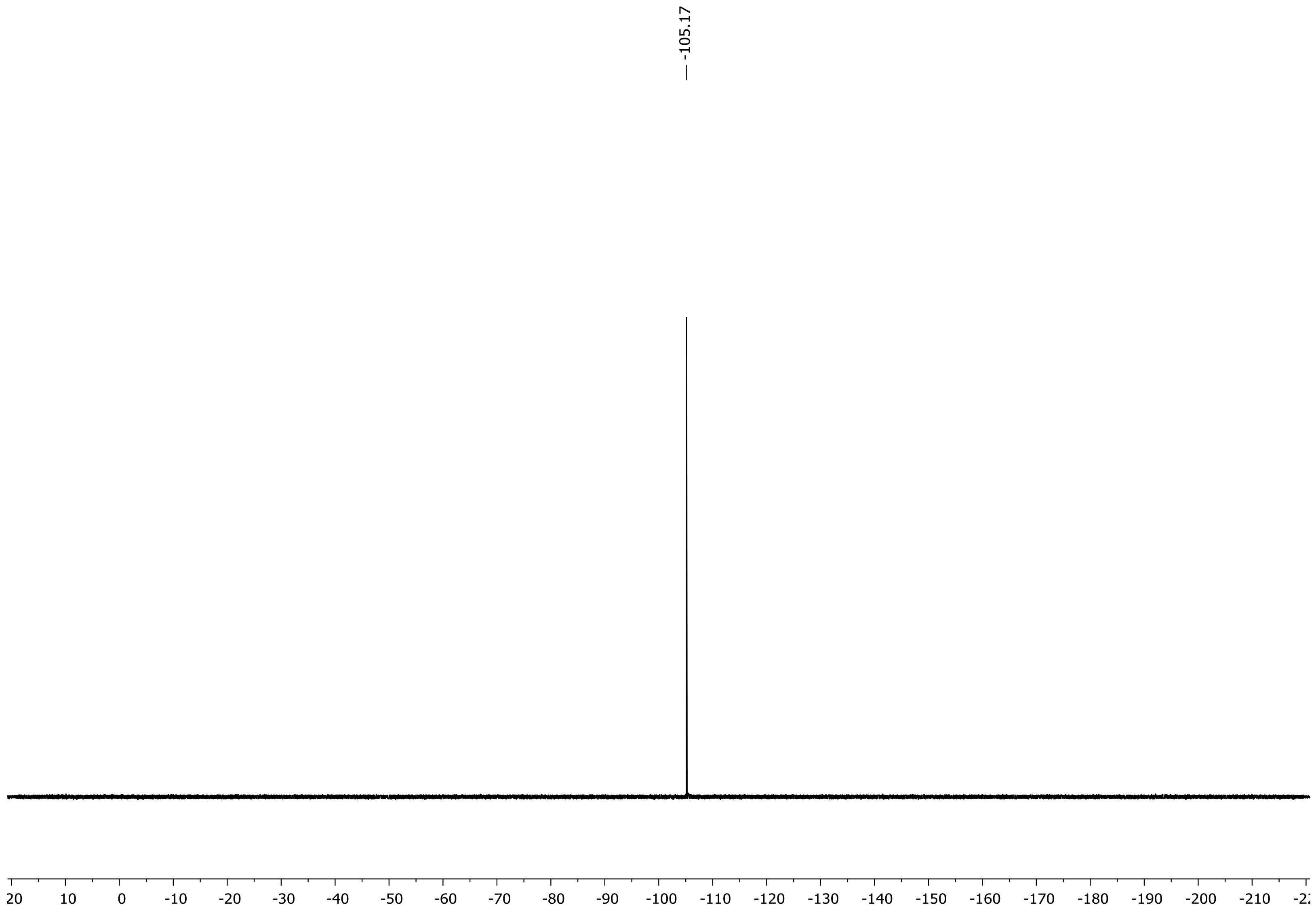

Figure S37: **^{1}H NMR** (500 MHz, $CDCl_3$, 298 K) spectrum of **4a**.

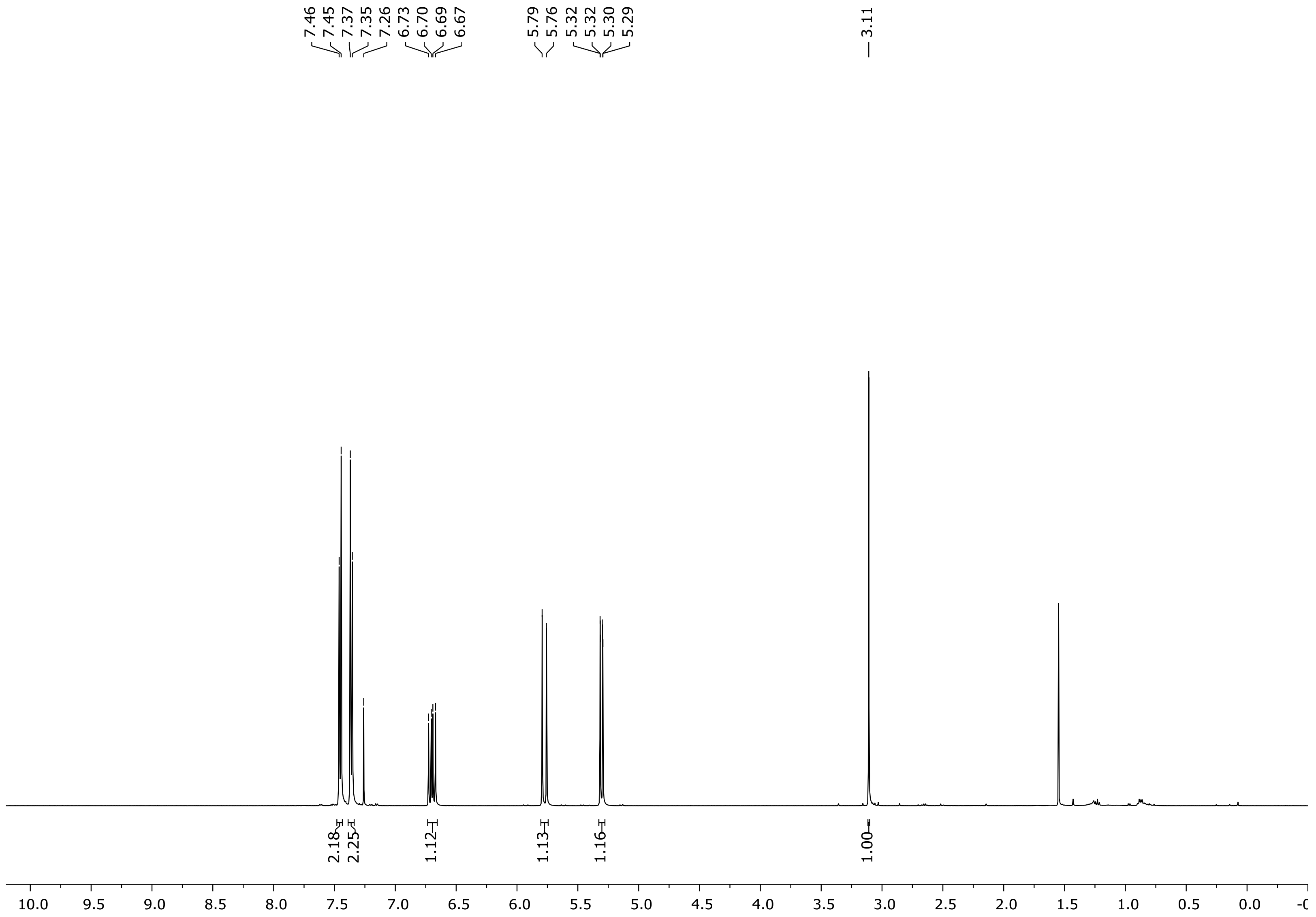

Figure S38: **^{13}C NMR** (126 MHz, $CDCl_3$, 298 K) spectrum of **4a**.

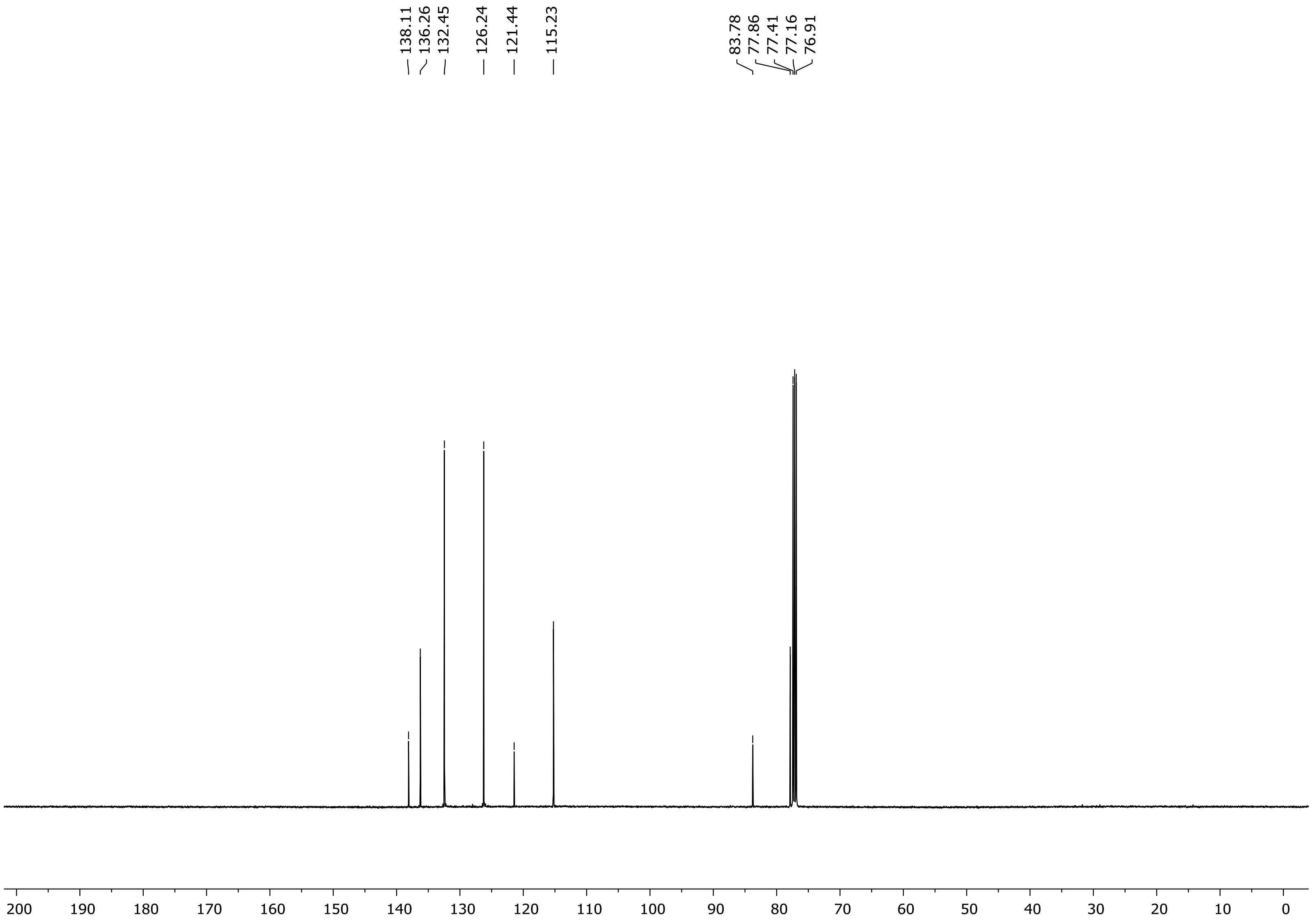

Figure S39: **^{1}H NMR** (500 MHz, $CDCl_3$, 298 K) spectrum of **4b**.

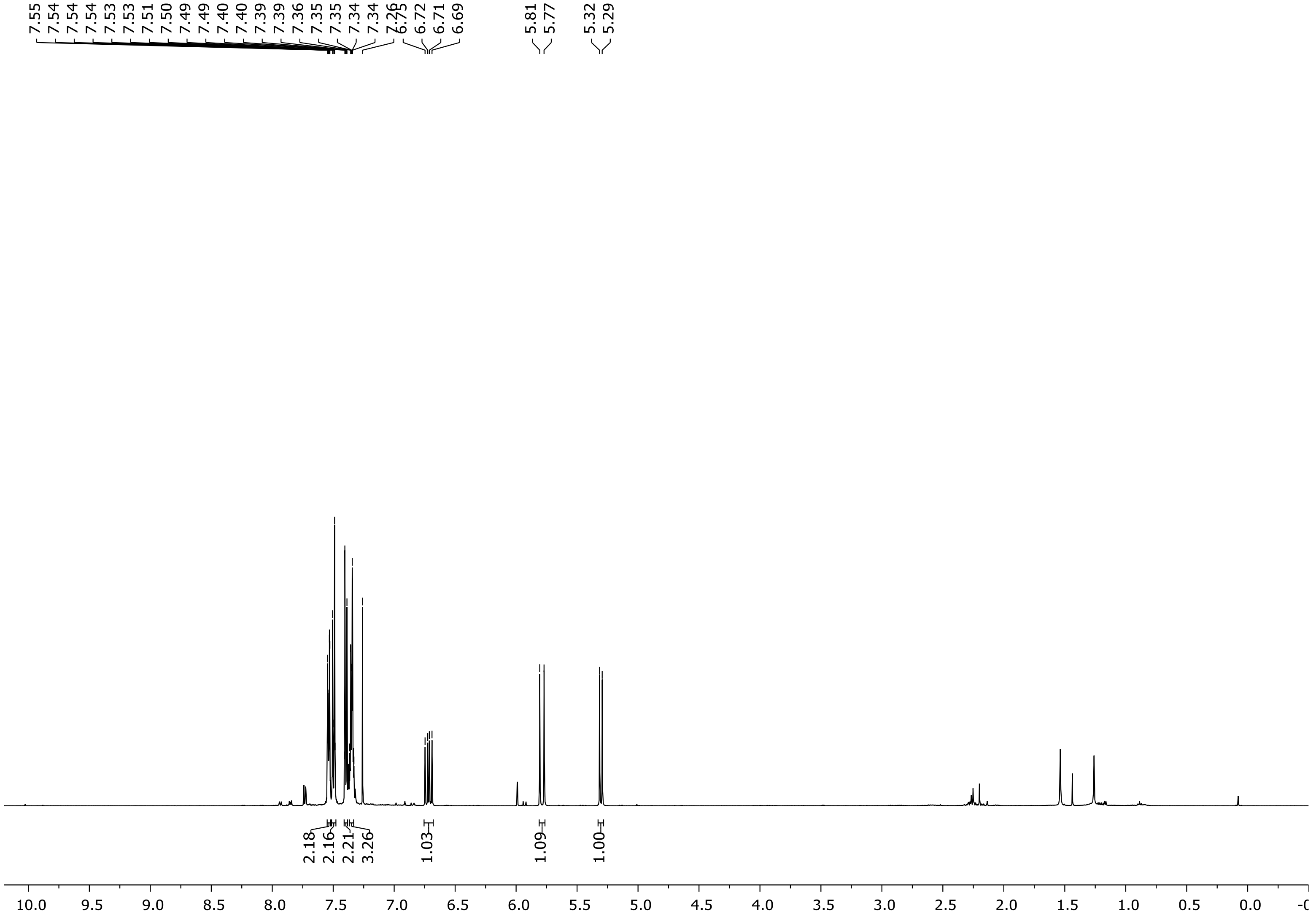

Figure S40: **^{13}C NMR** (126 MHz, $CDCl_3$, 298 K) spectrum of **4b**.

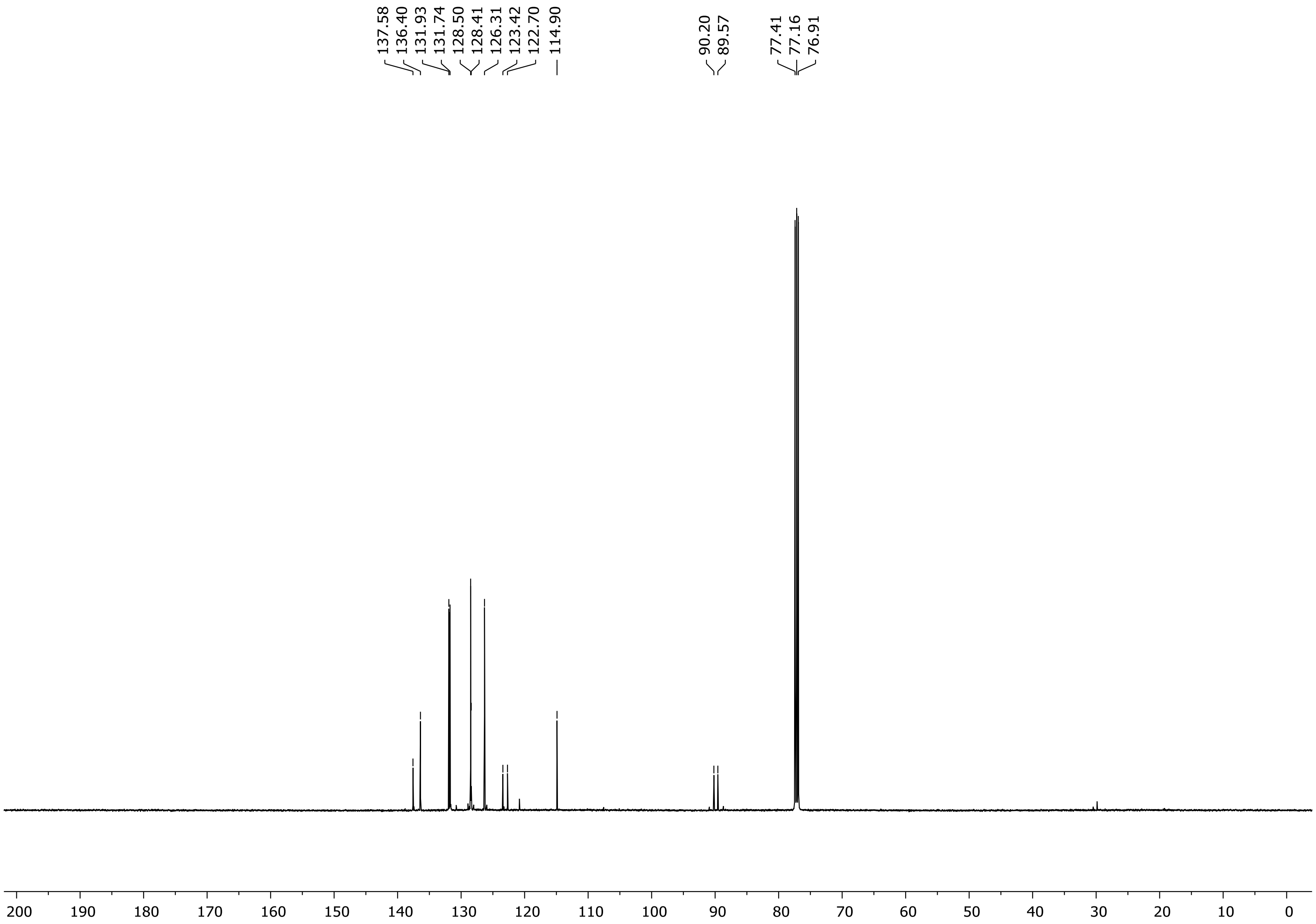

Figure S41: ^{1}**H NMR** (500 MHz, $CDCl_3$, 298 K) spectrum of **4c**.

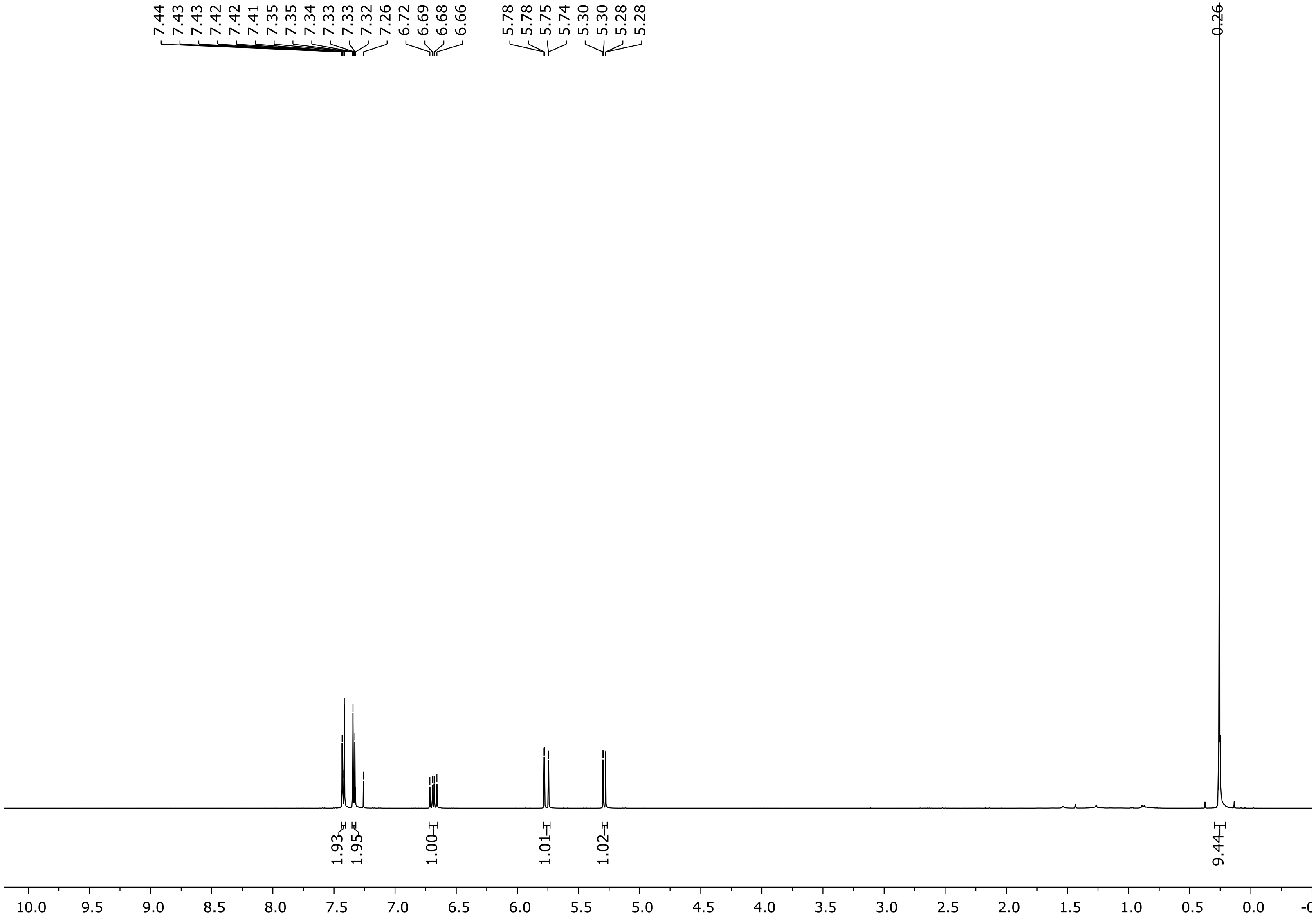

Figure S42: **^{13}C NMR** (126 MHz, $CDCl_3$, 298 K) spectrum of **4c**.

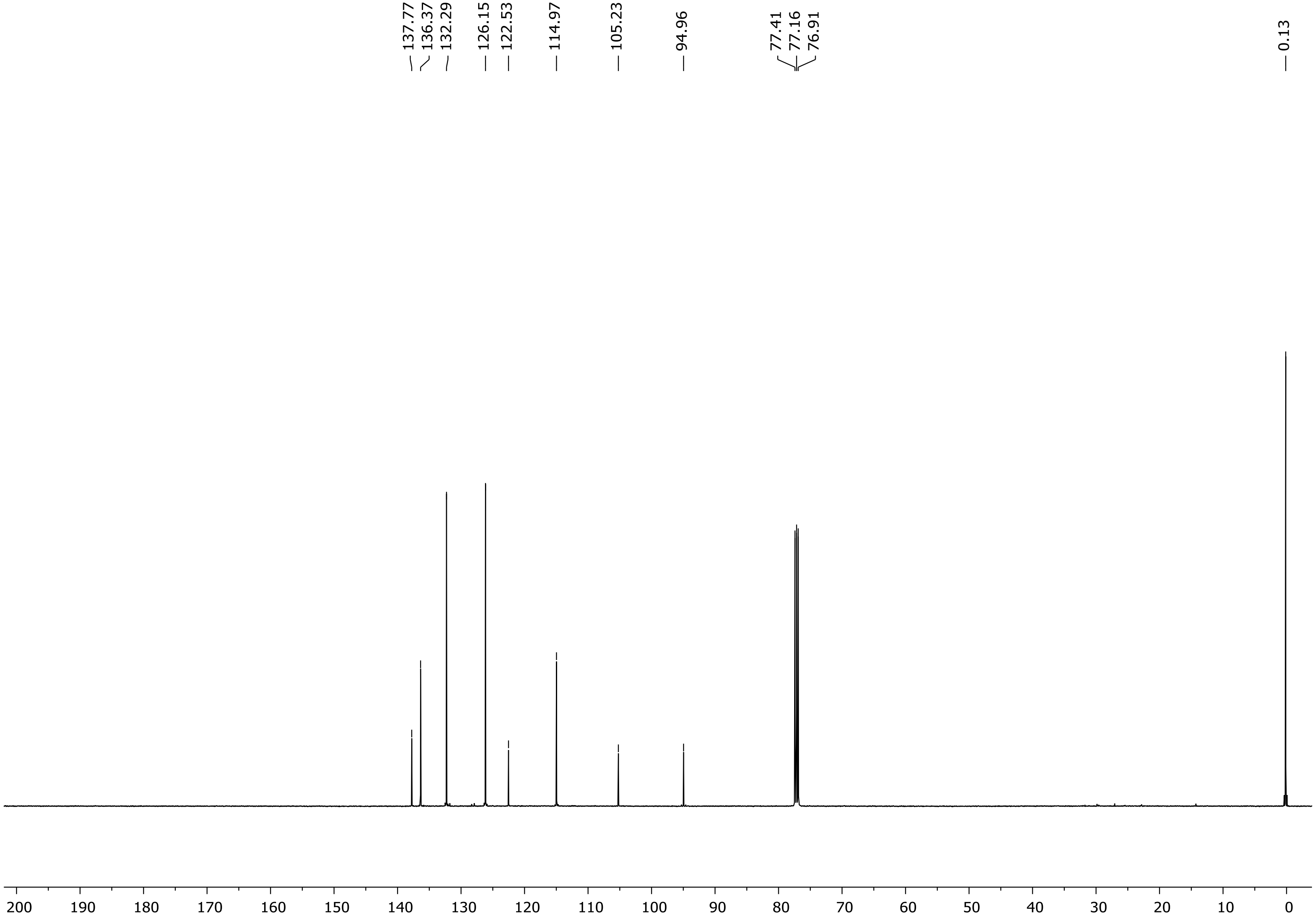

Figure S43: **^{1}H NMR** (500 MHz, $CDCl_3$, 298 K) spectrum of **4d**.

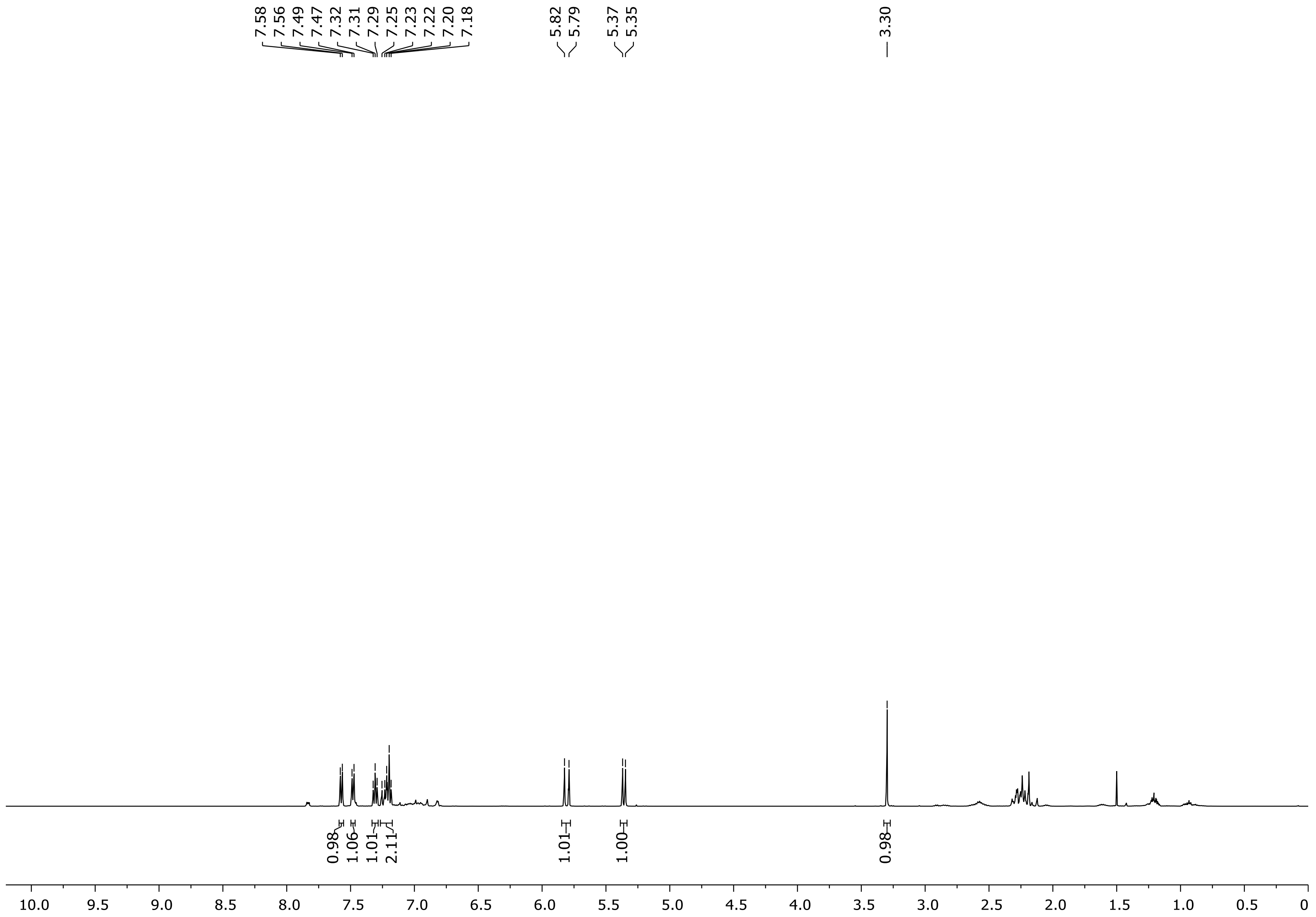

Figure S44: **^{13}C NMR** (126 MHz, $CDCl_3$, 298 K) spectrum of **4d**.

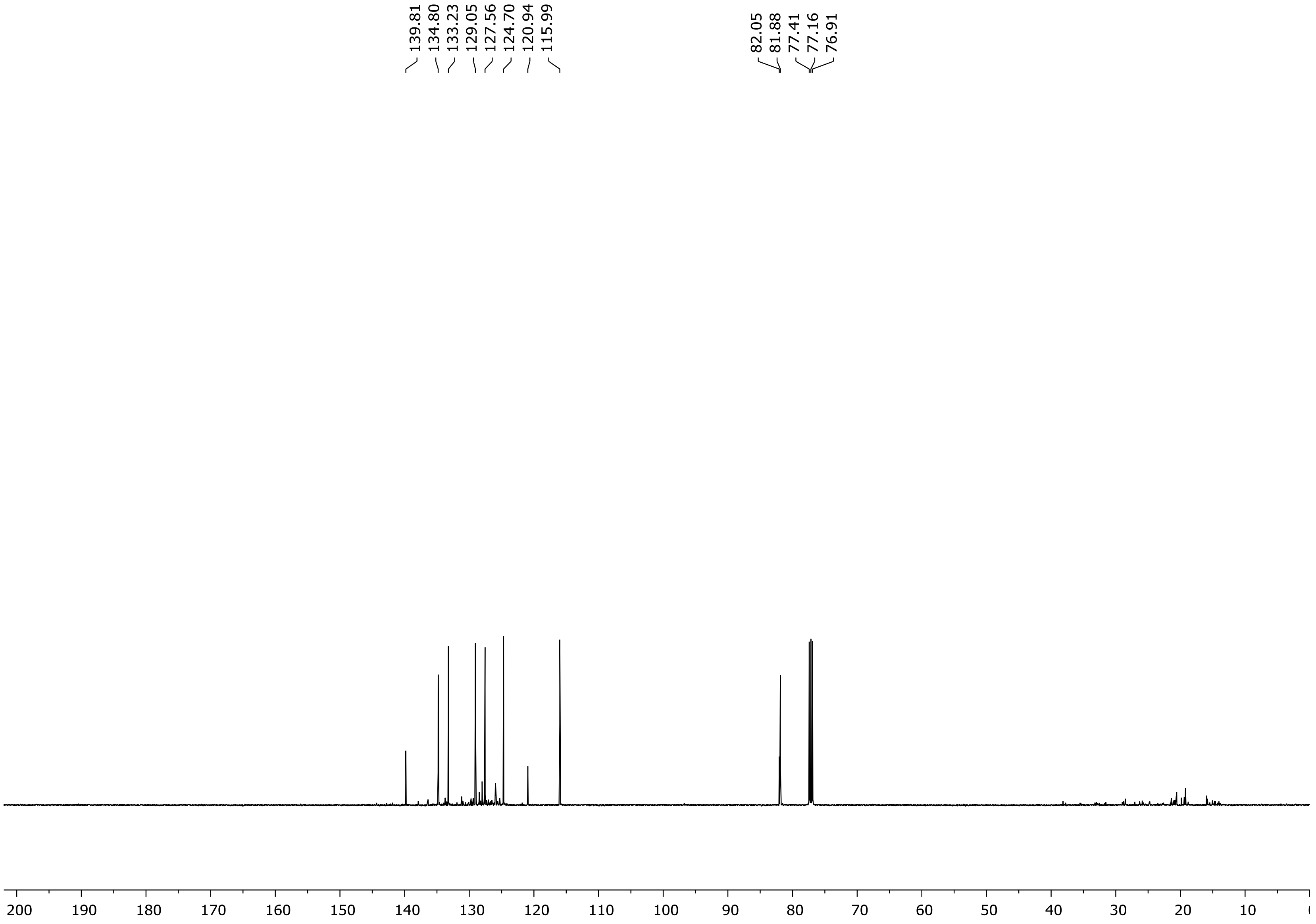

Figure S45: **^{1}H NMR** (500 MHz, $CDCl_3$, 298 K) spectrum of trimethyl(2-phenylethynyl)silane.

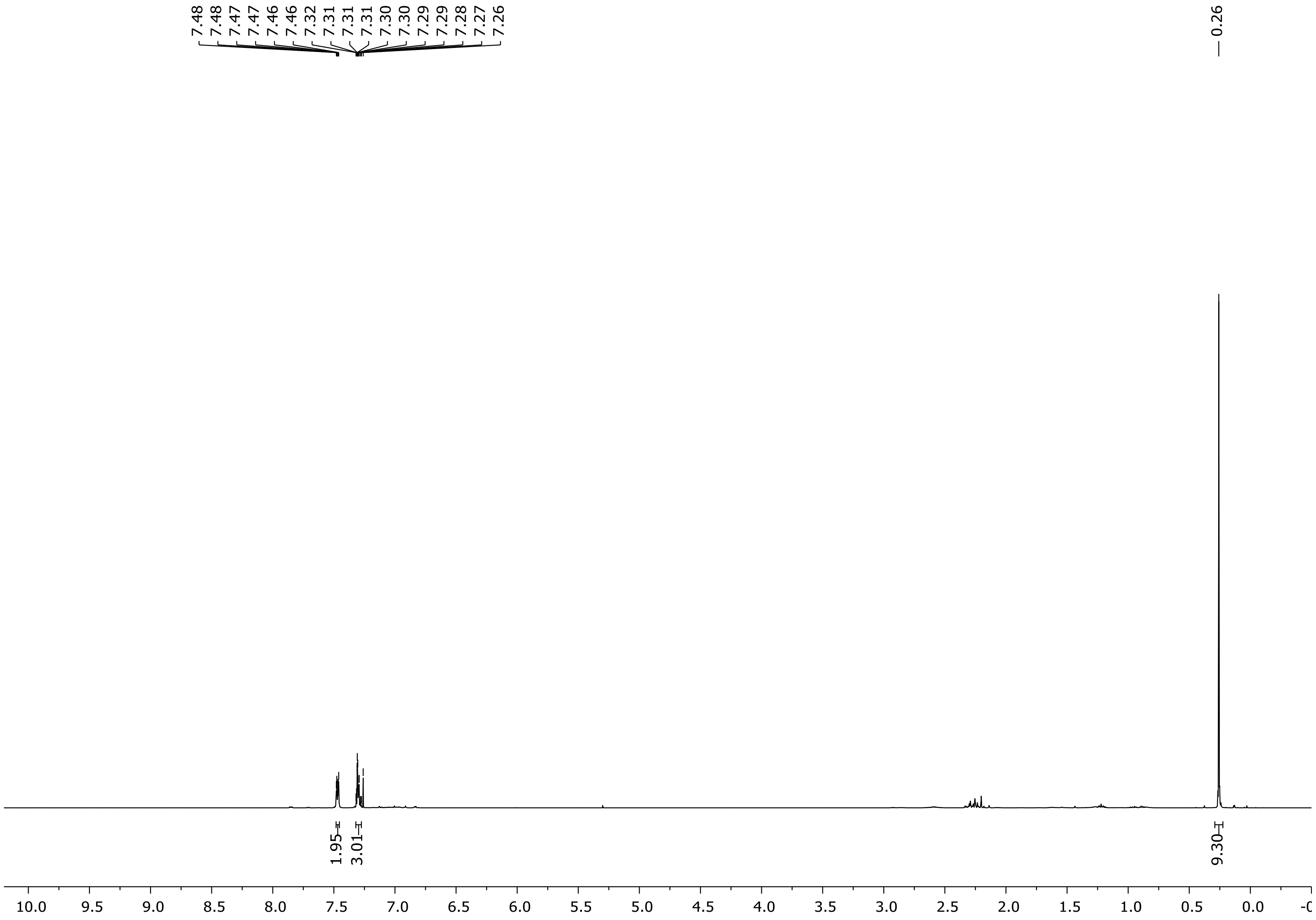

Figure S46: ^{13}**C NMR** (126 MHz, $CDCl_3$, 298 K) spectrum of trimethyl(2-phenylethynyl)silane.

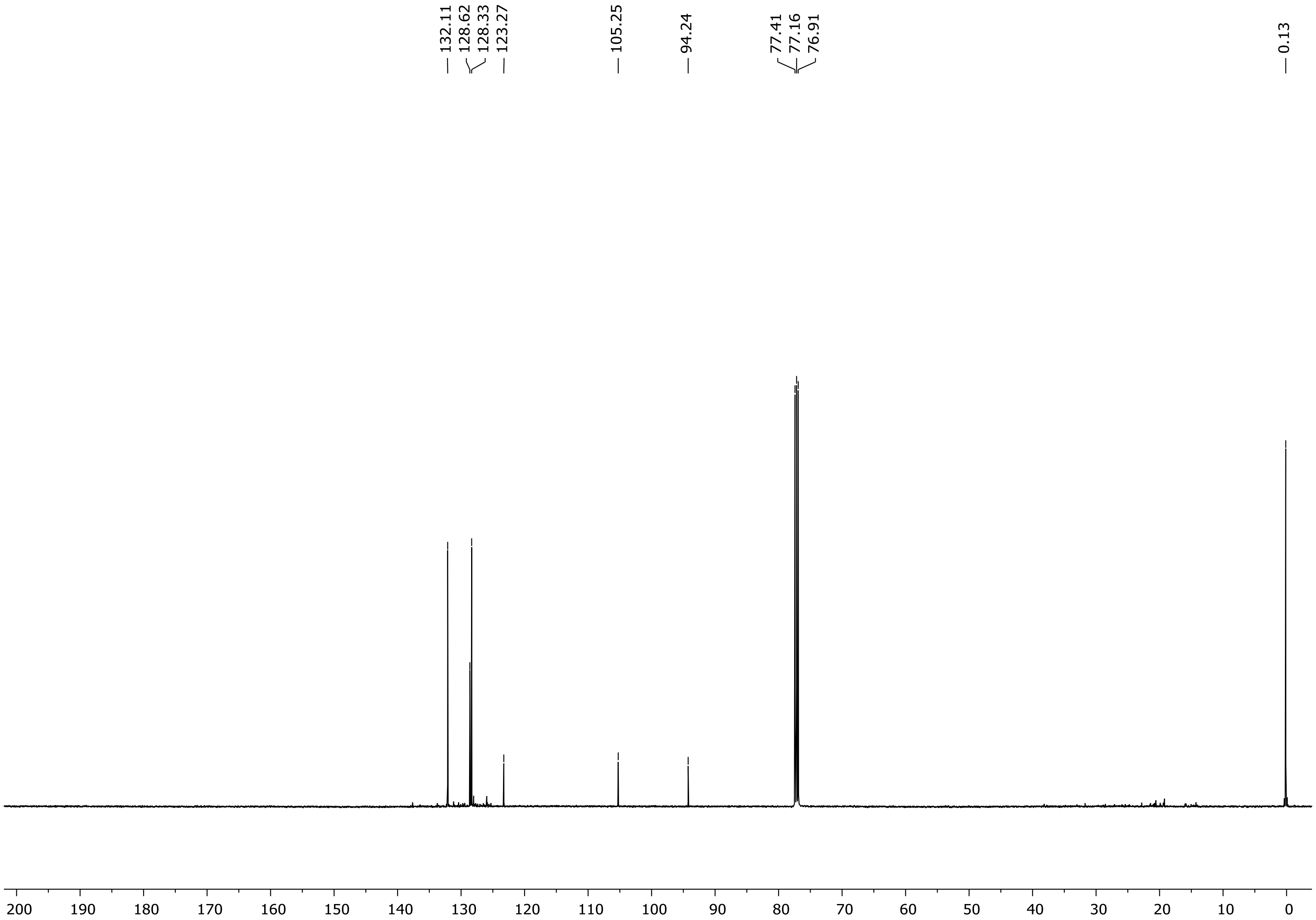

Figure S47: **^{1}H NMR** (400 MHz, $CDCl_3$, 298 K) spectrum of **2a**.

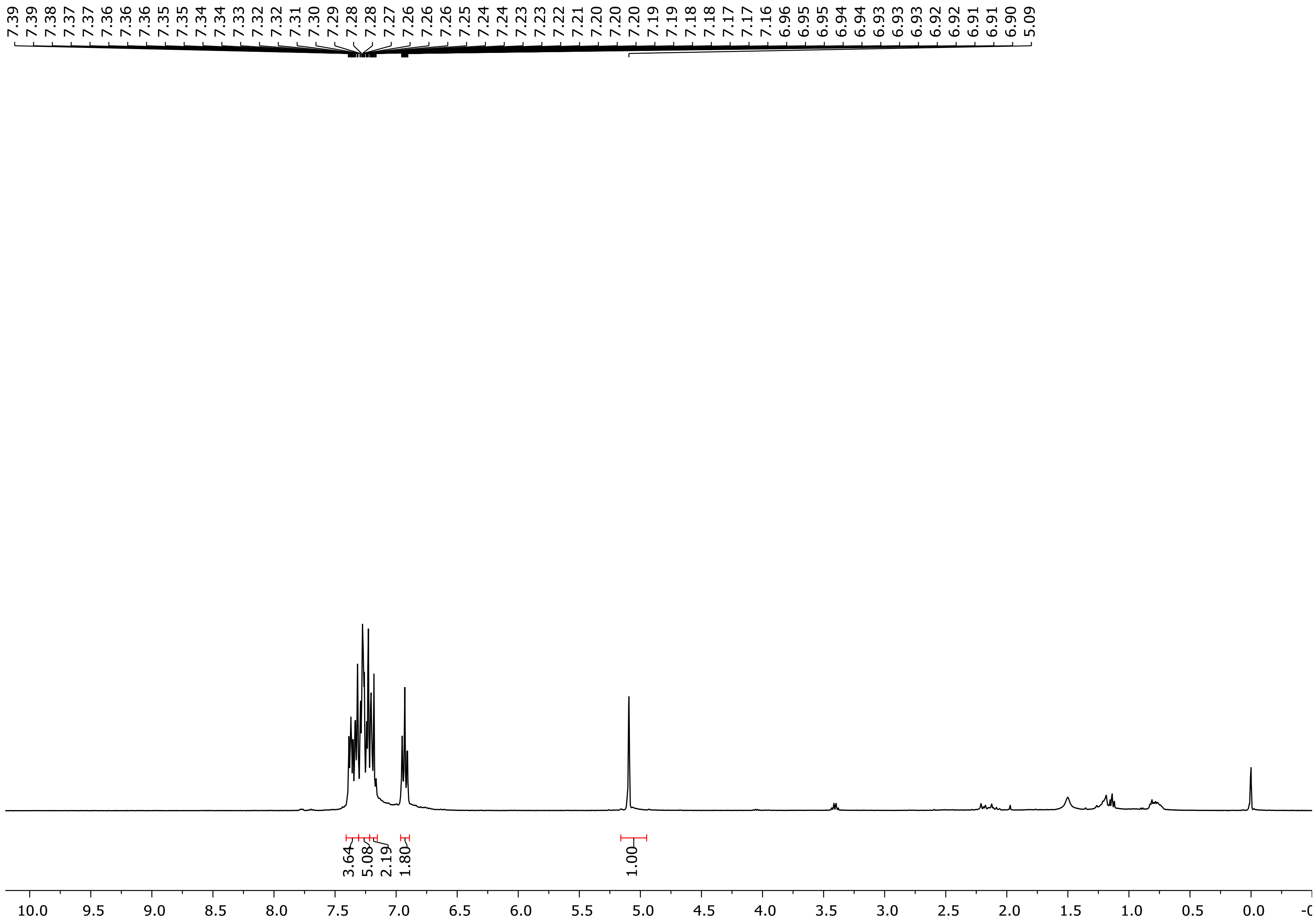

Figure S48: **^{13}C NMR** (101 MHz, $CDCl_3$, 298 K) spectrum of **2a**.

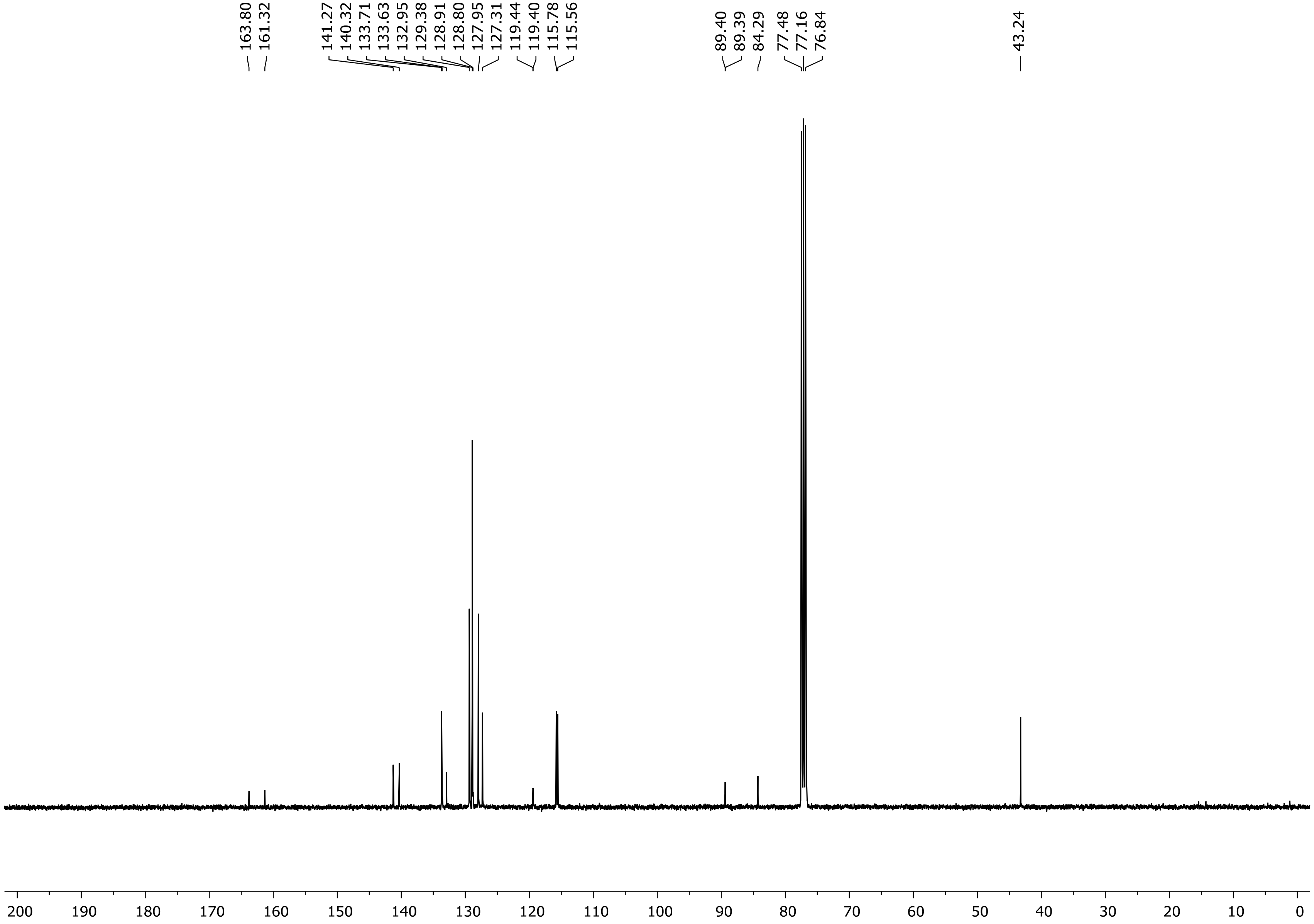

Figure S49: **¹⁹F NMR** (376 MHz, $CDCl_3$, 298 K) spectrum of **2a**.

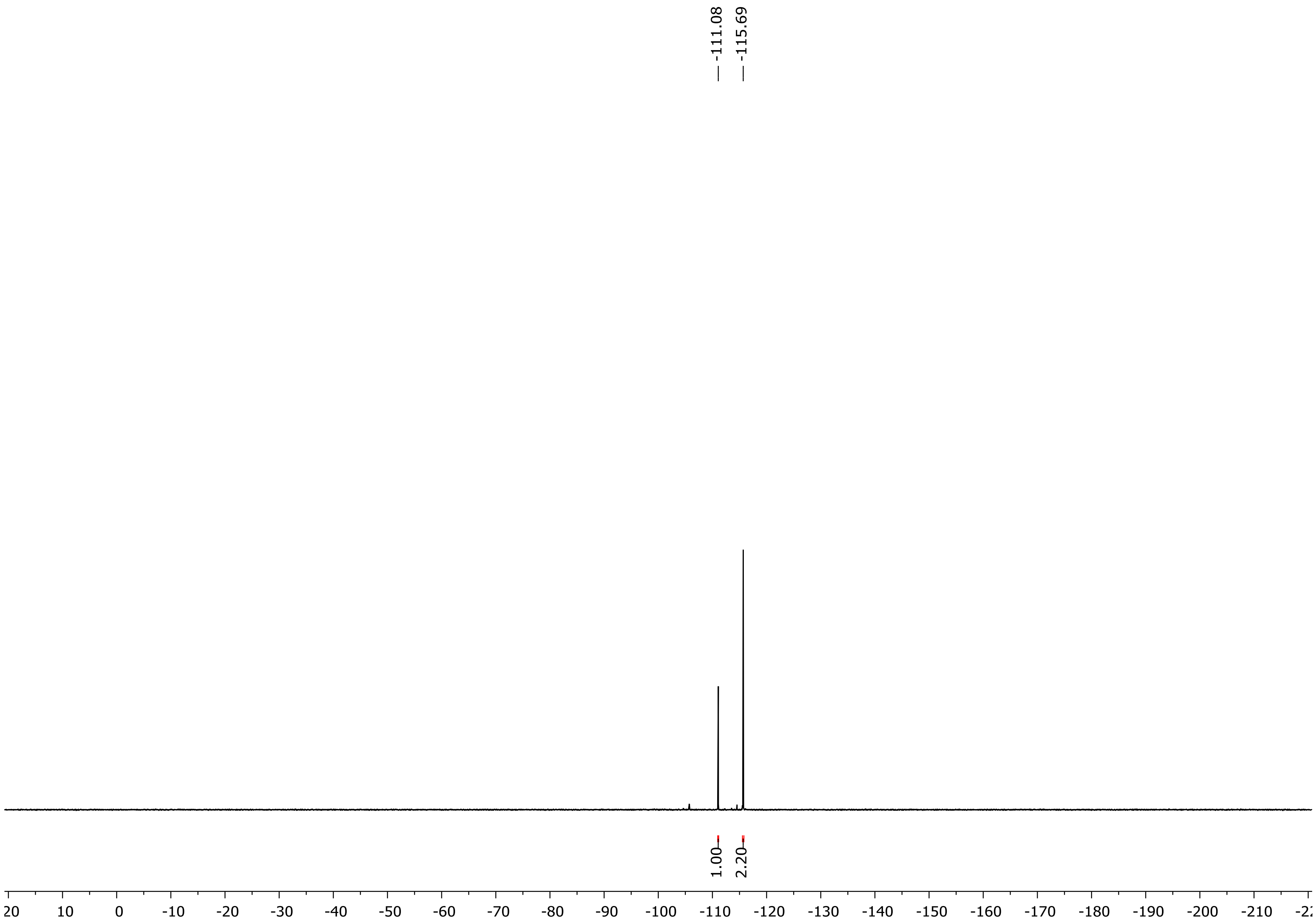

Figure S50: **^{1}H NMR** (400 MHz, $CDCl_3$, 298 K) spectrum of **2b**.

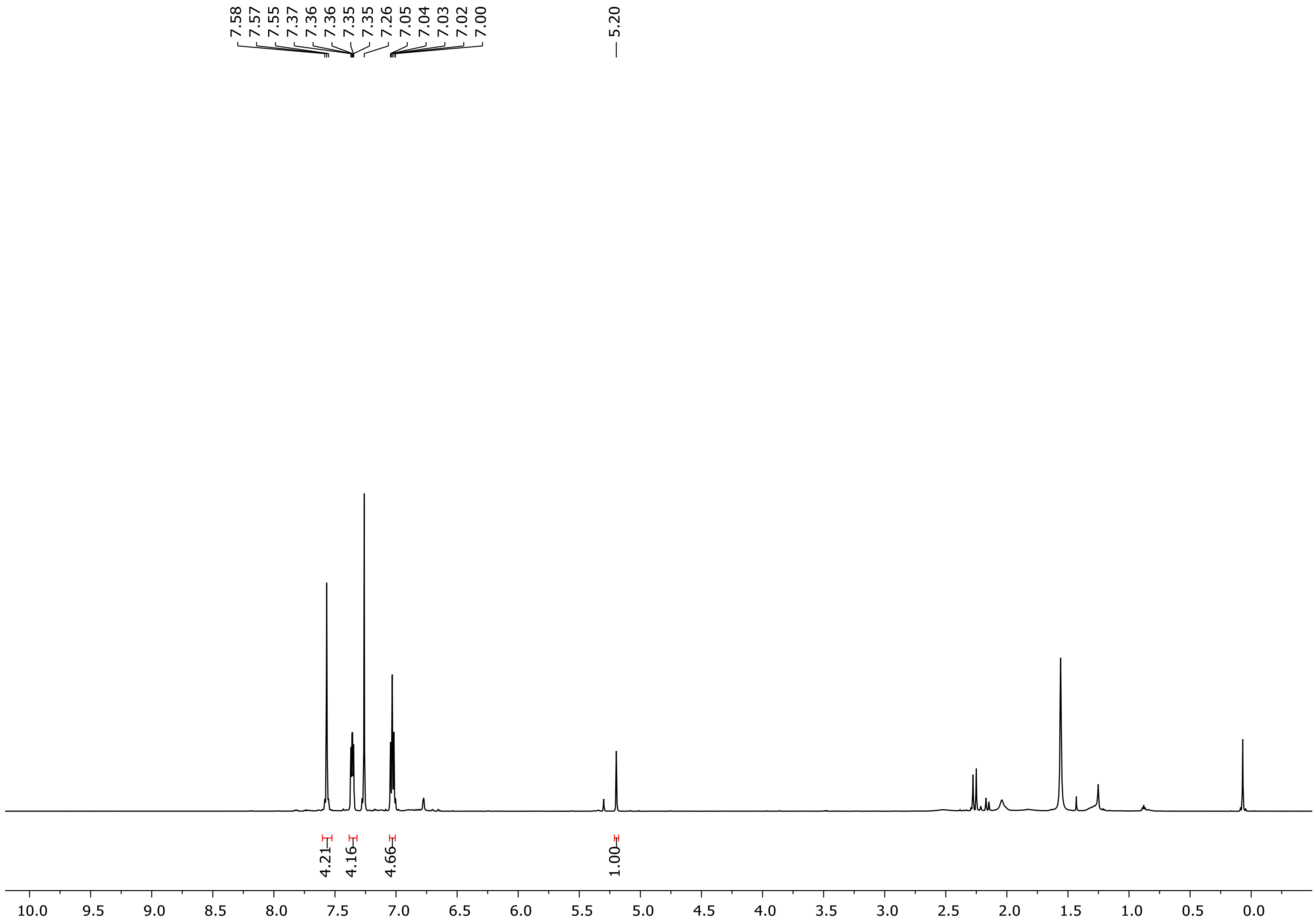

Figure S51: **^{13}C NMR** (101 MHz, $CDCl_3$, 298 K) spectrum of **2b**.

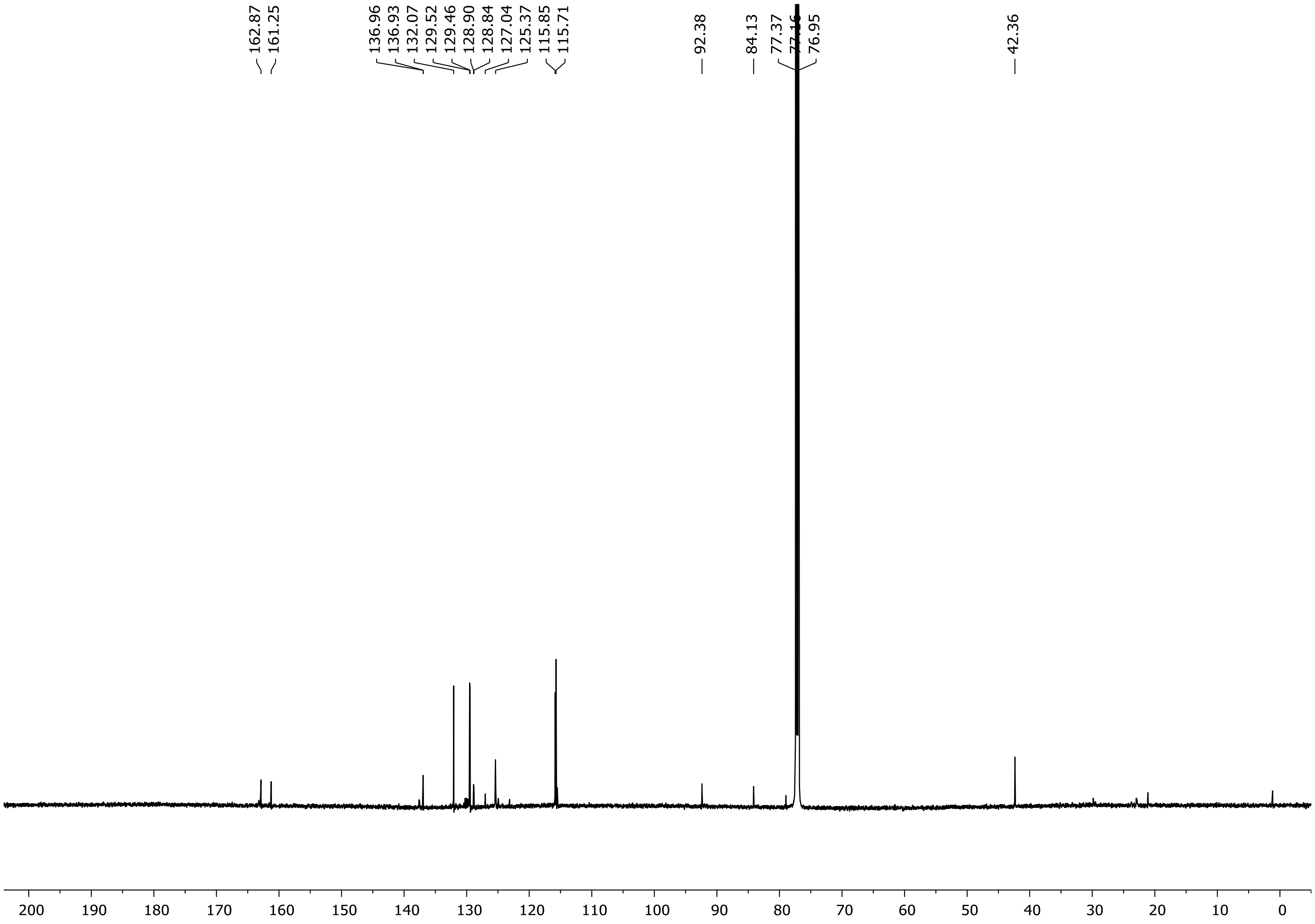

Figure S52: **^{19}F NMR** (376 MHz, $CDCl_3$, 298 K) spectrum of **2b**.

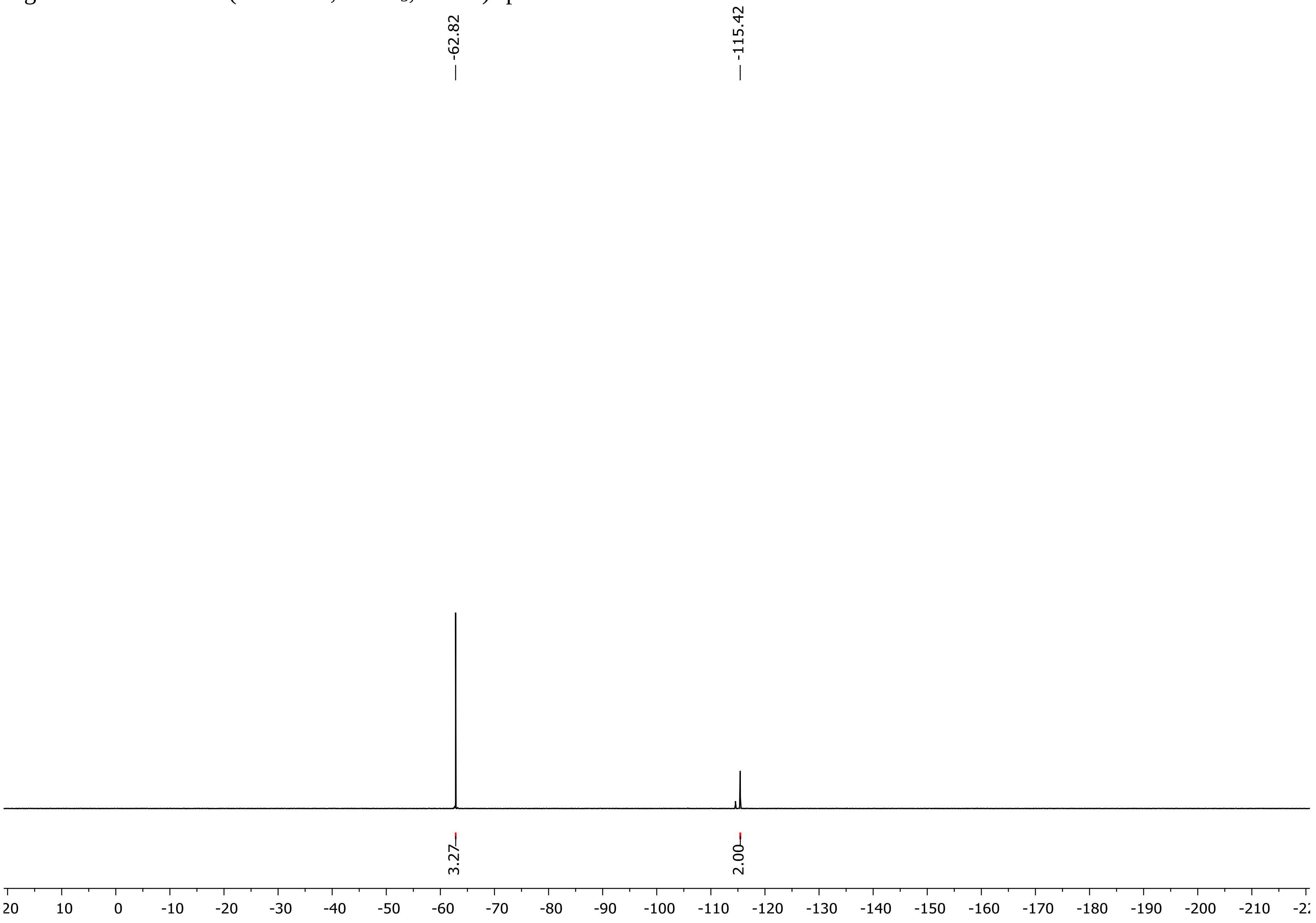

Figure S53: **^{1}H NMR** (400 MHz, $CDCl_3$, 298 K) spectrum of **2c**.

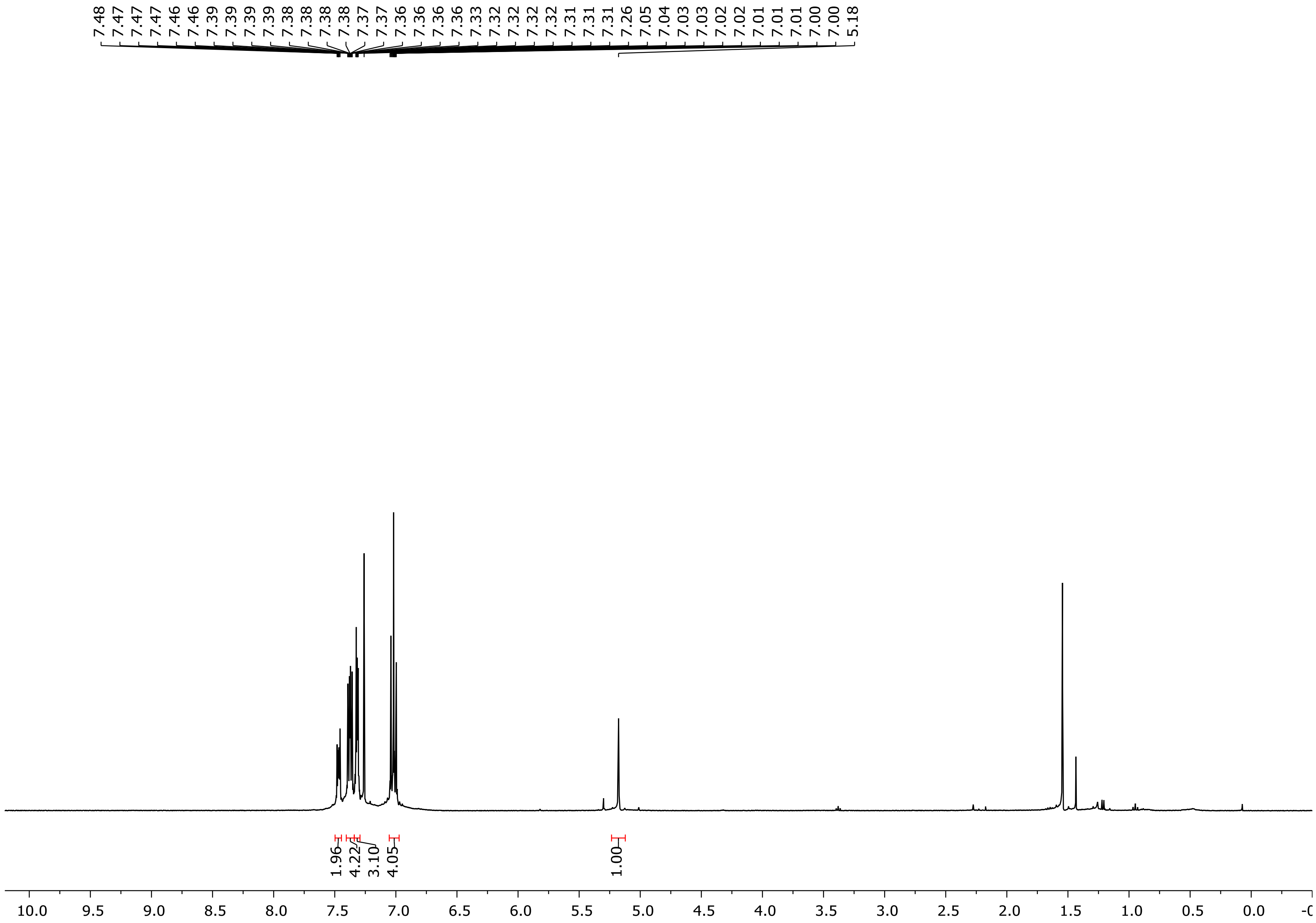

Figure S54: **^{13}C NMR** (101 MHz, $CDCl_3$, 298 K) spectrum of **2c**.

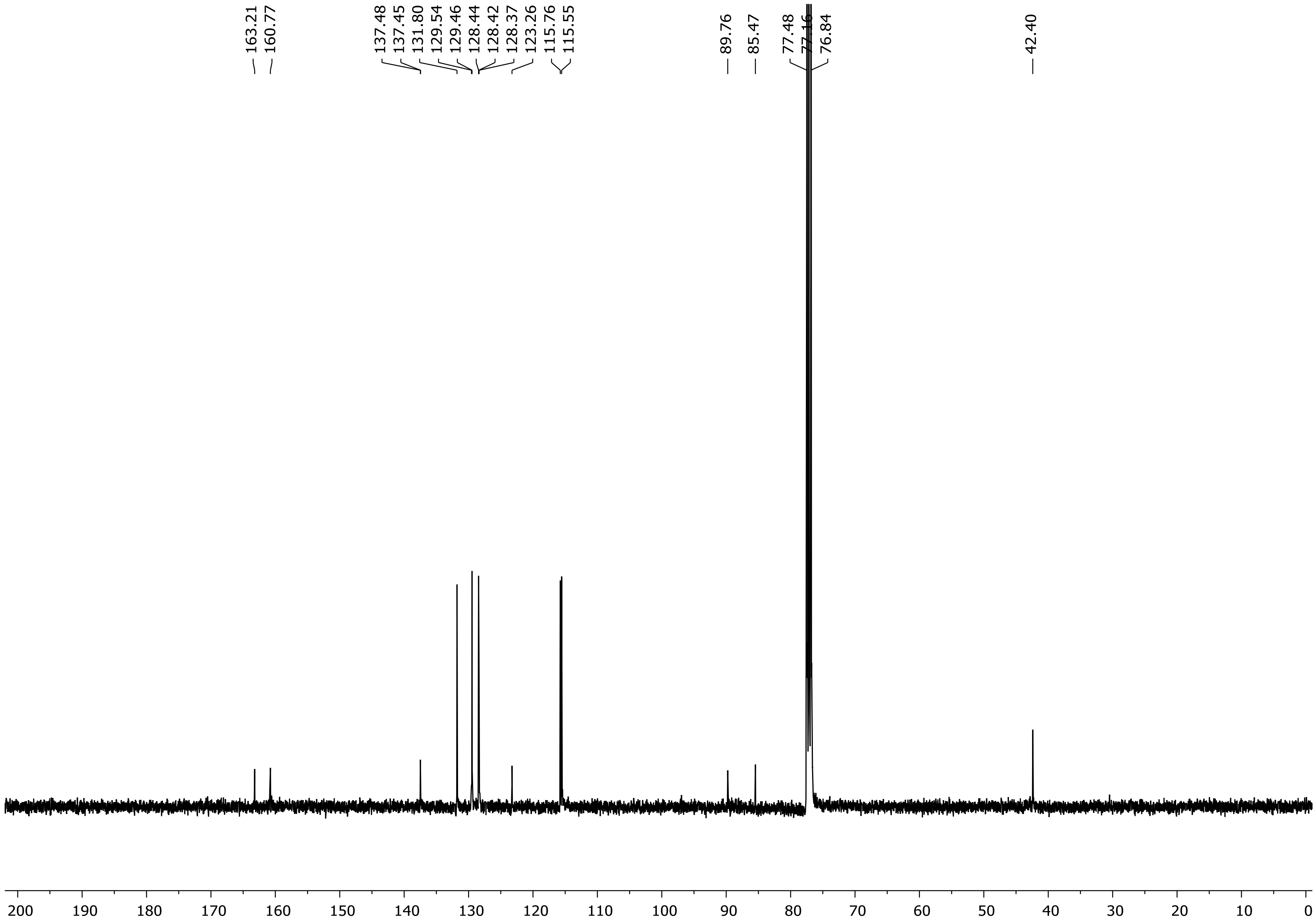

Figure S55: **^{19}F NMR** (376 MHz, $CDCl_3$, 298 K) spectrum of **2c**.

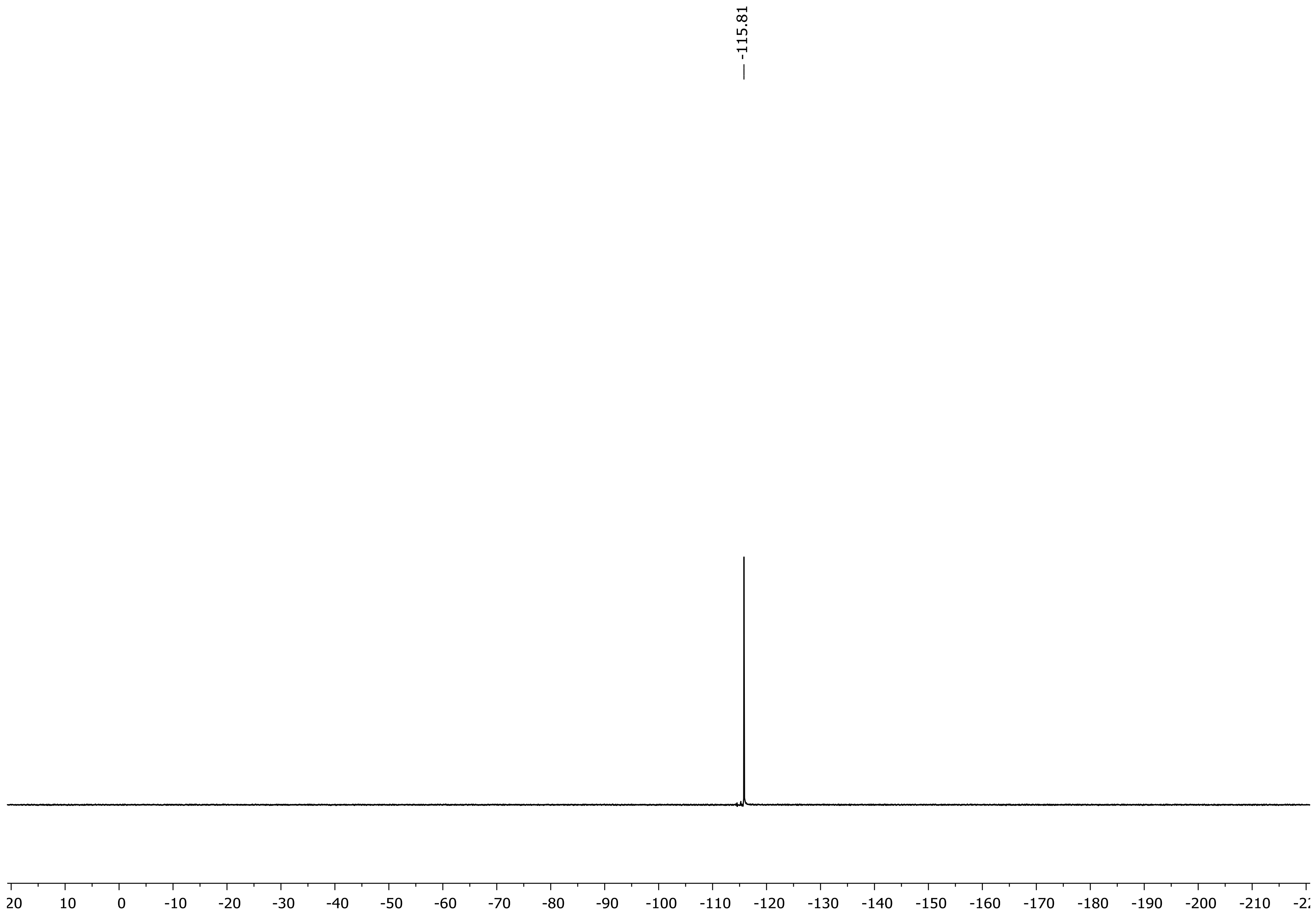

Figure S56: **^{1}H NMR** (400 MHz, $CDCl_3$, 298 K) spectrum of **2d**.

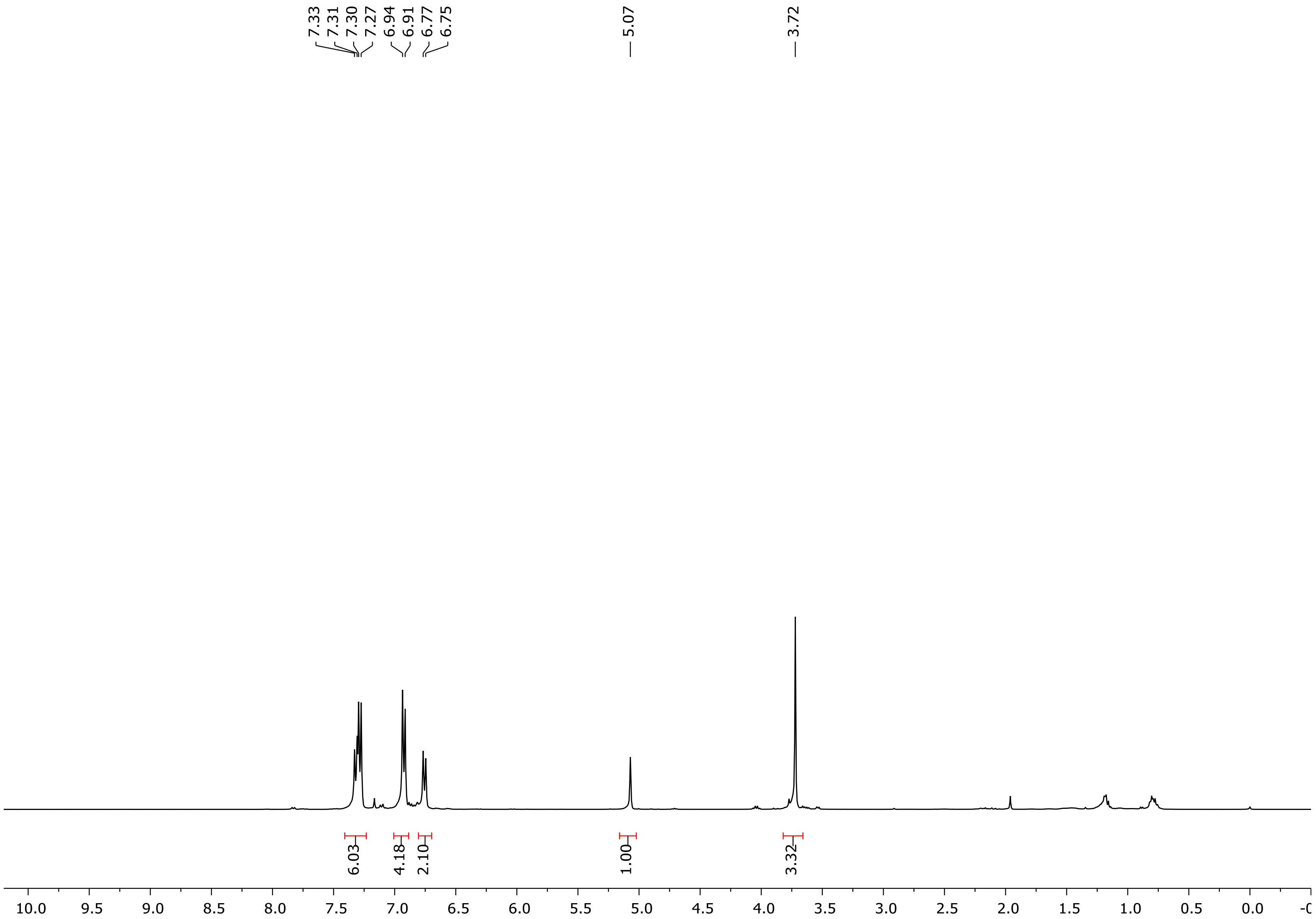

Figure S57: **^{13}C NMR** (101 MHz, $CDCl_3$, 298 K) spectrum of **2d**.

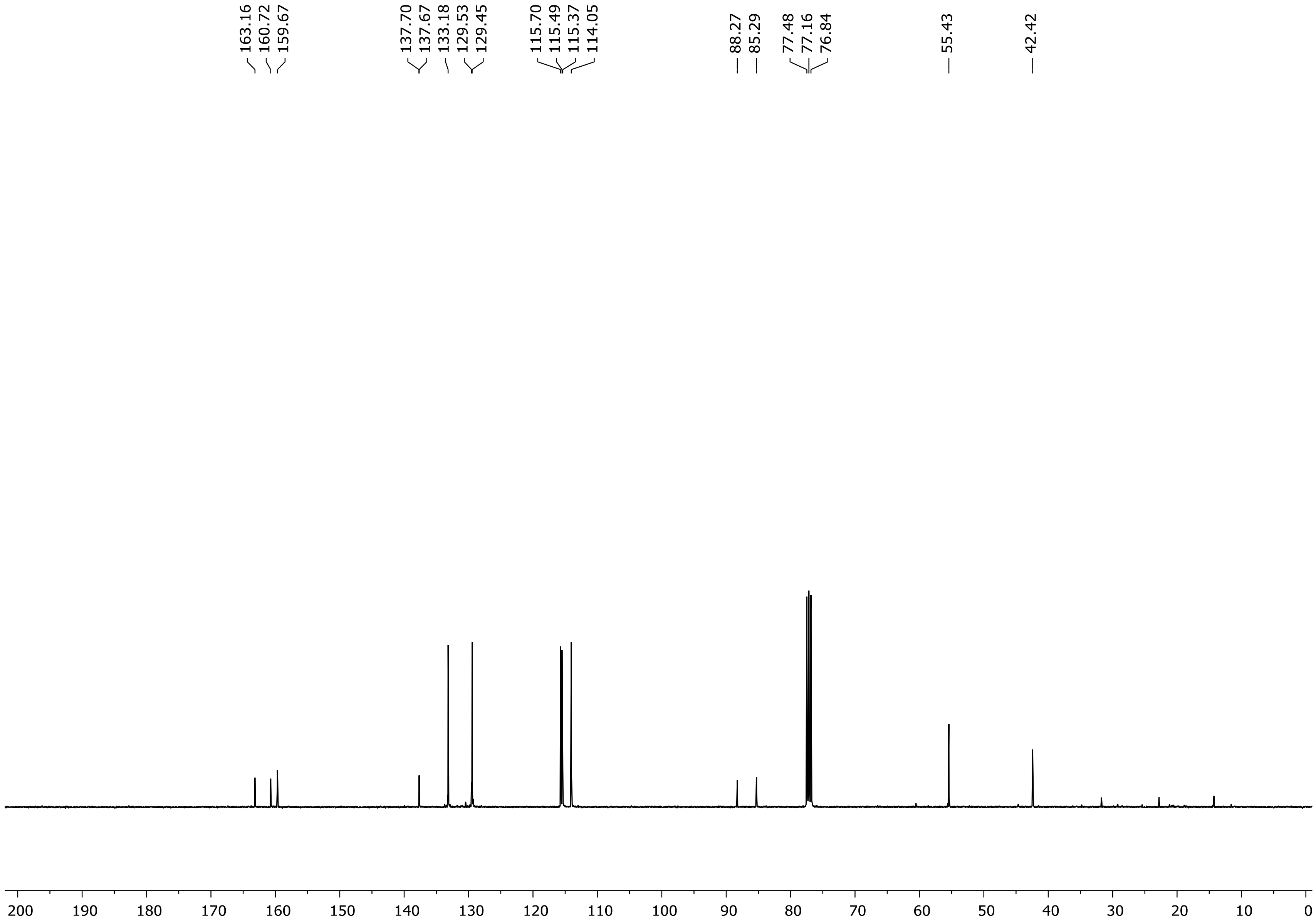

Figure S58: **^{19}F NMR** (376 MHz, $CDCl_3$, 298 K) spectrum of **2d**.

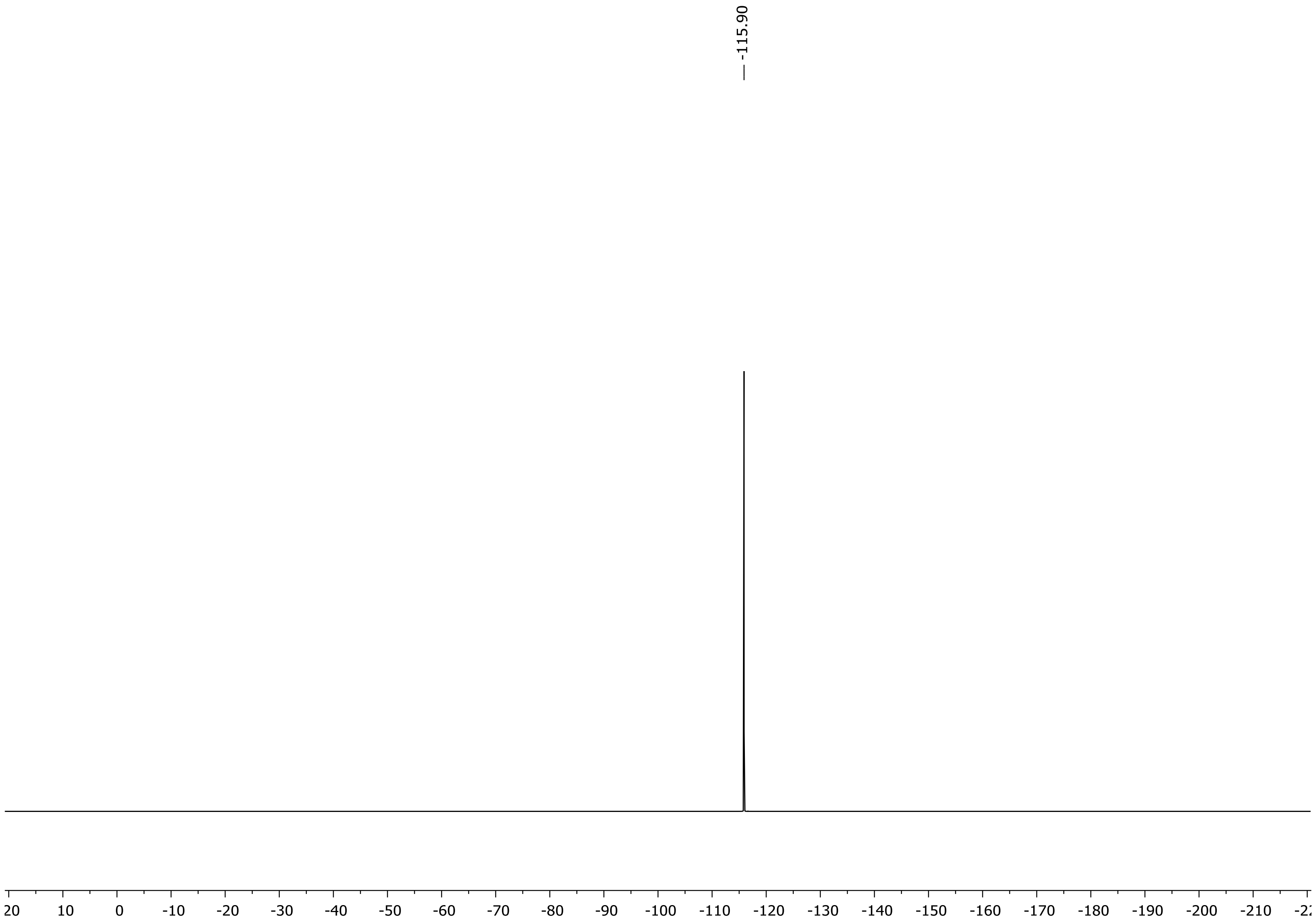

Figure S59: **^{1}H NMR** (500 MHz, $CDCl_3$, 298 K) spectrum of **2e**.

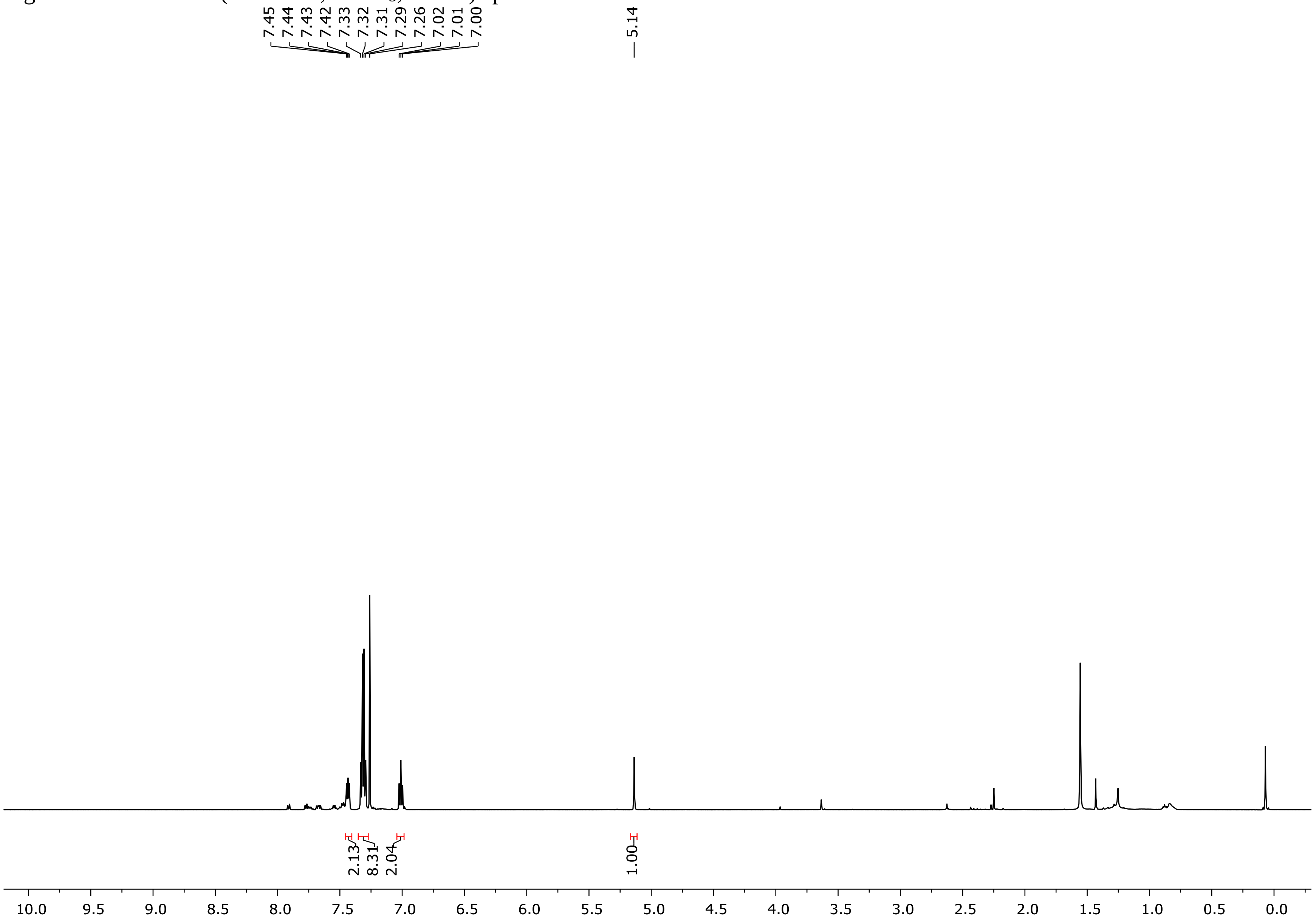

Figure S60: ^{13}**C NMR** (125 MHz, $CDCl_3$, 298 K) spectrum of **2e**.

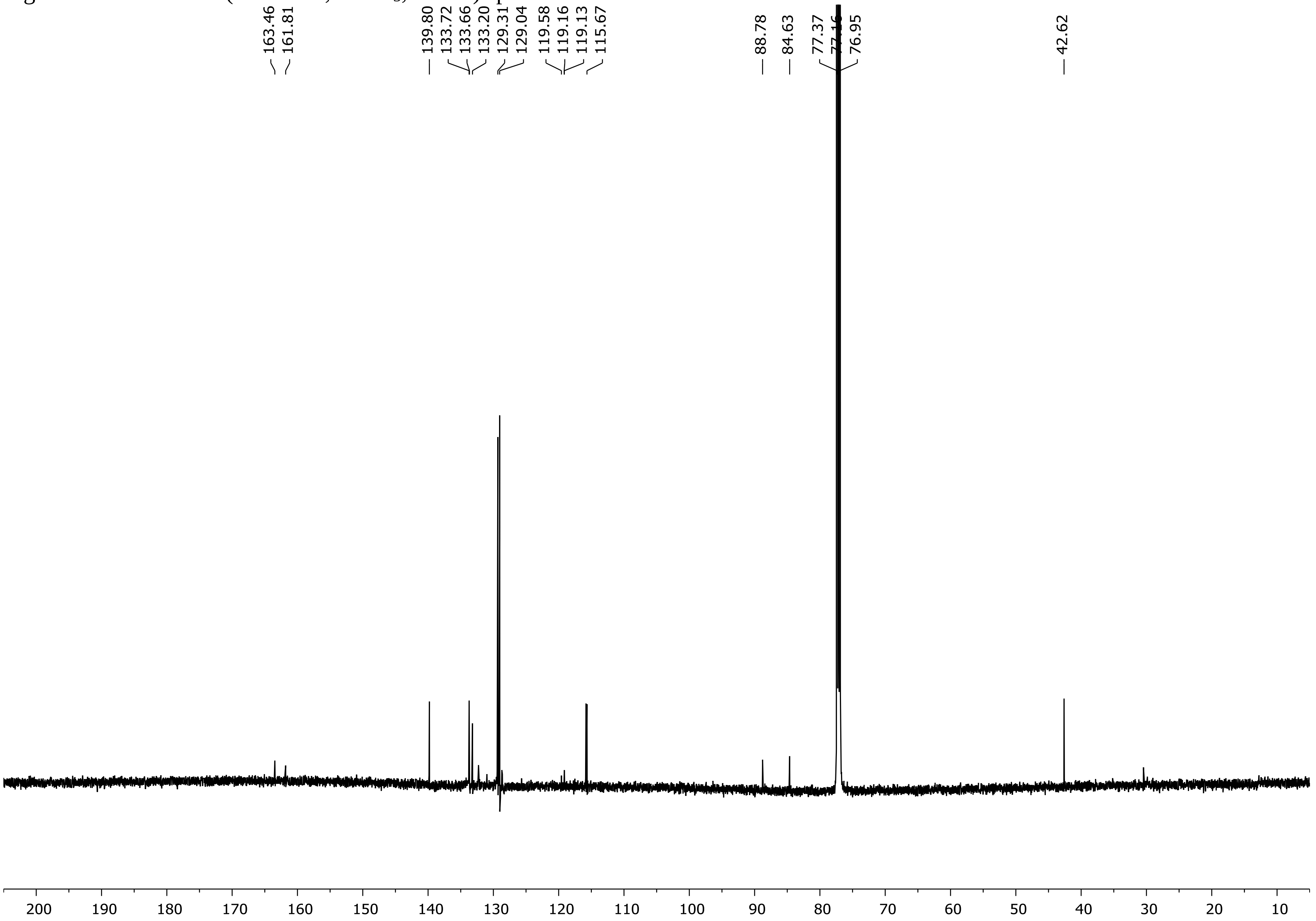

Figure S61: ^{19}**F NMR** (376 MHz, $CDCl_3$, 298 K) spectrum of **2e**.

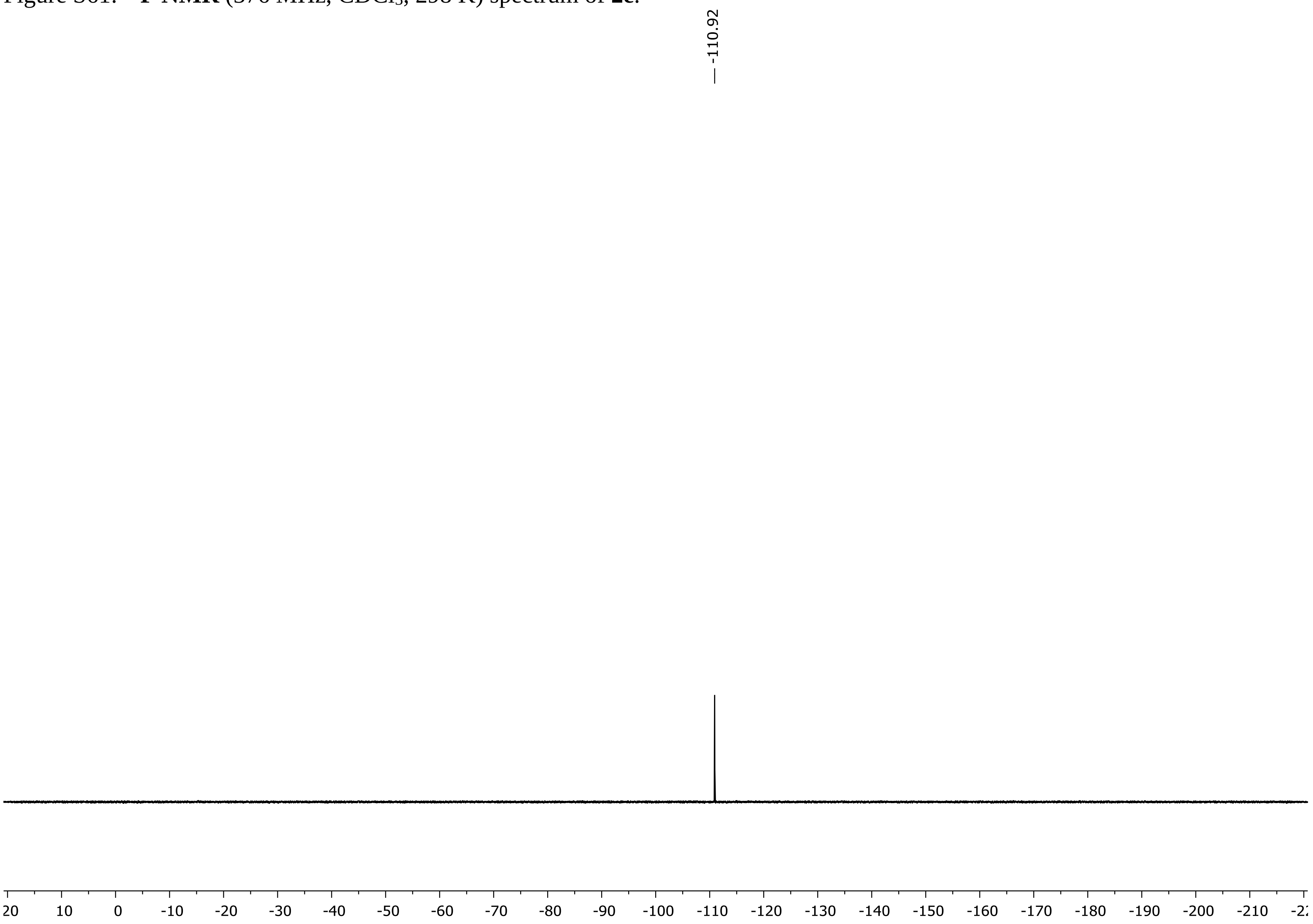

Figure S62: **^{1}H NMR** (400 MHz, $CDCl_3$, 298 K) spectrum of **2f**.

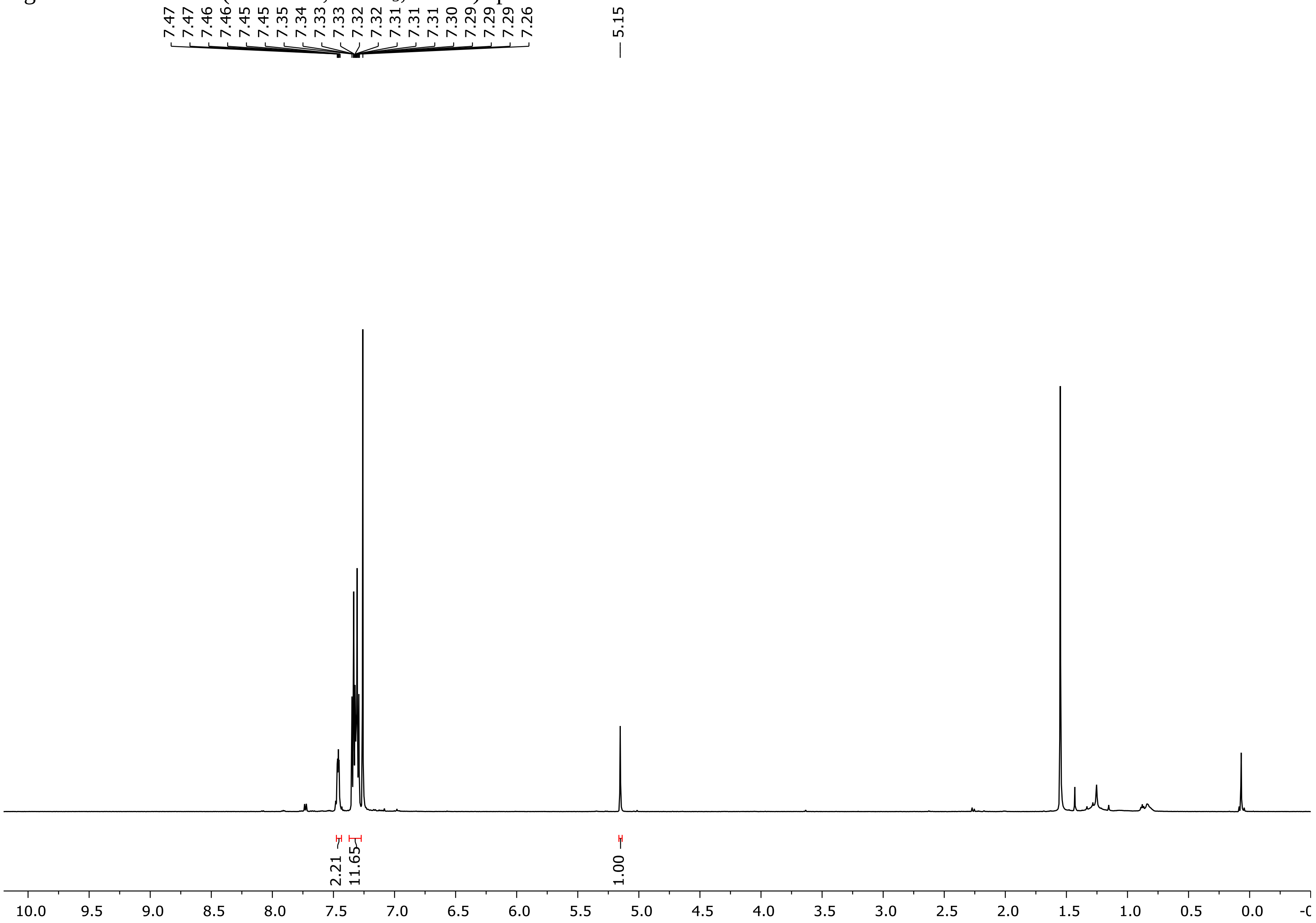

Figure S63: **^{13}C NMR** (101 MHz, $CDCl_3$, 298 K) spectrum of **2f**.

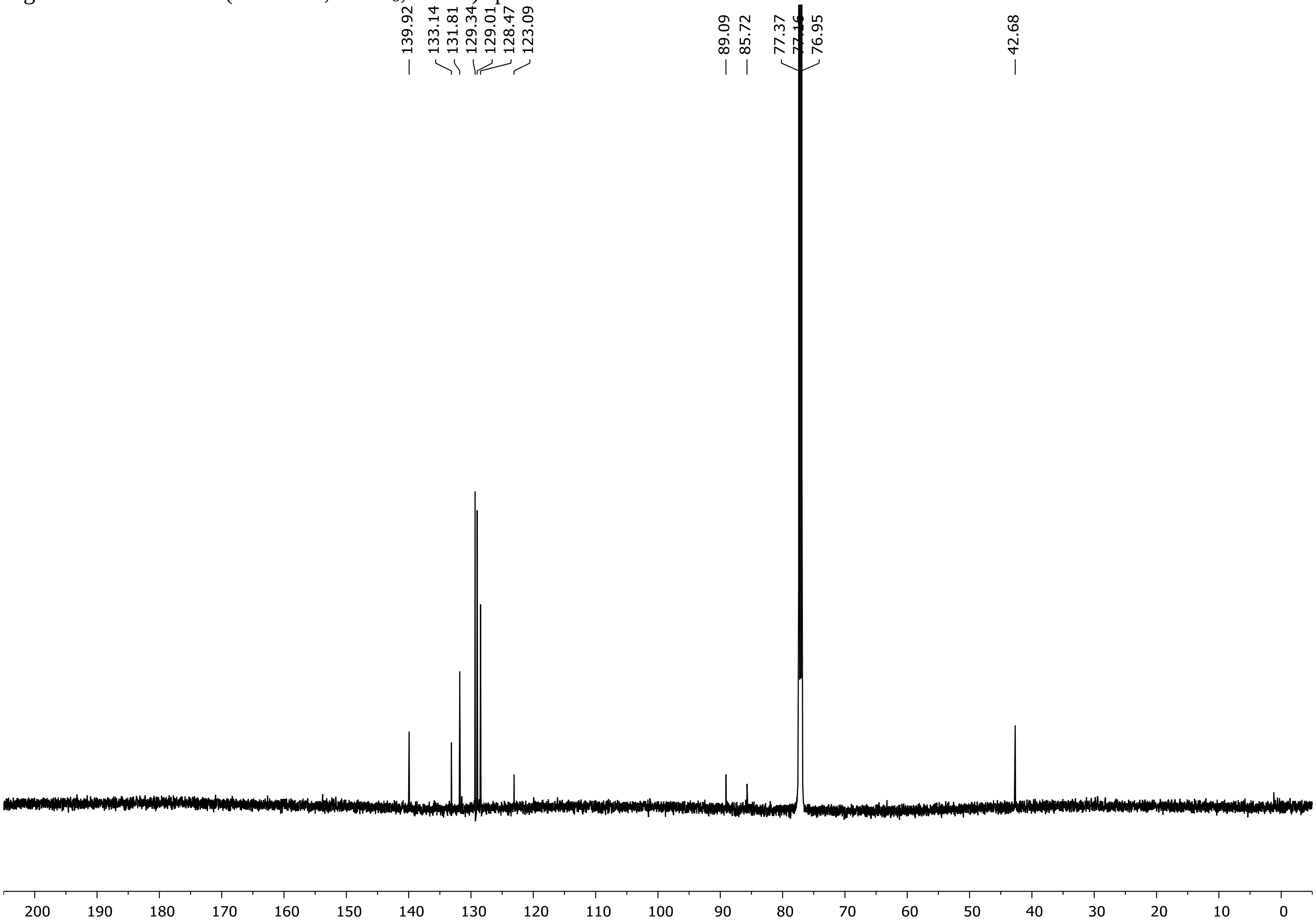

Figure S64: ^{1}**H NMR** (400 MHz, $CDCl_3$, 298 K) spectrum of **2g**.

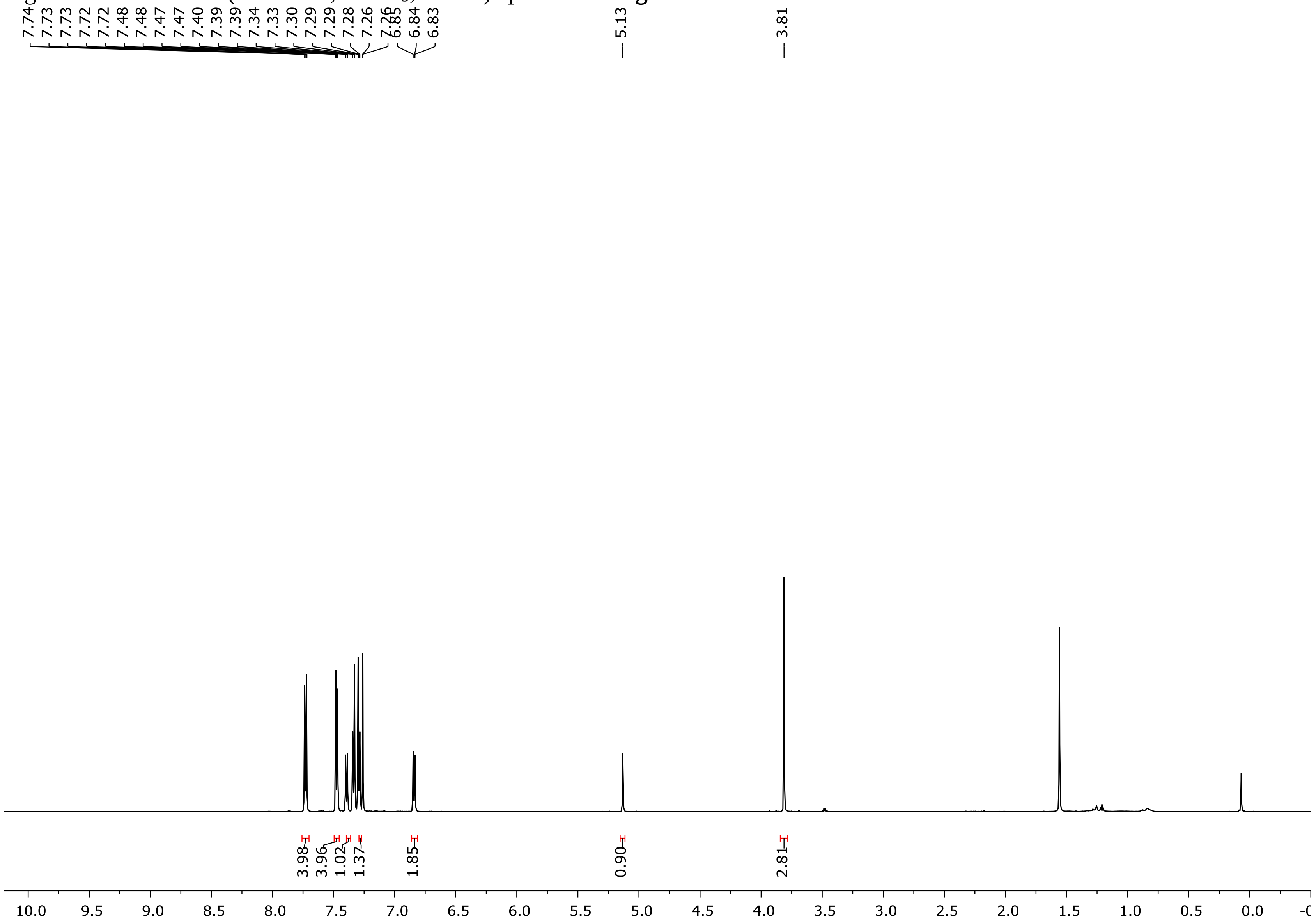

Figure S65: **^{13}C NMR** (101 MHz, $CDCl_3$, 298 K) spectrum of **2g**.

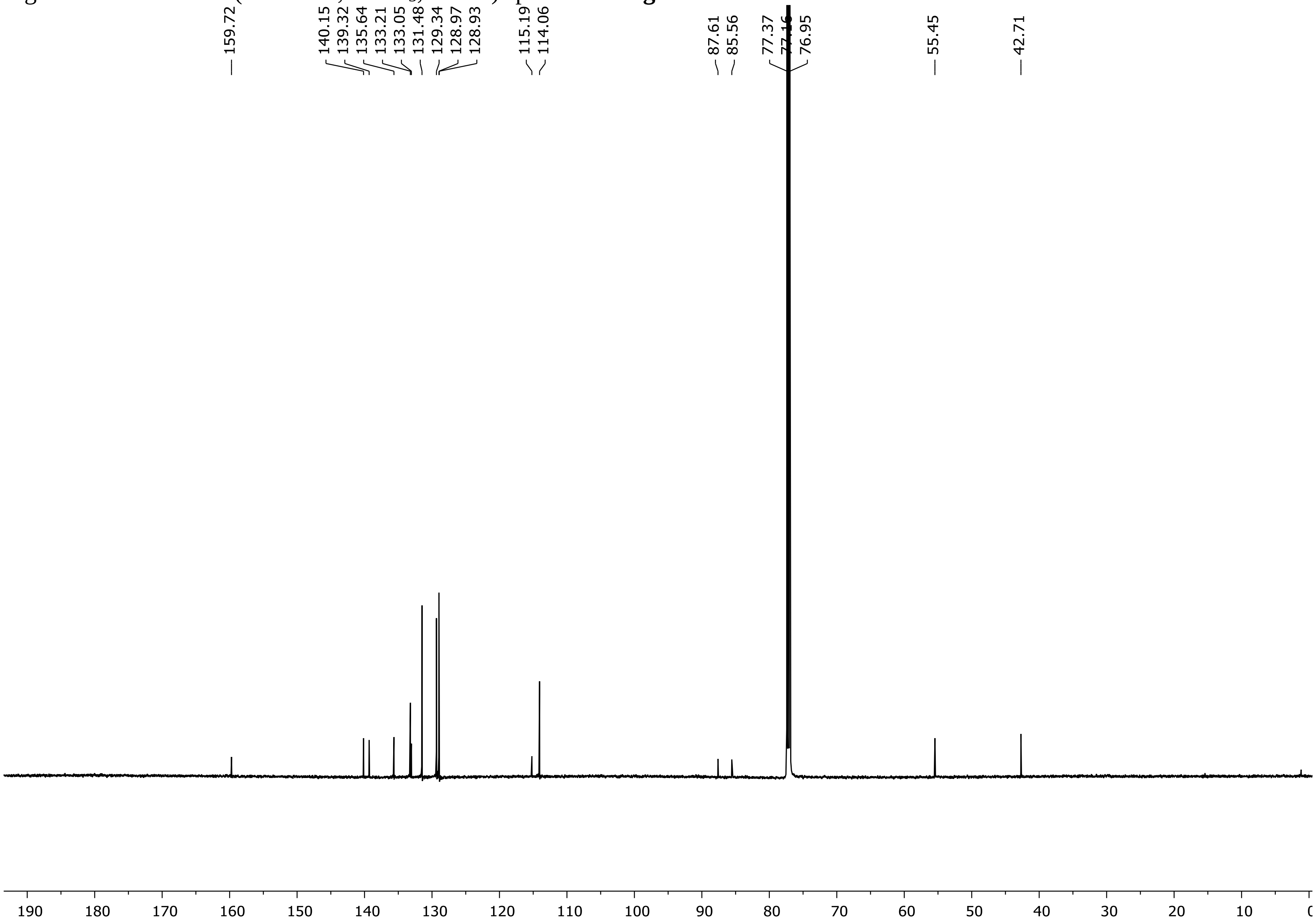

Figure S66: **^{1}H NMR** (400 MHz, $CDCl_3$, 298 K) spectrum of **2h**.

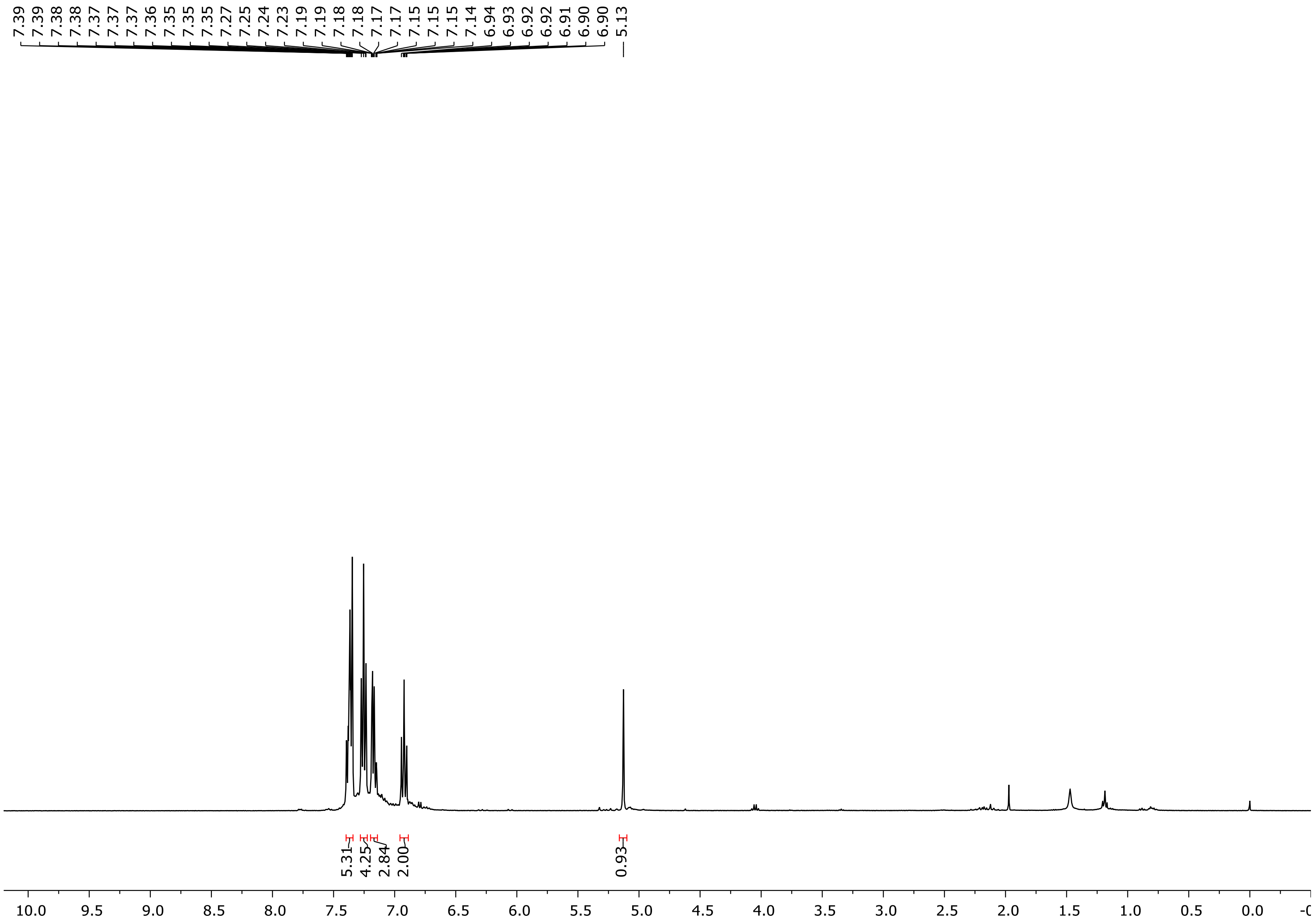

Figure S67: **^{13}C NMR** (101 MHz, $CDCl_3$, 298 K) spectrum of **2h**.

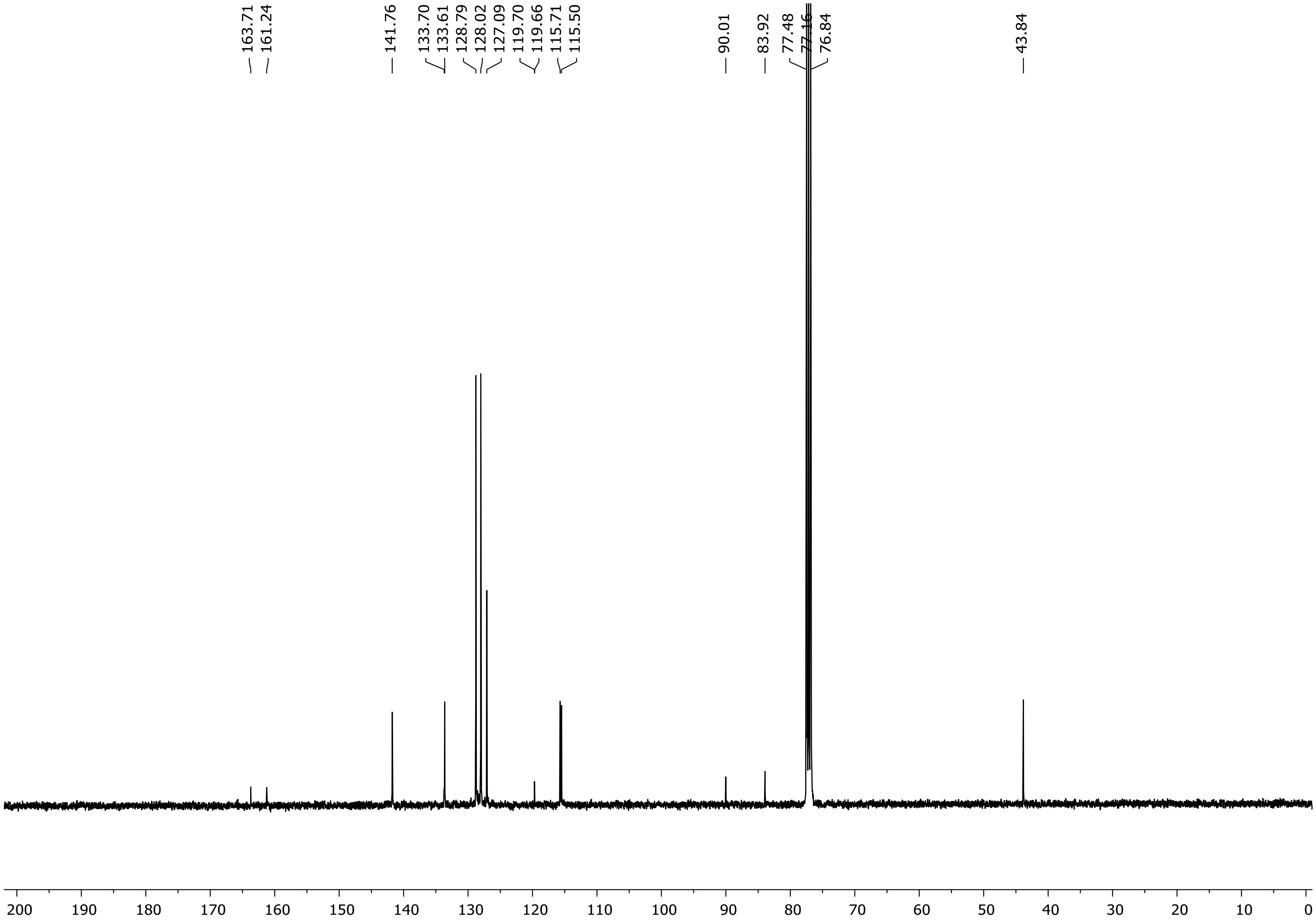

Figure S68: **^{19}F NMR** (376 MHz, $CDCl_3$, 298 K) spectrum of **2h**.

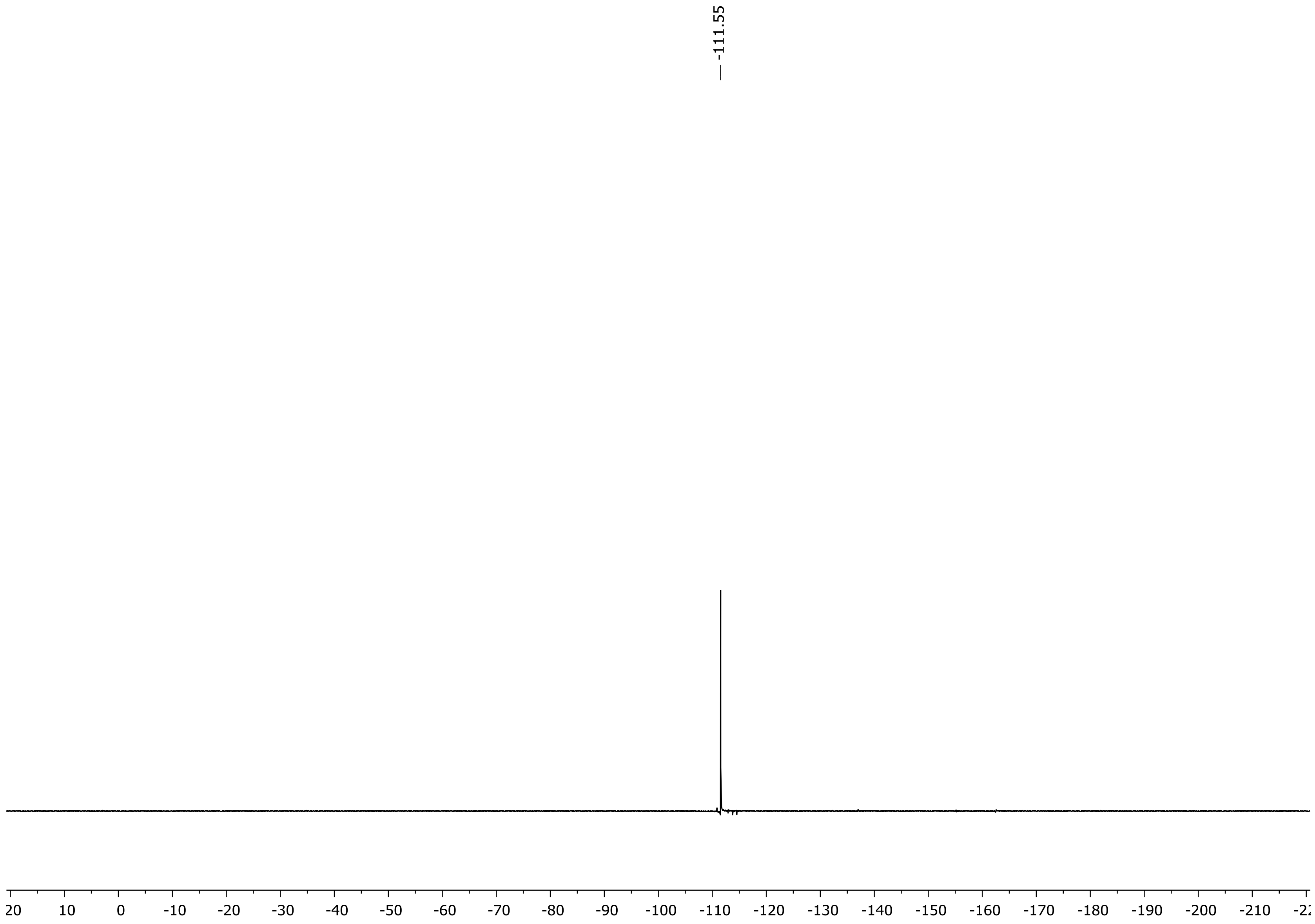

Figure S69: **^{1}H NMR** (500 MHz, $CDCl_3$, 298 K) spectrum of **2i**.

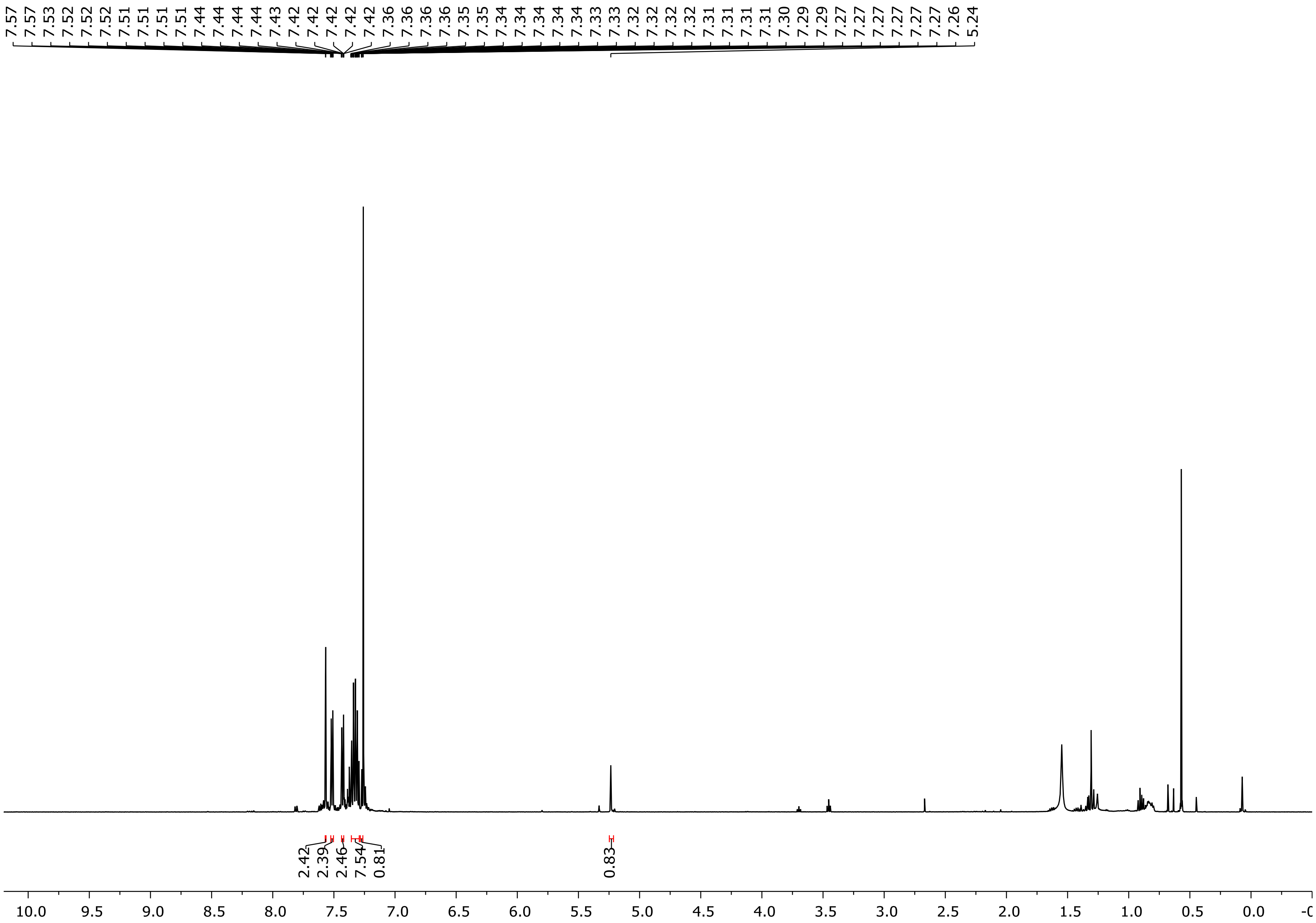

Figure S70: **^{13}C NMR** (126 MHz, $CDCl_3$, 298 K) spectrum of **2i**.

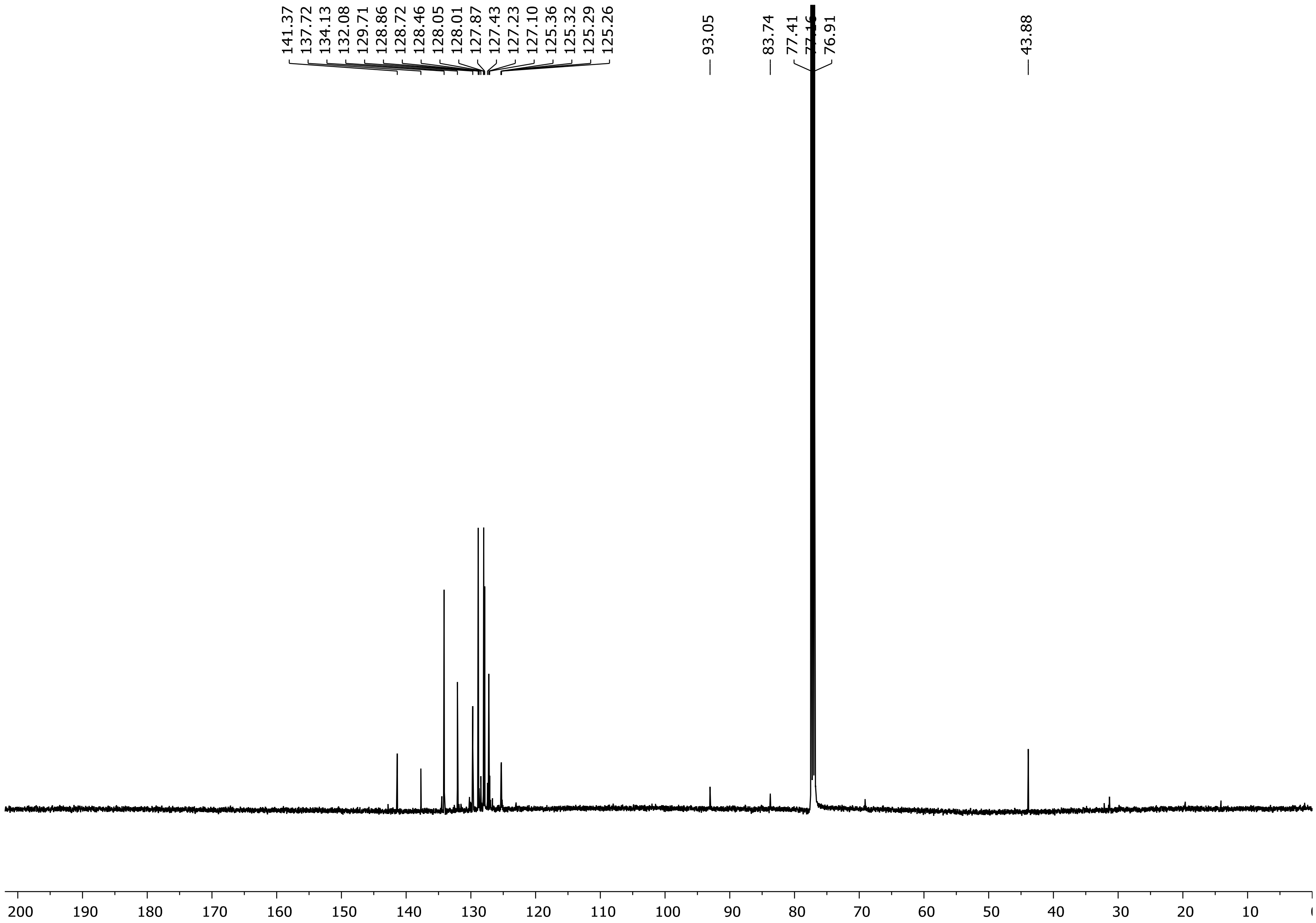

Figure S71: ^{19}**F NMR** (471 MHz, $CDCl_3$, 298 K) spectrum of **2i**.

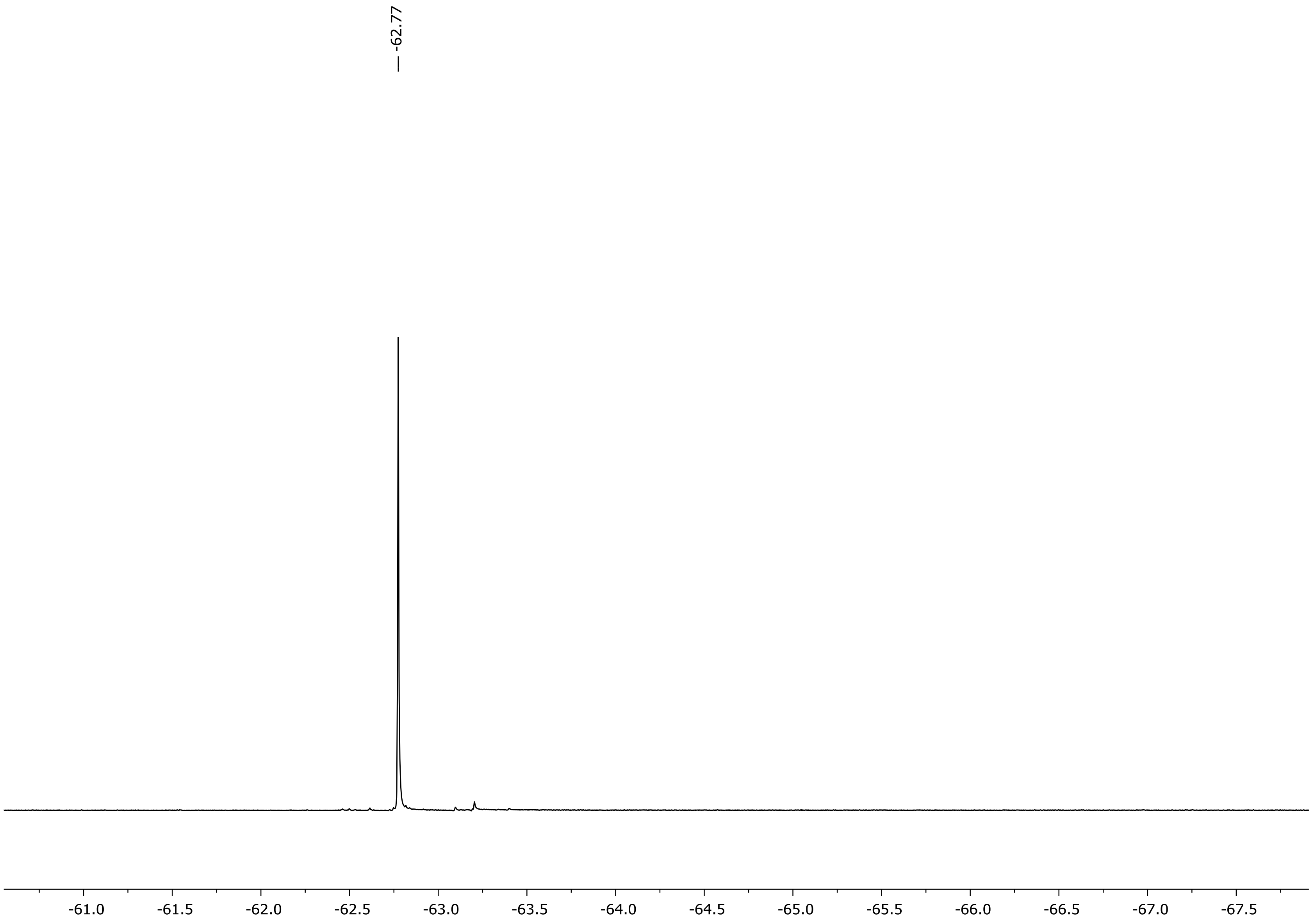

Figure S72: **^{1}H NMR** (400 MHz, $CDCl_3$, 298 K) spectrum of **2j**.

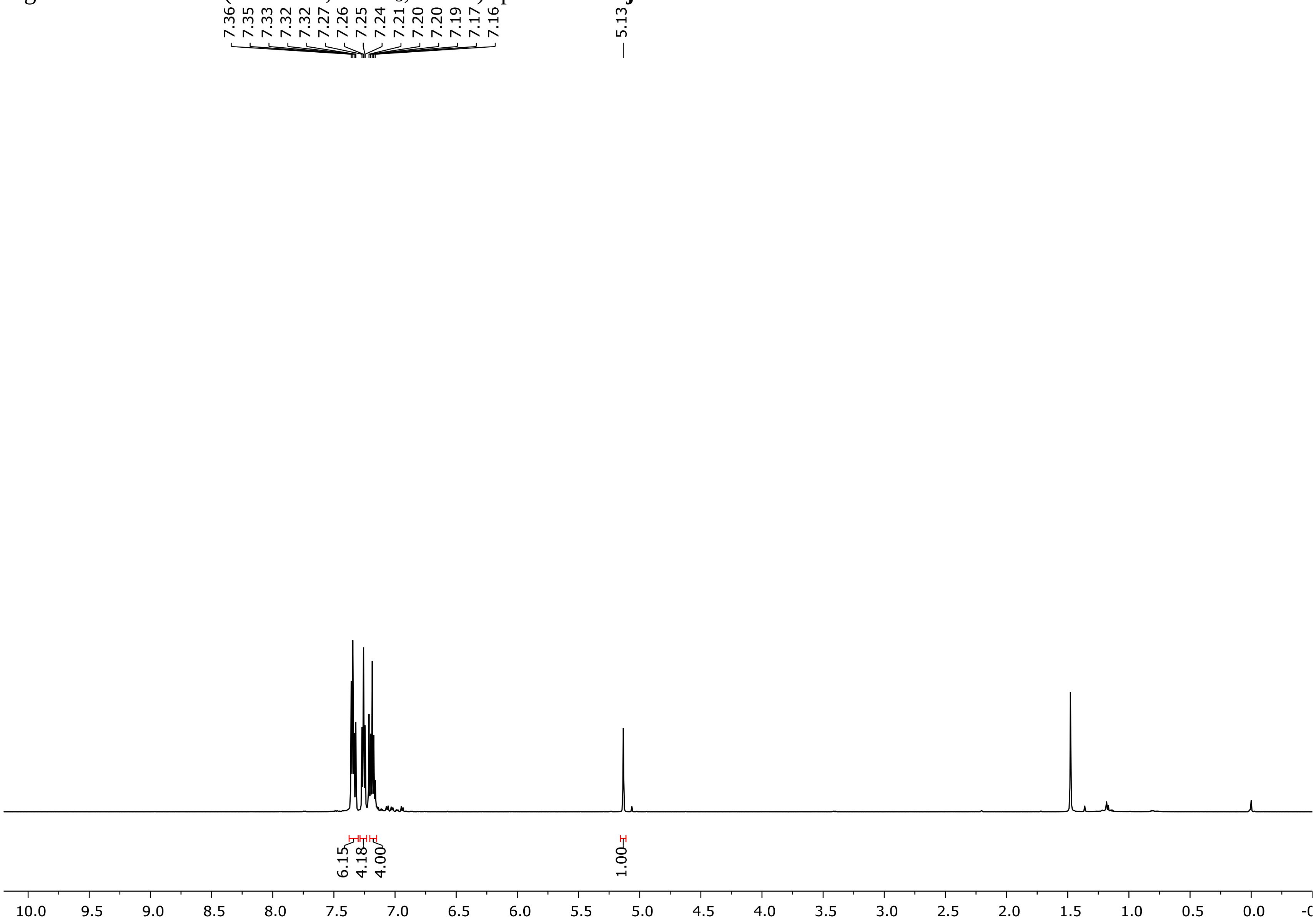

Figure S73: **^{13}C NMR** (126 MHz, $CDCl_3$, 298 K) spectrum of **2j**.

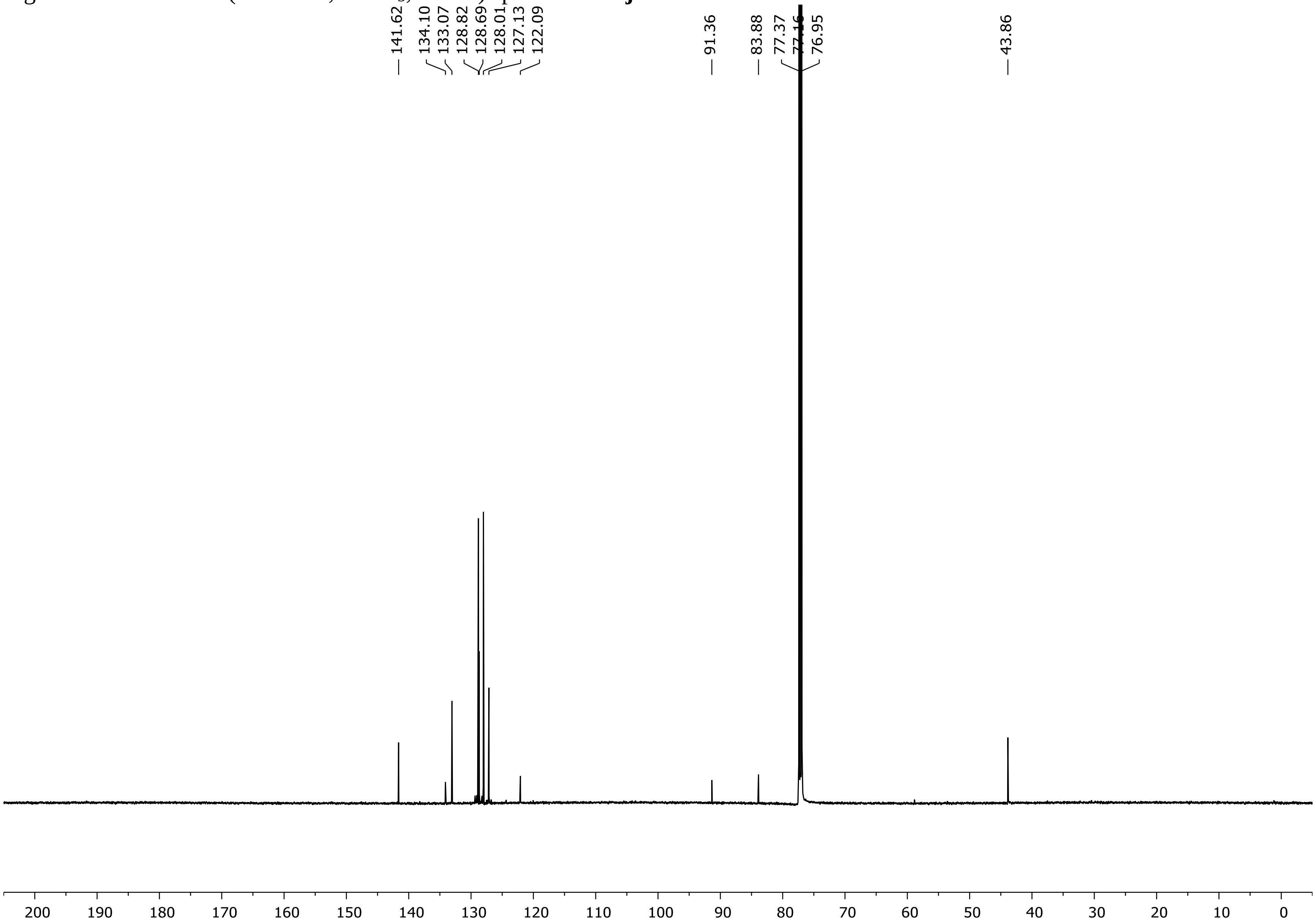

Figure S74: **^{1}H NMR** (400 MHz, $CDCl_3$, 298 K) spectrum of **2k**.

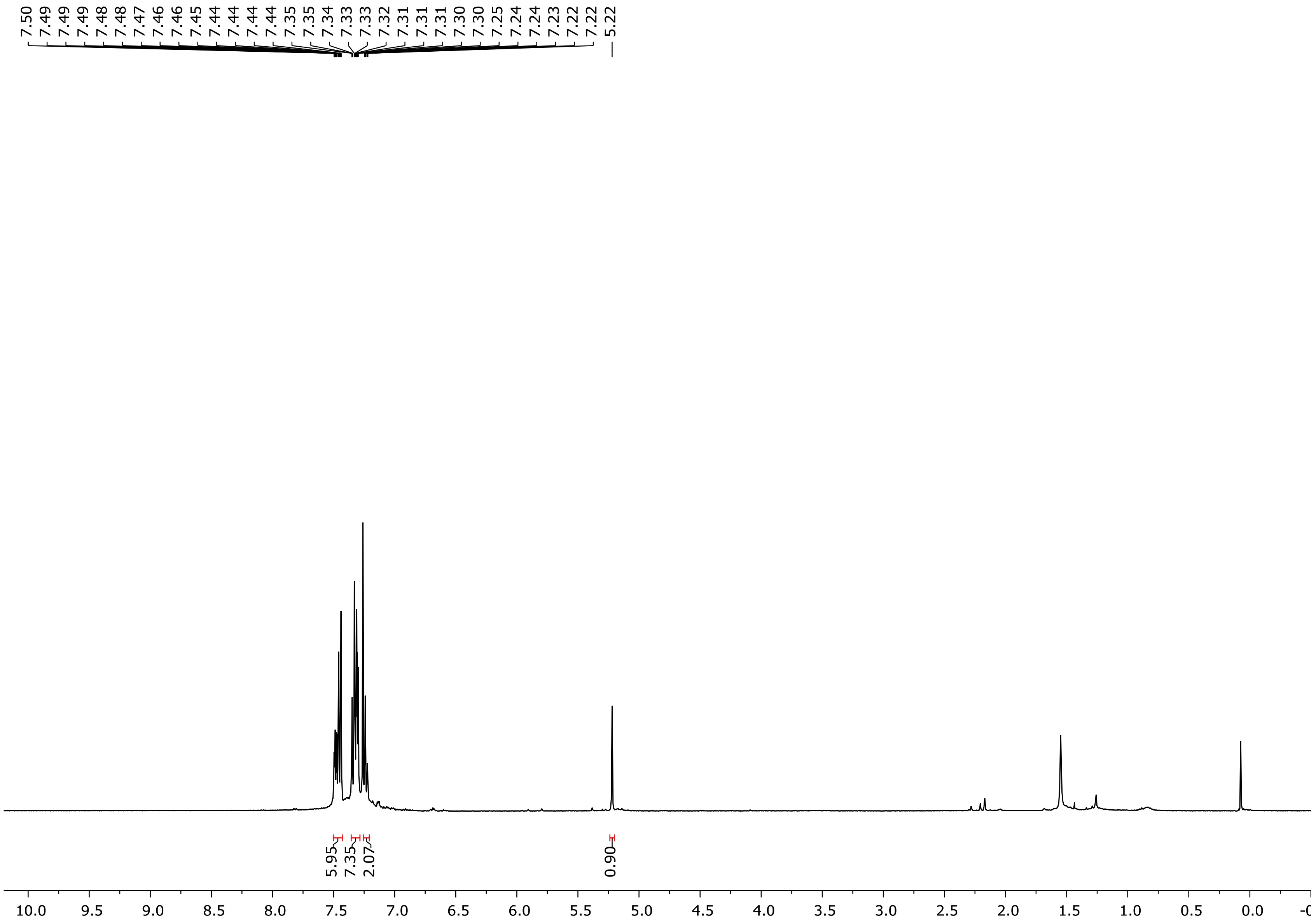

Figure S75: **^{13}C NMR** (101 MHz, $CDCl_3$, 298 K) spectrum of **2k**.

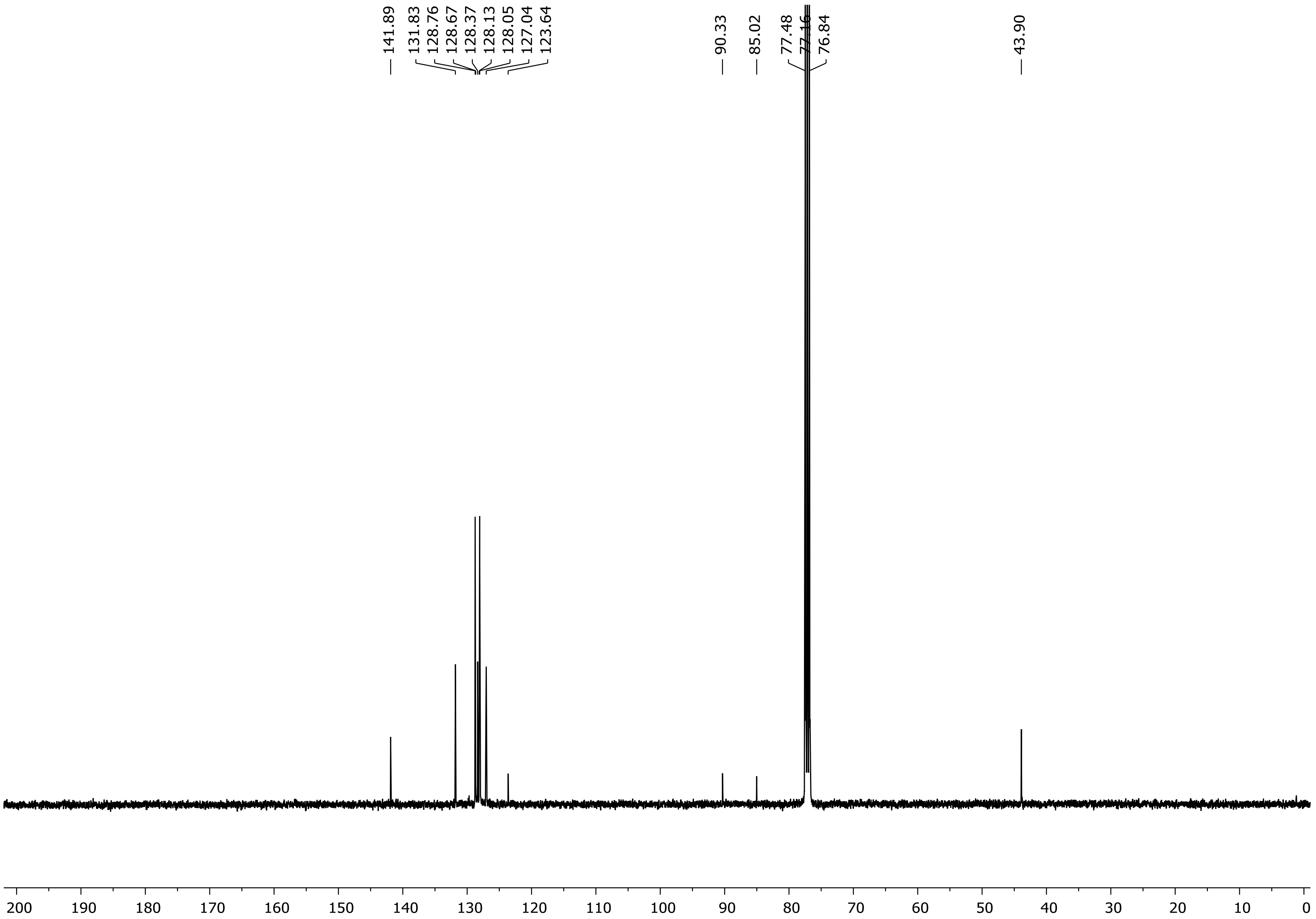

Figure S76: ^{1}**H NMR** (500 MHz, $CDCl_3$, 298 K) spectrum of **2l**.

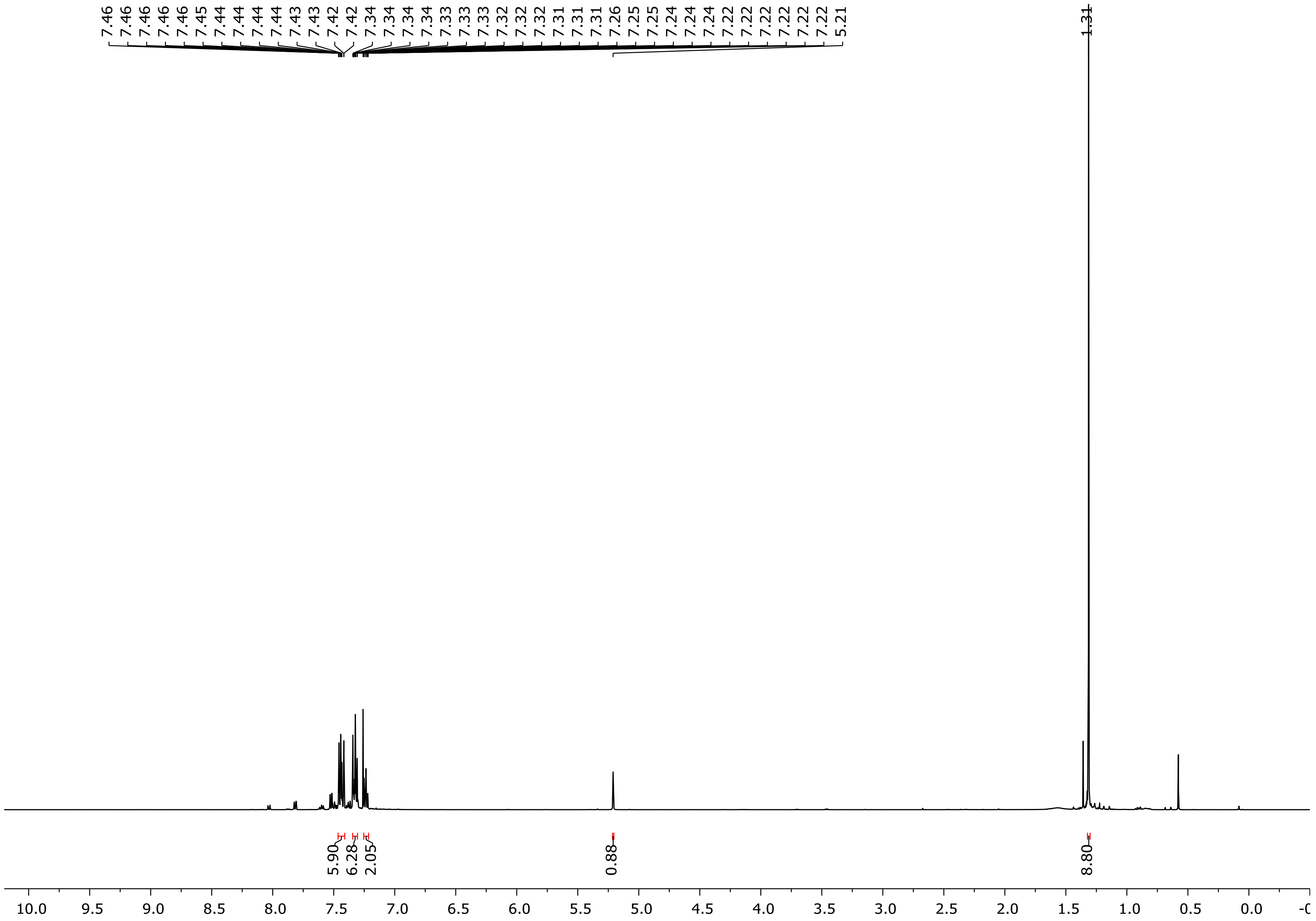

Figure S77: **^{13}C NMR** (126 MHz, $CDCl_3$, 298 K) spectrum of **2l**.

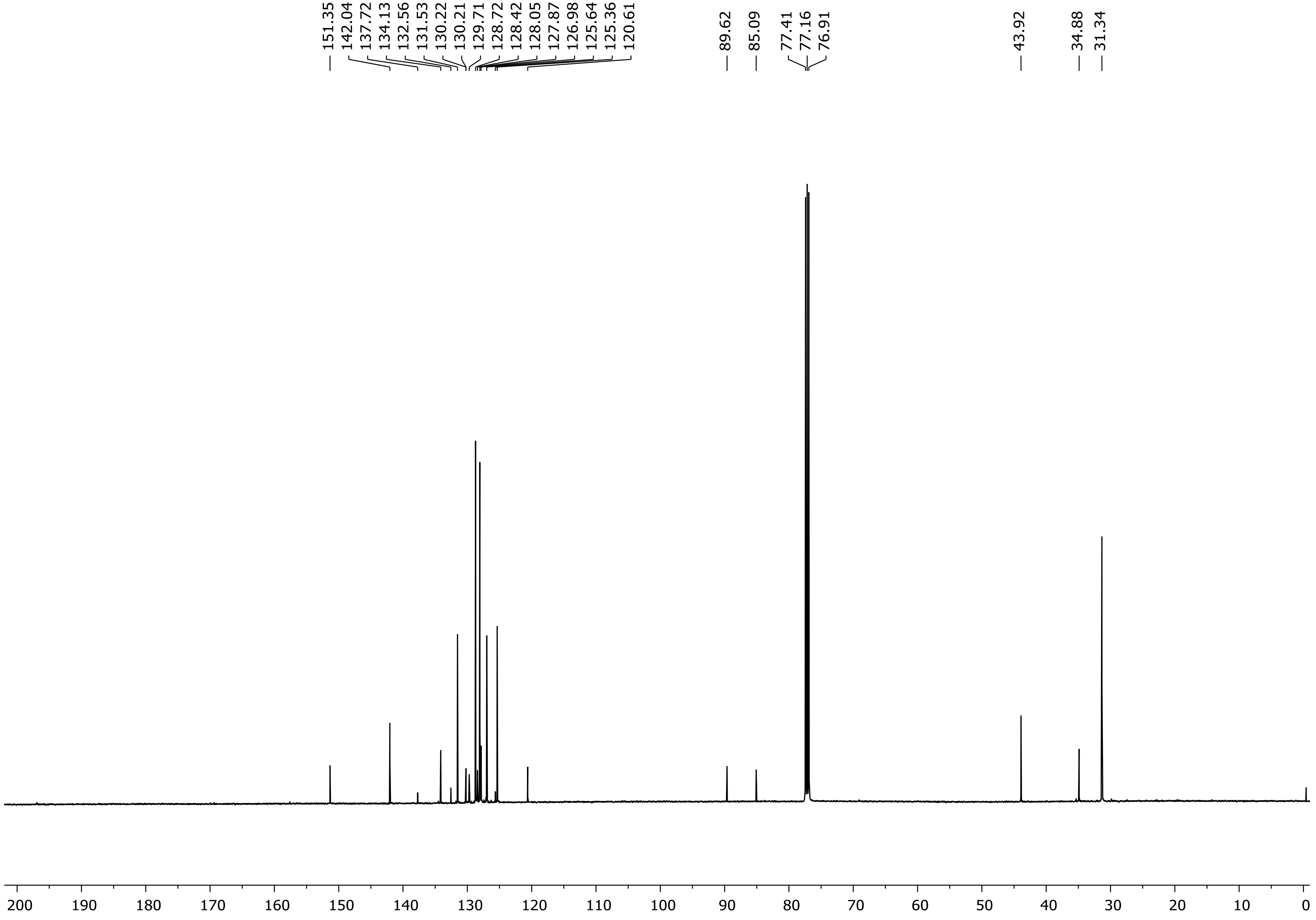

Figure S78: **^{1}H NMR** (400 MHz, $CDCl_3$, 298 K) spectrum of **2m**.

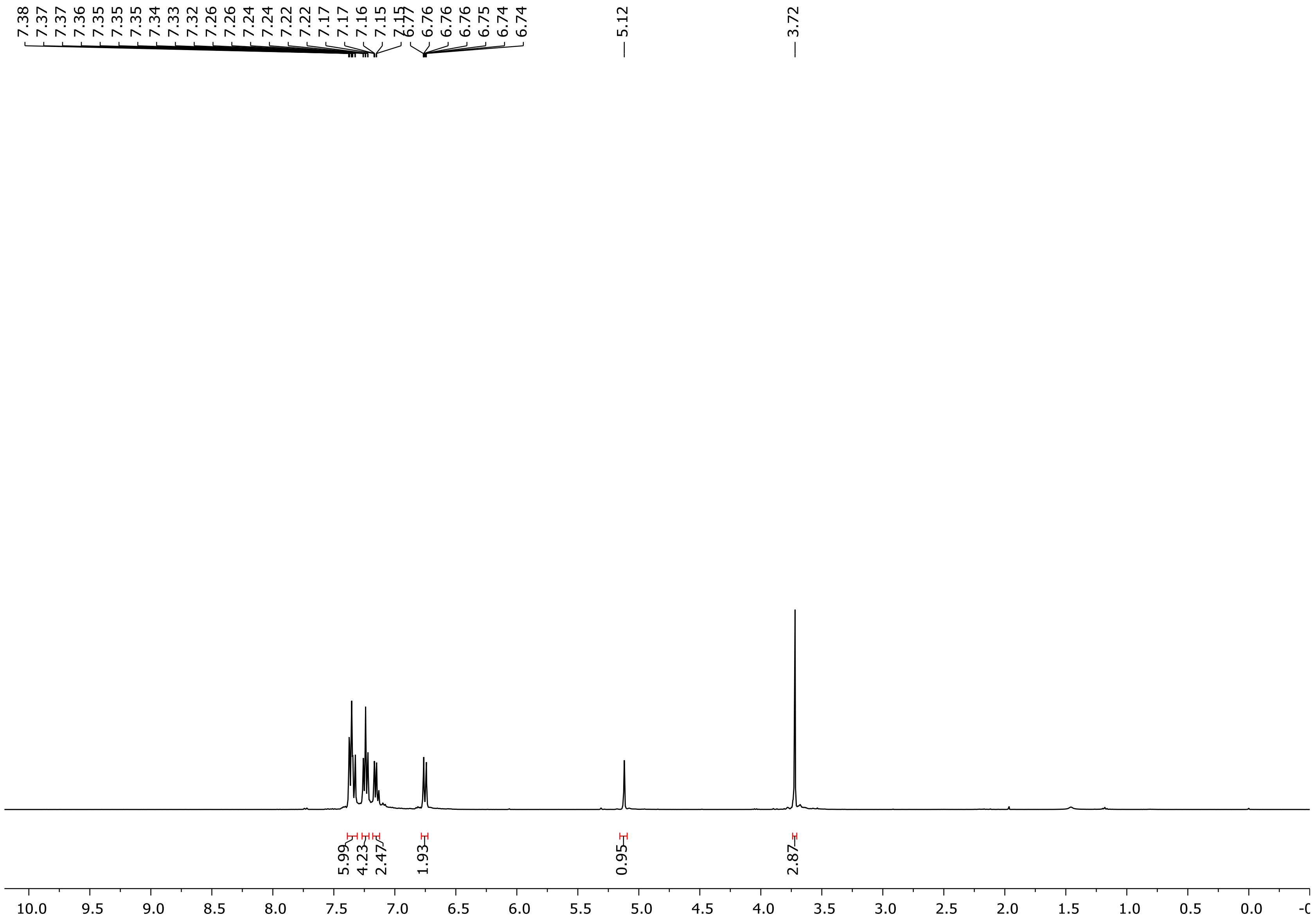

Figure S79: **^{13}C NMR** (101 MHz, $CDCl_3$, 298 K) spectrum of **2m**.

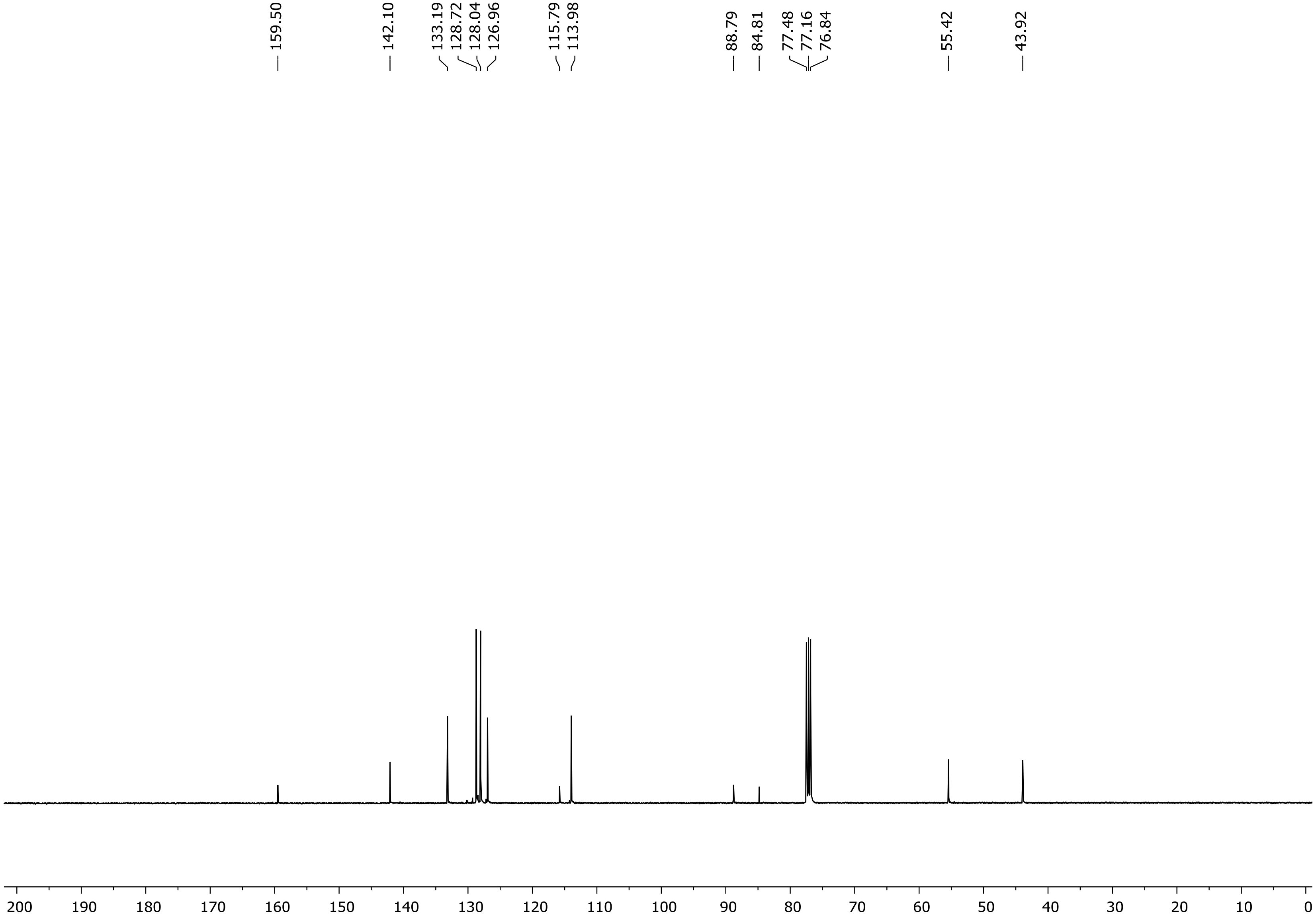

Figure S80: **^{1}H NMR** (500 MHz, $CDCl_3$, 298 K) spectrum of **2n**.

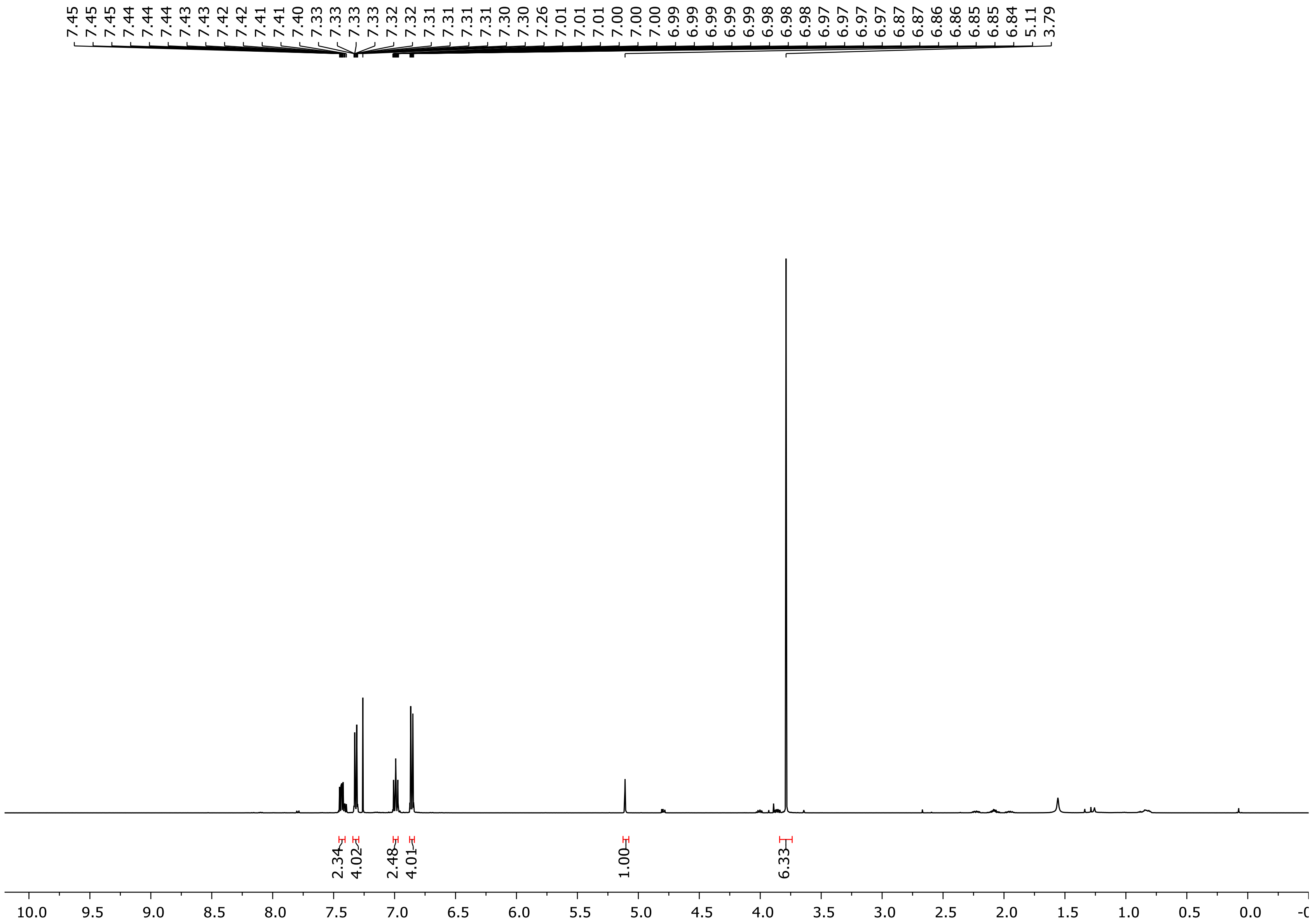

Figure S81: **^{13}C NMR** (126 MHz, $CDCl_3$, 298 K) spectrum of **2n**.

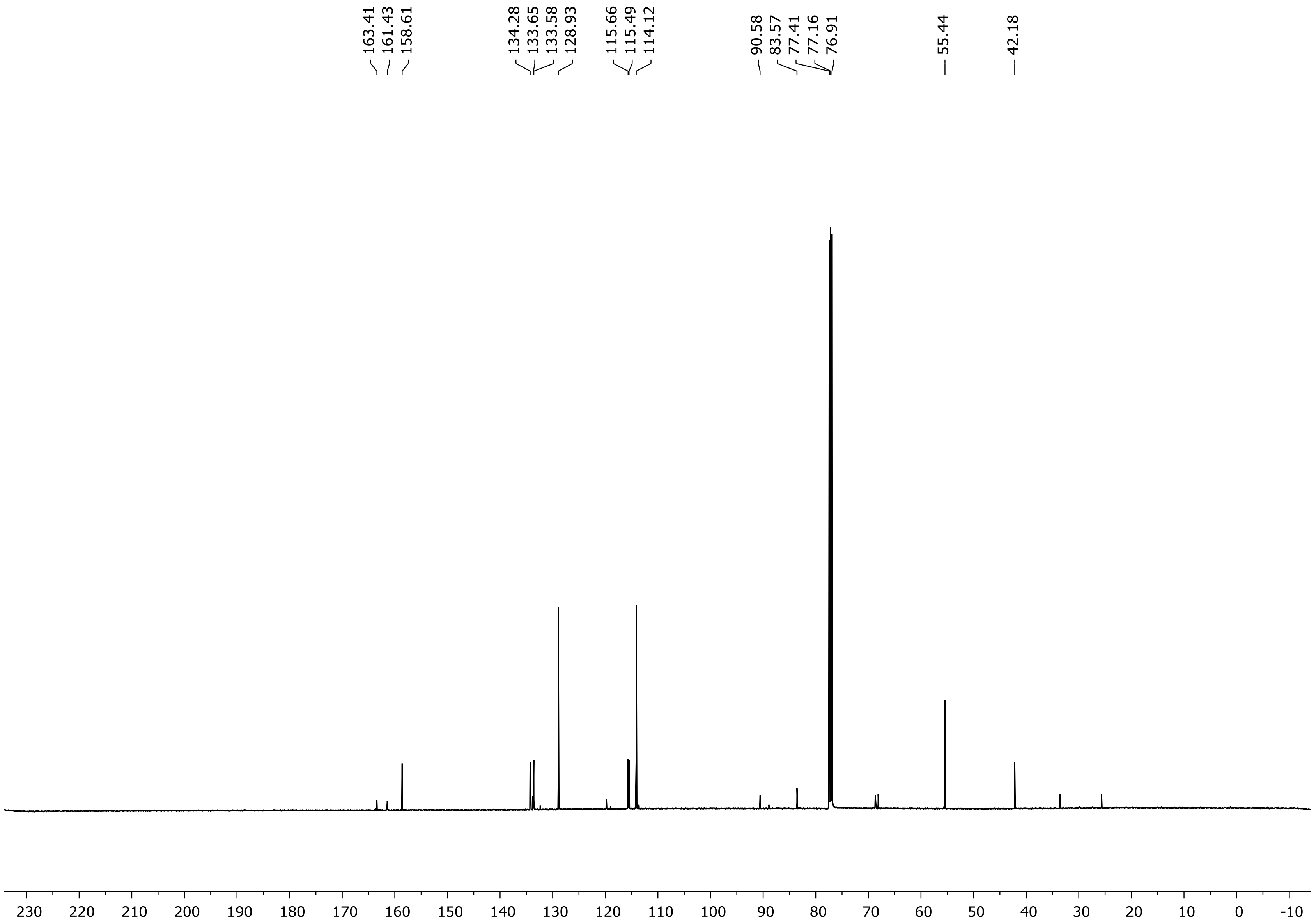

Figure S82: **^{19}F NMR** (471MHz, $CDCl_3$, 298 K) spectrum of **2n**.

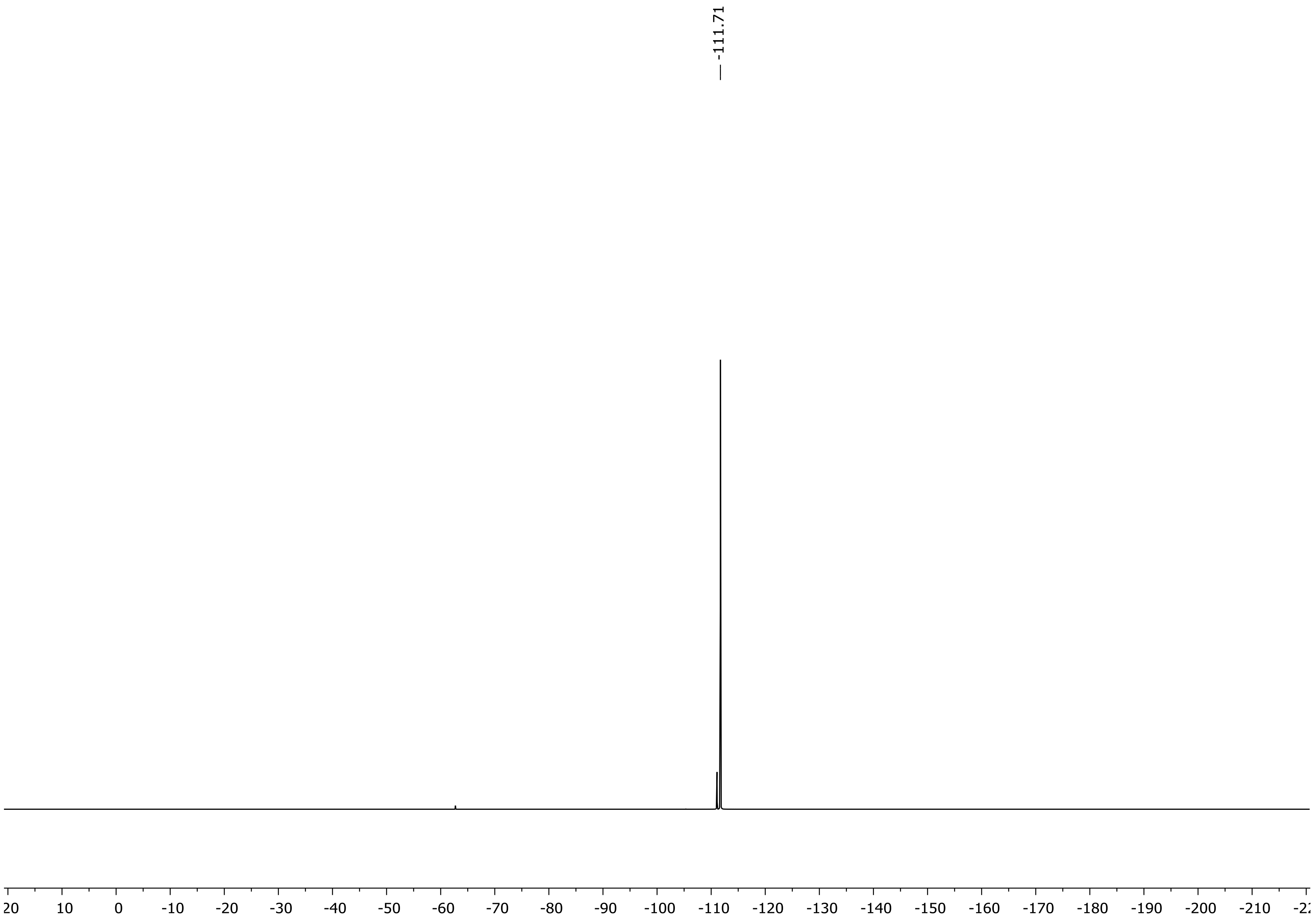

Figure S83: **^{1}H NMR** (500 MHz, $CDCl_3$, 298 K) spectrum of **2o**.

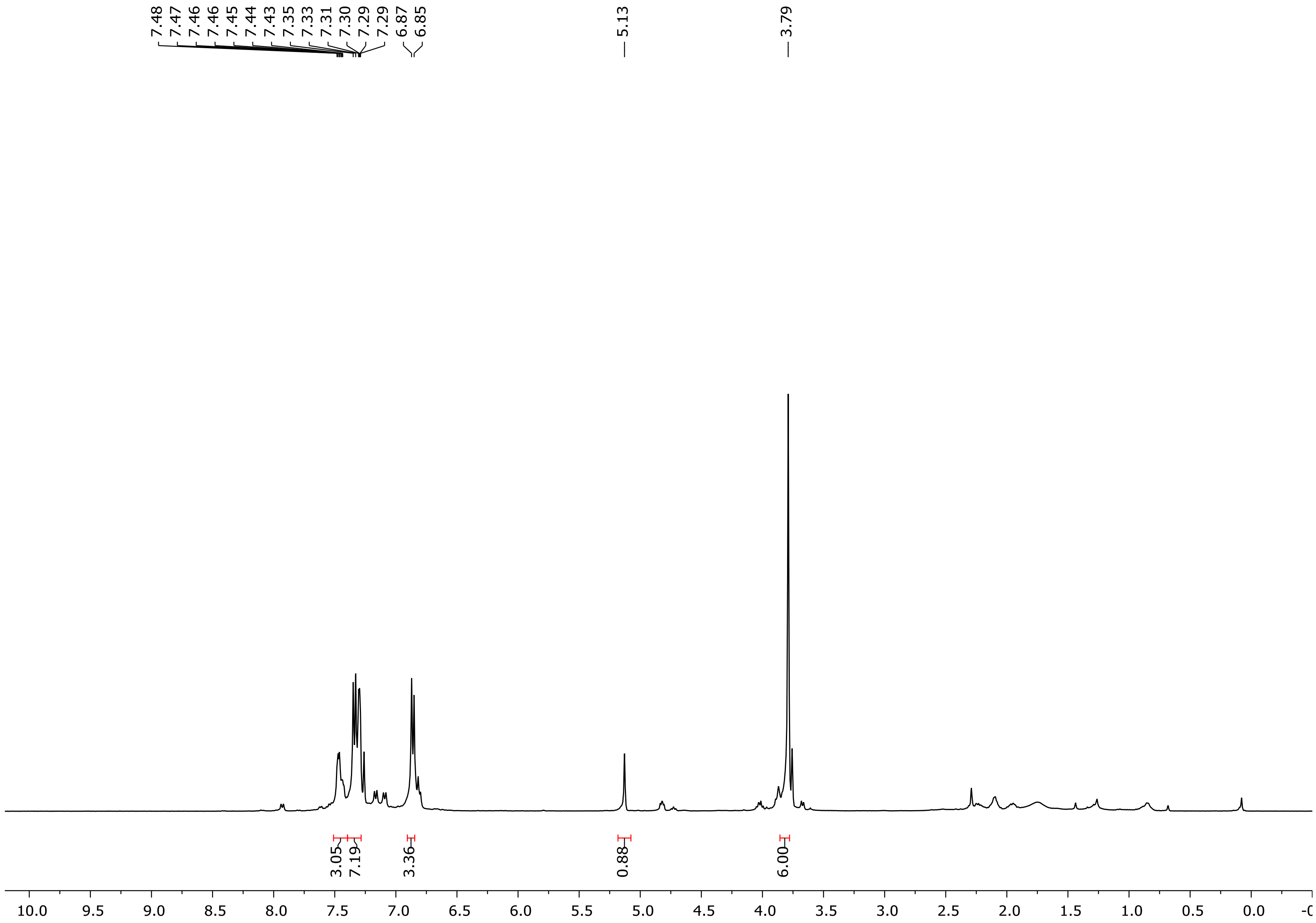

Figure S84: **^{13}C NMR** (126 MHz, $CDCl_3$, 298 K) spectrum of **2o**.

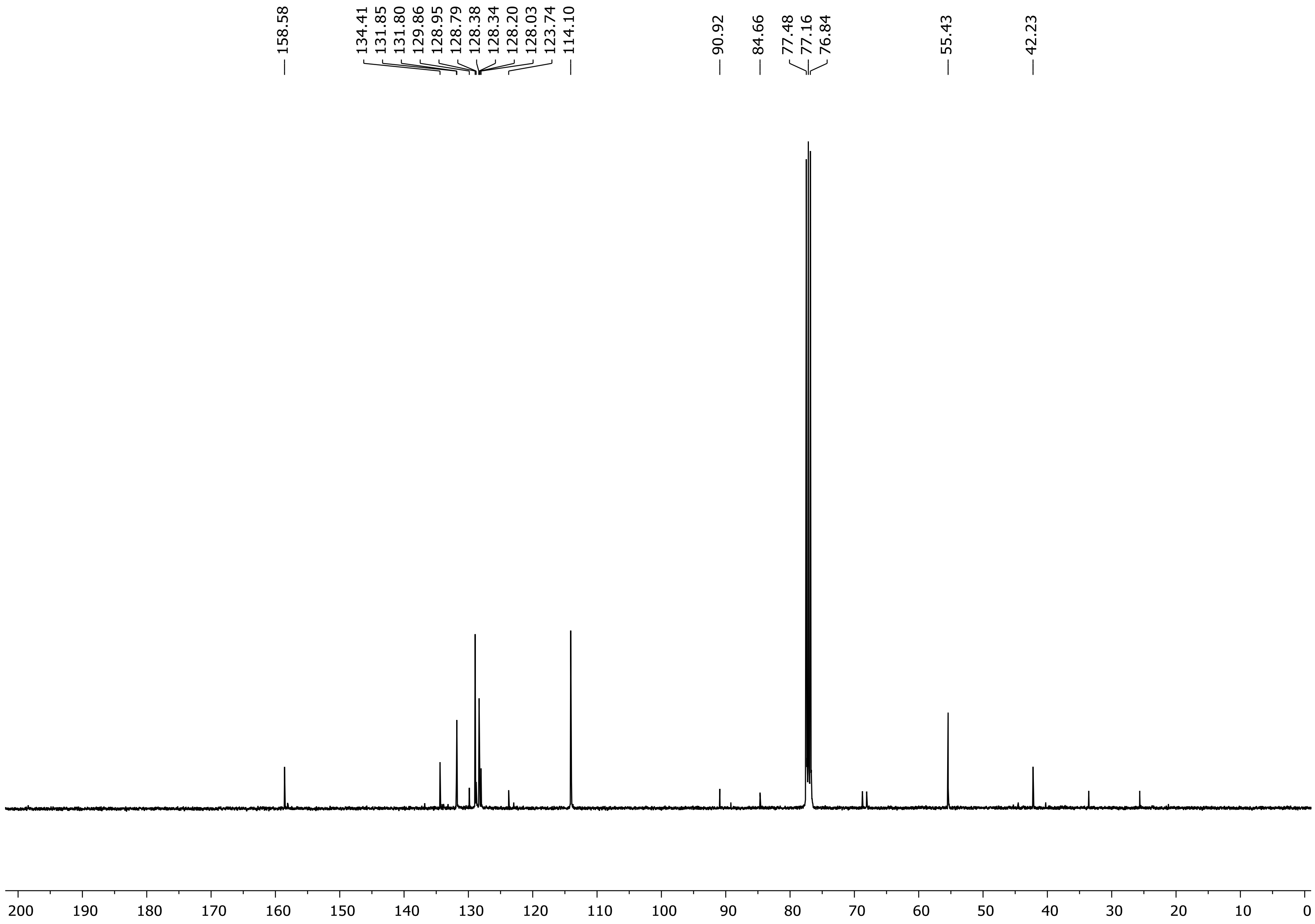

Figure S85: **^{1}H NMR** (500 MHz, $CDCl_3$, 298 K) spectrum of **2p**.

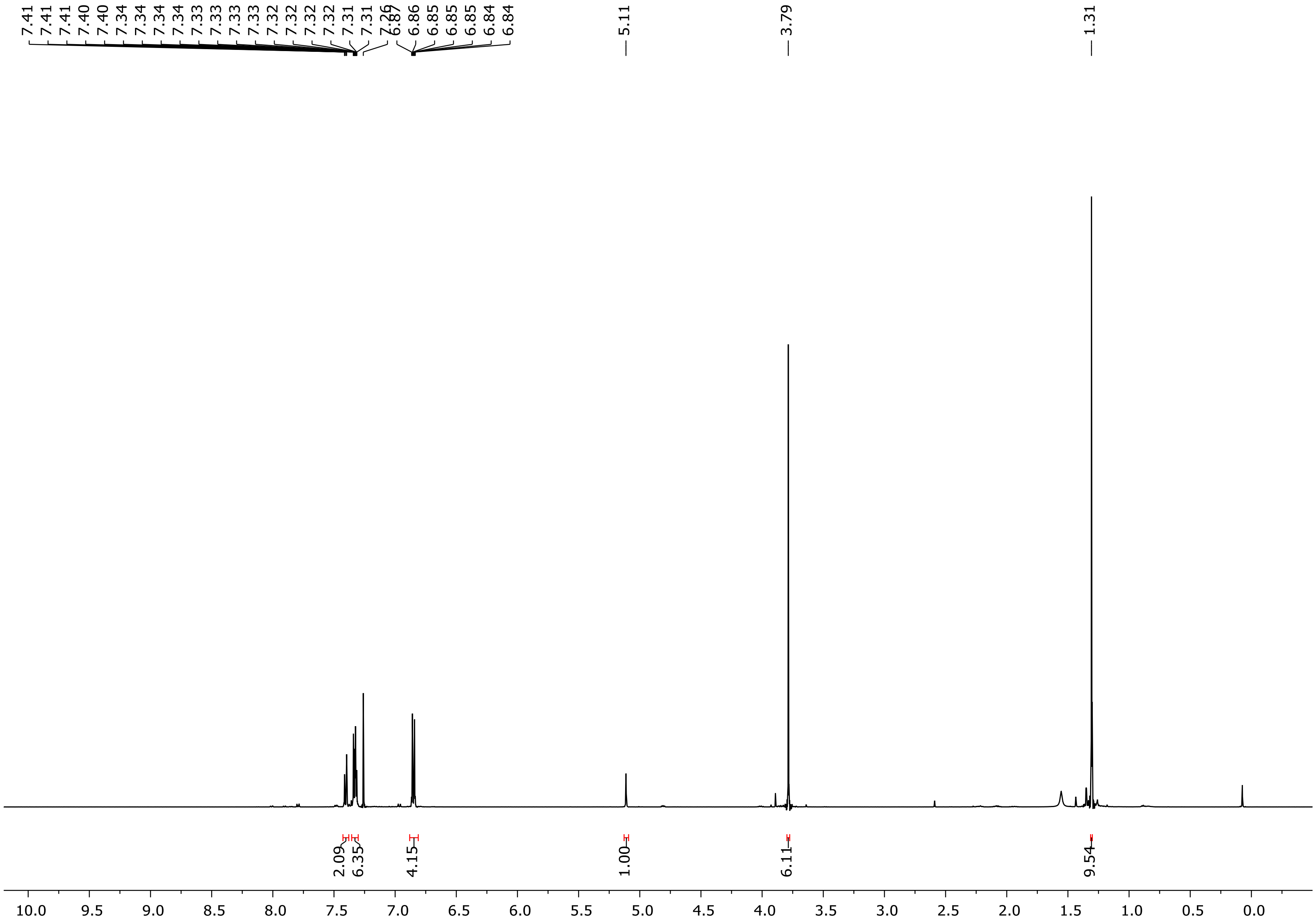

Figure S86: **^{13}C NMR** (126 MHz, $CDCl_3$, 298 K) spectrum of **2p**.

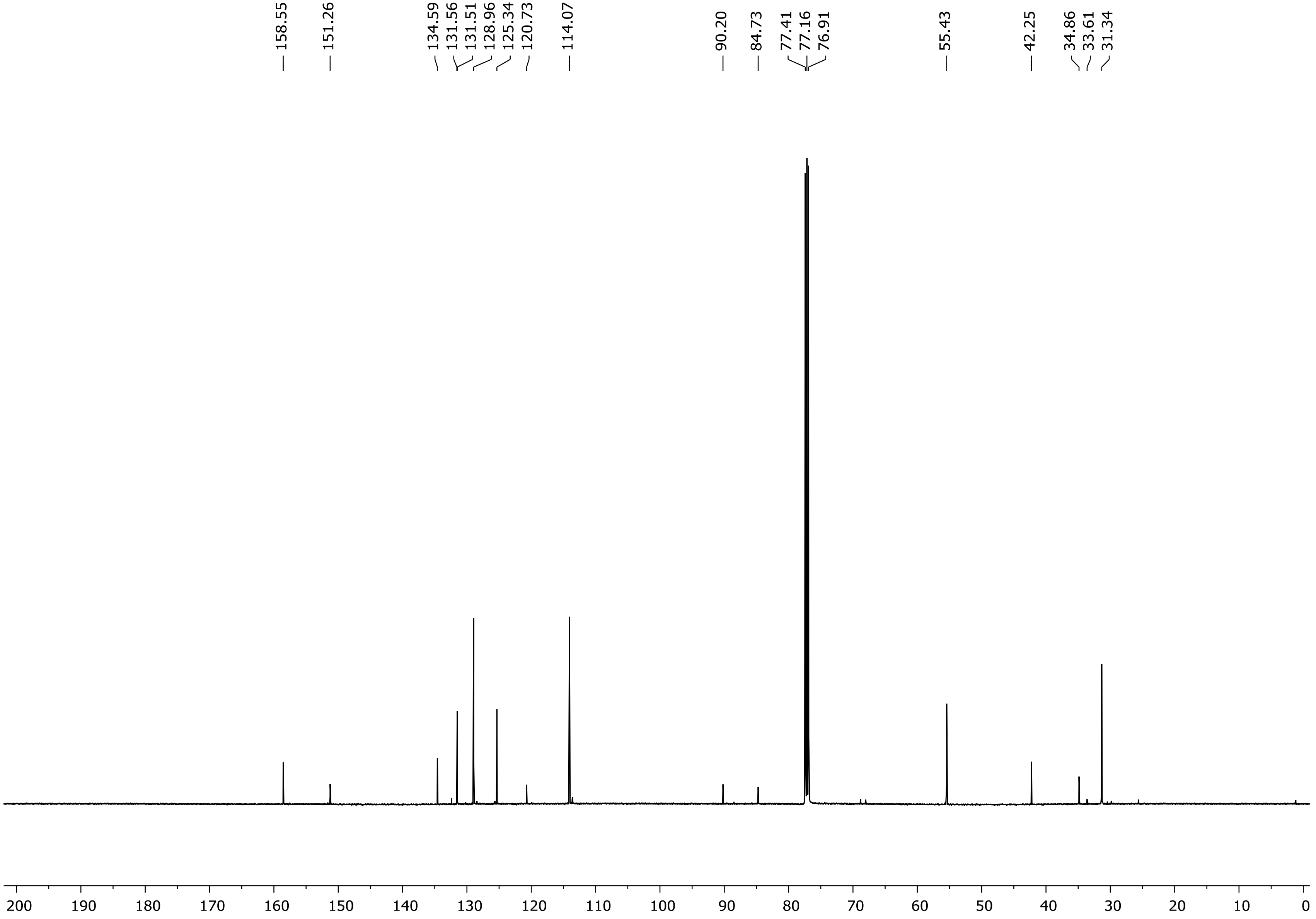

Figure S87: **^{1}H NMR** (400 MHz, $CDCl_3$, 298 K) spectrum of **2q**.

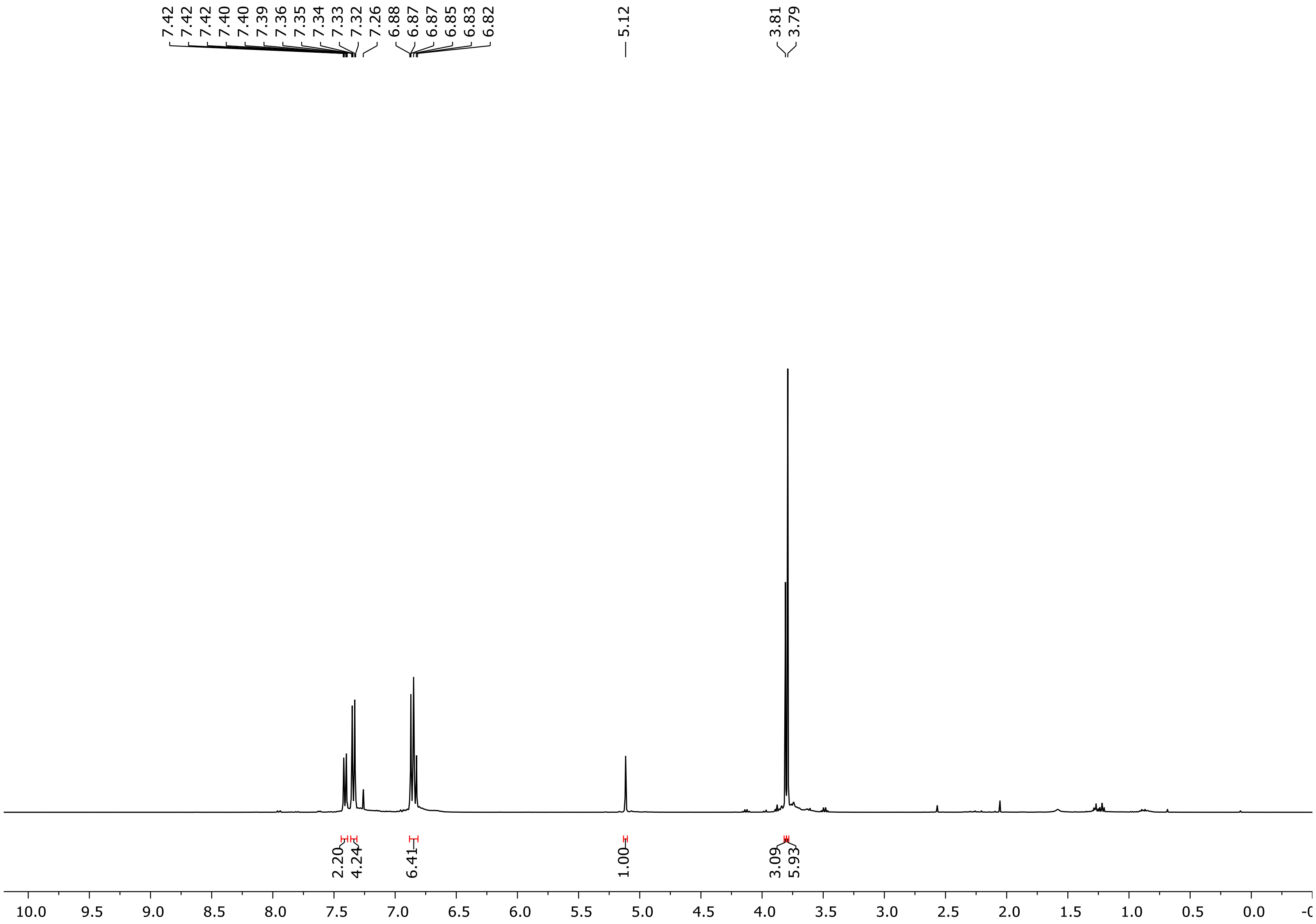

Figure S88: **^{13}C NMR** (101 MHz, CDCl$_3$, 298 K) spectrum of **2q**.

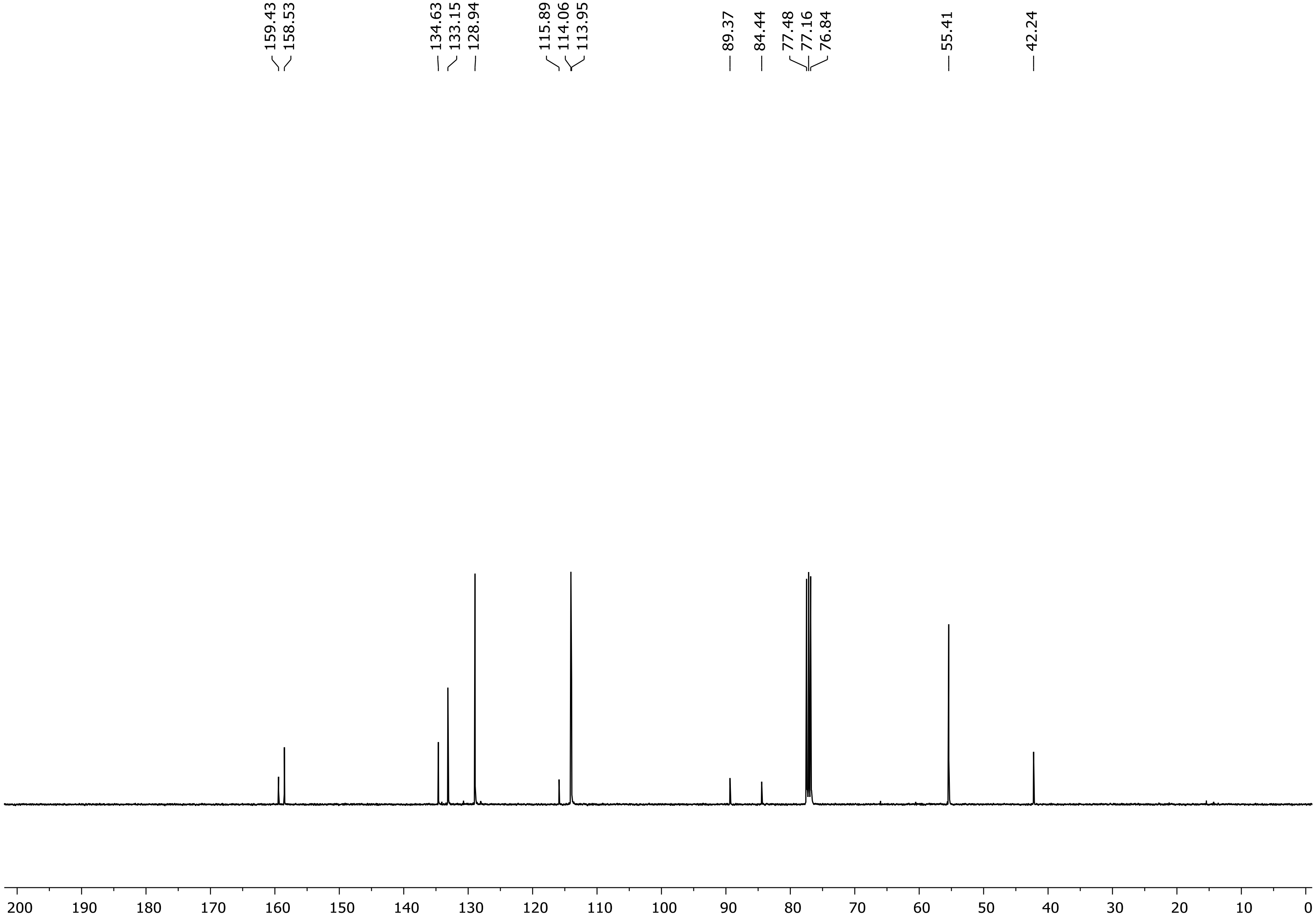

Figure S89: **^{1}H NMR** (400 MHz, $CDCl_3$, 298 K) spectrum of **2r**.

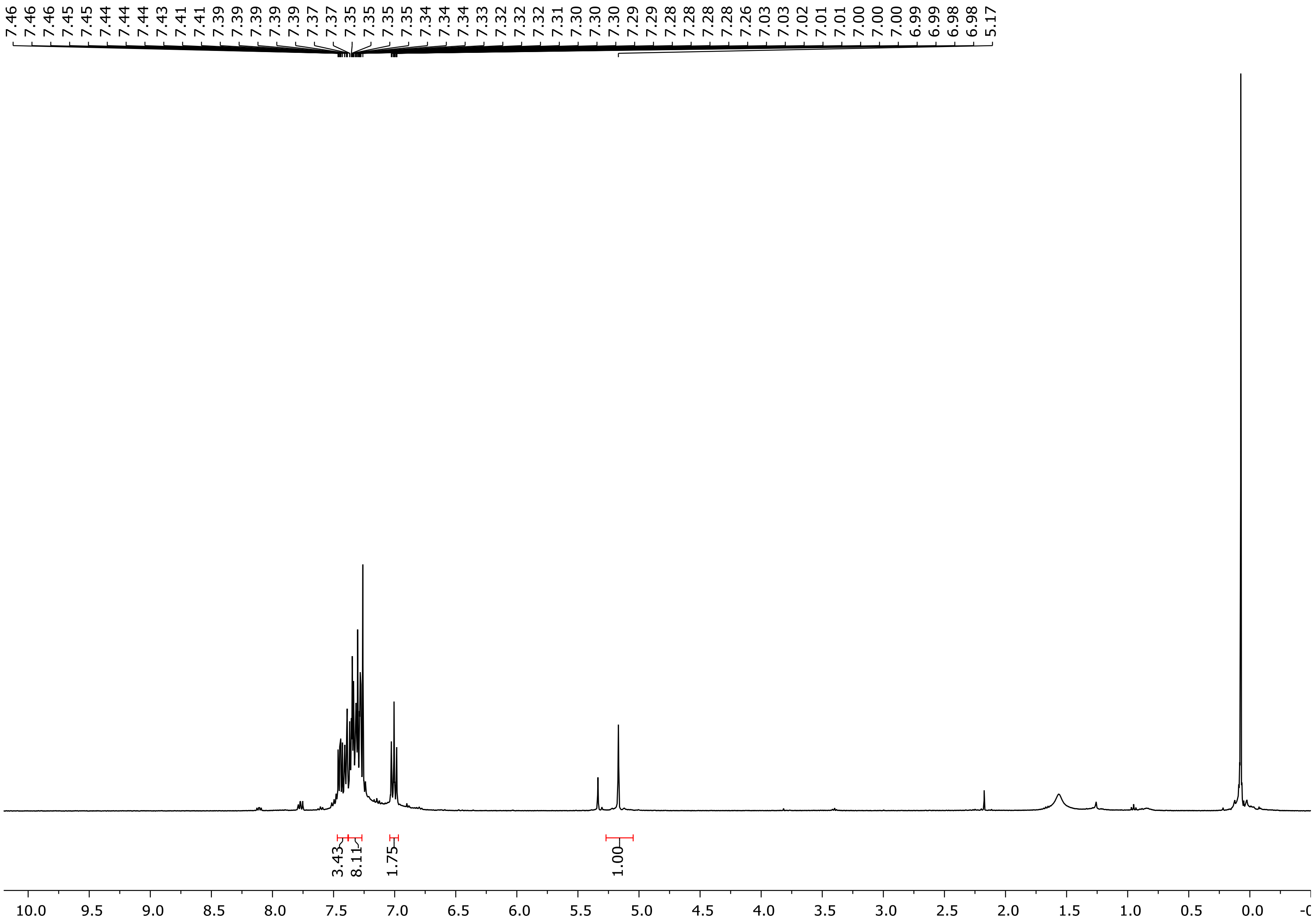

Figure S90: **^{13}C NMR** (101 MHz, $CDCl_3$, 298 K) spectrum of **2r**.

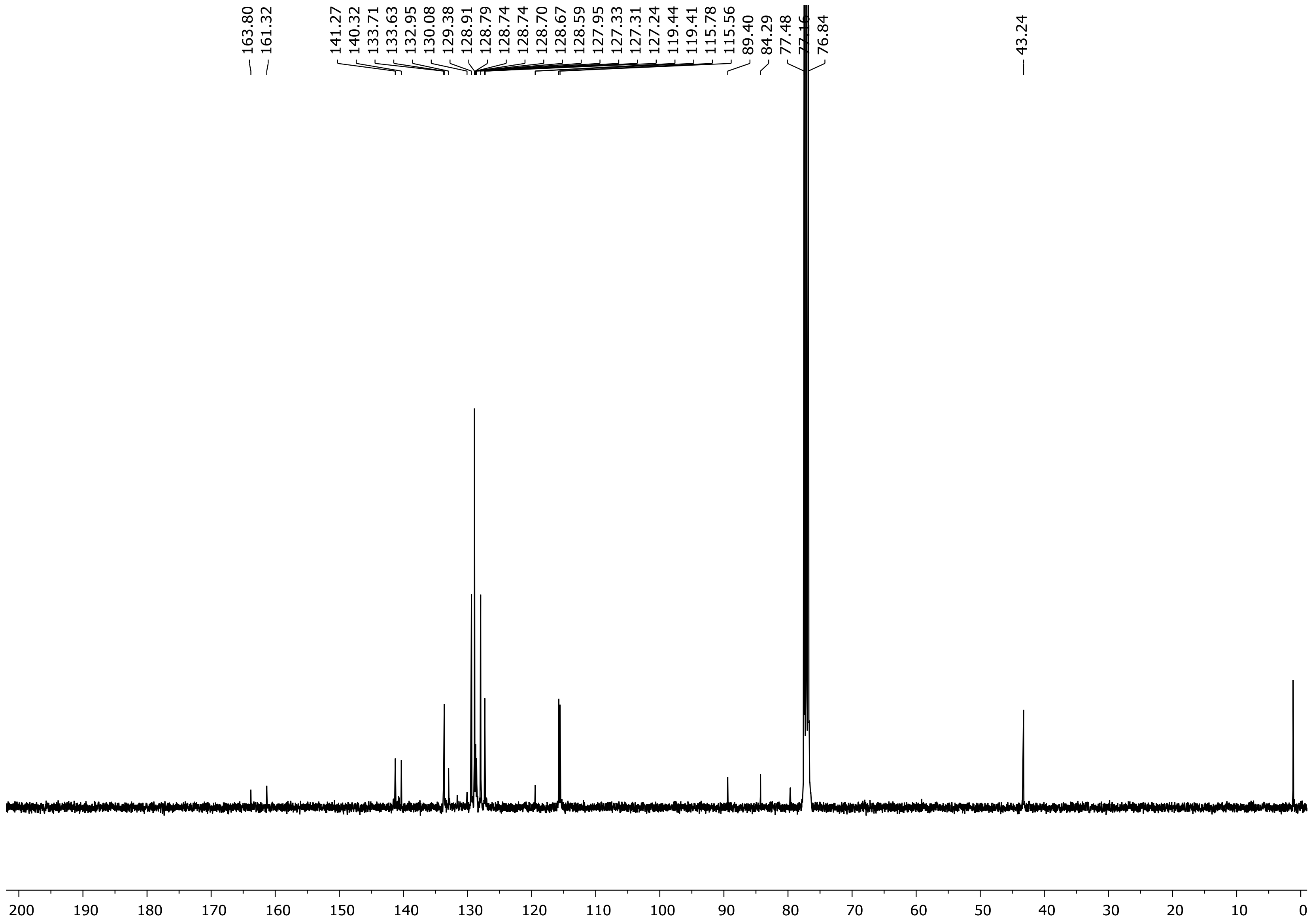

Figure S91: **^{19}F NMR** (376 MHz, $CDCl_3$, 298 K) spectrum of **2r**.

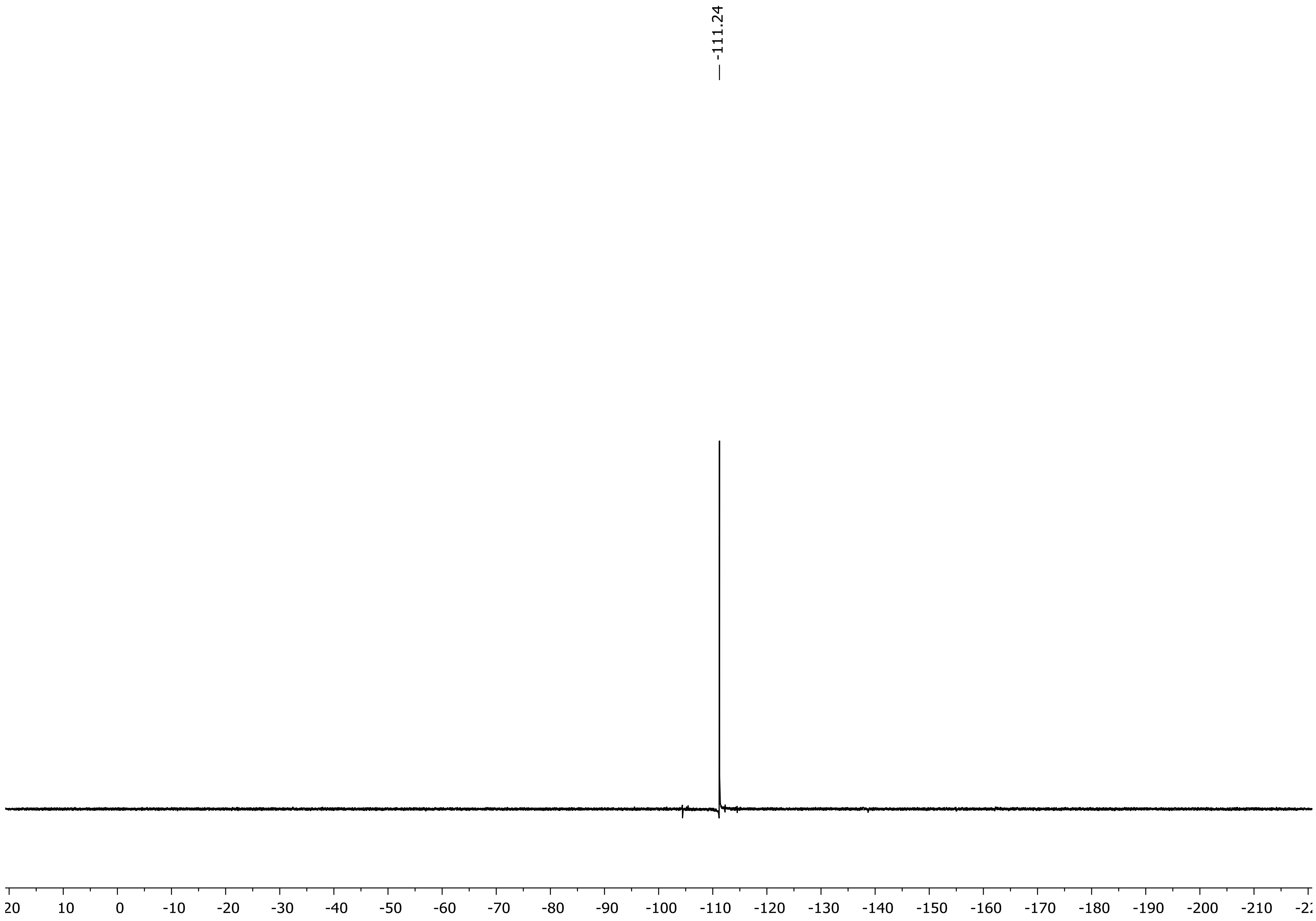

Figure S92: **^{1}H NMR** (500 MHz, $CDCl_3$, 298 K) spectrum of **2s**.

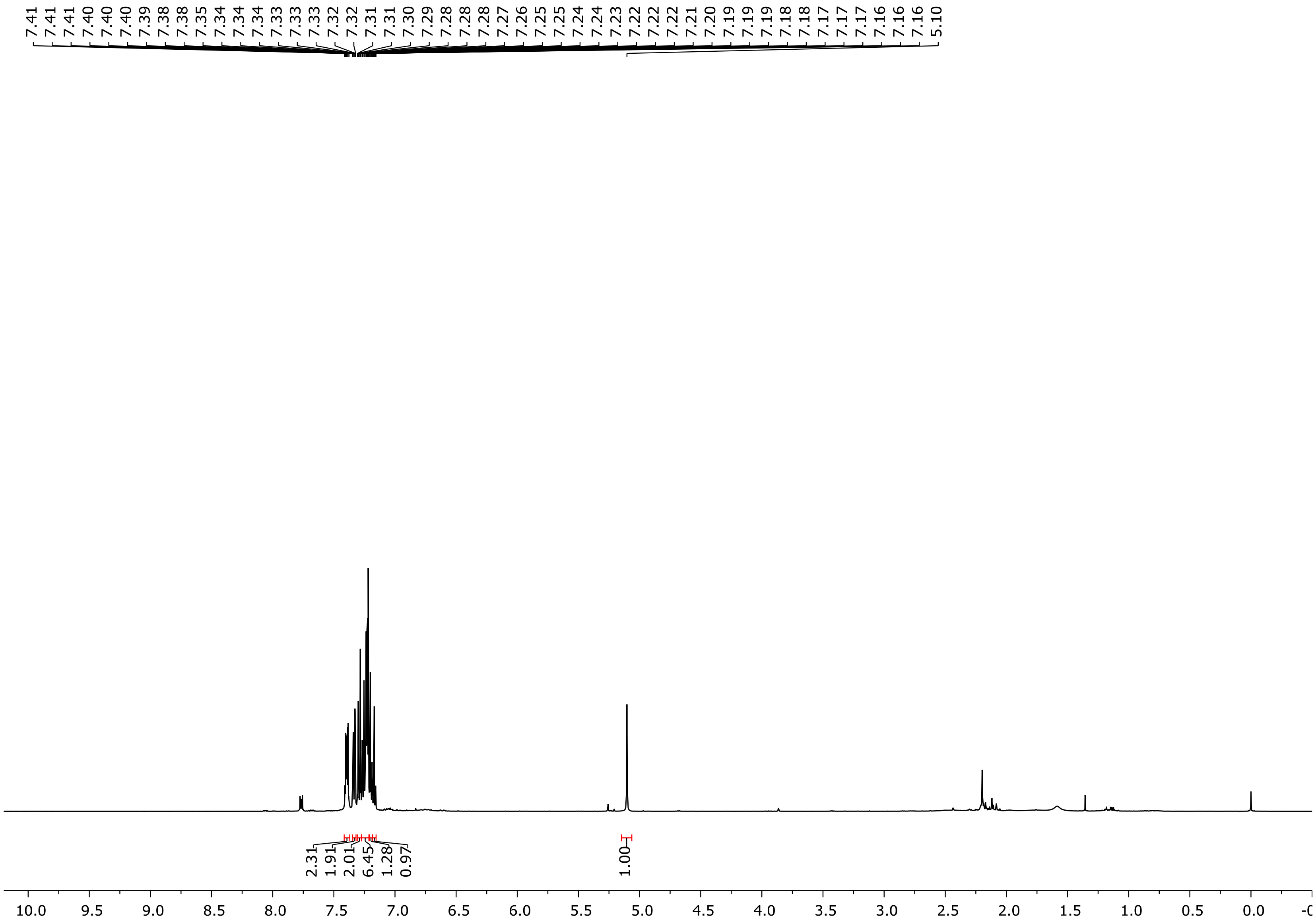

Figure S93: **^{13}C NMR** (126 MHz, $CDCl_3$, 298 K) spectrum of **2s**.

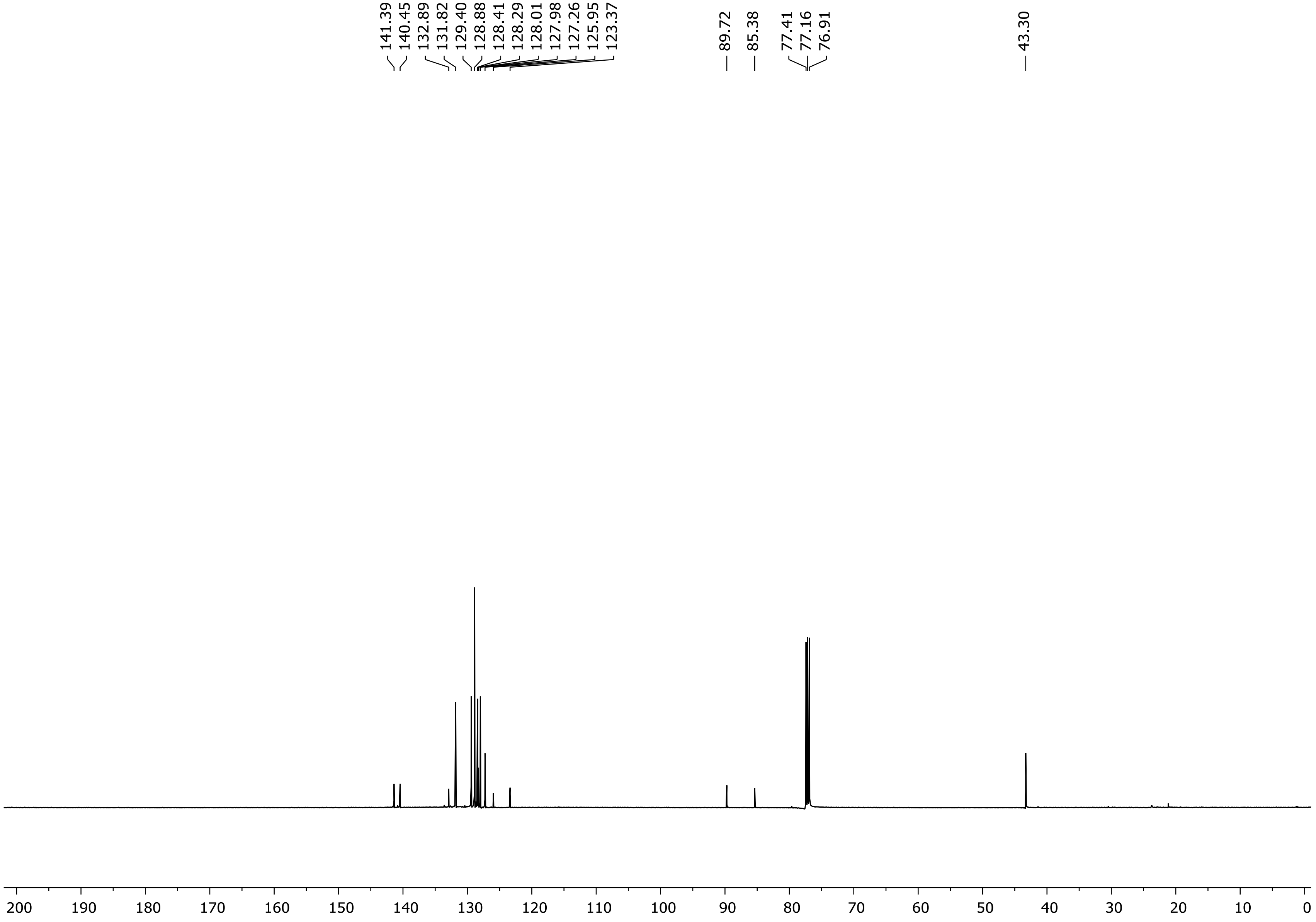

Figure S94: **^{1}H NMR** (400 MHz, $CDCl_3$, 298 K) spectrum of **2t**.

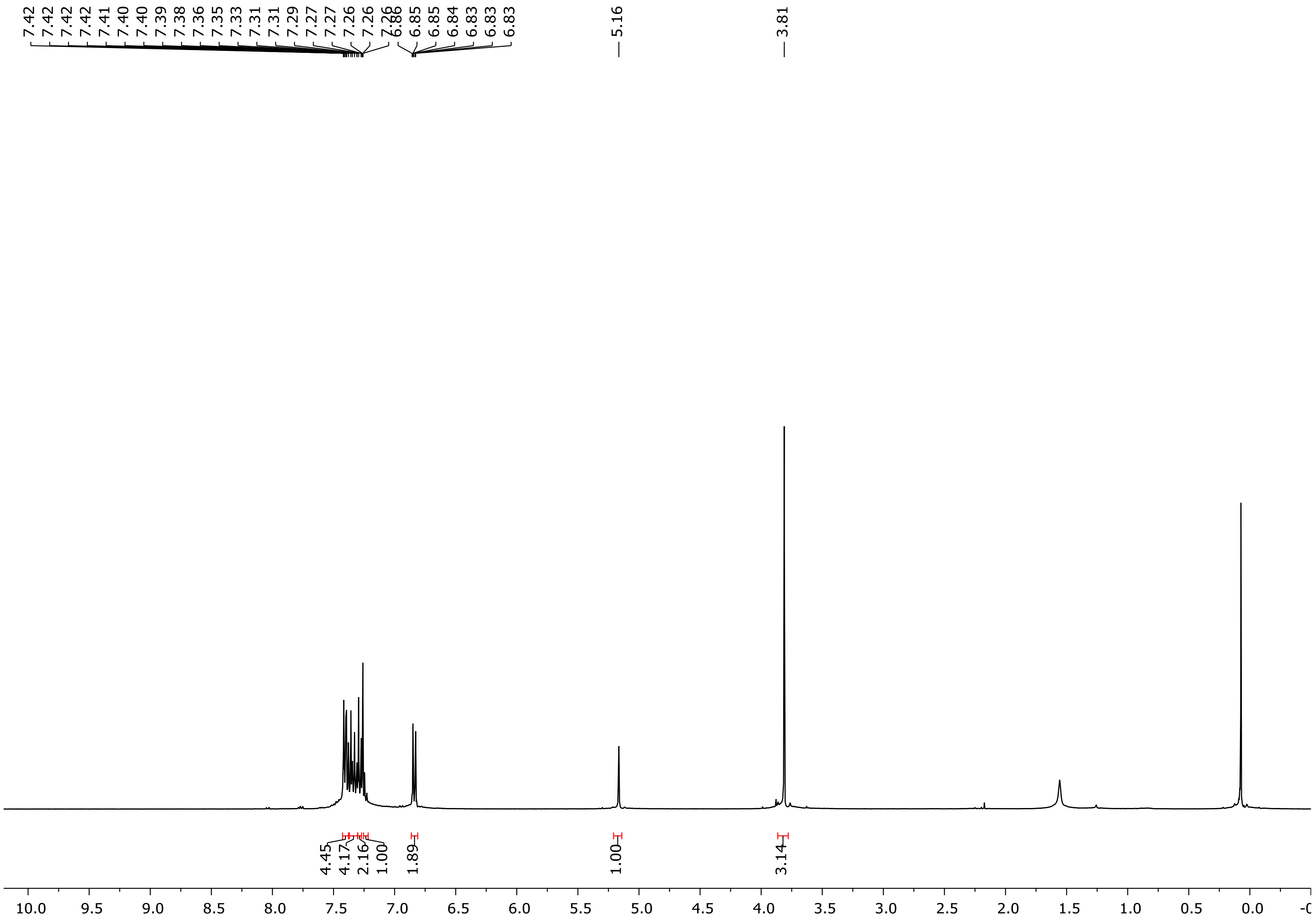

Figure S95: **^{13}C NMR** (101 MHz, $CDCl_3$, 298 K) spectrum of **2t**.

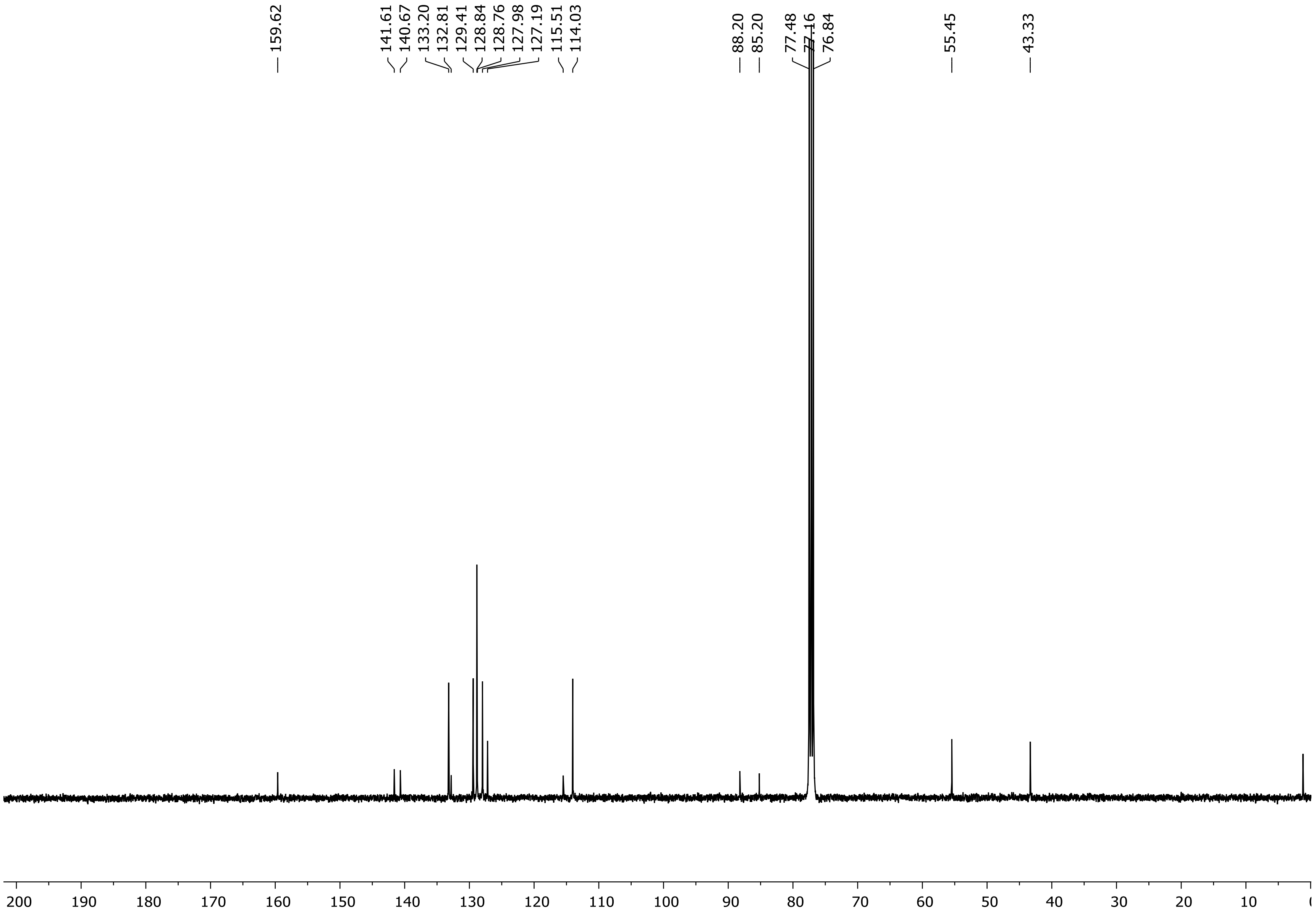

Figure S96: **^{1}H NMR** (400 MHz, $CDCl_3$, 298 K) spectrum of **2u**.

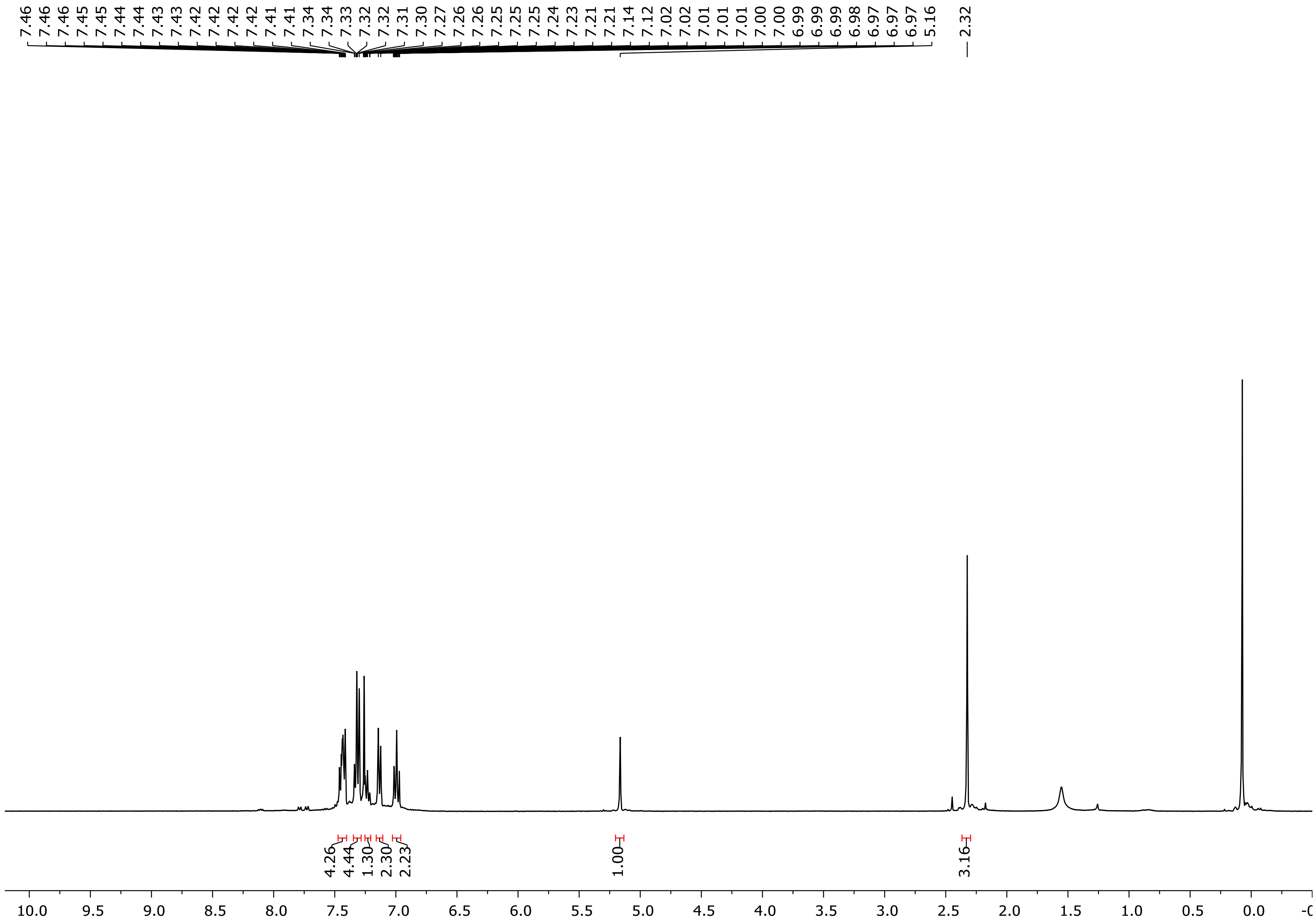

Figure S97: ^{13}**C NMR** (101 MHz, $CDCl_3$, 298 K) spectrum of **2u**.

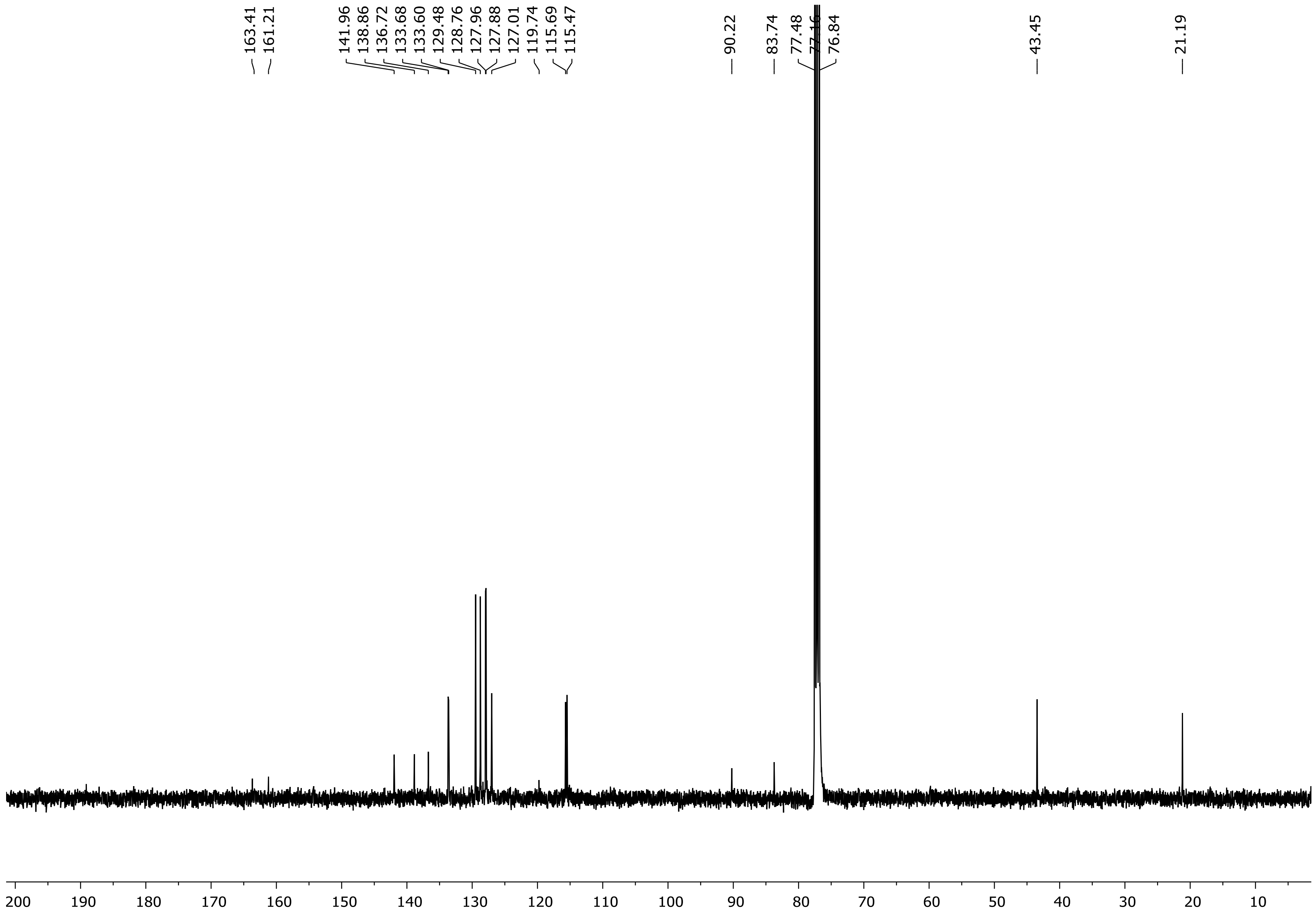

Figure S98: **^{19}F NMR** (376 MHz, $CDCl_3$, 298 K) spectrum of **2u**.

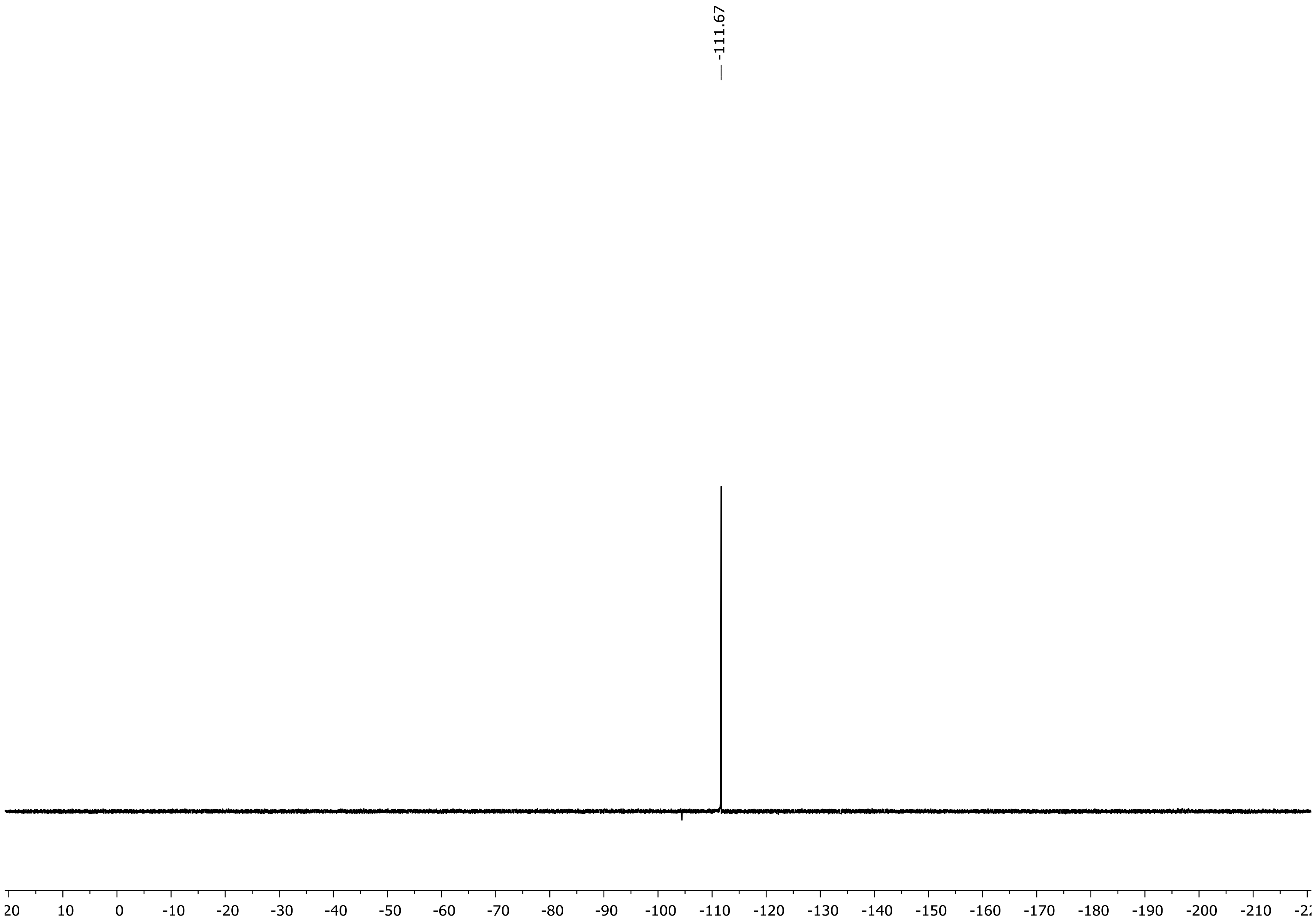

Figure S99: **^{1}H NMR** (500 MHz, $CDCl_3$, 298 K) spectrum of **2v**.

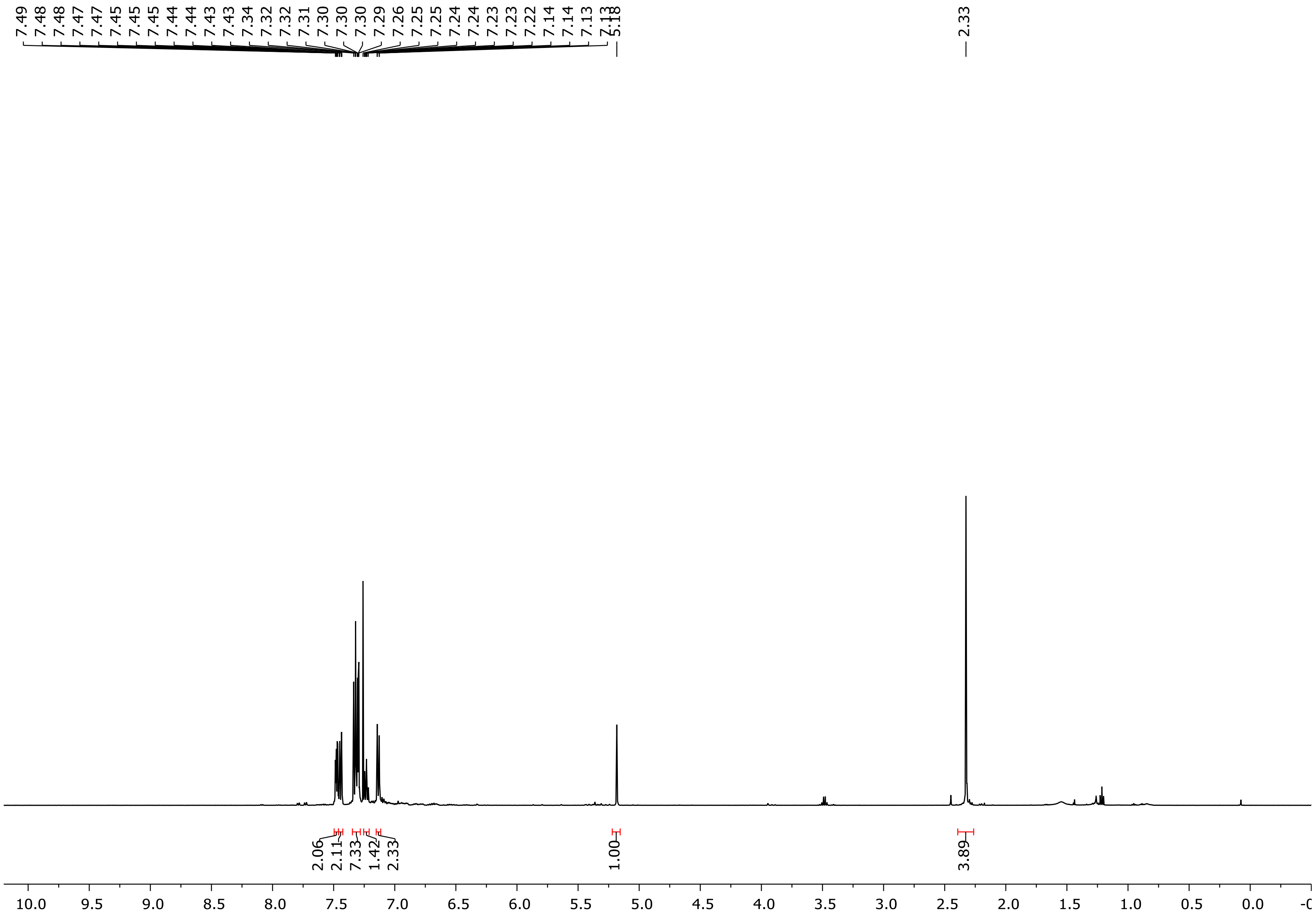

Figure S100: **^{13}C NMR** (126 MHz, $CDCl_3$, 298 K) spectrum of **2v**.

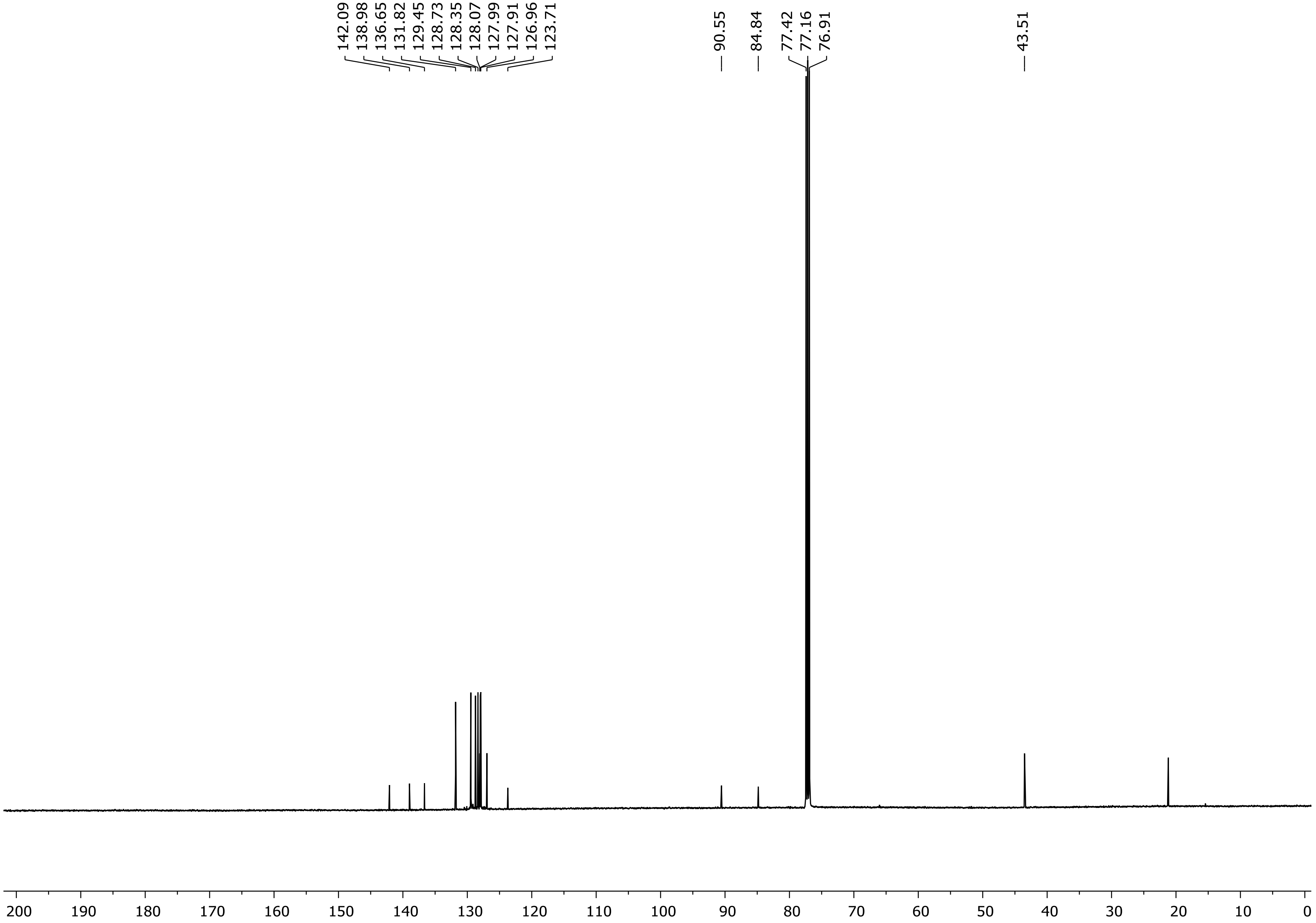

Figure S101: **^{1}H NMR** (400 MHz, $CDCl_3$, 298 K) spectrum of **2w**.

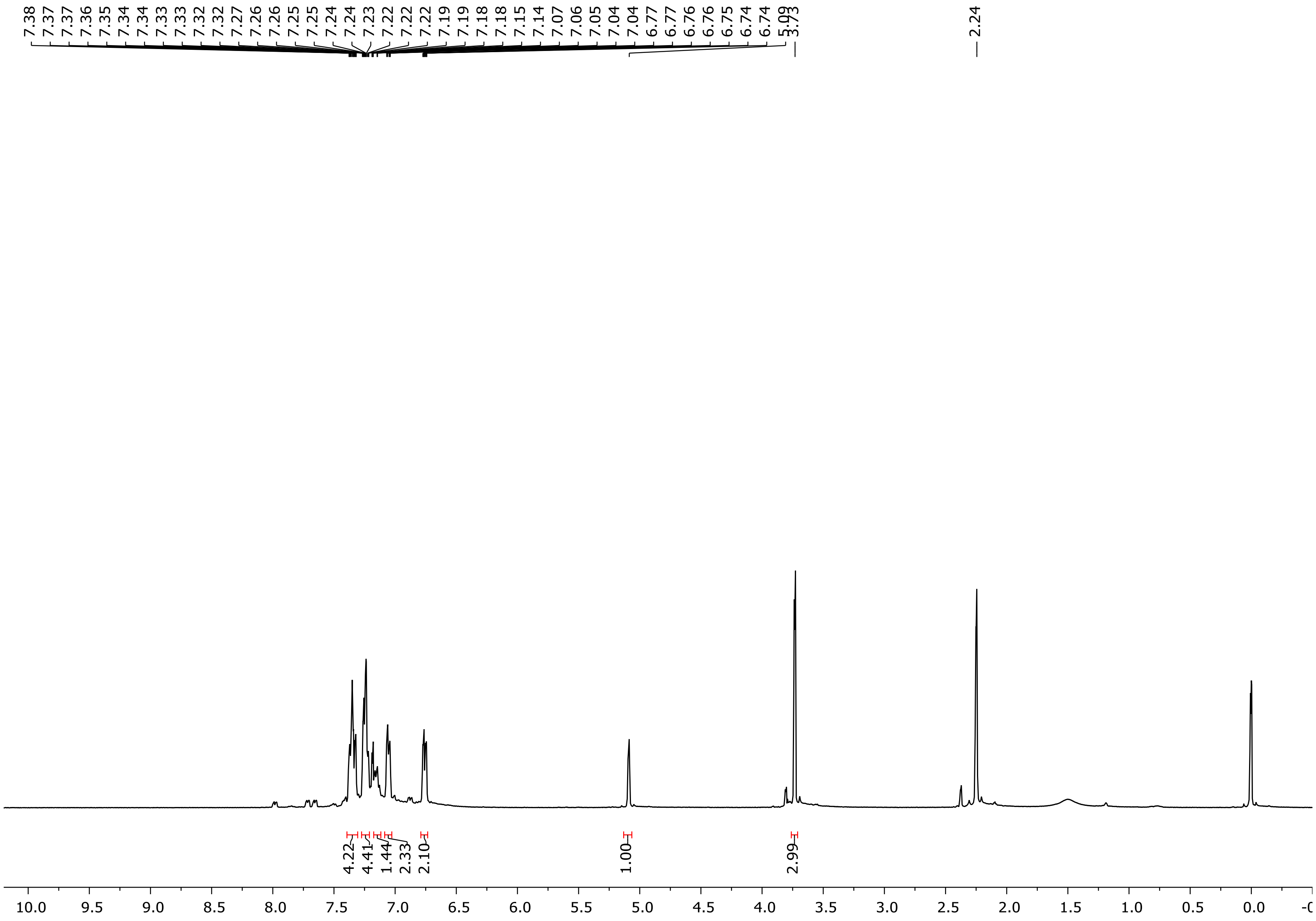

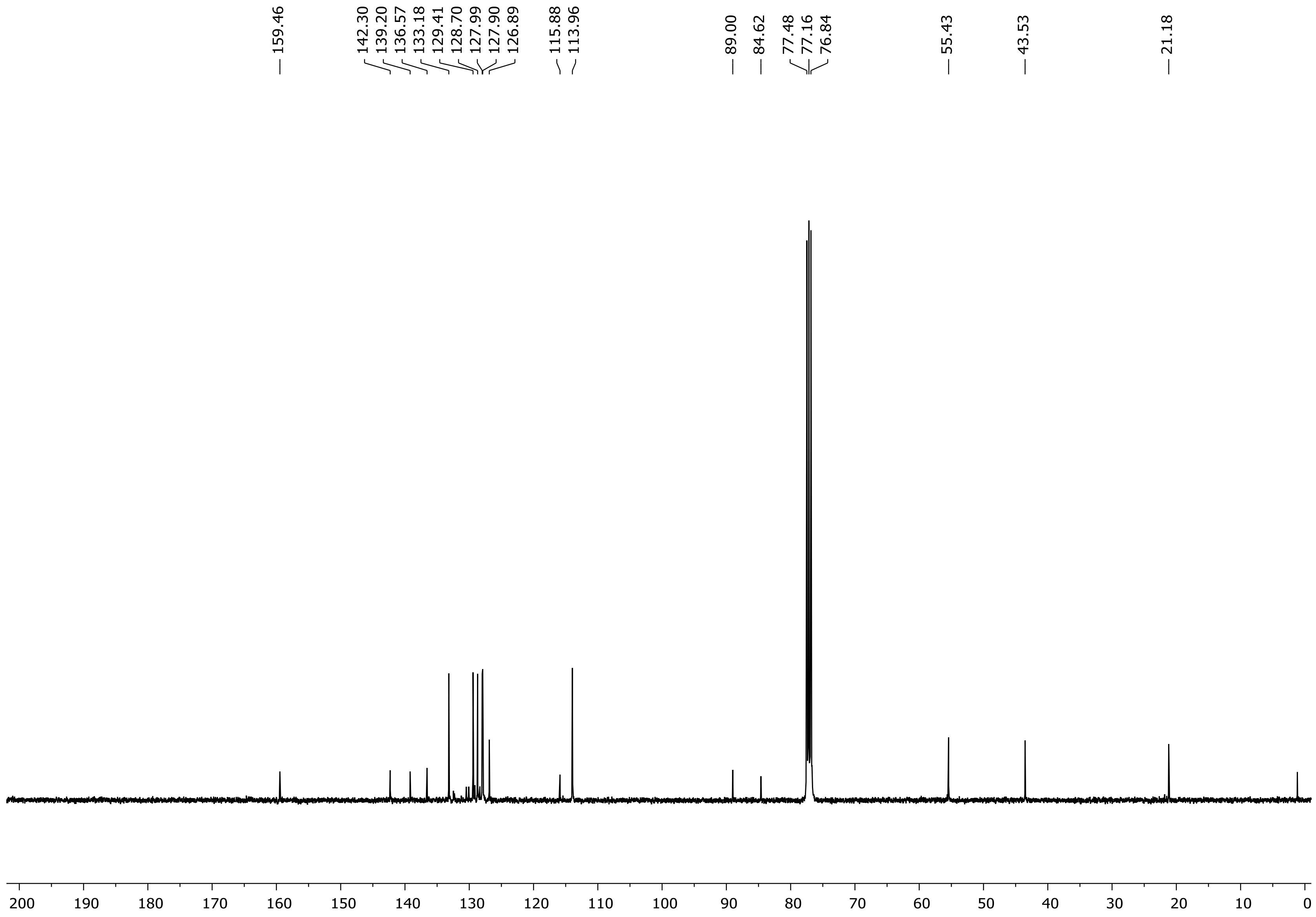

Figure S102: ^{13}**C NMR** (101 MHz, $CDCl_3$, 298 K) spectrum of **2w**.

Figure S103: **^{1}H NMR** (500 MHz, $CDCl_3$, 298 K) spectrum of **2x**.

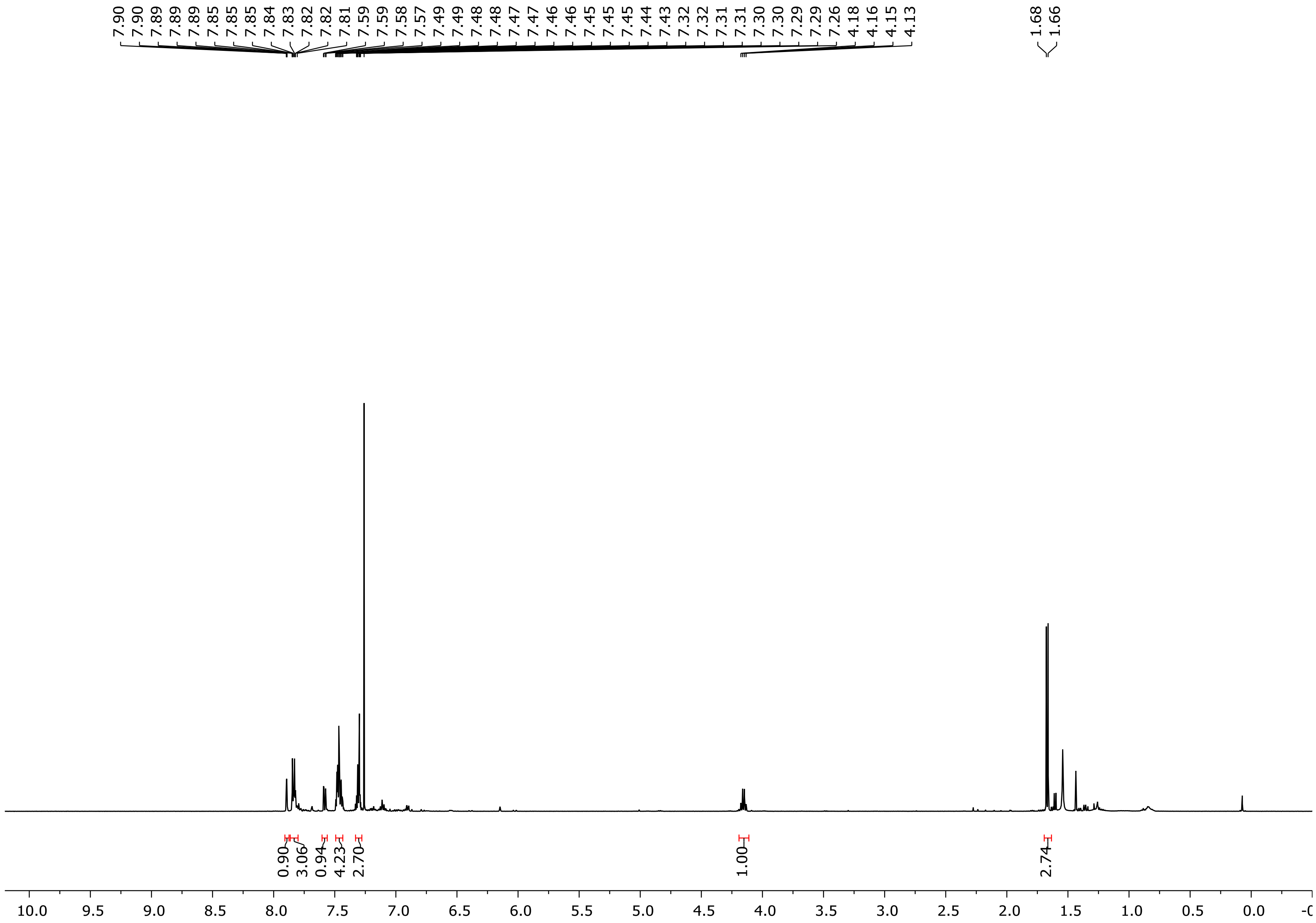

Figure S104: **^{13}C NMR** (126 MHz, $CDCl_3$, 298 K) spectrum of **2x**.

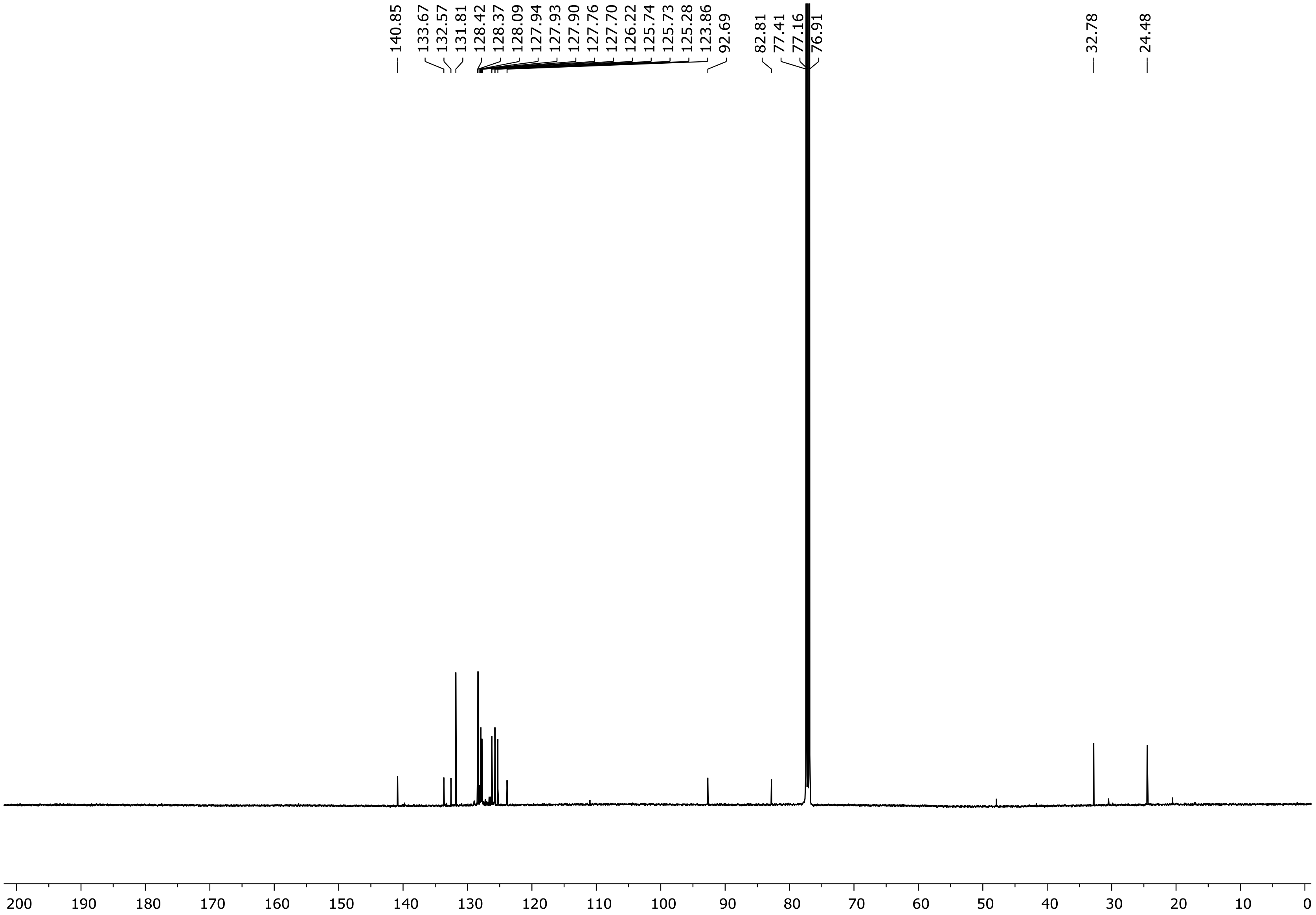

Figure S105: **^{1}H NMR** (400 MHz, $CDCl_3$, 298 K) spectrum of **2y**.

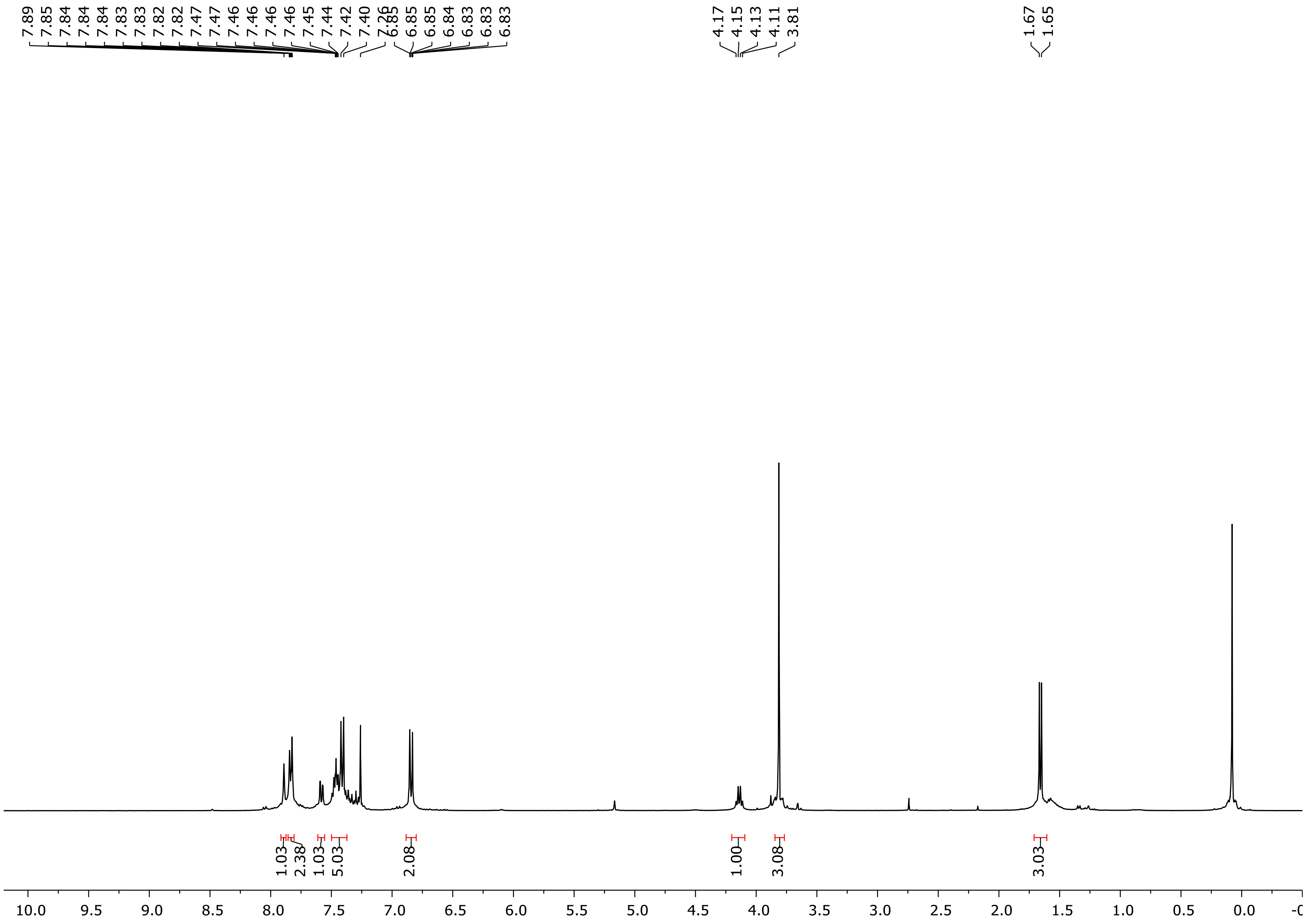

Figure S106: **^{13}C NMR** (101 MHz, $CDCl_3$, 298 K) spectrum of **2y**.

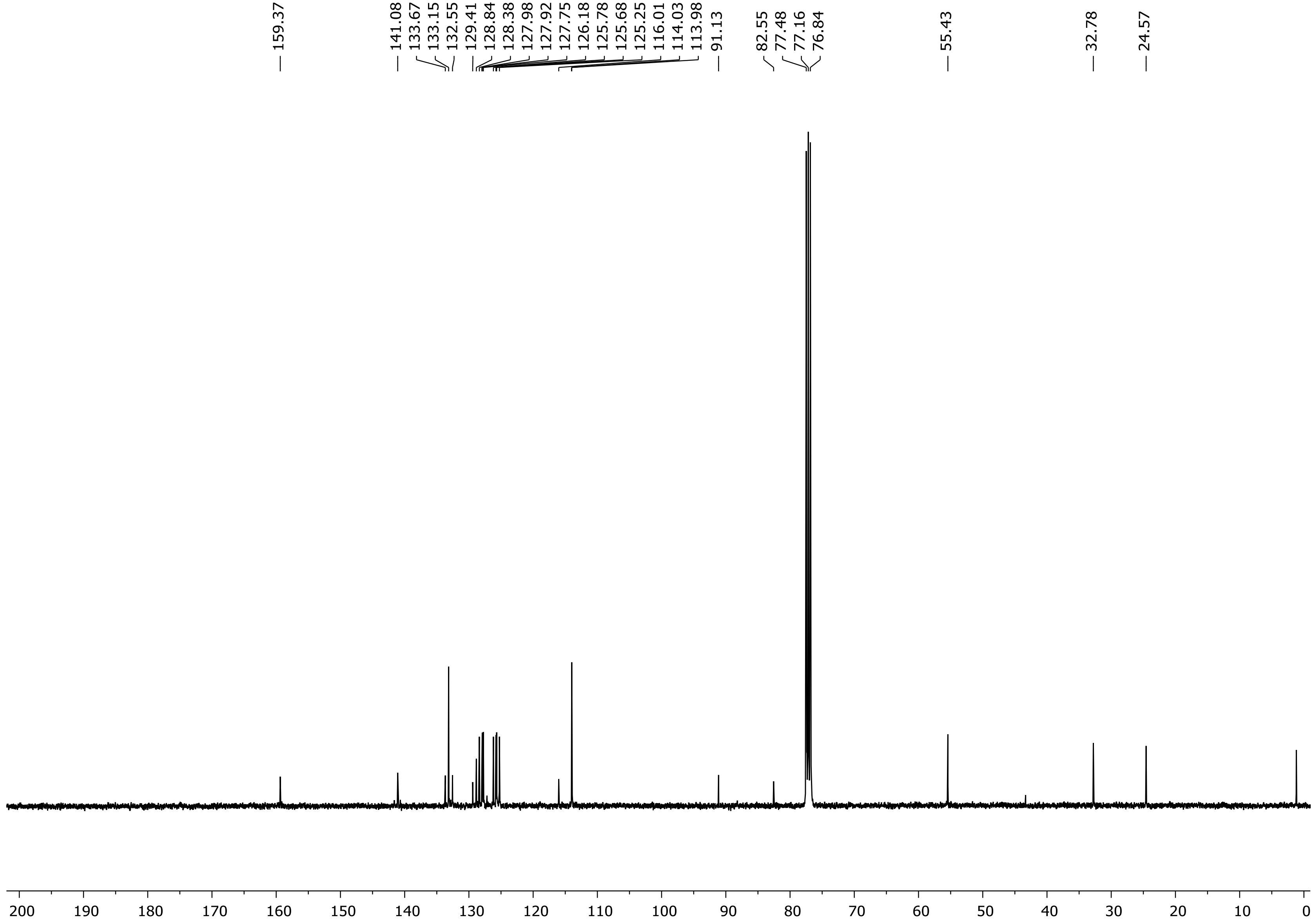

Figure S107: **^{1}H NMR** (500 MHz, $CDCl_3$, 298 K) spectrum of **2z**.

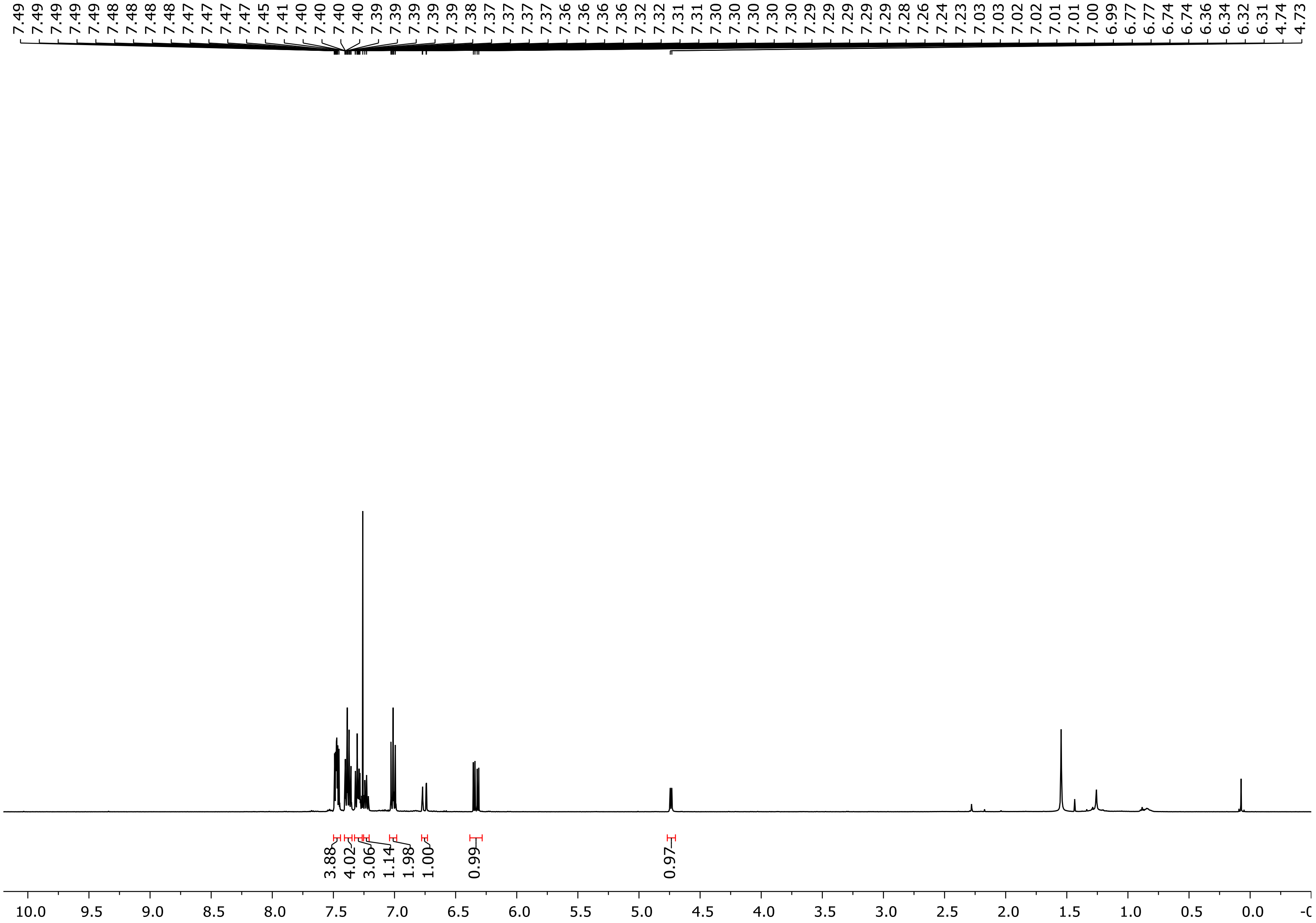

Figure S108: **^{13}C NMR** (126 MHz, $CDCl_3$, 298 K) spectrum of **2z**.

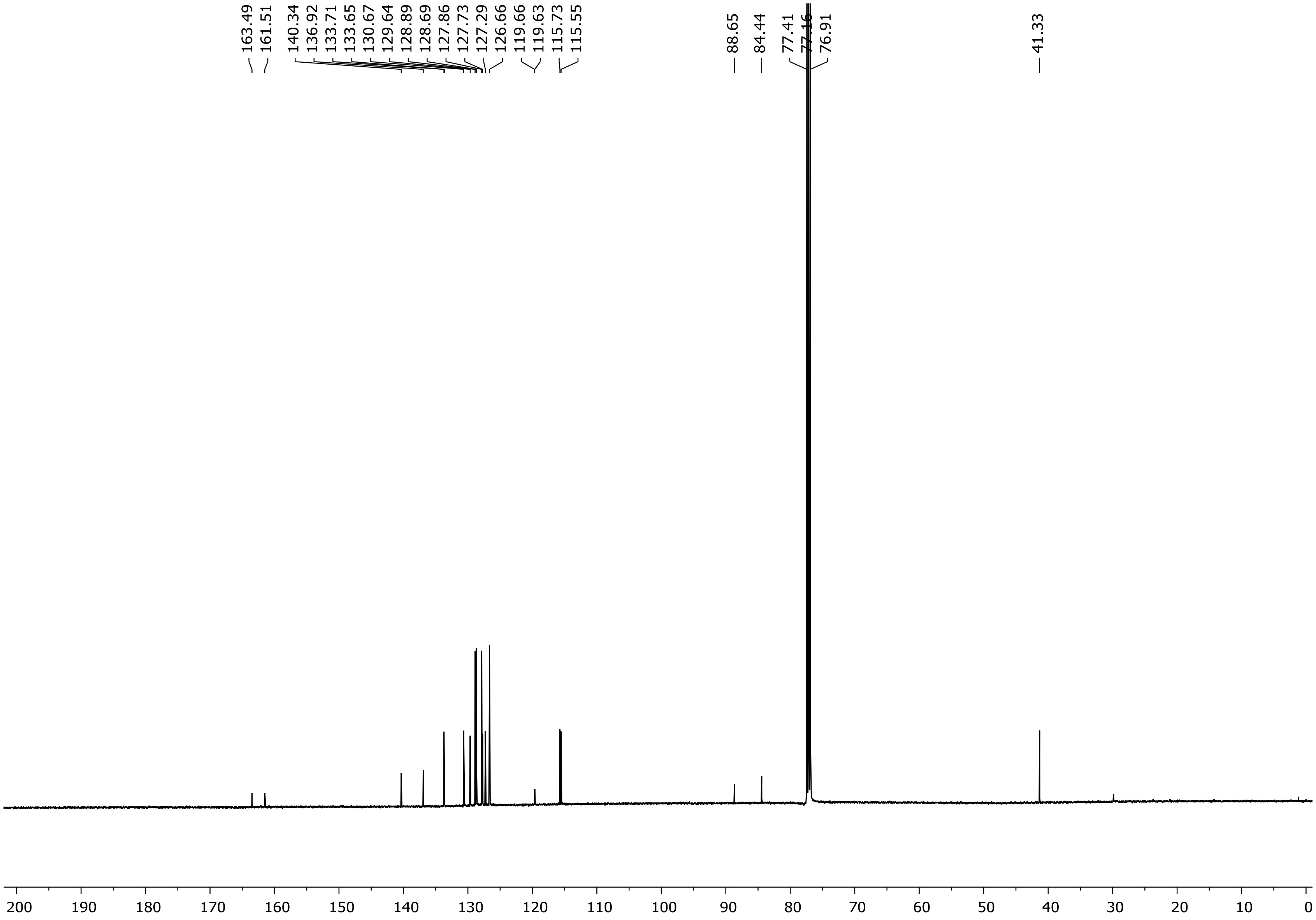

Figure S109: **^{19}F NMR** (471 MHz, $CDCl_3$, 298 K) spectrum of **2z**.

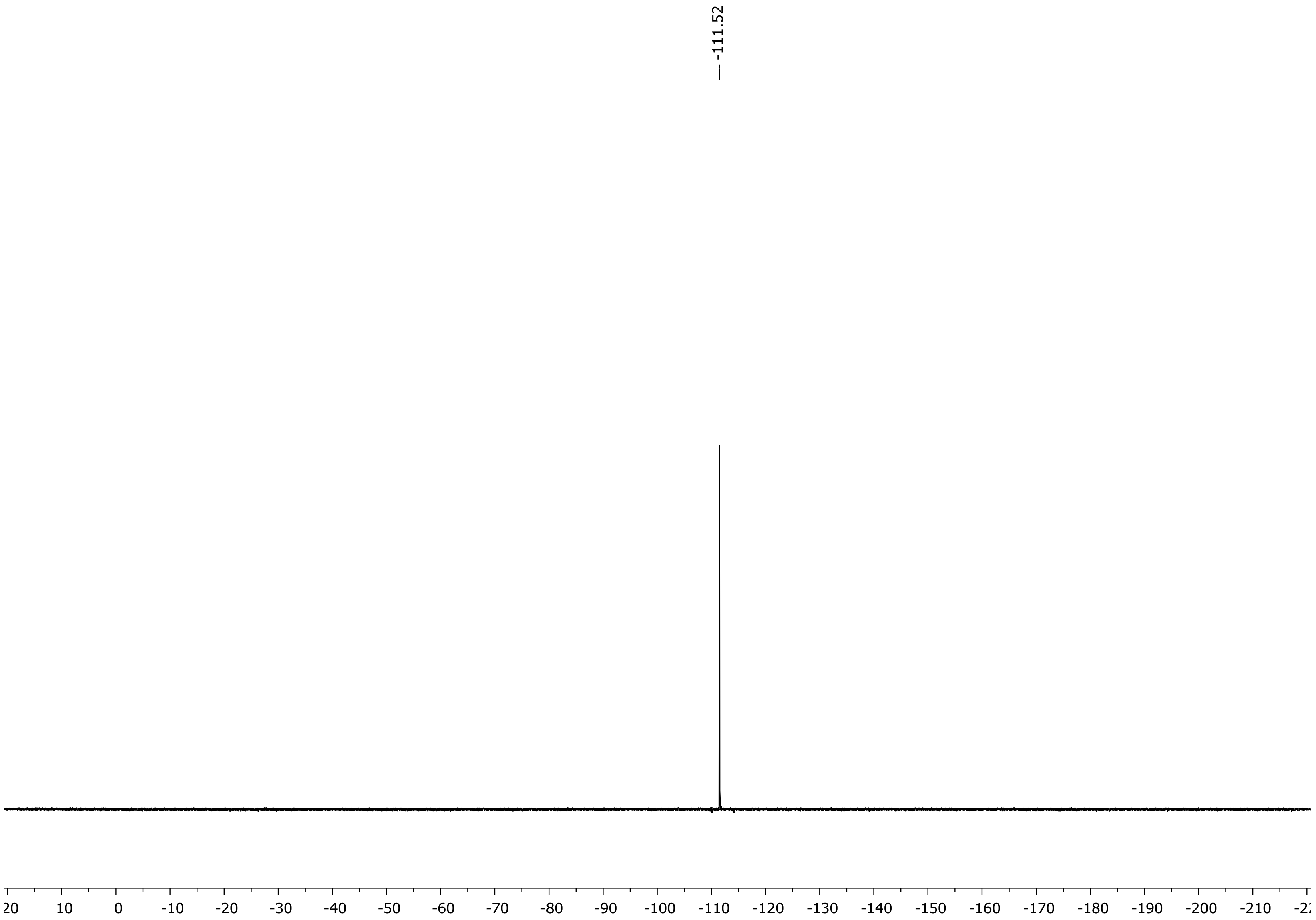

Figure S110: **^{1}H NMR** (500 MHz, $CDCl_3$, 298 K) spectrum of **2aa**.

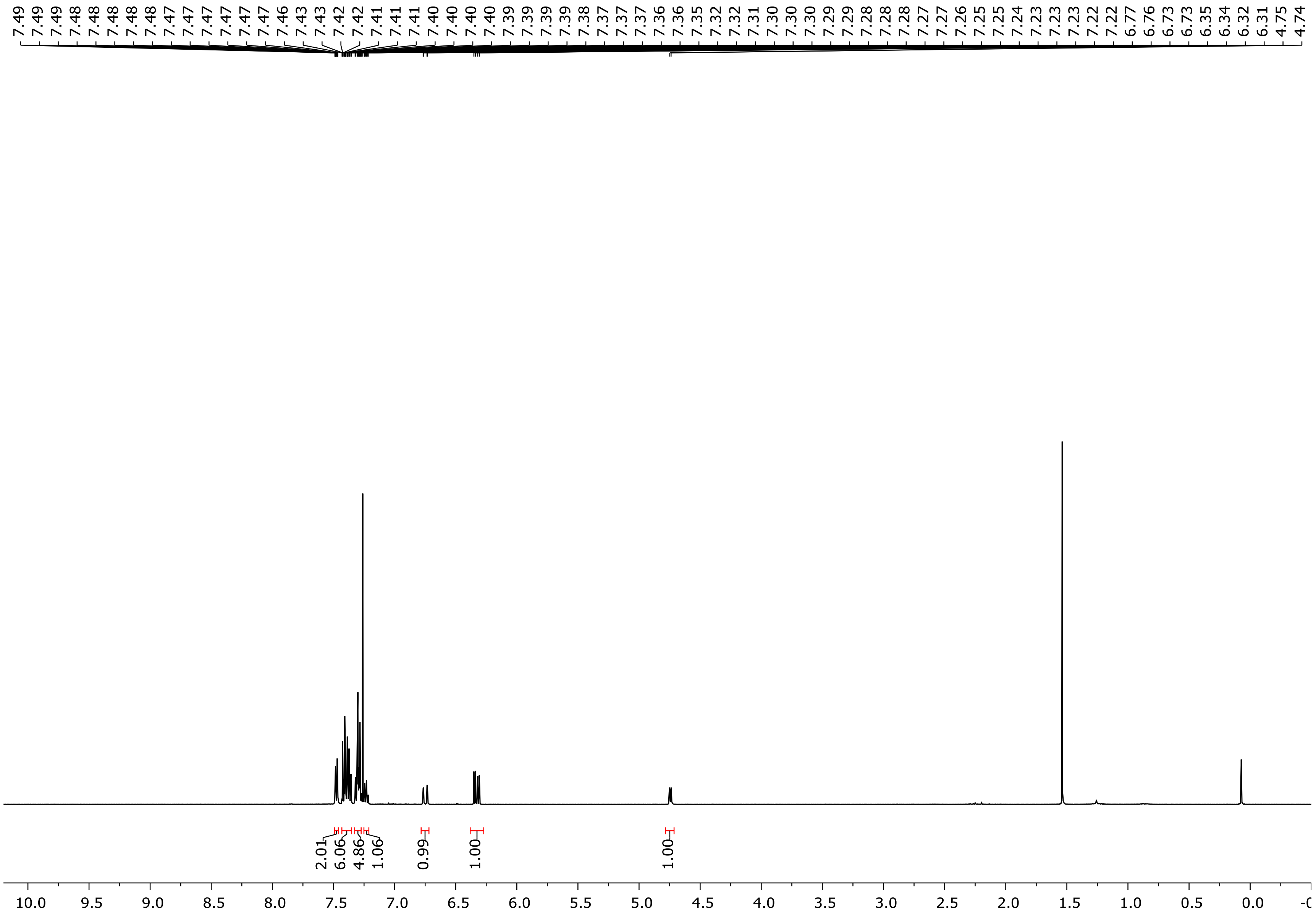

Figure S111: **^{13}C NMR** (126 MHz, $CDCl_3$, 298 K) spectrum of **2aa**.

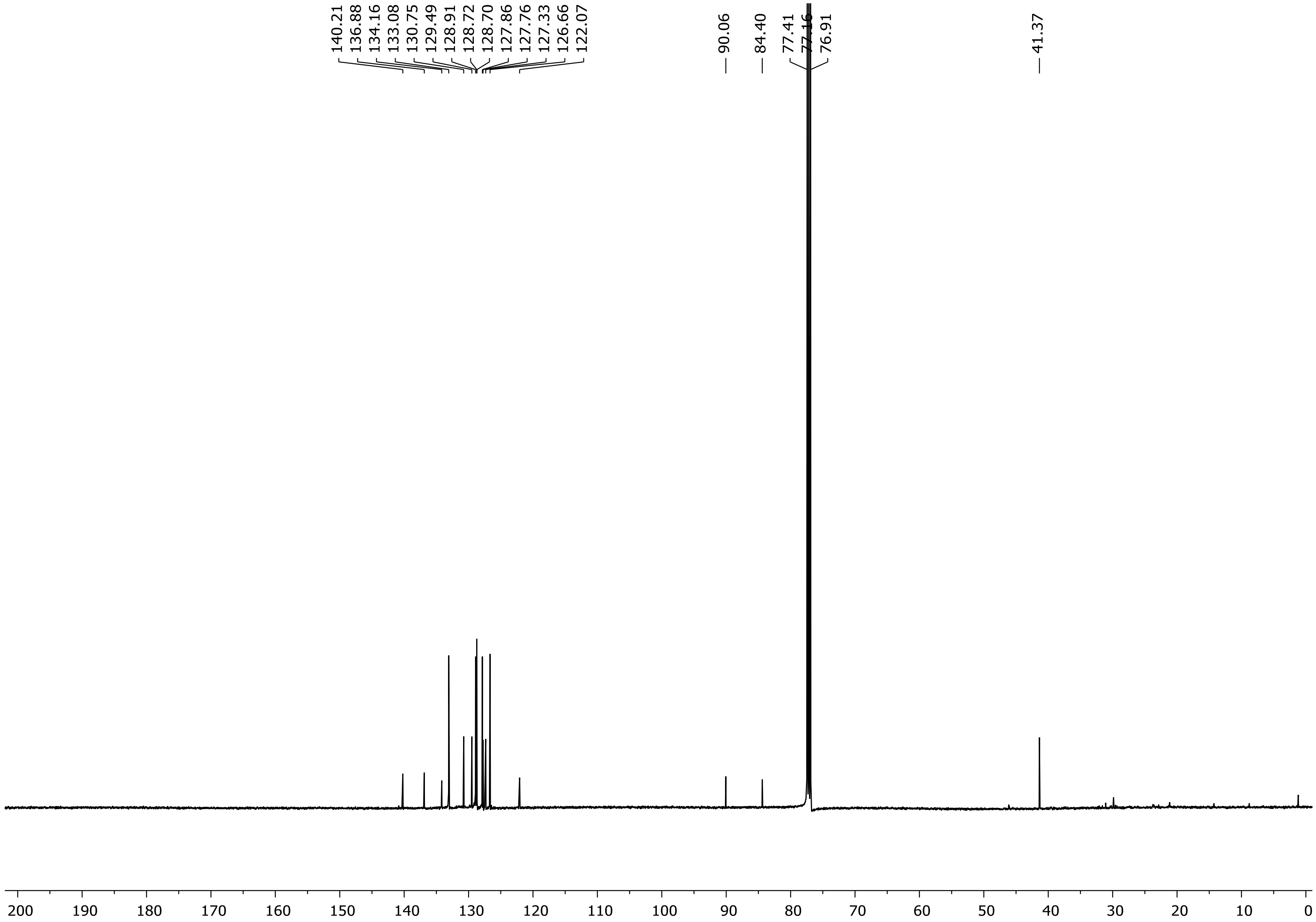

Figure S112: **^{1}H NMR** (500 MHz, $CDCl_3$, 298 K) spectrum of **2ab**.

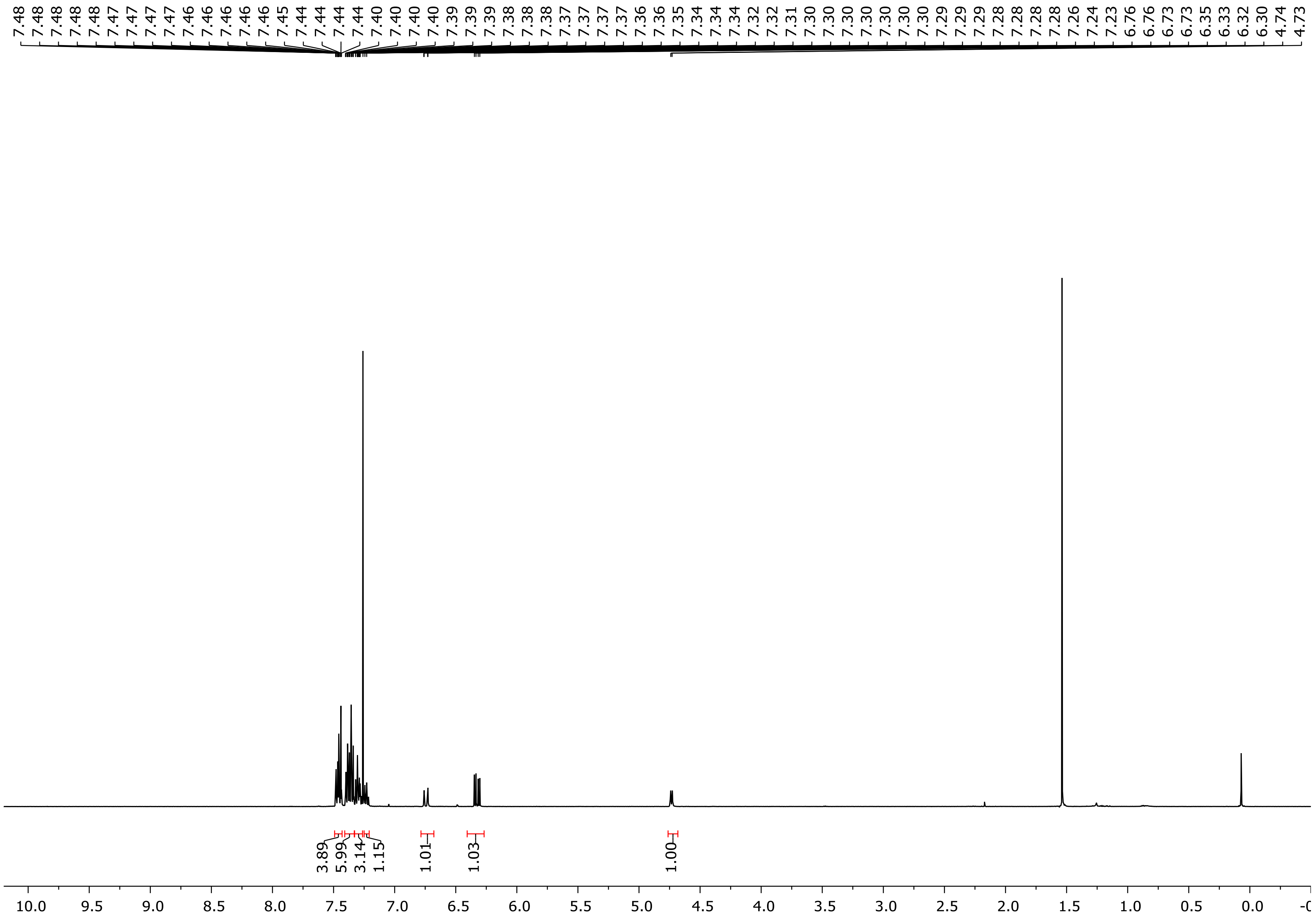

Figure S113: **^{13}C NMR** (126 MHz, $CDCl_3$, 298 K) spectrum of **2ab**.

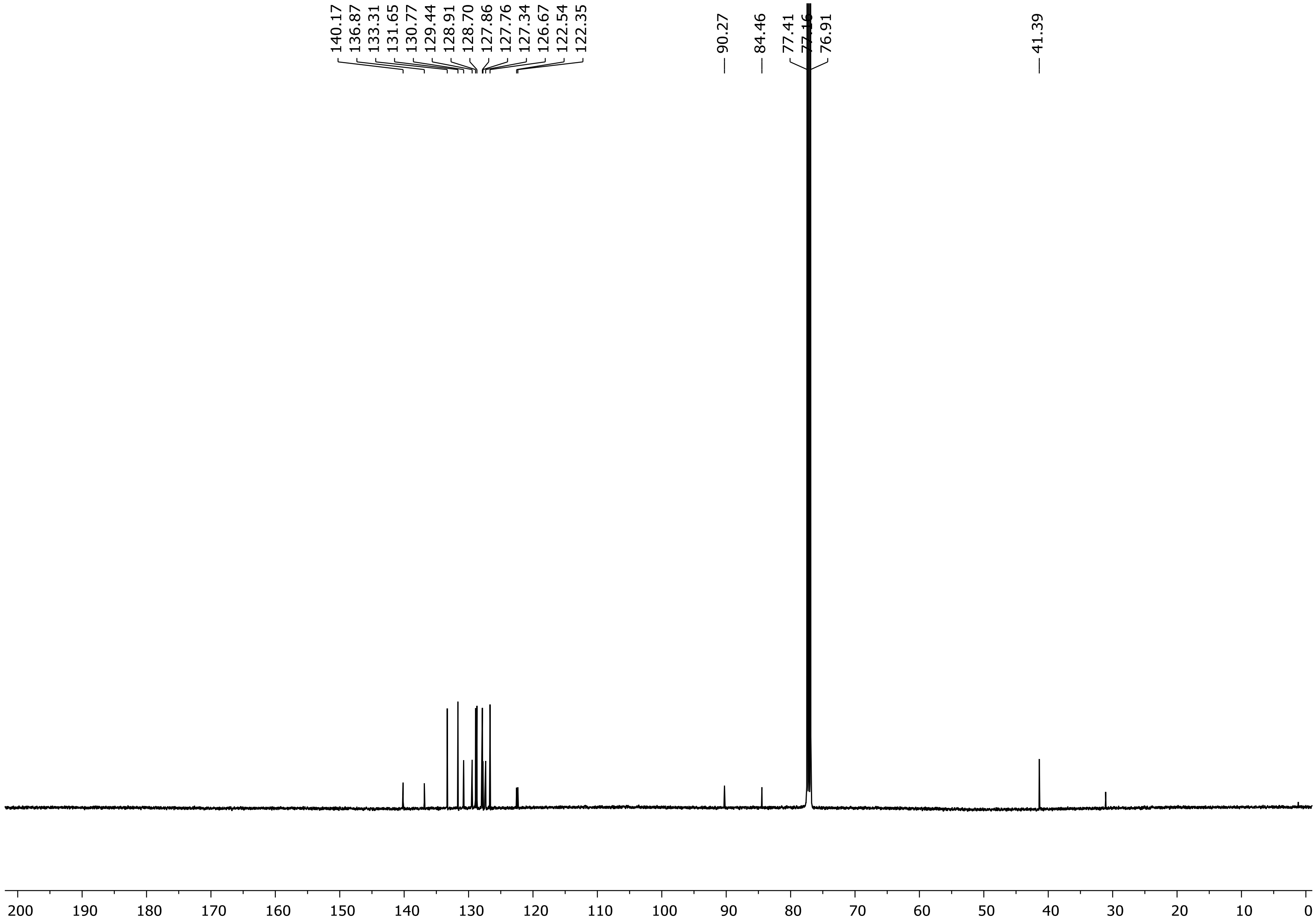

Figure S114: **^{1}H NMR** (500 MHz, $CDCl_3$, 298 K) spectrum of **2ac**.

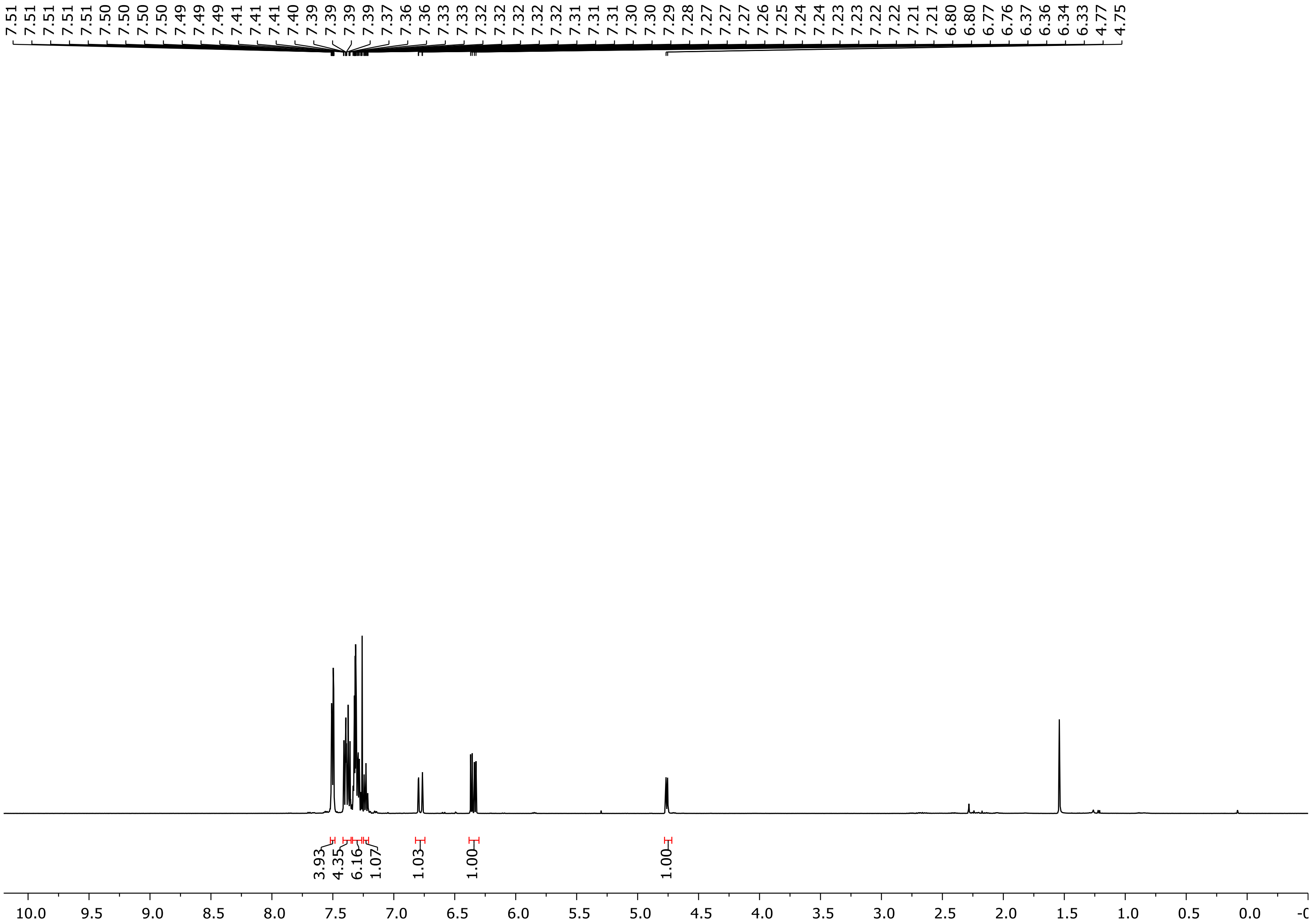

Figure S115: **^{13}C NMR** (126 MHz, $CDCl_3$, 298 K) spectrum of **2ac**.

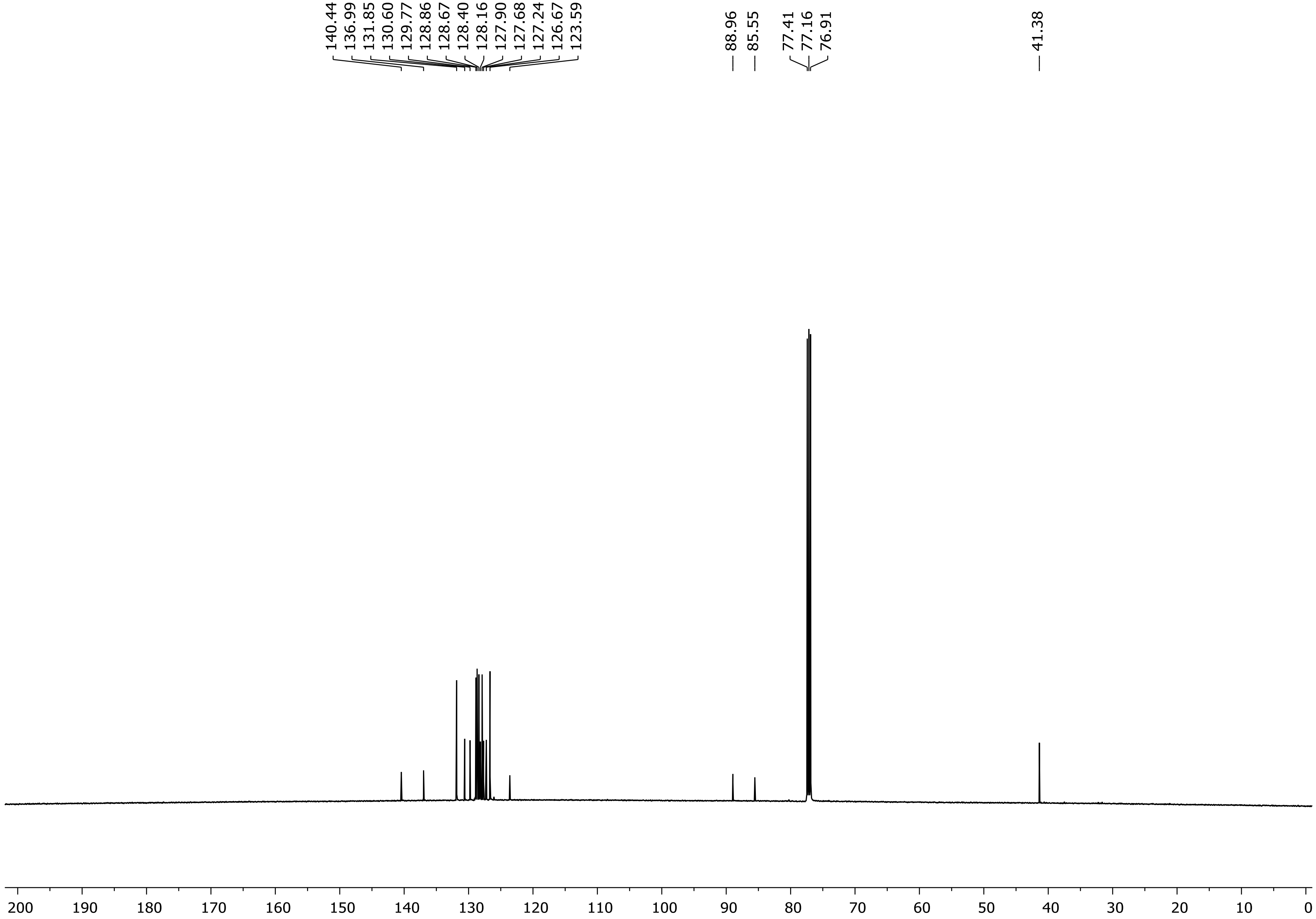

Figure S116: **^{1}H NMR** (500 MHz, $CDCl_3$, 298 K) spectrum of **2ad**.

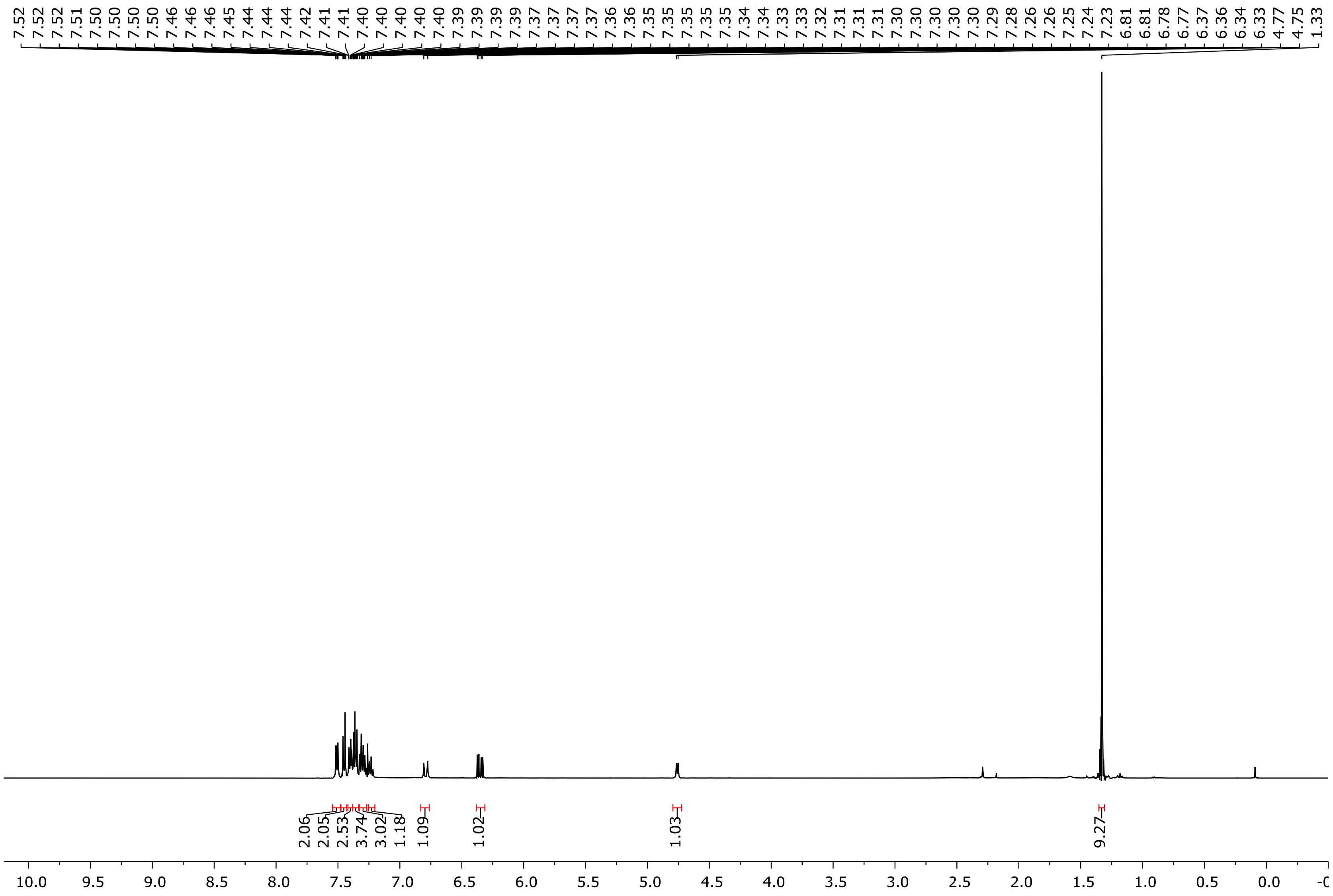

Figure S117: **^{13}C NMR** (126 MHz, $CDCl_3$, 298 K) spectrum of **2ad**.

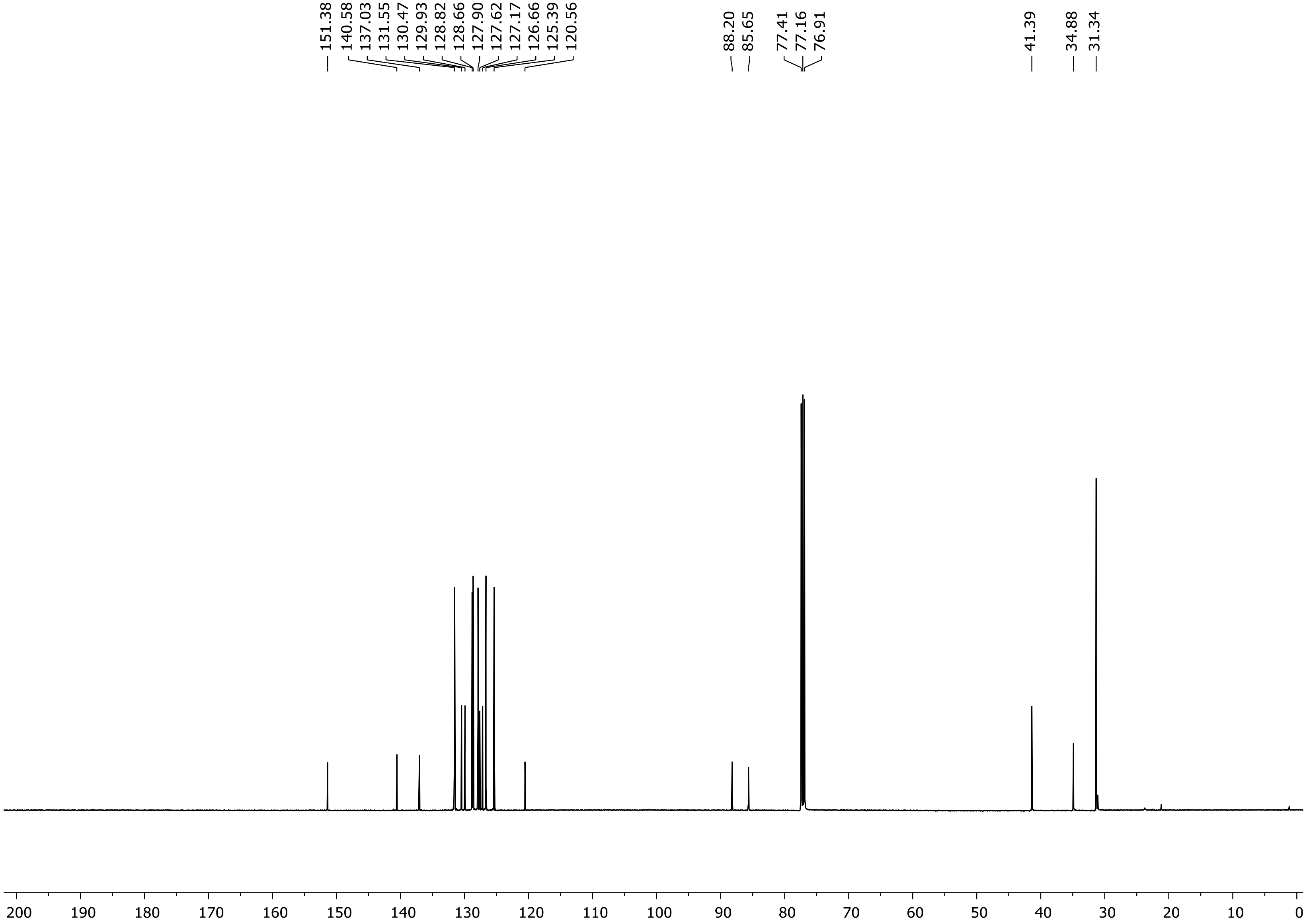

Figure S118: **^{1}H NMR** (500 MHz, $CDCl_3$, 298 K) spectrum of **2ae**.

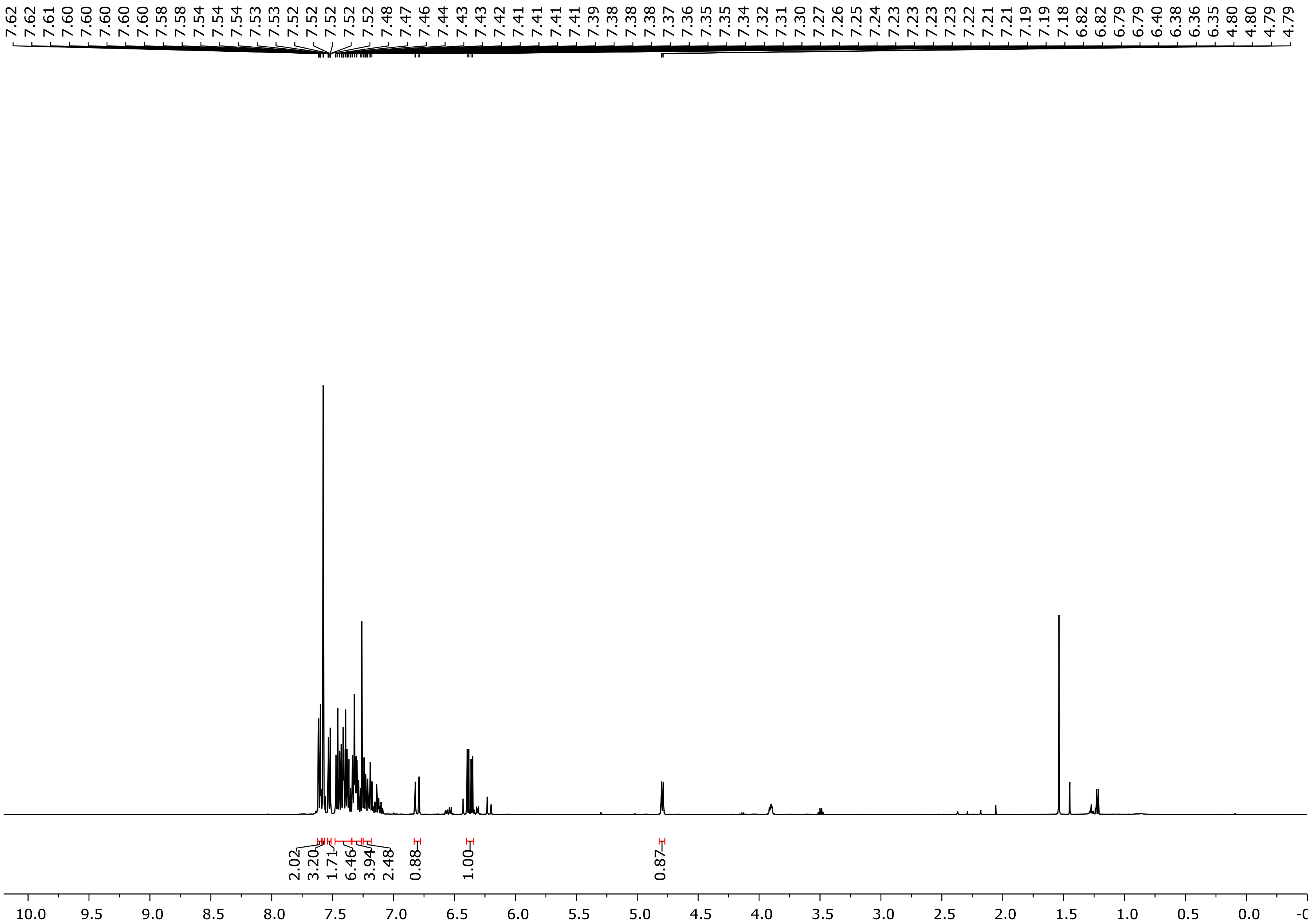

Figure S119: **^{13}C NMR** (126 MHz, $CDCl_3$, 298 K) spectrum of **2ae**.

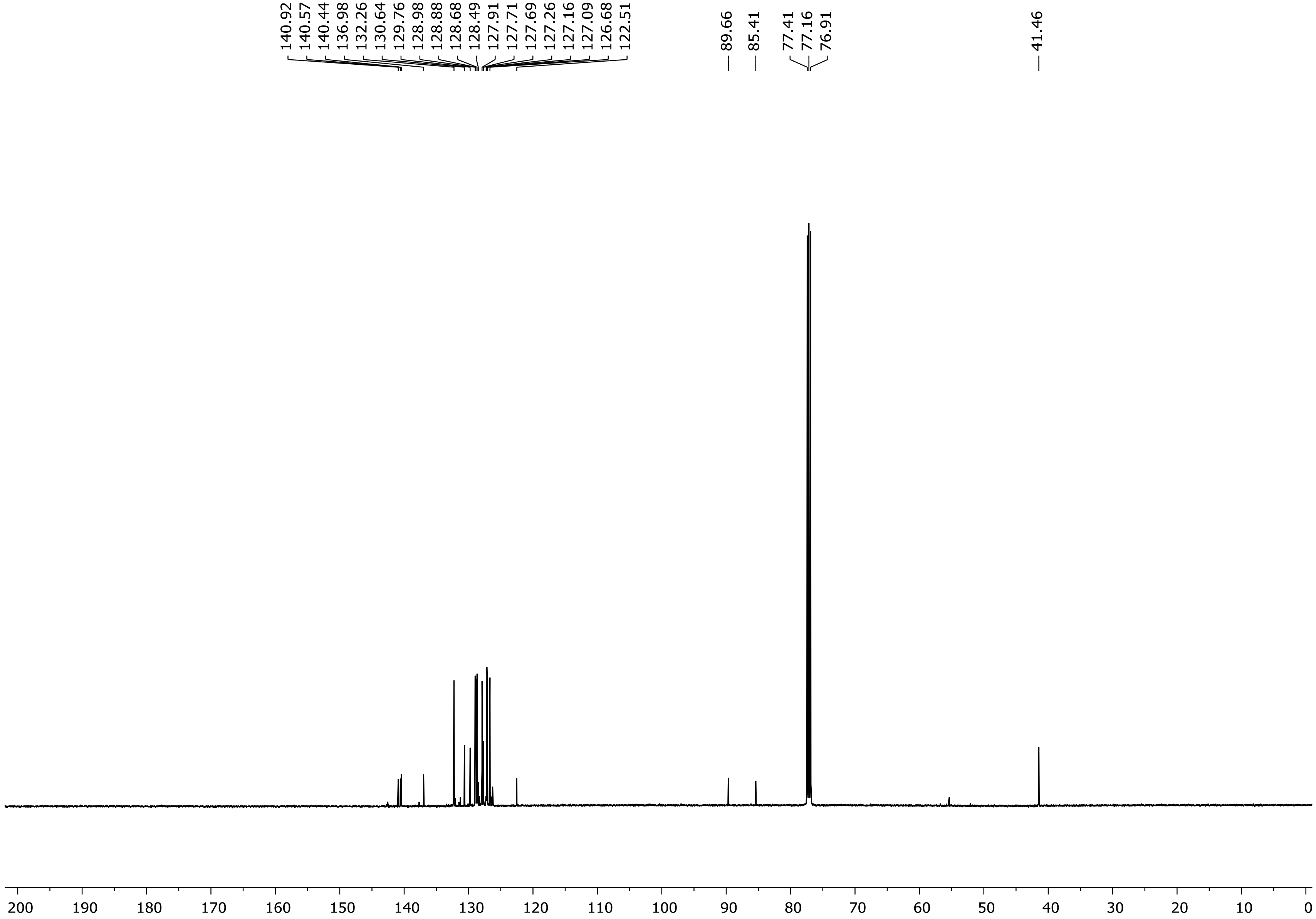

Figure S120: **^{1}H NMR** (500 MHz, $CDCl_3$, 298 K) spectrum of **2af**.

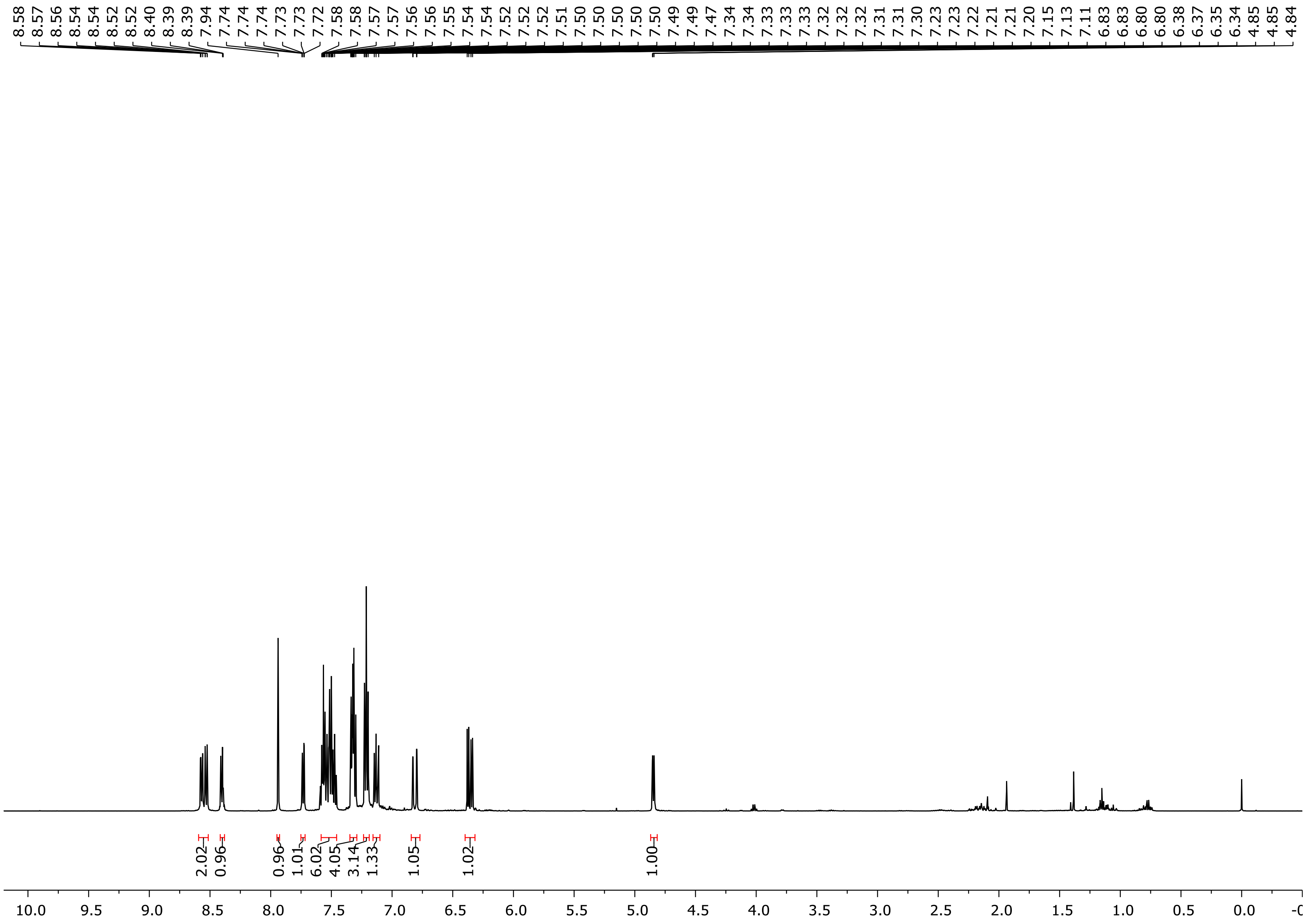

Figure S9121: ^{13}**C NMR** (126 MHz, $CDCl_3$, 298 K) spectrum of **2af**.

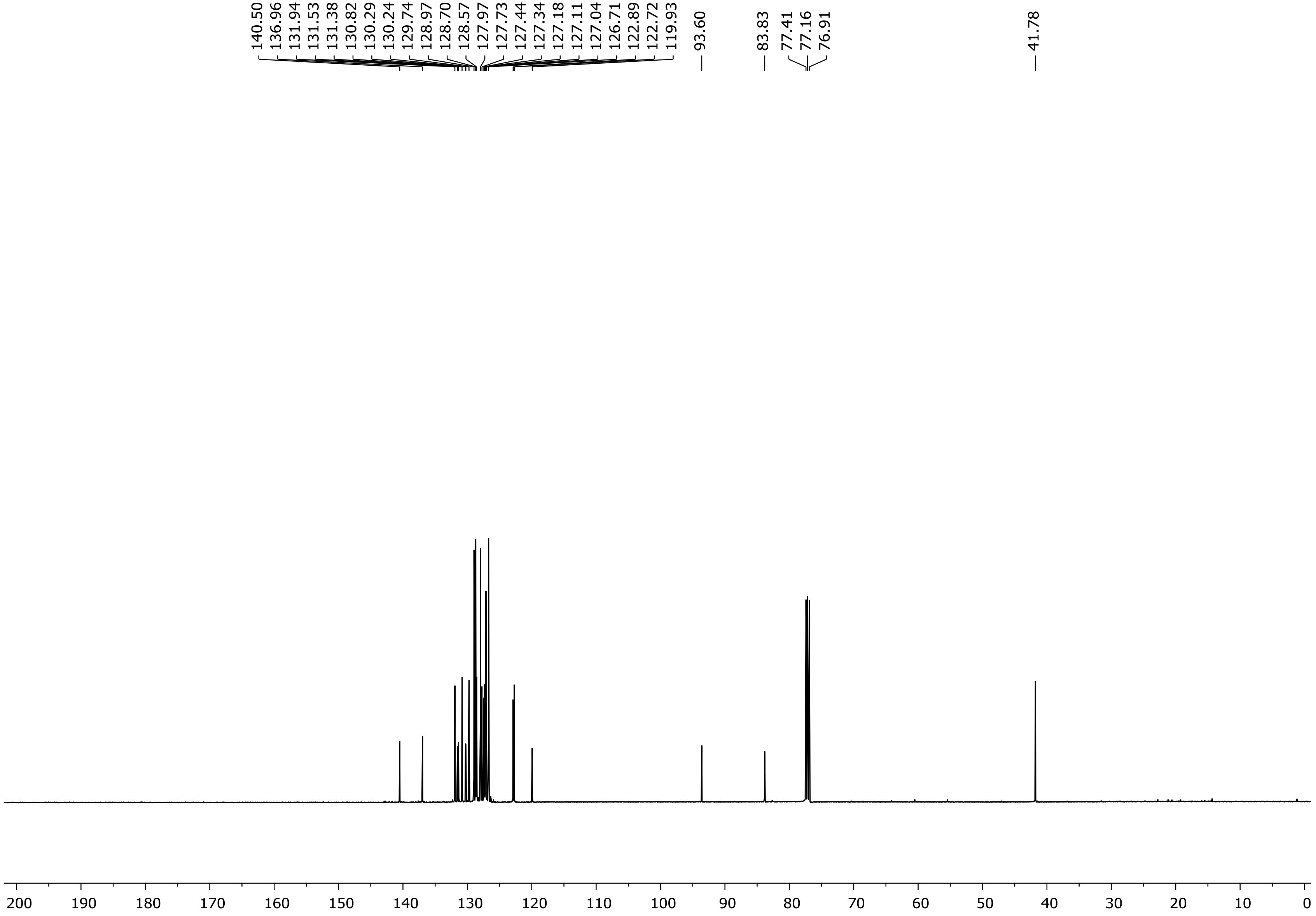

Figure S122: **^{1}H NMR** (500 MHz, $CDCl_3$, 298 K) spectrum of **2ag**.

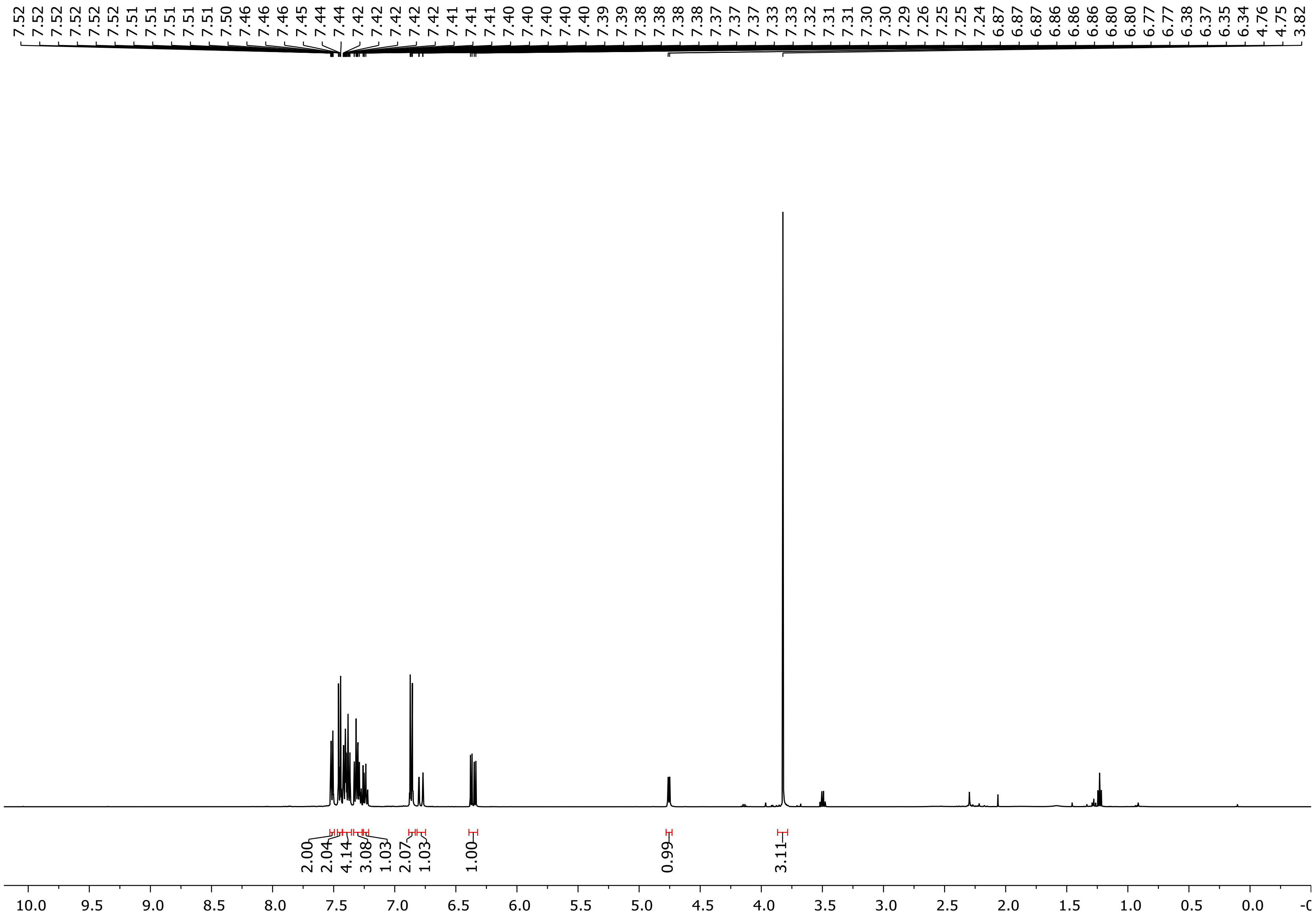

Figure S123: **^{13}C NMR** (126 MHz, $CDCl_3$, 298 K) spectrum of **2ag**.

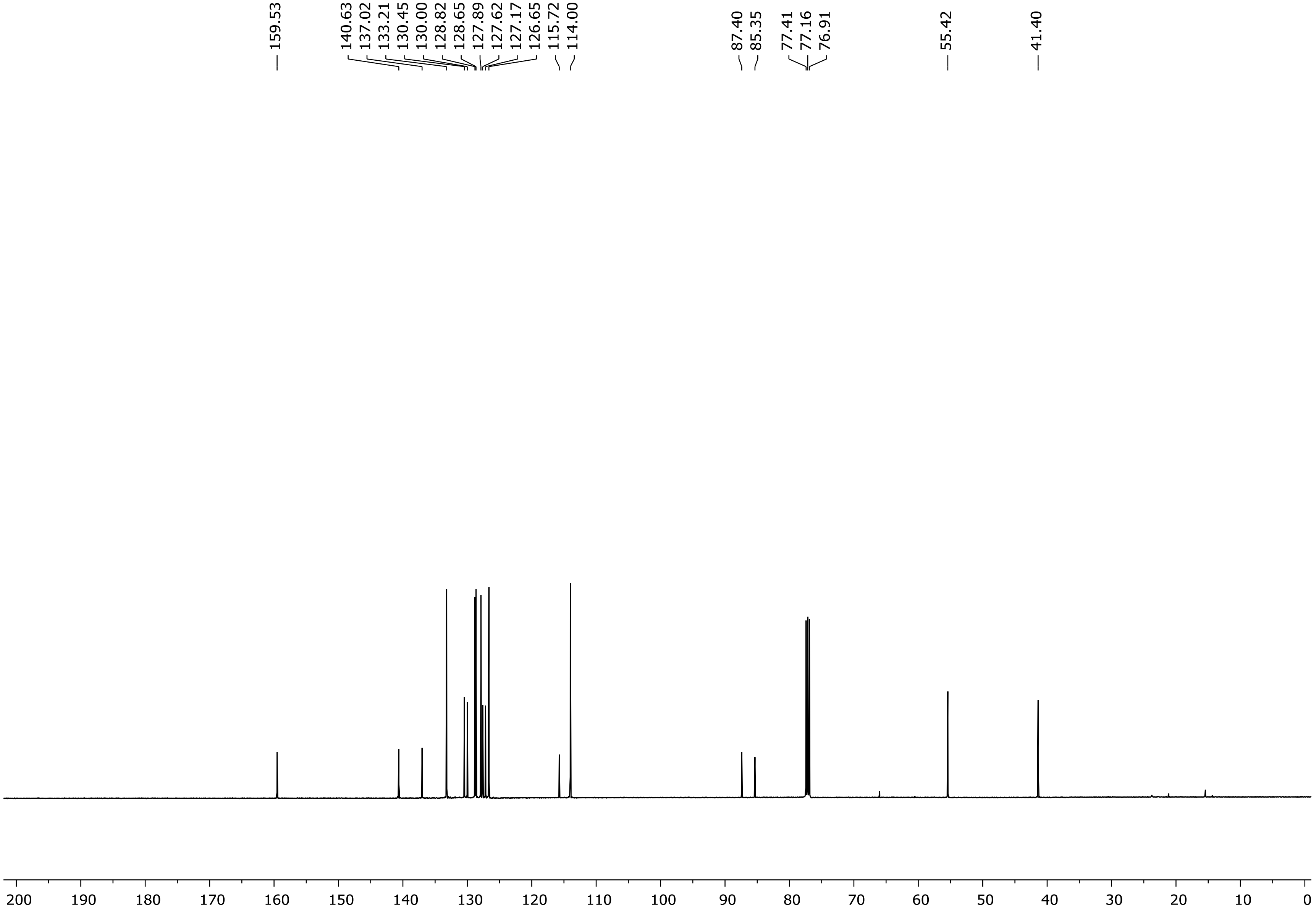

Figure S124: **^{1}H NMR** (500 MHz, $CDCl_3$, 298 K) spectrum of **2ah**.

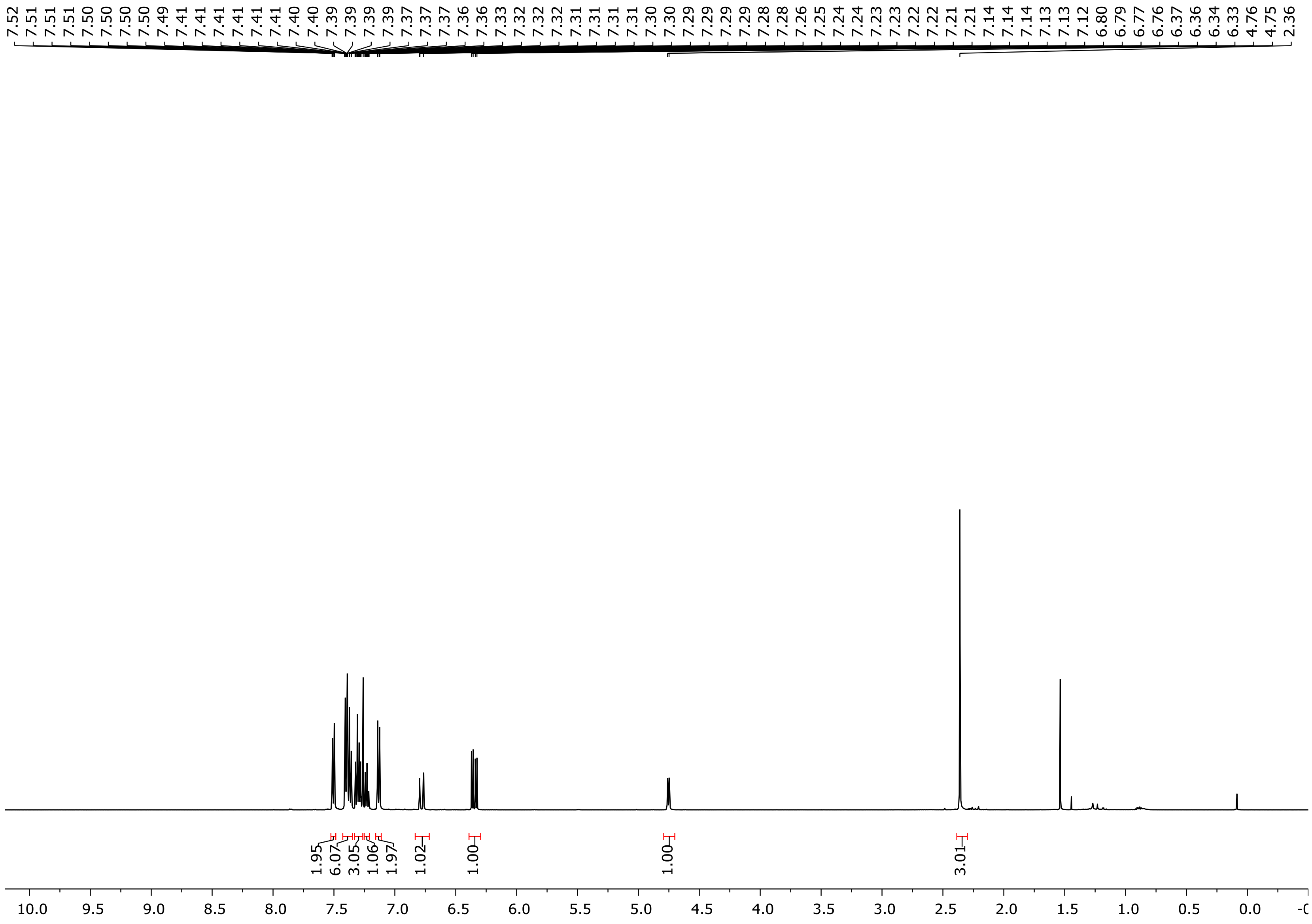

Figure S125: **^{13}C NMR** (126 MHz, $CDCl_3$, 298 K) spectrum of **2ah**.

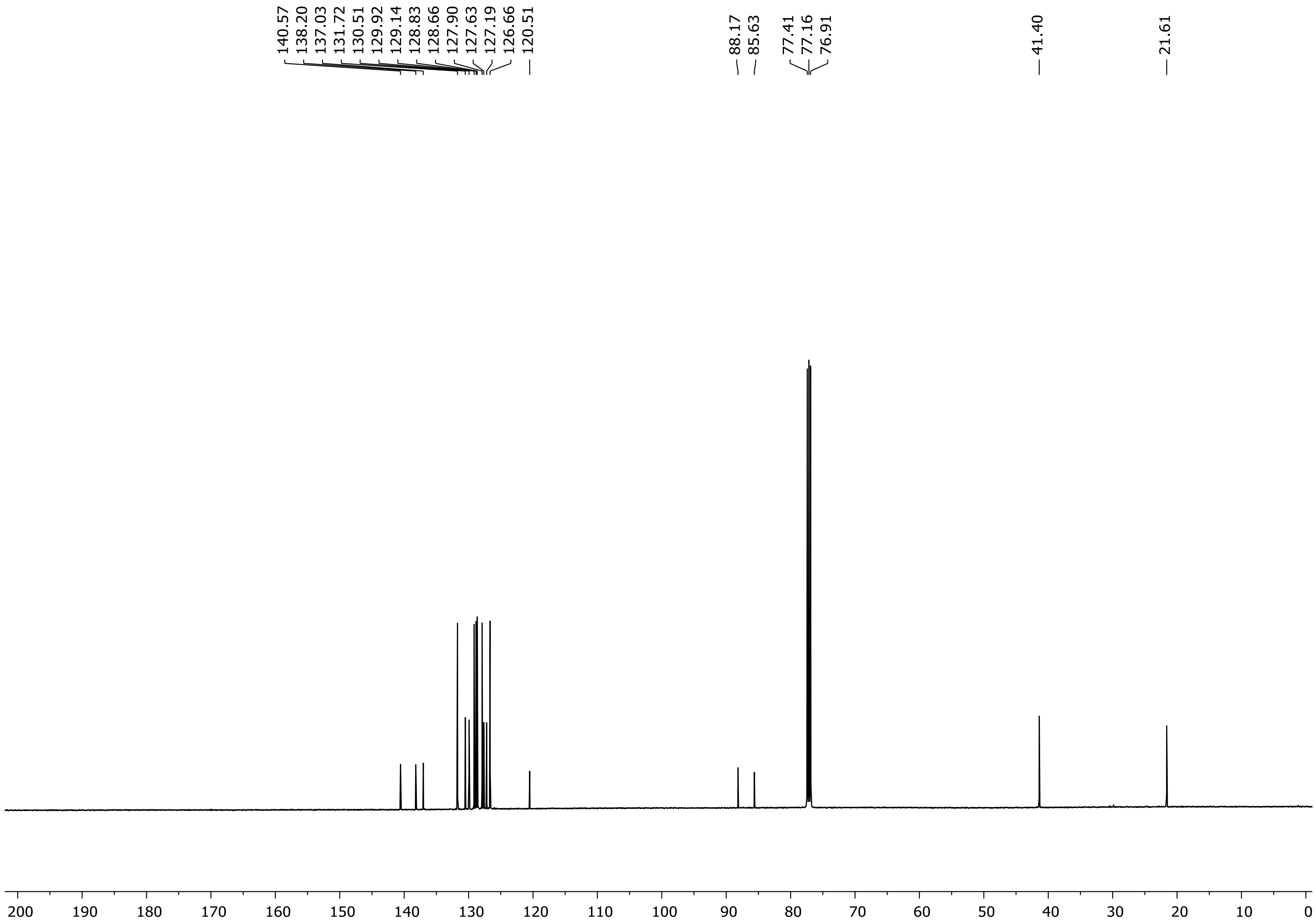

Figure S126: **^{1}H NMR** (500 MHz, $CDCl_3$, 298K) spectrum of **2ai**.

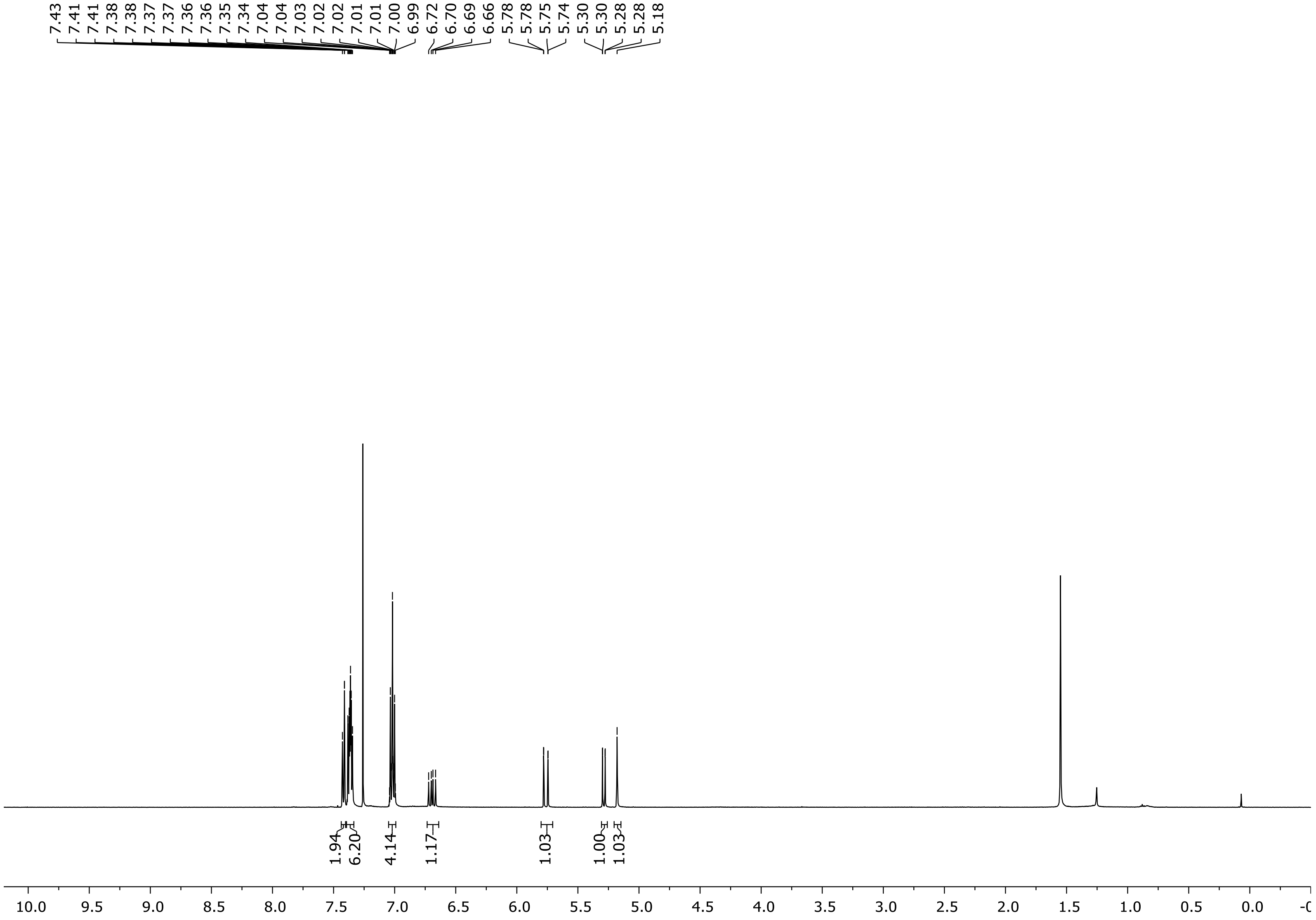

Figure S127: **^{13}C NMR** (126 MHz, $CDCl_3$, 298K) spectrum **2ai**.

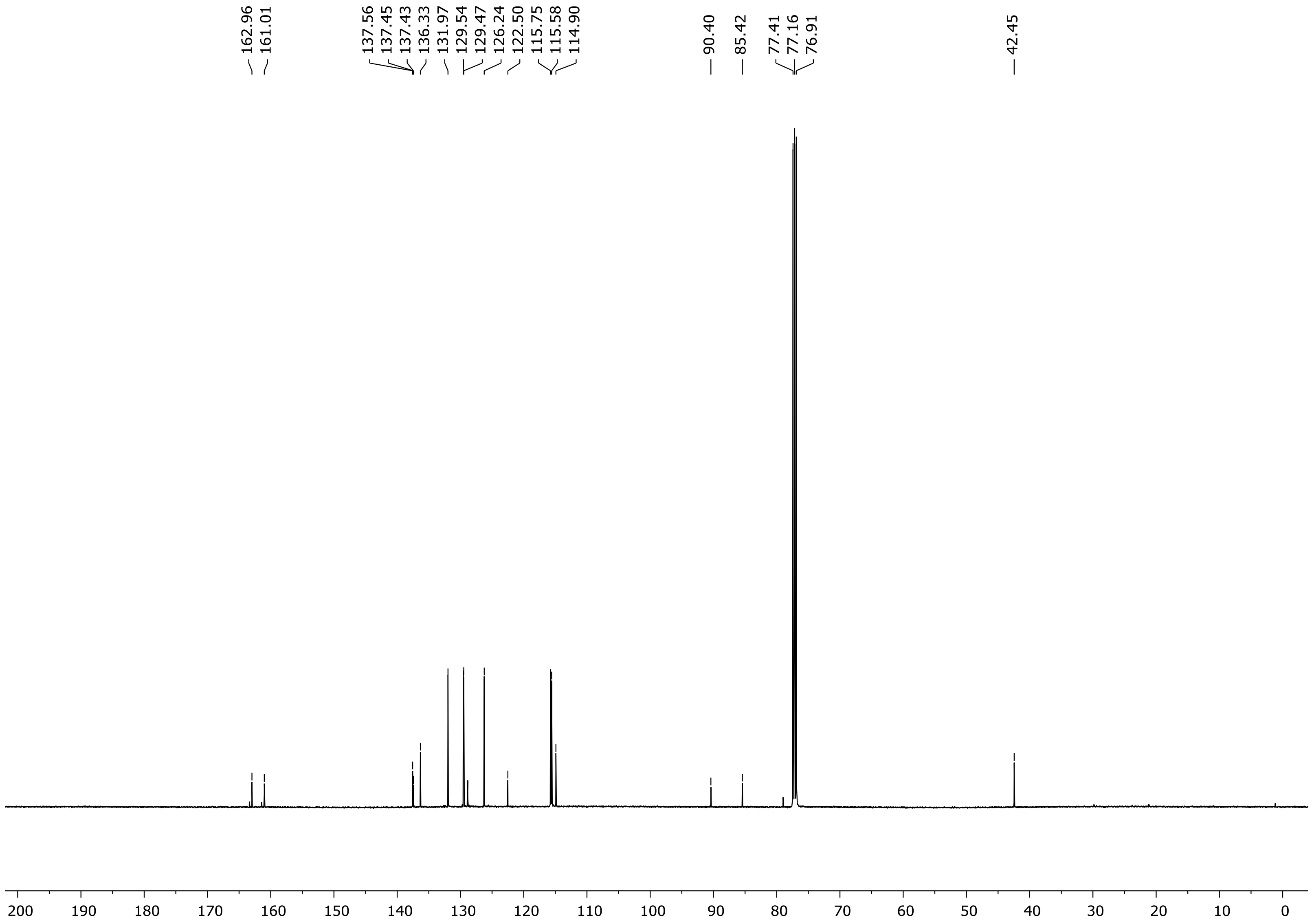

Figure S128: ^{19}**F NMR** (471 MHz, $CDCl_3$, 298K) spectrum of **2ai**.

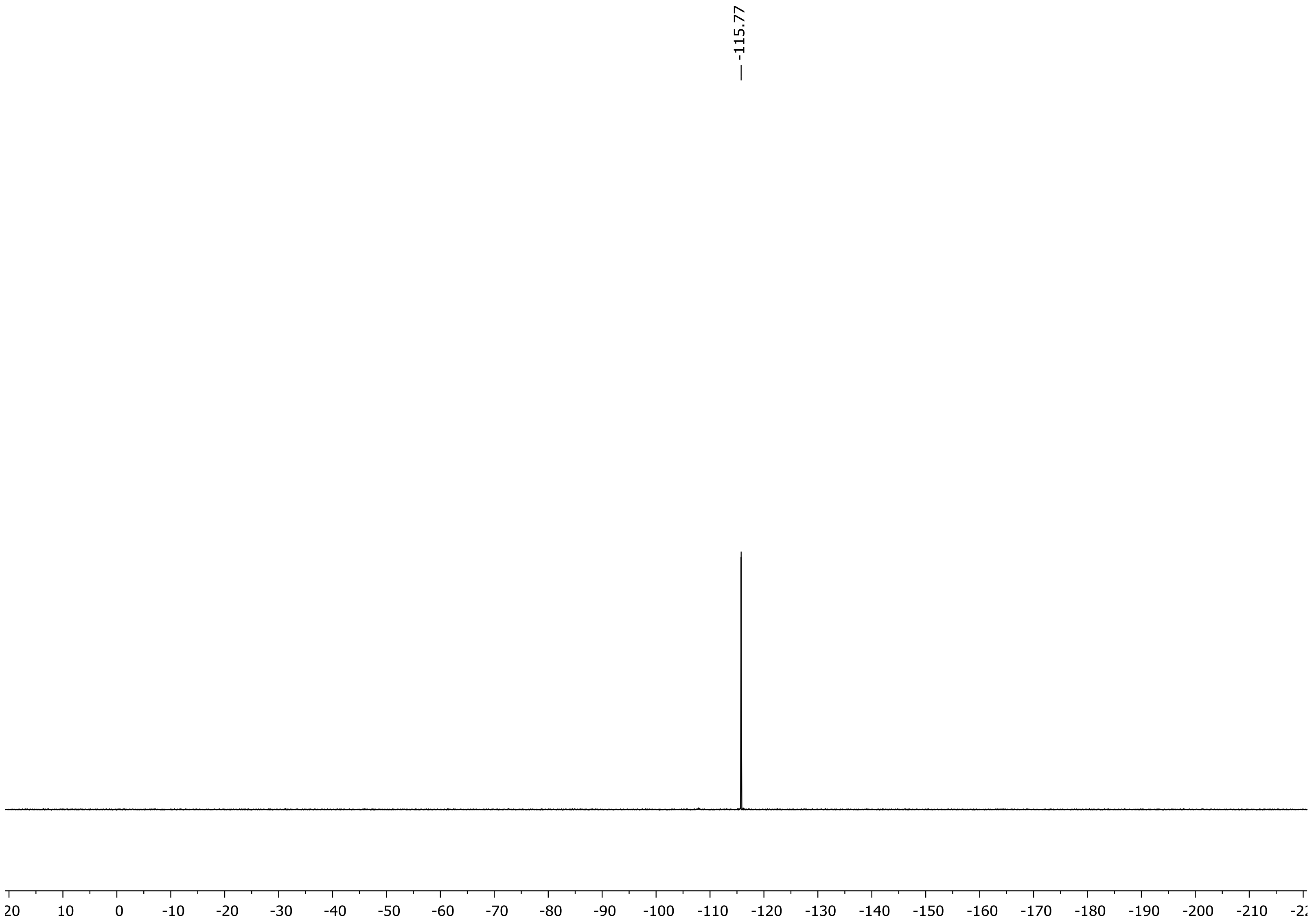

Figure S129: **^{1}H NMR** (500 MHz, $CDCl_3$, 298K) spectrum of **2aj**.

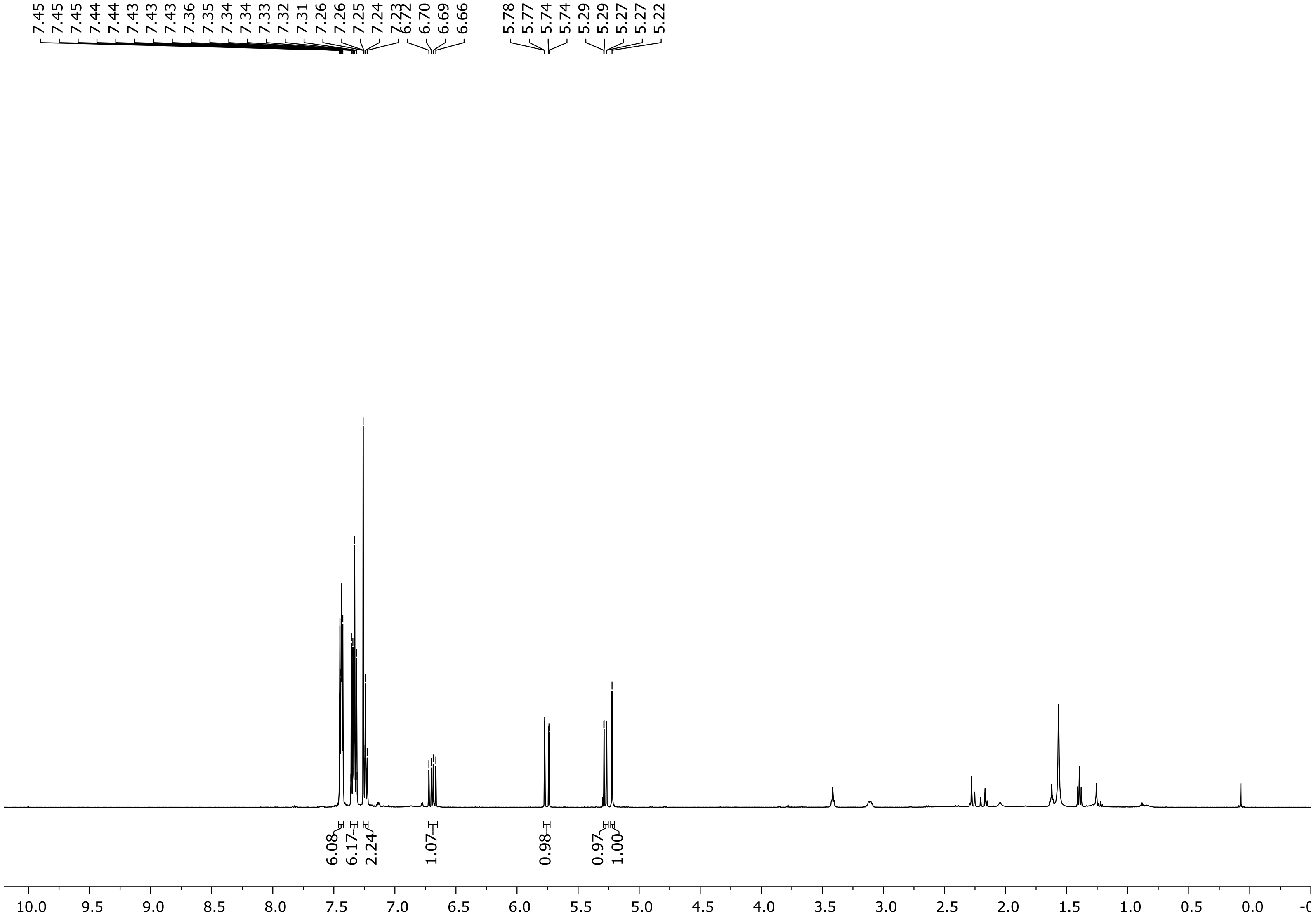

Figure S130: **^{13}C NMR** (126 MHz, $CDCl_3$, 298K) spectrum **2aj**.

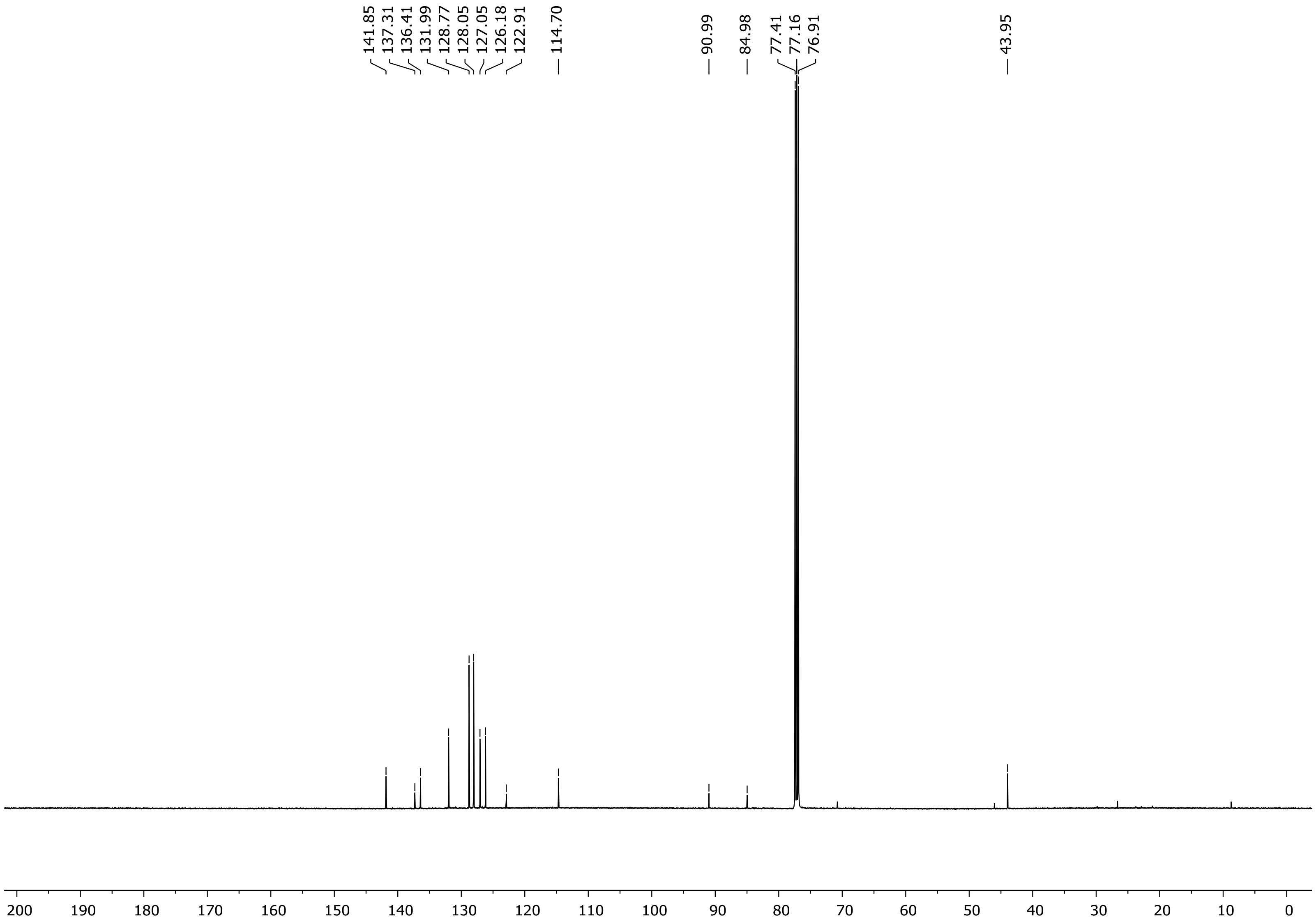

Figure S131: **^{1}H NMR** (500 MHz, $CDCl_3$, 298K) spectrum of **2ak**.

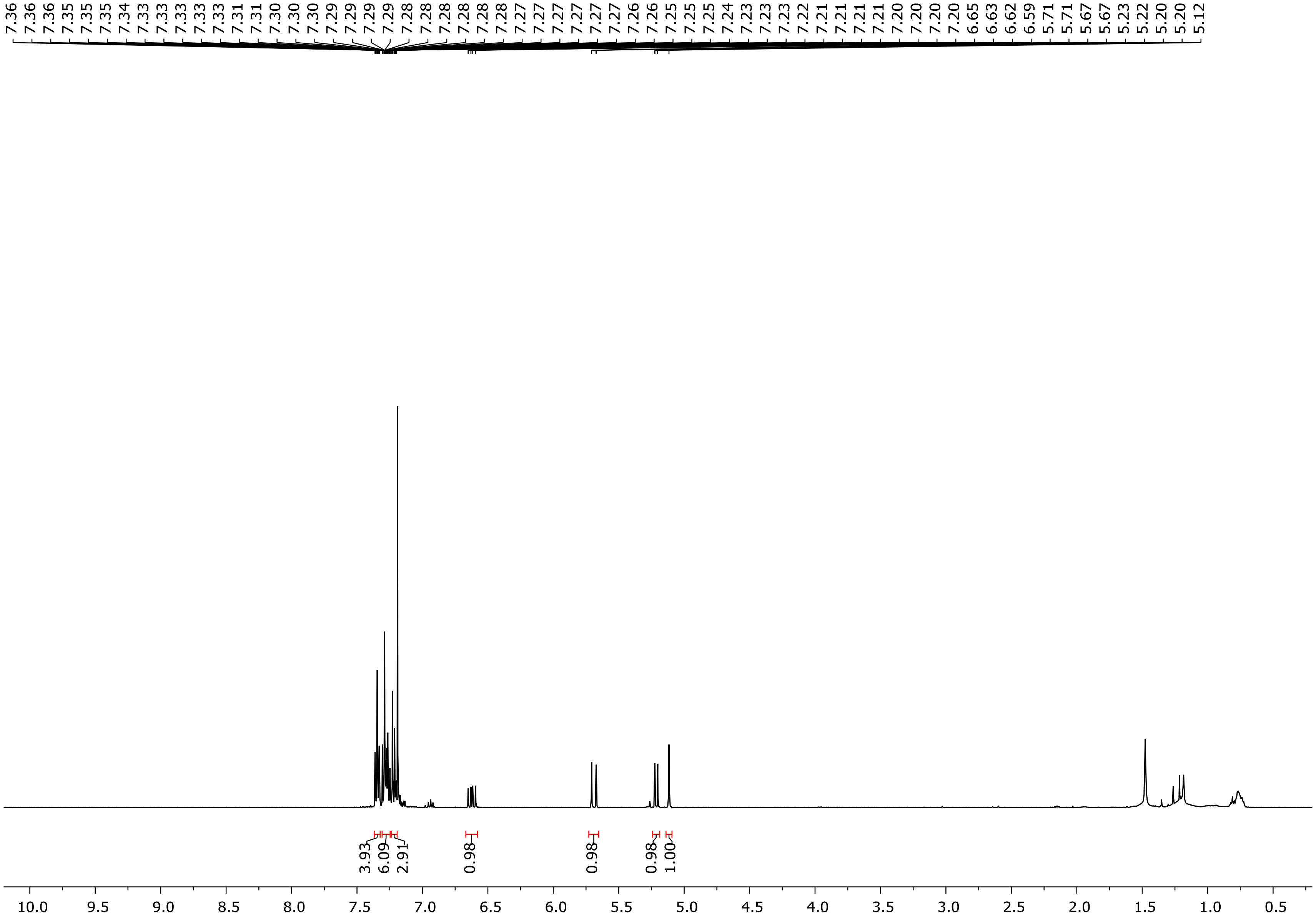

Figure S132: **^{13}C NMR** (126 MHz, $CDCl_3$, 298K) spectrum **2ak**.

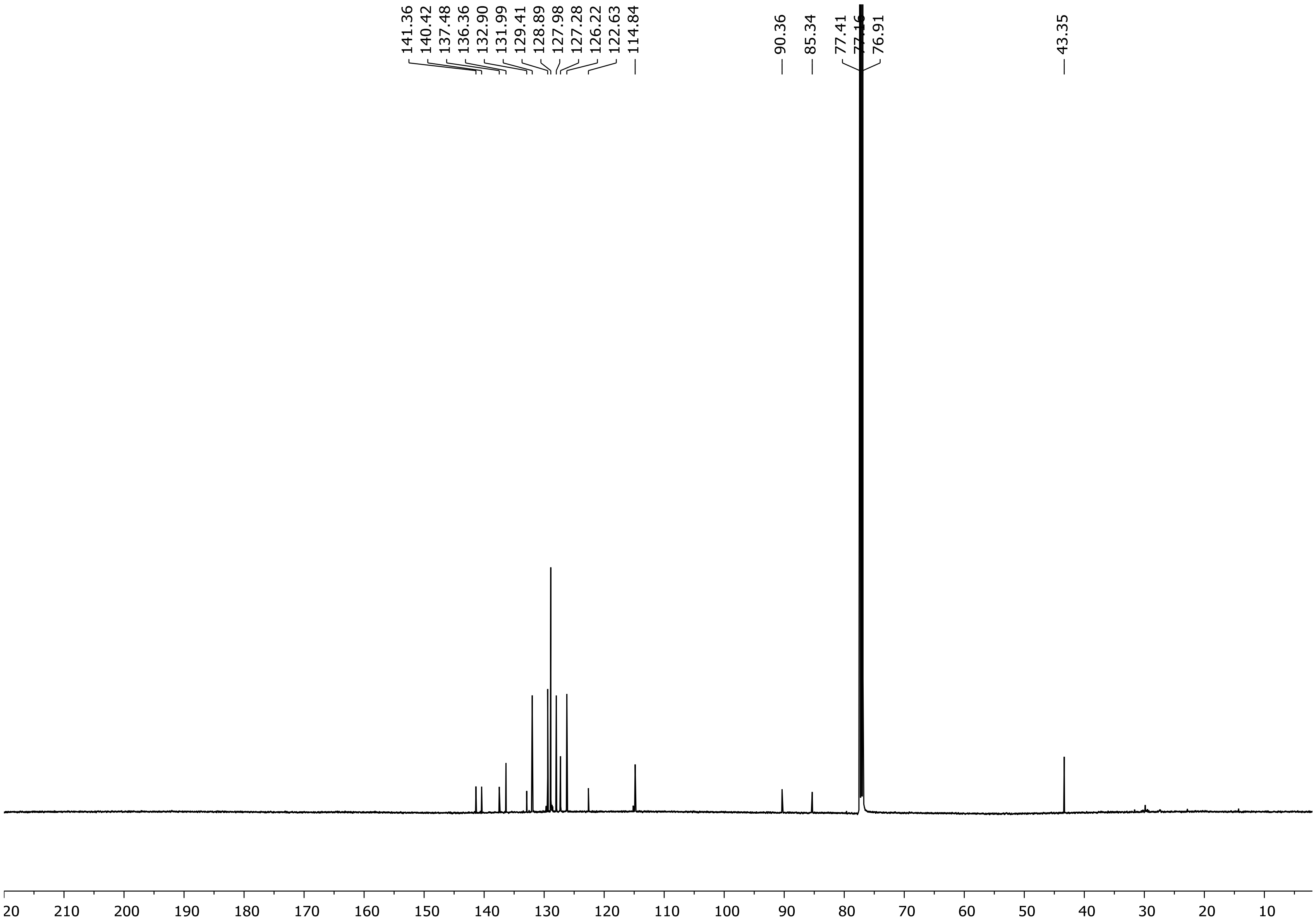

Figure S133: **^{1}H NMR** (500 MHz, $CDCl_3$, 298K) spectrum of **2al**.

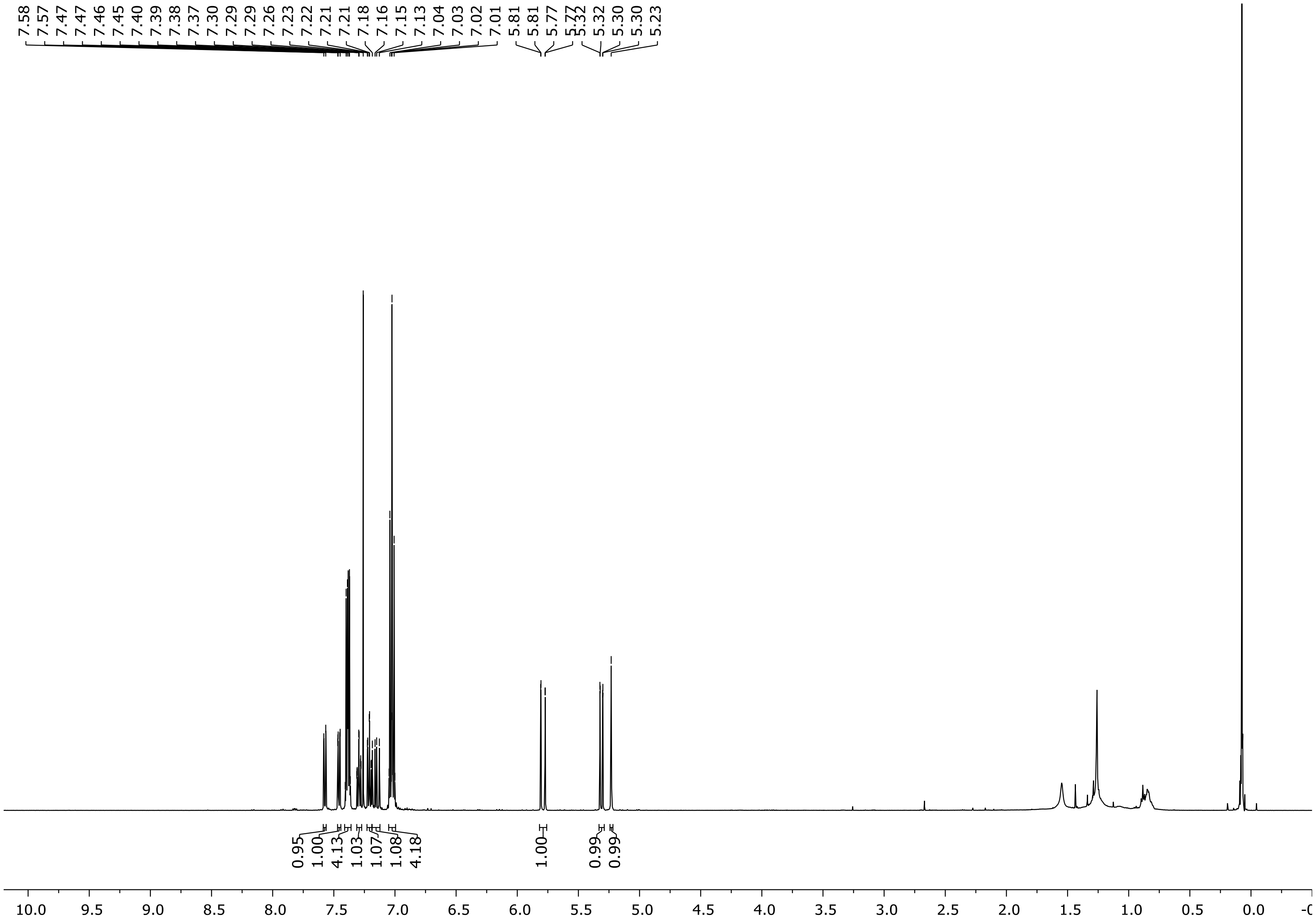

Figure S134: **^{13}C NMR** (126 MHz, $CDCl_3$, 298K) spectrum **2al**.

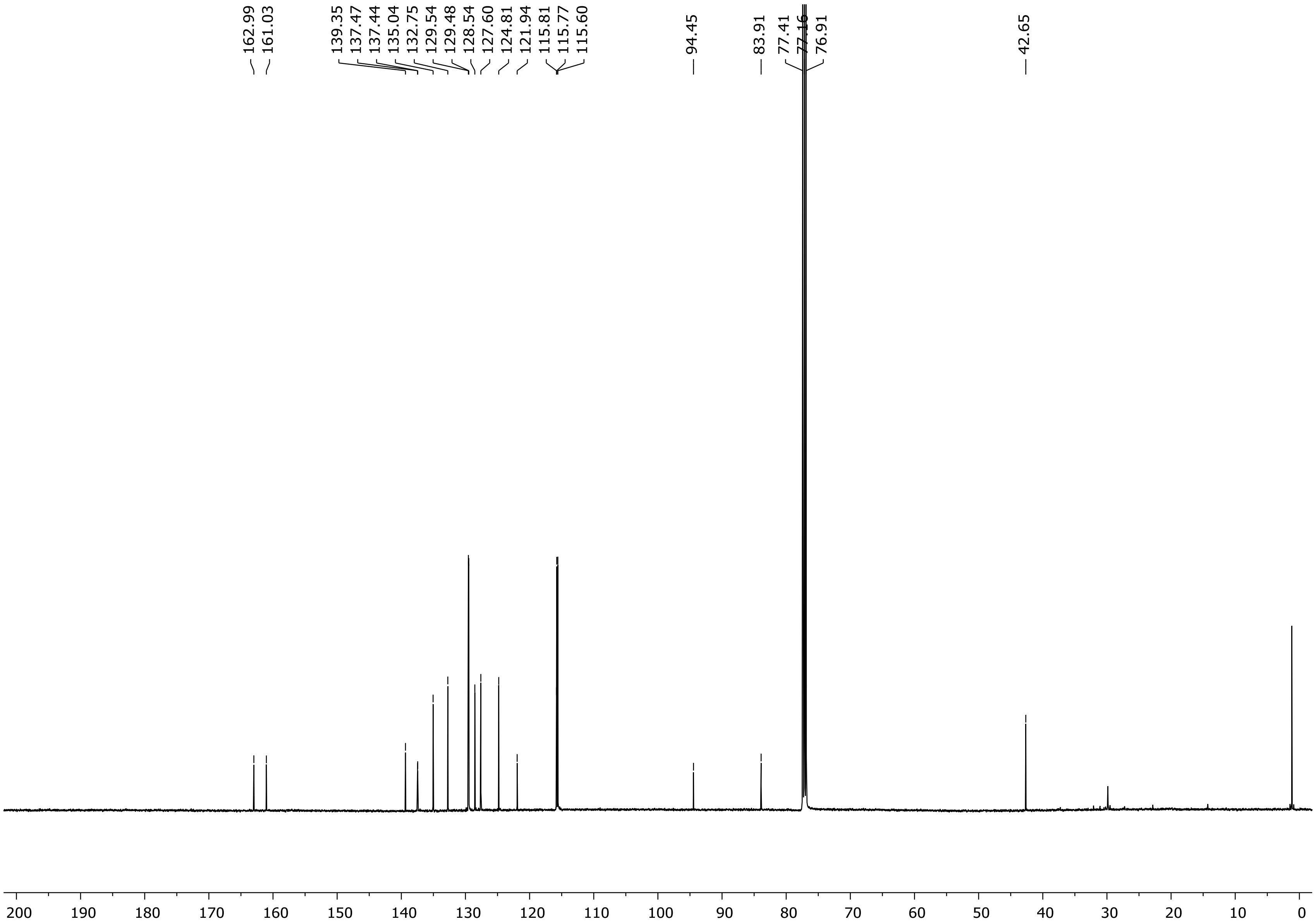

Figure S135: **^{19}F NMR** (471 MHz, $CDCl_3$, 298K) spectrum of **2al**.

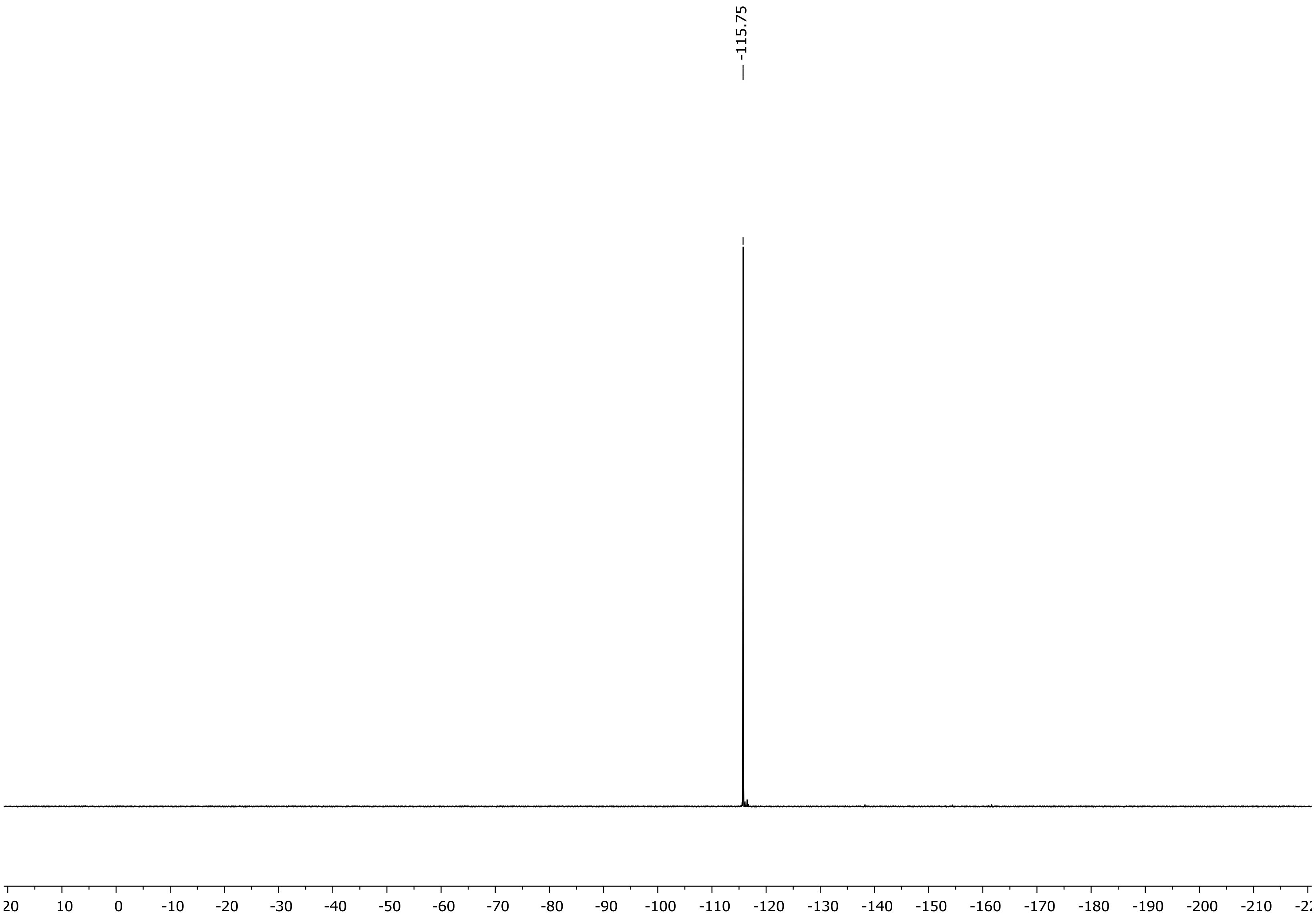

Figure S136: **^{1}H NMR** (500 MHz, $CDCl_3$, 298K) spectrum of **2am**.

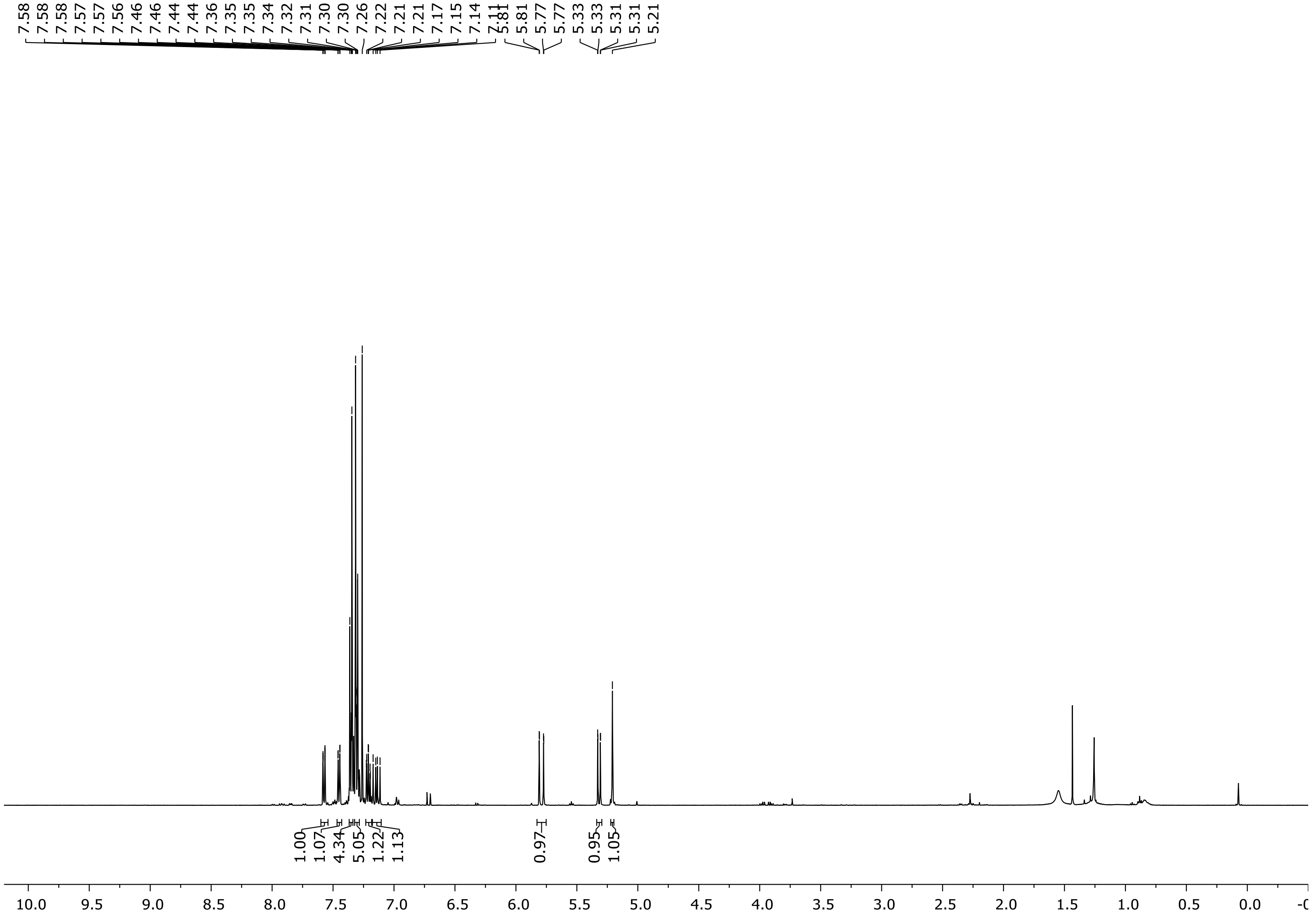

Figure S137: **^{13}C NMR** (126 MHz, $CDCl_3$, 298K) spectrum **2am**.

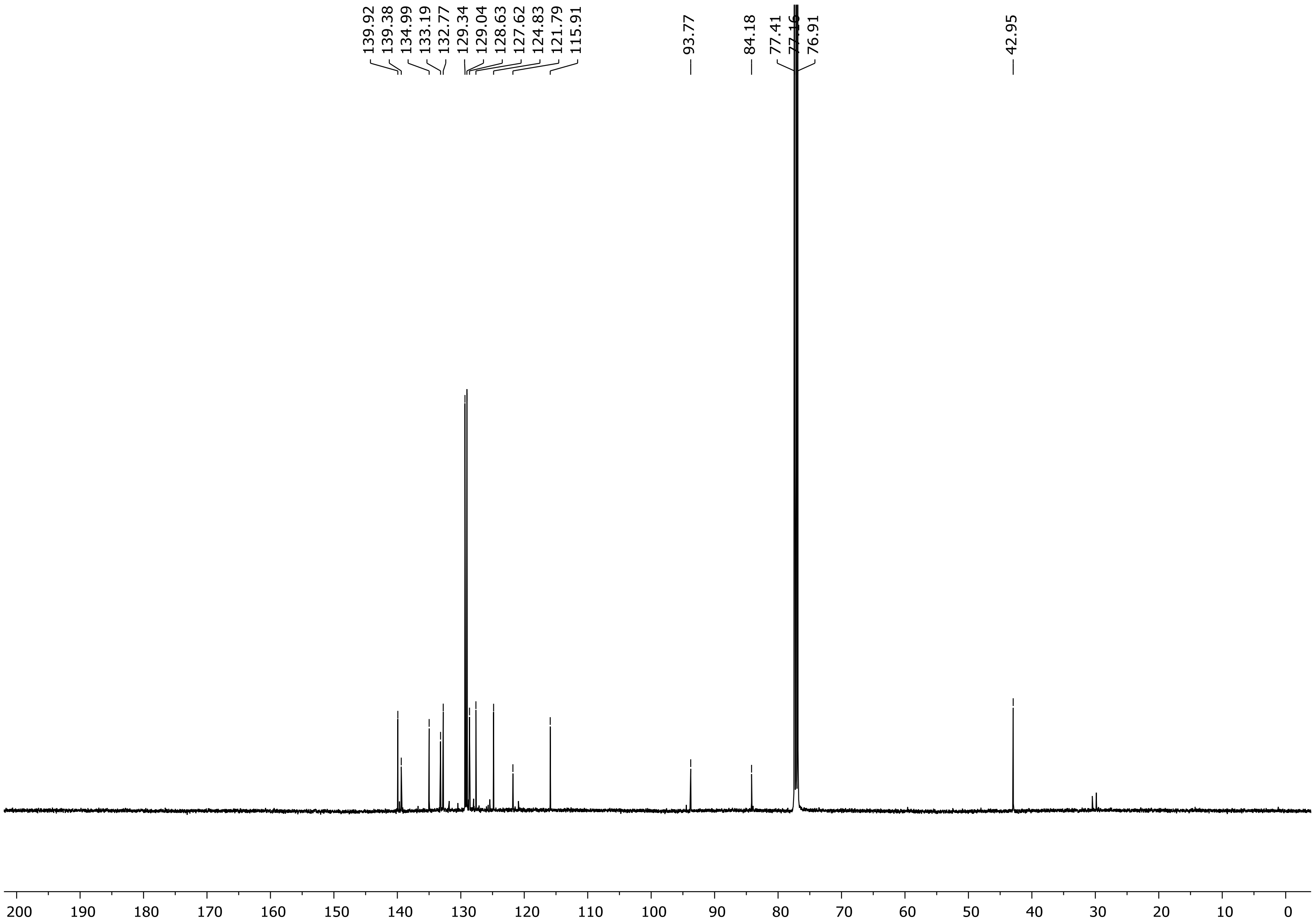

Figure S138: **^{1}H NMR** (500 MHz, $CDCl_3$, 298K) spectrum of **2an**.

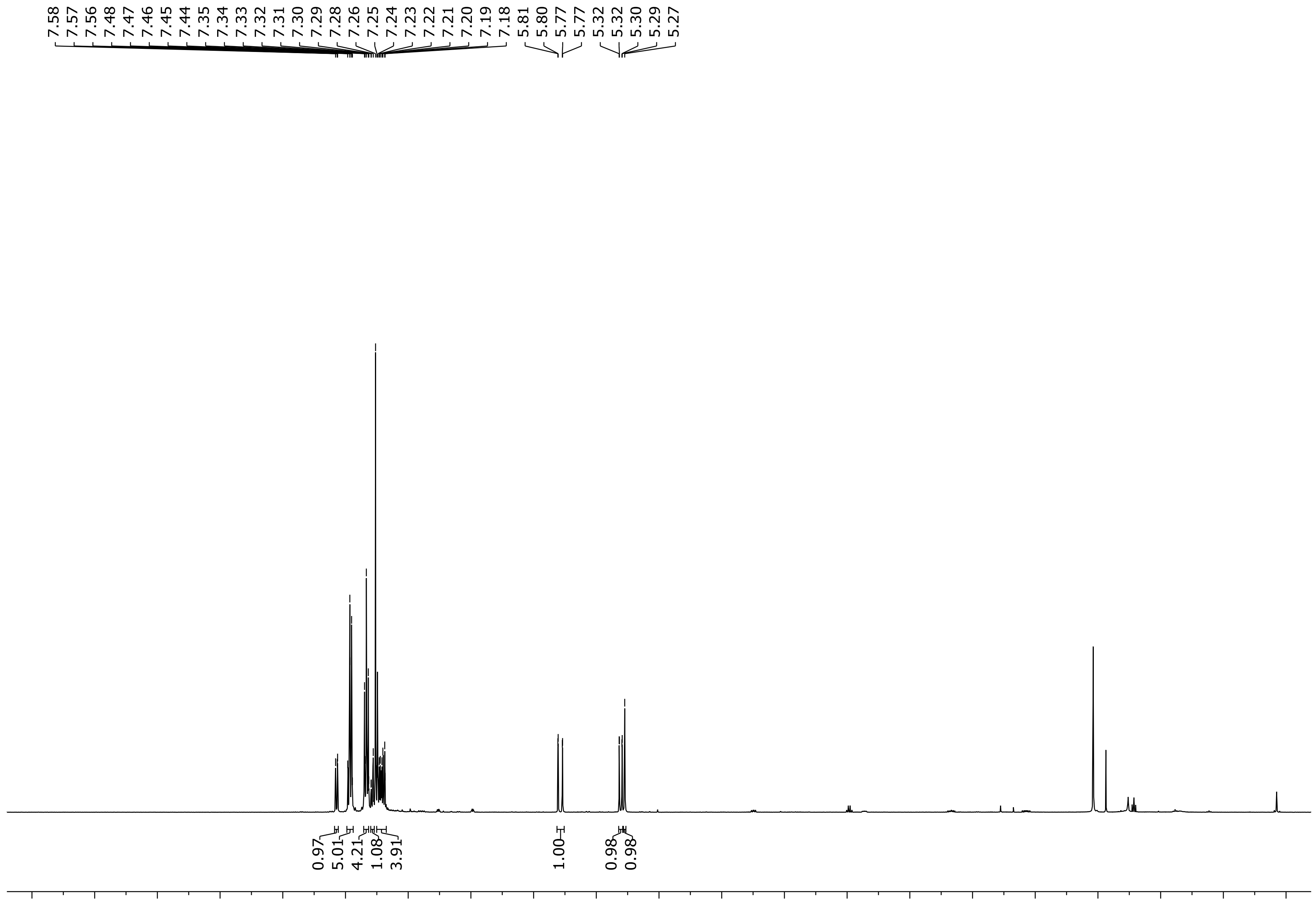

Figure S139: **^{13}C NMR** (126 MHz, $CDCl_3$, 298K) spectrum of **2an**.

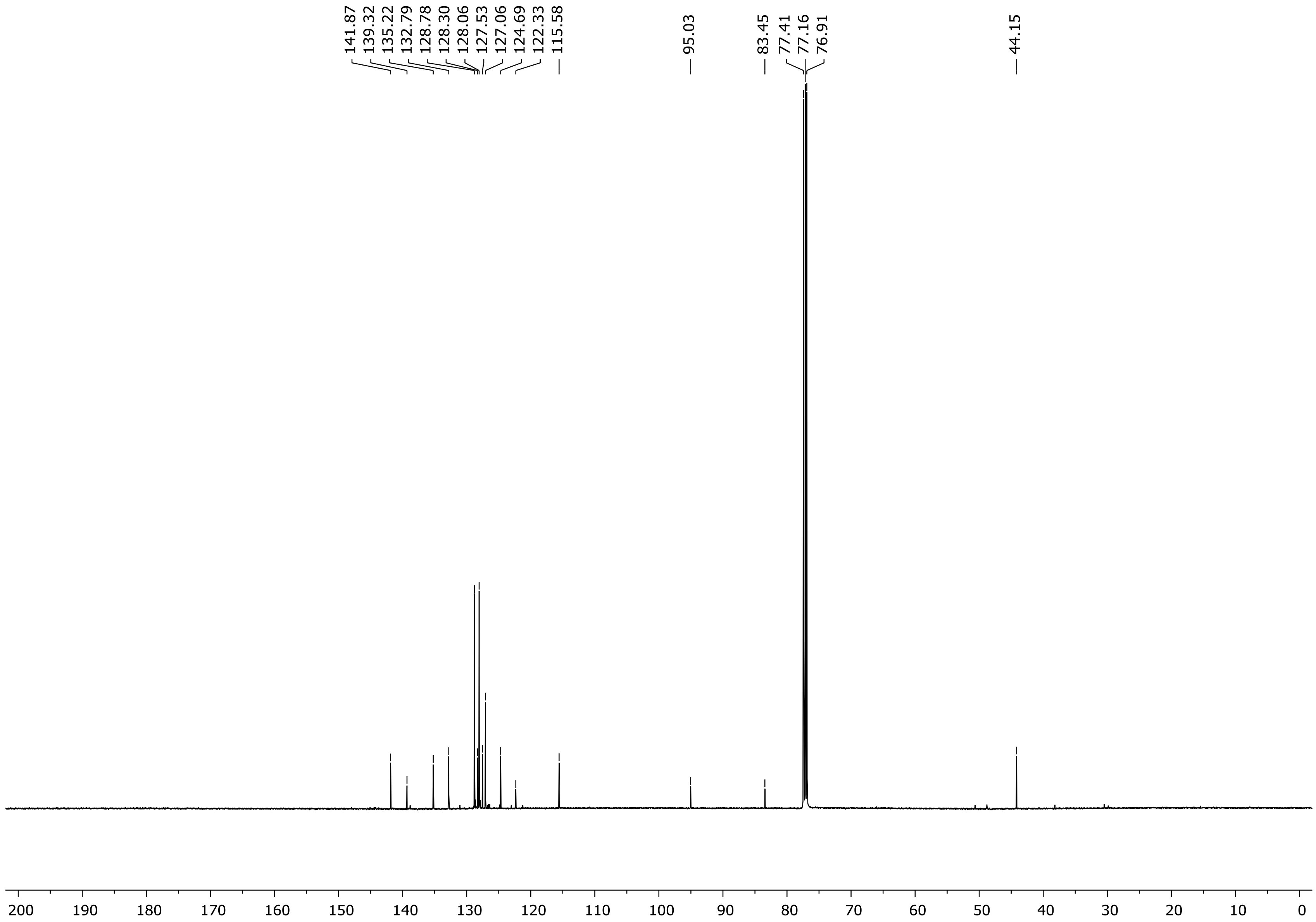

Figure S140: **^{1}H NMR** (500 MHz, $CDCl_3$, 298K) spectrum of **2ao**.

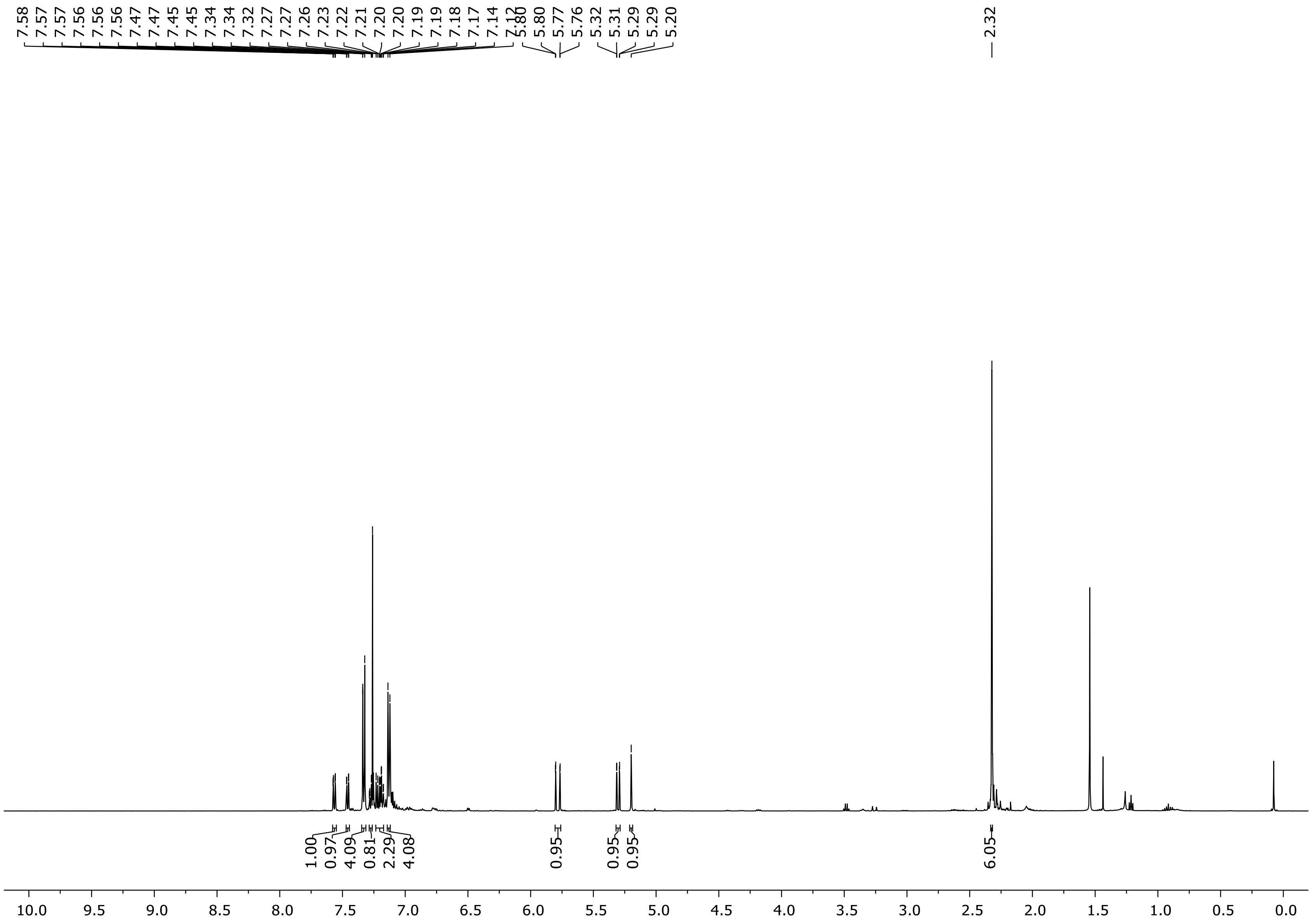

Figure S141: **^{13}C NMR** (126 MHz, $CDCl_3$, 298K) spectrum of **2ao**.

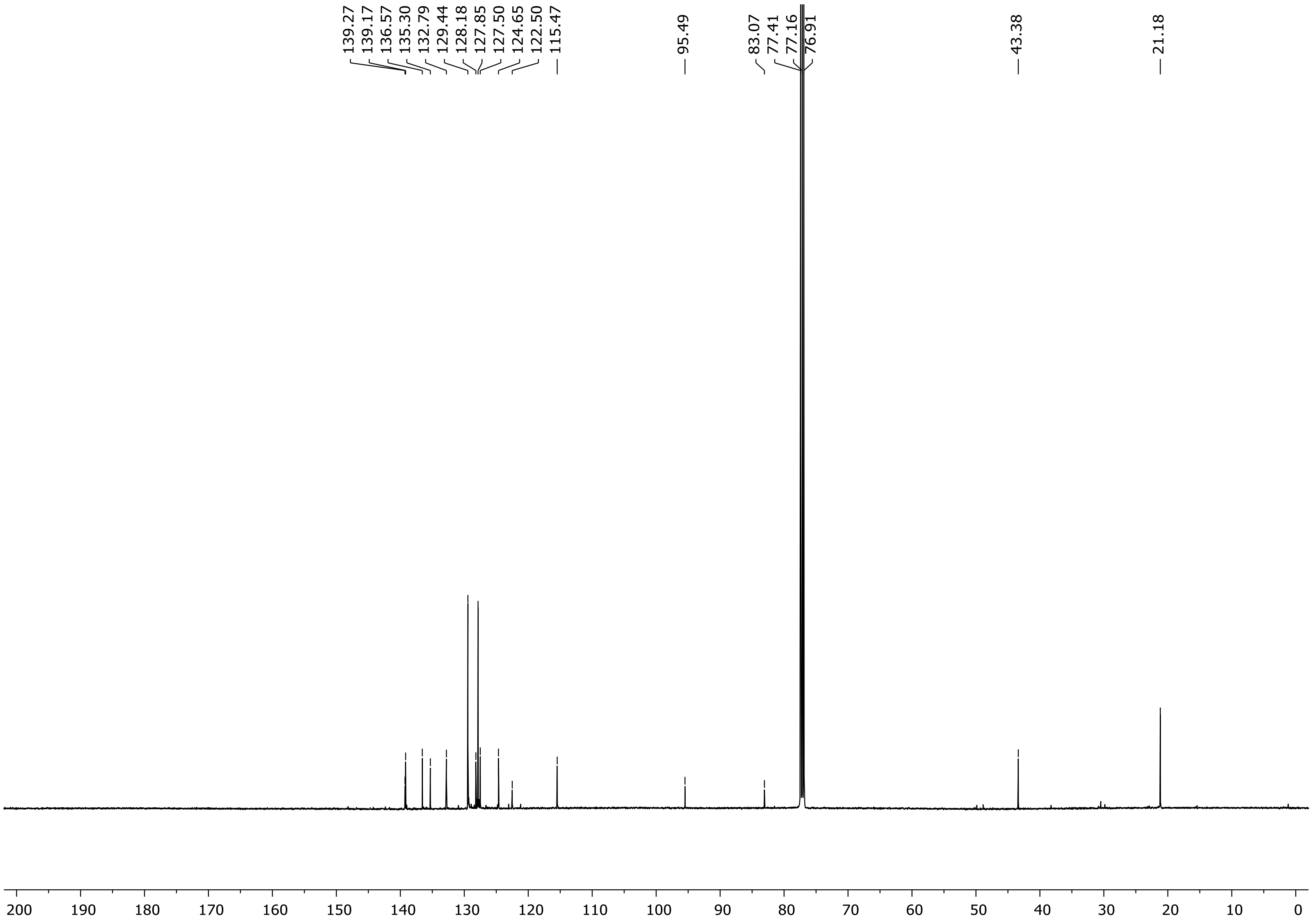

Figure S142: ^{1}**H NMR** (500 MHz, $CDCl_3$, 298K) spectrum of **2ap**.

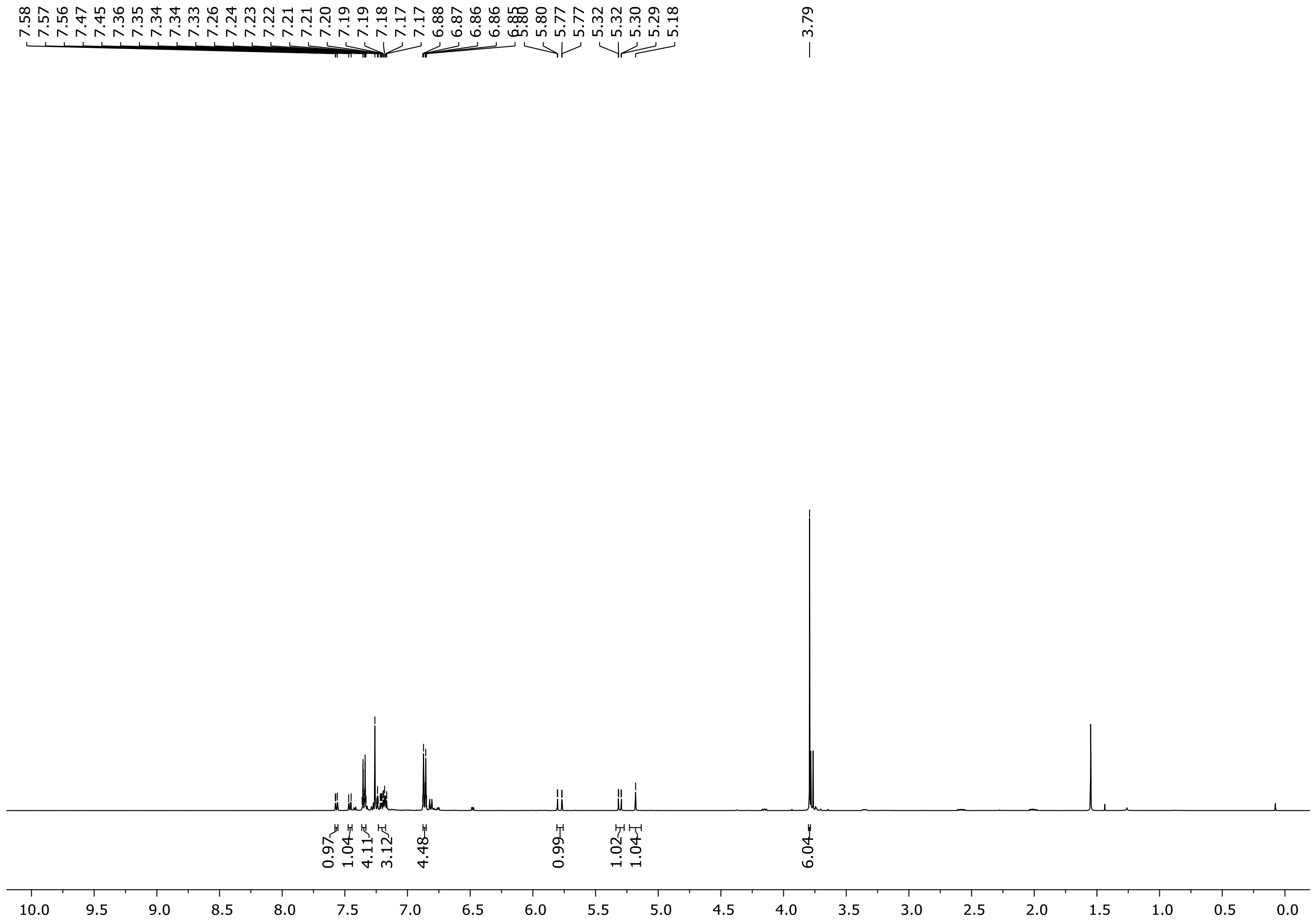

Figure S143: **^{13}C NMR** (126 MHz, $CDCl_3$, 298K) spectrum of **2ap**.

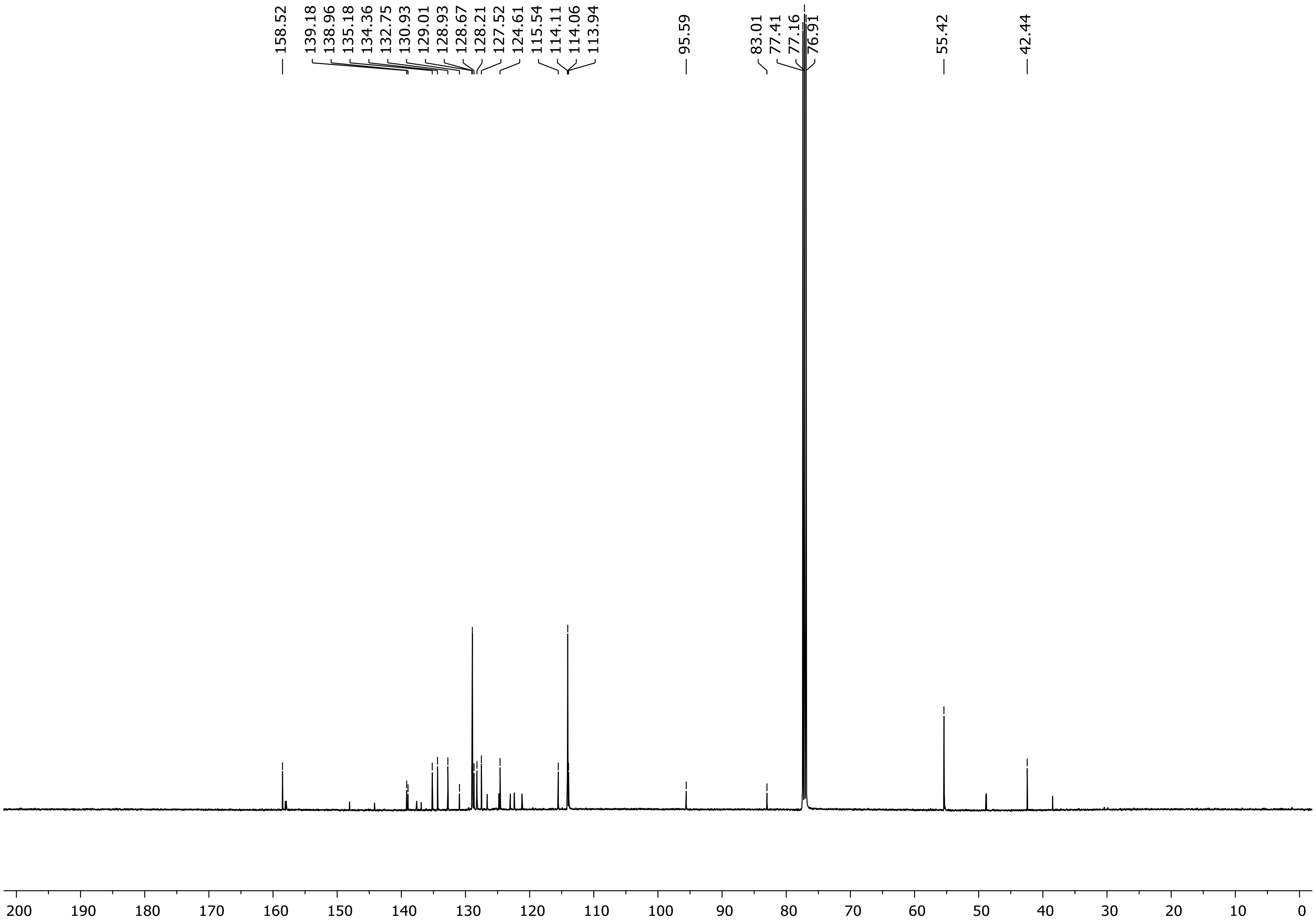

Figure S144: **^{1}H NMR** (500 MHz, $CDCl_3$, 298K) spectrum of **2aq**.

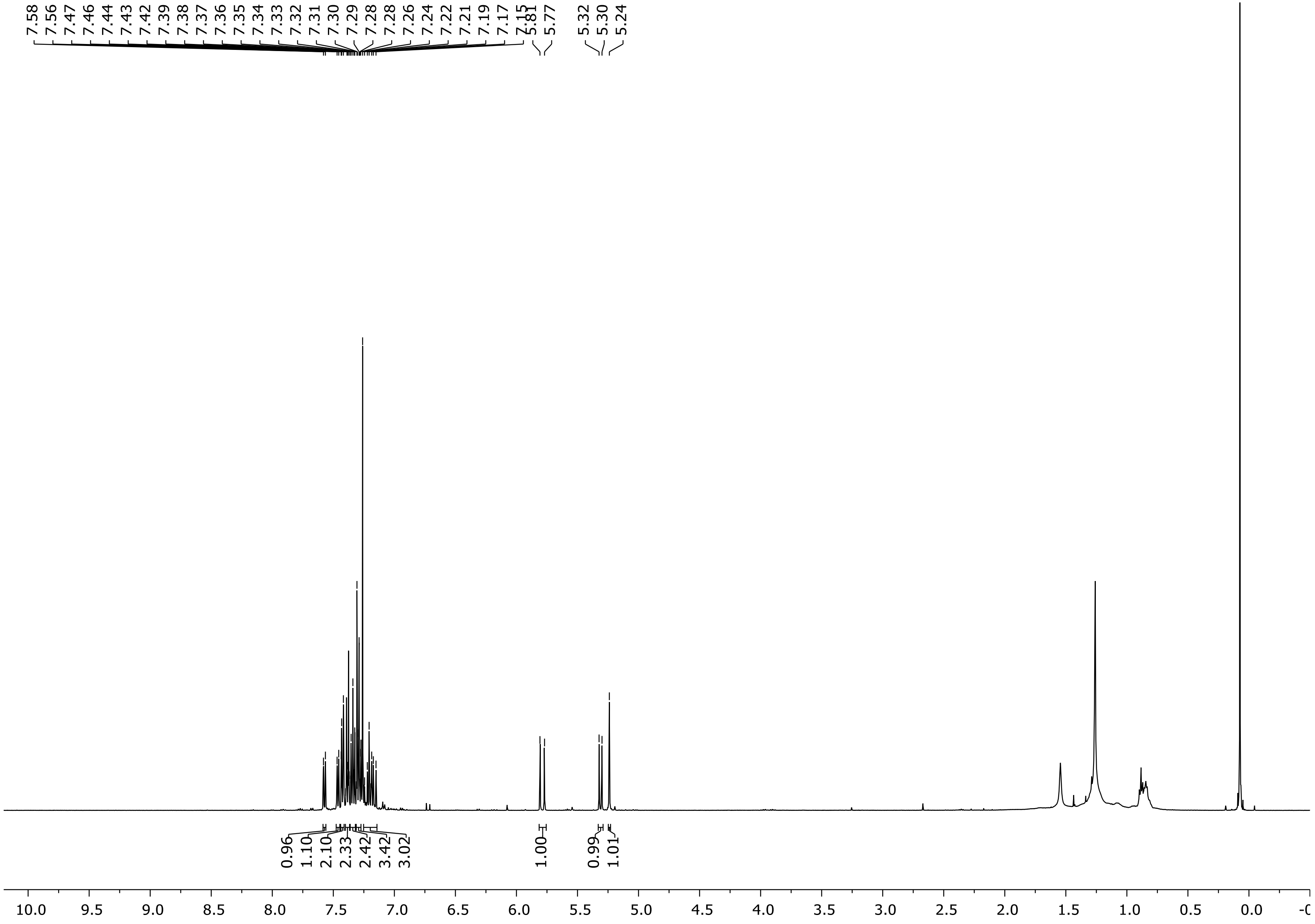

Figure S145: **^{13}C NMR** (126 MHz, $CDCl_3$, 298K) spectrum of **2aq**.

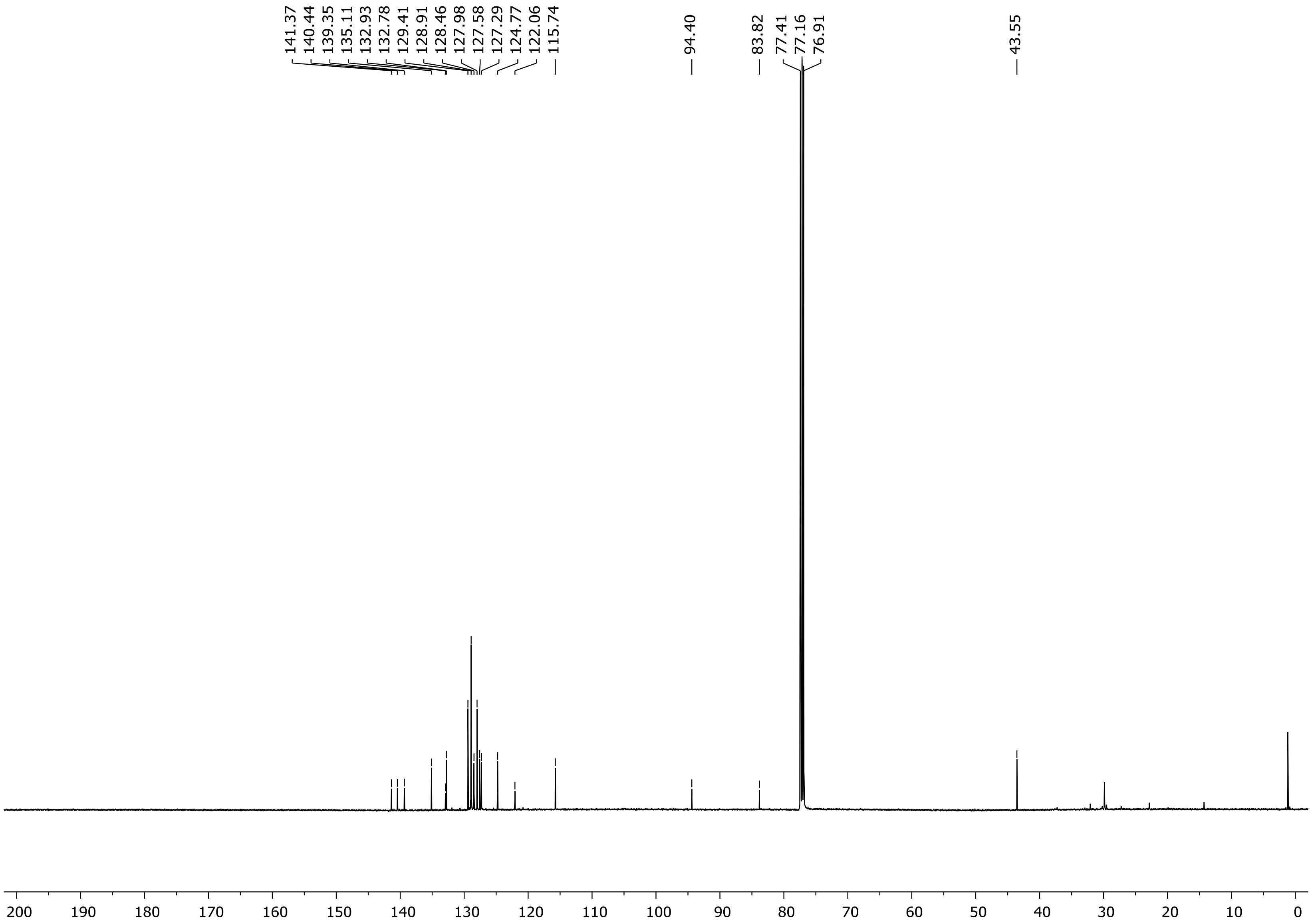

Figure S146: **^{1}H NMR** (500 MHz, $CDCl_3$, 298K) spectrum of **2ar**.

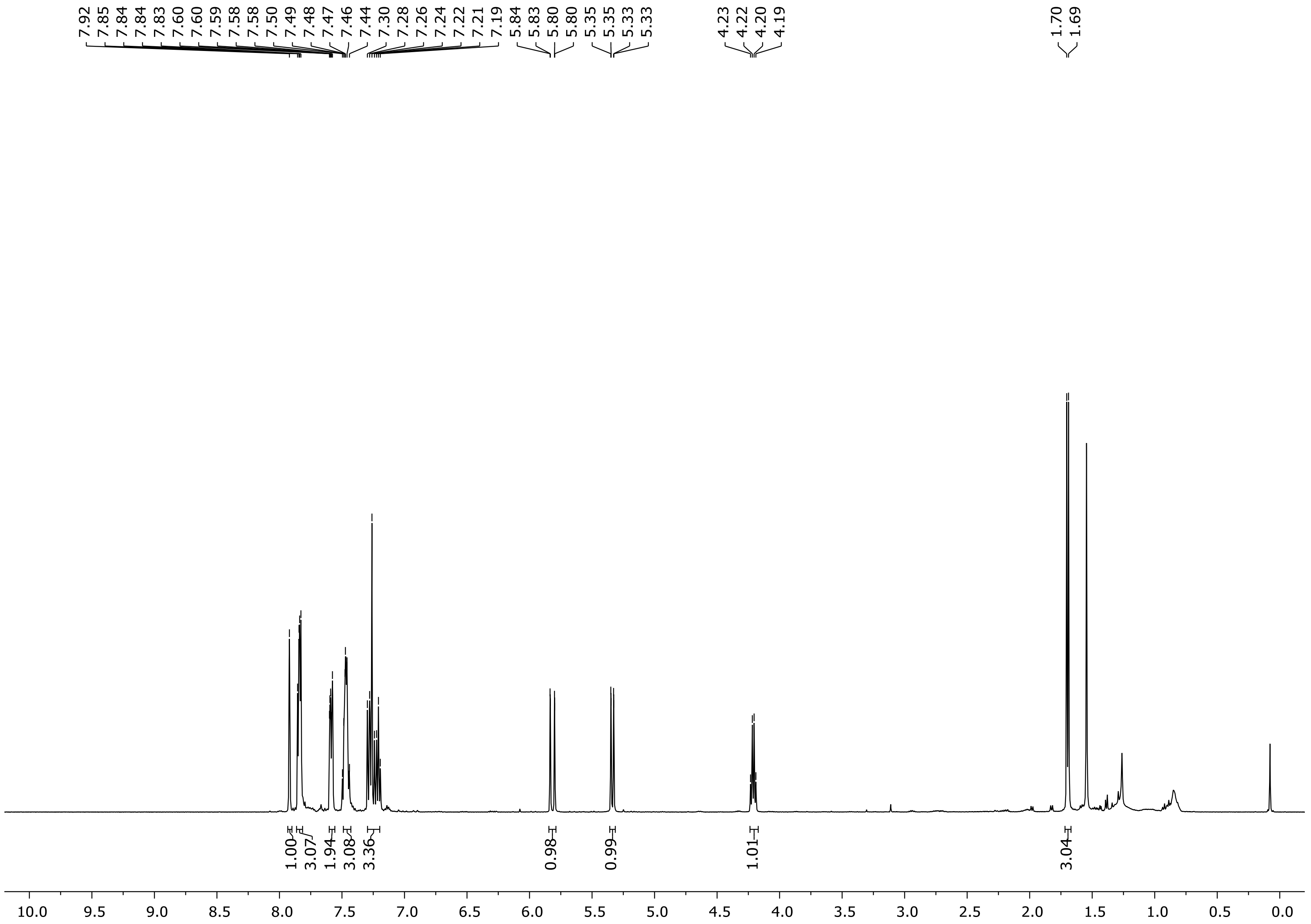

Figure S147: **^{13}C NMR** (126 MHz, $CDCl_3$, 298K) spectrum of **2ar**.

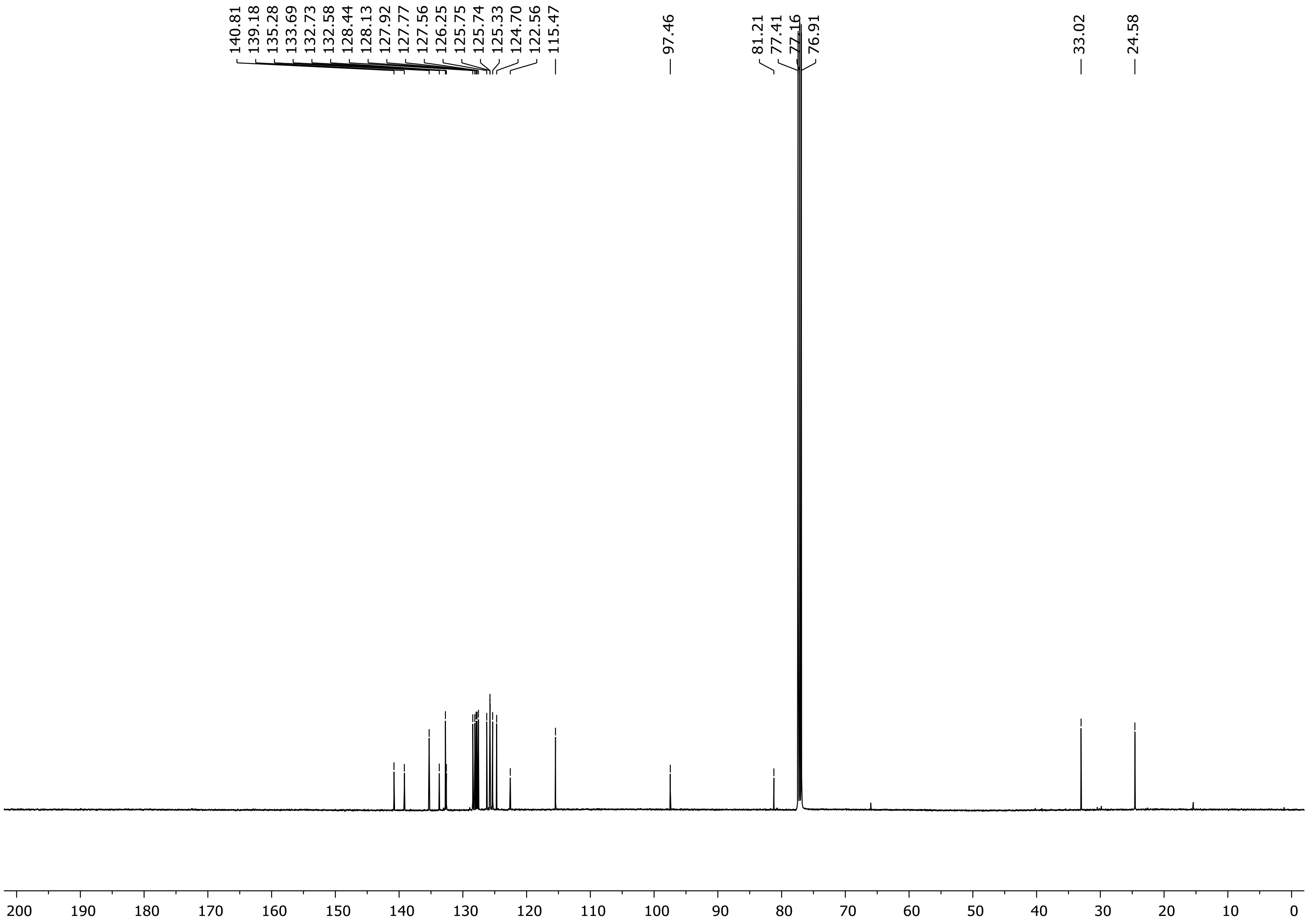

Figure S148: **^{1}H NMR** (500 MHz, $CDCl_3$, 298K) spectrum of **3a**.

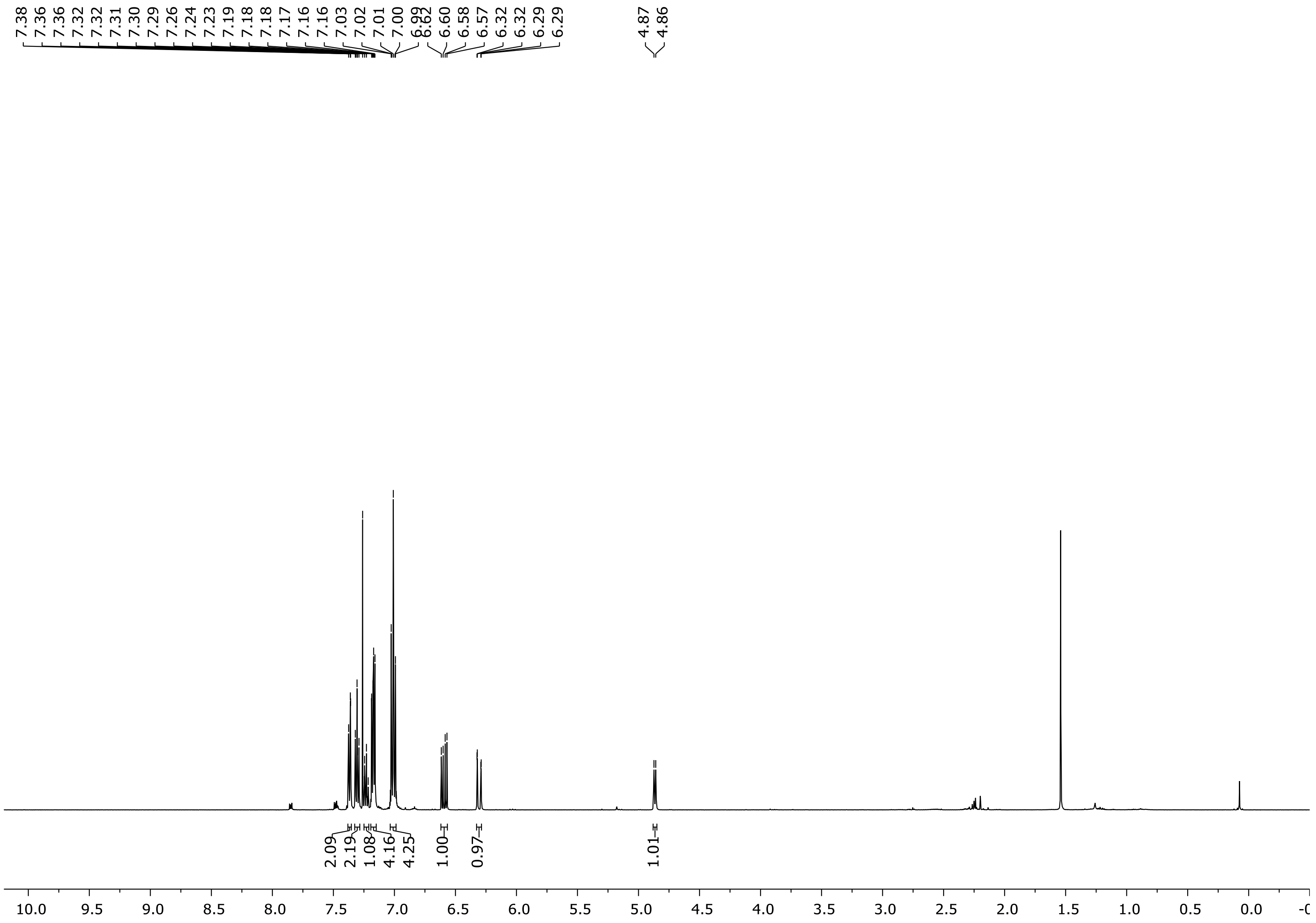

Figure S149: **^{13}C NMR** (126 MHz, $CDCl_3$, 298K) spectrum of **3a**.

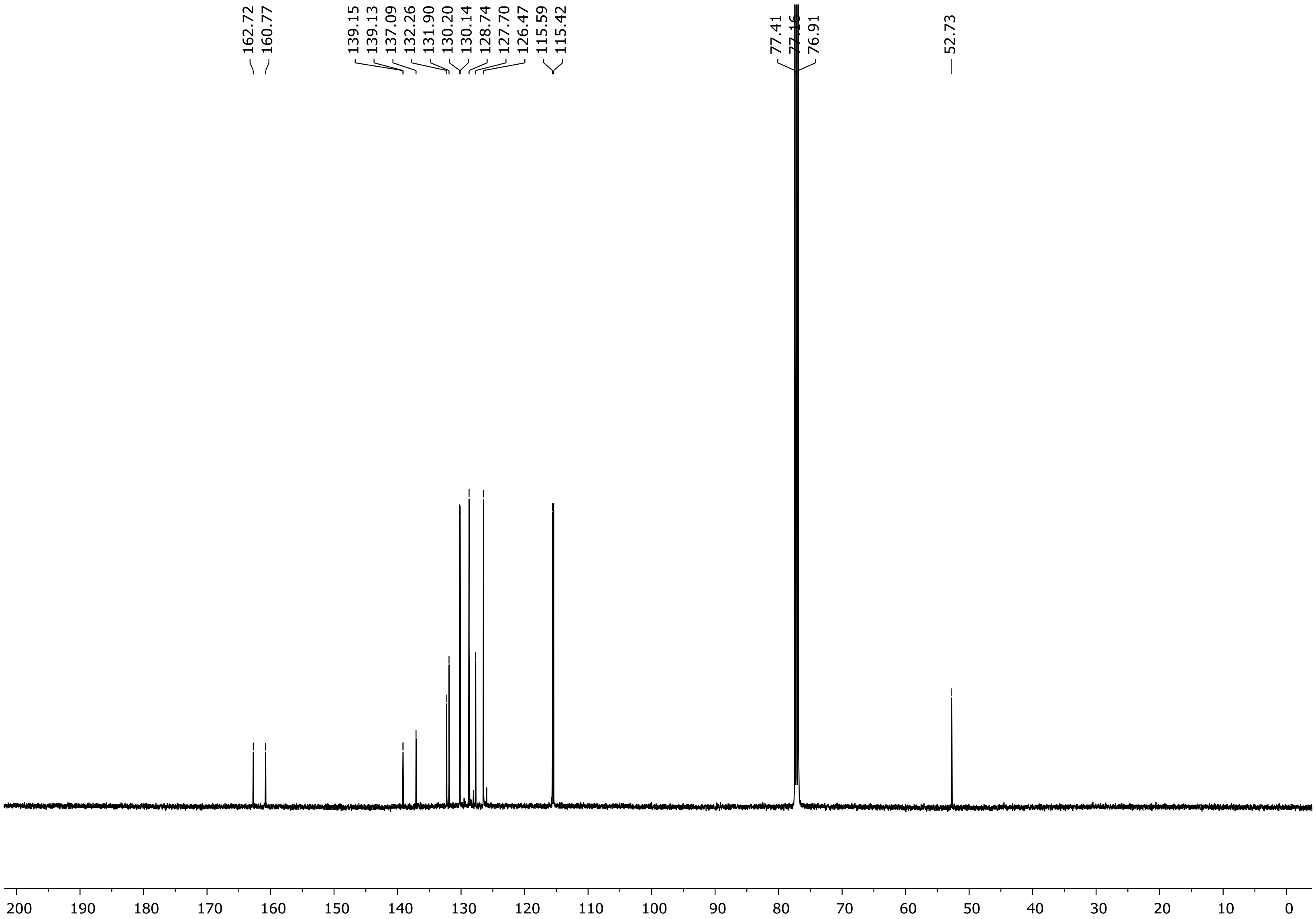

Figure S150: **^{19}F NMR** (376 MHz, $CDCl_3$, 298 K) spectrum of **3a**.

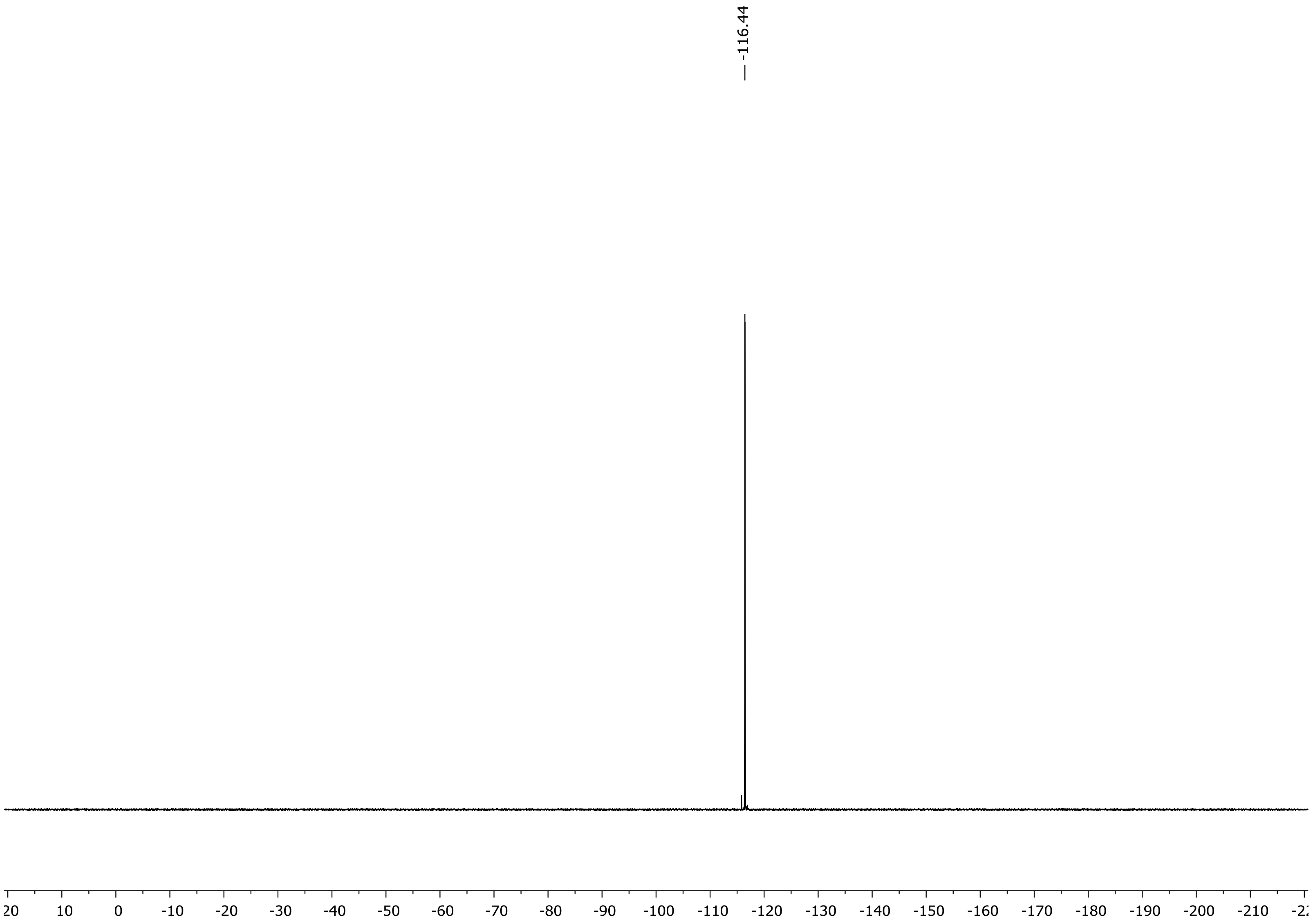

Figure S151: **^{1}H NMR** (500 MHz, $CDCl_3$, 298K) spectrum of **3b**.

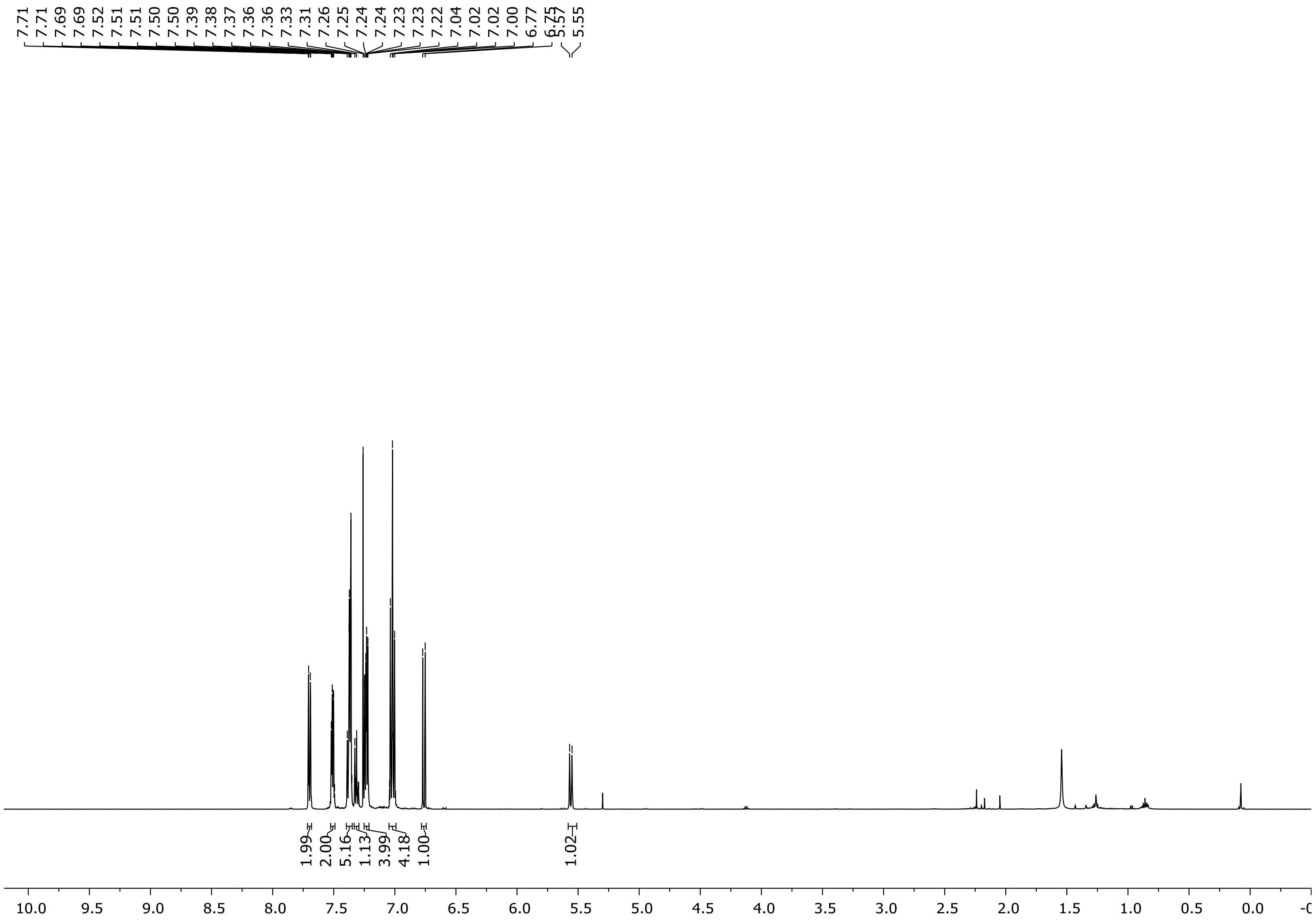

Figure S152: **^{13}C NMR** (126 MHz, $CDCl_3$, 298K) spectrum of **3b**.

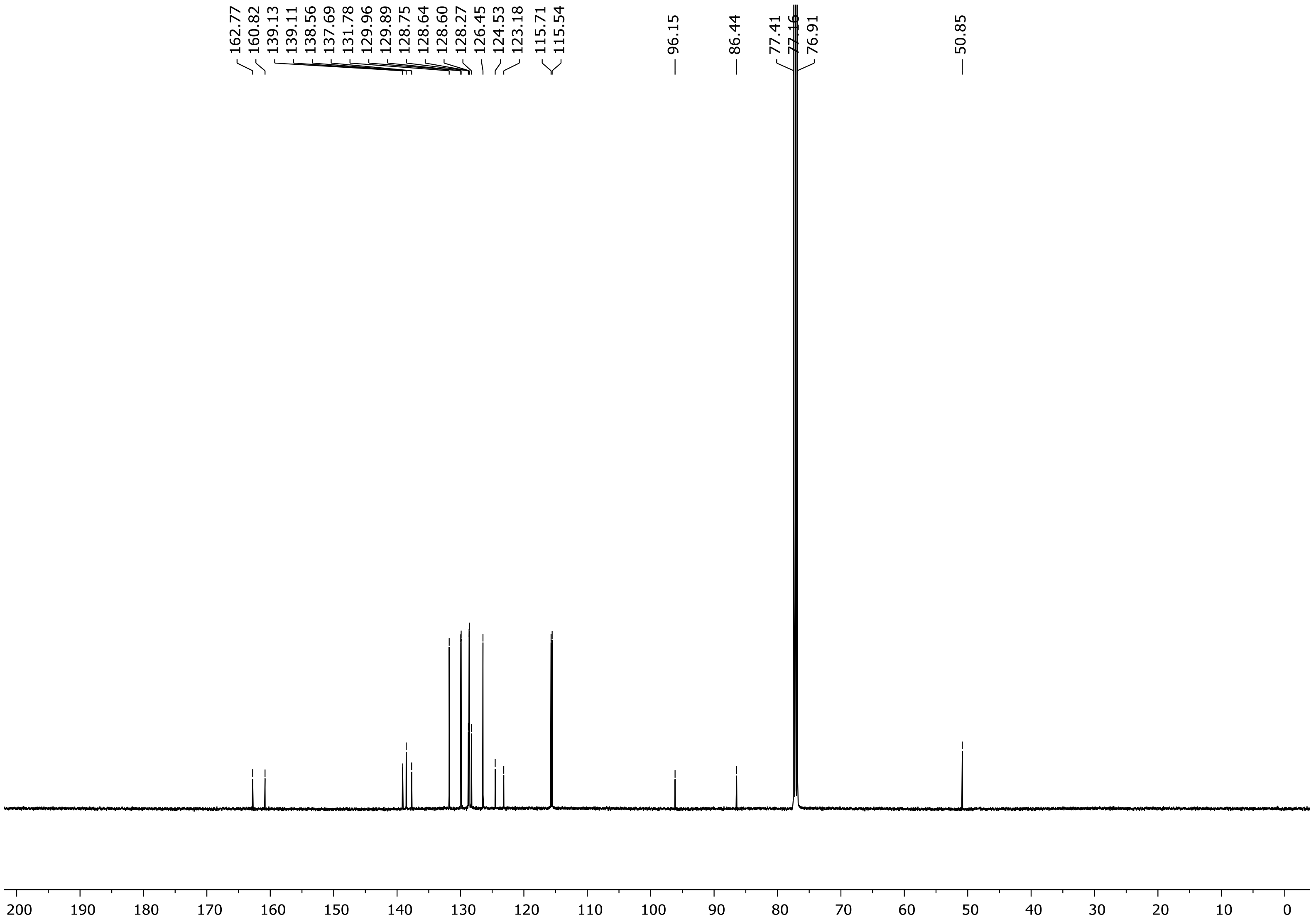

Figure S153: ^{19}**F NMR** (376 MHz, $CDCl_3$, 298 K) spectrum of **3b**.

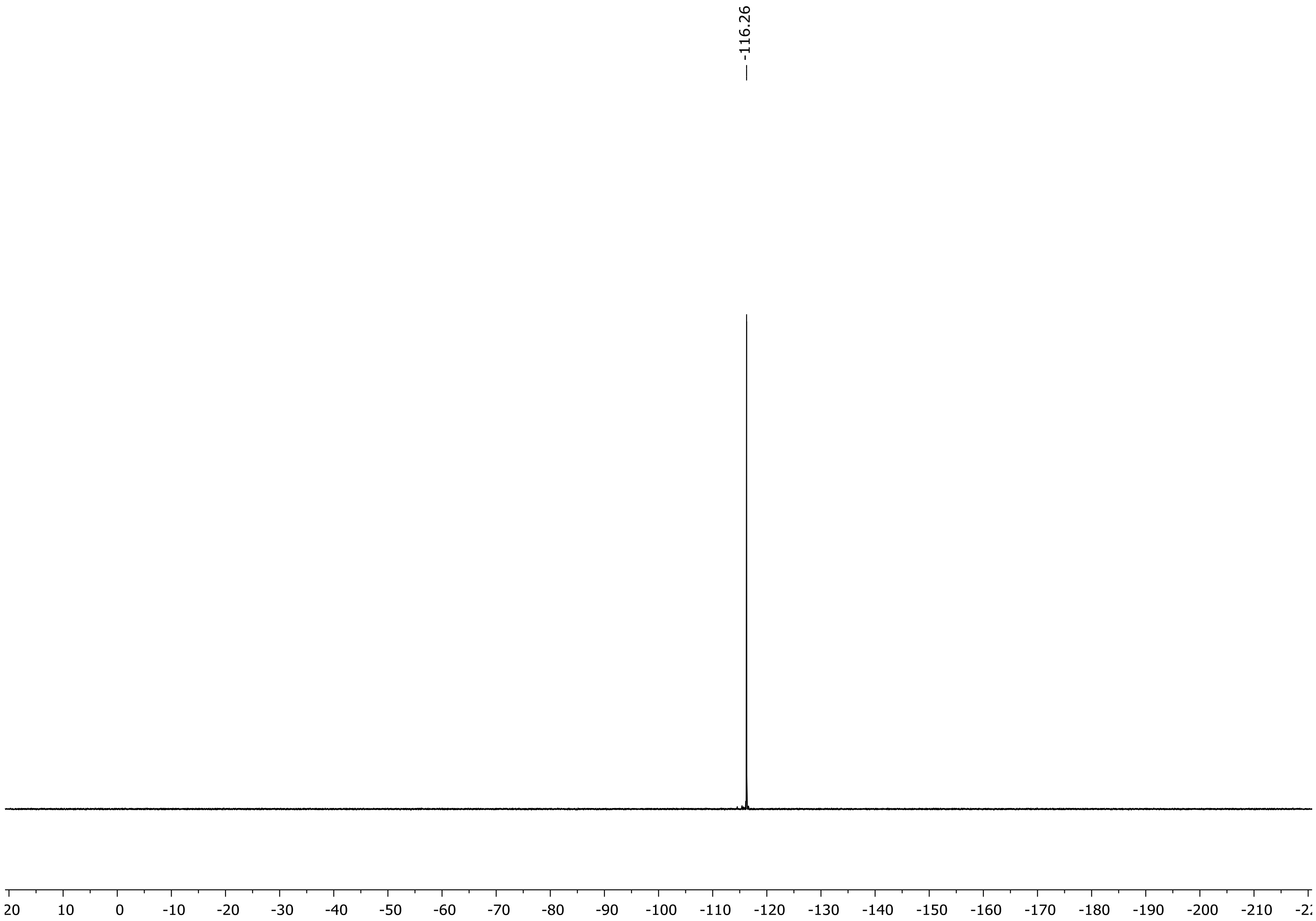

Figure S154: ^{1}**H NMR** (500 MHz, $CDCl_3$, 298K) spectrum of **3c**.

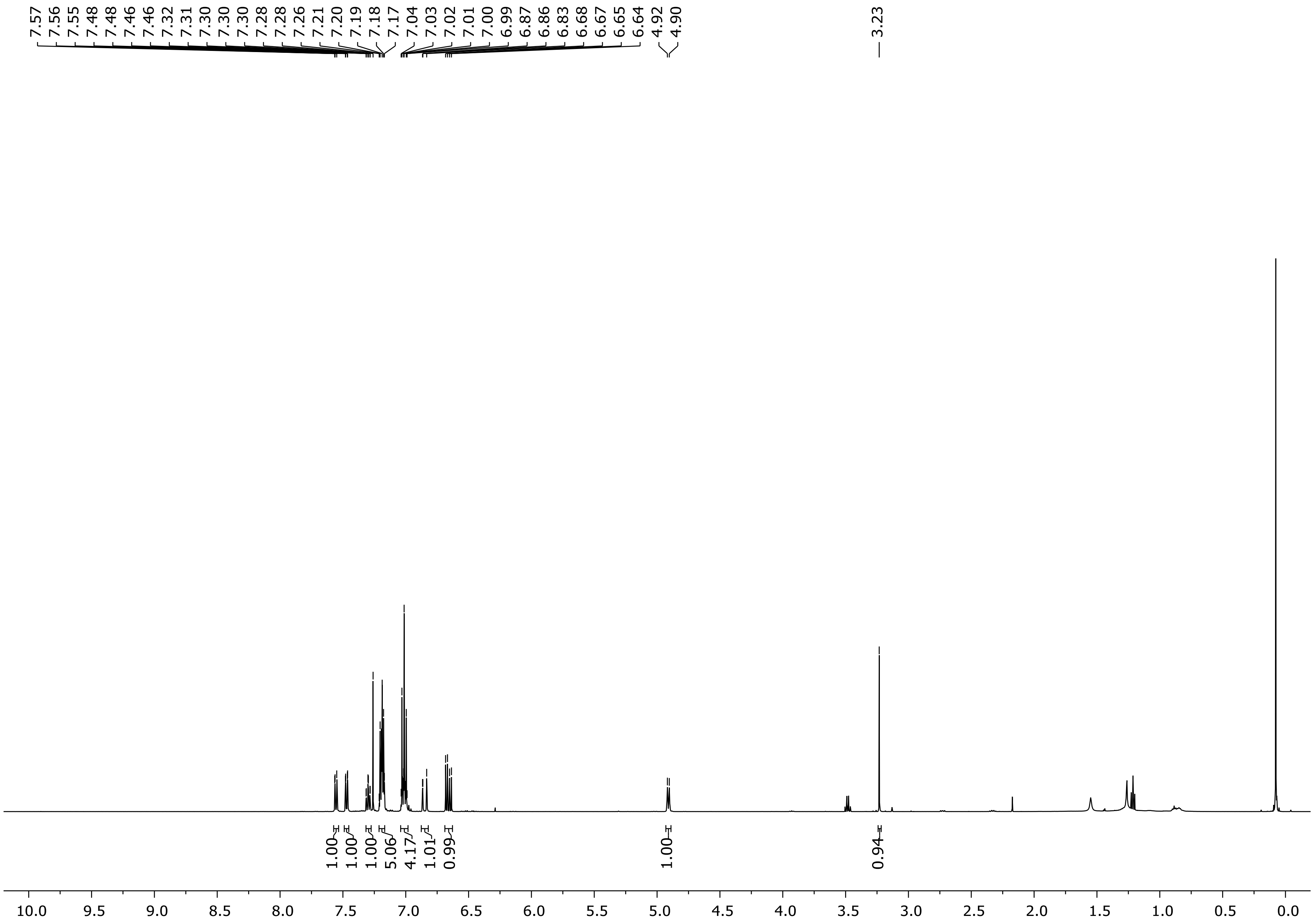

Figure S155: **^{13}C NMR** (126 MHz, $CDCl_3$, 298K) spectrum of **3c**.

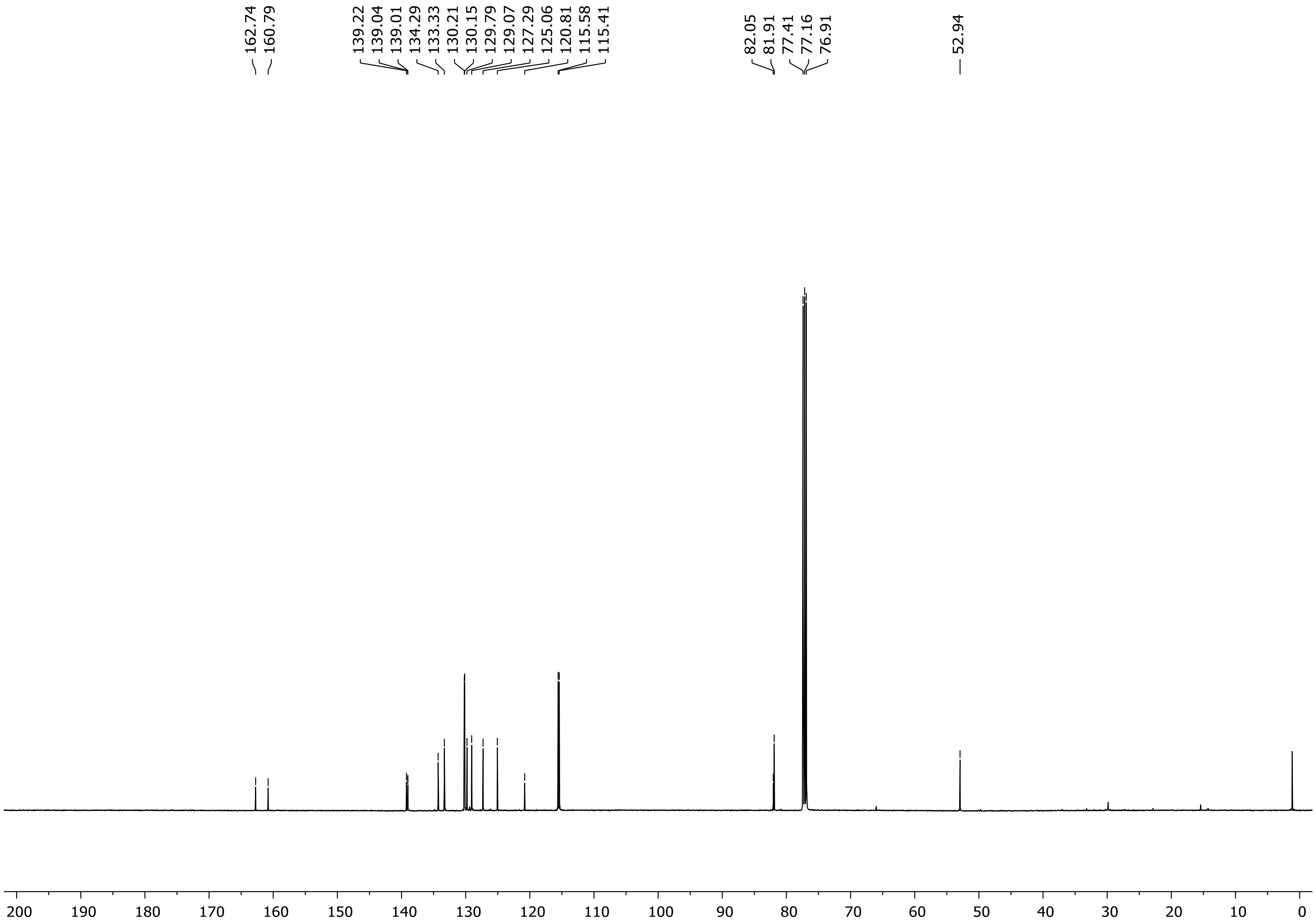

Figure S156: **^{19}F NMR** (471 MHz, $CDCl_3$, 298K) spectrum of **3c**.

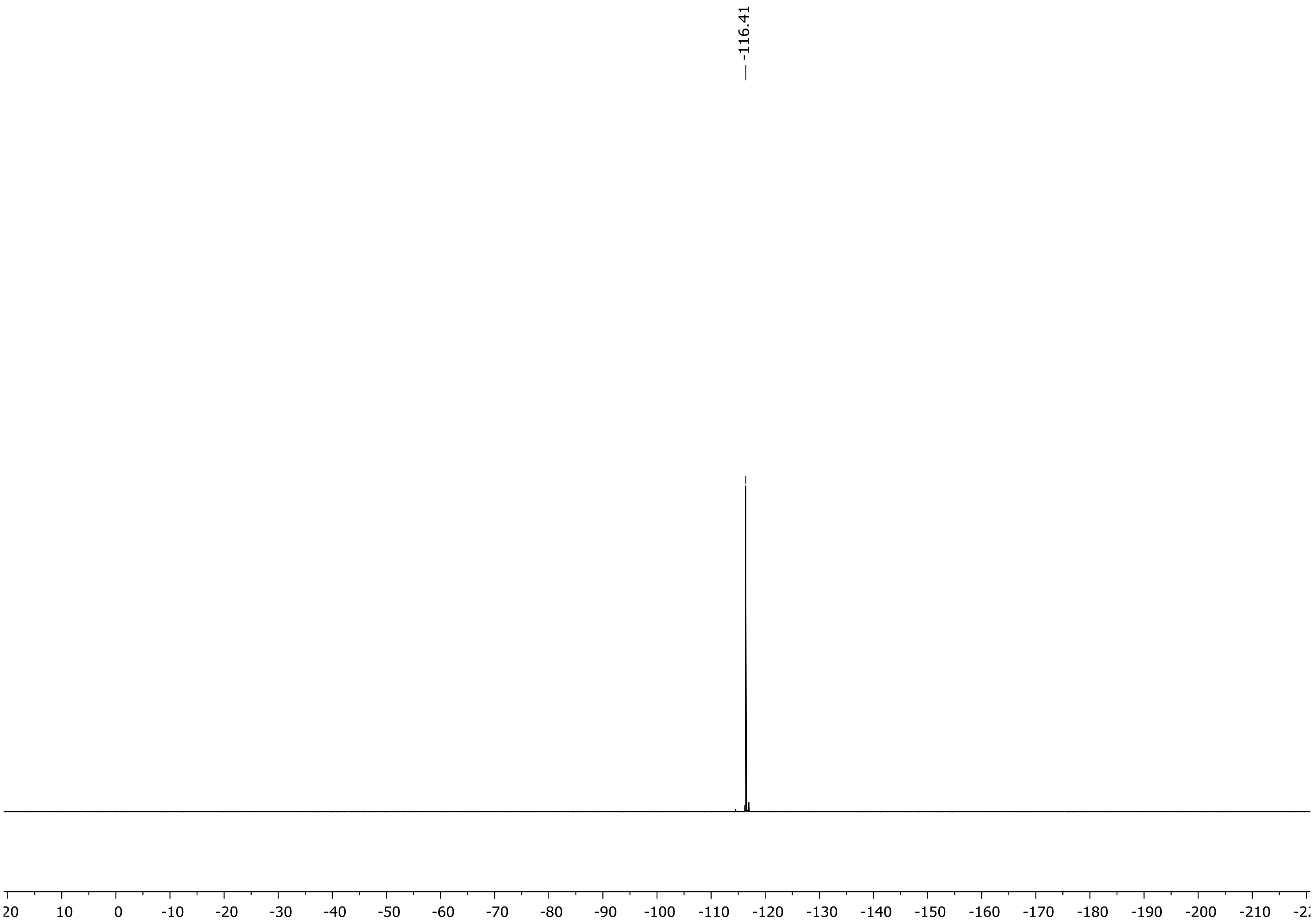

Figure S157: **^{1}H NMR** (500 MHz, $CDCl_3$, 298K) spectrum of **3d**.

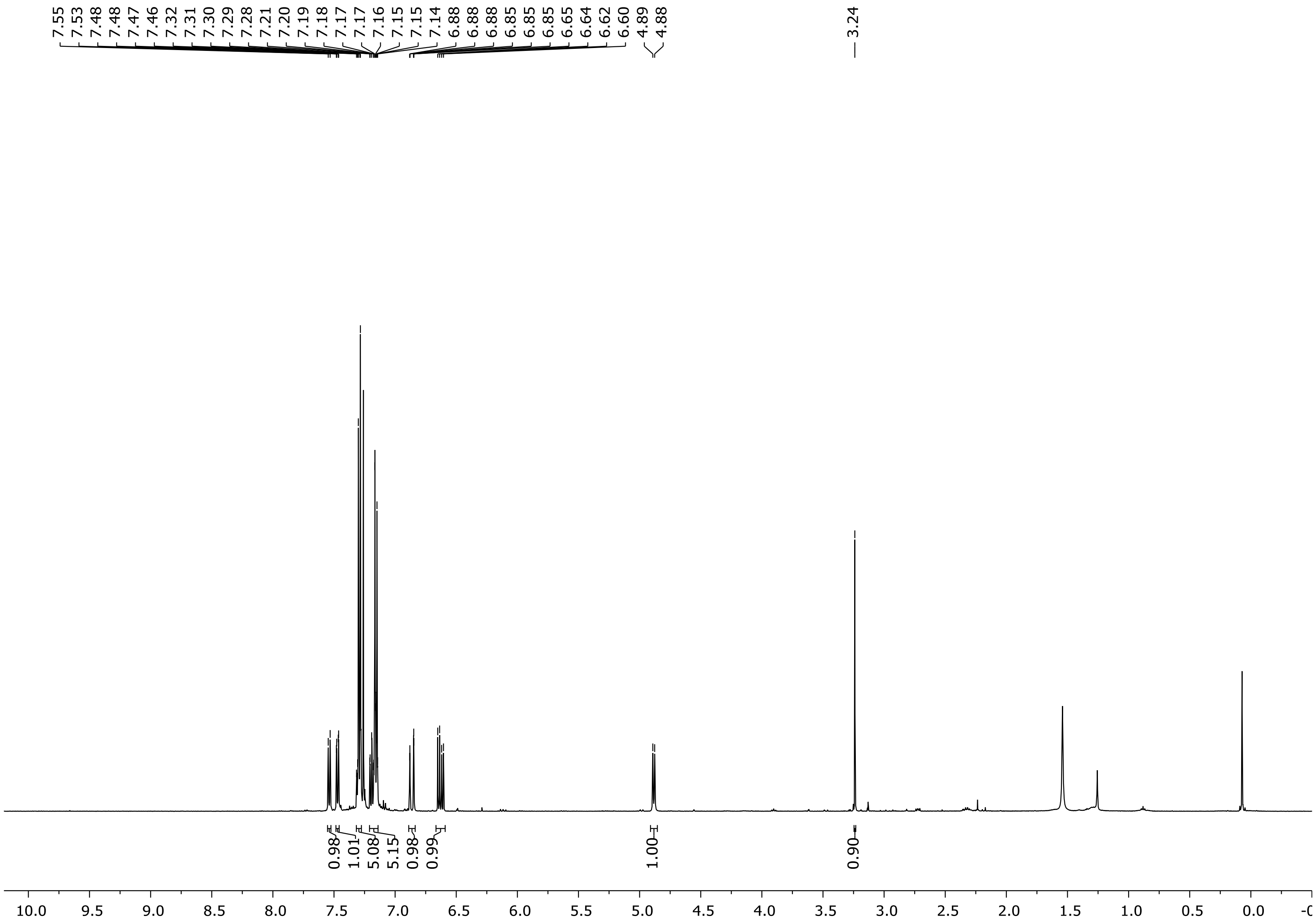

Figure S158: **^{13}C NMR** (126 MHz, $CDCl_3$, 298K) spectrum of **3d**.

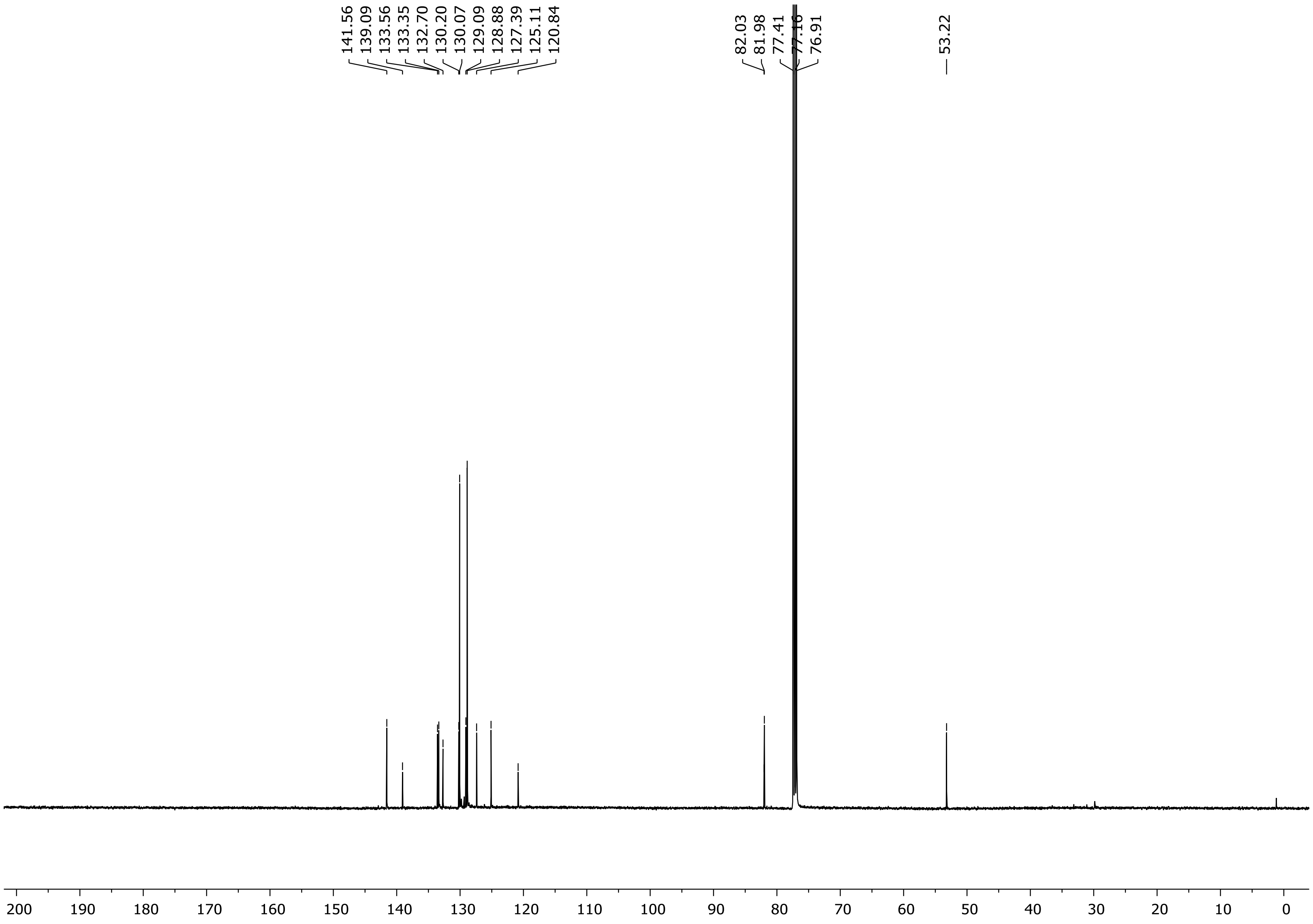

Figure S159: **^{1}H NMR** (500 MHz, $CDCl_3$, 298K) spectrum of **3e**.

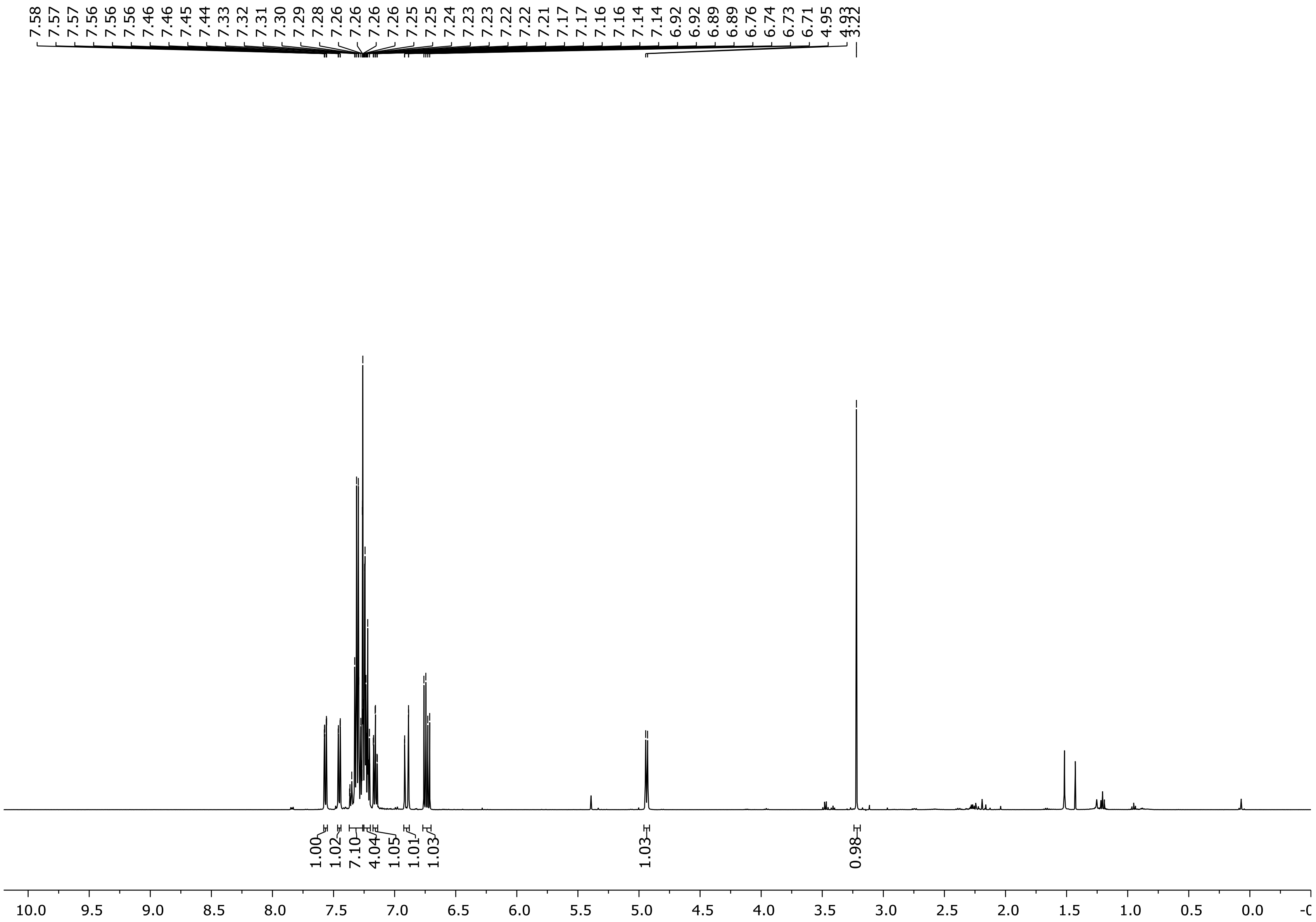

Figure S160: **^{13}C NMR** (126 MHz, $CDCl_3$, 298K) spectrum of **3e**.

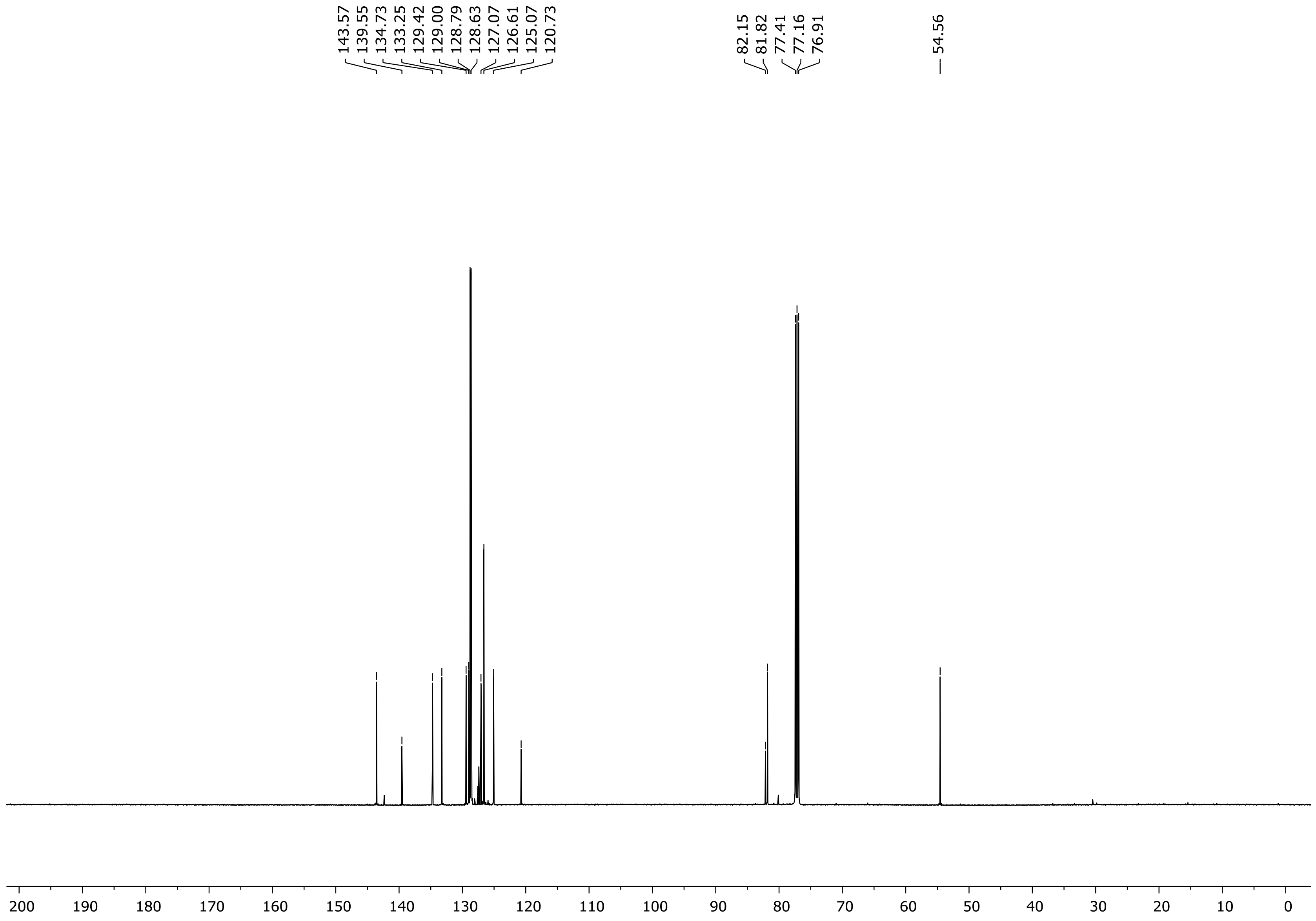

Figure S161: **^{1}H NMR** (500 MHz, $CDCl_3$, 298K) spectrum of **3f**.

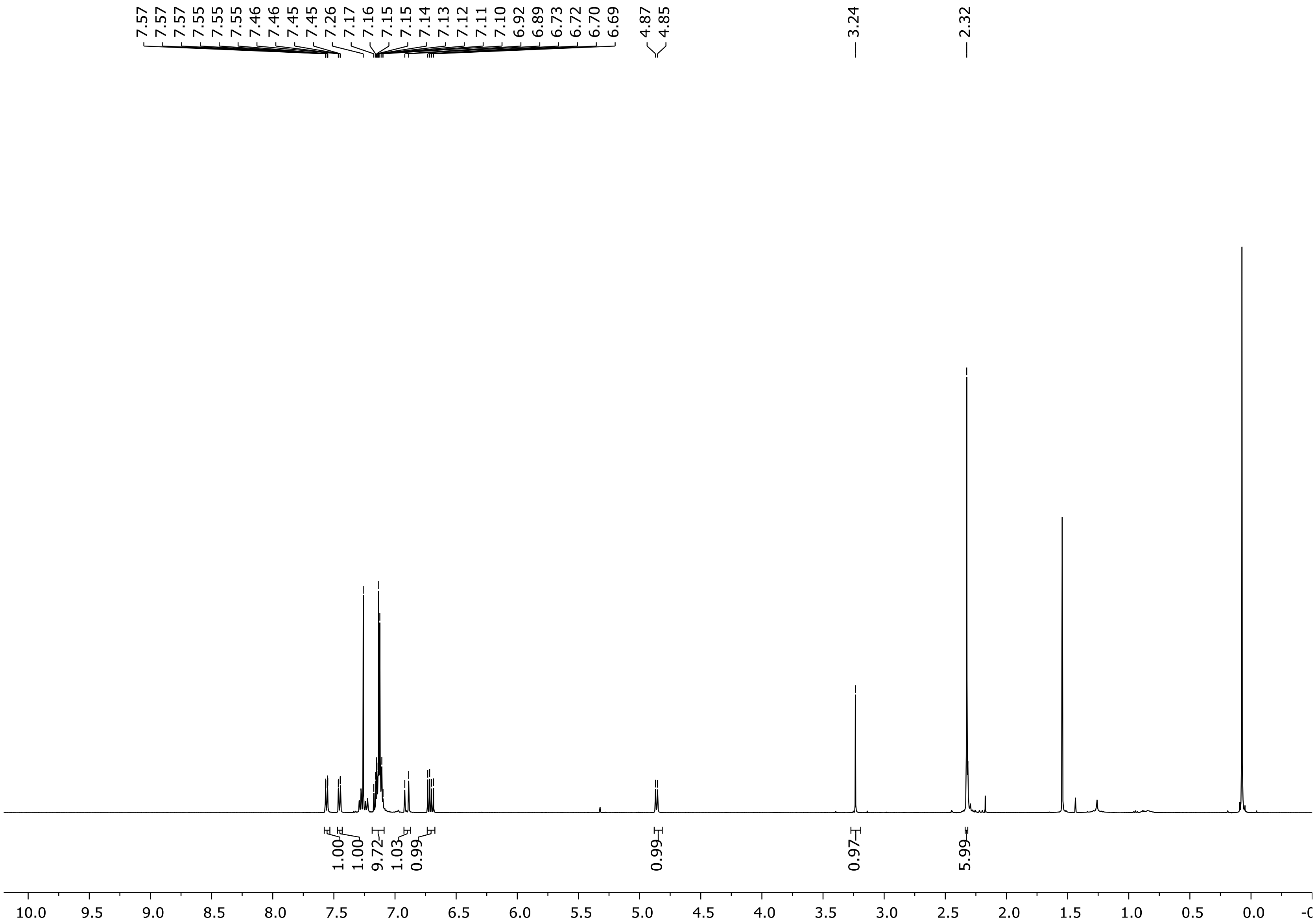

Figure S162: ^{13}**C NMR** (126 MHz, $CDCl_3$, 298K) spectrum of **3f**.

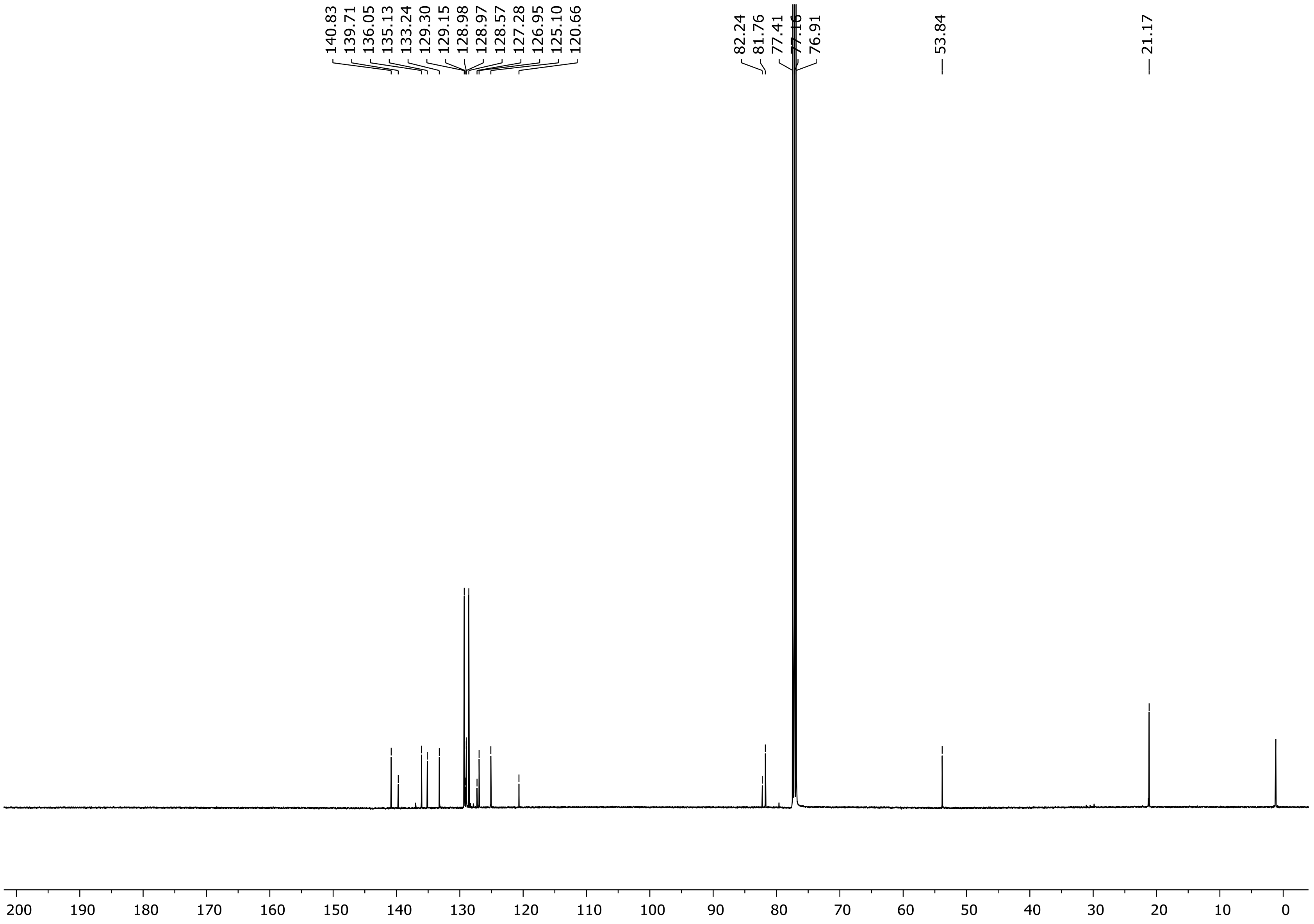

Figure S163: **^{1}H NMR** (500 MHz, $CDCl_3$, 298K) spectrum of **3h**.

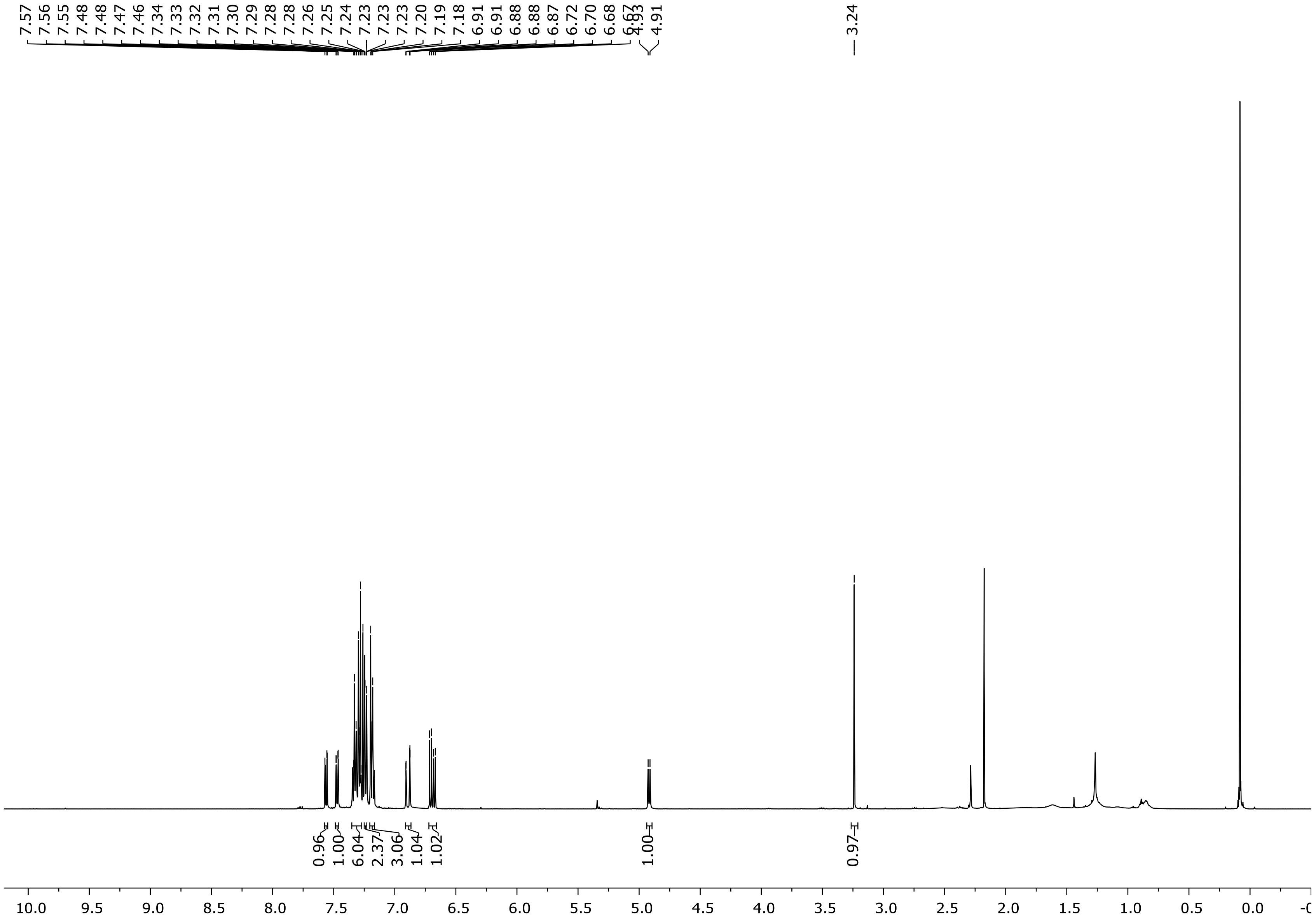

Figure S164: **^{13}C NMR** (126 MHz, $CDCl_3$, 298K) spectrum of **3h**.

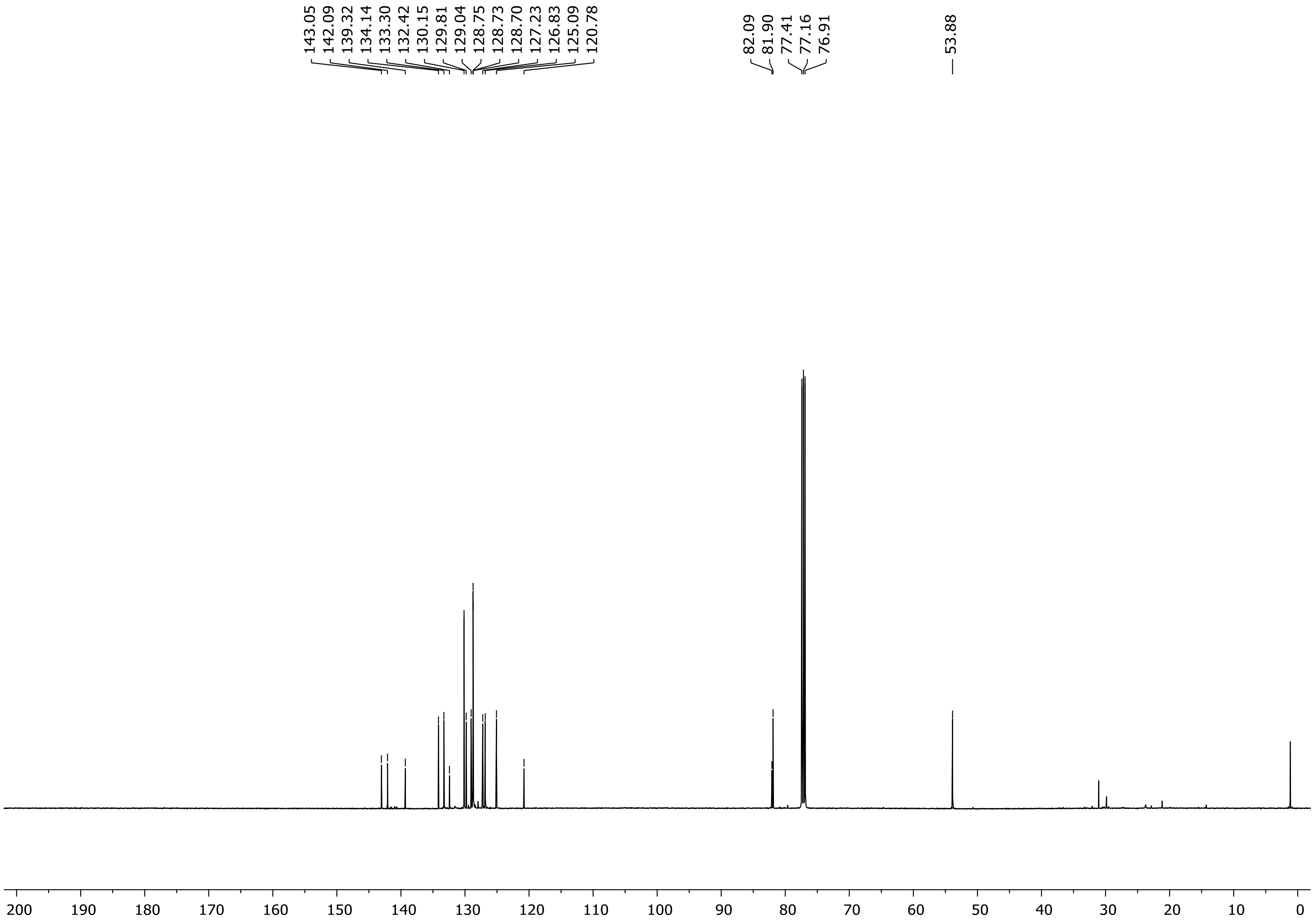

Figure S165: **^{1}H NMR** (500 MHz, $CDCl_3$, 298K) spectrum of **3i**.

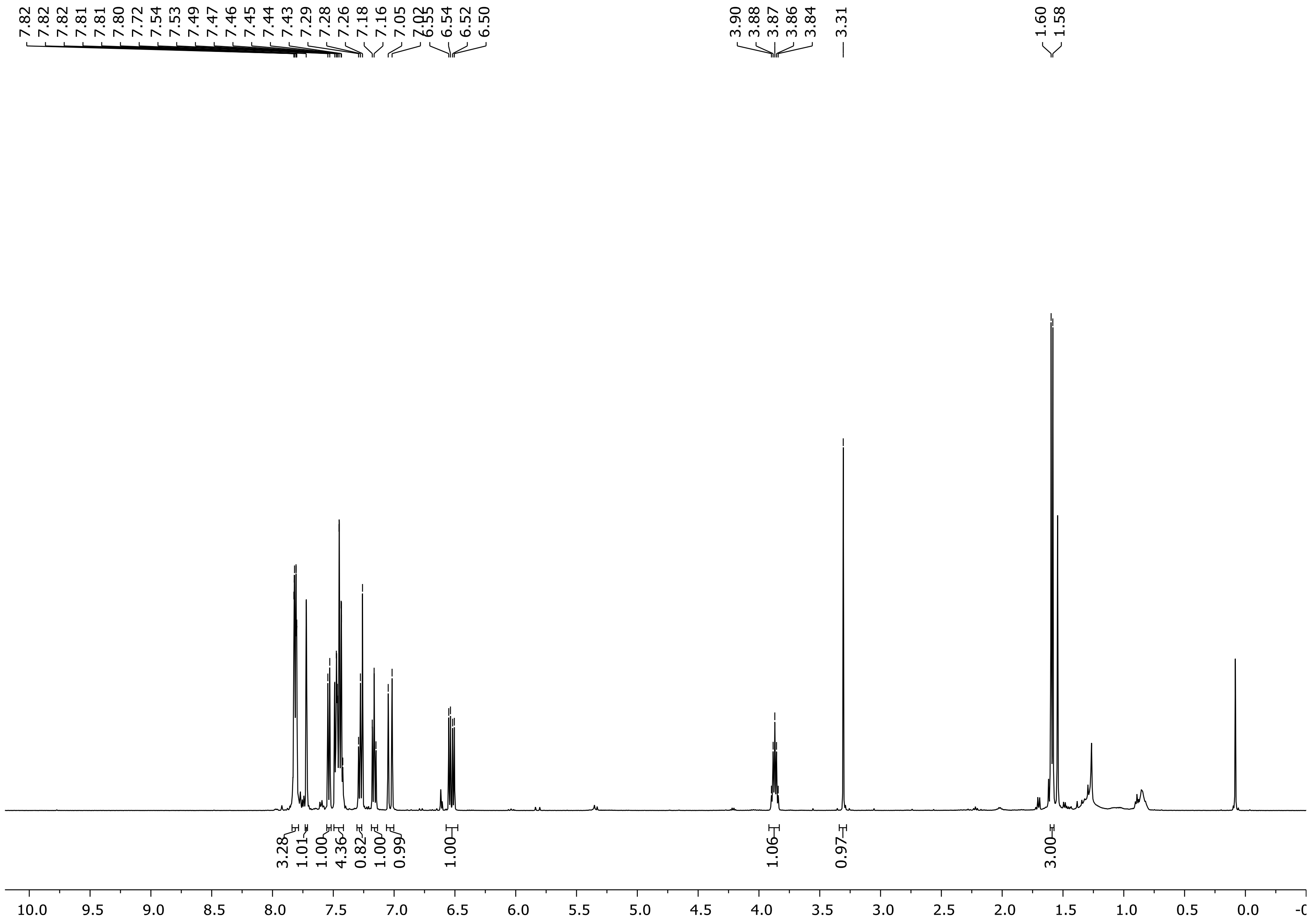

Figure S166: **^{13}C NMR** (126 MHz, $CDCl_3$, 298K) spectrum of **3i**.

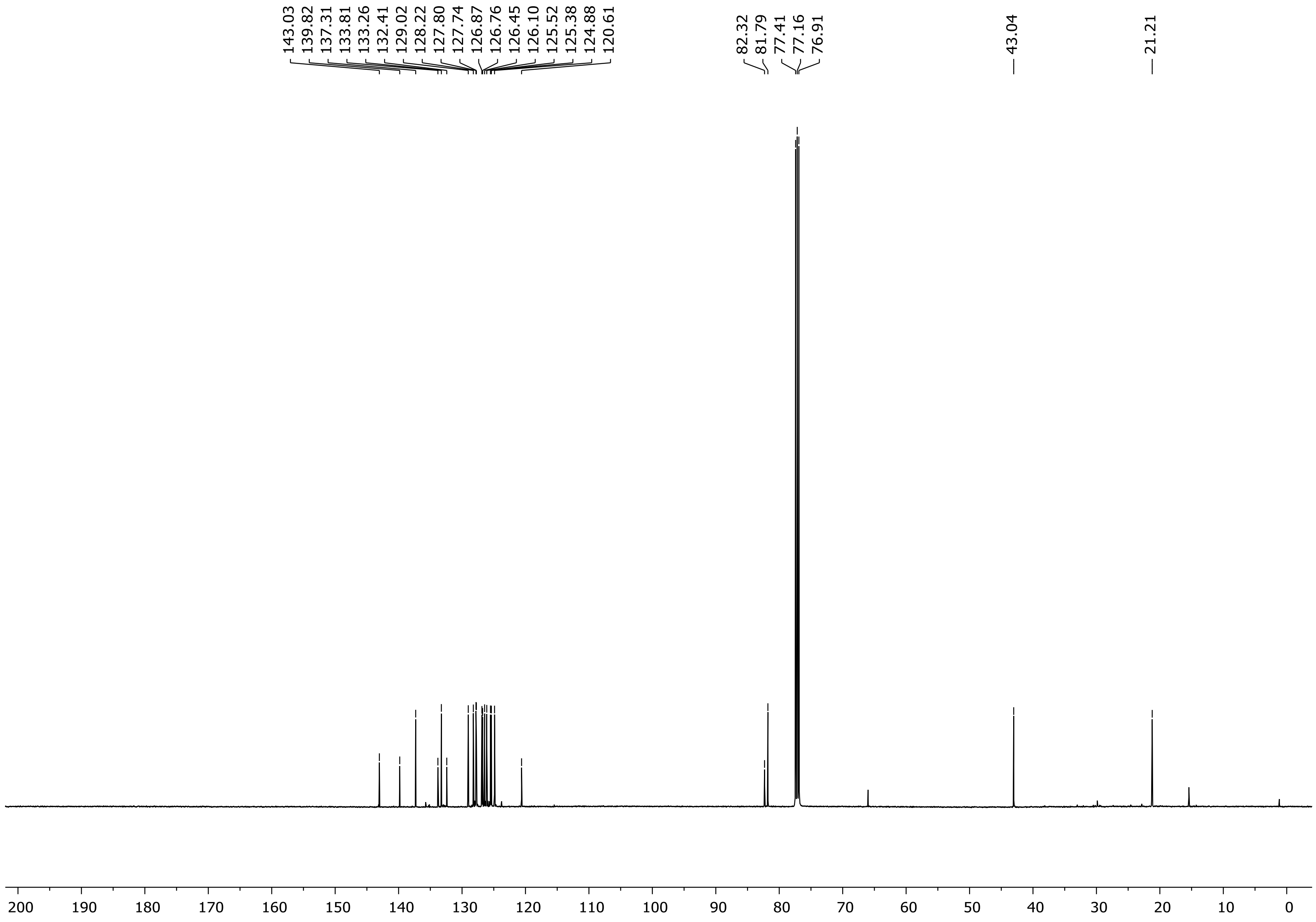

Figure S167: **^{1}H NMR** (500 MHz, $CDCl_3$, 298K) spectrum of **3j**.

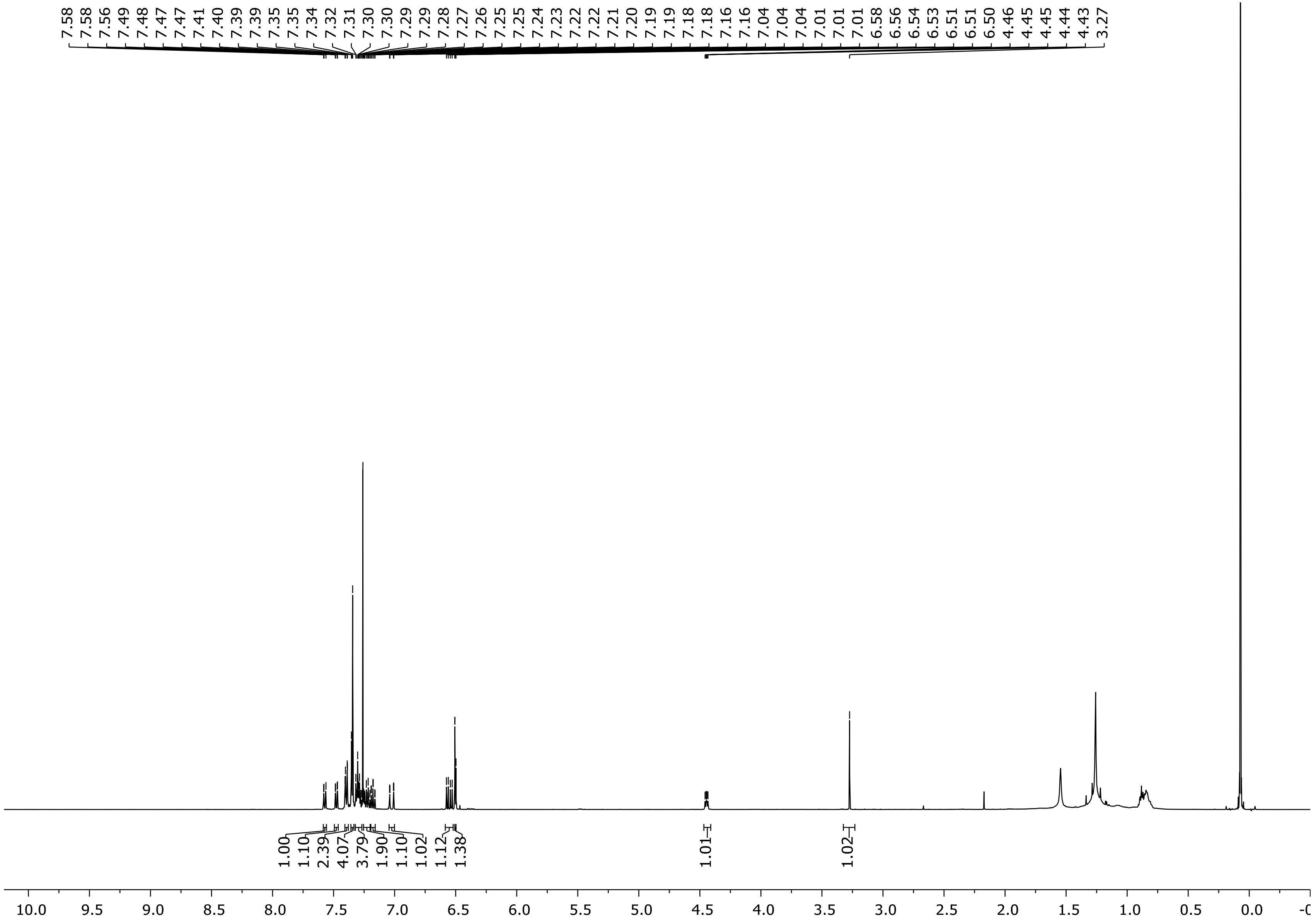

Figure S168: **^{13}C NMR** (126 MHz, $CDCl_3$, 298K) spectrum of **3j**.

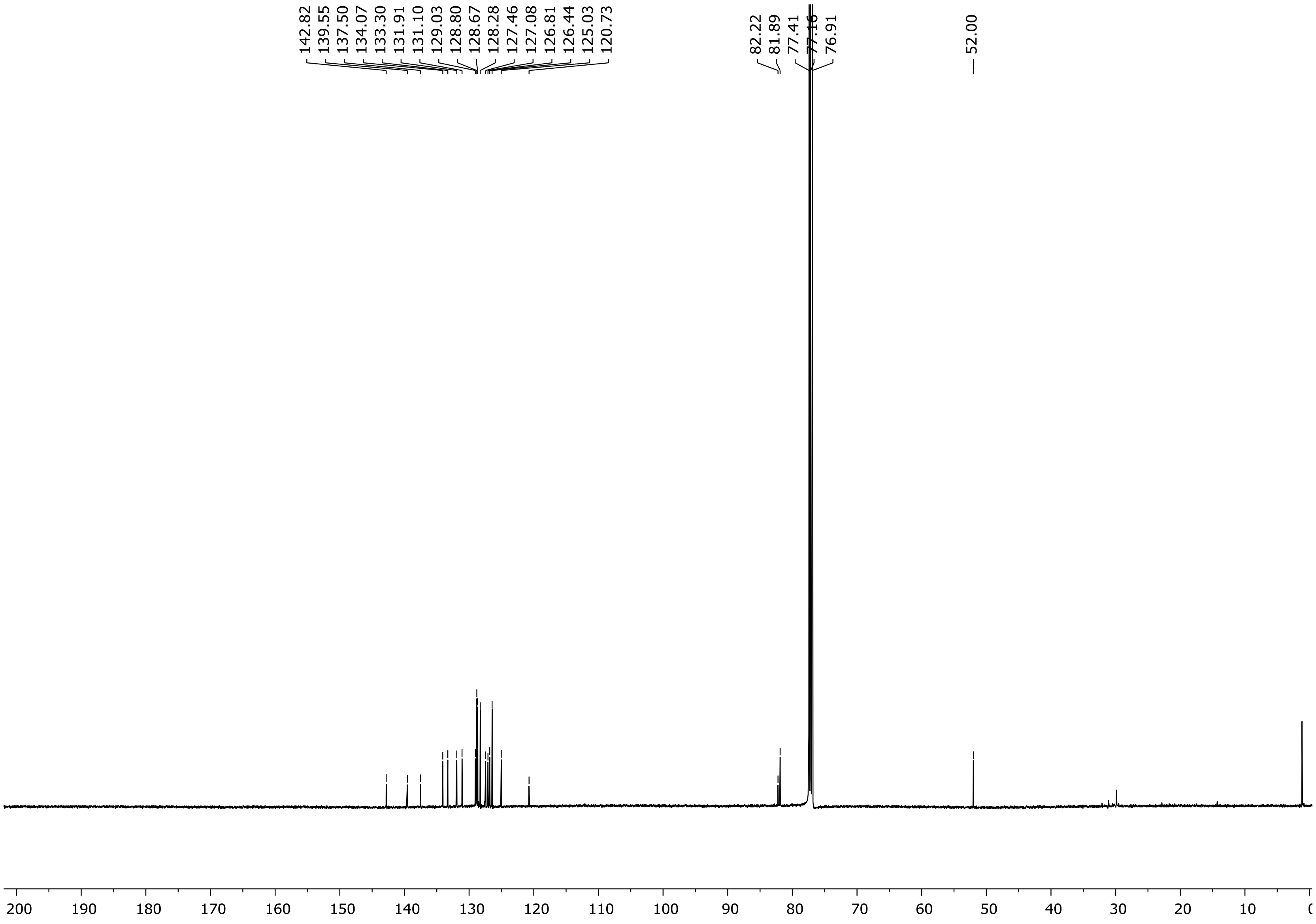

Figure S169: **¹H NMR** (500 MHz, $CDCl_3$, 298K) spectrum of **5d**.

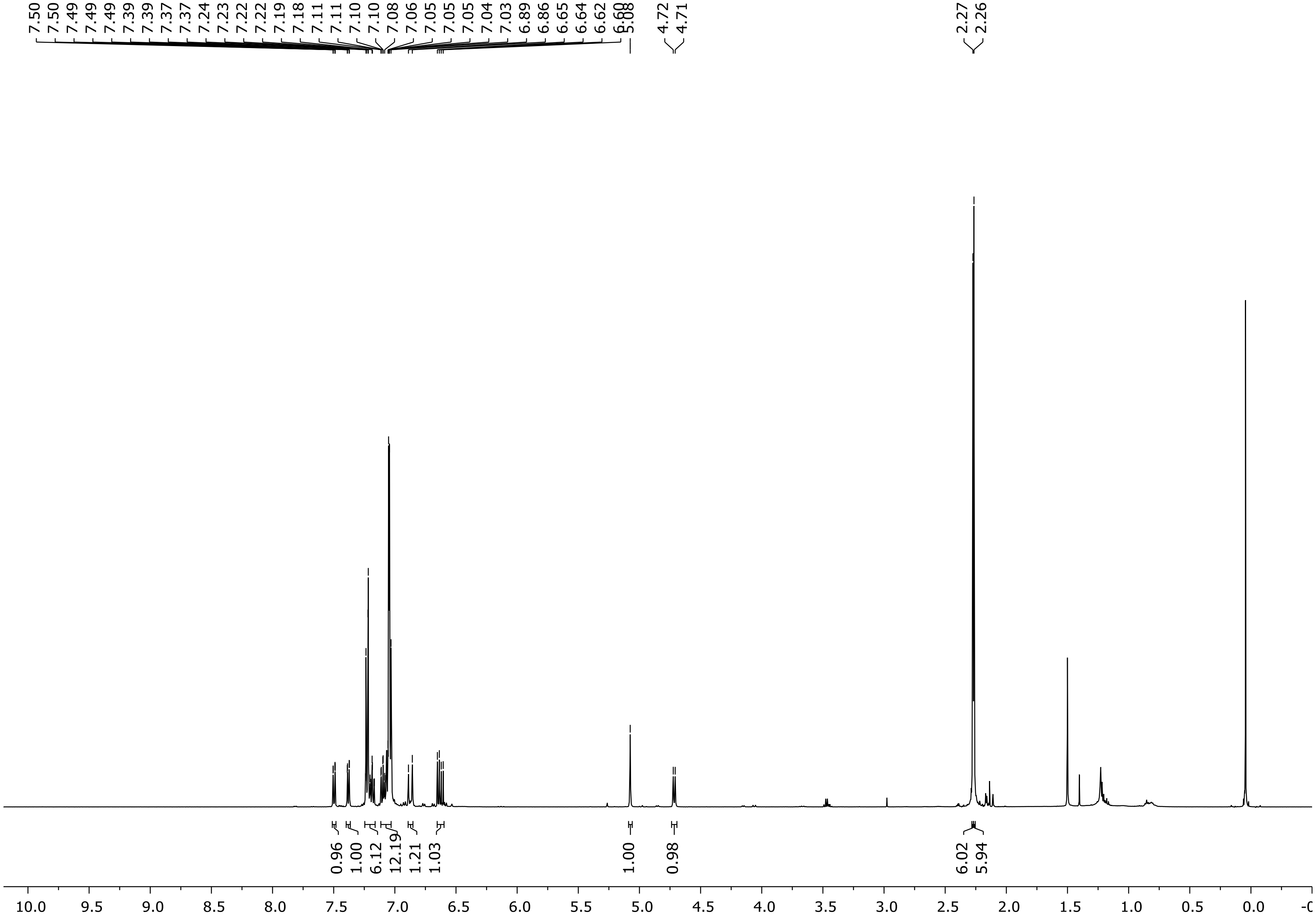

Figure S170: **^{13}C NMR** (126 MHz, $CDCl_3$, 298K) spectrum of **5d**.

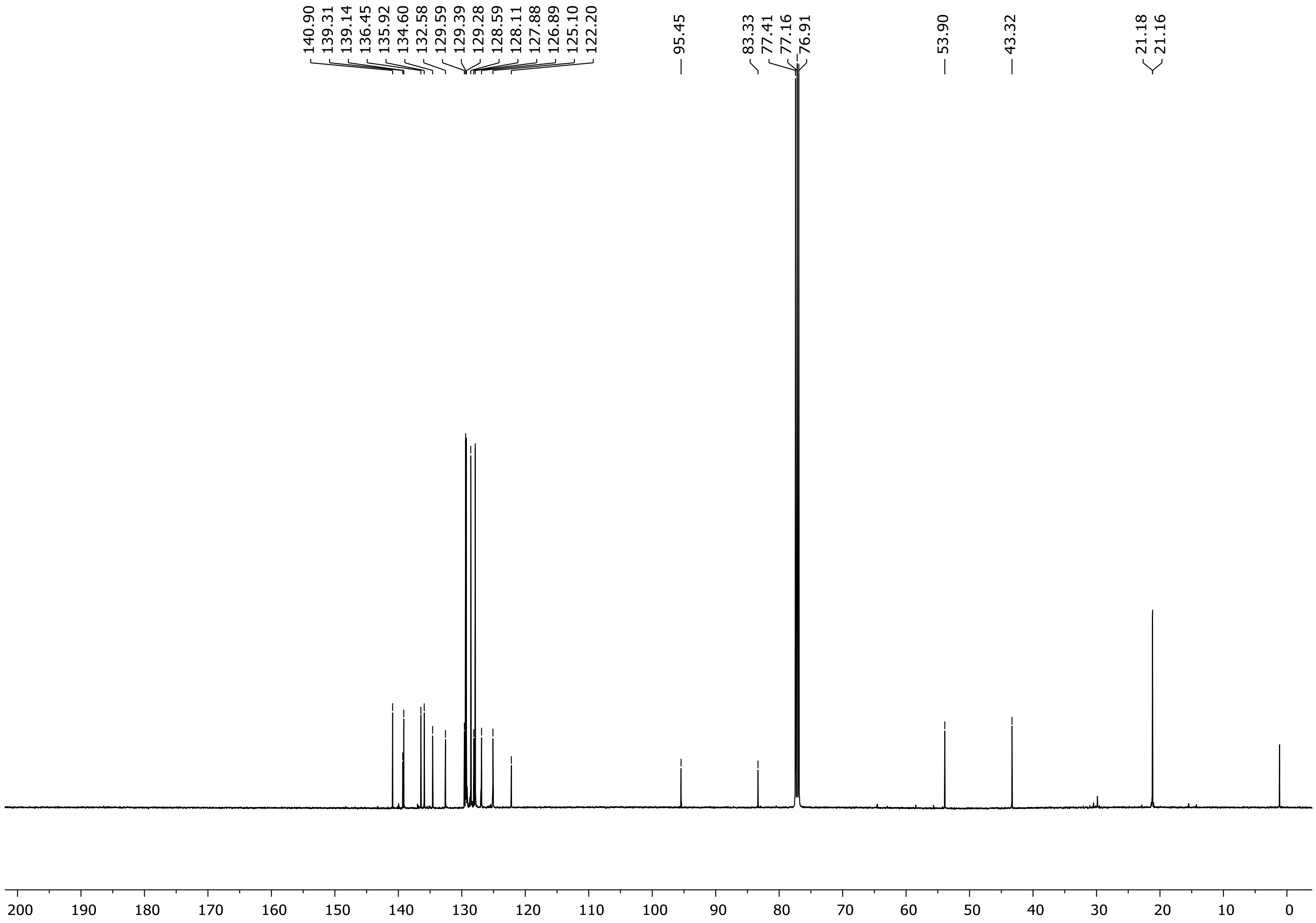

Figure S171: **^{1}H NMR** (500 MHz, $CDCl_3$, 298K) spectrum of **5g**.

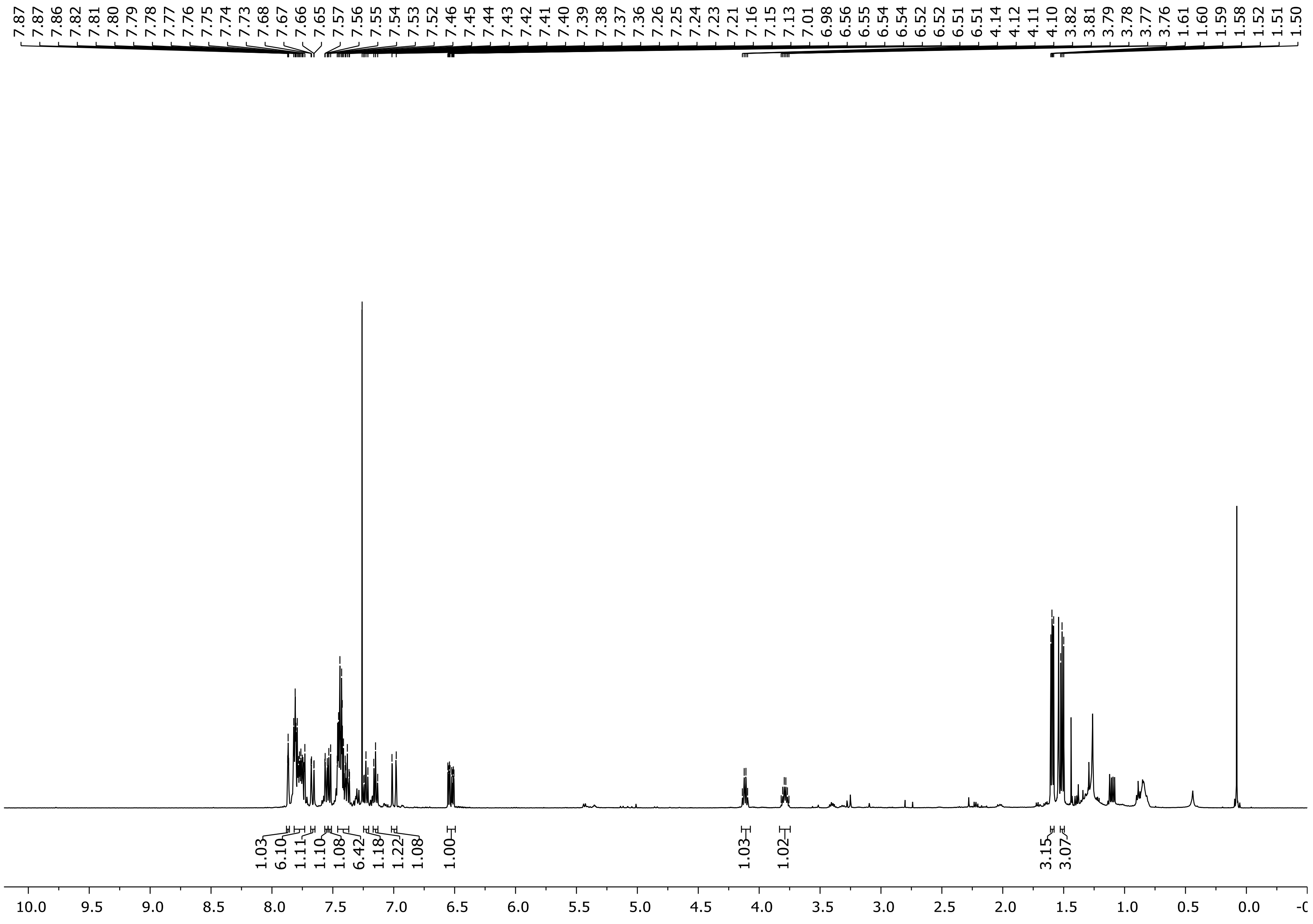

Figure S172: ^{13}**C NMR** (126 MHz, $CDCl_3$, 298K) spectrum of **5g**.

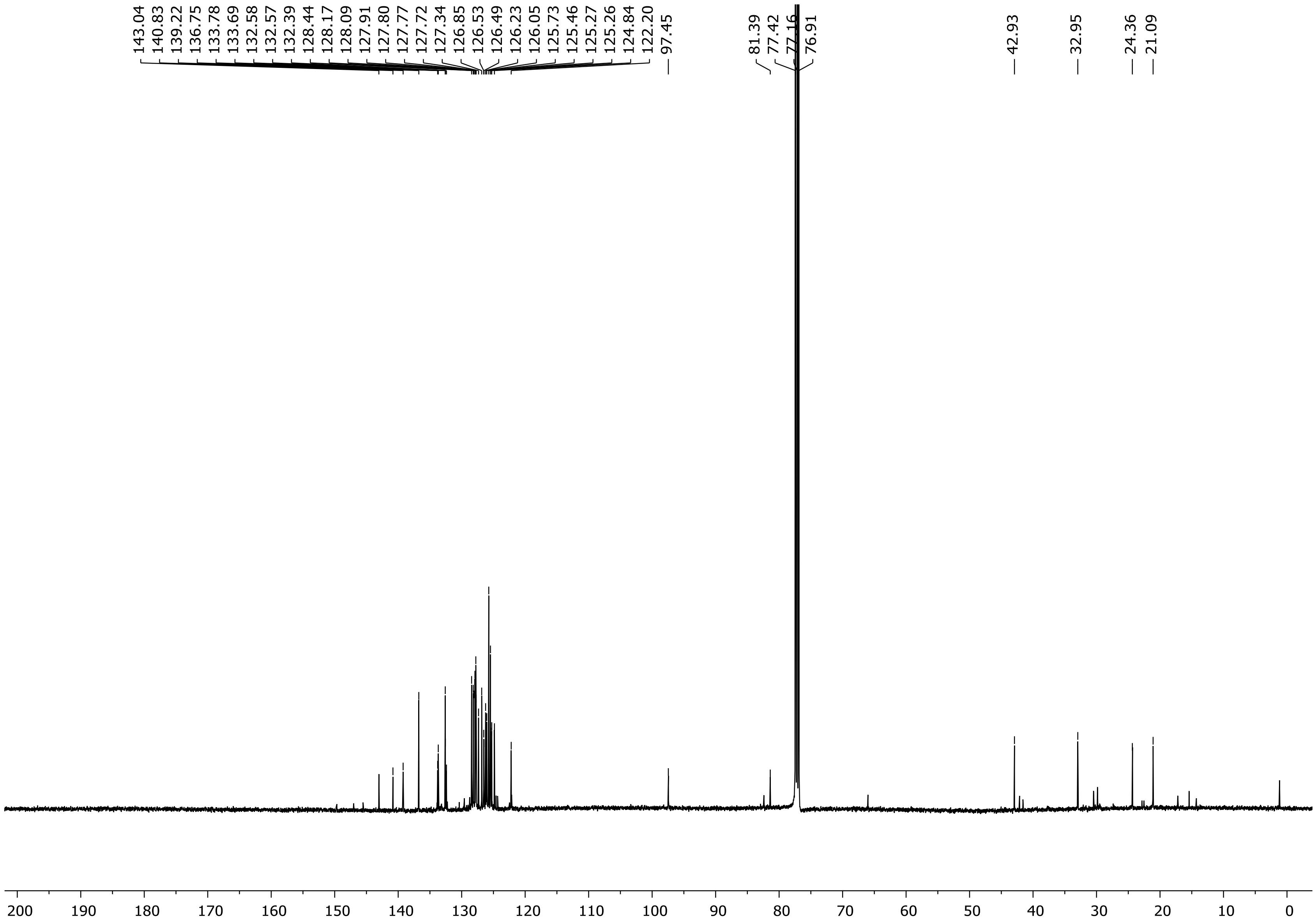

Figure S173: **^{1}H NMR** (500 MHz, $CDCl_3$, 298K) spectrum of **5h**.

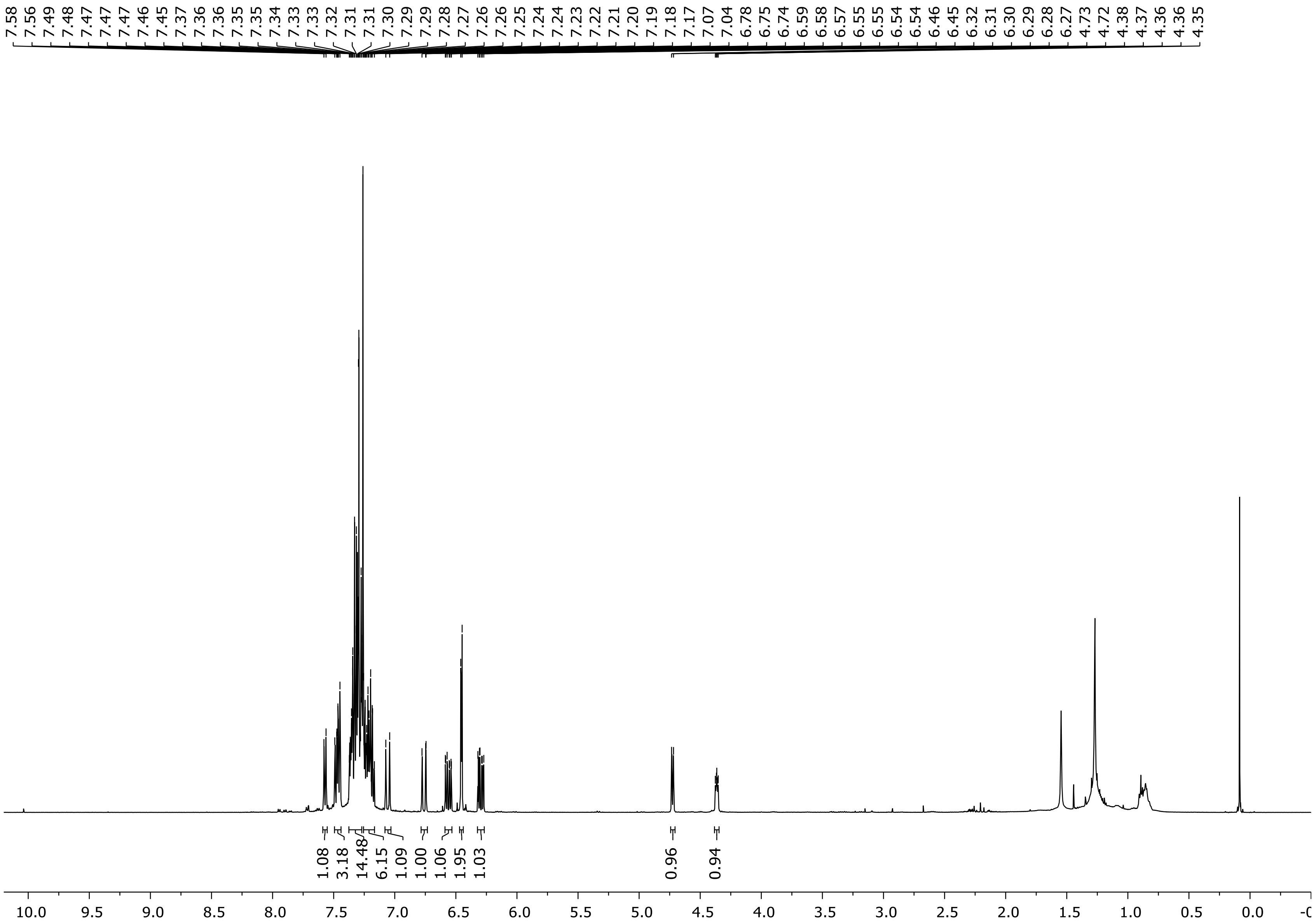

Figure S174: **^{13}C NMR** (126 MHz, $CDCl_3$, 298K) spectrum of **5h**.

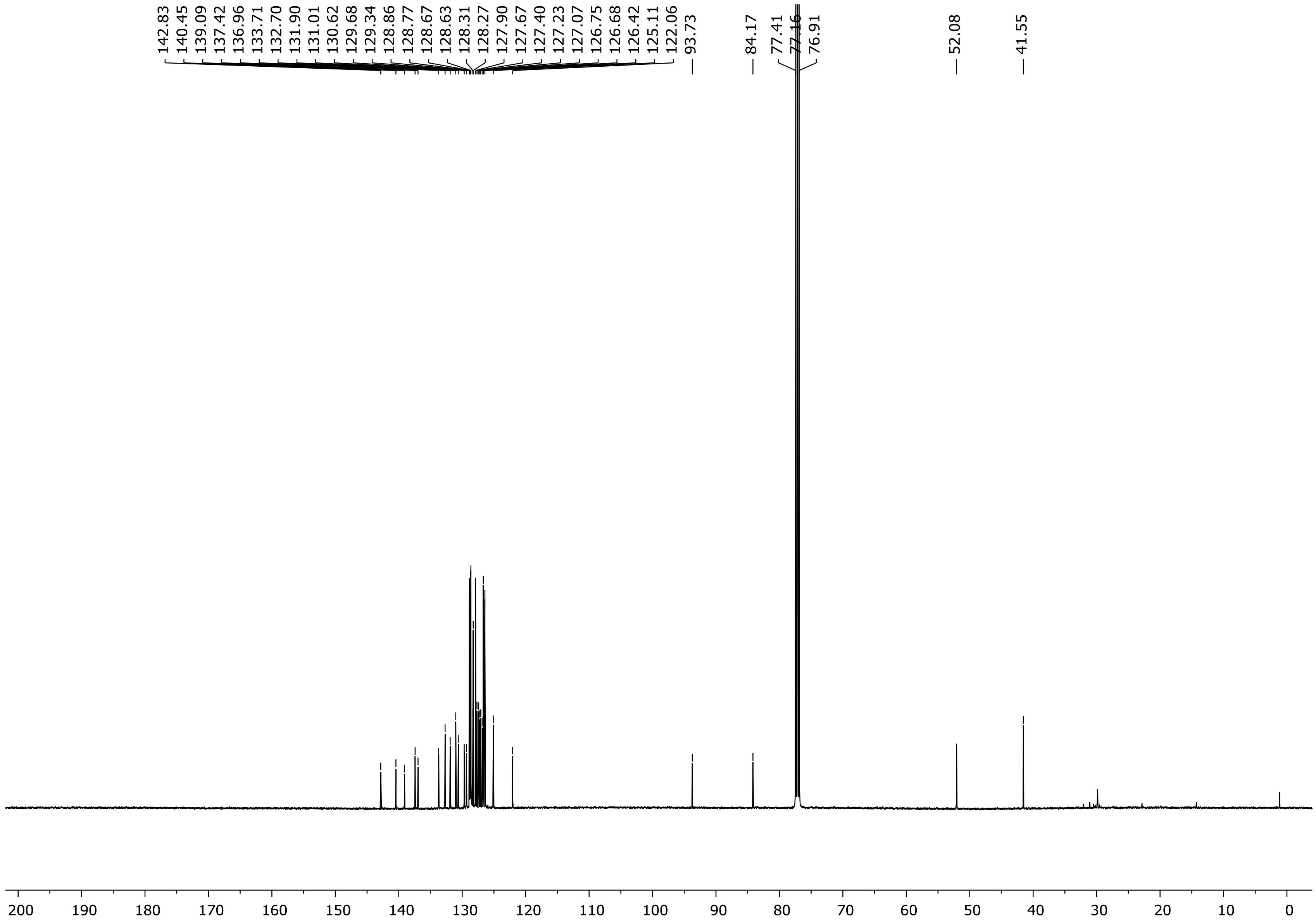

4. Competition Experiments:

Figure S175: **^{19}F NMR** (500 MHz, $CDCl_3$, 298K, without proton decoupled).

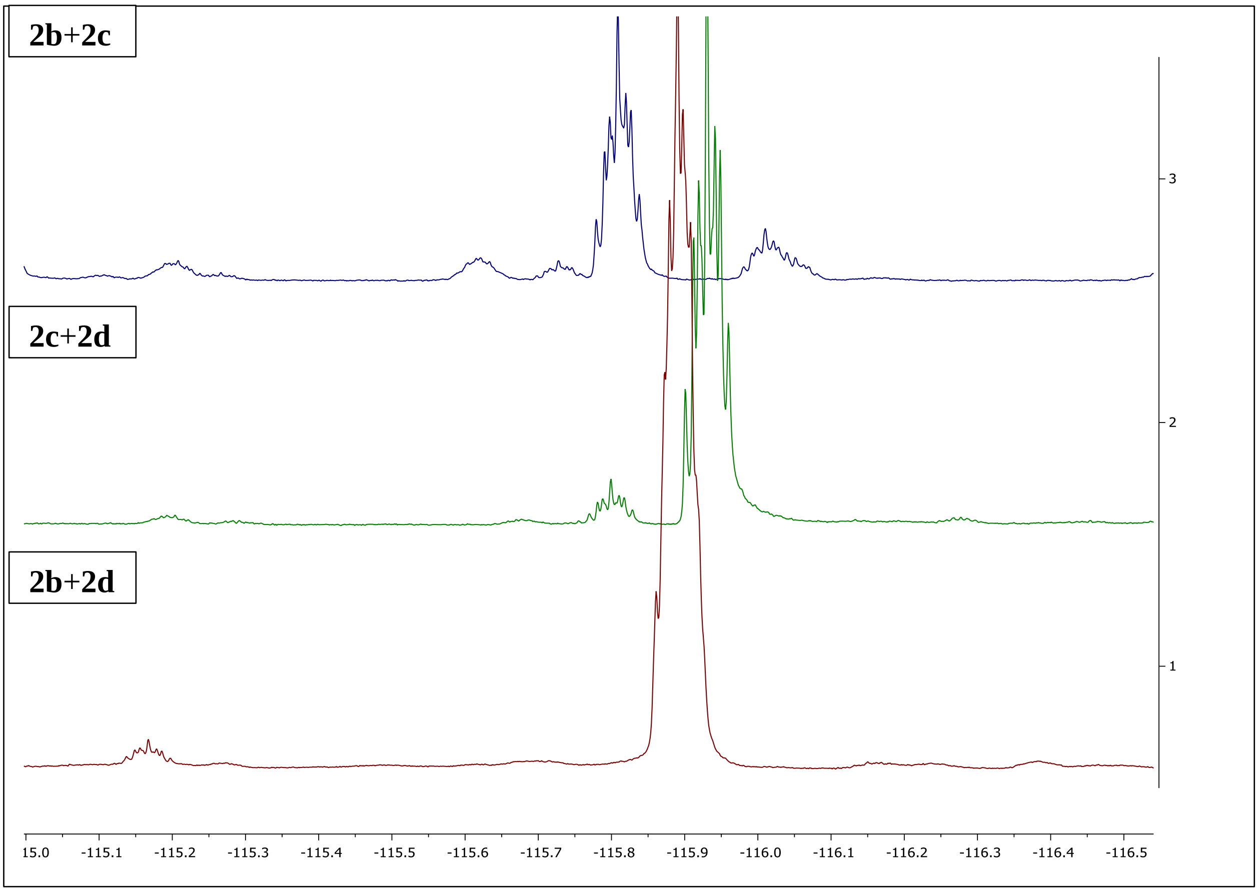

5. EPR Studies

The X-band CW EPR measurements were performed on a Bruker EMX spectrometer utilizing an ER4119HS resonator, 100 kHz field modulation and optimized field modulation depths (see Figure captions for details). Spectra were recorded at variable temperature, utilizing an ER4131VT control unit and high-temperature heating accessories. All simulations were performed using the garlic or pepper core functions within the Easyspin toolbox for Matlab,[18] which requires the principal values of the **g** and **A** tensors to be included. The garlic function describes fast-motional CW EPR spectra of radicals in solution, which results in inequivalent line broadening and thus different peak amplitudes of the resulting hyperfine lines originating from coupling to the ^{31}P (I = ½) nuclei.

In order to prove the mechanism of the FLP mediated reaction, in particular to distinguish the validity of a SET process, a series of EPR experiments were performed as detailed below. No EPR signal could be detected of a solution of the $Mes_3P:B(C_6F_5)_3$ FLP in the absence of any substrates.

Upon room temperature addition of an equimolar ratio of ester to the FLP, several EPR signals arising from multiple paramagnetic species were detected at room temperature, as shown in Figure S176. Upon comparison with previous reports,[19] the intense two resonance lines with a 1:1 ratio centered on g_{iso} = 2.012 (B ~ 335.0 mT) and separated by a phosphorus hyperfine splitting of $a_{iso}(^{31}P)$ = 670 MHz (23.8 mT) is attributed to formation of the $Mes_3P^{\bullet+}$ cation.

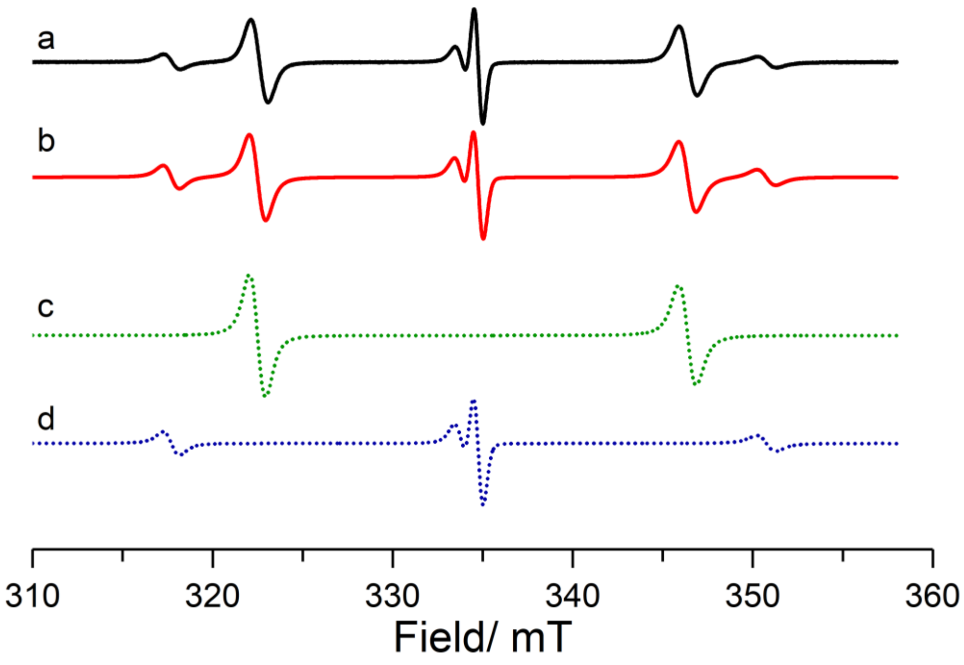

Figure S176: CW X-band EPR spectra (T = 298 K) of Mes_3P + $B(C_6F_5)_3$ + ester **1d**; (a, Black) Experimental and (b, red) simulated; and simulated spectra of (c, green) $Mes_3P^{\bullet+}$ and (d, blue) $(Mes_3P)_2^{\bullet+}$ radicals.
Spectra were recorded using the following experimental parameters: Modulation amplitude = 2.0 G; modulation frequency = 100 kHz; microwave power = 3.2 mW.

A second paramagnetic species is also detected in this sample, with two weak outer hyperfine lines and two resonance features centered on g_{iso} = 2.010, separated by a hyperfine splitting of 470 MHz (16.7 mT). This EPR profile must originate from two identical I = ½ nuclei, in this case associated with two equivalent ^{31}P nuclei. It is noted that in a first-order approximation, coupling to two equivalent phosphorus nuclei yields a three-line (1:2:1) EPR spectrum. However, in this case, the large magnitude of the phosphorus hyperfine coupling leads to splitting of the m_I = 0 transition which appears as a doublet, corresponding to the I = 0 (singlet) or I = 1 (triplet) states of two coupled phosphorus nuclei.[20] The signal is therefore assigned to the formation of a $[P(Mes)_{n=2,3}]_2^{\bullet+}$ dimer. Previous EPR evidence for the formation of phosphorus dimers, e.g. $(R_3P{-}PR_3)^{\bullet+}$, has been provided by Symons *et al*.[21] upon γ-irradiation of trialkyl phosphines, where it was noted that the dimer radicals exhibit similar **g** and **A** tensors to the parent $R_3P^{\bullet+}$ radical, suggesting that the dimer radicals are formed by the reaction:

[Equation S1] $PR_3^{\bullet+} + PR_3 \rightarrow R_3P–PR_3^{\bullet+}$

The spin Hamiltonian parameters for all the paramagnetic species detected in this work are listed in Table S1, with reference values from the literature reported for comparison.

Importantly, no evidence for formation of the carbon-based bismethoxy-diphenylmethylene radical formed upon C–O ester cleavage, was observed. In Figure S177, the center field region of the (FLP + ester) solution is shown, alongside simulations of relevant C-based radicals, namely diphenylmethylene and 4-methoxydiphenylmethylene, using spin Hamiltonian parameters from the literature (see Table S1). For derivatives of the diphenylmethyl radical, all reported EPR spectra in the literature are dominated by a large doublet splitting resulting from interaction of the unpaired electron with the proton of the central carbon atom. Additional coupling to aryl protons gives rise to further minor hyperfine splittings, although these are not always fully resolved in all cases. As can be seen, there is a very poor agreement of the simulations with our experimental data – hence it is not possible to prove the existence of the C-based radical under these experimental conditions.

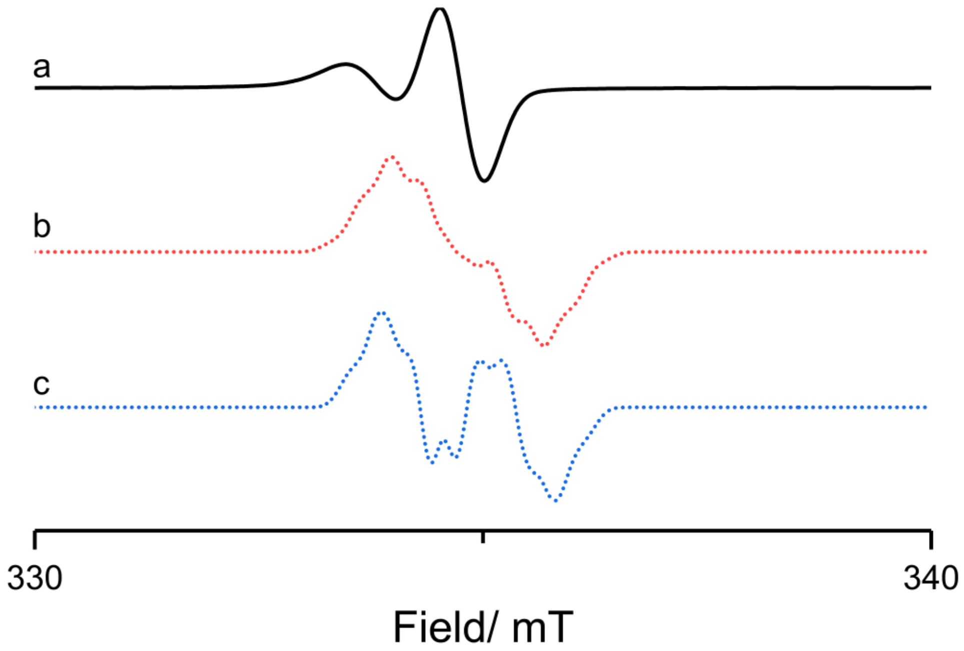

Figure S177: CW EPR spectra (T = 298 K) of (a) experimental spectrum of $Mes_3P + B(C_6F_5)_3$ + ester **1d**; (center field region only); and simulated spectra using literature values of (b) diphenylmethyl radical,[22] and (c) 4-methoxydiphenylmethyl radical.[23]

Spectra were recorded using the following experimental parameters: Modulation amplitude = 2.0 G; modulation frequency = 100 kHz; microwave power = 3.2 mW.

Table S1: Spin Hamiltonian parameters for radical species generated during FLP reactions.

Radical	g_{iso}	a_{iso}/ MHz	Ref
$Mes_3P^{\bullet+}$	2.012; $g_{\|\|}$ = 2.010, $g_{\perp}$ = 2.013	678; $A_{\|\|}$ = 1135, $A_{\perp}$ = 450	
$Mes_3P^{\bullet+}$	2.009; $g_{\|\|}$ = 2.004, $g_{\perp}$ = 2.012	664; $A_{\|\|}$ = 1147, $A_{\perp}$ = 448	
$[P(Mes)_{n=2,3})]_2^{\bullet+}$	2.014; $g_{\|\|}$ = 2.009, $g_{\perp}$ = 2.017	470; $A_{\|\|}$ = 761, $A_{\perp}$ = 325	19
$[P(Mes)_2)]_2^{\bullet+}$	2.006; $g_{\|\|}$ = 2.008, $g_{\perp}$ = 2.002	475; $A_{\|\|}$ = 753, $A_{\perp}$ = 337	24
$(PEt_3)_2^{+}$	2.008; $g_{\|\|}$ = 2.000, $g_{\perp}$ = 2.012	1277; $A_{\|\|}$ = 1511, $A_{\perp}$ = 1160	24
$(PBu_3)_2^{+}$	2.008; $g_{\|\|}$ = 2.000, $g_{\perp}$ = 2.012	1298; $A_{\|\|}$ = 1540, $A_{\perp}$ = 1177	24
Diphenylmethylene	2.0030	$^1H_{methylene}$ = 23.43; $^1H_{6,o/p}$ = 8.55; $^1H_{4,m}$ = 3.42;	22
4-methoxy-diphenyl-methyl	2.008	$^1H_{methylene}$ = 30.3	23
Di(2,6-dimethylphenyl)methyl	2.008	$^1H_{methylene}$ = 45.7; $^1H_{2,o}$ = 6.04; $^1H_{2,m}$ = 4.01; $^1H_{2,p}$ = 9.13	25
Fluorenyl	2.008	$^1H_{methylene}$ = 19.67; $^1H_{2,o}$ = 10.54; $^1H_{4,m}$ = 1.38; $^1H_{2,p}$ = 5.31	26
$B(C_6F_5)_3^{\bullet-}$	2.0114	$^{10,11}B$ = 31; $^{19}F_{6,o}$ = 12.94; $^{19}F_{6,m}$ = 3.66; $^{19}F_{3,p}$ = 14.9	27
	2.0114	$^{10,11}B$ = 27; $^{19}F_{6,o}$ = 18.3; $^{19}F_{6,m}$ = 3; $^{19}F_{3,p}$ = 20.2	
Styrene	2.012	$^1H_{1,2}$ = 10.7; $^1H_{1,3}$ = 2.4; $^1H_{1,4}$ = 15.4; $^1H_{1,5}$ = 1.6; $^1H_{1,6}$ = 5.6; $^1H_{1,\alpha}$ = 4.3; $^1H_{1,\beta}$ = 20.6	28
Styryl/ phenylacetylene	2.0023	$^1H_{2,\beta}$ = 116.3; $^1H_{3,o/p}$ = 17.09	29

For conversion to field units, a/ mT = [10^9 × (h/gμ_B)] × a/ MHz, where g = g-factor, h = Planck constant, μ_B = Bohr magneton.

6. Computational Data

6.1 Computational Details

All density functional theory (DFT) computations were performed by Gaussian 16, Revision B.01.[30] Molecular geometries were optimized by applying the B3LYP[31] functional augmented with Grimme's D3[32] empirical dispersion correction, a combination represented by B3LYP-D3. The 6-31G(d)[33] basis set was used for all atoms. Solvation effects were incorporated using the SMD[34] model with THF and Toluene as the solvent. Frequency calculations were performed at the same level of theory as that used for geometry optimization to characterize the stationary points as either minima (no imaginary frequencies) or first-order saddle points (one imaginary frequency). Transition structures were located using the Berny algorithm. Intrinsic reaction coordinate (IRC)[35] calculations were used to confirm the connectivity between transition structures and minima. Single point energies were calculated with the B3LYP-D3 functional using the def2-TZVP[36] basis set for all atoms and employing the SMD solvation model. To estimate the corresponding Gibbs free energies, entropy corrections were calculated at the B3LYP level and added to the single-point potential energies.

6.2 Additional free energy profiles

Figure S178: Generation of carbocation **I3** from reaction between **1a** and $B(C_6F_5)_3$. The relative free energies are given in kcal/mol.

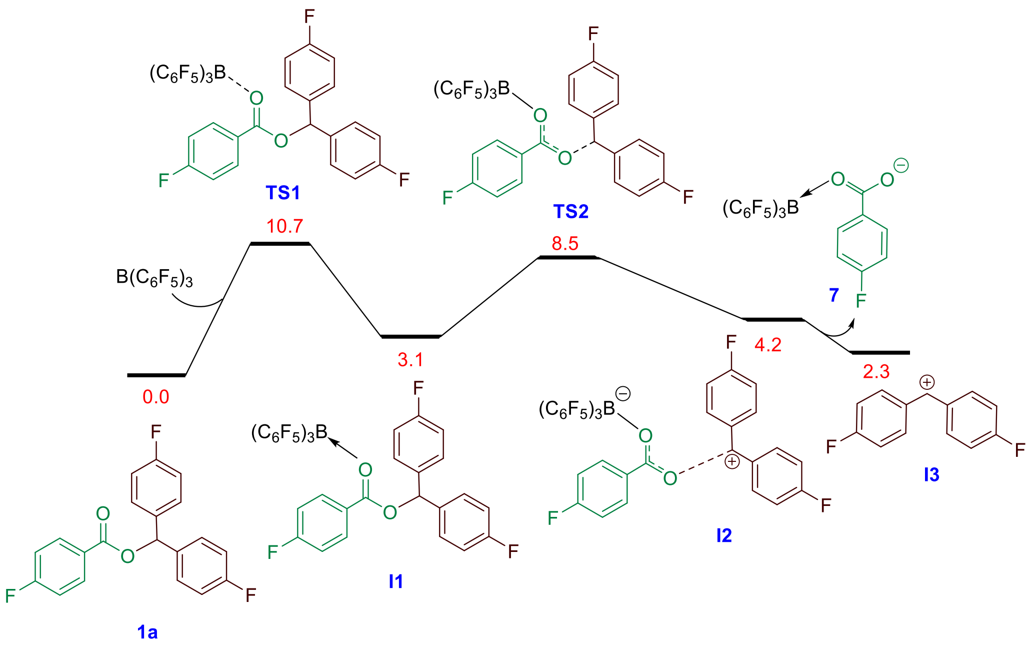

Figure S179: Generation of carbocation **I3$_{1d}$** from reaction between **1d** and B(C_6F_5)$_3$. The relative free energies are given in kcal/mol.

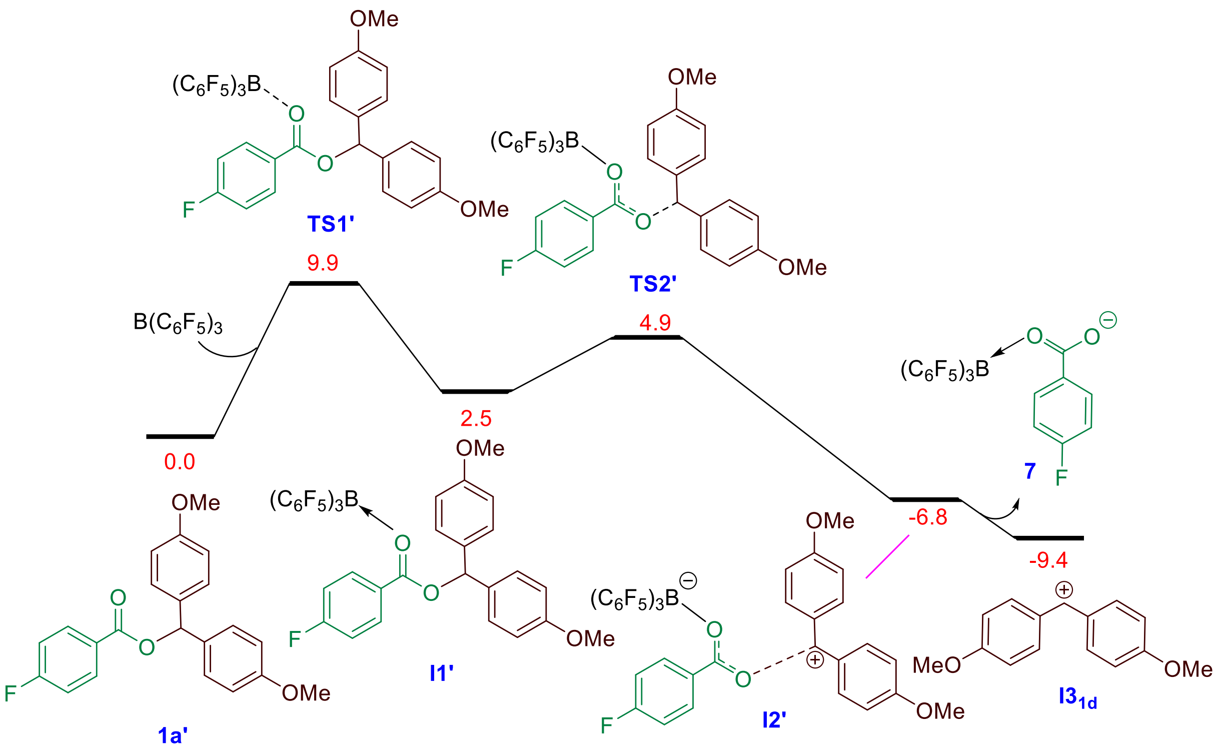

Figure S180: Generation of carbocation **I3$_{1j}$** from reaction between **1j** and B(C_6F_5)$_3$. The relative free energies are given in kcal/mol.

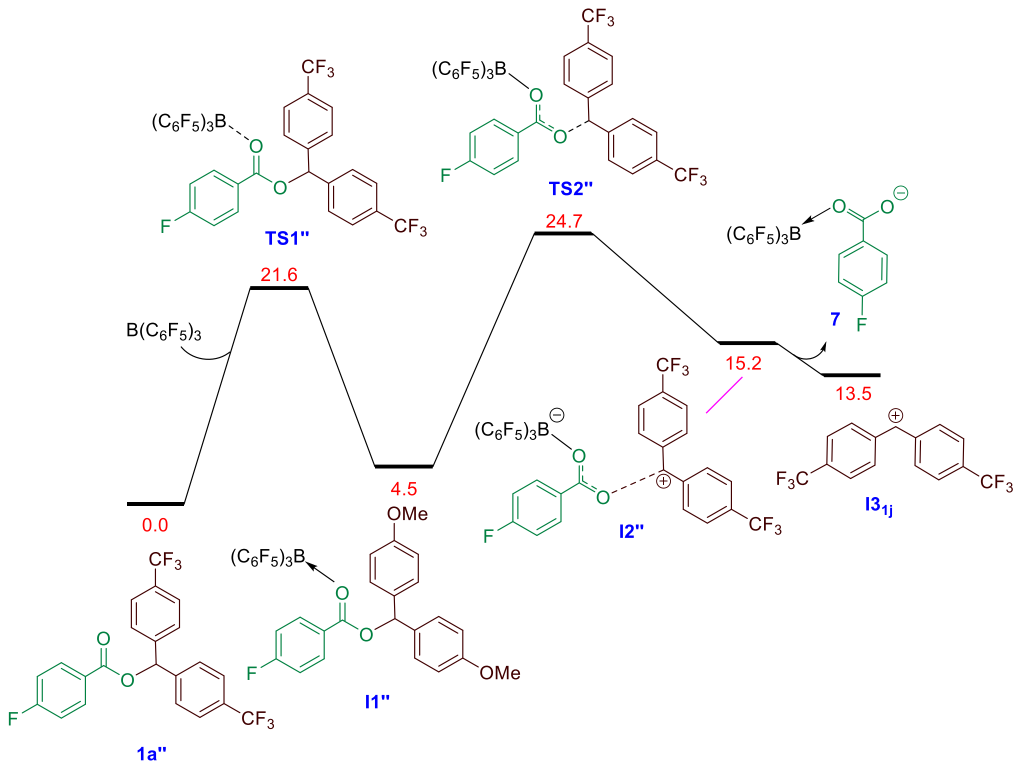

Figure S181. DFT (SMD/B3LYP-D3/def2-TZVP//SMD /B3LYP-D3/6- 31G(d) in THF) computed energy barrier and intermediate (**TS4**, **TS5**, **I7** and **I8**. Table 3; entry 1–9) heights are plotted as a function of Hammett substituent constant in a Hammett-style plot. The relative free energies are given in kcal/mol.

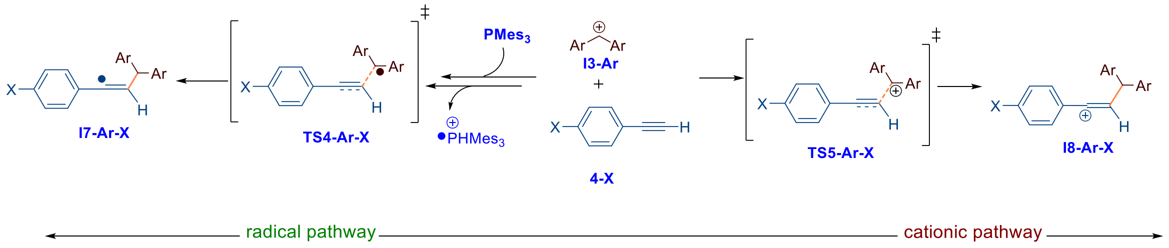

Entry	Ester Ar	X	TS4 radical pathway	TS5 cationic pathway	I7 radical pathway	I8 cationic pathway
1	**1a** (*p*-FC_6H_4)	NO_2	23.9	23.2	1.2	14.5
2	**1a** (*p*-FC_6H_4)	CF_3	26.2	20.1	2.9	11.0
3	**1a** (*p*-FC_6H_4)	H	26.3	18.6	5.3	4.8
4	**1a** (*p*-FC_6H_4)	OMe	26.0	13.9	6.3	-5.0
5	**1a** (*p*-FC_6H_4)	NMe_2	27.0	8.2	8.7	-16.8
6	**1d** (*p*-$OMeC_6H_4$)	CF_3	23.7	13.0	5.9	10.6
7	**1d** (*p*-$OMeC_6H_4$)	OMe	27.8	8.1	8.1	-5.7
8	**1j** (*p*-CF_3 C_6H_4)	CF_3	24.1	25.5	6.2	11.0
9	**1j** (*p*-CF_3 C_6H_4)	OMe	22.2	17.5	3.7	-7.7

Table S2. Benchmark calculations for the reaction of **I3** with 1-ethynyl-2-vinylbenzene/1-ethynyl-4-vinylbenzene and Mes_3P (Figure 4) calculated by SMD/B3LYP-D3/def2-TZVP//SMD/B3LYP-D3/6- 31G(d) in THF.

Entry	TS8-C	TS9-C	I11-C	I12-C
**M06(SMD)**	17.0	16.7	-1.1	4.0
**M06-D3(SMD)**	13.8	13.3	-3.4	1.0
**WB97XD(SMD)**	15.0	14.7	-6.8	-0.4
**M06-2X(SMD)**	15.6	14.4	-3.6	1.8
**M06-2X-D3(SMD)**	14.4	13.2	-4.6	0.7
**M06-2X-(CPCM)**	14.4	13.2	-4.0	1.5
**M06-2X-D3-(CPCM)**	13.2	12.1	-5.0	0.4

6.3 Cartesian coordinates and total energies for the calculated structures

1a
E(SMD/B3LYP-D3/6-31G(d)) = -1219.98341au
H(SMD/B3LYP-D3/6-31G(d)) = -1219.680731au
G(SMD/B3LYP-D3/6-31G(d)) = -1219.753724 au
E(SMD/ B3LYP-D3/def2-TZVP//SMD/B3LYP-D3/6-31G(d)) = -1220.472642 au

C	4.70042400	-0.23822600	-1.30187100
C	3.30990200	-0.23038100	-1.33539500
C	2.56756300	-0.12102400	-0.14937000
C	3.23528900	-0.01796900	1.08128300
C	4.62681700	-0.02429800	1.12887700
C	5.33097900	-0.13467900	-0.06573400
H	5.29518100	-0.32170600	-2.20537200
H	2.78428400	-0.30887600	-2.28103600
H	2.66683400	0.06795200	2.00001100
H	5.16674500	0.05462100	2.06657700
F	6.68057700	-0.14157900	-0.02392600
C	1.08210100	-0.11998800	-0.25626200
O	0.47694700	-0.19236700	-1.30963400
O	0.49252200	-0.04426600	0.95775500
C	-0.96852700	0.02020900	1.06418300
H	-1.08252400	0.03800900	2.15239300
C	-1.64125500	-1.25118700	0.56244500
C	-0.93147800	-2.45791300	0.49953100

C	-3.01189600	-1.26225700	0.27201600
C	-1.56117600	-3.64465600	0.12734700
H	0.12576000	-2.47918300	0.74163400
C	-3.66009700	-2.44216200	-0.09726100
H	-3.58824800	-0.34493400	0.33075600
C	-2.91832700	-3.61315600	-0.16768200
H	-1.01647700	-4.58126400	0.06594000
H	-4.71998800	-2.45801000	-0.32971400
F	-3.53727900	-4.76257200	-0.53223900
C	-1.49176600	1.34557100	0.53677700
C	-1.55534300	2.43020000	1.42170000
C	-1.88089100	1.53736000	-0.79745000
C	-1.99174600	3.68559800	0.99755900
H	-1.25926100	2.29549800	2.45900300
C	-2.32484300	2.78248600	-1.23859500
H	-1.82911000	0.70997900	-1.49355800
C	-2.36902600	3.83450900	-0.33050400
H	-2.04610400	4.53182400	1.67461800
H	-2.63021300	2.94572000	-2.26709300
F	-2.79917100	5.04584200	-0.75591000

6

E(SMD/B3LYP-D3/6-31G(d)) = -308.4094245 au
H(SMD/B3LYP-D3/6-31G(d)) = -308.29245 au
G(SMD/B3LYP-D3/6-31G(d)) = -308.330286 au
E(SMD/ B3LYP-D3/def2-TZVP//SMD/B3LYP-D3/6-31G(d)) = -308.5278846 au

C	-3.23479800	0.00028700	0.00005900
C	-2.02357000	-0.00056200	0.00003800
C	-0.59279400	-0.00026800	0.00001200
C	0.11951900	-1.21541700	-0.00002200
C	0.11902800	1.21517600	0.00002300
C	1.51291700	-1.20980700	-0.00004800
H	-0.42715000	-2.15370000	-0.00003000
C	1.51242500	1.21013700	-0.00000300
H	-0.42802800	2.15323400	0.00005100
C	2.21278000	0.00030500	-0.00003800
H	2.05388700	-2.15215900	-0.00007400
H	2.05301700	2.15270500	0.00000600
H	3.29943300	0.00052800	-0.00005600
H	-4.30420800	0.00028400	-0.00002900

7

E(SMD/B3LYP-D3/6-31G(d)) = -2727.940775 au
H(SMD/B3LYP-D3/6-31G(d)) = -2727.652198 au
G(SMD/B3LYP-D3/6-31G(d)) = -2727.762527 au
E(SMD/ B3LYP-D3/def2-TZVP//SMD/B3LYP-D3/6-31G(d)) = -2729.136029 au

C	4.70042400	-0.23822600	-1.30187100
C	3.30990200	-0.23038100	-1.33539500

C	2.56756300	-0.12102400	-0.14937000
C	3.23528900	-0.01796900	1.08128300
C	4.62681700	-0.02429800	1.12887700
C	5.33097900	-0.13467900	-0.06573400
H	5.29518100	-0.32170600	-2.20537200
H	2.78428400	-0.30887600	-2.28103600
H	2.66683400	0.06795200	2.00001100
H	5.16674500	0.05462100	2.06657700
F	6.68057700	-0.14157900	-0.02392600
C	1.08210100	-0.11998800	-0.25626200
O	0.47694700	-0.19236700	-1.30963400
O	0.49252200	-0.04426600	0.95775500

8

E(SMD/B3LYP-D3/6-31G(d)) = -2728.376582 au
H(SMD/B3LYP-D3/6-31G(d)) = -2728.07606 au
G(SMD/B3LYP-D3/6-31G(d)) = -2728.184367 au
E(SMD/ B3LYP-D3/def2-TZVP//SMD/B3LYP-D3/6-31G(d)) = -2729.567355 au

B	-0.28899000	0.08794100	-0.02168200
C	-0.83550900	-0.73111900	-1.32640500
C	-2.01668900	-0.43921700	-2.00891000
C	-0.18031900	-1.88574000	-1.76082800
C	-2.49758300	-1.20356000	-3.06954000
C	-0.61999500	-2.67319100	-2.81964700
C	-1.79284800	-2.32795800	-3.48136500
C	-0.74506500	1.65109900	0.07082100
C	-1.15307300	2.31011600	1.23043300
C	-0.64410100	2.46835600	-1.05804200
C	-1.47901400	3.66460800	1.26806800
C	-0.95671400	3.82267900	-1.06593900
C	-1.38132600	4.42767700	0.11193200
C	-0.63043400	-0.85689700	1.26532900
C	-1.96331400	-0.95960900	1.66961100
C	0.24521200	-1.71234100	1.92945900
C	-2.40524800	-1.80515800	2.67827400
C	-0.15137300	-2.57649800	2.94913400
C	-1.48629400	-2.62492100	3.32699000
F	0.94128700	-2.31252300	-1.12911100
F	0.06429100	-3.76712400	-3.19401800
F	-2.24047300	-3.07378400	-4.50101500
F	-3.63637500	-0.86370200	-3.69559700
F	-2.76795200	0.62850200	-1.66617400
F	-0.22607500	1.94571500	-2.23247800
F	-0.84696500	4.54857500	-2.19105900
F	-1.68459300	5.73261100	0.13230300
F	-1.86843800	4.24100100	2.41787700

F	-1.23696200	1.66002800	2.40972600
F	-2.89791300	-0.19836800	1.05708500
F	-3.70153100	-1.84642400	3.02582000
F	-1.88681600	-3.45202400	4.30267000
F	0.74711100	-3.36667200	3.56031900
F	1.56552100	-1.74987700	1.61712100
O	1.27072800	0.16195400	-0.25960200
C	2.15195000	0.58156100	0.52974600
O	1.76313600	1.14678100	1.65330500
C	3.55877100	0.43493200	0.17638500
C	3.90699700	-0.54237900	-0.77648300
C	4.55179500	1.23948700	0.76534400
C	5.23634600	-0.72051800	-1.12982600
H	3.13445900	-1.16797800	-1.20917300
C	5.88227700	1.07282400	0.40743200
H	4.29821300	2.02211400	1.47561700
C	6.19713300	0.09253200	-0.53150800
H	5.53989700	-1.47269100	-1.84942200
H	6.66932200	1.68552600	0.83247600
F	7.48346000	-0.07460400	-0.87436100
H	2.51446700	1.32089800	2.25454000

9

E(SMD/B3LYP-D3/6-31G(d)) = -4118.663288 au

H(SMD/B3LYP-D3/6-31G(d)) = -4117.802614 au

G(SMD/B3LYP-D3/6-31G(d)) = -4117.979195 au

E(SMD/ B3LYP-D3/def2-TZVP//SMD/B3LYP-D3/6-31G(d)) = -4120.244664 au

B	-2.66903700	0.19128000	-0.36361100
C	-4.32010600	0.16248700	-0.40187900
C	-5.11688600	1.03299200	-1.14635900
C	-5.03489100	-0.69795900	0.43576800
C	-6.50939200	1.03028800	-1.10903900
C	-6.42507300	-0.73834900	0.50202100
C	-7.17180200	0.13455000	-0.27957700
C	-1.98081400	0.79598000	-1.73287200
C	-0.87150200	1.63689900	-1.79759100
C	-2.41094400	0.32302000	-2.97427300
C	-0.26437600	2.01710800	-2.99092200
C	-1.83064800	0.66950700	-4.19064800
C	-0.73532800	1.52292300	-4.19924400
C	-2.33763800	1.00028700	1.02958800
C	-2.50642600	2.38373600	1.09716600
C	-2.05642000	0.39137100	2.25263200
C	-2.36849200	3.13167800	2.26036300
C	-1.92427000	1.10024800	3.44505400
C	-2.06757600	2.48051000	3.45194000

F	-4.38815000	-1.54080900	1.27638800
F	-7.04992000	-1.59893800	1.32658400
F	-8.51330000	0.11719900	-0.22858900
F	-7.21794900	1.88792100	-1.86590900
F	-4.56049900	1.94740500	-1.97348400
F	-3.46126800	-0.52747800	-3.04931500
F	-2.30753400	0.17772400	-5.34877900
F	-0.13660700	1.86014000	-5.35256800
F	0.83670500	2.79985100	-2.98347600
F	-0.28454700	2.12516000	-0.67805100
F	-2.83684500	3.06988700	-0.02231100
F	-2.52866500	4.46731300	2.24840600
F	-1.91721200	3.17619600	4.59045500
F	-1.60202700	0.46024800	4.58852100
F	-1.87633500	-0.94564000	2.35631800
O	-2.23927400	-1.25152900	-0.28773200
C	-0.97333500	-1.59870500	-0.19385600
O	-0.02414100	-0.81812400	-0.24634500
C	-0.78560800	-3.06932500	-0.01221700
C	-1.81471800	-3.86624800	0.51120100
C	0.45072700	-3.64753000	-0.32541400
C	-1.60854800	-5.22736800	0.72771000
H	-2.76608400	-3.40833900	0.75881100
C	0.66465700	-5.01008600	-0.13462700
H	1.24538500	-3.02117700	-0.71385500
C	-0.37146200	-5.77090200	0.39650700
H	-2.38238900	-5.86473500	1.14308200
H	1.61107400	-5.48210000	-0.37745300
F	-0.16693300	-7.09343700	0.60023300
H	2.22024200	0.72785000	-0.40716100
P	3.37258500	0.17303600	0.14879600
C	4.25905500	-0.54595400	-1.27347600
C	3.52342200	-0.82840800	-2.45490700
C	5.65660100	-0.79367300	-1.22441900
C	4.20025900	-1.39828100	-3.53958200
C	6.27266200	-1.35963000	-2.34093500
C	5.56613300	-1.67782600	-3.50492900
H	3.63557400	-1.62366000	-4.44056300
H	7.34025300	-1.56204100	-2.29888600
C	2.70167100	-0.95980200	1.40319500
C	1.73565900	-0.37956000	2.26963000
C	3.01664100	-2.33614300	1.49639700
C	1.11280100	-1.19138300	3.21726200
C	2.35764900	-3.09556000	2.46957500

C	1.39766100	-2.55399200	3.32485500
H	0.36079000	-0.75250100	3.86499900
H	2.58414700	-4.15646000	2.53624600
C	4.27338700	1.61949200	0.80618900
C	4.27412600	2.76948500	-0.03005500
C	4.93650500	1.63996200	2.05771900
C	4.97929500	3.89910900	0.38904100
C	5.62464500	2.80390000	2.41933500
C	5.66923200	3.93588200	1.60382300
H	4.98272600	4.77813600	-0.25092100
H	6.13937000	2.82323400	3.37677900
C	2.06043700	-0.50211700	-2.63491600
H	1.69433900	-0.91501100	-3.57907600
H	1.42046200	-0.88048900	-1.83443300
H	1.91654400	0.58331300	-2.68074500
C	6.52839300	-0.47738100	-0.03254600
H	7.49923400	-0.97061200	-0.13295800
H	6.70874800	0.59936200	0.05398200
H	6.09190700	-0.81112400	0.91162800
C	6.27448700	-2.28016100	-4.69068900
H	6.90411900	-1.53001100	-5.18705200
H	6.93397800	-3.10000300	-4.38309600
H	5.56620400	-2.66509700	-5.43080500
C	3.50956500	2.84638000	-1.33274100
H	3.76036800	2.03149900	-2.02039700
H	2.42701900	2.81548500	-1.16408000
H	3.72886000	3.78589700	-1.84734300
C	4.91315300	0.51444000	3.06753900
H	5.04528400	-0.47567500	2.62812400
H	5.70542000	0.65951200	3.80755000
H	3.95727800	0.49442600	3.60374600
C	6.44787800	5.16098100	2.00964100
H	6.65391100	5.16951900	3.08469400
H	7.41249500	5.19688100	1.48580700
H	5.90735900	6.07889500	1.75282700
C	1.31031600	1.06791900	2.19129600
H	0.68834100	1.23570200	1.30508100
H	0.71434800	1.33080500	3.06736900
H	2.15153400	1.76512600	2.15488300
C	3.98556000	-3.06514600	0.59376500
H	3.68597800	-3.01348200	-0.45711700
H	5.00160500	-2.66794700	0.66243100
H	4.02934200	-4.12096100	0.87461500
C	0.63598300	-3.42335400	4.29051400

H	0.39119600	-2.88388100	5.21189400
H	-0.31286000	-3.73986700	3.83656700
H	1.19554600	-4.32757700	4.55167600

$B(C_6F_5)_3$

E(SMD/B3LYP-D3/6-31G(d)) = -2208.274953 au
H(SMD/B3LYP-D3/6-31G(d)) = -2208.091836 au
G(SMD/B3LYP-D3/6-31G(d)) = -2208.182761 au
E(SMD/ B3LYP-D3/def2-TZVP//SMD/B3LYP-D3/6-31G(d)) = -2209.252271 au

B	-0.00002800	-0.00089500	-0.00032400
C	-1.44118400	-0.61697700	-0.00168200
C	-2.51126900	-0.02227800	-0.68606400
C	-3.79308400	-0.55670200	-0.70283900
C	-4.05084800	-1.72996400	0.00030300
C	-3.02467500	-2.35568900	0.70234600
C	-1.75151700	-1.80095400	0.68315500
C	0.18653000	1.55532700	-0.00002700
C	1.23901200	2.18526900	-0.68029600
C	1.41600600	3.56269900	-0.69744400
C	0.52556400	4.37255600	0.00160300
C	-0.53187200	3.79625700	0.69958300
C	-0.68714200	2.41622100	0.68044400
C	1.25403400	-0.94096300	0.00116900
C	2.43615500	-0.61493800	0.68204200
C	3.55471500	-1.43799700	0.69899700
C	3.52598000	-2.64062800	-0.00114200
C	2.37923600	-3.00732000	-0.69965100
C	1.27370100	-2.16685500	-0.68015400
F	-2.32343700	1.10973300	-1.38725800
F	-4.77832500	0.04336300	-1.38355900
F	-5.27771300	-2.25310100	0.00153400
F	-3.27282800	-3.48141000	1.38447600
F	-0.80352400	-2.44912800	1.38287000
F	2.12921500	1.45729000	-1.37747200
F	2.43066000	4.11592900	-1.37459600
F	0.68494200	5.69676800	0.00259100
F	-1.38674300	4.57372400	1.37701300
F	-1.72518600	1.91959400	1.37637100
F	0.19789500	-2.57412100	-1.37676100
F	2.35200600	-4.16169400	-1.37850300
F	4.59428400	-3.43914200	-0.00328600
F	4.65544600	-1.08649700	1.37650200
F	2.52376200	0.53114800	1.38002900

I1

E(SMD/B3LYP-D3/6-31G(d)) = -3428.299004 au
H(SMD/B3LYP-D3/6-31G(d)) = -3427.811112 au
G(SMD/B3LYP-D3/6-31G(d)) = -3427.948037 au
E(SMD/ B3LYP-D3/def2-TZVP//SMD/B3LYP-D3/6-31G(d)) = -3429.749139 au

C	3.73018000	-2.64290400	-0.59566300
C	2.41806300	-2.19312700	-0.50909900
C	1.77033400	-1.69324800	-1.64969400

C	2.44146500	-1.66030500	-2.88500400
C	3.76112700	-2.08528900	-2.97427800
C	4.37953200	-2.56782300	-1.82414600
H	4.25246800	-3.03844600	0.26845200
H	1.89825300	-2.24826100	0.43731800
H	1.93991600	-1.27194600	-3.76410000
H	4.31180600	-2.04536400	-3.90757200
F	5.65754400	-2.98042900	-1.90387800
C	0.39122200	-1.17817200	-1.57885300
O	-0.11317500	-0.50314000	-0.64408900
O	-0.30817800	-1.49941400	-2.63469200
C	-1.77726900	-1.25384700	-2.85574600
H	-1.81450000	-1.46373500	-3.92713500
C	-2.17338300	0.19245400	-2.64341200
C	-1.29041700	1.21829300	-3.00899400
C	-3.45984000	0.52487500	-2.20390200
C	-1.66273900	2.55496900	-2.89851500
H	-0.29323300	0.98015700	-3.36234500
C	-3.85811300	1.85912800	-2.11267600
H	-4.16058500	-0.25399100	-1.92517800
C	-2.94483600	2.84765300	-2.44973100
H	-0.97743400	3.35901400	-3.14426900
H	-4.84207900	2.13416100	-1.74982800
F	-3.30267200	4.14574600	-2.30053800
B	0.44977900	0.37566700	0.58897300
C	0.05506600	-0.45197500	1.94081500
C	0.02650900	0.23129500	3.15944200
C	-0.37403700	-1.77492000	2.01173300
C	-0.42020900	-0.32511400	4.35076000
C	-0.85208500	-2.36945400	3.17820100
C	-0.87799100	-1.63974600	4.35840700
C	-0.48399300	1.72594300	0.51988100
C	-0.02992200	3.03990200	0.37018500
C	-1.86574600	1.62380200	0.70395400
C	-0.86886900	4.15302200	0.37831700
C	-2.73584200	2.70665800	0.73587700
C	-2.23530500	3.98882600	0.55609900
C	2.03848600	0.65661100	0.37586400
C	3.03985900	0.41724900	1.31641900
C	2.50428800	1.13085300	-0.85242500
C	4.39672200	0.60825700	1.05613200
C	3.84179300	1.31296300	-1.16622500
C	4.80335600	1.05165000	-0.19430500
F	-0.34950200	-2.58420800	0.92294300

F	-1.29081900	-3.63974300	3.16094900
F	-1.32541500	-2.19502700	5.49258500
F	-0.41905800	0.38915300	5.48750700
F	0.46591800	1.50826000	3.21451700
F	-2.44365600	0.41269300	0.87844800
F	-4.05716900	2.51793100	0.88876000
F	-3.05742600	5.04438500	0.54586400
F	-0.36155900	5.38583900	0.21272900
F	1.28185200	3.32478800	0.22605500
F	2.75371100	-0.06679700	2.54315400
F	5.31322300	0.34400400	2.00103400
F	6.10289400	1.22061500	-0.46648500
F	4.21393800	1.72809400	-2.38711200
F	1.61371500	1.39422200	-1.84098200
C	-2.53659100	-2.34004300	-2.13398100
C	-2.73693300	-2.30738100	-0.74682900
C	-2.98543200	-3.44904300	-2.86373800
C	-3.35709100	-3.36842700	-0.09229100
H	-2.40332600	-1.45325700	-0.17789100
C	-3.62550200	-4.51176200	-2.22751100
H	-2.83128700	-3.48612800	-3.93887500
C	-3.79107500	-4.45118300	-0.84890800
H	-3.49228700	-3.37012200	0.98437400
H	-3.98302000	-5.37610800	-2.77721900
F	-4.39679400	-5.48371800	-0.22080400

I2

E(SMD/B3LYP-D3/6-31G(d)) = -3428.290827 au
H(SMD/B3LYP-D3/6-31G(d)) = -3427.803899 au
G(SMD/B3LYP-D3/6-31G(d)) = -3427.943142 au
E(SMD/ B3LYP-D3/def2-TZVP//SMD/B3LYP-D3/6-31G(d)) = -3429.744017 au

C	-3.83427900	2.64431700	-1.51036500
C	-2.51879500	2.18728100	-1.45141700
C	-2.11855100	1.07960200	-2.20745600
C	-3.04138300	0.44692600	-3.05348800
C	-4.36523000	0.87499300	-3.10099900
C	-4.73624600	1.96484600	-2.31920200
H	-4.16569900	3.50012800	-0.93182500
H	-1.80180400	2.70210800	-0.82734400
H	-2.72221800	-0.40001800	-3.65201500
H	-5.10462900	0.38104300	-3.72281500
F	-6.02201700	2.38369500	-2.35797200
C	-0.71999900	0.54011200	-2.15976200
O	-0.12342600	0.34047900	-1.00045000
O	-0.13260800	0.28800700	-3.21253700
C	2.86713000	0.07927800	-2.29002800
C	3.07049100	-1.25316100	-1.86194200
C	1.92302400	-2.10056800	-1.86413400

C	4.31340600	-1.77922200	-1.40438000
C	1.98527500	-3.37911100	-1.34386200
H	0.98268000	-1.70621200	-2.22890300
C	4.38735900	-3.06588400	-0.91131400
H	5.21584800	-1.18477500	-1.47821200
C	3.21423400	-3.82940900	-0.86175500
H	1.11168900	-4.01755100	-1.28505900
H	5.31785300	-3.49563500	-0.55804400
F	3.28424400	-5.05638700	-0.33825800
B	-0.68759200	-0.02985600	0.36661000
C	-0.71608700	1.25221200	1.39193700
C	-1.17472000	1.06577600	2.69734000
C	-0.14741700	2.50032000	1.15164700
C	-1.12602500	2.03876400	3.68664700
C	-0.07708000	3.51006100	2.11142900
C	-0.56542100	3.27780700	3.38993600
C	0.46334400	-1.01797000	1.03646100
C	0.24974700	-2.24671100	1.66027400
C	1.79291600	-0.58909100	1.05507400
C	1.27365400	-3.03134300	2.19009600
C	2.84240700	-1.33038800	1.58418900
C	2.58499500	-2.57601100	2.14158200
C	-2.12990600	-0.77838900	0.12185900
C	-3.38484800	-0.27971600	0.46618000
C	-2.16834200	-1.95108400	-0.63480700
C	-4.58414800	-0.87749500	0.08273800
C	-3.33531000	-2.59060100	-1.03070800
C	-4.56192800	-2.04531300	-0.66671300
F	0.35072000	2.82209000	-0.06669700
F	0.46910000	4.70246000	1.81059800
F	-0.50316600	4.23615400	4.32751800
F	-1.59788600	1.79613900	4.92220800
F	-1.70454500	-0.13057000	3.04622600
F	2.12656600	0.61105700	0.52594500
F	4.11090900	-0.87459400	1.52711500
F	3.58905100	-3.33009100	2.61236500
F	1.00452100	-4.22714900	2.74090300
F	-0.99104000	-2.76769100	1.77507900
F	-3.51433400	0.87793800	1.15047600
F	-5.76052400	-0.31672900	0.41185200
F	-5.70585600	-2.63114600	-1.04988600
F	-3.29247400	-3.71662400	-1.76471100
F	-1.00889300	-2.51776200	-1.05879000
H	1.94577000	0.24068500	-2.85026800
C	3.65522500	1.23756300	-2.07126300
C	4.62513100	1.36349500	-1.03605100
C	3.39869800	2.35996500	-2.90899500
C	5.34720400	2.53209300	-0.89210600
H	4.74460700	0.57508600	-0.30502300
C	4.13442600	3.52368000	-2.78577200
H	2.62729100	2.28500400	-3.66934100

C	5.10113800	3.58350600	-1.78054500
H	6.07270700	2.66384100	-0.09692300
H	3.96975300	4.38299400	-3.42619600
F	5.80427600	4.71120300	-1.63832200

I3

E(SMD/B3LYP-D3/6-31G(d)) = -700.3143863 au

H(SMD/B3LYP-D3/6-31G(d)) = -700.118093 au

G(SMD/B3LYP-D3/6-31G(d)) = -700.16965 au

E(SMD/ B3LYP-D3/def2-TZVP//SMD/B3LYP-D3/6-31G(d)) = -700.586296 au

C	1.29075300	0.56477200	0.03961900
C	2.38888500	1.42853400	-0.25295000
C	3.68395700	0.95135900	-0.28710900
C	3.89399900	-0.39504500	0.01926500
C	2.85929300	-1.27187600	0.36663200
C	1.56524800	-0.79385500	0.37674600
C	-1.29075300	0.56477100	-0.03960900
C	-1.56524500	-0.79385700	-0.37673400
C	-2.85929000	-1.27187700	-0.36662700
C	-3.89399900	-0.39504700	-0.01926800
C	-3.68396000	0.95135900	0.28710200
C	-2.38888800	1.42853400	0.25294800
H	-0.76774200	-1.45044100	-0.70006100
H	-3.09573600	-2.29453900	-0.63853000
H	-4.52866700	1.58653800	0.52908100
H	-2.19480700	2.47288500	0.47882400
H	0.76774800	-1.45043900	0.70008300
H	2.19480200	2.47288400	-0.47882500
H	4.52866300	1.58653800	-0.52909400
H	3.09574000	-2.29453600	0.63853600
F	5.14152200	-0.86304000	0.00689900
F	-5.14152300	-0.86304000	-0.00691400
C	0.00000000	1.14423800	0.00000600
H	0.00000100	2.23376500	0.00000100

I4

E(SMD/B3LYP-D3/6-31G(d)) = -2090.562726 au

H(SMD/B3LYP-D3/6-31G(d)) = -2089.804989 au

G(SMD/B3LYP-D3/6-31G(d)) = -2089.919107 au

E(SMD/ B3LYP-D3/def2-TZVP//SMD/B3LYP-D3/6-31G(d)) = -2091.236175 au

C	-1.63444000	-1.17192300	0.69088700
C	-2.18644900	-2.45943600	0.42454800
C	-3.53410100	-2.67872100	0.72485400
C	-4.35423300	-1.70456800	1.29974700
C	-3.77064600	-0.48430000	1.62813800
C	-2.43331400	-0.19449500	1.34500600
C	0.93151500	-1.96582200	-0.89401800
C	1.96353000	-2.75174500	-0.30136100

C	2.86226500	-3.42118100	-1.13736000
C	2.77130100	-3.38939300	-2.52900500
C	1.65195700	-2.76399400	-3.07721700
C	0.71143100	-2.08732500	-2.29594300
C	1.16702600	-0.26121600	1.52831000
C	2.45238200	0.27618000	1.23673200
C	3.21327500	0.81817700	2.27920000
C	2.78512500	0.80458700	3.60315800
C	1.59857100	0.12241200	3.88525700
C	0.79281900	-0.44471200	2.89539800
P	0.04594800	-0.68997700	0.12797200
H	-4.36783100	0.27837000	2.12286000
H	-3.95377400	-3.65966600	0.51677500
H	3.65328000	-4.00588300	-0.67348000
H	1.47275900	-2.83642900	-4.14769200
H	4.18181600	1.24861100	2.03584900
H	1.30451600	-0.01030400	4.92388700
C	3.17474900	0.20731400	-0.09374500
H	3.95806300	-0.55768700	-0.01910800
H	2.55919000	-0.05857900	-0.94666700
H	3.66022500	1.16181700	-0.31393300
C	-0.32766000	-1.32997200	3.41505100
H	-1.25631100	-0.78758500	3.60859100
H	-0.56934700	-2.15705200	2.74879900
H	-0.00800900	-1.76173100	4.36867600
C	3.58837300	1.45272100	4.69973300
H	3.16523000	2.43301700	4.95716900
H	3.57913200	0.84784600	5.61327600
H	4.62849900	1.61096500	4.39750300
C	-1.95320000	1.15004000	1.84839600
H	-2.62374800	1.94290900	1.50957700
H	-1.96697400	1.15762300	2.94456700
H	-0.94390000	1.41285700	1.54587600
C	-1.42626500	-3.66055700	-0.09621200
H	-1.18974900	-3.59070700	-1.16035900
H	-0.48861700	-3.82144100	0.43927600
H	-2.03780300	-4.55689700	0.03995300
C	-5.81452800	-1.96878100	1.55617100
H	-6.41278600	-1.69273600	0.67726100
H	-6.00168300	-3.02872700	1.75819900
H	-6.18632200	-1.38119300	2.40181600
C	2.14468700	-3.01777700	1.18086400
H	2.87035000	-2.34437400	1.64648900
H	1.21530300	-2.94984900	1.74547600

H	2.52240600	-4.03812400	1.30419600
C	-0.55864800	-1.67913500	-3.01629800
H	-0.46829600	-0.74829000	-3.58813700
H	-0.81692300	-2.46190800	-3.73687000
H	-1.40359600	-1.59047000	-2.33532400
C	3.80656500	-4.05204000	-3.39856000
H	4.61117400	-3.34483400	-3.64154100
H	4.26708000	-4.90794100	-2.89389500
H	3.37685100	-4.39513500	-4.34556500
C	-0.20911000	0.87036000	-1.08262900
C	-1.61304400	1.49273000	-1.15533400
C	-1.81751400	2.86458900	-0.92922500
C	-2.72758600	0.73454200	-1.55478800
C	-3.07557600	3.45083700	-1.06194300
H	-0.99256200	3.50423900	-0.64568600
C	-3.99379000	1.29815200	-1.69083800
H	-2.62923200	-0.32479800	-1.74286900
C	-4.14845200	2.65317700	-1.43381600
H	-3.22455500	4.51065800	-0.88290900
H	-4.84884500	0.69957600	-1.98688600
C	0.88928500	1.91837000	-1.04176200
C	1.18150000	2.70483700	0.08265500
C	1.59030400	2.17247800	-2.23116500
C	2.15685500	3.69966900	0.03346100
H	0.65537000	2.54546700	1.01593300
C	2.56563100	3.16474100	-2.30325200
H	1.37429600	1.58370100	-3.11871200
C	2.83508900	3.90832100	-1.16055700
H	2.39410800	4.30334400	0.90306700
H	3.11100200	3.36325800	-3.21996300
F	3.78207300	4.87180600	-1.21463300
F	-5.37340100	3.21169600	-1.55855200
H	-0.07113500	0.34336600	-2.02163900

I5

E(SMD/B3LYP-D3/6-31G(d)) = -2090.542153 au
H(SMD/B3LYP-D3/6-31G(d)) = -2089.789111 au
G(SMD/B3LYP-D3/6-31G(d)) = -2089.914587 au
E(SMD/ B3LYP-D3/def2-TZVP//SMD/B3LYP-D3/6-31G(d)) = -2091.215521 au

C	-0.82255800	-1.55523100	0.86768400
C	-1.28597000	-2.87237900	0.58638200
C	-2.59949500	-3.19412500	0.93469800
C	-3.45979100	-2.27992600	1.55060300
C	-2.96854100	-1.00207400	1.84841900
C	-1.67471000	-0.61204600	1.51971900
C	1.56604300	-1.77622000	-1.05263800

C	2.81770900	-2.44071700	-0.92894700
C	3.38053000	-2.99561700	-2.07958100
C	2.76152200	-2.91547900	-3.33211300
C	1.52426200	-2.26645800	-3.42269900
C	0.91100300	-1.68911600	-2.31293300
C	1.85959600	-0.23710800	1.55973700
C	2.68735300	0.83679400	1.12541200
C	3.52075400	1.44872500	2.05998800
C	3.56101100	1.04064600	3.39878400
C	2.74453600	-0.02486800	3.79586100
C	1.88650000	-0.67867400	2.91058300
P	0.77859500	-0.96927300	0.33751200
H	-3.62667900	-0.28231600	2.32725900
H	-2.95952900	-4.19814500	0.72532500
H	4.32896600	-3.52000700	-1.99306600
H	1.02691100	-2.20347300	-4.38733700
H	4.15399700	2.27103100	1.73617400
H	2.78599100	-0.36623200	4.82722000
C	2.71166700	1.32324700	-0.30431900
H	3.18440100	0.58834200	-0.96683500
H	1.70613400	1.51673400	-0.69360200
H	3.27883200	2.25424000	-0.37938700
C	1.06222400	-1.83484000	3.42353700
H	0.00682600	-1.56216800	3.53374800
H	1.10393600	-2.69932300	2.75192100
H	1.42758700	-2.15321300	4.40375000
C	4.44618800	1.74757600	4.39058300
H	3.93466700	2.63004800	4.79867400
H	4.70454200	1.09892100	5.23368300
H	5.37169800	2.09801200	3.92139800
C	-1.22687200	0.78261500	1.88332300
H	-2.09085500	1.40878200	2.11832300
H	-0.56806200	0.77156000	2.75966900
H	-0.67567400	1.26627900	1.07215100
C	-0.43302400	-3.95313600	-0.03472900
H	-0.32948900	-3.82102800	-1.11716800
H	0.57704200	-3.97674200	0.38635300
H	-0.89070000	-4.93194900	0.13510000
C	-4.87813900	-2.64345000	1.89800300
H	-5.57065600	-1.87009200	1.54826200
H	-5.17067100	-3.60199300	1.45891900
H	-5.00299900	-2.71773700	2.98647700
C	3.54684900	-2.61423800	0.38220500
H	4.02625100	-1.68421400	0.70659700

H	2.87563300	-2.93004600	1.18776800
H	4.32856100	-3.37225400	0.28033600
C	-0.42932400	-1.01687700	-2.48147900
H	-0.66810600	-0.89693700	-3.54208200
H	-1.23414400	-1.60263600	-2.02224500
H	-0.45082300	-0.02446300	-2.01930300
C	3.41835400	-3.49170200	-4.55821000
H	4.00831500	-2.72107400	-5.07306400
H	4.09938200	-4.31008100	-4.30348600
H	2.67612900	-3.86316600	-5.27276300
C	-1.80809600	2.23310900	-2.13490000
C	-2.97648900	1.80742900	-1.41192900
C	-3.55282100	2.49036600	-0.30924000
C	-3.61443600	0.60925200	-1.83735900
C	-4.65730100	1.97782000	0.36355900
H	-3.15978900	3.45076400	0.00082700
C	-4.71100400	0.08467200	-1.16822800
H	-3.21229800	0.07599600	-2.69420400
C	-5.20680600	0.77481100	-0.06602000
H	-5.10067400	2.50094900	1.20478300
H	-5.17817200	-0.84428300	-1.47864800
C	-0.70300400	3.05398000	-1.74152800
C	-0.48094600	3.57209700	-0.43349300
C	0.30795600	3.31180500	-2.71363900
C	0.66740000	4.28934100	-0.12138000
H	-1.19228500	3.38226400	0.35852600
C	1.45442400	4.03051800	-2.41128800
H	0.17755600	2.92524600	-3.72098200
C	1.61869700	4.50709600	-1.11273000
H	0.84278400	4.66330100	0.88225300
H	2.22359700	4.21504400	-3.15430000
F	2.75653900	5.16951100	-0.79673100
F	-6.26039300	0.25545500	0.61373100
H	-1.70386600	1.78475700	-3.12092000

I6

E(SMD/B3LYP-D3/6-31G(d)) = -700.4761188 au
H(SMD/B3LYP-D3/6-31G(d)) = -700.282625 au
G(SMD/B3LYP-D3/6-31G(d)) = -700.33521 au
E(SMD/ B3LYP-D3/def2-TZVP//SMD/B3LYP-D3/6-31G(d)) = -700.7564098 au

C	-0.00000400	1.20913300	-0.00003900
C	-1.30189600	0.60570500	0.01603300
C	-1.56592800	-0.74155200	0.38596400
C	-2.42422100	1.41810000	-0.30927600
C	-2.85864900	-1.25229000	0.39094000
H	-0.75642000	-1.38105400	0.71686700

C	-3.71782300	0.91581300	-0.31125400
H	-2.25932700	2.45885800	-0.57586700
C	-3.91453000	-0.41981500	0.03200000
H	-3.05989700	-2.27804000	0.68344600
H	-4.56984700	1.53609700	-0.57090300
C	1.30189800	0.60570500	-0.01607700
C	1.56594100	-0.74154200	-0.38602300
C	2.42420500	1.41809500	0.30928900
C	2.85866500	-1.25227900	-0.39096600
H	0.75644500	-1.38104000	-0.71696400
C	3.71781000	0.91581100	0.31130100
H	2.25930100	2.45884700	0.57589800
C	3.91453100	-0.41980900	-0.03197500
H	3.05992400	-2.27802300	-0.68348500
H	4.56982200	1.53609200	0.57099600
F	5.17368600	-0.91866900	-0.03239400
F	-5.17368500	-0.91867500	0.03245900
H	-0.00000200	2.29790900	-0.00005900

I7

E(SMD/B3LYP-D3/6-31G(d)) = -1008.914587 au
H(SMD/B3LYP-D3/6-31G(d)) = -1008.600318 au
G(SMD/B3LYP-D3/6-31G(d)) = -1008.672592 au
E(SMD/ B3LYP-D3/def2-TZVP//SMD/B3LYP-D3/6-31G(d)) = -1009.305473 au

C	-2.31015500	-0.14007400	0.68880100
C	-2.56745600	-1.18355400	-0.21013500
C	-3.79313400	-1.28045400	-0.87310300
C	-4.76002900	-0.31676400	-0.62178200
C	-4.54602200	0.73327100	0.26388000
C	-3.31541000	0.81198400	0.91453800
C	-0.00379500	0.94482700	0.65794900
C	0.12813200	0.92448700	-0.73642600
C	1.07968000	1.71463500	-1.38196100
C	1.89926100	2.52896000	-0.61114500
C	1.79565600	2.58487300	0.77271100
C	0.83847500	1.78488600	1.39804800
H	-0.51347800	0.28125000	-1.33065300
H	1.19399100	1.70134500	-2.46109700
H	2.45262400	3.23823000	1.33792700
H	0.75464400	1.80761000	2.48181200
H	-3.13224000	1.62980900	1.60715300
H	-1.80452100	-1.93404100	-0.39404700
H	-4.00194500	-2.08618700	-1.56964900
H	-5.33066800	1.46349200	0.43451000
F	-5.95499500	-0.40402900	-1.25695700
F	2.82821300	3.29927100	-1.23067800

C	-0.96227800	0.01089100	1.38970300
H	-1.16336700	0.45578900	2.37381200
C	-0.31187100	-1.35373800	1.67103500
C	0.84209200	-1.74872800	1.20006500
C	2.04334000	-1.68678000	0.50651100
C	2.14563600	-2.17402100	-0.83245500
C	3.21829000	-1.15229100	1.11813800
C	3.35030400	-2.10669000	-1.51605900
H	1.26108000	-2.58506700	-1.31019300
C	4.41237800	-1.09284200	0.41524500
H	3.15501900	-0.77595600	2.13461500
C	4.49125400	-1.56723900	-0.90278100
H	3.40702000	-2.47184500	-2.53837200
H	5.29294700	-0.66918200	0.89145800
H	5.43059500	-1.51651900	-1.44596300
H	-0.90676800	-2.00973500	2.31272800

I8

E(SMD/B3LYP-D3/6-31G(d)) = -1008.747861 au
H(SMD/B3LYP-D3/6-31G(d)) = -1008.431769 au
G(SMD/B3LYP-D3/6-31G(d)) = -1008.502508 au
E(SMD/ B3LYP-D3/def2-TZVP//SMD/B3LYP-D3/6-31G(d)) = -1009.131743 au

C	0.96248600	1.31854700	0.13302400
C	1.79747200	1.62418600	-0.95271100
C	2.36530300	2.88893800	-1.08090700
C	2.08538500	3.84309400	-0.10692400
C	1.26258600	3.57736100	0.97999200
C	0.69862300	2.30545500	1.08993500
C	1.32059400	-1.22190000	0.16766100
C	1.01696300	-2.40833100	-0.50971500
C	1.92152700	-3.47224600	-0.54023600
C	3.13162600	-3.33283100	0.12412100
C	3.46611200	-2.17295500	0.81415300
C	2.55442300	-1.11980200	0.82925500
H	0.06532900	-2.52070900	-1.02022100
H	1.69697700	-4.39364800	-1.06764000
H	4.42295100	-2.10292600	1.32109900
H	2.81034900	-0.20773400	1.35944700
H	0.05205700	2.07970900	1.93389500
H	2.01300100	0.86344500	-1.69808400
H	3.01583700	3.14169200	-1.91165000
H	1.07551200	4.35087100	1.71745500
F	2.63560700	5.07233600	-0.22394300
F	4.01558000	-4.35693900	0.09945600
C	0.32939000	-0.06021600	0.26465800
H	-0.16917700	-0.11306800	1.23791700

C	-0.75719400	-0.16678500	-0.82125200
C	-2.01370500	-0.25357900	-0.56730900
C	-3.34035000	-0.31540100	-0.26644100
C	-3.98182600	-1.58828200	-0.11225000
C	-4.09082500	0.89677900	-0.10881500
C	-5.32683200	-1.63323700	0.19211100
H	-3.39626700	-2.49303100	-0.23676800
C	-5.43534800	0.82359400	0.19217700
H	-3.58531500	1.84882200	-0.23120500
C	-6.04631000	-0.43407800	0.34102600
H	-5.83078100	-2.58583000	0.31517800
H	-6.02061700	1.72862500	0.31468100
H	-7.10540700	-0.48090300	0.57832800
H	-0.42572700	-0.14376100	-1.86136100

I9

E(SMD/B3LYP-D3/6-31G(d)) = -2399.046073 au
H(SMD/B3LYP-D3/6-31G(d)) = -2398.165768 au
G(SMD/B3LYP-D3/6-31G(d)) = -2398.297915 au
E(SMD/ B3LYP-D3/def2-TZVP//SMD/B3LYP-D3/6-31G(d)) = -2399.827738 au

C	-2.29601500	-1.24767100	-0.50316700
C	-3.70467200	-1.24269200	-0.29825000
C	-4.39536900	-2.45753400	-0.41418700
C	-3.76984300	-3.67003900	-0.69112700
C	-2.38370800	-3.65789000	-0.83807600
C	-1.63026100	-2.48859500	-0.73630500
C	-2.09986800	1.82360600	0.00718700
C	-2.89159500	2.31322600	-1.07966300
C	-3.62707600	3.48457000	-0.91205200
C	-3.62016100	4.21485000	0.27925600
C	-2.80135600	3.75489700	1.30249300
C	-2.01528500	2.59656900	1.19706900
C	-0.14649800	0.64415100	-1.79166500
C	0.72292200	1.77460200	-1.66231800
C	1.59651300	2.08836200	-2.70306700
C	1.64065200	1.35898600	-3.89282400
C	0.72315400	0.32452000	-4.03799100
C	-0.19017500	-0.04454200	-3.03699100
P	-1.19178600	0.23330300	-0.30174800
H	-1.86194700	-4.59209900	-1.03302200
H	-5.47203800	-2.44792700	-0.26552500
H	-4.23496300	3.83594000	-1.74224200
H	-2.73701100	4.32684300	2.22527100
H	2.26762600	2.93390100	-2.57550000
H	0.69022200	-0.22096200	-4.97798200
C	0.78345900	2.70176300	-0.46790600
H	-0.01214600	3.45180400	-0.52263800
H	0.69425300	2.19356500	0.48942100
H	1.74088500	3.22730700	-0.45517800
C	-1.15784300	-1.12795400	-3.47475700

H	-0.71475100	-2.12406800	-3.37578000
H	-2.10229400	-1.13475700	-2.94024000
H	-1.38248100	-0.98388500	-4.53597600
C	2.63333300	1.70134800	-4.97218600
H	3.65192200	1.44210300	-4.65524300
H	2.42284800	1.16248300	-5.90108400
H	2.62848500	2.77650600	-5.18614500
C	-0.13270500	-2.67976600	-0.86145100
H	0.29093500	-2.98237100	0.10100500
H	0.08286700	-3.48191400	-1.57317900
H	0.41684700	-1.80137100	-1.18881500
C	-4.57730500	-0.05701800	0.06230600
H	-4.12300300	0.61619900	0.78626600
H	-4.84240500	0.53848600	-0.81696500
H	-5.51262800	-0.42202000	0.49514100
C	-4.54933100	-4.95599400	-0.78139600
H	-4.47451700	-5.51803100	0.15911400
H	-5.61100400	-4.77090700	-0.97295800
H	-4.16017000	-5.60248900	-1.57581300
C	-3.00523200	1.66022300	-2.44049100
H	-2.14374000	1.90584300	-3.06947600
H	-3.08469300	0.57407400	-2.39939000
H	-3.90123000	2.02378200	-2.95099100
C	-1.08717900	2.40436300	2.38326100
H	-0.41403500	3.26834400	2.43983400
H	-1.66022400	2.38065200	3.31612600
H	-0.46625400	1.51734800	2.35139500
C	-4.46137700	5.45507500	0.42873800
H	-4.28778600	6.15066400	-0.40102200
H	-5.52952400	5.20281700	0.41685000
H	-4.24783800	5.97724700	1.36641400
C	-0.22310500	-0.38503300	1.17412600
C	-1.06947600	-1.07560500	2.21414600
C	-2.28369500	-0.56374600	2.70041100
C	-0.64074100	-2.32113800	2.71246800
C	-3.03153900	-1.25696100	3.65121100
H	-2.66435800	0.38331300	2.34450700
C	-1.38338900	-3.01160200	3.66914700
H	0.27503100	-2.76518500	2.33611300
C	-2.58547600	-2.48408300	4.14355000
H	-3.96627900	-0.83101200	4.00517600
H	-1.02550500	-3.97162700	4.03103400
H	-3.17013400	-3.02542000	4.88209500
C	1.11712900	-0.45043500	1.32100300
C	2.28629300	-0.12911900	0.42839700
H	1.93862600	0.19409100	-0.54812300
H	1.43402800	-0.88274600	2.26860900
C	3.17425900	0.98637300	0.98462100
C	2.96716900	1.57635300	2.23619000
C	4.21756000	1.46526300	0.17766200
C	3.77965000	2.62328900	2.68098300

H	2.15722400	1.24014700	2.87616300
C	5.03566500	2.50969100	0.60154100
H	4.38749200	1.01492800	-0.79683800
C	4.79872300	3.07035600	1.85281700
H	3.62627300	3.09010500	3.64863300
H	5.84276700	2.89034400	-0.01608000
C	3.07805700	-1.42022400	0.19541600
C	3.18449300	-1.94905200	-1.09705100
C	3.70743700	-2.08913600	1.25535500
C	3.89463600	-3.12732700	-1.33555200
H	2.70917500	-1.43768100	-1.92984300
C	4.41684800	-3.26893800	1.03777800
H	3.65576200	-1.67913500	2.26052400
C	4.49383200	-3.76701700	-0.25853600
H	3.98667100	-3.54608900	-2.33242900
H	4.90985200	-3.79649100	1.84785000
F	5.18123900	-4.91290400	-0.47754200
F	5.58774800	4.08844300	2.27270000

I10

E(SMD/B3LYP-D3/6-31G(d)) = -3736.734418 au

H(SMD/B3LYP-D3/6-31G(d)) = -3736.127 au

G(SMD/B3LYP-D3/6-31G(d)) = -3736.284547 au

E(SMD/ B3LYP-D3/def2-TZVP//SMD/B3LYP-D3/6-31G(d)) = -3738.297334 au

B	-1.91577700	-0.20751800	-0.09298800
C	-3.36102000	-0.21974400	0.71176300
C	-3.91140900	-1.31803400	1.37414600
C	-4.19882000	0.89851200	0.66403100
C	-5.16312100	-1.30596100	1.98597400
C	-5.45272300	0.95744100	1.26583300
C	-5.94198000	-0.15753800	1.93501800
C	-0.84880300	-1.33784000	0.44622300
C	-0.04102100	-2.13170700	-0.36448900
C	-0.62138000	-1.50013000	1.81360200
C	0.87779700	-3.05179300	0.13078000
C	0.28038800	-2.41151000	2.35426500
C	1.05814000	-3.18186800	1.49977200
C	-2.35429200	-0.36935000	-1.67462300
C	-2.84244700	-1.59418000	-2.13353800
C	-2.43243100	0.66473300	-2.60649300
C	-3.32994700	-1.81236700	-3.41549800
C	-2.91375200	0.49400000	-3.90506300
C	-3.36602000	-0.75219600	-4.31531800
F	-3.82871800	2.01036100	-0.01556100
F	-6.20133300	2.07288500	1.18819000
F	-7.15093200	-0.12698200	2.51777800
F	-5.62550700	-2.39818500	2.62150300
F	-3.23738900	-2.48709900	1.46713000

F	-1.31165200	-0.75707200	2.70928800
F	0.42065600	-2.53769500	3.68648900
F	1.97762300	-4.03538800	1.98977400
F	1.63233500	-3.77927500	-0.72017900
F	-0.09040600	-2.04525100	-1.71118200
F	-2.85062300	-2.66102000	-1.29820100
F	-3.77164200	-3.02659400	-3.79191800
F	-3.83457300	-0.93247700	-5.56095200
F	-2.95078200	1.53157600	-4.76157300
F	-2.03954000	1.92402100	-2.30318700
O	-1.31909200	1.14604700	0.18917600
C	-0.21417500	1.59862400	-0.36094400
O	0.59924800	0.91677700	-0.98661800
C	-0.02851900	3.07037600	-0.15784500
C	-1.02791900	3.85627200	0.43782300
C	1.15340700	3.67684200	-0.60575500
C	-0.84285900	5.22734500	0.59740100
H	-1.95468300	3.39148700	0.75207400
C	1.35206100	5.04710300	-0.45481500
H	1.91363600	3.06430500	-1.07512200
C	0.34641500	5.79360500	0.14928000
H	-1.60079600	5.85744900	1.05145000
H	2.25885100	5.53574500	-0.79427000
F	0.52952700	7.12531700	0.30282400
C	3.23991800	0.22318800	1.91633200
C	2.08601500	0.99974900	2.09184400
C	1.31903800	0.89417400	3.24888100
C	1.72280300	-0.00572000	4.22816900
C	2.86185900	-0.79081900	4.09258600
C	3.61304600	-0.67669700	2.92383400
C	4.27120400	1.68180000	0.04774600
C	4.35044200	1.89699100	-1.33383100
C	4.57184800	3.17384500	-1.85341300
C	4.72245600	4.22995400	-0.96623600
C	4.68379700	4.05247200	0.41057500
C	4.45893800	2.77169400	0.91064900
H	4.21705300	1.07021000	-2.02423300
H	4.60808200	3.35472100	-2.92259400
H	4.80542800	4.90571600	1.06956300
H	4.40870000	2.62836800	1.98474500
H	4.49801300	-1.29506900	2.79641200
H	1.79252100	1.70265200	1.32330100
H	0.41369400	1.47565900	3.38375300
H	3.13243900	-1.48575300	4.88023300

F	0.97764300	-0.12922300	5.34771700
F	4.87652000	5.48406300	-1.45752700
C	4.03802400	0.28540700	0.62299500
C	3.27621200	-0.60355600	-0.39767800
C	3.83331200	-1.66592300	-0.85103900
C	4.51991400	-2.77625500	-1.25317000
C	5.29473800	-2.73541300	-2.45597700
C	4.48779400	-3.96511700	-0.45505900
C	6.00927600	-3.85279100	-2.83957800
H	5.30442600	-1.82501700	-3.04596300
C	5.21777100	-5.06611500	-0.85627700
H	3.88895000	-3.97995300	0.44783900
C	5.96917900	-5.00943400	-2.04226500
H	6.59949300	-3.84137000	-3.74965600
H	5.20925200	-5.97422400	-0.26290200
H	6.53465200	-5.88406300	-2.35145700
H	2.26456600	-0.28660400	-0.67814900
H	5.01356400	-0.17932500	0.80202800

Mes_3P

E(SMD/B3LYP-D3/6-31G(d)) = -1390.216705 au
H(SMD/B3LYP-D3/6-31G(d)) = -1389.660467 au
G(SMD/B3LYP-D3/6-31G(d)) = -1389.754298 au
E(SMD/ B3LYP-D3/def2-TZVP//SMD/B3LYP-D3/6-31G(d)) = -1390.618137 au

C	-1.62715400	-0.64461200	0.22267000
C	-2.44553700	-0.07794400	-0.78787300
C	-3.72193100	-0.61261300	-1.01246700
C	-4.22031500	-1.70086500	-0.29520400
C	-3.39161600	-2.27081200	0.67400400
C	-2.11974300	-1.76224300	0.95351400
C	0.25554700	1.73036000	0.22323900
C	1.15537300	2.15587800	-0.78744100
C	1.33025100	3.52861800	-1.01214000
C	0.63673100	4.50424400	-0.29493100
C	-0.27116600	4.07140100	0.67421900
C	-0.46636800	2.71560500	0.95393900
C	1.37117500	-1.08727900	0.22415300
C	2.58467500	-0.95534800	0.95625700
C	3.66217100	-1.80069300	0.67550000
C	3.58457000	-2.80149600	-0.29577800
C	2.39322800	-2.91445100	-1.01342000
C	1.29087100	-2.07807300	-0.78765900
P	-0.00050200	-0.00056100	0.82858100
H	-3.74505600	-3.13497300	1.23323900
H	-4.33905400	-0.17078800	-1.79257800
H	2.02127700	3.84227000	-1.79239600
H	-0.84300600	4.80956200	1.23325600
H	4.58692400	-1.67497800	1.23541000
H	2.32022800	-3.66863700	-1.79475900

C	2.77795100	0.10998000	2.01355500
H	2.69186200	1.11991600	1.59426000
H	2.02672800	0.03446500	2.80905300
H	3.76868500	0.02459100	2.47121400
C	0.09370600	-2.26945600	-1.69156800
H	-0.74226100	-2.75701300	-1.17914500
H	-0.28297300	-1.31819800	-2.07679800
H	0.36613100	-2.89404300	-2.54859300
C	4.74258800	-3.73553000	-0.54782100
H	4.70604700	-4.59938200	0.13028400
H	4.72950800	-4.12547300	-1.57162100
H	5.70430200	-3.23655500	-0.38312700
C	-1.29261500	-2.46510200	2.00795000
H	-1.86213100	-3.28022900	2.46570900
H	-0.37650000	-2.89613000	1.58583600
H	-0.97947400	-1.77840500	2.80369600
C	-2.01299800	1.05521000	-1.69103800
H	-2.01874300	2.02275400	-1.17816100
H	-1.00054700	0.90729600	-2.07577000
H	-2.68980300	1.13127700	-2.54837700
C	-5.60953400	-2.23398700	-0.54585500
H	-6.33898200	-1.76308300	0.12762900
H	-5.93830600	-2.03370800	-1.57164300
H	-5.66213300	-3.31496200	-0.37370800
C	1.92015900	1.21477200	-1.69097300
H	2.76222700	0.73733600	-1.17896000
H	1.28618500	0.41071700	-2.07387500
H	2.32243000	1.76258400	-2.54941600
C	-1.48839400	2.35079400	2.00879300
H	-1.91069900	3.25177600	2.46511600
H	-2.31902200	1.77130900	1.58758000
H	-1.04975700	1.73817800	2.80575700
C	0.86927700	5.97399700	-0.54551200
H	1.64317900	6.36987400	0.12663600
H	1.20509500	6.15899800	-1.57188600
H	-0.04024300	6.55993300	-0.37125600

Mes_3P (radical)

E(SMD/B3LYP-D3/6-31G(d)) = -1390.04433 au

H(SMD/B3LYP-D3/6-31G(d)) = -1389.489395 au

G(SMD/B3LYP-D3/6-31G(d)) = -1389.578477 au

E(SMD/ B3LYP-D3/def2-TZVP//SMD/B3LYP-D3/6-31G(d)) = -1390.443679 au

C	-1.50404300	-0.92699400	0.14015600
C	-2.45193900	-0.53991700	-0.84842000
C	-3.60776500	-1.30879300	-0.98112500
C	-3.85975400	-2.42960000	-0.18013400
C	-2.90541500	-2.79375400	0.77717800
C	-1.73113500	-2.06574600	0.96333500
C	-0.05202400	1.76073400	0.14075800
C	0.74974900	2.38899500	-0.85305400
C	0.65994200	3.77475500	-0.98499300

C	-0.18123100	4.55236300	-0.18020600
C	-0.96607500	3.90685900	0.78290400
C	-0.91903200	2.52628400	0.97022500
C	1.55382900	-0.83966300	0.14068600
C	2.65168200	-0.46629000	0.96762200
C	3.87230800	-1.11237400	0.78060800
C	4.04068500	-2.11519600	-0.18181900
C	2.94654000	-2.46001500	-0.98345100
C	1.69890300	-1.85017500	-0.84972800
P	-0.00062000	-0.00272900	0.42711100
H	-3.08470800	-3.66641000	1.40002700
H	-4.32933200	-1.03469500	-1.74665400
H	1.25435200	4.26339000	-1.75326000
H	-1.62915400	4.49780800	1.40956900
H	4.71614800	-0.83051600	1.40548600
H	3.07319700	-3.21972000	-1.75079600
C	2.54692500	0.61403900	2.01870400
H	2.42149300	1.60456500	1.56614600
H	1.69470000	0.45209400	2.68983100
H	3.45290500	0.64112300	2.63048600
C	0.59209600	-2.26042700	-1.79205200
H	-0.16550600	-2.87169300	-1.28987000
H	0.07778100	-1.39226000	-2.21892600
H	1.00081700	-2.84885900	-2.61800800
C	5.36248700	-2.81916500	-0.33457300
H	5.42950600	-3.66230800	0.36660100
H	5.48885900	-3.22187100	-1.34466400
H	6.20033200	-2.14825000	-0.11667400
C	-0.74084800	-2.52616000	2.00744200
H	-1.17555400	-3.31516000	2.62737200
H	0.16830000	-2.92925900	1.54557900
H	-0.43377400	-1.70973300	2.67225900
C	-2.24634100	0.62239800	-1.79003600
H	-2.39743100	1.58505400	-1.28928200
H	-1.23629700	0.63199400	-2.21403100
H	-2.95860800	0.56470800	-2.61797700
C	-5.13755900	-3.21068000	-0.33441800
H	-5.94111000	-2.74889300	0.25544900
H	-5.47141900	-3.22980800	-1.37730000
H	-5.02376700	-4.24141300	0.01612100
C	1.64778300	1.63242800	-1.80279400
H	2.56036500	1.28184400	-1.30857900
H	1.14938200	0.75348700	-2.22454900
H	1.94634800	2.27888500	-2.63273300

C	-1.79879000	1.89958000	2.02695400
H	-2.27394700	2.67247900	2.63764200
H	-2.59491300	1.29364100	1.57854200
H	-1.23094200	1.24536100	2.69953300
C	-0.22253300	6.04936200	-0.33559400
H	0.56095000	6.51895800	0.27489100
H	-0.04739900	6.34817100	-1.37459900
H	-1.18296000	6.46112700	-0.00935300

Mes_3PH

E(SMD/B3LYP-D3/6-31G(d)) = -1390.68101 au
H(SMD/B3LYP-D3/6-31G(d)) = -1390.11345 au
G(SMD/B3LYP-D3/6-31G(d)) = -1390.205345 au
E(SMD/ B3LYP-D3/def2-TZVP//SMD/B3LYP-D3/6-31G(d)) = -1391.08412 au

H	0.00188000	-0.00369600	1.97260300
P	0.00364900	-0.00330300	0.57684200
C	-1.19354200	-1.31049700	0.17797300
C	-1.14635100	-2.46906000	0.99720600
C	-2.14394800	-1.19987300	-0.86844500
C	-2.06642600	-3.49306200	0.75490400
C	-3.03785700	-2.25653500	-1.05565000
C	-3.02268200	-3.40561200	-0.25878400
H	-2.02992900	-4.38475100	1.37560900
H	-3.76727800	-2.18201600	-1.85860700
C	1.73643100	-0.38126500	0.17909100
C	2.71522200	0.24910000	0.99445700
C	2.11975700	-1.26146500	-0.86254500
C	4.06192800	-0.02334700	0.74665700
C	3.48467400	-1.49588700	-1.05545800
C	4.46903000	-0.89536400	-0.26712700
H	4.81437400	0.46406800	1.36166000
H	3.78640500	-2.16528800	-1.85698900
C	-0.53537800	1.68562600	0.18057700
C	-1.56432100	2.22078500	1.00271800
C	0.03329700	2.45631100	-0.86177500
C	-1.99520700	3.52631200	0.76196400
C	-0.43944100	3.75943500	-1.04802500
C	-1.44634300	4.31364500	-0.25500700
H	-2.78343400	3.93930900	1.38675600
H	-0.00696300	4.35715300	-1.84621600
C	-0.14332800	-2.65725700	2.11447300
H	-0.20496700	-3.67415400	2.51051500
H	0.88846400	-2.49748400	1.78169500
H	-0.33141700	-1.97106800	2.95124000
C	-2.24927900	-0.01672800	-1.80375000
H	-2.91839700	-0.25791400	-2.63433100

H	-2.65280000	0.86599200	-1.29701700
H	-1.28413000	0.26826200	-2.23309200
C	-4.02159600	-4.50973500	-0.49024600
H	-5.01800900	-4.20769400	-0.14169600
H	-4.11223800	-4.74440600	-1.55702800
H	-3.74379900	-5.42393600	0.04320800
C	-2.21785300	1.44459600	2.12493200
H	-2.59545800	0.47031800	1.79515800
H	-1.52124500	1.26552700	2.95499100
H	-3.06533400	2.00460000	2.52859600
C	1.11435800	1.96136300	-1.79461800
H	0.88275900	0.98025900	-2.21903500
H	1.23596000	2.65916500	-2.62778800
H	2.08134400	1.87606500	-1.28837800
C	-1.95381100	5.71044800	-0.50342100
H	-1.24217400	6.29907100	-1.09074500
H	-2.90097200	5.68310100	-1.05850500
H	-2.14692500	6.23668000	0.43800200
C	2.37247500	1.20930000	2.11233400
H	1.87167600	0.70067000	2.94674100
H	3.28235200	1.66546200	2.51129100
H	1.71625300	2.02162000	1.78032900
C	1.15136900	-1.95748400	-1.79112500
H	0.58304600	-2.73813400	-1.27510800
H	0.42792100	-1.26713600	-2.23346300
H	1.69695300	-2.43069200	-2.61216900
C	5.92947600	-1.19201800	-0.48899800
H	6.55161000	-0.31505200	-0.28052400
H	6.26425000	-1.99507100	0.18141600
H	6.12156200	-1.51969700	-1.51561000

prod

E(SMD/B3LYP-D3/6-31G(d)) = -1008.33281 au
H(SMD/B3LYP-D3/6-31G(d)) = -1008.02842 au
G(SMD/B3LYP-D3/6-31G(d)) = -1008.100233 au
E(SMD/ B3LYP-D3/def2-TZVP//SMD/B3LYP-D3/6-31G(d)) = -1008.72494 au

C	-1.36279900	-1.24089100	0.56541400
C	-0.85879400	-2.10769000	-0.40882500
C	-1.62203300	-3.18069800	-0.87697300
C	-2.89295900	-3.36969100	-0.35328300
C	-3.42723800	-2.52857200	0.61717000
C	-2.65218800	-1.46288100	1.06964100
C	-1.17431500	1.28030200	0.52369400
C	-1.18118200	1.52666100	-0.85622100
C	-1.72967100	2.69950200	-1.36972900
C	-2.27375100	3.62177400	-0.48104900

C	-2.28554400	3.41175900	0.89052000
C	-1.72830600	2.23001500	1.38690600
H	-0.75510800	0.79203600	-1.53414200
H	-1.74289100	2.90552400	-2.43520300
H	-2.71973700	4.15786800	1.54822100
H	-1.72749200	2.05127300	2.45898200
H	-3.05733500	-0.78920500	1.82047100
H	0.14068300	-1.95372900	-0.80354800
H	-1.24187100	-3.86240800	-1.63108900
H	-4.42503700	-2.71331700	1.00204800
F	-3.63944800	-4.41003000	-0.79905500
F	-2.81102300	4.76433300	-0.97451900
C	-0.57928300	-0.02347200	1.07563900
H	-0.69660200	0.00295900	2.16686600
C	0.85719300	-0.09259200	0.78505400
C	2.04070700	-0.12005500	0.51963100
C	3.43455300	-0.15305400	0.20352000
C	4.39988200	-0.25039600	1.22507400
C	3.86053300	-0.08811600	-1.13811200
C	5.75642300	-0.28143000	0.90776500
H	4.07609000	-0.30082800	2.26045900
C	5.21919000	-0.12030000	-1.44558500
H	3.11928100	-0.01155100	-1.92831600
C	6.17062800	-0.21685800	-0.42584100
H	6.49234600	-0.35654100	1.70384800
H	5.53625500	-0.06932100	-2.48374400
H	7.22938700	-0.24140300	-0.66913300

TS1

E(SMD/B3LYP-D3/6-31G(d)) = -3428.286049 au
H(SMD/B3LYP-D3/6-31G(d)) = -3427.7989 au
G(SMD/B3LYP-D3/6-31G(d)) = -3427.935579 au
E(SMD/ B3LYP-D3/def2-TZVP//SMD/B3LYP-D3/6-31G(d)) = -3429.736445 au

C	3.80283900	-0.93383100	2.40406300
C	2.48082500	-0.57341600	2.16042100
C	1.46882500	-1.54405000	2.15687700
C	1.79092900	-2.88879700	2.39569100
C	3.11508600	-3.26741000	2.60210400
C	4.09523200	-2.27937900	2.60015000
H	4.60168000	-0.20030900	2.41073300
H	2.22655200	0.46460800	1.99302200
H	1.00848300	-3.64009600	2.39492600
H	3.39497300	-4.30351600	2.75956200
F	5.37905900	-2.64103500	2.80002800
C	0.07319300	-1.14689500	1.83093800
O	-0.23085600	-0.30240300	1.00067700

O	-0.81862700	-1.82959000	2.56373000
C	-2.25010700	-1.86392000	2.21735800
H	-2.57129600	-2.69769900	2.84750500
C	-2.45594200	-2.29484500	0.77077000
C	-1.91657500	-3.52494400	0.36538300
C	-3.20773800	-1.54858900	-0.14162500
C	-2.11176000	-4.00453100	-0.92698700
H	-1.33645500	-4.11659200	1.06890300
C	-3.42713100	-2.02137100	-1.43693000
H	-3.63716800	-0.59855400	0.15602700
C	-2.86777200	-3.23700600	-1.80528800
H	-1.68800200	-4.94653200	-1.25825300
H	-4.01620500	-1.46125900	-2.15507400
F	-3.06833300	-3.69303400	-3.06464000
B	0.80280000	0.85862800	-1.00511900
C	1.42472300	2.22133500	-0.46739300
C	2.08568400	2.98515400	-1.42958000
C	1.33373800	2.79290300	0.79778100
C	2.63830000	4.23274500	-1.17574700
C	1.87778600	4.04121900	1.09594200
C	2.52941100	4.76629800	0.10557000
C	-0.63694800	0.98486500	-1.62461600
C	-0.95643900	0.49304000	-2.89375700
C	-1.68063400	1.61021800	-0.93618800
C	-2.23442800	0.56556800	-3.43159700
C	-2.97974400	1.66837900	-1.42634500
C	-3.25507000	1.14561700	-2.68408900
C	1.68102200	-0.42659300	-1.15905000
C	3.05736700	-0.44985800	-0.88889700
C	1.10168800	-1.69198300	-1.36395500
C	3.79835900	-1.62304400	-0.80047000
C	1.80647000	-2.88225200	-1.26480900
C	3.16664700	-2.84763000	-0.97044600
F	0.73028000	2.14920300	1.81794800
F	1.77276600	4.54754100	2.33361200
F	3.05283300	5.96657700	0.38214800
F	3.26554900	4.92017800	-2.14057000
F	2.20091300	2.48438600	-2.68149300
F	-1.47203400	2.15010800	0.27609300
F	-3.96767300	2.18423300	-0.67960100
F	-4.50645300	1.15056000	-3.15595200
F	-2.50492200	0.04909800	-4.63862000
F	-0.00832400	-0.09527000	-3.64584800
F	3.73223700	0.68388700	-0.63232600

F	5.09490000	-1.58364300	-0.46242000
F	3.85294700	-3.98236400	-0.82924300
F	1.18464700	-4.06134200	-1.41241700
F	-0.21191700	-1.81083300	-1.59461900
C	-3.03876000	-0.64544300	2.66186200
C	-2.54455500	0.66400300	2.67414600
C	-4.37710300	-0.86516500	3.02539600
C	-3.37002000	1.73389400	3.02602200
H	-1.52372100	0.87205800	2.39185400
C	-5.21535900	0.19146700	3.37467400
H	-4.77523500	-1.87682700	3.02374900
C	-4.69076600	1.47854400	3.36572400
H	-2.99781100	2.75313900	3.03016900
H	-6.25168100	0.03035900	3.65322200
F	-5.49534900	2.51488800	3.70296000

TS2

E(SMD/B3LYP-D3/6-31G(d)) = -3428.284168 au
H(SMD/B3LYP-D3/6-31G(d)) = -3427.798575 au
G(SMD/B3LYP-D3/6-31G(d)) = -3427.935303 au
E(SMD/ B3LYP-D3/def2-TZVP//SMD/B3LYP-D3/6-31G(d)) = -3429.738338 au

C	-4.00400800	1.00312200	-2.32942500
C	-2.62237500	0.88816300	-2.18660400
C	-2.01831000	-0.37404500	-2.16207600
C	-2.80253000	-1.52613100	-2.32230600
C	-4.18588900	-1.42537800	-2.43872300
C	-4.75997700	-0.15774400	-2.43151100
H	-4.49426900	1.97057800	-2.34715900
H	-2.01889000	1.78126900	-2.10755800
H	-2.32769800	-2.50188200	-2.32236700
H	-4.81735700	-2.30299500	-2.52874800
F	-6.10344300	-0.05359800	-2.53942900
C	-0.53929200	-0.55067200	-1.98921000
O	0.10174200	-0.04761700	-0.97553800
O	0.05984400	-1.21051800	-2.85436900
C	2.28085500	-1.78324700	-2.68260200
H	1.97136000	-2.02863600	-3.69168200
C	2.23645100	-2.85694300	-1.74444300
C	1.97814800	-4.16371300	-2.23340900
C	2.38171300	-2.66329000	-0.34661100
C	1.92713000	-5.24574700	-1.37266900
H	1.83654000	-4.31326400	-3.29966200
C	2.31819100	-3.73732300	0.52347700
H	2.48898300	-1.66388800	0.05300600
C	2.10113500	-5.00879500	-0.00763000
H	1.74710000	-6.25580000	-1.72383400

H	2.40044500	-3.60963800	1.59705600
F	2.03699100	-6.04955600	0.83301300
B	-0.39929600	0.45027400	0.38854800
C	-0.61080300	2.07620900	0.39365300
C	-0.99380400	2.69922100	1.58237600
C	-0.26658000	2.95089200	-0.63351700
C	-1.07685200	4.07540900	1.74829400
C	-0.33550500	4.33840400	-0.51667000
C	-0.73884600	4.90662100	0.68428400
C	0.90366400	0.21294800	1.37827600
C	0.92388500	-0.46667000	2.59556100
C	2.12899200	0.78114800	1.01274800
C	2.07601500	-0.63818000	3.36259000
C	3.29954700	0.64473600	1.75247200
C	3.27560300	-0.08399900	2.93643700
C	-1.71814000	-0.44082600	0.78924500
C	-3.02391200	0.02723800	0.92483000
C	-1.59667900	-1.82976500	0.87024700
C	-4.12502900	-0.80581600	1.11781300
C	-2.65982600	-2.69866600	1.07406800
C	-3.94357100	-2.17892600	1.20061700
F	0.14008400	2.48977600	-1.84278700
F	-0.00397000	5.12907100	-1.55327900
F	-0.80808500	6.24005800	0.81673400
F	-1.46448000	4.61026600	2.91910800
F	-1.30829800	1.93546200	2.65510400
F	2.21864700	1.52465400	-0.11100400
F	4.44740500	1.21925800	1.34960100
F	4.39191700	-0.23288900	3.66448000
F	2.03293700	-1.32948700	4.51481400
F	-0.19624700	-1.01452900	3.11389100
F	-3.31460300	1.34083400	0.80447000
F	-5.36362200	-0.28939800	1.19480000
F	-4.99259900	-2.99516800	1.37801500
F	-2.46414700	-4.02800200	1.13452500
F	-0.38080800	-2.40564200	0.71005300
C	2.94893600	-0.51934700	-2.51605100
C	2.52835200	0.58828500	-3.29119600
C	4.07442200	-0.37559500	-1.67039500
C	3.16698600	1.81037800	-3.18432200
H	1.65817100	0.48474300	-3.92974900
C	4.75558700	0.83100100	-1.60051900
H	4.45362600	-1.22831000	-1.11942800
C	4.27416000	1.90606500	-2.33994300

H	2.83210200	2.68316700	-3.73395900
H	5.63085000	0.95573600	-0.97473600
F	4.90963000	3.08539000	-2.24850900

TS4

E(SMD/B3LYP-D3/6-31G(d)) = -1008.877372 au
H(SMD/B3LYP-D3/6-31G(d)) = -1008.565865 au
G(SMD/B3LYP-D3/6-31G(d)) = -1008.636599 au
E(SMD/ B3LYP-D3/def2-TZVP//SMD/B3LYP-D3/6-31G(d)) = -1009.270764 au

C	2.45990000	-0.06101100	-0.55763800
C	2.65900600	-0.08072800	0.84099900
C	3.93779300	-0.11705700	1.38972500
C	5.03222700	-0.15176600	0.53171300
C	4.88702600	-0.16768900	-0.85050000
C	3.60077200	-0.13044800	-1.38441900
C	0.03501300	0.83737900	-0.79481400
C	0.09216700	1.75796600	0.27389400
C	-1.00779400	2.54222400	0.61449700
C	-2.18009500	2.40941700	-0.12012400
C	-2.27759700	1.54072200	-1.20125400
C	-1.17161300	0.76535000	-1.52931200
H	1.01085400	1.88397700	0.83485400
H	-0.96428700	3.25234700	1.43430300
H	-3.20910100	1.46654800	-1.75271800
H	-1.24311100	0.06588800	-2.35735200
H	3.47427600	-0.14095400	-2.46383100
H	1.79966100	-0.10328900	1.50315900
H	4.09547700	-0.13561800	2.46341200
H	5.76661100	-0.20761000	-1.48513800
F	6.27911900	-0.18682800	1.06153200
F	-3.25679000	3.16037500	0.21888600
C	1.12725500	-0.05918100	-1.16927500
H	1.12631300	-0.33098500	-2.22357800
C	0.43962900	-2.05070000	-0.55666500
C	-0.74431700	-2.12907900	-0.18492300
C	-2.07549500	-1.82081500	0.17029300
C	-2.35276500	-1.13590900	1.37863800
C	-3.15290700	-2.15570700	-0.68592300
C	-3.65745300	-0.77778700	1.69836800
H	-1.53065500	-0.87045100	2.03608800
C	-4.45322900	-1.79404600	-0.35156000
H	-2.94840900	-2.68429600	-1.61239400
C	-4.71326700	-1.10157400	0.83737000
H	-3.85457600	-0.23735500	2.62042700
H	-5.27036200	-2.04924600	-1.02117400
H	-5.73050600	-0.81669100	1.09115400

H	1.37132700	-2.57155300	-0.68553700

TS5

E(SMD/B3LYP-D3/6-31G(d)) = -1008.725025 au
H(SMD/B3LYP-D3/6-31G(d)) = -1008.411982 au
G(SMD/B3LYP-D3/6-31G(d)) = -1008.479279 au
E(SMD/ B3LYP-D3/def2-TZVP//SMD/B3LYP-D3/6-31G(d)) = -1009.110136 au

C	1.10190600	-1.32024400	-0.18946400
C	2.14370500	-1.65046400	0.70826600
C	2.66893700	-2.93261700	0.74127600
C	2.14373300	-3.89057400	-0.12688500
C	1.09840000	-3.61775100	-1.00553800
C	0.56925300	-2.33483000	-1.01772200
C	1.19417100	1.28509200	-0.22493500
C	2.59224200	1.42452600	-0.07295400
C	3.18112100	2.68213100	-0.05818000
C	2.36577100	3.80291700	-0.19171600
C	0.98349900	3.71260700	-0.36054600
C	0.40893300	2.45366900	-0.38365200
H	3.22898100	0.55092700	-0.01682900
H	4.25404900	2.80713000	0.03902000
H	0.39457200	4.61626600	-0.47329200
H	-0.66620700	2.35549200	-0.50376500
H	-0.25299700	-2.10022600	-1.68771100
H	2.51251400	-0.91156100	1.41099000
H	3.46073800	-3.20853100	1.42900100
H	0.71602800	-4.40248200	-1.64911800
F	2.65438200	-5.13126300	-0.09738200
F	2.93255700	5.01881900	-0.17424700
C	0.51075800	0.00240400	-0.28850300
H	-0.39458900	0.02705700	-0.88754300
C	-0.53819700	0.05526800	1.61614000
C	-1.71727100	0.07200900	1.25868900
C	-2.98739100	0.07184700	0.65293600
C	-3.63984600	1.29472300	0.36754200
C	-3.60184100	-1.15533200	0.30793900
C	-4.88199000	1.28187300	-0.25389600
H	-3.16262900	2.23055000	0.64092900
C	-4.84381300	-1.15057200	-0.31453700
H	-3.09263400	-2.08738400	0.53216800
C	-5.48282400	0.06347500	-0.59444300
H	-5.38682500	2.21755500	-0.47408300
H	-5.31875800	-2.08966500	-0.58161500
H	-6.45493400	0.06036000	-1.07904000
H	0.26471800	0.04556800	2.32662800

TS7

E(SMD/B3LYP-D3/6-31G(d)) = -3736.72851 au
H(SMD/B3LYP-D3/6-31G(d)) = -3736.126696 au
G(SMD/B3LYP-D3/6-31G(d)) = -3736.281549 au
E(SMD/ B3LYP-D3/def2-TZVP//SMD/B3LYP-D3/6-31G(d)) = -3738.295038 au

B	-1.88367800	-0.40215800	0.36714600
C	-3.22941100	-1.03886400	1.05322400
C	-4.24856500	-1.70066300	0.36911300
C	-3.48185200	-0.82437600	2.41054400
C	-5.41339200	-2.15806000	0.98148900
C	-4.62870300	-1.26673700	3.06287200
C	-5.60441400	-1.94393600	2.34068300
C	-1.42963500	-1.09527900	-1.05070900
C	-0.95100700	-0.41714200	-2.17018300
C	-1.37050600	-2.48715300	-1.15402700
C	-0.49861700	-1.05351200	-3.32278800
C	-0.94623700	-3.16716700	-2.29074400
C	-0.51944200	-2.43880400	-3.39487100
C	-2.16374600	1.21271900	0.29902500
C	-3.06841700	1.69894200	-0.64559900
C	-1.65173400	2.17988700	1.16228500
C	-3.40350400	3.03953900	-0.78610700
C	-1.95452100	3.53654000	1.05522000
C	-2.83996100	3.97009800	0.07911600
F	-2.60131400	-0.12654000	3.16274900
F	-4.80815200	-1.03326400	4.37519700
F	-6.72033600	-2.37703600	2.94681900
F	-6.35439900	-2.80230600	0.26878200
F	-4.15492400	-1.93779600	-0.95797700
F	-1.74639200	-3.25740500	-0.10603200
F	-0.92042200	-4.51007700	-2.32373100
F	-0.07198800	-3.06616900	-4.49287200
F	-0.00853900	-0.33624300	-4.35154400
F	-0.87724800	0.93216000	-2.19369700
F	-3.64867500	0.83546600	-1.51271400
F	-4.25271300	3.44562800	-1.74697500
F	-3.12492700	5.27699900	-0.04776900
F	-1.38223100	4.43334300	1.88002900
F	-0.79958200	1.85495200	2.16232400
O	-0.73232600	-0.76494900	1.33277400
C	0.46685700	-0.33363000	1.15313400
O	0.72226400	0.50852300	0.24970100
C	1.50045500	-0.88815400	2.04713900
C	1.36524500	-2.19669400	2.53647900
C	2.64023700	-0.13286200	2.35652200
C	2.37674700	-2.75898600	3.30849000

H	0.48137000	-2.77322500	2.28471900
C	3.64973700	-0.67845100	3.14231400
H	2.73797900	0.88108700	1.98633100
C	3.49920800	-1.98596400	3.59407300
H	2.31171400	-3.77420900	3.68483300
H	4.54327300	-0.11787300	3.39233000
F	4.48290100	-2.52722200	4.34057500
C	3.53070300	-0.76482200	-1.73205000
C	3.20048400	-1.76311700	-0.80541900
C	2.65270000	-2.97441800	-1.22207700
C	2.44745600	-3.17458400	-2.58128000
C	2.77146500	-2.21494900	-3.53183100
C	3.31090400	-1.00489900	-3.09368200
C	5.04789000	0.60955000	-0.13917500
C	5.12316100	1.67985400	0.76157100
C	6.08694900	1.71012500	1.77043100
C	6.98083900	0.65150800	1.86467800
C	6.94838800	-0.42065600	0.98202900
C	5.97987600	-0.43108900	-0.02145200
H	4.42124200	2.50405200	0.68378900
H	6.14657700	2.53320600	2.47518500
H	7.66713300	-1.22710000	1.08495100
H	5.95052700	-1.26694900	-0.71250600
H	3.54957900	-0.23468000	-3.82238600
H	3.37806500	-1.60038200	0.25049700
H	2.37954200	-3.74919100	-0.51338800
H	2.57489600	-2.40660300	-4.58065900
F	1.88490700	-4.33671700	-2.98870400
F	7.91238000	0.66486300	2.84695100
C	4.02975400	0.61010500	-1.28419700
C	2.82360900	1.45946500	-0.97316700
C	2.20689800	2.51861400	-1.20950600
C	1.31326500	3.58898500	-1.33593200
C	1.32353400	4.64001900	-0.38606300
C	0.36934100	3.59177100	-2.39266900
C	0.39989000	5.66977500	-0.49471200
H	2.04396100	4.61896900	0.42539800
C	-0.54248000	4.63322600	-2.48976100
H	0.35720700	2.76735600	-3.09570300
C	-0.52611200	5.66834000	-1.54609000
H	0.38910900	6.46998700	0.23826400
H	-1.27785200	4.63719400	-3.28825700
H	-1.25290700	6.47110000	-1.61992900
H	1.87146000	0.92764800	-0.23090500

H	4.51089400	1.08354300	-2.14883200

I3 (Toluene)

E(SMD/B3LYP-D3/6-31G(d)) = -700.2943884 au
H(SMD/B3LYP-D3/6-31G(d)) = -700.09715 au
G(SMD/B3LYP-D3/6-31G(d)) = -700.147704 au
E(SMD/ B3LYP-D3/def2-TZVP//SMD/B3LYP-D3/6-31G(d)) = -700.5470858 au

B	-0.00056200	0.00017500	-0.00067400
C	0.31722400	-1.53435000	-0.00015600
C	1.41968700	-2.07210400	0.68101500
C	1.71231300	-3.43007000	0.70005400
C	0.89293000	-4.31287100	0.00096000
C	-0.20973700	-3.82879100	-0.69865200
C	-0.48054300	-2.46630200	-0.68093400
C	1.16990900	1.04233700	-0.00079900
C	1.08654100	2.26500200	0.68261100
C	2.11728000	3.19632200	0.70161400
C	3.29034700	2.92791200	0.00047200
C	3.42001100	1.73226000	-0.70184100
C	2.37472300	0.81737800	-0.68389300
C	-1.48819400	0.49276100	-0.00064900
C	-1.89622000	1.64993600	-0.68155700
C	-3.21161300	2.09653400	-0.69970000
C	-4.18238500	1.38392600	-0.00008400
C	-3.82774600	0.23290200	0.69923500
C	-2.50541500	-0.19270700	0.68058700
F	2.24297100	-1.27009700	1.37786800
F	2.76858300	-3.89499400	1.37756500
F	1.16338900	-5.61788300	0.00156900
F	-0.99419200	-4.67566700	-1.37563700
F	-1.55455000	-2.05798700	-1.37827500
F	-0.01828800	2.57706600	1.38167200
F	1.99421000	4.34254300	1.38124900
F	4.28624500	3.81358200	0.00111400
F	4.54414600	1.47660900	-1.38139100
F	2.55605500	-0.31586500	-1.38344700
F	-2.22298200	-1.30657800	1.37761800
F	-4.75860700	-0.44893000	1.37709400
F	-5.44771000	1.80253400	0.00030600
F	-3.55249600	3.19928700	-1.37681300
F	-1.00587000	2.37623400	-1.37897500

4d (Toluene)

E(SMD/B3LYP-D3/6-31G(d)) = -385.8105991 au
H(SMD/B3LYP-D3/6-31G(d)) = -385.658135 au
G(SMD/B3LYP-D3/6-31G(d)) = -385.7022 au
E(SMD/ B3LYP-D3/def2-TZVP//SMD/B3LYP-D3/6-31G(d)) = -385.9452716 au

C	-2.32138400	-2.02564400	-0.08205400

C	-1.31523300	-1.35260300	-0.04370900
C	-0.07181400	-0.64779800	-0.00388200
C	0.03584000	0.77175700	-0.04308700
C	1.09688700	-1.43362200	0.04873200
C	1.33159600	1.32225400	-0.04741000
C	2.36106400	-0.85459600	0.05903500
H	0.99076100	-2.51390100	0.07600900
C	2.47794500	0.53564300	0.00760100
H	1.43225400	2.40408000	-0.08742400
H	3.24569800	-1.48367600	0.09988900
H	3.45747400	1.00576500	0.00819400
C	-1.08390100	1.72770300	-0.08909000
H	-0.76884400	2.74065500	-0.34139400
C	-2.38621400	1.53810000	0.16349800
H	-2.80629700	0.58143100	0.44764400
H	-3.07650100	2.37530300	0.09722400
H	-3.22325100	-2.59682400	-0.11795900

4d (THF)
E(SMD/B3LYP-D3/6-31G(d)) = -385.810543 au
H(SMD/B3LYP-D3/6-31G(d)) = -385.65814 au
G(SMD/B3LYP-D3/6-31G(d)) = -385.701893 au
E(SMD/ B3LYP-D3/def2-TZVP//SMD/B3LYP-D3/6-31G(d)) = -385.945229 au

C	-2.32809900	-2.01622200	-0.10301100
C	-1.31598300	-1.35194500	-0.05426500
C	-0.07064000	-0.64997900	-0.00494600
C	0.03611100	0.76927100	-0.05450200
C	1.09828100	-1.43510000	0.06076200
C	1.33049800	1.32293500	-0.05937400
C	2.36174500	-0.85330800	0.07370700
H	0.99499100	-2.51558600	0.09496200
C	2.47741100	0.53710600	0.00964300
H	1.42929900	2.40446300	-0.10980200
H	3.24701900	-1.48072300	0.12465600
H	3.45660000	1.00790900	0.01062400
C	-1.08807400	1.72044900	-0.11357700
H	-0.78840700	2.71867900	-0.43391400
C	-2.37675000	1.53779400	0.20689300
H	-2.77446000	0.59651900	0.56708200
H	-3.07764500	2.36481000	0.12383500
H	-3.23440200	-2.58206600	-0.14542300

TS3a (Toluene)
E(SMD/B3LYP-D3/6-31G(d)) = -1086.112409 au
H(SMD/B3LYP-D3/6-31G(d)) = -1085.762758 au
G(SMD/B3LYP-D3/6-31G(d)) = -1085.832379 au
E(SMD/ B3LYP-D3/def2-TZVP//SMD/B3LYP-D3/6-31G(d)) = -1086.473576 au

C	2.46647900	-0.27400500	-0.27040900
C	3.11378700	0.96718200	-0.10623200
C	4.49981100	1.04664000	-0.01140800
C	5.24497000	-0.12465200	-0.08012900
C	4.64941100	-1.37169400	-0.26696900

C	3.26852600	-1.43332300	-0.37041700
C	0.06492200	0.72097700	-0.46586300
C	0.11051200	1.76523800	0.48489500
C	-0.81109500	2.79873300	0.45117900
C	-1.80590100	2.77915500	-0.52863200
C	-1.90444500	1.76124300	-1.46894400
C	-0.97317000	0.72890500	-1.42158000
H	0.85246000	1.74726500	1.27593800
H	-0.79492200	3.60442500	1.17709600
H	-2.70022000	1.78069800	-2.20524300
H	-1.03847800	-0.07743500	-2.14580400
H	2.79883300	-2.39936100	-0.53413900
H	2.54344800	1.88674200	-0.09973800
H	5.00593600	1.99960800	0.09808600
H	5.26891000	-2.25908900	-0.33709800
F	6.58062700	-0.05428600	0.01766600
F	-2.70587800	3.77344000	-0.54992000
C	1.00585900	-0.40180200	-0.47604500
H	0.78185700	-1.12006600	-1.26070500
C	-2.04106500	0.35344700	3.09105300
C	-2.29816800	-0.25457500	2.07655600
C	-2.71856900	-0.92936400	0.89459100
C	-1.94328100	-1.93250000	0.21890800
C	-4.00082400	-0.62555100	0.41045200
C	-2.53617800	-2.61151600	-0.87912600
C	-4.53107100	-1.28604100	-0.69581100
H	-4.58335000	0.13520400	0.91908900
C	-3.80057300	-2.29031000	-1.34282200
H	-1.96324200	-3.38781700	-1.37868600
H	-5.52515300	-1.02428000	-1.04637300
H	-4.22187100	-2.81650600	-2.19334500
C	-0.58378400	-2.27176100	0.50024500
H	-0.23565300	-3.17663900	0.00182400
C	0.40250000	-1.52788600	1.14536300
H	0.14139200	-0.72245900	1.81899400
H	1.32518400	-2.04633000	1.38004100
H	-1.79979300	0.88439300	3.98723400

TS3b (Toluene)

E(SMD/B3LYP-D3/6-31G(d)) = -1086.114004 au
H(SMD/B3LYP-D3/6-31G(d)) = -1085.763726 au
G(SMD/B3LYP-D3/6-31G(d)) = -1085.837471 au
E(SMD/ B3LYP-D3/def2-TZVP//SMD/B3LYP-D3/6-31G(d)) = -1086.481343 au

C	-0.13041300	1.66901900	-0.19045300
C	-0.64507600	2.65946200	0.67874000
C	-0.07106100	3.91895600	0.73787400

C	1.03095700	4.19059300	-0.07545100
C	1.58574700	3.23861600	-0.92768200
C	1.01164400	1.97586700	-0.96744500
C	-2.06463400	-0.07913300	-0.23705300
C	-3.13752900	0.83923100	-0.19388000
C	-4.45285600	0.39423500	-0.20993500
C	-4.69549800	-0.97617800	-0.25941700
C	-3.66586300	-1.91805700	-0.31415800
C	-2.35877900	-1.46322600	-0.31138200
H	-2.94778600	1.90536300	-0.21137300
H	-5.28799700	1.08624200	-0.20251200
H	-3.90698100	-2.97444400	-0.36014300
H	-1.53980000	-2.17759900	-0.33466700
H	1.43396700	1.21793600	-1.62016200
H	-1.46999600	2.42483100	1.34183000
H	-0.44246300	4.68655700	1.40804300
H	2.44891200	3.49635000	-1.53149300
F	1.58628100	5.40688100	-0.01864100
F	-5.96221200	-1.40725100	-0.26761200
C	-0.66598400	0.32398500	-0.28295600
H	-0.04658700	-0.34804800	-0.86932800
C	0.02205300	-0.50875900	1.61252900
C	1.08594900	-1.02904300	1.26501900
C	2.27391300	-1.41882400	0.62941100
C	2.41069900	-2.65573700	-0.07888300
C	3.32962200	-0.47035900	0.62840100
C	3.60257900	-2.85389200	-0.79110200
C	4.50351300	-0.71979500	-0.06449200
H	3.19231100	0.45763700	1.17381200
C	4.63444000	-1.91635100	-0.77940200
H	3.72583600	-3.77605400	-1.35193500
H	5.30752100	0.00917900	-0.05800100
H	5.54743200	-2.12118200	-1.33095800
H	-0.75003000	-0.26790600	2.31526800
C	1.39045000	-3.71212800	-0.13964000
H	1.48090400	-4.36525700	-1.00655200
C	0.43443200	-3.98747400	0.75966400
H	0.31030100	-3.43345300	1.68406200
H	-0.24198900	-4.82287300	0.60054200

TS3a (THF)
E(SMD/B3LYP-D3/6-31G(d)) = -1086.132866 au
H(SMD/B3LYP-D3/6-31G(d)) = -1085.782662 au
G(SMD/B3LYP-D3/6-31G(d)) = -1085.854457 au
E(SMD/ B3LYP-D3/def2-TZVP//SMD/B3LYP-D3/6-31G(d)) = -1086.4936 au

C	-2.46859900	0.12733600	-0.43398100

C	-2.97256500	-0.95895900	0.30956600
C	-4.33798800	-1.09492500	0.53681100
C	-5.20194400	-0.13626900	0.01799400
C	-4.75039500	0.94550300	-0.73549600
C	-3.38699000	1.06583300	-0.95806500
C	0.01072000	-0.67976800	-0.56822000
C	0.19636500	-1.42214500	0.61797200
C	1.22705300	-2.34485700	0.72969600
C	2.09049900	-2.51422700	-0.35040800
C	1.96313600	-1.78794300	-1.52926500
C	0.93104400	-0.86148500	-1.62302400
H	-0.43348100	-1.23729800	1.48009800
H	1.39014000	-2.90770600	1.64228000
H	2.67041800	-1.93919800	-2.33729400
H	0.82272800	-0.27727500	-2.53193700
H	-3.01620100	1.90363500	-1.54246400
H	-2.31018300	-1.73018200	0.67938700
H	-4.73890700	-1.93440800	1.09463600
H	-5.46193700	1.66055200	-1.13429600
F	-6.52262800	-0.26480100	0.23849200
F	3.09767500	-3.40004000	-0.23970800
C	-1.04374500	0.32299000	-0.75095600
H	-0.91811100	0.89053100	-1.66899800
C	1.36953200	0.71759300	3.38727300
C	1.82961000	0.90071200	2.28232900
C	2.45758100	1.09336500	1.01575100
C	1.88123700	1.86634300	-0.04653500
C	3.73596600	0.54638400	0.83838700
C	2.66480900	2.11029900	-1.20311300
C	4.45434700	0.75758300	-0.33941900
H	4.16913900	-0.04263500	1.63974500
C	3.92637900	1.55577000	-1.35907100
H	2.24089800	2.71921300	-1.99683300
H	5.43849300	0.31201900	-0.44980600
H	4.49477500	1.73776100	-2.26551000
C	0.53410500	2.35127200	-0.08902800
H	0.33925700	3.08331100	-0.87124300
C	-0.58221500	1.85610000	0.57563900
H	-0.48267300	1.24767700	1.46534400
H	-1.49884700	2.43036900	0.49792400
H	0.95142200	0.56816400	4.36089100

TS3b (THF)
E(SMD/B3LYP-D3/6-31G(d)) = -1086.130227 au
H(SMD/B3LYP-D3/6-31G(d)) = -1085.780458 au
G(SMD/B3LYP-D3/6-31G(d)) = -1085.853442 au

E(SMD/ B3LYP-D3/def2-TZVP//SMD/B3LYP-D3/6-31G(d)) = -1086.49878 au

C	0.22283600	-1.72741300	-0.18689300
C	0.83577800	-2.65562100	0.68749200
C	0.37008600	-3.95795800	0.77532000
C	-0.72025900	-4.33080800	-0.01175800
C	-1.37189500	-3.44375200	-0.86473100
C	-0.90558600	-2.13831600	-0.93506900
C	2.00341600	0.17276300	-0.27160600
C	3.14656300	-0.65614800	-0.24280900
C	4.42231800	-0.10592000	-0.26614600
C	4.55058000	1.27908000	-0.30837600
C	3.44933900	2.13534600	-0.35036100
C	2.18292300	1.57616700	-0.34114400
H	3.04434400	-1.73375300	-0.26280300
H	5.31010800	-0.72894800	-0.26713000
H	3.60313100	3.20821500	-0.39053900
H	1.31005200	2.22327900	-0.35426300
H	-1.40318600	-1.42595600	-1.58629000
H	1.65257000	-2.34145900	1.32810400
H	0.81927700	-4.68186400	1.44645000
H	-2.22403200	-3.78081100	-1.44485000
F	-1.16983800	-5.59263900	0.07224300
F	5.78177900	1.81382400	-0.32258800
C	0.64249000	-0.34524000	-0.30878700
H	-0.03392900	0.26841900	-0.89537600
C	-0.09864100	0.42393800	1.60079200
C	-1.16575500	0.92200600	1.23578200
C	-2.34711300	1.30994600	0.58280700
C	-2.51569100	2.59799800	-0.01680700
C	-3.35202800	0.31861200	0.44782300
C	-3.68354700	2.80720600	-0.76521800
C	-4.50447700	0.57511300	-0.27963600
H	-3.19342200	-0.64869600	0.91395700
C	-4.66487500	1.82448200	-0.89027800
H	-3.82742500	3.76993500	-1.24772400
H	-5.26822900	-0.18970400	-0.37974500
H	-5.56050900	2.03496600	-1.46770600
H	0.67006900	0.16289800	2.30050700
C	-1.55076000	3.70351500	0.07174700
H	-1.63835700	4.43036800	-0.73508300
C	-0.64971000	3.93647700	1.03759000
H	-0.53488600	3.30076600	1.90926400
H	-0.00899900	4.81285300	0.98793000

4a

E(SMD/B3LYP-D3/6-31G(d)) = -385.8145761 au

H(SMD/B3LYP-D3/6-31G(d)) = -385.662144 au
G(SMD/B3LYP-D3/6-31G(d)) = -385.706222 au
E(SMD/ B3LYP-D3/def2-TZVP//SMD/B3LYP-D3/6-31G(d)) = -385.9618356 au

C	4.11919900	-0.24047100	-0.00051200
C	2.91228000	-0.13438300	-0.00037100
C	1.48885600	-0.01226500	0.00002000
C	0.67069700	-1.16145100	0.00048700
C	0.87485900	1.25372100	-0.00001000
C	-0.71198100	-1.04434000	0.00076500
H	1.13328500	-2.14392000	0.00066500
C	-0.51173300	1.35958300	0.00021300
H	1.49104000	2.14781600	-0.00017300
C	-1.33612000	0.21915500	0.00049200
H	-1.31397900	-1.94788500	0.00137600
H	-0.96971600	2.34575400	0.00019300
H	5.18436400	-0.33461900	-0.00086700
C	-2.79651800	0.39715500	0.00016200
H	-3.12611600	1.43614000	0.00075600
C	-3.73004100	-0.56451500	-0.00104000
H	-3.48904100	-1.62482200	-0.00197300
H	-4.78682200	-0.31160000	-0.00121700

I11-C
E(SMD/B3LYP-D3/6-31G(d)) = -1086.161312 au
H(SMD/B3LYP-D3/6-31G(d)) = -1085.809517 au
G(SMD/B3LYP-D3/6-31G(d)) = -1085.884856 au
E(SMD/ B3LYP-D3/def2-TZVP//SMD/B3LYP-D3/6-31G(d)) = -1086.573804 au

C	0.80594800	1.13945100	0.59724100
C	0.64964600	1.14033100	-0.79519200
C	-0.11516900	2.11963600	-1.42854900
C	-0.71866600	3.09833000	-0.64790100
C	-0.58222800	3.13325200	0.73431200
C	0.18493600	2.14397500	1.35030400
C	2.86798000	-0.37840000	0.62987400
C	2.93010900	-1.41402500	-0.31070900
C	4.12927400	-1.72935200	-0.95269000
C	5.26234100	-0.99076200	-0.63900100
C	5.23965100	0.04415700	0.28942400
C	4.03446600	0.34292000	0.92196800
H	2.03830500	-1.98657300	-0.54910000
H	4.19210100	-2.53139200	-1.68097100
H	6.14993300	0.59299300	0.50800800
H	3.99975000	1.15113100	1.64784600
H	0.29800700	2.15418800	2.43134400
H	1.13149300	0.37426800	-1.39506800
H	-0.24330900	2.13434300	-2.50592600

H	-1.06449100	3.92025100	1.30485000
F	-1.46230400	4.05218600	-1.25546000
F	6.42987900	-1.29058700	-1.25386600
C	1.56667600	0.02432100	1.31286000
H	1.81887000	0.38014600	2.31927000
C	-2.10828800	-1.82229800	-0.66800700
C	-1.81533600	-1.29177300	0.63440400
C	-3.39653300	-1.79602600	-1.13811100
C	-2.88328700	-0.73709900	1.42256600
C	-4.45350700	-1.25107700	-0.35629200
H	-3.62332400	-2.19451000	-2.12213300
C	-4.16194200	-0.71888400	0.93140000
H	-2.65279400	-0.32795700	2.40048600
H	-4.95362700	-0.28818600	1.53283800
C	-0.54458200	-1.26998900	1.10007300
C	0.66387200	-1.19109900	1.54725900
H	1.07253200	-2.02310700	2.12481500
H	-1.29992600	-2.23348800	-1.26333900
C	-5.79422700	-1.26319600	-0.91956500
H	-5.87438200	-1.66801900	-1.92596200
C	-6.91178600	-0.83219000	-0.30449800
H	-6.92262600	-0.42221000	0.70134600
H	-7.87077400	-0.88748600	-0.81139500

I11-R

E(SMD/B3LYP-D3/6-31G(d)) = -1086.322474 au
H(SMD/B3LYP-D3/6-31G(d)) = -1085.972924 au
G(SMD/B3LYP-D3/6-31G(d)) = -1086.051091 au
E(SMD/ B3LYP-D3/def2-TZVP//SMD/B3LYP-D3/6-31G(d)) = -1086.74251 au

C	0.75659400	1.16308900	0.58853000
C	0.50027000	0.99055800	-0.77813100
C	-0.36962800	1.84100400	-1.46094300
C	-0.98091600	2.87011800	-0.75605500
C	-0.75011900	3.07865300	0.59741800
C	0.12247300	2.21538900	1.26165000
C	2.87890500	-0.24692200	0.61532300
C	2.91727500	-1.41476100	-0.15766100
C	4.06187000	-1.76750500	-0.87661500
C	5.16954900	-0.93370300	-0.80921000
C	5.17342700	0.23273300	-0.05268400
C	4.02078600	0.56685300	0.65687500
H	2.04520100	-2.06053000	-0.19937000
H	4.10149900	-2.67068600	-1.47719500
H	6.06246800	0.85470300	-0.02471000
H	4.00738000	1.47787700	1.25018400
H	0.30396600	2.35947000	2.32399600

H	0.98125700	0.18207100	-1.32003800
H	-0.57777400	1.71397400	-2.51850200
H	-1.24526100	3.89704400	1.11020900
F	-1.82771700	3.70186900	-1.41248900
F	6.28707100	-1.26911600	-1.50018100
C	1.62507100	0.18338900	1.37179500
H	1.96325300	0.69919100	2.28059500
C	-2.03350400	-1.84424200	-0.23030800
C	-1.70718800	-1.20447800	1.01507100
C	-3.30898700	-1.78788500	-0.74307800
C	-2.76635500	-0.52262900	1.70165400
C	-4.35409200	-1.10525400	-0.06553600
H	-3.51329500	-2.27564300	-1.69184000
C	-4.03662200	-0.48022400	1.16586200
H	-2.54685300	-0.02671600	2.64227700
H	-4.82047100	0.05283500	1.69965400
C	-0.43645200	-1.25285400	1.52522700
C	0.80577600	-1.02382400	1.85268600
H	1.34916000	-1.71027100	2.50838300
H	-1.24758200	-2.36584600	-0.76872300
C	-5.71721800	-1.01878700	-0.57727000
H	-6.40554000	-0.45765900	0.05552800
C	-6.19839200	-1.54474300	-1.71947500
H	-5.58621200	-2.11804000	-2.41131400
H	-7.24052400	-1.41040700	-1.99585400

I12-C

E(SMD/B3LYP-D3/6-31G(d)) = -1086.15131 au
H(SMD/B3LYP-D3/6-31G(d)) = -1085.79902 au
G(SMD/B3LYP-D3/6-31G(d)) = -1085.872986 au
E(SMD/ B3LYP-D3/def2-TZVP//SMD/B3LYP-D3/6-31G(d)) = -1086.564291 au

C	-0.44318300	-0.45112800	0.45253000
C	-0.48048500	-1.28349300	-0.67724400
C	0.37602600	-2.37666100	-0.79419900
C	1.27476700	-2.62933500	0.23637600
C	1.33798000	-1.83649200	1.37524700
C	0.47389600	-0.74569300	1.47180000
C	-2.78630200	0.52469300	0.24264400
C	-3.31425300	0.59718200	-1.05342000
C	-4.65612000	0.30380800	-1.30179100
C	-5.46478300	-0.06210700	-0.23390600
C	-4.98078200	-0.14197200	1.06640300
C	-3.63824500	0.15605500	1.29455500
H	-2.68504100	0.88529900	-1.88976200
H	-5.07393000	0.35875100	-2.30170700
H	-5.64655600	-0.42646400	1.87465200

H	-3.24603600	0.09556000	2.30642800
H	0.52062300	-0.11030700	2.35247100
H	-1.18597000	-1.07939100	-1.47694600
H	0.35495500	-3.02703800	-1.66256700
H	2.04991900	-2.07203500	2.15936300
F	2.11291500	-3.68506600	0.12717600
F	-6.76777800	-0.34429600	-0.46615700
C	-1.31902200	0.78671800	0.55820300
H	-1.26823200	1.14535800	1.59100700
C	2.01905700	0.94002600	-1.07799300
C	1.78282700	1.93009000	-0.06952100
C	3.24789900	0.33460800	-1.18470500
C	2.87006500	2.32120800	0.77843600
C	4.30009500	0.70514100	-0.30673000
H	3.42523000	-0.42933900	-1.93314500
C	4.09966900	1.71697000	0.66814800
H	2.70154800	3.08289600	1.53379000
H	4.91812600	1.99542900	1.32238400
C	0.51548700	2.43723900	0.19880100
H	0.44241600	3.17672600	0.99591900
C	-0.75327900	1.96690200	-0.36609300
H	-0.64939200	1.58782800	-1.38409800
H	-1.49114400	2.77325700	-0.34663000
H	1.22453300	0.65565900	-1.75566700
C	5.55839300	0.06519600	-0.40990300
C	6.63350700	-0.48752300	-0.49492100
H	7.58342100	-0.97652100	-0.57097900

I12-R

E(SMD/B3LYP-D3/6-31G(d)) = -1086.319984 au
H(SMD/B3LYP-D3/6-31G(d)) = -1085.970077 au
G(SMD/B3LYP-D3/6-31G(d)) = -1086.045419 au
E(SMD/ B3LYP-D3/def2-TZVP//SMD/B3LYP-D3/6-31G(d)) = -1086.74095 au

C	0.59990000	0.58049400	0.54146400
C	0.69952800	1.35306000	-0.62473200
C	-0.07937300	2.49626600	-0.80297700
C	-0.96329200	2.86010100	0.20607200
C	-1.08368100	2.13129700	1.38131500
C	-0.29339700	0.99129000	1.53925700
C	2.82808000	-0.59246400	0.27007100
C	3.29665100	-0.97017200	-0.99575000
C	4.63678400	-0.79737300	-1.35363100
C	5.50545600	-0.23982700	-0.42664000
C	5.08359100	0.14873700	0.83975300
C	3.74307200	-0.03193700	1.17561500
H	2.62077200	-1.40845100	-1.72268200

H	5.00606200	-1.08993300	-2.33143200
H	5.79502000	0.57790700	1.53816500
H	3.39857200	0.27193800	2.16117800
H	-0.38847800	0.40335300	2.44815900
H	1.39386000	1.06082600	-1.40733800
H	-0.01045400	3.09943900	-1.70270900
H	-1.78343500	2.45166300	2.14648000
F	-1.72758900	3.96830600	0.03870800
F	6.80832900	-0.07135900	-0.76417400
C	1.36905600	-0.72872100	0.69143900
H	1.36649500	-0.98718600	1.75742700
C	-2.18036000	-0.84678100	-0.85872100
C	-1.95808200	-1.74956700	0.22881000
C	-3.44224700	-0.37244200	-1.15375700
C	-3.10950400	-2.14753800	0.98075700
C	-4.56835100	-0.77314200	-0.39294500
H	-3.58086000	0.32310500	-1.97640300
C	-4.37076000	-1.67619100	0.68152700
H	-2.97652800	-2.83575900	1.81206400
H	-5.22618200	-1.99196500	1.27174100
C	-0.68590800	-2.23071500	0.59069300
H	-0.63424700	-2.87576500	1.46662000
C	0.61873400	-1.88486900	-0.05204600
H	0.48219100	-1.60419400	-1.10156900
H	1.26975200	-2.76826700	-0.03599800
H	-1.33897600	-0.50400300	-1.44988300
C	-5.86635700	-0.27923900	-0.69893400
C	-6.97451400	0.14161600	-0.95927700
H	-7.95038900	0.51346400	-1.18880400

TS8-C

E(SMD/B3LYP-D3/6-31G(d)) = -1086.134967 au
H(SMD/B3LYP-D3/6-31G(d)) = -1085.785243 au
G(SMD/B3LYP-D3/6-31G(d)) = -1085.858893 au
E(SMD/ B3LYP-D3/def2-TZVP//SMD/B3LYP-D3/6-31G(d)) = -1086.548228 au

C	0.37296700	1.65547500	-0.00344600
C	-0.09460300	1.00684500	-1.16845500
C	-1.30882700	1.36125300	-1.74220500
C	-2.06017600	2.36910100	-1.14574300
C	-1.63289200	3.05115500	-0.00727300
C	-0.42088100	2.68739200	0.55670800
C	2.68531400	0.46848300	0.17215300
C	2.45439200	-0.80408800	-0.39706500
C	3.51063900	-1.57865900	-0.85291600
C	4.80618800	-1.07562300	-0.73663700
C	5.08151300	0.15884300	-0.15685600

C	4.01631100	0.91676200	0.31422400
H	1.44916800	-1.20673200	-0.43468200
H	3.35407100	-2.56295100	-1.28094500
H	6.10757500	0.50042100	-0.07406600
H	4.21023000	1.87887600	0.77997000
H	-0.07552200	3.19257600	1.45432400
H	0.50567000	0.24940500	-1.65529200
H	-1.67651800	0.87576400	-2.63952400
H	-2.24928100	3.83790400	0.41379700
F	-3.23921000	2.70602000	-1.69339800
F	5.82921700	-1.82390800	-1.18412800
C	1.62446200	1.34228400	0.66172300
H	1.98140800	2.15496900	1.29218600
C	-1.06365900	-1.98043600	0.85856200
C	-1.07048000	-0.78577900	1.62642300
C	-2.19662300	-2.37186700	0.17371300
C	-2.25640500	-0.01622400	1.69851900
C	-3.37915100	-1.59611300	0.22181300
H	-2.16800500	-3.28088500	-0.41751600
C	-3.37932400	-0.41473200	0.99551900
H	-2.26539800	0.89532100	2.28532000
H	-4.28104900	0.18946500	1.03775700
C	0.10252300	-0.31305900	2.22172000
C	1.15645900	0.26916900	2.49793900
H	1.98198000	0.50912200	3.14024000
H	-0.15558000	-2.57307300	0.80942500
C	-4.60334000	-1.96349100	-0.49547300
H	-5.40996100	-1.23453600	-0.43035100
C	-4.81034700	-3.09073700	-1.19319400
H	-4.06200200	-3.87272700	-1.29352700
H	-5.76120500	-3.26764700	-1.68789500

TS8-R

E(SMD/B3LYP-D3/6-31G(d)) = -1086.284023 au
H(SMD/B3LYP-D3/6-31G(d)) = -1085.937158 au
G(SMD/B3LYP-D3/6-31G(d)) = -1086.013999 au
E(SMD/ B3LYP-D3/def2-TZVP//SMD/B3LYP-D3/6-31G(d)) = -1086.706224 au

C	0.61060200	0.81950000	0.92108300
C	0.55842300	1.72427800	-0.16199500
C	-0.58067000	2.48406500	-0.41725500
C	-1.68295300	2.34312700	0.41785500
C	-1.66964800	1.49348200	1.51834400
C	-0.52603400	0.74147500	1.75993400
C	3.01966300	-0.05232900	0.49088700
C	3.10672600	-0.07633500	-0.91922000
C	4.33766500	-0.10392200	-1.56845800

C	5.49754900	-0.12485600	-0.80076600
C	5.46353500	-0.13581900	0.58874500
C	4.22400200	-0.10793000	1.22398800
H	2.19769500	-0.11093700	-1.51048200
H	4.40870600	-0.12677100	-2.65124500
H	6.39146200	-0.16509800	1.15093900
H	4.18503700	-0.11462700	2.31022900
H	-0.51252900	0.05476600	2.60161900
H	1.42210300	1.85571800	-0.80320200
H	-0.62190000	3.18027300	-1.24902200
H	-2.54816700	1.41455900	2.15015000
F	-2.79903600	3.06787100	0.16066700
F	6.69821000	-0.15144900	-1.42855100
C	1.74139000	-0.05833000	1.20782600
H	1.82513100	-0.33587600	2.25718600
C	-1.86327500	-1.08483600	-1.11277000
C	-1.52450400	-1.79732300	0.06710800
C	-3.16505400	-0.67949500	-1.34508100
C	-2.56210300	-2.11707000	0.97600700
C	-4.20072700	-0.97562800	-0.43023600
H	-3.38591100	-0.11908700	-2.24861800
C	-3.86367300	-1.71041500	0.72595300
H	-2.32437100	-2.66802700	1.88114500
H	-4.64463100	-1.95557700	1.44211600
C	-0.18414700	-2.13281900	0.33644800
C	1.02100300	-2.06900100	0.63429000
H	1.96083400	-2.58604600	0.70101400
H	-1.07864300	-0.83244100	-1.81918200
C	-5.59118900	-0.55340100	-0.62619700
H	-6.28187600	-0.89398000	0.14562500
C	-6.08084800	0.19451700	-1.62768500
H	-5.46302600	0.58233900	-2.43392500
H	-7.13688400	0.44741200	-1.66622200

TS9-C

E(SMD/B3LYP-D3/6-31G(d)) = -1086.137489 au
H(SMD/B3LYP-D3/6-31G(d)) = -1085.786881 au
G(SMD/B3LYP-D3/6-31G(d)) = -1085.858939 au
E(SMD/ B3LYP-D3/def2-TZVP//SMD/B3LYP-D3/6-31G(d)) = -1086.550797 au

C	0.34277200	0.54996600	0.67774600
C	0.30382400	1.60914500	-0.26028600
C	-0.78930900	2.46017100	-0.33008800
C	-1.85527900	2.25772900	0.54539900
C	-1.85370400	1.24946500	1.50452800
C	-0.75940400	0.39927400	1.55670100
C	2.82026500	-0.17337900	0.33429200

C	3.14551400	0.21737800	-0.98173100
C	4.46601500	0.42509500	-1.35810400
C	5.46898100	0.23344000	-0.41013800
C	5.19674200	-0.17883000	0.88947000
C	3.87087000	-0.39706800	1.24834500
H	2.36796900	0.32074700	-1.73086600
H	4.73040300	0.71734000	-2.36867600
H	6.01008600	-0.32900700	1.59128800
H	3.64127200	-0.72315600	2.25859400
H	-0.74577900	-0.40158600	2.28914000
H	1.14330400	1.79333200	-0.91746700
H	-0.82734500	3.27915300	-1.04032100
H	-2.70004700	1.13723300	2.17261600
F	-2.91610400	3.07823500	0.46945700
F	6.74883700	0.43697300	-0.77382500
C	1.44837700	-0.38424300	0.82369000
H	1.42382700	-0.91385700	1.77253900
C	-1.70625200	-1.05580900	-1.22349600
C	-1.51482500	-1.98262000	-0.16634500
C	-2.92680200	-0.43598800	-1.40608400
C	-2.62190200	-2.29843500	0.66342000
C	-4.00991400	-0.72783200	-0.54610600
H	-3.06134400	0.28597300	-2.20437700
C	-3.84628700	-1.68310000	0.48236900
H	-2.49003400	-3.01448500	1.46989300
H	-4.68278300	-1.91612200	1.13260400
C	-0.23355300	-2.52592900	0.16212400
H	-0.21340000	-3.24022100	0.98407300
C	0.98095900	-2.10627300	-0.35436300
H	1.02114200	-1.56688100	-1.29423800
H	1.87065200	-2.67265600	-0.09999800
H	-0.88932300	-0.81591500	-1.89363200
C	-5.25588900	-0.06262800	-0.71838100
C	-6.31232800	0.51141900	-0.86427800
H	-7.24642700	1.01754400	-0.99395600

TS9-R
E(SMD/B3LYP-D3/6-31G(d)) = -1086.287609 au
H(SMD/B3LYP-D3/6-31G(d)) = -1085.940427 au
G(SMD/B3LYP-D3/6-31G(d)) = -1086.015551 au
E(SMD/ B3LYP-D3/def2-TZVP//SMD/B3LYP-D3/6-31G(d)) = -1086.710527 au

C	0.48777100	0.76631500	0.84678100
C	0.43450000	1.76403300	-0.15258100
C	-0.68726900	2.57524800	-0.30758000
C	-1.76571400	2.40331500	0.55252700
C	-1.74845600	1.46399100	1.57690400

C	-0.62263600	0.66084100	1.71871200
C	2.91678500	-0.07645300	0.40433500
C	3.11195500	0.14133400	-0.97780700
C	4.38803700	0.17954300	-1.53448500
C	5.48618900	-0.01576400	-0.70474900
C	5.34678600	-0.26188100	0.65634200
C	4.06447100	-0.29754900	1.19741700
H	2.25650100	0.24912200	-1.63585800
H	4.53866200	0.34351600	-2.59676100
H	6.22842000	-0.42070500	1.26918400
H	3.94467700	-0.48970900	2.26047400
H	-0.60641500	-0.08875700	2.50424300
H	1.27952500	1.92446100	-0.80983900
H	-0.72750000	3.34243700	-1.07430800
H	-2.60498300	1.36668500	2.23546700
F	-2.86270300	3.18364900	0.39325500
F	6.72970000	0.02111500	-1.24215900
C	1.59611100	-0.16565300	1.03805100
H	1.63481900	-0.57374900	2.04517000
C	-1.73909800	-1.05112600	-1.00208100
C	-1.62740300	-1.89145700	0.13474900
C	-2.96068500	-0.53721400	-1.39947100
C	-2.82279700	-2.21150300	0.82564000
C	-4.14292800	-0.85031600	-0.69046100
H	-3.01761600	0.12163000	-2.26073000
C	-4.05034300	-1.70658800	0.42788000
H	-2.77013200	-2.85844200	1.69771400
H	-4.95009800	-1.95940500	0.98108600
C	-0.36683400	-2.38729500	0.63209200
H	-0.42161100	-2.98906400	1.53842400
C	0.88682200	-2.06135200	0.14594200
H	1.00209700	-1.65934100	-0.85554300
H	1.74691400	-2.61358300	0.51342400
H	-0.85081900	-0.77368600	-1.55880600
C	-5.39850800	-0.31070500	-1.09507100
C	-6.46631800	0.15022500	-1.43836900
H	-7.40805400	0.55577300	-1.74151800

1d

E(SMD/B3LYP-D3/6-31G(d)) = -1250.573837 au

H(SMD/B3LYP-D3/6-31G(d)) = -1250.185436 au

G(SMD/B3LYP-D3/6-31G(d)) = -1250.265981 au

E(SMD/ B3LYP-D3/def2-TZVP//SMD/B3LYP-D3/6-31G(d)) = -1251.060232 au

C	4.96787400	-0.79397500	-1.34931300
C	3.58450100	-0.64712300	-1.35980200
C	2.87786700	-0.44693400	-0.16416200

C	3.57470500	-0.39465500	1.05326700
C	4.95942400	-0.54054600	1.07834500
C	5.62767000	-0.73749100	-0.12559600
H	5.53475500	-0.94986300	-2.26119100
H	3.03630600	-0.68574500	-2.29504300
H	3.03362000	-0.23971000	1.97951400
H	5.52119200	-0.50437200	2.00582800
F	6.97081300	-0.87977400	-0.10566300
C	1.39611000	-0.30009400	-0.24657600
O	0.77119400	-0.34502000	-1.29032700
O	0.84349100	-0.12614800	0.97115600
C	-0.61007800	0.06866800	1.10919800
H	-0.68583000	0.12573200	2.19964700
C	-1.39303400	-1.15620300	0.65833200
C	-0.79939600	-2.42874100	0.65896000
C	-2.75309000	-1.06954700	0.35175300
C	-1.53148600	-3.56371200	0.33744900
H	0.25107600	-2.53579700	0.91079500
C	-3.50700200	-2.20356200	0.03149200
H	-3.24907900	-0.10406700	0.35822200
C	-2.89461300	-3.46187800	0.01729200
H	-1.06530600	-4.54458900	0.32844500
H	-4.55830100	-2.08819300	-0.20574400
C	-1.03519800	1.41618600	0.55977500
C	-1.02550700	2.52125100	1.41555500
C	-1.41188200	1.62014800	-0.77939100
C	-1.36961500	3.80096400	0.97240500
H	-0.74192700	2.38823100	2.45742500
C	-1.76117200	2.88301500	-1.23617800
H	-1.42150500	0.78057700	-1.46311500
C	-1.73950700	3.98628300	-0.36577800
H	-1.35074900	4.63045700	1.66961500
H	-2.05224000	3.04255700	-2.27025400
O	-3.52275700	-4.63231500	-0.28601200
O	-2.09505900	5.17919700	-0.91421300
C	-4.90762700	-4.58419900	-0.62182100
H	-5.08506900	-3.97277600	-1.51628400
H	-5.20129400	-5.61591100	-0.82750200
H	-5.51134700	-4.19578600	0.20891100
C	-2.07297900	6.33764300	-0.08157800
H	-1.06698600	6.53075800	0.31279000
H	-2.37507000	7.17002400	-0.72068200
H	-2.78030900	6.24774300	0.75299200

I1′

E(SMD/B3LYP-D3/6-31G(d)) = -3458.894734 au
H(SMD/B3LYP-D3/6-31G(d)) = -3458.320871 au
G(SMD/B3LYP-D3/6-31G(d)) = -3458.463003 au
E(SMD/ B3LYP-D3/def2-TZVP//SMD/B3LYP-D3/6-31G(d)) = -3460.340189 au

C	3.49069000	-3.07927800	-0.86782500
C	2.25229600	-2.47161300	-0.69593400
C	1.63360300	-1.82159200	-1.77454400
C	2.25599500	-1.79655700	-3.03462400
C	3.50506200	-2.38021600	-3.20848700
C	4.09803200	-3.00962200	-2.11780400
H	3.98925800	-3.59187700	-0.05262300
H	1.76556900	-2.51674200	0.26830500
H	1.77686400	-1.29017900	-3.86480800
H	4.02128100	-2.35075500	-4.16165700
F	5.30876400	-3.57555400	-2.27895800
C	0.33957600	-1.13027400	-1.61306300
O	-0.02298000	-0.43429800	-0.62780800
O	-0.44243700	-1.30713000	-2.63999400
C	-1.88753600	-0.86289900	-2.76885800
H	-1.99988900	-1.03291400	-3.84232200
C	-2.07130300	0.61176700	-2.49104000
C	-1.07690800	1.53561200	-2.85300200
C	-3.28243900	1.10055200	-1.99837400
C	-1.27627000	2.89835900	-2.69280200
H	-0.12626700	1.18806800	-3.24430000
C	-3.50795200	2.47199500	-1.85487100
H	-4.07329100	0.41222500	-1.72032900
C	-2.49625600	3.37915700	-2.18920900
H	-0.49577600	3.61027200	-2.94241400
H	-4.45635100	2.81200900	-1.45765700
B	0.70080800	0.29430600	0.61044400
C	0.21617700	-0.52184800	1.94156600
C	0.28938500	0.11048800	3.18557200
C	-0.38326100	-1.77887600	1.96922500
C	-0.22215600	-0.42547500	4.36014700
C	-0.93140900	-2.34708200	3.11790900
C	-0.85416400	-1.66504800	4.32397600
C	-0.00677300	1.77963000	0.62512000
C	0.65081000	3.00780000	0.50271600
C	-1.37885700	1.89604300	0.86790600
C	0.01048400	4.24213500	0.59737700
C	-2.05372800	3.10513500	0.98605300
C	-1.35309000	4.29454500	0.84473000
C	2.30946000	0.33633000	0.36279900
C	3.28262000	-0.07945100	1.27055700

C	2.81473100	0.77054600	-0.86482200
C	4.64626100	-0.09636100	0.97735300
C	4.15606000	0.74783200	-1.21166000
C	5.08721400	0.31093700	-0.27370600
F	-0.46935200	-2.54482000	0.85329400
F	-1.53785400	-3.54508300	3.05917900
F	-1.36806200	-2.19698300	5.44134200
F	-0.11876600	0.23894500	5.52253500
F	0.89793200	1.31340500	3.28539000
F	-2.14059700	0.79035900	1.02986600
F	-3.38164100	3.13317600	1.20105700
F	-1.98724600	5.47206800	0.92926900
F	0.70823300	5.38165600	0.46005400
F	1.98434600	3.08356300	0.30480600
F	2.95264000	-0.54064500	2.49550300
F	5.53279900	-0.52412600	1.89078800
F	6.39029200	0.27996500	-0.57905000
F	4.55995100	1.13054200	-2.43319800
F	1.95336200	1.20043400	-1.81998200
C	-2.73749900	-1.86496700	-2.03527500
C	-2.89222100	-1.83666000	-0.63912200
C	-3.32779700	-2.91240200	-2.74916100
C	-3.60208700	-2.82989600	0.01735300
H	-2.44660800	-1.03486100	-0.06958600
C	-4.06093400	-3.91112500	-2.10598700
H	-3.21519300	-2.95670600	-3.82992900
C	-4.19419800	-3.87654500	-0.71032100
H	-3.69667900	-2.82653800	1.09903000
H	-4.51028600	-4.70363900	-2.69264500
O	-2.58532000	4.72868500	-2.04269100
O	-4.86892800	-4.79681000	0.02489400
C	-3.80188800	5.27392200	-1.52760300
H	-4.02590600	4.88414300	-0.52961800
H	-3.63992800	6.35153100	-1.46548400
H	-4.64453000	5.07001000	-2.20038400
C	-5.47555800	-5.89649700	-0.65444400
H	-4.72927900	-6.50160300	-1.18480000
H	-5.94565800	-6.50272100	0.12247900
H	-6.24229800	-5.55795100	-1.36267300

I2′

E(SMD/B3LYP-D3/6-31G(d)) = -3458.900592 au

H(SMD/B3LYP-D3/6-31G(d)) = -3458.327481 au

G(SMD/B3LYP-D3/6-31G(d)) = -3458.473559 au

E(SMD/ B3LYP-D3/def2-TZVP//SMD/B3LYP-D3/6-31G(d)) = -3460.350294 au

C	3.49069000	-3.07927800	-0.86782500

C	2.25229600	-2.47161300	-0.69593400
C	1.63360300	-1.82159200	-1.77454400
C	2.25599500	-1.79655700	-3.03462400
C	3.50506200	-2.38021600	-3.20848700
C	4.09803200	-3.00962200	-2.11780400
H	3.98925800	-3.59187700	-0.05262300
H	1.76556900	-2.51674200	0.26830500
H	1.77686400	-1.29017900	-3.86480800
H	4.02128100	-2.35075500	-4.16165700
F	5.30876400	-3.57555400	-2.27895800
C	0.33957600	-1.13027400	-1.61306300
O	-0.02298000	-0.43429800	-0.62780800
O	-0.44243700	-1.30713000	-2.63999400
C	-1.88753600	-0.86289900	-2.76885800
H	-1.99988900	-1.03291400	-3.84232200
C	-2.07130300	0.61176700	-2.49104000
C	-1.07690800	1.53561200	-2.85300200
C	-3.28243900	1.10055200	-1.99837400
C	-1.27627000	2.89835900	-2.69280200
H	-0.12626700	1.18806800	-3.24430000
C	-3.50795200	2.47199500	-1.85487100
H	-4.07329100	0.41222500	-1.72032900
C	-2.49625600	3.37915700	-2.18920900
H	-0.49577600	3.61027200	-2.94241400
H	-4.45635100	2.81200900	-1.45765700
B	0.70080800	0.29430600	0.61044400
C	0.21617700	-0.52184800	1.94156600
C	0.28938500	0.11048800	3.18557200
C	-0.38326100	-1.77887600	1.96922500
C	-0.22215600	-0.42547500	4.36014700
C	-0.93140900	-2.34708200	3.11790900
C	-0.85416400	-1.66504800	4.32397600
C	-0.00677300	1.77963000	0.62512000
C	0.65081000	3.00780000	0.50271600
C	-1.37885700	1.89604300	0.86790600
C	0.01048400	4.24213500	0.59737700
C	-2.05372800	3.10513500	0.98605300
C	-1.35309000	4.29454500	0.84473000
C	2.30946000	0.33633000	0.36279900
C	3.28262000	-0.07945100	1.27055700
C	2.81473100	0.77054600	-0.86482200
C	4.64626100	-0.09636100	0.97735300
C	4.15606000	0.74783200	-1.21166000
C	5.08721400	0.31093700	-0.27370600

F	-0.46935200	-2.54482000	0.85329400
F	-1.53785400	-3.54508300	3.05917900
F	-1.36806200	-2.19698300	5.44134200
F	-0.11876600	0.23894500	5.52253500
F	0.89793200	1.31340500	3.28539000
F	-2.14059700	0.79035900	1.02986600
F	-3.38164100	3.13317600	1.20105700
F	-1.98724600	5.47206800	0.92926900
F	0.70823300	5.38165600	0.46005400
F	1.98434600	3.08356300	0.30480600
F	2.95264000	-0.54064500	2.49550300
F	5.53279900	-0.52412600	1.89078800
F	6.39029200	0.27996500	-0.57905000
F	4.55995100	1.13054200	-2.43319800
F	1.95336200	1.20043400	-1.81998200
C	-2.73749900	-1.86496700	-2.03527500
C	-2.89222100	-1.83666000	-0.63912200
C	-3.32779700	-2.91240200	-2.74916100
C	-3.60208700	-2.82989600	0.01735300
H	-2.44660800	-1.03486100	-0.06958600
C	-4.06093400	-3.91112500	-2.10598700
H	-3.21519300	-2.95670600	-3.82992900
C	-4.19419800	-3.87654500	-0.71032100
H	-3.69667900	-2.82653800	1.09903000
H	-4.51028600	-4.70363900	-2.69264500
O	-2.58532000	4.72868500	-2.04269100
O	-4.86892800	-4.79681000	0.02489400
C	-3.80188800	5.27392200	-1.52760300
H	-4.02590600	4.88414300	-0.52961800
H	-3.63992800	6.35153100	-1.46548400
H	-4.64453000	5.07001000	-2.20038400
C	-5.47555800	-5.89649700	-0.65444400
H	-4.72927900	-6.50160300	-1.18480000
H	-5.94565800	-6.50272100	0.12247900
H	-6.24229800	-5.55795100	-1.36267300

$I3_{1d}$

E(SMD/B3LYP-D3/6-31G(d)) = -730.9244384 au
H(SMD/B3LYP-D3/6-31G(d)) = -730.64165 au
G(SMD/B3LYP-D3/6-31G(d)) = -730.69946 au
E(SMD/ B3LYP-D3/def2-TZVP//SMD/B3LYP-D3/6-31G(d)) = -731.1946185 au

C	0.00001400	1.14723800	-0.00001900
H	0.00001400	2.23689800	-0.00008200
C	1.28901700	0.57244900	0.06115700
C	2.39688600	1.43798400	-0.17861500
C	1.57463900	-0.79320900	0.37187400

C	3.69661800	0.97765600	-0.19027000
H	2.20508700	2.48702800	-0.38690200
C	2.86577000	-1.25726600	0.38313400
H	0.77602200	-1.46608400	0.65681300
C	3.94401600	-0.38592600	0.08375100
H	4.51039200	1.66092500	-0.39944500
H	3.09247200	-2.28716900	0.63782500
C	-1.28900700	0.57246200	-0.06110300
C	-2.39686000	1.43801000	0.17864400
C	-1.57463100	-0.79320800	-0.37178900
C	-3.69659900	0.97770100	0.19023400
H	-2.20505000	2.48705100	0.38691800
C	-2.86577300	-1.25724800	-0.38309800
H	-0.77601300	-1.46612600	-0.65660300
C	-3.94401300	-0.38587900	-0.08381900
H	-4.51036400	1.66099600	0.39934000
H	-3.09246900	-2.28717000	-0.63772100
O	-5.15299300	-0.94456100	-0.10972400
O	5.15297500	-0.94462700	0.10950600
C	-6.31710600	-0.13860700	0.15771100
H	-7.16301700	-0.82231100	0.08307600
H	-6.27096600	0.28679100	1.16567700
H	-6.41596200	0.65803300	-0.58692900
C	6.31706700	-0.13852900	-0.15761500
H	7.16307400	-0.82204600	-0.08238100
H	6.27128800	0.28655700	-1.16572900
H	6.41542000	0.65836000	0.58682200

TS1′

E(SMD/B3LYP-D3/6-31G(d)) = -3458.880646 au
H(SMD/B3LYP-D3/6-31G(d)) = -3458.307534 au
G(SMD/B3LYP-D3/6-31G(d)) = -3458.451215 au
E(SMD/ B3LYP-D3/def2-TZVP//SMD/B3LYP-D3/6-31G(d)) = -3460.326059 au

C	-3.52562400	1.46714800	-2.64738900
C	-2.24271900	1.36960800	-2.11609000
C	-1.31057700	0.47904700	-2.66647700
C	-1.67210600	-0.31991800	-3.76108700
C	-2.96256200	-0.25546300	-4.28173000
C	-3.86458300	0.63691500	-3.71041000
H	-4.26242700	2.14889700	-2.23689000
H	-1.95103600	1.99613300	-1.28371400
H	-0.95194200	-1.00913200	-4.18861600
H	-3.27722300	-0.88453000	-5.10752900
F	-5.11630900	0.70086600	-4.20997100
C	0.03857500	0.36424600	-2.04380400
O	0.24519900	0.44586000	-0.83827500

O	0.98172800	0.16950700	-2.96715600
C	2.41485100	-0.06561500	-2.62049700
H	2.77644300	-0.36135000	-3.60810100
C	2.57432100	-1.27132300	-1.71278100
C	2.05622200	-2.50038700	-2.15922900
C	3.27778100	-1.25431600	-0.50815000
C	2.22446900	-3.66407900	-1.42567700
H	1.50559300	-2.54103300	-3.09601200
C	3.46847300	-2.42198200	0.23859800
H	3.69779100	-0.32757800	-0.13218700
C	2.93508100	-3.63422700	-0.21411900
H	1.80841700	-4.60752500	-1.76564200
H	4.02778400	-2.36990600	1.16486700
B	-1.13074900	-0.09961000	1.21280100
C	-1.71301100	1.29423100	1.71539100
C	-2.61164900	1.19996700	2.77903700
C	-1.39038000	2.58740800	1.31334200
C	-3.17716900	2.29827600	3.41077700
C	-1.94141500	3.71726400	1.91698000
C	-2.83360200	3.57408000	2.97241300
C	0.15168000	-0.60012600	1.97778100
C	0.22881700	-1.87171900	2.55607600
C	1.27729000	0.20976300	2.15291300
C	1.36149500	-2.33445200	3.21203700
C	2.44316000	-0.23403900	2.76477800
C	2.48053300	-1.51452100	3.30181200
C	-1.97034600	-1.04766100	0.29230900
C	-3.28426200	-0.76633700	-0.11069600
C	-1.38665000	-2.15567400	-0.34745400
C	-3.96015700	-1.50025800	-1.07889200
C	-2.02527300	-2.90080700	-1.32741100
C	-3.32294500	-2.56542400	-1.70144900
F	-0.54385400	2.81549900	0.29134500
F	-1.60892400	4.94373300	1.48707900
F	-3.36264500	4.65358600	3.56087900
F	-4.03741700	2.14396600	4.42758500
F	-2.96122300	-0.03092000	3.21975100
F	1.29008400	1.46892000	1.67979000
F	3.53261800	0.54624500	2.81083600
F	3.60862500	-1.98150200	3.84988300
F	1.40673000	-3.57728800	3.71682100
F	-0.81896800	-2.71250700	2.48704200
F	-3.95366400	0.28501500	0.39186800
F	-5.19147400	-1.13989200	-1.46840600

F	-3.93947100	-3.24126900	-2.67212500
F	-1.38960900	-3.90989400	-1.94128100
F	-0.12162300	-2.50792500	-0.08315100
C	3.12472700	1.21857600	-2.25763400
C	2.86228100	1.95273500	-1.08654900
C	4.10790600	1.70269700	-3.12669000
C	3.56074400	3.11790000	-0.80345000
H	2.10216700	1.61217600	-0.39973600
C	4.82394700	2.87121000	-2.85422000
H	4.33135000	1.15835100	-4.04146700
C	4.55042600	3.58831900	-1.68253300
H	3.35402900	3.68098700	0.10201500
H	5.57834000	3.20813500	-3.55546400
O	5.17785500	4.73616500	-1.31057500
O	3.05252300	-4.82549900	0.43293600
C	6.18800800	5.26239500	-2.17028000
H	6.54908600	6.17125900	-1.68443800
H	7.02278800	4.55945200	-2.28857200
H	5.78323700	5.51684600	-3.15833200
C	3.82667000	-4.87309000	1.63123200
H	3.38288200	-4.26569300	2.42692800
H	3.82749100	-5.92019300	1.94173700
H	4.86008400	-4.54743200	1.45551100

TS2′

E(SMD/B3LYP-D3/6-31G(d)) = -3458.8838 au

H(SMD/B3LYP-D3/6-31G(d)) = -3458.312201 au

G(SMD/B3LYP-D3/6-31G(d)) = -3458.456497 au

E(SMD/ B3LYP-D3/def2-TZVP//SMD/B3LYP-D3/6-31G(d)) = -3460.331974 au

C	-0.69148800	4.04595800	-2.41608800
C	-0.21060800	2.75835600	-2.19066100
C	-1.09271900	1.67138300	-2.18424400
C	-2.45773200	1.86988900	-2.43980300
C	-2.95139300	3.15560200	-2.63889400
C	-2.05540800	4.22032000	-2.61645600
H	-0.02966400	4.90524300	-2.42287500
H	0.84717900	2.60358300	-2.03237300
H	-3.13368300	1.02100100	-2.44829400
H	-4.00699300	3.34197900	-2.80543900
F	-2.53019500	5.46981800	-2.80607200
C	-0.62158600	0.27892500	-1.91922000
O	0.00287600	-0.06634200	-0.85556500
O	-0.89467700	-0.55597600	-2.82871800
C	-0.52132300	-2.41548700	-2.77357400
H	-0.79346000	-2.41664800	-3.82543100
C	-1.54550000	-2.97708800	-1.90826800

C	-2.73371700	-3.46216400	-2.48681100
C	-1.42681400	-3.00680000	-0.50014400
C	-3.75315600	-4.00013300	-1.71116800
H	-2.85307400	-3.42720700	-3.56678600
C	-2.43767400	-3.52655300	0.28383600
H	-0.55138800	-2.58362500	-0.02663700
C	-3.60869100	-4.03488400	-0.31254600
H	-4.64753200	-4.37889400	-2.19081600
H	-2.35931300	-3.53247800	1.36612400
B	0.19205400	0.67298700	0.51125000
C	1.60252100	1.50501000	0.53002900
C	1.98936600	2.12261300	1.72070100
C	2.58863800	1.47729800	-0.45207900
C	3.24152300	2.68510300	1.93374500
C	3.86510600	2.00703700	-0.27881200
C	4.19762900	2.61539000	0.92394000
C	0.43714600	-0.57478700	1.55945300
C	-0.25422200	-0.83193000	2.74295200
C	1.47160200	-1.47620400	1.28610700
C	-0.00152700	-1.93809000	3.55376300
C	1.75474000	-2.59500100	2.06206800
C	1.00430600	-2.83161600	3.20834300
C	-1.16658600	1.53811700	0.78808300
C	-1.25347300	2.92740900	0.85800600
C	-2.39787000	0.88026100	0.83872800
C	-2.45966200	3.61819500	0.96297600
C	-3.62214200	1.52366600	0.95700400
C	-3.65337100	2.91271700	1.02104500
F	2.35202600	0.92397800	-1.66648200
F	4.78205800	1.90612900	-1.26076600
F	5.41995800	3.13467000	1.11031100
F	3.54671600	3.27062800	3.10411800
F	1.11307600	2.17333600	2.75038100
F	2.26374400	-1.28029600	0.21316800
F	2.74922300	-3.43562200	1.72700500
F	1.25725100	-3.90039700	3.97704200
F	-0.71909600	-2.14258400	4.67176100
F	-1.23181200	-0.00918000	3.17853200
F	-0.15328100	3.70344500	0.75516000
F	-2.47496100	4.96175200	0.97982800
F	-4.82278200	3.56115400	1.11430000
F	-4.76967600	0.82445000	0.99214400
F	-2.43932000	-0.46816500	0.73244800
C	0.92166500	-2.53076700	-2.52246700

C	1.81002700	-1.67736800	-3.19827000
C	1.46420400	-3.51438900	-1.67039900
C	3.17613300	-1.71948600	-2.96270600
H	1.41437000	-0.92308900	-3.87113700
C	2.83236200	-3.59970700	-1.46561500
H	0.81268100	-4.22805200	-1.17849700
C	3.69431500	-2.66649400	-2.06366500
H	3.82182500	-1.00123000	-3.45257900
H	3.24849200	-4.33979600	-0.79084600
O	-4.53313900	-4.52092700	0.54089600
O	4.99963100	-2.74792900	-1.71514900
C	5.87725300	-1.69703900	-2.13329800
H	5.50643700	-0.71594400	-1.80930100
H	6.83463200	-1.90258000	-1.65064400
H	6.01428600	-1.69707000	-3.22169400
C	-5.76473000	-5.02500600	0.01181100
H	-5.59144200	-5.87819300	-0.65498800
H	-6.34414500	-5.35159900	0.87702000
H	-6.31622600	-4.24254900	-0.52308400

1j

E(SMD/B3LYP-D3/6-31G(d)) = -1695.599638 au
H(SMD/B3LYP-D3/6-31G(d)) = -1695.265474 au
G(SMD/B3LYP-D3/6-31G(d)) = -1695.353299 au
E(SMD/ B3LYP-D3/def2-TZVP//SMD/B3LYP-D3/6-31G(d)) = -1696.305481 au

C	1.41168800	5.17858600	1.43000400
C	0.98624300	3.85560700	1.37583400
C	0.85526600	3.19813900	0.14265100
C	1.15698000	3.88190500	-1.04598600
C	1.58411700	5.20628400	-1.00537000
C	1.70242900	5.82754300	0.23377500
H	1.52063700	5.70758500	2.37087900
H	0.75143900	3.31802300	2.28830200
H	1.05877300	3.37824900	-2.00055000
H	1.82455500	5.75700200	-1.90867900
F	2.11613000	7.11163100	0.27736900
C	0.39648500	1.78340300	0.15590300
O	0.14806900	1.15382800	1.16666200
O	0.27285800	1.28270300	-1.09717400
C	-0.09958100	-0.11675200	-1.28280000
H	-0.11723700	-0.17902700	-2.37540100
C	-1.51311500	-0.41807100	-0.80379700
C	-2.44484600	0.61063100	-0.61809200
C	-1.93519800	-1.74636700	-0.65917200
C	-3.76034700	0.32308900	-0.26456600
H	-2.14841000	1.64508400	-0.75198200

C	-3.25114800	-2.04109500	-0.30933900
H	-1.23535100	-2.56004900	-0.81898300
C	-4.16455400	-1.00466400	-0.10711300
H	-4.47130300	1.12969700	-0.11816100
H	-3.56438200	-3.07405500	-0.19781600
C	1.01401600	-1.03458500	-0.79723100
C	2.07462800	-1.29961700	-1.67293700
C	1.04011700	-1.58690800	0.49015200
C	3.14520200	-2.09852000	-1.27746200
H	2.06604200	-0.87457300	-2.67326200
C	2.10313600	-2.39221700	0.89084000
H	0.23312700	-1.37776400	1.18066400
C	3.15656200	-2.64441300	0.00782100
H	3.96385800	-2.29555100	-1.96198300
H	2.11852200	-2.81452500	1.89018600
C	4.27591000	-3.55823400	0.41704400
C	-5.56421700	-1.31419100	0.33596200
F	4.02790700	-4.84698700	0.06758600
F	5.44916300	-3.22106400	-0.16844100
F	4.47890000	-3.55384500	1.75470400
F	-5.66864700	-1.36550700	1.68916800
F	-6.45148100	-0.38032400	-0.08167500
F	-5.99489800	-2.51164400	-0.12713900

I1″

E(SMD/B3LYP-D3/6-31G(d)) = -3903.91705 au
H(SMD/B3LYP-D3/6-31G(d)) = -3903.397725 au
G(SMD/B3LYP-D3/6-31G(d)) = -3903.548434 au
E(SMD/ B3LYP-D3/def2-TZVP//SMD/B3LYP-D3/6-31G(d)) = -3905.580718 au

C	0.97531500	-4.61753500	-1.36752900
C	0.39819900	-3.39032600	-1.06494000
C	0.38877700	-2.36406100	-2.02302400
C	0.94882000	-2.57937000	-3.29511600
C	1.54939800	-3.79525600	-3.59607700
C	1.55143400	-4.79053400	-2.62258800
H	0.99231100	-5.42930100	-0.64868800
H	-0.05682300	-3.24042800	-0.09574400
H	0.94386500	-1.78404400	-4.03189800
H	2.01484100	-3.97951300	-4.55807700
F	2.13354500	-5.96908000	-2.90702500
C	-0.17655300	-1.04007400	-1.71597000
O	-0.07939300	-0.40774700	-0.63367800
O	-0.82751600	-0.53523600	-2.73431000
C	-1.61205500	0.73758100	-2.74479200
H	-1.73647000	0.85208900	-3.82416400
C	-0.82013000	1.93345800	-2.25316700

C	0.55119700	2.01851500	-2.53072500
C	-1.46417000	3.01664900	-1.64782700
C	1.27128200	3.15841000	-2.19398300
H	1.06706100	1.18874700	-3.00061000
C	-0.75085900	4.17177500	-1.32826100
H	-2.52390800	2.96934100	-1.42404000
C	0.61577400	4.23995600	-1.59830100
H	2.33751400	3.20594600	-2.38963900
H	-1.25415200	5.00292500	-0.84771300
B	0.85894400	-0.48456500	0.68485700
C	-0.12230100	-0.99121100	1.88858400
C	0.25347900	-0.74087400	3.21118000
C	-1.38320900	-1.56545200	1.74729400
C	-0.55974600	-0.99038600	4.30899100
C	-2.24440700	-1.80957400	2.81545600
C	-1.83217300	-1.51825500	4.10822000
C	1.23271100	1.09248100	0.95273600
C	2.52068900	1.63323800	1.02674900
C	0.21799600	2.00882900	1.24295400
C	2.78159700	2.96340200	1.34867500
C	0.43309400	3.34034000	1.57753700
C	1.73254500	3.82315100	1.64049100
C	2.14541400	-1.42944100	0.37781700
C	2.56790100	-2.50282500	1.16108100
C	2.89782400	-1.22979500	-0.78161200
C	3.63416700	-3.33157000	0.81272700
C	3.94771700	-2.04042800	-1.18256300
C	4.32483400	-3.10566100	-0.36957500
F	-1.86064100	-1.92849500	0.52984400
F	-3.47041300	-2.31450300	2.59542400
F	-2.64083500	-1.75277000	5.14994200
F	-0.13588700	-0.72344300	5.55430600
F	1.47958200	-0.23425500	3.46855500
F	-1.07843000	1.62088600	1.22489000
F	-0.60233600	4.16442500	1.80596900
F	1.96743400	5.10752600	1.93198900
F	4.04329600	3.42046800	1.37955900
F	3.61938300	0.88044500	0.80651700
F	1.92714100	-2.83799800	2.30035800
F	3.98140100	-4.36015500	1.60238100
F	5.33650800	-3.90451500	-0.72917500
F	4.59166500	-1.81470500	-2.33818900
F	2.56879500	-0.21284500	-1.61664000
C	-2.96862400	0.44501100	-2.14155600

C	-3.15271300	0.33242600	-0.75860100
C	-4.04950800	0.20562100	-2.99985600
C	-4.38882100	-0.04209200	-0.23964500
H	-2.32992500	0.53115500	-0.09057200
C	-5.29539200	-0.14677700	-2.48626400
H	-3.91754500	0.29199300	-4.07498300
C	-5.45918900	-0.27824400	-1.10499000
H	-4.51469600	-0.16001600	0.83147100
H	-6.13156500	-0.32428400	-3.15458500
C	1.39143300	5.48649200	-1.28173300
C	-6.78113900	-0.73818600	-0.55707400
F	0.79427400	6.23308500	-0.32636400
F	1.53898200	6.28405900	-2.36882000
F	2.64444100	5.20186100	-0.84452900
F	-6.87790100	-2.09157200	-0.55329100
F	-7.82164800	-0.27947500	-1.29156900
F	-6.97764600	-0.33053900	0.71703900

I2″

E(SMD/B3LYP-D3/6-31G(d)) = -3903.896277 au
H(SMD/B3LYP-D3/6-31G(d)) = -3903.378056 au
G(SMD/B3LYP-D3/6-31G(d)) = -3903.529009 au
E(SMD/ B3LYP-D3/def2-TZVP//SMD/B3LYP-D3/6-31G(d)) = -3905.562226 au

C	3.26891300	-3.88428100	-1.38556900
C	2.14709500	-3.06056000	-1.30991600
C	2.02944300	-1.94459100	-2.14633200
C	3.03331400	-1.67386100	-3.08802100
C	4.17226400	-2.47151400	-3.15504900
C	4.26904600	-3.56003000	-2.29340300
H	3.38037500	-4.75359200	-0.74626400
H	1.35725800	-3.29644700	-0.61068000
H	2.92946000	-0.81856700	-3.74760200
H	4.97677000	-2.26292700	-3.85254400
F	5.37470700	-4.33691700	-2.35105000
C	0.85005400	-1.02123600	-2.08682800
O	0.41796000	-0.56483600	-0.92712400
O	0.28430000	-0.69317900	-3.13126500
C	-2.44916000	0.39735100	-2.08544400
C	-2.23605200	1.76010800	-1.76304700
C	-0.90054600	2.23464300	-1.89571400
C	-3.24011300	2.64622400	-1.28319500
C	-0.56221000	3.50670800	-1.46452900
H	-0.14107700	1.56614100	-2.28006700
C	-2.89956700	3.92352800	-0.88049300
H	-4.27709900	2.33486100	-1.26273900
C	-1.55821200	4.33559000	-0.94052900

H	0.46746000	3.84090600	-1.50603100
H	-3.65770900	4.59896900	-0.50106400
B	1.15693900	-0.27258800	0.37519500
C	0.88647900	-1.42813100	1.50989600
C	1.46798700	-1.28915300	2.77156800
C	-0.03415400	-2.46810800	1.40889000
C	1.20769100	-2.12919100	3.84605700
C	-0.32729000	-3.33915700	2.45800400
C	0.29359900	-3.16691300	3.68759400
C	0.38289100	1.05234900	1.00679200
C	0.97764000	2.20973200	1.50938600
C	-1.00838700	1.02050100	1.13843400
C	0.25702400	3.28036600	2.03777000
C	-1.76551600	2.05505200	1.67448200
C	-1.12781400	3.20496600	2.12085800
C	2.73202800	0.00351200	-0.00008600
C	3.81276200	-0.81093600	0.33320200
C	3.05113800	1.05154400	-0.86541300
C	5.10267900	-0.61969600	-0.15888100
C	4.32094200	1.29083900	-1.37279000
C	5.36265400	0.44196100	-1.01418100
F	-0.68702900	-2.71744800	0.24790100
F	-1.21396700	-4.33706900	2.29063800
F	0.02104800	-3.99288500	4.70949700
F	1.81283900	-1.94551700	5.03251100
F	2.34333200	-0.27886100	2.98719000
F	-1.70348300	-0.06272100	0.71809100
F	-3.11142800	1.97810000	1.71993200
F	-1.84006800	4.23758000	2.59352900
F	0.88680800	4.38908900	2.45657300
F	2.31804900	2.37050500	1.49652000
F	3.65318800	-1.89951400	1.11697300
F	6.08983600	-1.47086100	0.16877100
F	6.59617300	0.63767100	-1.50211600
F	4.55002800	2.31940600	-2.20792000
F	2.07447700	1.89512000	-1.28996800
H	-1.65352900	-0.06062200	-2.67521700
C	-3.52474600	-0.46436200	-1.73103900
C	-4.40238600	-0.24038300	-0.63565000
C	-3.67949300	-1.64307900	-2.50761500
C	-5.42541200	-1.13428800	-0.37519800
H	-4.22767300	0.58227800	0.04495400
C	-4.72202900	-2.52143200	-2.25716100
H	-2.98628900	-1.83927400	-3.31947200

C	-5.59235000	-2.26051100	-1.19438900
H	-6.08740600	-0.97512100	0.46921100
H	-4.85253200	-3.40768300	-2.86726000
C	-6.68665400	-3.24490300	-0.85675400
C	-1.18699600	5.71810100	-0.45586600
F	-2.03023100	6.15344700	0.50215800
F	0.06083200	5.74273900	0.05804300
F	-1.22488500	6.61551000	-1.46577100
F	-7.00529000	-4.03046200	-1.90482000
F	-6.30862900	-4.05601300	0.15643200
F	-7.81298600	-2.61338900	-0.46124200

I3$_{1j}$

E(SMD/B3LYP-D3/6-31G(d)) = -1175.91375 au
H(SMD/B3LYP-D3/6-31G(d)) = -1175.686222 au
G(SMD/B3LYP-D3/6-31G(d)) = -1175.751751 au
E(SMD/ B3LYP-D3/def2-TZVP//SMD/B3LYP-D3/6-31G(d)) = -1176.401956 au

C	-0.00107800	1.58844700	0.02343500
H	-0.00638600	2.67784100	0.00961400
C	1.28779600	0.99962700	0.08621700
C	2.38983100	1.82827400	-0.26649000
C	1.53611600	-0.34138000	0.49331400
C	3.67442300	1.31279500	-0.30251200
H	2.21161800	2.86378100	-0.54019400
C	2.82507800	-0.84099000	0.48156500
H	0.72907400	-0.95519700	0.87399300
C	3.88464700	-0.02121700	0.06392000
H	4.50954700	1.93785600	-0.59681600
H	3.02063700	-1.85594500	0.80896400
C	-1.28355500	0.98630900	-0.03253800
C	-2.40039900	1.82100600	0.25727800
C	-1.51286000	-0.37096900	-0.39340300
C	-3.68270100	1.30202100	0.26272500
H	-2.23381500	2.86613900	0.50040200
C	-2.80002500	-0.87781400	-0.40498300
H	-0.69212300	-0.99660800	-0.72191000
C	-3.87596900	-0.04635300	-0.06375800
H	-4.53164400	1.93281500	0.50151000
H	-2.97909400	-1.90644300	-0.69598400
C	-5.27796800	-0.61239100	-0.02159100
C	5.27553500	-0.61033800	-0.01279200
F	-5.60357600	-0.97012200	1.24001100
F	-6.18826400	0.29353400	-0.43109200
F	-5.40571000	-1.70510700	-0.79855100
F	5.44604300	-1.26161800	-1.18448800
F	6.23046500	0.33577100	0.06795900

F 5.49203600 -1.49983600 0.97705800

TS1″

E(SMD/B3LYP-D3/6-31G(d)) = -3903.892098 au
H(SMD/B3LYP-D3/6-31G(d)) = -3903.37581 au
G(SMD/B3LYP-D3/6-31G(d)) = -3903.519973 au
E(SMD/ B3LYP-D3/def2-TZVP//SMD/B3LYP-D3/6-31G(d)) = -3905.556943 au

C	2.86123100	-3.34138300	-2.33467400
C	1.71771800	-2.68205600	-1.89394100
C	1.28176600	-1.52729000	-2.55805400
C	1.96990000	-1.05098700	-3.68755200
C	3.12526600	-1.69288200	-4.11745500
C	3.55527500	-2.81994700	-3.42143700
H	3.22667200	-4.23448000	-1.83970000
H	1.17073900	-3.07141200	-1.04305900
H	1.62094800	-0.16030100	-4.19900600
H	3.70014600	-1.32965300	-4.96262100
F	4.68342700	-3.43587400	-3.82500700
C	0.11452500	-0.77209400	-2.06049900
O	-0.05841100	-0.42465200	-0.87676000
O	-0.73923900	-0.45201100	-2.98154700
C	-1.92481800	0.46441700	-2.84283000
H	-2.04153900	0.66749500	-3.90790900
C	-1.60122400	1.74444300	-2.09759900
C	-0.78598100	2.71612200	-2.69730100
C	-2.11614000	1.98763800	-0.82079600
C	-0.42321900	3.86485000	-1.99671800
H	-0.38892100	2.54852900	-3.69536500
C	-1.81001700	3.16161900	-0.14354700
H	-2.77443200	1.27199900	-0.34584000
C	-0.92536000	4.07405700	-0.70639200
H	0.25739400	4.58727900	-2.43703100
H	-2.24709900	3.35240900	0.82732300
B	1.07239000	-0.37763700	0.76883700
C	1.04233100	-1.71414300	1.67463000
C	1.75218100	-1.74808900	2.87531500
C	0.19166400	-2.79018100	1.45647000
C	1.67966600	-2.80377500	3.77506700
C	0.09148800	-3.87419200	2.32534500
C	0.83943900	-3.87893300	3.49637300
C	0.28424800	0.79889900	1.54364900
C	0.80219700	2.03969600	1.92597500
C	-1.02328300	0.55537100	1.97171500
C	0.05175900	3.01655100	2.57578500
C	-1.78566400	1.48309500	2.67227900
C	-1.25302100	2.73615800	2.95174100

C	2.51920800	-0.01040300	0.14834200
C	3.64159000	-0.84456000	0.18511400
C	2.67906100	1.13385500	-0.64317900
C	4.81189500	-0.58862200	-0.52308800
C	3.84802800	1.45397400	-1.32046800
C	4.92718600	0.57825800	-1.26665300
F	-0.59093600	-2.84394700	0.34350500
F	-0.73324900	-4.89761800	2.05109600
F	0.75109700	-4.90645900	4.35150400
F	2.39638400	-2.79187200	4.91003600
F	2.54478200	-0.70709500	3.21191700
F	-1.66676800	-0.61305100	1.69469500
F	-3.05976400	1.21343000	2.99885300
F	-2.01041500	3.68037500	3.52431700
F	0.56916500	4.23048900	2.81492400
F	2.08086500	2.37736200	1.67305200
F	3.62856300	-2.01676700	0.85004500
F	5.81157500	-1.48390700	-0.52087400
F	6.04600800	0.84160300	-1.95057000
F	3.92847100	2.57192500	-2.05775000
F	1.63610400	1.96737700	-0.84478600
C	-3.13458900	-0.28101700	-2.33796700
C	-3.14216900	-0.96529300	-1.10526300
C	-4.30601200	-0.23850100	-3.10020600
C	-4.30568500	-1.59386600	-0.67204400
H	-2.29682000	-0.98204900	-0.41948300
C	-5.47289100	-0.86611800	-2.66252100
H	-4.31151500	0.28576400	-4.05195100
C	-5.46968200	-1.54689800	-1.44738700
H	-4.31177700	-2.10809600	0.28459600
H	-6.36990600	-0.82630900	-3.27037700
C	-0.49306000	5.28754600	0.07764200
C	-6.70293900	-2.23579100	-0.91932400
F	-1.16377800	5.40062600	1.24773600
F	-0.67430700	6.43473100	-0.61449700
F	0.82585500	5.21666500	0.38855800
F	-7.74889200	-2.14634600	-1.76930000
F	-7.09806000	-1.70326800	0.26245200
F	-6.47677800	-3.55247700	-0.69419200

TS2′′

E(SMD/B3LYP-D3/6-31G(d)) = -3903.88822 au

H(SMD/B3LYP-D3/6-31G(d)) = -3903.374106 au

G(SMD/B3LYP-D3/6-31G(d)) = -3903.517789 au

E(SMD/ B3LYP-D3/def2-TZVP//SMD/B3LYP-D3/6-31G(d)) = -3905.550297 au

C	-3.57079900	1.84758700	-3.06041400

C	-2.24315300	1.48968400	-2.82920100
C	-1.93820100	0.39265200	-2.01517100
C	-2.97905900	-0.36915100	-1.46239000
C	-4.30963300	-0.04194500	-1.71494900
C	-4.57813700	1.06737800	-2.50659300
H	-3.82826300	2.71188100	-3.66365000
H	-1.44922000	2.08221300	-3.26467100
H	-2.75885400	-1.18331600	-0.78239700
H	-5.12314100	-0.62656000	-1.30071000
F	-5.86710100	1.39784400	-2.74919500
C	-0.53677700	-0.13336800	-1.83781900
O	0.11477500	0.00962600	-0.72668900
O	-0.06907700	-0.79920000	-2.77849900
C	0.85845100	-2.76767900	-2.25806500
H	0.76355000	-2.80509400	-3.33251000
C	-0.28670900	-3.23608500	-1.53691800
C	-1.39458100	-3.70429600	-2.29139200
C	-0.42038100	-3.10664100	-0.14021200
C	-2.60134800	-3.97841700	-1.67254900
H	-1.30076900	-3.80317200	-3.36814400
C	-1.61495100	-3.43587900	0.48654100
H	0.36728700	-2.65990700	0.44787100
C	-2.71608200	-3.80052500	-0.28575100
H	-3.46732700	-4.26735700	-2.25883600
H	-1.71014400	-3.30714300	1.55766700
B	0.00091300	1.02850500	0.41595200
C	0.09459000	2.57404200	-0.12897100
C	-0.06794900	3.62186500	0.77975700
C	0.43686700	2.97815300	-1.41666400
C	0.04739200	4.96551000	0.44765900
C	0.55478300	4.31285200	-1.80058000
C	0.36777600	5.31625800	-0.86020600
C	1.39596000	0.81072100	1.26021600
C	1.52687200	0.74502600	2.64754500
C	2.60593200	0.75077500	0.56338200
C	2.73505000	0.49580600	3.29794300
C	3.83581500	0.54984900	1.17770200
C	3.90229200	0.37729400	2.55325500
C	-1.38825100	0.67423600	1.21765300
C	-2.58427400	1.37569500	1.05515100
C	-1.51759200	-0.49363700	1.96584500
C	-3.80616800	0.96280500	1.58040700
C	-2.71402500	-0.94783200	2.51226100
C	-3.88142700	-0.22751800	2.29005800

F	0.62060700	2.07603600	-2.41747600
F	0.85329500	4.63249800	-3.07194500
F	0.48705600	6.60626900	-1.20779700
F	-0.12949200	5.92352400	1.37329000
F	-0.35970900	3.34000600	2.07099700
F	2.64327300	0.92238400	-0.77647800
F	4.96724300	0.58031500	0.45558900
F	5.08052600	0.12692200	3.14354400
F	2.77926200	0.37657900	4.63656600
F	0.45031700	0.88387800	3.45144400
F	-2.63924200	2.48541700	0.28729100
F	-4.92735600	1.65538000	1.32004400
F	-5.05108600	-0.66193100	2.77731800
F	-2.75963400	-2.10030000	3.20777800
F	-0.44575400	-1.30011000	2.17090100
C	2.20045700	-2.53665700	-1.78354800
C	3.03623900	-1.74823000	-2.61281000
C	2.74551400	-3.10126700	-0.60895200
C	4.36186200	-1.52972200	-2.28339000
H	2.61263600	-1.28768500	-3.49901300
C	4.07724000	-2.87768400	-0.27842800
H	2.15239000	-3.75490900	0.01673500
C	4.88393900	-2.10944400	-1.12172700
H	4.99438600	-0.92353300	-2.92275800
H	4.49565600	-3.31913400	0.61889100
C	6.35355100	-1.95613700	-0.81214100
C	-4.09182400	-3.81241500	0.32828400
F	7.05292900	-3.01701800	-1.28810900
F	6.58082700	-1.89841000	0.51805100
F	6.89092100	-0.85714500	-1.37623600
F	-4.05950900	-3.76844700	1.67335800
F	-4.79305900	-2.72695300	-0.09043000
F	-4.79585700	-4.90387200	-0.03691300

6-X= CF_3

E(SMD/B3LYP-D3/6-31G(d)) = -645.4498488 au
H(SMD/B3LYP-D3/6-31G(d)) = -645.32564 au
G(SMD/B3LYP-D3/6-31G(d)) = -645.37056 au
E(SMD/ B3LYP-D3/def2-TZVP//SMD/B3LYP-D3/6-31G(d)) = -645.7253413 au

C	4.77448700	-0.02184200	-0.00040900
C	3.56384400	-0.01012000	-0.00000600
C	2.13414200	0.00358300	0.00013200
C	1.43408900	1.22396000	0.00020700
C	1.41177300	-1.20688300	0.00024200
C	0.04180000	1.23553100	0.00032800
H	1.98604200	2.15829500	0.00015400

C	0.02288100	-1.19119900	0.00033200
H	1.94781500	-2.15042100	0.00022100
C	-0.66145600	0.02980500	0.00040600
H	-0.49329200	2.17861600	0.00040500
H	-0.53195700	-2.12460200	0.00046600
H	5.84415300	-0.03210700	-0.00097700
C	-2.16307600	0.00580700	-0.00004200
F	-2.70641100	1.24228100	0.00476200
F	-2.65231600	-0.64130000	-1.08744700
F	-2.65279200	-0.65005100	1.08186300

6-X= OMe

E(SMD/B3LYP-D3/6-31G(d)) = -422.9387294 au
H(SMD/B3LYP-D3/6-31G(d)) = -422.786318 au
G(SMD/B3LYP-D3/6-31G(d)) = -422.830037 au
E(SMD/ B3LYP-D3/def2-TZVP//SMD/B3LYP-D3/6-31G(d)) = -423.1047468 au

C	-4.16168500	-0.29278800	0.00018600
C	-2.95613100	-0.16674200	-0.00011400
C	-1.53498900	-0.01841300	-0.00012100
C	-0.69328600	-1.14413600	-0.00007200
C	-0.94262800	1.26308300	-0.00007900
C	0.69393300	-1.00914500	0.00001800
H	-1.13173200	-2.13771600	-0.00006700
C	0.43591600	1.40486000	-0.00003100
H	-1.57633300	2.14492500	-0.00009700
C	1.26758800	0.27128900	0.00004200
H	1.31218700	-1.89921100	0.00007300
H	0.89662300	2.38799900	-0.00001700
H	-5.22502300	-0.40406900	0.00030400
O	2.60200400	0.52111100	0.00017500
C	3.50102200	-0.58873700	-0.00004500
H	3.37268400	-1.20913100	-0.89611600
H	4.50427800	-0.15796300	-0.00008000
H	3.37283800	-1.20934800	0.89589500

6-X= NMe_2

E(SMD/B3LYP-D3/6-31G(d)) = -442.3901923 au
H(SMD/B3LYP-D3/6-31G(d)) = -442.196315 au
G(SMD/B3LYP-D3/6-31G(d)) = -442.242451 au
E(SMD/ B3LYP-D3/def2-TZVP//SMD/B3LYP-D3/6-31G(d)) = -442.5587897 au

C	4.55073000	0.00044700	0.00798600
C	3.33761100	0.00005000	0.00367000
C	1.91091000	-0.00020500	-0.00085400
C	1.18051900	-1.20521000	-0.01416900
C	1.18045000	1.20492900	0.00912100
C	-0.20618800	-1.21178500	-0.01779200
H	1.71559100	-2.15084900	-0.02218500
C	-0.20618400	1.21149800	0.00532000

H	1.71543500	2.15055500	0.02191200
C	-0.94667900	-0.00014100	-0.00969900
H	-0.71803200	-2.16635400	-0.03037900
H	-0.71815400	2.16599300	0.01780400
H	5.61966200	0.00064500	0.01065400
N	-2.32102300	-0.00014400	-0.01697200
C	-3.05250500	-1.25721500	0.03113100
H	-4.12279800	-1.04816300	0.05534700
H	-2.84758100	-1.88086500	-0.85009200
H	-2.80029600	-1.84034300	0.92785800
C	-3.05280700	1.25753800	-0.01079200
H	-4.12258300	1.04964500	-0.05827800
H	-2.85769800	1.84169600	0.90005000
H	-2.79153600	1.87960500	-0.87742200

6-X= NO_2

E(SMD/B3LYP-D3/6-31G(d)) = -512.916417 au
H(SMD/B3LYP-D3/6-31G(d)) = -512.794316 au
G(SMD/B3LYP-D3/6-31G(d)) = -512.839082 au
E(SMD/ B3LYP-D3/def2-TZVP//SMD/B3LYP-D3/6-31G(d)) = -513.1229176 au

C	4.41095300	0.00013300	0.00036600
C	3.20038400	-0.00015800	0.00003900
C	1.77261400	-0.00008200	-0.00014400
C	1.06265000	1.21782300	0.00011500
C	1.06259600	-1.21791800	-0.00051900
C	-0.32593000	1.22124600	0.00009300
H	1.60713400	2.15604300	0.00042200
C	-0.32600700	-1.22126200	-0.00044700
H	1.60698800	-2.15619600	-0.00079100
C	-1.00195000	0.00000000	-0.00013100
H	-0.88195400	2.15043600	0.00030600
H	-0.88203400	-2.15045300	-0.00057000
H	5.48086100	0.00039000	0.00065300
N	-2.46824900	0.00000400	0.00009800
O	-3.04912400	-1.08825200	0.00094300
O	-3.04901500	1.08838500	-0.00056100

TS4-Ar= 1a (*p*-FC_6H_4)-X= CF_3

E(SMD/B3LYP-D3/6-31G(d)) = -1345.919984 au
H(SMD/B3LYP-D3/6-31G(d)) = -1345.600217 au
G(SMD/B3LYP-D3/6-31G(d)) = -1345.678695 au
E(SMD/ B3LYP-D3/def2-TZVP//SMD/B3LYP-D3/6-31G(d)) = -1346.468823 au

C	3.51180800	-0.04437700	-0.49673100
C	3.59284600	-0.03803600	0.91363600
C	4.82090800	-0.00436500	1.56777900
C	5.98407600	0.00680600	0.80473700
C	5.95696600	-0.03119800	-0.58442200
C	4.72016700	-0.06498400	-1.22458700

C	1.07484400	0.73135600	-0.95952300
C	0.97679400	1.64584500	0.11237500
C	-0.19436800	2.36181000	0.34930100
C	-1.28257500	2.16833700	-0.49416700
C	-1.22051400	1.31603200	-1.59097200
C	-0.04608600	0.60710000	-1.81347800
H	1.82732000	1.81598900	0.76187600
H	-0.27278600	3.06033700	1.17635500
H	-2.08939400	1.19791800	-2.22958600
H	0.00301900	-0.08565000	-2.64890400
H	4.68626300	-0.09352000	-2.31059400
H	2.68361900	-0.09750700	1.50239300
H	4.88809500	-0.00381500	2.65100700
H	6.88778200	-0.03385100	-1.14249600
F	7.18152200	0.03940100	1.43722400
F	-2.43447200	2.83588900	-0.24775100
C	2.23809600	-0.11370400	-1.22055100
H	2.34110500	-0.40197300	-2.26542500
C	1.61044700	-2.13885500	-0.63309800
C	0.41628000	-2.24666400	-0.30093500
C	-0.91508900	-1.87306800	-0.01938300
C	-1.20881600	-1.12626500	1.14784000
C	-1.96293800	-2.16057700	-0.92773300
C	-2.47983400	-0.61127400	1.34801700
H	-0.41607300	-0.91666300	1.85794600
C	-3.23402400	-1.64546500	-0.71774500
H	-1.75224000	-2.75178000	-1.81318100
C	-3.49081300	-0.85410500	0.40847200
H	-2.68848700	-0.00312900	2.22247800
H	-4.02488600	-1.83984700	-1.43470000
H	2.56304400	-2.62644300	-0.73567300
C	-4.80679800	-0.15622900	0.55726700
F	-4.75162600	1.11290600	0.07038600
F	-5.18954900	-0.04948500	1.85276800
F	-5.80639100	-0.78191000	-0.10685800

I7-Ar= 1a (*p*-FC$_6$H$_4$)-X= CF$_3$

E(SMD/B3LYP-D3/6-31G(d)) = -1345.956931 au

H(SMD/B3LYP-D3/6-31G(d)) = -1345.634382 au

G(SMD/B3LYP-D3/6-31G(d)) = -1345.71639 au

E(SMD/ B3LYP-D3/def2-TZVP//SMD/B3LYP-D3/6-31G(d)) = -1346.505077 au

C	-3.45590600	-0.24715500	0.59716700
C	-3.47256800	-1.41658900	-0.17425400
C	-4.59866000	-1.77338600	-0.91972200
C	-5.70961100	-0.94200600	-0.88047200
C	-5.73441600	0.22591100	-0.12668000
C	-4.60002200	0.56424200	0.60978600
C	-1.32488800	1.15519700	0.62041900
C	-1.04259800	0.98272900	-0.74084000
C	-0.13820200	1.81591800	-1.40000800
C	0.48051700	2.82769700	-0.67691800
C	0.22225300	3.03747800	0.67159800

C	-0.68432600	2.19167500	1.31200900
H	-1.52826600	0.18692800	-1.29708500
H	0.09116700	1.68803500	-2.45304400
H	0.72357300	3.84280100	1.19880900
H	-0.88644700	2.33538200	2.37069500
H	-4.60317300	1.47624000	1.20169300
H	-2.59841000	-2.06073300	-0.19368000
H	-4.62193100	-2.67788800	-1.51912200
H	-6.62537300	0.84569000	-0.12096400
F	-6.80928100	-1.28160600	-1.59710700
F	1.36207800	3.64086000	-1.30960200
C	-2.22270900	0.18637800	1.38421100
H	-2.58347300	0.70968600	2.27978200
C	-1.42379800	-1.02077200	1.90026900
C	-0.17382500	-1.25842500	1.60916400
C	1.09927100	-1.13077300	1.08764900
C	1.45144300	-1.74791000	-0.15383900
C	2.10438000	-0.38086600	1.77549400
C	2.72378100	-1.60474000	-0.67518600
H	0.69913900	-2.31746300	-0.69023200
C	3.37182400	-0.24601700	1.23924800
H	1.85188300	0.09932700	2.71523300
C	3.69039700	-0.85248900	0.01306600
H	2.97514400	-2.06560200	-1.62524500
H	4.12136700	0.33967200	1.76186800
H	-1.98649200	-1.69823000	2.54800900
C	5.08180500	-0.75336800	-0.53054100
F	5.10743500	-0.79557200	-1.88452700
F	5.70071700	0.39066700	-0.15312600
F	5.87310000	-1.77437400	-0.10301900

TS5-Ester Ar= 1a (*p*-FC$_6$H$_4$)-X= CF$_3$

E(SMD/B3LYP-D3/6-31G(d)) = -1345.763487 au
H(SMD/B3LYP-D3/6-31G(d)) = -1345.441664 au
G(SMD/B3LYP-D3/6-31G(d)) = -1345.520502 au
E(SMD/ B3LYP-D3/def2-TZVP//SMD/B3LYP-D3/6-31G(d)) = -1346.302289 au

C	3.34523100	-0.05502800	-0.46130200
C	3.37242600	-0.18404400	0.94362900
C	4.57305900	-0.12355900	1.63787600
C	5.75057800	0.06289100	0.91548200
C	5.77029500	0.16280800	-0.47232500
C	4.56341900	0.08106600	-1.15734800
C	0.92965800	0.72517800	-0.95714500
C	0.79120100	1.51453500	0.20688200
C	-0.35719500	2.26341600	0.42837400
C	-1.37983000	2.21510300	-0.51537500
C	-1.27660600	1.47398200	-1.69090900
C	-0.12228200	0.73638100	-1.90404500
H	1.59787000	1.57556100	0.92582000
H	-0.47133100	2.88108300	1.31250800
H	-2.09429200	1.47830900	-2.40313800
H	-0.02860200	0.14197300	-2.80785200

H	4.55854200	0.14801100	-2.24146100
H	2.45303500	-0.37283300	1.48772700
H	4.61527300	-0.22989800	2.71639200
H	6.71414100	0.29268800	-0.99072100
F	6.91464600	0.12825900	1.58551000
F	-2.49714900	2.92580700	-0.29641900
C	2.10515600	-0.09024900	-1.24674900
H	2.26464900	-0.23607300	-2.31427400
C	1.70235500	-2.09598000	-1.04974200
C	0.53063700	-2.20634900	-0.65642700
C	-0.77767900	-1.92212400	-0.25222400
C	-0.99315800	-1.36782300	1.03271900
C	-1.84694300	-2.01406500	-1.17712300
C	-2.23715400	-0.84811400	1.35377200
H	-0.17337600	-1.31916900	1.74077500
C	-3.09183700	-1.51069400	-0.83708000
H	-1.67401900	-2.45162400	-2.15432600
C	-3.27084900	-0.90301400	0.41207000
H	-2.40454000	-0.38574400	2.32018900
H	-3.91361700	-1.55706000	-1.54278800
H	2.62503300	-2.58131900	-1.31062700
C	-4.57427300	-0.20800300	0.71918900
F	-4.50479800	1.09611500	0.36099100
F	-4.86885000	-0.24431700	2.03513500
F	-5.61118200	-0.75273000	0.05129100

I8-Ester Ar= 1a (*p*-FC_6H_4)-X= CF_3

E(SMD/B3LYP-D3/6-31G(d)) = -1345.780504 au
H(SMD/B3LYP-D3/6-31G(d)) = -1345.457236 au
G(SMD/B3LYP-D3/6-31G(d)) = -1345.533529 au
E(SMD/ B3LYP-D3/def2-TZVP//SMD/B3LYP-D3/6-31G(d)) = -1346.320682 au

C	-3.46882500	-0.36841800	0.59348500
C	-3.44404000	-1.43114900	-0.31876900
C	-4.59866600	-1.81495900	-1.00265400
C	-5.77488200	-1.11750700	-0.76045200
C	-5.83790400	-0.05796800	0.13770700
C	-4.67654300	0.30901900	0.81478700
C	-1.42316700	1.18790300	0.58201400
C	-1.21098900	1.12443500	-0.80174800
C	-0.39646600	2.05779900	-1.44259400
C	0.19849700	3.05507500	-0.67928000
C	0.00335100	3.15559100	0.69317000
C	-0.81369800	2.21313600	1.31750600
H	-1.68541300	0.34468400	-1.38923100
H	-0.22369300	2.02301300	-2.51325200
H	0.48023100	3.95606100	1.24912300
H	-0.97236100	2.27341500	2.39124800
H	-4.70873100	1.13578500	1.51946000
H	-2.51994200	-1.97188400	-0.50274100

H	-4.59541200	-2.63885400	-1.70875100
H	-6.77913500	0.45691100	0.30014200
F	-6.89901800	-1.48508300	-1.41613300
F	0.99168500	3.96158800	-1.29379100
C	-2.22018600	0.11036000	1.32036600
H	-2.53089900	0.52034200	2.28872000
C	-1.30552300	-1.06843000	1.68658400
C	-0.07377000	-1.10716800	1.31604100
C	1.22889000	-1.06878500	0.90790000
C	1.59647500	-1.63213100	-0.35364300
C	2.20328200	-0.40133100	1.71410200
C	2.90198900	-1.52369700	-0.78908900
H	0.84137000	-2.12730600	-0.95426000
C	3.50275000	-0.29898400	1.25877300
H	1.90365900	0.02801700	2.66355900
C	3.84065400	-0.85831900	0.01629300
H	3.20039400	-1.93729500	-1.74538600
H	4.25496900	0.21168000	1.84886900
H	-1.71720600	-1.88559700	2.28311500
C	5.28289600	-0.78336100	-0.44773800
F	5.37443900	-0.79783300	-1.79007900
F	5.88871700	0.33263700	-0.00250600
F	5.97389100	-1.84193300	0.02393300

TS4-Ester Ar= 1a (*p*-FC_6H_4)-X= NMe_2

E(SMD/B3LYP-D3/6-31G(d)) = -1142.860753 au
H(SMD/B3LYP-D3/6-31G(d)) = -1142.471197 au
G(SMD/B3LYP-D3/6-31G(d)) = -1142.549805 au
E(SMD/ B3LYP-D3/def2-TZVP//SMD/B3LYP-D3/6-31G(d)) = -1143.302088 au

C	3.31072600	-0.10719800	-0.51363500
C	3.44759200	0.05431800	0.88362400
C	4.70001300	0.15375000	1.48398400
C	5.83381600	0.07209100	0.68271600
C	5.75306300	-0.12076100	-0.69131100
C	4.49247800	-0.21569100	-1.27736000
C	0.86354600	0.63236700	-0.97486000
C	0.80307200	1.65911500	-0.00617400
C	-0.35426900	2.41163200	0.18498400
C	-1.46566900	2.14482400	-0.60433400
C	-1.44536400	1.17734900	-1.60222600
C	-0.28497600	0.43206300	-1.77744600
H	1.67225600	1.88672800	0.59984700
H	-0.40200300	3.19485000	0.93517400
H	-2.33183100	1.00054700	-2.20231600
H	-0.26823800	-0.34798000	-2.53346400
H	4.41819900	-0.36403500	-2.35167400
H	2.56122100	0.06504100	1.50926100
H	4.80732300	0.27496800	2.55733900

H	6.66197200	-0.19182700	-1.28041800
F	7.05618500	0.16820900	1.26293900
F	-2.60959200	2.85076900	-0.39402500
C	2.00939000	-0.25195500	-1.17420400
H	2.07424300	-0.65593000	-2.18323400
C	1.37019600	-2.17610000	-0.31205500
C	0.17477900	-2.25224600	0.03336100
C	-1.15993600	-1.87357600	0.27374700
C	-1.47570900	-0.96151200	1.31048600
C	-2.22050500	-2.29737800	-0.56269700
C	-2.74754800	-0.43848200	1.44678100
H	-0.68659600	-0.62612700	1.97660300
C	-3.49915700	-1.78465800	-0.42459100
H	-2.01524000	-3.00846800	-1.35803700
C	-3.79878500	-0.81232900	0.56647700
H	-2.92406800	0.28800400	2.23049800
H	-4.26466800	-2.11744400	-1.11505700
H	2.32171500	-2.67736700	-0.32150500
N	-5.05010200	-0.23931100	0.67056400
C	-6.05558700	-0.53386800	-0.34137400
H	-6.24087200	-1.61136100	-0.40218100
H	-6.99431600	-0.05317100	-0.06027200
H	-5.76547100	-0.17538400	-1.34192800
C	-5.18445000	1.01781500	1.40231700
H	-4.50009800	1.78725800	1.01563700
H	-6.20917100	1.37958400	1.30372200
H	-4.98326500	0.87908300	2.47059300

I7-Ester Ar= 1a (*p*-FC$_6$H$_4$)-X= NMe$_2$

E(SMD/B3LYP-D3/6-31G(d)) = -1142.894458 au

H(SMD/B3LYP-D3/6-31G(d)) = -1142.502078 au

G(SMD/B3LYP-D3/6-31G(d)) = -1142.581516 au

E(SMD/ B3LYP-D3/def2-TZVP//SMD/B3LYP-D3/6-31G(d)) = -1143.333338 au

C	-3.11099600	0.12245800	0.53053800
C	-3.19036100	-1.21926400	0.13546300
C	-4.24556400	-1.67856500	-0.65650700
C	-5.22437600	-0.77459100	-1.04568500
C	-5.18566700	0.56412400	-0.67275000
C	-4.12249300	1.00206800	0.11592000
C	-0.83995400	1.27055400	0.49156500
C	-0.38509100	0.64767400	-0.67843700
C	0.72681900	1.12975400	-1.36835300
C	1.37338500	2.25587000	-0.87646800
C	0.92890200	2.93207300	0.25132300
C	-0.18162200	2.42682100	0.93041500
H	-0.88327100	-0.24524700	-1.04234600
H	1.10735900	0.63276300	-2.25462700
H	1.45582500	3.81653300	0.59508800
H	-0.52186600	2.92686500	1.83406900
H	-4.07607700	2.04758000	0.41095600
H	-2.42225500	-1.91705000	0.45455700
H	-4.31559200	-2.71646700	-0.96634600

H	-5.97387500	1.23718500	-0.99499900
F	-6.25581700	-1.21158600	-1.81089500
F	2.49062000	2.70062900	-1.51204400
C	-1.94073800	0.65940600	1.35249200
H	-2.34142800	1.46542600	1.98178500
C	-1.39123500	-0.39777100	2.33454000
C	-0.21634200	-0.98361500	2.26508100
C	1.01157700	-1.07258300	1.60108300
C	1.27318500	-2.09081400	0.63787700
C	2.04369900	-0.12001400	1.82097500
C	2.42388100	-2.08409400	-0.12434900
H	0.52162400	-2.85624200	0.46492000
C	3.18976400	-0.10748700	1.04621300
H	1.89935800	0.64637400	2.57615600
C	3.40085600	-1.06367500	0.02156500
H	2.54387600	-2.84312400	-0.89039300
H	3.92814500	0.66436900	1.22954500
H	-2.09591400	-0.65436400	3.13036000
N	4.47866500	-0.96690600	-0.85411200
C	5.29751200	0.23542100	-0.82422100
H	4.65968200	1.12427300	-0.86092000
H	5.94683300	0.29746600	0.06511700
H	5.93900800	0.24195900	-1.71061100
C	5.19637600	-2.16427400	-1.27858900
H	4.52353200	-3.01535500	-1.38529600
H	5.66673400	-1.98290600	-2.25075600
H	5.98531400	-2.43993500	-0.55916200

TS5-Ester Ar= 1a (*p*-FC_6H_4)-X= NMe_2

E(SMD/B3LYP-D3/6-31G(d)) = -1142.723798 au
H(SMD/B3LYP-D3/6-31G(d)) = -1142.331825 au
G(SMD/B3LYP-D3/6-31G(d)) = -1142.409542 au
E(SMD/ B3LYP-D3/def2-TZVP//SMD/B3LYP-D3/6-31G(d)) = -1143.157429 au

C	3.22249100	-0.07006700	-0.49652400
C	3.35661300	0.26004000	0.87208000
C	4.61022900	0.41131300	1.44730100
C	5.73753000	0.22728000	0.64822300
C	5.65392900	-0.13018400	-0.69461400
C	4.39401900	-0.29663100	-1.25466500
C	0.75715900	0.54551900	-0.99598400
C	0.63986500	1.63807300	-0.09550000
C	-0.54838400	2.33839200	0.02334800
C	-1.63706000	1.94725400	-0.75755500
C	-1.56193800	0.91765400	-1.69039700
C	-0.37030200	0.21948600	-1.79731100
H	1.49547500	1.96295900	0.48256700
H	-0.64975300	3.17706900	0.70356900
H	-2.43092800	0.66441800	-2.28640800
H	-0.29654900	-0.60534300	-2.49899400
H	4.30713000	-0.58292800	-2.29890800
H	2.47517200	0.34320700	1.49725800
H	4.73061300	0.64987800	2.49862200

H	6.56098000	-0.28008000	-1.27019900
F	6.95300800	0.37825100	1.20352200
F	-2.80101200	2.60763400	-0.61764600
C	1.94485500	-0.24020000	-1.16210300
H	1.99107300	-0.79361000	-2.09656000
C	1.48690500	-2.36802500	-0.13807800
C	0.29923500	-2.26652800	0.17133200
C	-1.01562900	-1.85448700	0.38488400
C	-1.30933500	-0.88523800	1.38073000
C	-2.07851600	-2.29277000	-0.45425200
C	-2.55764600	-0.31793700	1.47452100
H	-0.51961700	-0.55990500	2.05001900
C	-3.33665600	-1.75161200	-0.35039300
H	-1.87929500	-3.05248200	-1.20381700
C	-3.61186100	-0.70766300	0.58937800
H	-2.73014800	0.44060700	2.22698300
H	-4.11194900	-2.10195600	-1.01948200
H	2.46729500	-2.79564800	-0.18784200
N	-4.82251400	-0.10218100	0.63276400
C	-5.88711700	-0.50171300	-0.28642200
H	-6.12231600	-1.56608800	-0.17744900
H	-6.78592900	0.07018800	-0.05864600
H	-5.60576600	-0.30820600	-1.32944000
C	-5.04817800	1.03938100	1.51993500
H	-4.34101800	1.84737700	1.30148300
H	-6.05948100	1.41433900	1.36494300
H	-4.94659700	0.75048300	2.57245900

I8-Ester Ar= 1a (*p*-FC_6H_4)-X= NMe_2

E(SMD/B3LYP-D3/6-31G(d)) = -1142.763591 au
H(SMD/B3LYP-D3/6-31G(d)) = -1142.368233 au
G(SMD/B3LYP-D3/6-31G(d)) = -1142.44874 au
E(SMD/ B3LYP-D3/def2-TZVP//SMD/B3LYP-D3/6-31G(d)) = -1143.197915 au

C	-3.20455100	-0.36672600	0.62336900
C	-3.17793000	-1.48679900	-0.21728200
C	-4.32746100	-1.91039600	-0.88743500
C	-5.50300100	-1.19514300	-0.70326800
C	-5.56978200	-0.07900800	0.12326700
C	-4.41226200	0.32768500	0.78451800
C	-1.20541100	1.22523800	0.55042500
C	-0.97124700	1.10191800	-0.82570400
C	-0.21332100	2.05031000	-1.51214800
C	0.30618100	3.12534300	-0.80112200
C	0.09149500	3.28579800	0.56183800
C	-0.66780000	2.32504400	1.23114200
H	-1.38657200	0.26064500	-1.37195200
H	-0.02652500	1.96795400	-2.57801300
H	0.50935000	4.14454400	1.07720900
H	-0.84016900	2.43297700	2.29913100

H	-4.44732500	1.19962200	1.43277900
H	-2.25404600	-2.04169900	-0.35265000
H	-4.32011200	-2.77840800	-1.53875100
H	-6.51060300	0.44839500	0.24283700
F	-6.62356700	-1.60043000	-1.34614900
F	1.04460200	4.04963900	-1.46075600
C	-1.95559100	0.14817200	1.33169600
H	-2.27892400	0.59036200	2.28225100
C	-1.02251800	-1.00046800	1.72311100
C	0.21571000	-1.07630200	1.34736700
C	1.48951600	-1.08969200	0.93086000
C	1.85922500	-1.71772400	-0.31919800
C	2.53189400	-0.43429300	1.69097000
C	3.13895500	-1.68118800	-0.77320300
H	1.08646800	-2.21513300	-0.89635000
C	3.81230400	-0.39536100	1.23879400
H	2.26741900	0.03942500	2.63071500
C	4.17367900	-1.01038600	-0.01848200
H	3.37658100	-2.15850900	-1.71450800
H	4.56474800	0.10941000	1.82989300
H	-1.44271900	-1.78626900	2.35357100
N	5.42675700	-0.95543800	-0.47172800
C	6.47931000	-0.26713700	0.29203700
H	6.23325100	0.79200700	0.41571000
H	6.60385000	-0.72912900	1.27608900
H	7.41811100	-0.34753600	-0.25197000
C	5.79594000	-1.56662400	-1.75830700
H	5.23381600	-1.10562800	-2.57646700
H	6.85869200	-1.40890700	-1.93151800
H	5.59808600	-2.64248900	-1.74322700

TS4-Ester Ar= 1a (*p*-FC_6H_4)-X= NO_2

E(SMD/B3LYP-D3/6-31G(d)) = -1213.388418 au
H(SMD/B3LYP-D3/6-31G(d)) = -1213.071743 au
G(SMD/B3LYP-D3/6-31G(d)) = -1213.148897 au
E(SMD/ B3LYP-D3/def2-TZVP//SMD/B3LYP-D3/6-31G(d)) = -1213.870205 au

C	3.25996200	-0.06164700	-0.49988100
C	3.34979400	-0.11454800	0.90910600
C	4.58205200	-0.17928000	1.55185900
C	5.73817600	-0.20621000	0.77808200
C	5.69966800	-0.18921100	-0.61153900
C	4.45901900	-0.12750500	-1.24088700
C	0.86455600	0.85988400	-0.90434000
C	0.82424100	1.73444000	0.20441600
C	-0.30283800	2.50450400	0.47663300
C	-1.40387700	2.40503200	-0.36723400
C	-1.40107000	1.58635600	-1.49145400

C	-0.26968000	0.82216300	-1.74961900
H	1.68831500	1.83561900	0.85022200
H	-0.33650500	3.17871400	1.32644200
H	-2.27694000	1.54349700	-2.13028500
H	-0.26254200	0.16055200	-2.61128200
H	4.41490300	-0.11336200	-2.32673000
H	2.44270500	-0.14432700	1.50338400
H	4.65749100	-0.22577600	2.63345000
H	6.62496500	-0.22468000	-1.17744000
F	6.93886500	-0.26789200	1.39951500
F	-2.50669300	3.14007500	-0.09505200
C	1.98252800	-0.02251500	-1.21472900
H	2.05477200	-0.28685000	-2.26813600
C	1.24503300	-2.05376800	-0.69111000
C	0.04336900	-2.08320400	-0.38386000
C	-1.28222500	-1.72053600	-0.09515200
C	-1.59097000	-1.04911400	1.11725400
C	-2.32793300	-1.97890100	-1.02108300
C	-2.87906800	-0.61654800	1.37355000
H	-0.79782400	-0.84769100	1.82895900
C	-3.61798100	-1.55453700	-0.76219600
H	-2.10180300	-2.49899300	-1.94612500
C	-3.88588300	-0.86970200	0.43152100
H	-3.11678500	-0.08114500	2.28430600
H	-4.41820200	-1.73770100	-1.46845900
H	2.17799900	-2.57597700	-0.79430000
N	-5.23571600	-0.40650200	0.69552300
O	-6.10980000	-0.62958900	-0.15358100
O	-5.45411500	0.19247400	1.75747300

I7-Ester Ar= 1a (*p*-FC_6H_4)-X= NO_2

E(SMD/B3LYP-D3/6-31G(d)) = -1213.427185 au

H(SMD/B3LYP-D3/6-31G(d)) = -1213.107723 au

G(SMD/B3LYP-D3/6-31G(d)) = -1213.187009 au

E(SMD/ B3LYP-D3/def2-TZVP//SMD/B3LYP-D3/6-31G(d)) = -1213.90705 au

C	-3.13100300	-0.35798600	0.62718600
C	-3.12779200	-1.47653100	-0.21629700
C	-4.28074000	-1.86577300	-0.90193200
C	-5.43781100	-1.11847500	-0.72970200
C	-5.48291800	-0.00328400	0.09936900
C	-4.32158200	0.36866300	0.77484700
C	-1.10542400	1.18972100	0.58387300
C	-0.85850800	1.07238400	-0.79074100
C	-0.08561800	2.01686600	-1.46582000
C	0.43703200	3.08382500	-0.74515200
C	0.21219200	3.23834900	0.61653600
C	-0.56225200	2.28085600	1.27374600
H	-1.27367000	0.23602000	-1.34477500
H	0.11334500	1.93573000	-2.52961000
H	0.63603800	4.08876400	1.14089400
H	-0.74004400	2.38361300	2.34141400
H	-4.33993600	1.24036900	1.42431400

H	-2.21763900	-2.05581300	-0.34106700
H	-4.28943900	-2.73158800	-1.55631200
H	-6.40982300	0.55049400	0.20901100
F	-6.56295300	-1.49026800	-1.38759800
F	1.18804200	4.00686300	-1.39420000
C	-1.87353500	0.11619300	1.35021700
H	-2.19716500	0.55763200	2.30178800
C	-0.96116600	-1.05719600	1.72659800
C	0.28079400	-1.16693000	1.35516400
C	1.56494700	-1.12897100	0.89218900
C	1.91139500	-1.72025000	-0.37176300
C	2.60459100	-0.48680200	1.64962000
C	3.20321600	-1.66490900	-0.84258400
H	1.13518200	-2.20485900	-0.95472200
C	3.89357600	-0.43638000	1.17012700
H	2.35516500	-0.03148800	2.60220200
C	4.19329400	-1.02488200	-0.07308500
H	3.46882800	-2.10327300	-1.79659600
H	4.67939500	0.05212500	1.73284900
H	-1.42370700	-1.83007200	2.34646200
N	5.54804000	-0.96776300	-0.57280700
O	6.40797200	-0.40162100	0.11941000
O	5.79268600	-1.48757900	-1.67245900

TS5-Ester Ar= 1a (*p*-FC$_6$H$_4$)-X= NO$_2$

E(SMD/B3LYP-D3/6-31G(d)) = -1213.225871 au
H(SMD/B3LYP-D3/6-31G(d)) = -1212.906501 au
G(SMD/B3LYP-D3/6-31G(d)) = -1212.981254 au
E(SMD/ B3LYP-D3/def2-TZVP//SMD/B3LYP-D3/6-31G(d)) = -1213.698427 au

C	-2.88791000	0.38942100	0.20933600
C	-2.61323700	-0.85769600	-0.39317800
C	-3.63955700	-1.64181800	-0.89881800
C	-4.94946000	-1.17468900	-0.79418400
C	-5.26734000	0.03332700	-0.18142100
C	-4.23132400	0.80123700	0.33649200
C	-0.62537400	1.68664400	0.06179300
C	-0.13026100	1.07858800	-1.11092400
C	1.04809900	1.52288200	-1.70044000
C	1.73465800	2.57873900	-1.10935600
C	1.27690400	3.21858800	0.04077000
C	0.10042000	2.76583900	0.61900700
H	-0.68014900	0.28191400	-1.59466100
H	1.43350600	1.07105000	-2.60789100
H	1.84035900	4.04533900	0.45947800
H	-0.26631300	3.24151800	1.52423700
H	-4.46045200	1.74253000	0.82816500
H	-1.59684900	-1.23238900	-0.42825400
H	-3.44871900	-2.60547500	-1.35867700
H	-6.30298600	0.34732500	-0.10990100
F	-5.94340300	-1.93228500	-1.28719200
F	2.87790300	3.00384800	-1.67256600
C	-1.85264200	1.27798800	0.74959900

H	-2.26298900	2.09153600	1.34753300
C	-1.39886500	0.29085400	2.45464100
C	-0.32822700	-0.29074100	2.22858400
C	0.86478900	-0.74215400	1.64765100
C	0.89199900	-1.95965700	0.92621200
C	2.00723900	0.09102100	1.68869300
C	2.03617600	-2.32480700	0.23412900
H	0.01145100	-2.59267700	0.90760600
C	3.14785500	-0.26945600	0.98647600
H	1.97680600	1.01955000	2.24567600
C	3.13750600	-1.46690700	0.27091300
H	2.07871900	-3.24626200	-0.33245400
H	4.02741600	0.36142100	0.98367300
H	-2.21099700	0.49451500	3.12914900
N	4.34939900	-1.84312800	-0.48843300
O	5.31142500	-1.07789500	-0.44550500
O	4.32310100	-2.89776500	-1.12108100

I8-Ester Ar= 1a (*p*-FC_6H_4)-X= NO_2

E(SMD/B3LYP-D3/6-31G(d)) = -1213.239592 au
H(SMD/B3LYP-D3/6-31G(d)) = -1212.918901 au
G(SMD/B3LYP-D3/6-31G(d)) = -1212.99531 au
E(SMD/ B3LYP-D3/def2-TZVP//SMD/B3LYP-D3/6-31G(d)) = -1213.711937 au

C	-3.14127200	-0.31659200	0.61386600
C	-3.11215500	-1.39809500	-0.27638900
C	-4.26917800	-1.81210800	-0.93744200
C	-5.45239500	-1.12620100	-0.69474000
C	-5.51977900	-0.04944800	0.18225800
C	-4.35585800	0.34743100	0.83777200
C	-1.08766300	1.23533200	0.53596400
C	-0.87144800	1.11516900	-0.84353100
C	-0.05426400	2.02114400	-1.51938900
C	0.53883200	3.04851900	-0.79575900
C	0.33930300	3.20540800	0.57109400
C	-0.48024100	2.29024700	1.23103300
H	-1.34647000	0.31437100	-1.40103900
H	0.12072300	1.94311300	-2.58740100
H	0.81401000	4.02841400	1.09506900
H	-0.64403900	2.39666200	2.30044200
H	-4.39163500	1.18682800	1.52705200
H	-2.18340400	-1.93124300	-0.45865100
H	-4.26304800	-2.65069000	-1.62598500
H	-6.46625400	0.45524200	0.34607300
F	-6.57857300	-1.52312300	-1.32859000
F	1.33407000	3.92857900	-1.44405100
C	-1.89112700	0.19127000	1.31644900
H	-2.19891800	0.63800200	2.26916900
C	-0.97762300	-0.97471600	1.72211500

C	0.24444500	-1.04115900	1.32840600
C	1.54087300	-1.06918500	0.88660600
C	1.85954300	-1.73947100	-0.33330000
C	2.55364200	-0.39184800	1.63138400
C	3.16356400	-1.73354800	-0.79151600
H	1.07515600	-2.24180500	-0.88805400
C	3.85466400	-0.38839100	1.16275100
H	2.28968900	0.11955400	2.55033200
C	4.12516800	-1.05893500	-0.03313900
H	3.44141500	-2.23150100	-1.71162200
H	4.64755600	0.11717000	1.69876900
H	-1.37853400	-1.75902300	2.36892700
N	5.52785700	-1.05221400	-0.53338300
O	6.37401100	-0.50596600	0.16803300
O	5.74437600	-1.59373800	-1.61335600

TS4-Ester Ar= 1a (*p*-FC$_6$H$_4$)-X= OMe

E(SMD/B3LYP-D3/6-31G(d)) = -1123.406788 au
H(SMD/B3LYP-D3/6-31G(d)) = -1123.059895 au
G(SMD/B3LYP-D3/6-31G(d)) = -1123.136935 au
E(SMD/ B3LYP-D3/def2-TZVP//SMD/B3LYP-D3/6-31G(d)) = -1123.84756 au

C	-3.07794000	-0.07054500	0.53816500
C	-3.21957900	-0.15756300	-0.86515100
C	-4.47374300	-0.24343900	-1.46338800
C	-5.60223800	-0.26064900	-0.65040600
C	-5.51393400	-0.21062100	0.73575600
C	-4.25177000	-0.12364200	1.31938800
C	-0.68019300	0.88320300	0.83741900
C	-0.69750900	1.74528300	-0.28057800
C	0.40734700	2.52712700	-0.61124500
C	1.54505500	2.45199100	0.18329700
C	1.60061400	1.64602600	1.31474600
C	0.49055900	0.87166900	1.63127900
H	-1.58892300	1.82483500	-0.89189900
H	0.39458900	3.18984700	-1.47087400
H	2.50516400	1.61727400	1.91336600
H	0.53161000	0.21833600	2.49831200
H	-4.17091300	-0.08168500	2.40255800
H	-2.33365300	-0.19401100	-1.49057700
H	-4.58574500	-0.31420500	-2.54069600
H	-6.41825300	-0.23848000	1.33537300
F	-6.82579000	-0.34430300	-1.22854700
F	2.62856800	3.19809300	-0.14633500
C	-1.77198600	-0.01716500	1.20276100
H	-1.81144200	-0.24394100	2.26699000
C	-1.01423000	-2.01383700	0.69002800
C	0.17836500	-2.07076900	0.33837700
C	1.49694400	-1.70722800	-0.00564300
C	1.76473600	-1.03404100	-1.22590600
C	2.57931200	-1.94996900	0.86981200

C	3.04197300	-0.60057300	-1.52957300
H	0.94679300	-0.83274000	-1.91041200
C	3.86579700	-1.51740600	0.56581300
H	2.39665900	-2.46725100	1.80714300
C	4.10583000	-0.83195400	-0.63653800
H	3.24607100	-0.06682700	-2.45285300
H	4.66959500	-1.71139300	1.26632000
H	-1.93053300	-2.56194300	0.81797800
O	5.31611500	-0.35755200	-1.02754600
C	6.42853100	-0.52880900	-0.14870000
H	7.27939100	-0.06601600	-0.65259100
H	6.25968100	-0.02728800	0.81275900
H	6.64433000	-1.59073200	0.02511400

I7-Ester Ar= 1a (*p*-FC_6H_4)-X= OMe

E(SMD/B3LYP-D3/6-31G(d)) = -1123.443842 au

H(SMD/B3LYP-D3/6-31G(d)) = -1123.093951 au

G(SMD/B3LYP-D3/6-31G(d)) = -1123.170933 au

E(SMD/ B3LYP-D3/def2-TZVP//SMD/B3LYP-D3/6-31G(d)) = -1123.882026 au

C	-2.90128100	-0.08487100	0.62877800
C	-3.04104800	-1.25810000	-0.12398800
C	-4.18864400	-1.49687600	-0.88393900
C	-5.19741800	-0.54345200	-0.87783800
C	-5.09983900	0.63270600	-0.14302900
C	-3.94544700	0.85191200	0.60751100
C	-0.63758800	1.08056500	0.66569900
C	-0.34465700	0.85156400	-0.68484700
C	0.64961300	1.57747800	-1.34085200
C	1.34677100	2.54283400	-0.62592500
C	1.07763800	2.81226900	0.70958100
C	0.08157000	2.07108100	1.34703900
H	-0.88920900	0.08919200	-1.23317800
H	0.89149600	1.39868300	-2.38358200
H	1.64056300	3.58046700	1.23016600
H	-0.12845900	2.25945900	2.39712500
H	-3.85299200	1.76927400	1.18377100
H	-2.24727300	-1.99892800	-0.11421600
H	-4.30645900	-2.40367100	-1.46869800
H	-5.91406100	1.35023500	-0.16283300
F	-6.31776600	-0.76749800	-1.60877100
F	2.31945400	3.25008600	-1.25443600
C	-1.63732100	0.21938800	1.42992300
H	-1.95300900	0.79468100	2.31085300
C	-0.98492000	-1.06147700	1.98052700
C	0.20008600	-1.51784800	1.65563500
C	1.41561600	-1.44419600	0.97581900
C	1.62002100	-2.13976100	-0.25499100
C	2.50633800	-0.68661100	1.48719700
C	2.81722300	-2.04485600	-0.93500300
H	0.80907800	-2.73441400	-0.66520100
C	3.70909100	-0.59104900	0.79971800
H	2.37956400	-0.14991000	2.42225900

C	3.87480900	-1.26579900	-0.42238400
H	2.96542000	-2.55999400	-1.87962600
H	4.50709200	0.01454000	1.21378100
H	-1.61382000	-1.60333900	2.69258300
O	5.00243100	-1.23396000	-1.17971100
C	6.09706800	-0.43396900	-0.73250900
H	6.87522800	-0.54377100	-1.49059800
H	5.81443700	0.62346300	-0.65298100
H	6.48166900	-0.78316800	0.23419900

TS5-Ester Ar= 1a (*p*-FC_6H_4)-X= OMe

E(SMD/B3LYP-D3/6-31G(d)) = -1123.262571 au

H(SMD/B3LYP-D3/6-31G(d)) = -1122.913154 au

G(SMD/B3LYP-D3/6-31G(d)) = -1122.98721 au

E(SMD/ B3LYP-D3/def2-TZVP//SMD/B3LYP-D3/6-31G(d)) = -1123.694499 au

C	-2.94931200	-0.05797800	0.51271400
C	-3.09872100	-0.06204300	-0.89196400
C	-4.36027800	-0.04777700	-1.47020500
C	-5.47615400	-0.03305300	-0.63473700
C	-5.37452600	-0.06076100	0.75300400
C	-4.10636800	-0.09479900	1.31989900
C	-0.51971100	0.76737000	0.83714100
C	-0.49460000	1.65223400	-0.26894000
C	0.62963600	2.41573900	-0.54370500
C	1.74219700	2.29274800	0.28752400
C	1.75524200	1.46219000	1.40517500
C	0.62534400	0.70484400	1.67106200
H	-1.37176100	1.77645700	-0.89110800
H	0.65903600	3.10579600	-1.38000900
H	2.63927200	1.41319000	2.03074000
H	0.61769600	0.03753700	2.52761900
H	-4.00400300	-0.12543200	2.40079200
H	-2.22360400	-0.12440500	-1.52928500
H	-4.49586600	-0.06277300	-2.54615500
H	-6.27330800	-0.06474000	1.36012100
F	-6.69885800	-0.01334400	-1.19363200
F	2.83606100	3.02126000	0.01214900
C	-1.65323100	-0.06134700	1.18037100
H	-1.68654400	-0.35262000	2.22763000
C	-1.15595400	-2.18657300	0.71281600
C	0.01311500	-2.16075200	0.31254000
C	1.30497500	-1.79817700	-0.06538900
C	1.52675000	-1.13704800	-1.30482900
C	2.39466400	-1.96985100	0.82722000
C	2.76981700	-0.63086500	-1.60801700
H	0.70081500	-1.01136000	-1.99661700
C	3.64895300	-1.48054900	0.51684400
H	2.23019000	-2.48038500	1.77066800
C	3.84201000	-0.78800300	-0.69883300
H	2.95258000	-0.10184300	-2.53743400
H	4.46577900	-1.61589100	1.21491900
H	-2.08453400	-2.68241700	0.91829100

O	5.00454600	-0.24938300	-1.08415900
C	6.14775000	-0.34513400	-0.21787700
H	6.95423100	0.16543500	-0.74512200
H	5.95573800	0.15617800	0.73720100
H	6.42350200	-1.39141000	-0.04650600

I8-Ester Ar= 1a (*p*-FC$_6$H$_4$)-X= OMe

E(SMD/B3LYP-D3/6-31G(d)) = -1123.294271 au

H(SMD/B3LYP-D3/6-31G(d)) = -1122.942243 au

G(SMD/B3LYP-D3/6-31G(d)) = -1123.017623 au

E(SMD/ B3LYP-D3/def2-TZVP//SMD/B3LYP-D3/6-31G(d)) = -1123.725853 au

C	-2.91363500	-0.37463200	0.63761400
C	-2.96076500	-1.41658600	-0.29697100
C	-4.15309300	-1.74736400	-0.94393000
C	-5.29507900	-1.01803600	-0.64124400
C	-5.28753100	0.02284200	0.28069400
C	-4.08873000	0.33723300	0.91792700
C	-0.86548600	1.15966600	0.60345600
C	-0.70572700	1.15265100	-0.78857800
C	0.05225600	2.13409500	-1.42699800
C	0.64575500	3.12313600	-0.65182400
C	0.50608200	3.16608000	0.72981100
C	-0.25408700	2.17439800	1.35071800
H	-1.17953400	0.37867500	-1.38460000
H	0.18270900	2.14245500	-2.50418800
H	0.98020600	3.96108700	1.29610200
H	-0.36983600	2.19114000	2.43141900
H	-4.06598600	1.15063100	1.63846200
H	-2.06224500	-1.98210600	-0.52667200
H	-4.20390700	-2.55443400	-1.66759900
H	-6.20420300	0.56438900	0.49063300
F	-6.45628200	-1.33312600	-1.26077300
F	1.38288600	4.07928800	-1.26429300
C	-1.61937300	0.04435200	1.32537100
H	-1.88241400	0.40895800	2.32584000
C	-0.71001800	-1.16188700	1.57592500
C	0.49692800	-1.24790300	1.12170700
C	1.75617400	-1.27976300	0.64126200
C	2.03738700	-1.83134000	-0.66241300
C	2.83591100	-0.71335400	1.40839600
C	3.30967000	-1.81344500	-1.15223700
H	1.22219500	-2.25228100	-1.24105100
C	4.11114300	-0.69401200	0.91257900
H	2.61631700	-0.29235600	2.38388500
C	4.36354400	-1.24744500	-0.37484400
H	3.55354100	-2.21739000	-2.12871900
H	4.91433800	-0.25772500	1.49270100

H	-1.11503600	-1.98465600	2.16851800
O	5.55185700	-1.28160700	-0.94137700
C	6.71324600	-0.74425600	-0.26115900
H	7.54332200	-0.91503300	-0.94550600
H	6.58600500	0.32719900	-0.08252900
H	6.88279900	-1.27773500	0.67834700

TS4-Ester Ar= 1d (*p*-OMeC$_6$H$_4$)-X= CF$_3$

E(SMD/B3LYP-D3/6-31G(d)) = -1376.508434 au
H(SMD/B3LYP-D3/6-31G(d)) = -1376.102823 au
G(SMD/B3LYP-D3/6-31G(d)) = -1376.192917 au
E(SMD/ B3LYP-D3/def2-TZVP//SMD/B3LYP-D3/6-31G(d)) = -1377.056367 au

C	2.86229100	-0.83800700	-0.43628400
C	3.82708000	-0.55647200	0.55303300
C	5.02601800	-1.26400700	0.63723100
C	5.29179200	-2.29648300	-0.27384800
C	4.33275200	-2.61422000	-1.25163200
C	3.14403000	-1.90604100	-1.32067800
C	1.36263400	1.28811200	-0.40847200
C	2.37674900	2.23135600	-0.15219900
C	2.10621700	3.59680700	-0.04774000
C	0.79442100	4.06438600	-0.20004200
C	-0.23348200	3.14401400	-0.47971700
C	0.04934200	1.79464000	-0.58578200
H	3.40750100	1.90746300	-0.06402100
H	2.92413300	4.28286400	0.14048300
H	-1.24520200	3.51663300	-0.61181000
H	-0.75896700	1.10011000	-0.79631300
H	2.41307200	-2.16961100	-2.08135300
H	3.62833800	0.21208700	1.29374300
H	5.73514200	-1.01300100	1.41765100
H	4.54405400	-3.42350000	-1.94442200
C	1.57480800	-0.15336700	-0.52610500
H	0.86704900	-0.64165600	-1.19186200
C	0.57273600	-0.90909500	1.26954600
C	-0.65829100	-1.04597200	1.19521700
C	-2.02403500	-1.07736200	0.85283600
C	-2.83562300	0.07352500	1.02565800
C	-2.62013400	-2.25578700	0.33791700
C	-4.18097600	0.04366900	0.68948500
H	-2.38989100	0.98157700	1.41864200
C	-3.96673900	-2.27613500	0.00378600
H	-2.01195600	-3.14513800	0.20626400
C	-4.75253800	-1.12988800	0.17942700
H	-4.79314200	0.92969600	0.82513700
H	-4.41358300	-3.18333100	-0.39015900
H	1.45314500	-1.00100300	1.88040400
C	-6.19009500	-1.13578100	-0.24157100
F	-6.34764100	-0.73142300	-1.53061000
F	-6.94814800	-0.30366800	0.51248600
F	-6.74486900	-2.36866000	-0.15899000

O	6.42518600	-3.04843600	-0.28739700
O	0.41523700	5.36759400	-0.11035200
C	1.41649700	6.34324400	0.17504100
H	0.89585100	7.30237100	0.21429400
H	1.89933000	6.15293400	1.14230400
H	2.18034100	6.37819800	-0.61241300
C	7.42670700	-2.77689500	0.69199700
H	8.23415700	-3.48575900	0.49723100
H	7.81318300	-1.75368200	0.59944900
H	7.04703200	-2.93218500	1.71014700

I7-Ester Ar= 1d (*p*-OMeC$_6$H$_4$)-X= CF$_3$

E(SMD/B3LYP-D3/6-31G(d)) = -1376.548344 au

H(SMD/B3LYP-D3/6-31G(d)) = -1376.140092 au

G(SMD/B3LYP-D3/6-31G(d)) = -1376.226794 au

E(SMD/ B3LYP-D3/def2-TZVP//SMD/B3LYP-D3/6-31G(d)) = -1377.090751 au

C	-3.29684500	-0.49968100	0.57174600
C	-3.61438000	-1.04164100	-0.68399700
C	-4.85767800	-0.82627500	-1.26925500
C	-5.82665900	-0.05674000	-0.60800900
C	-5.52873900	0.49172500	0.64699900
C	-4.27387900	0.26385400	1.21772200
C	-0.91998200	0.40110500	0.85215100
C	-0.84823100	0.95263500	-0.43599400
C	0.21054600	1.77179000	-0.80988600
C	1.24310300	2.05296200	0.09795600
C	1.15303900	1.56811700	1.40859400
C	0.07609200	0.75238700	1.76669300
H	-1.61477500	0.71670900	-1.16813900
H	0.27887400	2.17341600	-1.81673600
H	1.92114500	1.78884900	2.14088000
H	0.04363500	0.34325500	2.77432700
H	-4.05475200	0.69748800	2.19118200
H	-2.87775700	-1.64229000	-1.21116300
H	-5.10027500	-1.24838100	-2.24016800
H	-6.25744300	1.08707900	1.18524200
C	-1.92769100	-0.68642500	1.21262800
H	-2.07414400	-0.65803700	2.30176500
C	-1.35145800	-2.08720900	0.90344800
C	-0.11594300	-2.32045900	0.54024100
C	1.18955200	-1.94372500	0.23446500
C	1.54296000	-1.51287100	-1.07808600
C	2.19829500	-1.92478700	1.24308300
C	2.80095600	-0.99607000	-1.33341900
H	0.79168800	-1.53542000	-1.86028300
C	3.45559500	-1.41389500	0.97272800
H	1.95119300	-2.26955400	2.24205000
C	3.75536700	-0.92212600	-0.30721800
H	3.03945700	-0.61535500	-2.32148600
H	4.20095800	-1.36350300	1.75986600
H	-2.06381300	-2.90767500	1.01945400
C	5.09768500	-0.32206500	-0.58726800

F	5.02277500	0.69345100	-1.48280600
F	5.67262900	0.18347100	0.53389400
F	5.98022300	-1.21893000	-1.09910200
O	-7.01379800	0.09327800	-1.26067400
O	2.28036600	2.79088500	-0.38868800
C	-8.03403200	0.86582900	-0.63222700
H	-7.71389600	1.90370800	-0.47161600
H	-8.88330700	0.85423200	-1.31892700
H	-8.33970800	0.42774300	0.32700900
C	3.42771400	2.96455100	0.44035800
H	3.84419300	2.00425300	0.76583700
H	4.16513800	3.48469200	-0.17470300
H	3.19808000	3.57763300	1.32191800

TS5-Ester Ar= 1d (*p*-OMeC_6H_4)-X= CF_3

E(SMD/B3LYP-D3/6-31G(d)) = -1376.362661au
H(SMD/B3LYP-D3/6-31G(d)) = -1375.954009 au
G(SMD/B3LYP-D3/6-31G(d)) = -1376.040702 au
E(SMD/ B3LYP-D3/def2-TZVP//SMD/B3LYP-D3/6-31G(d)) = -1376.902024 au

C	-1.99987100	1.36944000	-0.04985100
C	-3.01778800	1.76210700	0.85267900
C	-3.49678100	3.05612300	0.86043300
C	-2.97241100	4.01548000	-0.03294700
C	-1.93309200	3.65671600	-0.90971900
C	-1.44927500	2.35512400	-0.89414400
C	-2.22678800	-1.22474400	-0.14943900
C	-3.62626100	-1.29592800	0.04486800
C	-4.28878800	-2.50712400	-0.00919000
C	-3.57824000	-3.70063300	-0.24965800
C	-2.18547300	-3.65213900	-0.45730000
C	-1.53597000	-2.42960100	-0.40933400
H	-4.20466400	-0.39268100	0.19275800
H	-5.36541800	-2.56148000	0.11640100
H	-1.61804200	-4.55312900	-0.65681100
H	-0.46025100	-2.39763200	-0.56129400
H	-0.64181800	2.08444200	-1.56963000
H	-3.40715900	1.05223000	1.57514200
H	-4.27234000	3.36377100	1.55424400
H	-1.50257100	4.37935900	-1.59232500
C	-1.45741000	0.01742800	-0.13414600
H	-0.57374700	-0.03540000	-0.76456600
C	-0.52927200	-0.12166000	1.64651800
C	0.69328900	-0.17428500	1.44900500
C	2.02518500	-0.18799700	1.00358600
C	2.70486700	-1.41392600	0.81895800
C	2.68542700	1.03522300	0.73109100
C	4.01818300	-1.41453200	0.36704700
H	2.19584500	-2.34738200	1.03467500
C	3.99371700	1.02243900	0.27454000
H	2.15833000	1.97203000	0.87759300
C	4.65479300	-0.20009100	0.09529700
H	4.54509100	-2.35067300	0.22290200

H	4.50700300	1.95370000	0.05685100
H	-1.32676300	-0.10594300	2.36671300
C	6.08235300	-0.16698700	-0.38779700
F	6.89083900	0.42833800	0.51927100
F	6.19212600	0.54501600	-1.53329200
F	6.57864600	-1.39729400	-0.62609400
O	-3.52001000	5.24151300	0.04600100
O	-4.31687100	-4.82355400	-0.27350600
C	-3.67167100	-6.07866300	-0.53221900
H	-4.46599100	-6.82612700	-0.50581400
H	-3.19765500	-6.08222300	-1.52033800
H	-2.92799300	-6.30656300	0.24008600
C	-3.03892100	6.27455700	-0.82510100
H	-3.63979600	7.15529200	-0.59402100
H	-1.98172100	6.49163100	-0.63334200
H	-3.17861300	6.00059800	-1.87722100

I8-Ester Ar= 1d (*p*-OMeC$_6$H$_4$)-X= CF$_3$

E(SMD/B3LYP-D3/6-31G(d)) = -1376.376689 au
H(SMD/B3LYP-D3/6-31G(d)) = -1375.966928 au
G(SMD/B3LYP-D3/6-31G(d)) = -1376.050021 au
E(SMD/ B3LYP-D3/def2-TZVP//SMD/B3LYP-D3/6-31G(d)) = -1376.910479 au

C	-3.39613900	-0.08774600	0.76738700
C	-3.54654300	-1.19882500	-0.07867700
C	-4.73729800	-1.42564300	-0.75746500
C	-5.81635900	-0.53891800	-0.60983600
C	-5.68304600	0.57267100	0.23467400
C	-4.48103200	0.78225800	0.91332300
C	-1.03404200	0.98495600	0.66581000
C	-0.83036200	0.73818300	-0.70634600
C	0.32069300	1.17671400	-1.33625200
C	1.29456600	1.89812700	-0.61268600
C	1.04084200	2.26305800	0.72415300
C	-0.12081300	1.81409000	1.34056400
H	-1.55167600	0.14757800	-1.26154500
H	0.51171300	0.95185100	-2.38046300
H	1.74830100	2.86334400	1.28320300
H	-0.29183000	2.05138000	2.38765600
H	-4.39222000	1.64672100	1.56692900
H	-2.72555300	-1.89951200	-0.20693200
H	-4.85507300	-2.28646400	-1.40866400
H	-6.49969900	1.27122600	0.37471700
C	-2.09436700	0.21487100	1.48284900
H	-2.32211200	0.78952100	2.38917100
C	-1.33471200	-1.03368900	1.91259000
C	-0.10251800	-1.08870200	1.49931600
C	1.19853000	-1.17501900	1.06114900
C	1.47530600	-1.69537600	-0.23802000

C	2.25015700	-0.58298600	1.82421400
C	2.76132000	-1.63060500	-0.74645200
H	0.66831200	-2.12532700	-0.82015300
C	3.52949600	-0.53203500	1.30412400
H	2.02868400	-0.17232900	2.80268100
C	3.77455000	-1.04378700	0.02046600
H	2.97880500	-2.01408100	-1.73613300
H	4.33618300	-0.08599600	1.87585600
H	-1.78336800	-1.82668700	2.51272600
C	5.16625500	-0.88983300	-0.54801000
F	5.34847300	-1.61132200	-1.66840100
F	5.41020600	0.40672500	-0.85308000
F	6.10278800	-1.26959000	0.34586900
O	-6.93495200	-0.84661700	-1.31663100
O	2.42573800	2.18470500	-1.28285400
C	-8.06635800	0.01747400	-1.20529900
H	-7.83196900	1.03252600	-1.55039800
H	-8.83459500	-0.41418600	-1.84988500
H	-8.43994000	0.05928700	-0.17432800
C	3.48183900	2.86976700	-0.59549400
H	3.80813500	2.30531600	0.28429700
H	4.30157500	2.93810600	-1.31092700
H	3.16704700	3.87668900	-0.29791500

TS4-Ester Ar= 1d (*p*-OMeC$_6$H$_4$)-X= OMe

E(SMD/B3LYP-D3/6-31G(d)) = -1154.000332 au
H(SMD/B3LYP-D3/6-31G(d)) = -1153.567491 au
G(SMD/B3LYP-D3/6-31G(d)) = -1153.648185 au
E(SMD/ B3LYP-D3/def2-TZVP//SMD/B3LYP-D3/6-31G(d)) = -1154.436482 au

C	2.83800200	0.02600300	-0.60134100
C	2.96937300	-0.05574100	0.80554000
C	4.21096000	-0.01162000	1.42086600
C	5.38377100	0.10020500	0.65402000
C	5.28550300	0.14820000	-0.74317600
C	4.02853300	0.10337900	-1.34797900
C	0.35054100	0.69922500	-0.94198800
C	0.21641800	1.51532000	0.20540600
C	-0.98825500	2.12755000	0.52335300
C	-2.11704600	1.95242500	-0.29290300
C	-1.99769700	1.19827300	-1.46839400
C	-0.78420000	0.58838700	-1.77256600
H	1.06955900	1.68533600	0.85254200
H	-1.08023300	2.74411000	1.41298500
H	-2.84415000	1.05462300	-2.12936500
H	-0.72022200	-0.02589300	-2.66730800
H	3.96987800	0.14348700	-2.43339300
H	2.08373000	-0.19335400	1.41751700
H	4.30142400	-0.07998400	2.50114900

H	6.17102800	0.22028600	-1.36436500
C	1.54928800	-0.05875200	-1.29422700
H	1.64209200	-0.24731700	-2.36323100
C	1.03725900	-2.14341300	-0.86737000
C	-0.13158100	-2.36736100	-0.49206500
C	-1.46113600	-2.08563300	-0.10697200
C	-1.72278200	-1.42892600	1.11638800
C	-2.55953500	-2.36091800	-0.96105600
C	-2.99982400	-0.98658500	1.44091900
H	-0.89876600	-1.22147700	1.79149900
C	-3.83252600	-1.92225600	-0.64031500
H	-2.38686400	-2.88328100	-1.89739600
C	-4.05978600	-1.20220700	0.54688100
H	-3.15253300	-0.44929500	2.36940000
H	-4.66963000	-2.09911500	-1.30920800
H	2.00475000	-2.58211300	-1.03912200
O	6.55057500	0.13888600	1.35564100
O	-3.26737200	2.54791700	0.13530700
O	-5.32846800	-0.74257000	0.72907900
C	-5.57770200	0.11269700	1.84629400
H	-6.61699600	0.43318300	1.74959100
H	-5.45103700	-0.42205500	2.79622300
H	-4.91999400	0.99029400	1.83041000
C	-4.43904900	2.39863100	-0.66673400
H	-5.22673100	2.94950300	-0.14831600
H	-4.29770900	2.82915700	-1.66660500
H	-4.73479000	1.34694600	-0.75949300
C	7.77172900	0.23321700	0.62526800
H	8.56716600	0.24830300	1.37352300
H	7.91413000	-0.63151000	-0.03603200
H	7.81646400	1.15569500	0.03169600

I7-Ester Ar= 1d (*p*-OMeC_6H_4)-X= OMe

E(SMD/B3LYP-D3/6-31G(d)) = -1154.033274 au

H(SMD/B3LYP-D3/6-31G(d)) = -1153.597567 au

G(SMD/B3LYP-D3/6-31G(d)) = -1153.680835 au

E(SMD/ B3LYP-D3/def2-TZVP//SMD/B3LYP-D3/6-31G(d)) = -1154.468197 au

C	-2.74063500	-0.20525400	0.79701400
C	-2.99027400	-1.22719700	-0.13241200
C	-4.18866000	-1.28592900	-0.83653400
C	-5.18046700	-0.31635800	-0.62693400
C	-4.95198300	0.70970800	0.30020700
C	-3.74107000	0.75154300	0.99606100
C	-0.39022300	0.79060200	0.84211100
C	-0.20625200	0.76268700	-0.54824900
C	0.82907200	1.46602000	-1.15218600
C	1.71798700	2.22346500	-0.37510200
C	1.53213000	2.28974400	1.01134000
C	0.48444700	1.57443900	1.59858900
H	-0.87050800	0.16747200	-1.16785800
H	0.98045300	1.42658500	-2.22692800
H	2.19271800	2.87790200	1.63826400

H	0.36626700	1.61328500	2.67960900
H	-3.57470100	1.55629700	1.70918700
H	-2.23610400	-1.99032000	-0.30378100
H	-4.37802500	-2.07897300	-1.55420100
H	-5.70056100	1.47090100	0.48821800
C	-1.41571600	-0.09120800	1.54516100
H	-1.63739600	0.37746000	2.51451400
C	-0.83455600	-1.47892700	1.87910500
C	0.32255900	-1.94248300	1.47237500
C	1.53415200	-1.81910400	0.79063000
C	1.68419600	-2.29175500	-0.54881700
C	2.67306200	-1.22716500	1.40440500
C	2.87504200	-2.13707800	-1.22926800
H	0.83496800	-2.75674400	-1.04086200
C	3.86910200	-1.06841500	0.71622200
H	2.58986300	-0.86378100	2.42395100
C	3.97908100	-1.51490800	-0.61181300
H	2.98020900	-2.47723800	-2.25537800
H	4.70454700	-0.58906800	1.21368300
H	-1.48959900	-2.09164300	2.50523300
O	-6.31962600	-0.46083300	-1.36243300
O	2.71739500	2.85358200	-1.05758400
C	-7.35679400	0.50179300	-1.19047000
H	-7.01902300	1.51220600	-1.45621600
H	-8.15890200	0.20150000	-1.86830500
H	-7.73800800	0.50624800	-0.16078400
C	3.71959300	3.52721400	-0.30224500
H	4.22389000	2.84396400	0.39442300
H	4.44407000	3.90023400	-1.02978200
H	3.30580200	4.37557200	0.25895100
O	5.09430700	-1.39674800	-1.38033200
C	6.22175800	-0.71320900	-0.83503100
H	6.97773400	-0.70890500	-1.62306400
H	5.97143800	0.32159800	-0.56717000
H	6.62096800	-1.23190600	0.04612700

TS5-Ester Ar= 1d (*p*-OMeC$_6$H$_4$)-X= OMe

E(SMD/B3LYP-D3/6-31G(d)) = -1153.865313 au

H(SMD/B3LYP-D3/6-31G(d)) = -1153.430238 au

G(SMD/B3LYP-D3/6-31G(d)) = -1153.510377 au

E(SMD/ B3LYP-D3/def2-TZVP//SMD/B3LYP-D3/6-31G(d)) = -1154.29278 au

C	2.74780500	-0.08972500	-0.54113700
C	2.91819500	-0.06423100	0.86256500
C	4.17243900	0.08482900	1.42334500
C	5.31047400	0.20991900	0.60078200
C	5.16692900	0.14974000	-0.79545500
C	3.90042700	-0.01883800	-1.34558400
C	0.25943200	0.53520200	-0.87994000
C	0.13747100	1.39057300	0.24323600
C	-1.03925100	2.06788000	0.50053900
C	-2.15356300	1.91112600	-0.34717400
C	-2.04352900	1.10701200	-1.49476100

C	-0.85618800	0.43371400	-1.74102100
H	0.98266700	1.55029000	0.90084700
H	-1.12784000	2.72595200	1.35906700
H	-2.87609300	0.98815800	-2.17656200
H	-0.78908600	-0.20854400	-2.61465300
H	3.79956500	-0.07208200	-2.42655700
H	2.06194100	-0.20685800	1.51320900
H	4.30753000	0.09378900	2.50014400
H	6.02658900	0.22469400	-1.45046800
C	1.44804700	-0.23976300	-1.19908000
H	1.52268200	-0.48533800	-2.25710900
C	1.11869900	-2.25331600	-0.74232200
C	-0.05460000	-2.39798000	-0.35282100
C	-1.36995200	-2.13870700	0.01062000
C	-1.63221700	-1.45124700	1.22405600
C	-2.44534200	-2.38369900	-0.89171800
C	-2.88976400	-0.94667000	1.49241500
H	-0.82065500	-1.28280800	1.92350900
C	-3.70325900	-1.90479500	-0.61459500
H	-2.25267600	-2.92449600	-1.81254900
C	-3.92826100	-1.14376500	0.55759600
H	-3.05978100	-0.38685800	2.40343700
H	-4.53105300	-2.05912600	-1.29868900
H	2.06848800	-2.72723700	-0.90890200
O	6.48415500	0.36365300	1.24841400
O	-3.27016600	2.57513600	0.01910100
O	-5.16393000	-0.64334000	0.68809800
C	-5.45752100	0.22346300	1.79938600
H	-6.48569600	0.55222300	1.64555200
H	-5.38166600	-0.32002000	2.74720400
H	-4.78584900	1.08803600	1.80441700
C	-4.43544700	2.47700800	-0.81195100
H	-5.19977600	3.07689700	-0.31575100
H	-4.24162400	2.88489200	-1.81070100
H	-4.77924100	1.44038100	-0.89356900
C	7.68716400	0.47218900	0.47852000
H	8.49087300	0.59981500	1.20542300
H	7.86582000	-0.43732800	-0.10738400
H	7.65529500	1.34247600	-0.18782300

I8-Ester Ar= 1d (*p*-OMeC_6H_4)-X= OMe

E(SMD/B3LYP-D3/6-31G(d)) = -1153.8859 au

H(SMD/B3LYP-D3/6-31G(d)) = -1153.448161 au

G(SMD/B3LYP-D3/6-31G(d)) = -1153.531065 au

E(SMD/ B3LYP-D3/def2-TZVP//SMD/B3LYP-D3/6-31G(d)) = -1154.31459 au

C	-2.70504000	-0.34773900	0.76563700
C	-2.83524200	-1.34681000	-0.21129200
C	-4.05179200	-1.57778900	-0.84333300
C	-5.17810500	-0.80823900	-0.51325300
C	-5.06451600	0.19338200	0.46183300
C	-3.83566200	0.40887100	1.08864600

C	-0.53367000	1.02447600	0.73304300
C	-0.42650900	1.07618000	-0.66523700
C	0.42186100	1.98619000	-1.28370800
C	1.18996900	2.87595900	-0.51545000
C	1.08694700	2.84363400	0.88199900
C	0.22970100	1.92030500	1.48647600
H	-1.01597500	0.40135900	-1.27875300
H	0.50365200	2.02849600	-2.36572200
H	1.65801100	3.52459800	1.50252000
H	0.16452500	1.89836300	2.57224700
H	-3.76004800	1.18971700	1.84198700
H	-1.97682600	-1.95610100	-0.48167300
H	-4.15239900	-2.35287100	-1.59726100
H	-5.91656600	0.80172200	0.74221300
C	-1.37680900	-0.03405000	1.44215600
H	-1.59891000	0.33333900	2.45213300
C	-0.56439900	-1.31527800	1.66344800
C	0.61805500	-1.48880000	1.17202400
C	1.85356600	-1.61375100	0.64446400
C	2.03850600	-2.17035200	-0.67336800
C	3.00427500	-1.15258400	1.37603600
C	3.28964000	-2.26040300	-1.20868100
H	1.16855200	-2.50890700	-1.22596700
C	4.25862300	-1.24079600	0.83463200
H	2.85813700	-0.72551400	2.36257800
C	4.41572500	-1.80138200	-0.46428800
H	3.46099000	-2.67248300	-2.19711100
H	5.11798700	-0.88773400	1.39024000
H	-1.01913300	-2.11081100	2.25688900
O	-6.32075600	-1.11243900	-1.18504500
O	1.99260900	3.72206100	-1.21596000
C	-7.49562200	-0.35350700	-0.90002600
H	-7.35113000	0.71096700	-1.12524400
H	-8.27526600	-0.75649200	-1.54972400
H	-7.80360900	-0.46616500	0.14743900
C	2.79117100	4.65356400	-0.48726600
H	3.50074000	4.14275200	0.17696600
H	3.34549400	5.22161600	-1.23719200
H	2.17125200	5.34128100	0.10225000
O	5.57756700	-1.93794400	-1.07160000
C	6.80269700	-1.51550800	-0.42499100
H	7.59359500	-1.76043700	-1.13300100
H	6.78230700	-0.43773400	-0.24075100
H	6.94904600	-2.06582200	0.50876900

TS4-Ester Ar= 1j (*p*-$CF_3C_6H_4$)-X= CF_3

E(SMD/B3LYP-D3/6-31G(d)) = -1821.534919 au

H(SMD/B3LYP-D3/6-31G(d)) = -1821.183305 au

G(SMD/B3LYP-D3/6-31G(d)) = -1821.279555 au

E(SMD/ B3LYP-D3/def2-TZVP//SMD/B3LYP-D3/6-31G(d)) = -1822.302342 au

C	2.59502500	-0.51466400	-0.30260100
C	3.45151700	-0.03602000	0.71394100
C	4.76733600	-0.47493800	0.80883200
C	5.25871500	-1.41503000	-0.10238900
C	4.42177100	-1.92372200	-1.10203600
C	3.10660900	-1.48585000	-1.19118400
C	0.67384400	1.24671100	-0.33406300
C	1.45256600	2.38146700	-0.02061800
C	0.88014400	3.64803100	0.04805300
C	-0.48431800	3.81828300	-0.19981000
C	-1.27517700	2.71030800	-0.53308400
C	-0.70144200	1.45077100	-0.60525200
H	2.51974600	2.28556400	0.13813400
H	1.49996200	4.50776200	0.28156300
H	-2.33231200	2.84000500	-0.74314300
H	-1.32041300	0.59778600	-0.86584700
H	2.45930400	-1.88984900	-1.96483100
H	3.07435500	0.66019900	1.45537800
H	5.40819400	-0.09980200	1.60013200
H	4.79823300	-2.66376400	-1.80112100
C	1.19263900	-0.11733600	-0.42609800
H	0.62165800	-0.74894200	-1.10138500
C	0.33931700	-1.03486300	1.36858500
C	-0.84097900	-1.39437400	1.25270400
C	-2.17095100	-1.68220700	0.88444700
C	-3.18365700	-0.70390000	1.04343400
C	-2.51959600	-2.94804300	0.35479900
C	-4.49237100	-0.98333600	0.67686900
H	-2.92387600	0.26838100	1.44904100
C	-3.83137500	-3.21855300	-0.00954100
H	-1.75164800	-3.70561900	0.23550100
C	-4.81958200	-2.23972300	0.15126500
H	-5.26319300	-0.22966900	0.80100200
H	-4.09223600	-4.19086200	-0.41454000
H	1.20691800	-0.95782500	1.99853400
C	-6.22261500	-2.51458400	-0.30328500
F	-6.41054400	-2.16636600	-1.60325600
F	-7.13757000	-1.82241900	0.41500700
F	-6.54398600	-3.82594100	-0.20741700
C	6.69353300	-1.84561900	-0.04114100
C	-1.11654200	5.16967100	-0.06206300
F	-0.23606100	6.17571000	-0.27505800
F	-1.63226800	5.36675700	1.18070300
F	-2.14254400	5.34425500	-0.92993100
F	7.48744600	-1.08646700	-0.84146700
F	7.20577500	-1.74987600	1.20849000

F	6.85962200	-3.12768200	-0.44562800

I7-Ester Ar= 1j (*p*-$CF_3C_6H_4$)-X= CF_3

E(SMD/B3LYP-D3/6-31G(d)) = -1821.577466 au
H(SMD/B3LYP-D3/6-31G(d)) = -1821.223391 au
G(SMD/B3LYP-D3/6-31G(d)) = -1821.315775 au
E(SMD/ B3LYP-D3/def2-TZVP//SMD/B3LYP-D3/6-31G(d)) = -1822.33716 au

C	-2.82742900	-0.02118100	-1.17314200
C	-3.14595400	1.11497500	-0.41996300
C	-4.30206000	1.15340000	0.35985700
C	-5.15274900	0.04752600	0.39268700
C	-4.85031800	-1.09398300	-0.35731300
C	-3.69525700	-1.12273200	-1.13221600
C	-0.41940800	-0.83087300	-1.25716000
C	-0.12297000	-0.54385600	0.08038100
C	1.01790100	-1.06569900	0.68754000
C	1.86266200	-1.90815000	-0.03632600
C	1.55312700	-2.24692000	-1.35697400
C	0.41951200	-1.70754200	-1.95893100
H	-0.76537300	0.12395100	0.64536200
H	1.26080700	-0.80067900	1.71073100
H	2.20518200	-2.91256700	-1.91453600
H	0.19795300	-1.94698700	-2.99580000
H	-3.45683800	-2.01367700	-1.70757700
H	-2.48729800	1.97761000	-0.44286800
H	-4.53712500	2.03846600	0.94216400
H	-5.51255800	-1.95363700	-0.33098200
C	-1.54674800	-0.11789700	-1.99692800
H	-1.78421200	-0.71557500	-2.88636500
C	-1.09094800	1.25620800	-2.52863600
C	0.01327900	1.86690800	-2.17736400
C	1.20920000	1.90415700	-1.45837200
C	1.28167200	2.54766700	-0.18822700
C	2.36745300	1.23324000	-1.94401800
C	2.41242100	2.41757400	0.59775000
H	0.41915400	3.09530000	0.17799200
C	3.48882600	1.09966700	-1.14232900
H	2.33514900	0.76413000	-2.92137500
C	3.50801100	1.66892500	0.13789400
H	2.44473500	2.87131300	1.58366900
H	4.33924200	0.52583500	-1.49211400
H	-1.78198700	1.71075500	-3.24193900
C	4.63381200	1.38185300	1.08200700
F	4.29031800	0.42252100	1.98404000
F	5.74766500	0.94535700	0.45427800
F	4.98178300	2.47325900	1.80911400
C	3.15516100	-2.38914800	0.55341900
C	-6.42786100	0.09821300	1.18199300
F	3.20305100	-2.25181400	1.89582400
F	4.22080700	-1.70669000	0.05102300
F	3.38503300	-3.69659200	0.27367100
F	-6.35903100	0.96845900	2.21621200

F	-7.48244000	0.48775200	0.41891100
F	-6.75660900	-1.11094500	1.69691100

TS5-Ester Ar= 1j (*p*-$CF_3C_6H_4$)-X= CF_3

E(SMD/B3LYP-D3/6-31G(d)) = -1821.377035 au
H(SMD/B3LYP-D3/6-31G(d)) = -1821.023309 au
G(SMD/B3LYP-D3/6-31G(d)) = -1821.113996 au
E(SMD/ B3LYP-D3/def2-TZVP//SMD/B3LYP-D3/6-31G(d)) = -1822.129833 au

C	2.70737200	-0.87793800	0.70440700
C	2.77184900	0.40444200	0.11995700
C	3.96412500	0.87108800	-0.41051800
C	5.10845800	0.06266700	-0.36961000
C	5.07056100	-1.19584000	0.22962600
C	3.87941700	-1.65397000	0.78657500
C	0.19642700	-1.54972700	0.57408700
C	-0.13439600	-0.81802900	-0.58665500
C	-1.39823200	-0.93012100	-1.15413400
C	-2.34109200	-1.78772000	-0.58519500
C	-2.02799400	-2.54651800	0.54928000
C	-0.77532400	-2.41961400	1.12833300
H	0.59768400	-0.18079100	-1.06482100
H	-1.65140800	-0.35111600	-2.03424800
H	-2.76473500	-3.21840100	0.97720300
H	-0.53316900	-2.98620100	2.02299100
H	3.84877000	-2.62900200	1.26419500
H	1.90148500	1.05012300	0.13374200
H	4.01491600	1.86378300	-0.84682500
H	5.96333700	-1.80900500	0.27309600
C	1.48110300	-1.46786300	1.24339500
H	1.65501900	-2.28837200	1.93640000
C	1.29202000	-0.15944100	3.00292900
C	0.32609100	0.53518500	2.68024000
C	-0.80620800	1.07966800	2.05296400
C	-0.66842500	2.10133900	1.08128600
C	-2.06671000	0.48195900	2.27878000
C	-1.76027500	2.46150100	0.30778100
H	0.29589800	2.57485900	0.93182100
C	-3.15124400	0.84514600	1.49373000
H	-2.16766600	-0.28580400	3.03736000
C	-2.98561300	1.80821500	0.49449100
H	-1.66191000	3.22470000	-0.45680700
H	-4.10983300	0.36145000	1.63180400
H	2.11401600	-0.47404700	3.61681100
C	-4.10648500	2.08689800	-0.47504400
F	-3.90734700	1.40710700	-1.63245000
F	-4.17557400	3.39629300	-0.79738500
F	-5.30868000	1.71686000	0.00445500
C	-3.73873700	-1.86283100	-1.14481900
C	6.37742300	0.59268200	-0.98390700
F	6.23653100	0.76802800	-2.31908100
F	6.70574800	1.79973800	-0.46699900
F	7.42933500	-0.22860700	-0.79399100

F	-4.19233200	-3.13502300	-1.17225700
F	-3.82338700	-1.36970500	-2.39454000
F	-4.60730000	-1.15682900	-0.37559800

I8-Ester Ar= 1j (*p*-$CF_3C_6H_4$)-X= CF_3

E(SMD/B3LYP-D3/6-31G(d)) = -1821.395837 au
H(SMD/B3LYP-D3/6-31G(d)) = -1821.040205 au
G(SMD/B3LYP-D3/6-31G(d)) = -1821.133168 au
E(SMD/ B3LYP-D3/def2-TZVP//SMD/B3LYP-D3/6-31G(d)) = -1822.152667 au

C	-2.86244600	-0.45792000	0.98709200
C	-3.10269500	-1.35007700	-0.06438600
C	-4.35468500	-1.40198900	-0.67606200
C	-5.37425000	-0.55479500	-0.23876400
C	-5.14575400	0.34079600	0.80971800
C	-3.89471600	0.38628300	1.41764200
C	-0.61337200	0.76646800	1.07885200
C	-0.50840000	0.96841500	-0.30345700
C	0.38252500	1.91023400	-0.81218600
C	1.17622500	2.65816600	0.06161700
C	1.07328000	2.47270700	1.44187000
C	0.18053500	1.52805000	1.94448700
H	-1.12188100	0.38951100	-0.98654200
H	0.46329400	2.06047900	-1.88377400
H	1.68837100	3.05701400	2.11802100
H	0.10624400	1.37507400	3.01787100
H	-3.71618600	1.08544200	2.23013500
H	-2.31305300	-2.00943600	-0.41295400
H	-4.53393900	-2.09504500	-1.49157500
H	-5.94004200	0.99786500	1.14793400
C	-1.50186200	-0.33497400	1.65823700
H	-1.66887100	-0.11341100	2.71956900
C	-0.75364300	-1.67437000	1.66136900
C	0.44584400	-1.78926500	1.21013900
C	1.73205300	-1.83134200	0.75676700
C	1.99833100	-2.11529400	-0.61936500
C	2.80445600	-1.52387200	1.65324600
C	3.30096000	-2.08873900	-1.07637100
H	1.17301000	-2.33985500	-1.28583200
C	4.09865400	-1.49984300	1.17560300
H	2.58378900	-1.30382500	2.69177700
C	4.33658900	-1.78384400	-0.17918500
H	3.52461000	-2.29414500	-2.11665200
H	4.92505300	-1.26088800	1.83504400
H	-1.25797800	-2.55078200	2.07527100
C	5.77305200	-1.80614300	-0.66787700
F	5.85434000	-1.61040500	-1.99565500

F	6.51122700	-0.85767900	-0.06158700
F	6.33429000	-3.00115100	-0.39067800
C	2.09981800	3.70767200	-0.48981700
C	-6.74094800	-0.65389700	-0.85500000
F	1.46467300	4.89054600	-0.67714100
F	2.61205900	3.34931600	-1.69081700
F	3.14359800	3.95198100	0.33547600
F	-7.41924700	0.51389000	-0.78406300
F	-7.50575000	-1.58611700	-0.23197500
F	-6.68516000	-1.00955400	-2.15948300

TS4-Ester Ar= 1j (*p*-$CF_3C_6H_4$)-X= OMe

E(SMD/B3LYP-D3/6-31G(d)) = -1599.026988 au
H(SMD/B3LYP-D3/6-31G(d)) = -1598.64848 au
G(SMD/B3LYP-D3/6-31G(d)) = -1598.739253 au
E(SMD/ B3LYP-D3/def2-TZVP//SMD/B3LYP-D3/6-31G(d)) = -1599.683003 au

C	2.30813300	-1.12298200	0.62010600
C	2.36450800	0.16267200	0.03523800
C	3.56396600	0.68810300	-0.42790600
C	4.74781600	-0.04880600	-0.30466600
C	4.72246200	-1.31151300	0.29594000
C	3.51948900	-1.83419200	0.75736100
C	-0.21514800	-1.75682400	0.46985000
C	-0.51815700	-1.06140100	-0.71898000
C	-1.80152300	-1.07986900	-1.25767000
C	-2.81560000	-1.80651100	-0.63143400
C	-2.53182900	-2.53777000	0.53046600
C	-1.25399900	-2.51217800	1.06606500
H	0.25423800	-0.50915000	-1.23969700
H	-2.01306900	-0.52584700	-2.16622500
H	-3.31420800	-3.11686500	1.01166600
H	-1.04612800	-3.06518500	1.97839000
H	3.50888900	-2.81550400	1.22456000
H	1.46792600	0.77026800	-0.01276600
H	3.58764700	1.68098100	-0.86595400
H	5.64071100	-1.87970300	0.40578800
C	1.07584000	-1.71140600	1.15105500
H	1.24697300	-2.56754700	1.80162500
C	0.68232500	-0.34646300	2.81270800
C	-0.17483600	0.51359300	2.54479600
C	-1.15140600	1.25324400	1.84736500
C	-0.79759100	2.39088900	1.08634100
C	-2.50847300	0.83958300	1.84173900
C	-1.74278800	3.07262300	0.32731300
H	0.23475700	2.72775000	1.08567700
C	-3.44813600	1.51019300	1.08251900
H	-2.79845400	-0.03572400	2.41325600
C	-3.07646700	2.63043300	0.31392600
H	-1.43518800	3.93559600	-0.25152500
H	-4.47846800	1.17391500	1.04603100

H	1.38187900	-0.73461500	3.53049500
C	-4.22049600	-1.75739500	-1.14454200
C	6.02978700	0.49468000	-0.85713200
F	6.07016900	1.84861300	-0.82374600
F	7.11306800	0.04526600	-0.17871900
F	6.21899800	0.13974800	-2.15623200
F	-4.84762300	-2.95486500	-1.03206500
F	-4.28861500	-1.39018900	-2.44493900
F	-4.98687300	-0.86819400	-0.45305700
O	-4.07445800	3.20761500	-0.39980300
C	-3.76368000	4.33714200	-1.21748100
H	-3.02152800	4.08370200	-1.98500100
H	-4.70088900	4.62012400	-1.70065600
H	-3.39672400	5.17843300	-0.61628000

I7-Ester Ar= 1j (*p*-$CF_3C_6H_4$)-X= OMe

E(SMD/B3LYP-D3/6-31G(d)) = -1599.064014 au
H(SMD/B3LYP-D3/6-31G(d)) = -1598.682686 au
G(SMD/B3LYP-D3/6-31G(d)) = -1598.772732 au
E(SMD/ B3LYP-D3/def2-TZVP//SMD/B3LYP-D3/6-31G(d)) = -1599.716104 au

C	-2.51502900	-0.61992200	0.88105800
C	-2.92045500	-1.14611000	-0.35244800
C	-4.15457900	-0.80417400	-0.90414500
C	-4.99959800	0.07325000	-0.22168300
C	-4.61139700	0.60495900	1.01199000
C	-3.37695600	0.25829100	1.55362700
C	-0.08905800	0.09403900	1.14551300
C	-0.00196300	0.66828800	-0.12813400
C	1.08072000	1.47327800	-0.47440700
C	2.08834400	1.72086200	0.45992100
C	1.99454700	1.18965100	1.74901200
C	0.90739300	0.38748100	2.08513700
H	-0.76949900	0.46591600	-0.86814000
H	1.15038000	1.89206300	-1.47293700
H	2.77454900	1.38819100	2.47724400
H	0.85081300	-0.04753400	3.07959900
H	-3.07377700	0.67833100	2.50932700
H	-2.26500300	-1.82696100	-0.88729400
H	-4.45587600	-1.21463700	-1.86264300
H	-5.26893600	1.28735400	1.54109600
C	-1.15387000	-0.94228100	1.48650700
H	-1.28198200	-0.93802000	2.57773200
C	-0.67728500	-2.35971200	1.11071800
C	0.50504200	-2.66986600	0.63443700
C	1.79079700	-2.32903100	0.21026800
C	2.04980900	-1.92921300	-1.13019200
C	2.89932300	-2.36059200	1.11026600
C	3.31276800	-1.51719500	-1.53319700
H	1.22804600	-1.90935600	-1.83962100
C	4.15528200	-1.95638000	0.70568600
H	2.73543700	-2.67712200	2.13603500
C	4.37494200	-1.50805600	-0.61259800

H	3.45974500	-1.18582000	-2.55470300
H	4.99085300	-1.95113000	1.39946800
H	-1.42799400	-3.13501700	1.28688800
C	3.32293400	2.46852800	0.05668100
C	-6.35367000	0.39619700	-0.78114300
F	3.10756300	3.30642400	-0.98416800
F	4.31906300	1.62331100	-0.33114700
F	3.83116100	3.20885100	1.07044500
F	-6.37287800	0.35183300	-2.13449100
F	-7.30217100	-0.47849000	-0.35496800
F	-6.77827200	1.62889300	-0.41474500
O	5.63690500	-1.09395600	-0.89111600
C	5.88761800	-0.49639500	-2.16368000
H	5.24491300	0.37673900	-2.32417500
H	6.93216800	-0.17886500	-2.14157600
H	5.74677500	-1.21526900	-2.98087500

TS5-Ester Ar= 1j (*p*-$CF_3C_6H_4$)-X= OMe

E(SMD/B3LYP-D3/6-31G(d)) = -1598.874848 au

H(SMD/B3LYP-D3/6-31G(d)) = -1598.493683 au

G(SMD/B3LYP-D3/6-31G(d)) = -1598.57991 au

E(SMD/ B3LYP-D3/def2-TZVP//SMD/B3LYP-D3/6-31G(d)) = -1599.51979 au

C	-0.06344800	0.18034700	-0.78811000
C	0.16365900	0.95141400	0.37842000
C	1.36292100	1.62286700	0.55395000
C	2.36995200	1.51864900	-0.41437700
C	2.17670700	0.75385500	-1.56529200
C	0.97680500	0.07697100	-1.74021000
C	-2.61343400	-0.31225300	-0.62020700
C	-3.00637800	0.88950800	0.01162900
C	-4.34032100	1.12089100	0.32177100
C	-5.30643500	0.16231000	0.00460300
C	-4.94675100	-1.02324800	-0.64746000
C	-3.61764000	-1.24943800	-0.96601800
H	-2.28242600	1.66726100	0.21397100
H	-4.63169600	2.05228300	0.79512600
H	-5.70431600	-1.75303600	-0.91051200
H	-3.33657200	-2.16642700	-1.47577800
H	0.83110200	-0.53945400	-2.62167100
H	-0.57025100	0.96831000	1.17498300
H	1.54085300	2.18746600	1.46260400
H	2.96985800	0.65648400	-2.29783500
C	-1.25140200	-0.60140700	-1.02509100
H	-1.17299700	-1.29180000	-1.86038200
C	-0.85016000	-2.58160000	0.27469600
C	0.37580500	-2.55846600	0.37896000
C	1.74481600	-2.27626000	0.39470600
C	2.59365900	-2.69564600	-0.66925700
C	2.29269100	-1.48774800	1.43619400
C	3.91415200	-2.31098300	-0.69745900
H	2.18470100	-3.30932600	-1.46531200
C	3.61662300	-1.09148200	1.40649200

H	1.65318000	-1.17334900	2.25380100
C	4.43797900	-1.48953600	0.32898900
H	4.57237500	-2.61126300	-1.50603900
H	4.00547600	-0.46667600	2.20036000
H	-1.84791300	-2.95225500	0.39272000
C	3.68257400	2.22469300	-0.20051300
C	-6.74150600	0.39366900	0.40262900
F	-6.97288400	-0.04261600	1.66365600
F	-7.60312500	-0.25869700	-0.40626400
F	-7.06700600	1.70396400	0.37300200
F	3.65772100	3.49061700	-0.67242800
F	4.00095600	2.30111300	1.11506300
F	4.70231300	1.58809300	-0.82010500
O	5.72515700	-1.15337200	0.19403700
C	6.35236200	-0.33208200	1.19495500
H	6.36076400	-0.84209400	2.16441100
H	7.37567600	-0.18607700	0.84740000
H	5.84976900	0.63450500	1.27955200

I8-Ester Ar= 1j (*p*-$CF_3C_6H_4$)-X= OMe
E(SMD/B3LYP-D3/6-31G(d)) = -1598.910293 au
H(SMD/B3LYP-D3/6-31G(d)) = -1598.526698 au
G(SMD/B3LYP-D3/6-31G(d)) = -1598.616866 au
E(SMD/ B3LYP-D3/def2-TZVP//SMD/B3LYP-D3/6-31G(d)) = -1599.558485 au

C	-2.38079200	-0.42786200	1.03814300
C	-2.67820300	-1.29554700	-0.01931100
C	-3.93197000	-1.26682900	-0.62901800
C	-4.89832200	-0.36361600	-0.18352400
C	-4.61465900	0.50690100	0.87300900
C	-3.36172600	0.47152100	1.47779200
C	-0.08032700	0.66979400	1.10020200
C	0.03287200	0.84224800	-0.28595900
C	0.94330600	1.75442500	-0.81259900
C	1.74921300	2.50608200	0.04732400
C	1.63968500	2.35041800	1.43050000
C	0.72810800	1.43281700	1.95013100
H	-0.59119600	0.26123200	-0.95767700
H	1.03037300	1.87937000	-1.88705800
H	2.26390500	2.93708800	2.09626200
H	0.64919200	1.30479600	3.02641800
H	-3.13913300	1.15289400	2.29456200
H	-1.93069900	-1.99871200	-0.37464800
H	-4.15406000	-1.94076100	-1.44988600
H	-5.36713900	1.20841200	1.21799800
C	-1.00617300	-0.38923900	1.69607500
H	-1.15234500	-0.15081900	2.75650100
C	-0.34268900	-1.76860400	1.68432200
C	0.81147000	-1.99743800	1.14930900

C	2.01666600	-2.17126400	0.57293400
C	2.11804900	-2.50586500	-0.82808200
C	3.22370700	-1.97067100	1.33375600
C	3.34216200	-2.62342600	-1.41560900
H	1.20730300	-2.65272400	-1.39871000
C	4.45083700	-2.09011000	0.74092900
H	3.14102200	-1.71433400	2.38463500
C	4.52423500	-2.41833200	-0.64328400
H	3.45253300	-2.86778600	-2.46639400
H	5.35289500	-1.93096200	1.31803000
H	-0.88419100	-2.59003200	2.15739500
C	2.69149400	3.52583200	-0.52572200
C	-6.26849500	-0.37063200	-0.79895300
F	2.07185800	4.70695700	-0.77096500
F	3.22244500	3.11807500	-1.70340200
F	3.72353000	3.79386600	0.30749000
F	-6.85569600	0.84724900	-0.74999100
F	-7.10369200	-1.22898500	-0.15987000
F	-6.24195600	-0.75313800	-2.09689400
O	5.65196700	-2.55333200	-1.30830400
C	6.92895300	-2.37189800	-0.64619800
H	7.04767300	-3.10756500	0.15387500
H	7.67362400	-2.53802300	-1.42351300
H	7.01169000	-1.35385700	-0.25566900

7. References

1 Santi, M.; Ould, D. M. C.; Wenz, J., Soltani, Y.; Melen, R. L.; Wirth, T. *Angew. Chem. Int. Ed*. **2019**, *58*, 7861–7865.

2 Harris, R. K.; Becker, E. D.; Cabral De Menezes, S. M.; Goodfellow, R.; Granger, P. *Solid State Nucl Magn Reson*. **2002**, *4*, 458–483.

3 La, M. T.; Kim, H. *Tetrahedron Letters*, **2018**, *59*, 1855–1859.

4 Dasgupta, A.; Stefkova, K.; Babaahmadi, R.; Ariafard, A.; Melen, R. L. *Angew. Chem. Int. Ed.* **2020**, *59*, 15492–15496.

5 Malkoch, M.; Thibault, R. J.; Drockenmuller, E.; Messerschmidt, M.; Voit, B.; Russell, T. P.; Hawker, C. J. *J. Am. Chem. Soc.* **2005**, *127*, 14942–14949.

6 Chen, Q.; Gao, F.; Tang, H.; Yao, M.; Zhao, Q.; Shi, Y.; Dang, Y.; Cao, C. *ACS Catal.* **2019**, *9*, 3730–3736.

7 Barluenga, J.; Andina, F.; Aznar, F.; Valdés, C. *Org. Lett.* **2007**, *9*, 4143–4146.

8 Sun, F.; Gu, Z. *Org. Lett.* **2015**, *9*, 2222–2225.
9 Mi, Z.; Tang, J.; Guan, Z.; Shi W.; Chen, H. *Eur. J. Org. Chem*. **2018**, 4479–4482.
10 Kabalka, G. W.; Yao, M.-L.; Borella, S. *Org. Lett*. **2005**, *8*, 879–881.
11 Xiang, S.-K.; Zhanga, L.-H.; Jiao, N. *Chem. Commun*. **2009**, 6487–6489
12 Zhou, L.; Ye, F.; Zhang, Y.; Wang, J. *J. Am. Chem. Soc*. **2010**, *32*, 13590–13591.
13 Correiaa, C. A.; Lia, C.-J. *Adv. Synth. Catal*. **2010**, *352*, 1446–1450.
14 Fisher, K. M.; Bolshan, Y. *J. Org. Chem*. **2015**, *80*, 12676−12685.
15 Dong, X.-Y.; Zhang, Y.-F.; Ma, C.-L.; Gu, Q.-S.; Wang, F.-L.; Li, Z.-L.; Jiang, S.-P.; Liu, X.-Y. *Nat. Chem.* **2019**, *11*, 1158–1166.
16 Yang, Q.; Zhou, Y.; Chen, J.; He, X.; Xu, J.; Kwong, F. Y.; Fan, B. *Eur. J. Org. Chem*. **2015**, *24*, 5330–5333.
17 Soltani, Y.; Dasgupta, A.; Gazis, T. A.; Ould, D. M. C.; Richards, E.; Slater, B.; Stefkova, K.; Vladimirov, V. Y.; Wilkins, L. C.; Willcox, D.; Melen, R. L. Radical reactivity of frustrated Lewis pairs with diaryl esters. *Cell Reports Physical Science* **2020**, *1*, 100016
18 Stoll, S.; Schweiger, A. *J. Magn. Reson.* **2006**, *178*, 42
19 Boeré, R. T.; Bond, A. M.; Cronin, S.; Duffy, N. W.; Hazendonk, P.; Masuda, J. D.; Pollard, K.; Roemmele, T. L.; Tran, P.; Zhang, Y. *New J. Chem.* **2008**, *32*, 214–3820.
20 M. Culcasi, G. Gronchi, P. Tordo, *J. Am. Chem. Soc.* **1985**, *107*, 7191–7193
21 Symons, M. C. R.; Tordo, P.; Wyatt, J. *J. Organomet. Chem.* **1993**, *443*, C29–C32.
22 Dalton, D. R.; Liebman, S. A.; Waldman, H.; Sheinson, R. S. *Tet. Lett.* **1968**, *.2*, 145–148.
23 David R. Dalton and Shirley A. Liebman, *J. Am. Chem. Soc.*, **1969**, *91*, 1194–1199.
24 Symons, M. C. R.; Tordo, P.; Wyatt, J. *J. Organomet. Chem.* **1993**, *443*, C29–C32.
25 De Jong, J.; Fleurke, K. H.; Van Hardeveld, R. *Recueil des Travaux Chimiques des Pays-Bas* **1966,** *85*, 284–290.
26 (a) Dalton, D. R.; Liebman, A. *Tetrahedron* **1970**, *26*, 3265-3270; (b) Atto, A.; Hudson, A.; Jackson, R. A.; Simmon, N. P. C. *Chem. Phys. Lett.*, **1975**, *33*, 477–478.
27 Kwaan, R. J.; Harlan, C. J.; Norton, J. R. *Organometallics* **2001**, *20*, 3818–3820.
28 Buick, A. R.; Kemp, T. J.; Stone, T. J. *J. Phys. Chem.*, **1970**, *74*, 3439–3444.
29 Bennett, E.; Howard, J. A. *Chem. Phys. Lett.*, **1971**, *9*, 460−462.
30 Gaussian 16, Revision C.01, Frisch, M. J.; Trucks, G. W.; Schlegel, H. B.; Scuseria, G. E.; Robb, M. A.; Cheeseman, J. R.; Scalmani, G.; Barone, V.; Petersson, G. A.; Nakatsuji, H.; Li, X.; Caricato, M.; Marenich, A. V.; Bloino, J.; Janesko, B. G.; Gomperts, R.; Mennucci,

B.; Hratchian, H. P.; Ortiz, J. V.; Izmaylov, A. F.; Sonnenberg, J. L.; Williams-Young, D.; Ding, F.; Lipparini, F.; Egidi, F.; Goings, J.; Peng, B.; Petrone, A.; Henderson, T.; Ranasinghe, D.; Zakrzewski, V. G.; Gao, J.; Rega, N.; Zheng, G.; Liang, W.; Hada, M.; Ehara, M.; Toyota, K.; Fukuda, R.; Hasegawa, J.; Ishida, M.; Nakajima, T.; Honda, Y.; Kitao, O.; Nakai, H.; Vreven, T.; Throssell, K.; Montgomery, J. A., Jr.; Peralta, J. E.; Ogliaro, F.; Bearpark, M. J.; Heyd, J. J.; Brothers, E. N.; Kudin, K. N.; Staroverov, V. N.; Keith, T. A.; Kobayashi, R.; Normand, J.; Raghavachari, K.; Rendell, A. P.; Burant, J. C.; Iyengar, S. S.; Tomasi, J.; Cossi, M.; Millam, J. M.; Klene, M.; Adamo, C.; Cammi, R.; Ochterski, J. W.; Martin, R. L.; Morokuma, K.; Farkas, O.; Foresman, J. B.; Fox, D. J. Gaussian, Inc., Wallingford CT, **2016**.

31 (a) Head-Gordon, M.; Pople, J. A.; Frisch, M. J. *Chem. Phys. Lett.* **1988**, *153*, 503−506; (b) Becke, A. D. *J. Chem. Phys.* **1993**, *98*, 5648–5652; (c) Lee, C.; Yang, W.; Parr, R. G. *Phys. Rev. B.* **1988**, *37*, 785–789; (d) Vosko, S. H.; Wilk, L.; Nusair, M. *Can. J. Phys.* **1980**, *58*, 1200–1211; (e) Stephens, P. J.; Devlin, F. J.; Chabalowski, C. F.; Frisch, M. J. *J. Phys. Chem.* **1994**, *98*, 11623–11627.

32 Grimme, S.; Antony, J.; Ehrlich, S.; Krieg, H. *J. Chem. Phys.* **2010**, 132, 154104−154119.

33 Hariharan, P. C.; Pople, J. A. *Theor. Chim. Acta J. A.* **1973**, *28*, 213−222.

34 Marenich, A. V.; Cramer, C. J.; Truhlar, D. G. *J. Phys. Chem. B.* **2009**, *113*, 6378−6396.

35 (a) Fukui, K. J. *Phys. Chem.* **1970**,*74*, 4161−4163. (b) Fukui, K. *Acc. Chem. Res.* **1981**, *14*, 363−368.

36 Weigend, F.; Furche, F.; Ahlrichs, R. *J. Chem. Phys.* **2003**, *119*, 12753–12762.
